# Monocyte adhesion to atherosclerotic matrix proteins is enhanced by Asn-Gly-Arg deamidation

**DOI:** 10.1038/s41598-017-06202-2

**Published:** 2017-07-18

**Authors:** Bamaprasad Dutta, Jung Eun Park, Subodh Kumar, Piliang Hao, Xavier Gallart-Palau, Aida Serra, Yan Ren, Vitaly Sorokin, Chuen Neng Lee, Hee Hwa Ho, Dominique de Kleijn, Siu Kwan Sze

**Affiliations:** 1 School of Biological Sciences, Nanyang Technological University, 60 Nanyang Drive, Singapore, 637551 Singapore; 2 Department of Surgery, Yong Loo Lin School of Medicine, National University of Singapore, Singapore, 119228 Singapore; 3 Department of Cardiac, Thoracic and Vascular Surgery, National University Heart Centre, National University Health System, Singapore, 119228 Singapore; 4 Department of Cardiology, Tan Tock Seng Hospital, 11 Jalan Tan Tock Seng, Singapore, 308433 Singapore

## Abstract

Atherosclerosis arises from leukocyte infiltration and thickening of the artery walls and constitutes a major component of vascular disease pathology, but the molecular events underpinning this process are not fully understood. Proteins containing an Asn-Gly-Arg (NGR) motif readily undergo deamidation of asparagine to generate isoDGR structures that bind to integrin α_v_β_3_ on circulating leukocytes. Here we report the identification of isoDGR motifs in human atherosclerotic plaque components including extracellular matrix (ECM) proteins fibronectin and tenascin C, which have been strongly implicated in human atherosclerosis. We further demonstrate that deamidation of NGR motifs in fibronectin and tenascin C leads to increased adhesion of the monocytic cell line U937 and enhanced binding of primary human monocytes, except in the presence of a α_v_β_3_-blocking antibody or the α_v_-selective inhibitor cilengitide. In contrast, under the same deamidating conditions monocyte-macrophages displayed only weak binding to the alternative ECM component vitronectin which lacks NGR motifs. Together, these findings confirm a critical role for isoDGR motifs in mediating leukocyte adhesion to the ECM via integrin α_v_β_3_ and suggest that protein deamidation may promote the pathological progression of human atherosclerosis by enhancing monocyte recruitment to developing plaques.

## Introduction

Endothelial dysfunction and inflammation promote leukocyte and lipid accumulation in the arterial intima, leading to the formation of atherosclerotic plaques which are the primary cause of cardiovascular disease (CVD)^1^. Plaque formation narrows the blood vessel lumen and can limit or even occlude oxygen supply to vital tissues, leading to myocardial infarction, stroke and/or fatal organ damage^2, 3^. Consequently, CVD is a leading cause of human morbidity and mortality worldwide^4, 5^, and there is an urgent need to better understand the molecular basis of atherosclerotic plaque formation so that this can be more effectively targeted with novel therapies and disease interventions.

Blood vessel walls are arranged in three concentric layers, the innermost *tunica intima*, the *tunica media* and outer *tunica adventitia*, which are separated by a sheet-like layer of extracellular matrix (ECM) proteins. The vascular ECM is comprised of glycoproteins that play major roles in host processes including haemostasis, thrombosis, inflammation, wound healing, angiogenesis and embryogenesis^6, 7^. During a conventional inflammatory response, circulating monocytes are recruited to cardiac tissues via cell surface integrin binding to RGD motifs in ECM proteins, but dysregulation of this process in CVD may enhance leukocyte infiltration of the blood vessel wall and promote vascular pathology^8^.

Protein deamidation is an irreversible, degenerative protein modification (DPM) that is strongly associated with the pathogenesis of several age-related human disorders including atherosclerosis^9–19^. Deamidation of asparagine (N) residues is a spontaneous process that leads to the formation of aspartic acid and isoaspartic acid isoforms from a succinimide intermediary^20, 21^. Blast analyses indicate that ~5% of all proteins contain at least one NGR sequence and that ~0.5% contain several of these motifs. However, not all NGR sites are equally susceptible to deamidation, and subsequent changes in protein function also depend on amino acid sequence, molecular structure, and microenvironmental conditions^22^. Indeed, while the majority of previous studies have reported that deamidation inhibits protein function^23^, in certain settings this modification may instead confer gain-of-function changes that mediate key pathological events in age-related human diseases.

Inflammatory processes such as atherosclerosis induce protein oxidation, which leads to structural changes that promote NGR deamidation^22, 24^. Rather than disrupting immune responses, deamidation of NGR has previously been identified as a gain-of-function change that generates an integrin-binding isoAsp-Gly-Arg (isoDGR) structure which mimics the cognate Arg-Gly-Asp (RGD) target sequence^21, 25–32^. Integrin-mediated adhesion of circulating monocytes to endothelial cells is an early event in atherosclerotic plaque formation^33^, and isoDGR formation has already been shown to promote leukocyte binding to endothelial cells during cancer development^34^. However, it remains unknown whether isoDGR motifs also contribute to the pathogenesis of human atherosclerosis. In a recent proteomic analysis of human carotid atherosclerotic plaques, we detected extensive deamidation of NGR motifs in several ECM component proteins including fibronectin (FN) and tenascin C (TNC)^35–38^, which have been strongly implicated in the pathophysiology of atherosclerosis and CVD^39–44^. We therefore hypothesized that deamidation of FN and TNC generates gain-of-function isoDGR motifs that mediate integrin binding and promote leukocyte recruitment to atherosclerotic plaque proteins. To test this hypothesis, we assessed the ECM adhesion capacity of mononuclear cells that express integrin α_v_β_3_. Using this approach, we demonstrate that as little as 5–8% deamidation of NGR motifs in FN and TNC can significantly enhance mononuclear cell adhesion to key ECM components. Our data indicate that ECM protein deamidation may promote plaque formation via recruitment of circulating monocytes due to gain-of-function structural changes that increase binding to integrin α_v_β_3_.

## Results

### Atherosclerotic plaque-derived ECM proteins exhibit highly deamidated NGR motifs

In a previous mass spectrometry-based proteomic analysis of carotid plaque tissues, we identified >4,500 individual proteins and multiple novel pathways implicated in the development and progression of human atherosclerosis^38^. Sample processing for this earlier investigation was conducted in ammonium acetate buffer (pH 6.5) which permits in-depth analysis of protein deamidation^45^, so in the current study we interrogated these data to identify that many key ECM proteins including FN and TNC displayed extensive deamidation at NGR motifs (Table 1). Most of the isoDGR-containing proteins identified displayed ~50% deamidation of NGR sites, and selected proteins including EMILIN1 and LOXL1 exhibited 100% deamidation of specific residues (N219 in EMILIN1 and N365 in LOXL1). Given that EMILIN1 and LOXL1 are involved in the regulation of vascular assembly and flexibility^46–51^, it is possible that deamidation leading to functional impairment of these proteins could promote atherosclerosis in human patients.

**Table 1:** IsoDGR-containing peptides identified by LC-MS/MS analysis of human carotid atherosclerotic plaques.

Accession	Gene	Protein	Most abundant modified peptide	Modified site	N#GR	NGR	Total	% N#GR
P01023	A2M	Alpha-2-macroglobulin	K.EQAPHCICAN#GR.Q	851	22	7	29	75.9
C9JF17	APOD	Apolipoprotein D	R.WYEIEKIPTTFEN#GR.C	86	59	9	68	86.8
P51452	DUSP3	Dual specificity protein phosphatase 3	R.AADFIDQALAQKN#GR.V	117	6	3	9	66.7
A0A0C4DFX3	EMILIN1	EMILIN-1	R.AVETAFN#GR.Q	219	10	0	10	100.0
FA9	F9	Coagulation factor IX	K.NCELDVTCNIKN#GR.C	138	6	1	7	85.7
P35555	FBN1	Fibrillin-1	R.DIDECLQN#GR.I	537	65	10	75	86.7
P35555	FBN1	Fibrillin-1	R.DIDECLQN#GRICN#N#GR.C	543	9	25	34	26.5
P35555	FBN1	Fibrillin-1	R.YCKDINECETPGICMN#GR.C	625	44	68	112	39.3
P35555	FBN1	Fibrillin-1	R.RPDGEGCVDENECQTKPGICEN#GR.C	2304	11	42	53	20.8
C9JC84	FGG	Fibrinogen gamma chain	R.VELEDWN#GR.T	288	73	156	229	31.9
P02751	FN1	Fibronectin	R.GNLLQCICTGN#GRGEWK.C	263	54	43	97	55.7
P02751	FN1	Fibronectin	K.QMLCTCLGNGVSCQETAVTQTYGGNSNGEPCVLPFTYN#GR.T	367	5	76	81	6.2
P02751	FN1	Fibronectin	R.CTCVGN#GRGEWTCIAYSQLR.D	501	9	16	25	36.0
P02751	FN1	Fibronectin	R.NSITLTNLTPGTEYVVSIVALN#GR.E	1432	29	91	120	24.2
P10915	HAPLN1	Hyaluronan and proteoglycan link protein 1	K.SRYDVFCFTSN#FN#GR.F	258	28	29	57	49.1
A0A0G2JIW1	HSPA1B	Heat shock 70 kDa protein 1B	K.LLQDFFN#GRDLNK.S	356	11	11	22	50.0
P11021	HSPA5	78 kDa glucose-regulated protein	K.N#GRVEIIANDQGNR.I	47	3	7	10	30.0
P11047	LAMC1	Laminin subunit gamma-1	R.ATAESASECLPCDCN#GR.S	345	6	30	36	16.7
Q08397	LOXL1	Lysyl oxidase homolog 1	R.LSVGSVYRPNQN#GRGLPDLVPDPN#YVQASTYVQR.A	365	12	0	12	100.0
Q15149	PLEC	Plectin	K.TLPN#GRDALDGPAAEAEPEHSFDGLRR.K	2765	30	2	32	93.8
P08567	PLEK	Pleckstrin	R.GCVVTSVESNSN#GR.K	305	10	3	13	76.9
PLMN	PLG	Plasminogen	R.YEFLN#GR.V	736	44	2	46	95.7
K7EKI8	PPL	Periplakin	R.SLLDLEN#GR.R	814	4	0	4	100.0
H7C2N1	PTMA	Prothymosin alpha (Fragment)	K.EVVEEAEN#GR.D	67	20	26	46	43.5
Q96B97	SH3KBP1	SH3 domain-containing kinase-binding protein 1	R.KEDGGWWEGQIN#GR.R	42	2	1	3	66.7
H0Y7S5	SHANK2	SH3 and multiple ankyrin repeat domains protein 2	-.FM#N#VPGGGAAAVMMTGYNN#GR.C	19	2	0	2	100.0
A0A0C4DG40	SYNE1	Nesprin-1	R.DLQ#DRLSQMN#GRWDR.V	8480	1	0	1	100.0
A0JNU9	TBC1D1	TBC1 domain family member 1	R.KQ#N#LDLLEQLQ#VAN#GR.I	369	1	0	1	100.0
J3QSU6	TNC	Tenascin	K.N#GRENFYQNWK.A	1849	3	3	6	50.0
J3QSU6	TNC	Tenascin	K.VEGYSGTAGDSMAYHN#GR.S	1936	14	62	76	18.4
O76076	WISP2	WNT1-inducible-signaling pathway protein 2	R.GALCLLAEDDSSCEVN#GR.L	103	15	82	97	15.5

**Figure 1:**
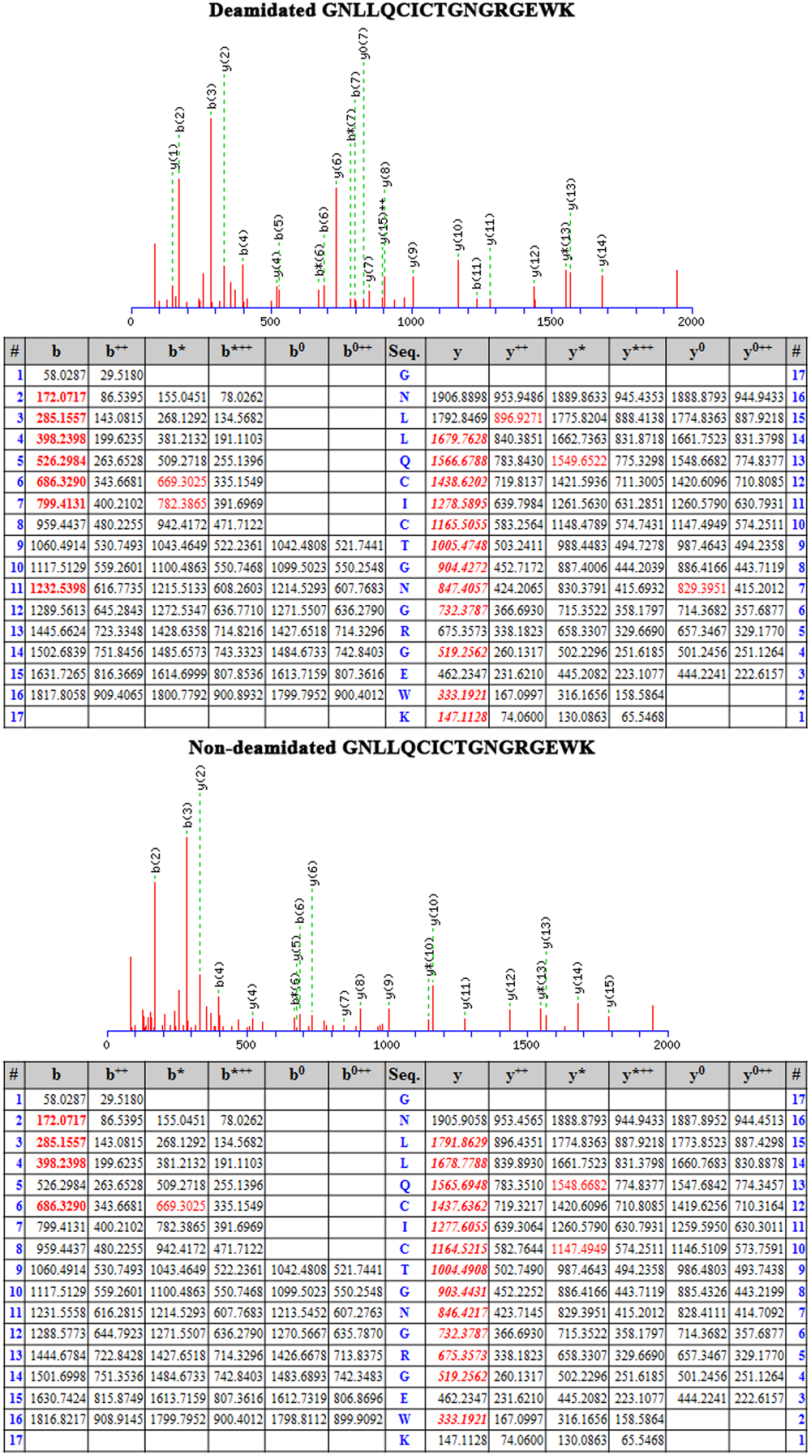
MS/MS spectra of FN peptides showing both the deamidated variant (GNLLQCICTG**N**#GRGEW) and unmodified form (GNLLQCICTG**N**GRGEW) as annotated by Mascot database search software (**a**). The deamidation site was identified with high confidence as being located between sequences (y1, y2, y4, y6) and (y7–y14). Each of the fragmented y-ions containing a deamidation site (y7–y14) displayed a characteristic mass increase of 0.984 Da compared with the unmodified peptide^45, 52^.

We and others have previously reported the optimal protein database search parameters for robust identification of deamidation sites. By applying a 5ppm precursor ion tolerance and #^13^C of 2 in Mascot searches, we prevented the C13 peaks of native peptides being misassigned as the monoisotopic peaks of the deamidated peptides^45, 52^. Numerous earlier studies have indicated that atherosclerotic disease is strongly associated with plaque accumulation of FN (which features NGR motifs at residues 263, 367, 501 and 1432)^42–44^, as well as TNC (which includes NGR motifs at residues 2031 and 2118)^40, 53–55^. In the current report, our use of optimized search parameters revealed that both FN and TNC exhibit significant deamidation of their respective NGR motifs. The deamidated and native peptides were identified with high confidence by Mascot protein database searches with extensive y-ions fragments (Figs 1 and S1 and Supplementary Data 1). The Mascot-identified peptides are included in Supplementary Data 2. Using a spectral counting quantitation method^56^, we observed that FN peptide GNLLQCICTGNGRGEW displayed 55.7% deamidation of the NGR motif, whereas TNC peptides NGRENFYQNWK and VEGYSGTAGDSMAYHNGR exhibited 50% and 18% deamidation respectively at the NGR domain (Table 1), which was significantly higher than the 6.4% basal deamidation level detected across the rest of the proteome. These data suggest that ECM proteins FN and TNC display elevated levels of deamidation in atherosclerotic plaques and that these post-translational modifications may promote disease pathogenesis.

### Monocyte adhesion to fibronectin and tenascin C is increased by NGR deamidation

**Figure 2:**
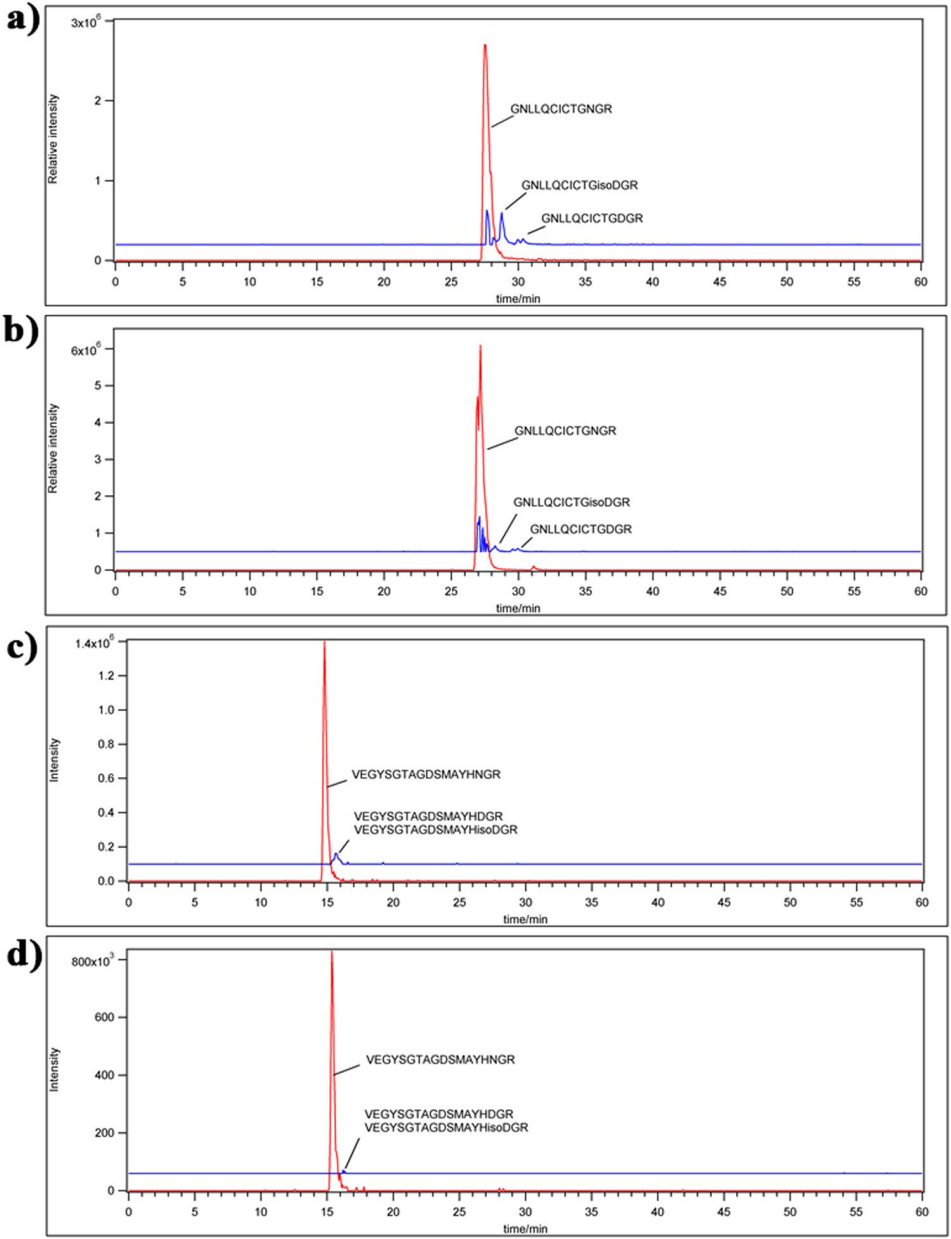
LC-MS/MS analysis of FN and TNC as extracted from cell culture plates treated with TEAB or PBS-only control. Shown are the FN peptide GNLLQCICTG**N**GRGEW native (**a**) and deamidated (**b**) forms as well as TNC peptide VEGYSGTAGDSMAYHNGR native (**c**) and deamidated (**d**) forms as identified by Mascot search of the MS/MS spectra. The percentage of deamidation of each targeted NGR motif was determined by integrating the area of the extracted ion chromatogram with +/− 5 ppm mass range of the calculated precursor masses of the native and deamidated peptides. TEAB-induced deamidation was 8% for FN and 5.3% for TNC, whereas only trace levels of protein deamidation were detected in the PBS-only control condition.

**Figure 3:**
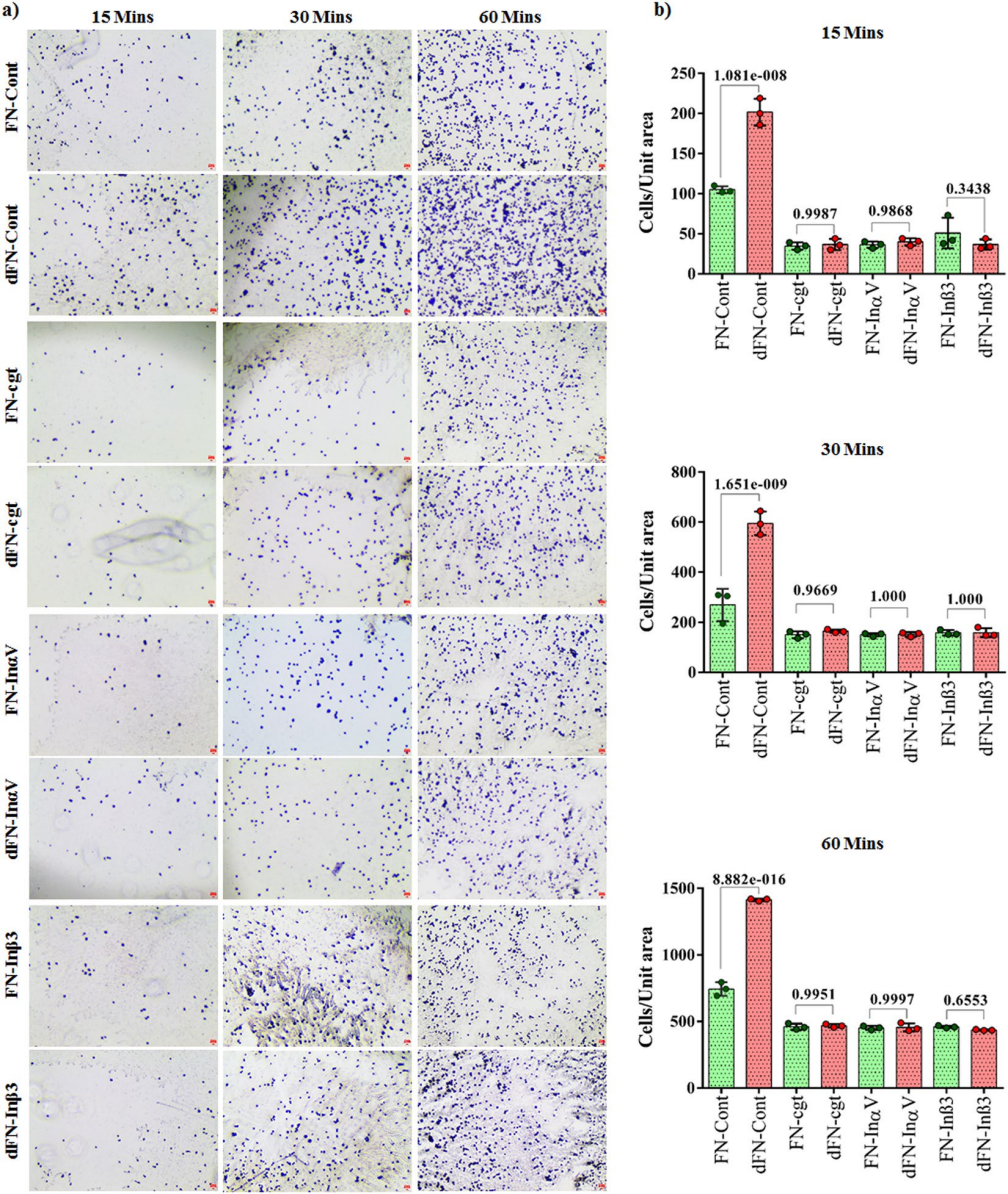
Microscopy images of differentiated U937 monocytic cell adhesion to culture plates coated with ECM proteins fibronectin (FN) or deamidated fibronectin (dFN) in different experimental conditions (**a**). Scale bar indicates 100 µm. Quantitation of cell adhesion to ECM proteins fibronectin and deamidated fibronectin under different experimental conditions. Statistics were calculated from experimental triplicates. Fibronectin (FN); Deamidated fibronectin (dFN), Differentiated U937 cells (Cont) were treated or not with Cilengitide (cgt) or monoclonal antibodies against integrin α_V_ (Inα_V_) or integrin β_3_ (Inβ_3_).

**Figure 4:**
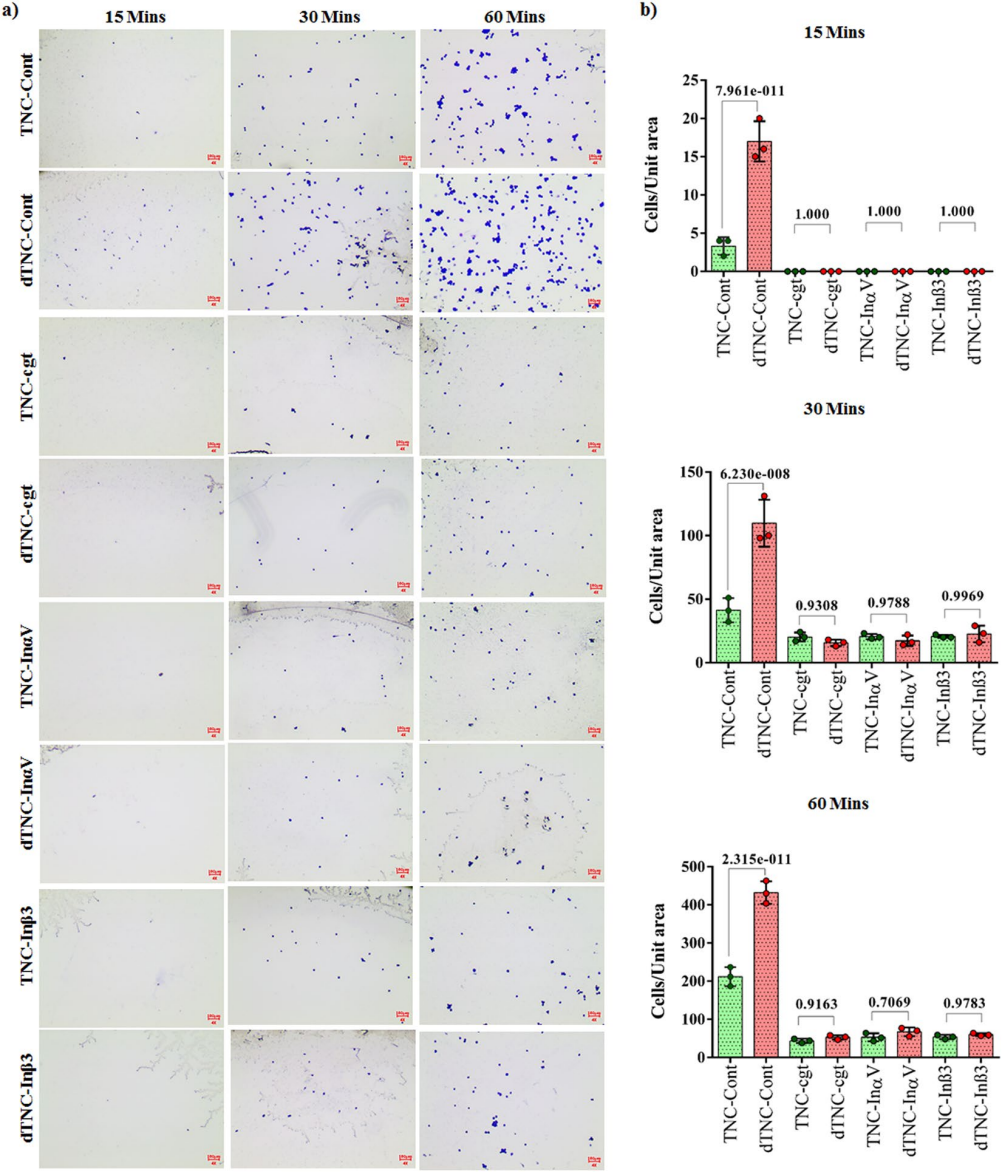
Microscopy images of differentiated U937 monocytic cell adhesion to culture plates coated with ECM proteins tenascin C (TNC) or deamidated tenascin C (dTNC) in different experimental conditions (**a**). Scale bar indicates 100 µm. Quantitation of cell adhesion to ECM proteins Tenascin C and deamidated Tenascin C under different experimental conditions. Statistics were calculated from experimental triplicates. Tenascin C (TNC); Deamidated Tenascin C (dTNC). Differentiated U937 cells (Cont) were treated or not with Cilengitide (cgt) or monoclonal antibodies against integrin α_v_ (Inα_v_) or integrin β_3_ (Inβ_3_).

**Figure 5:**
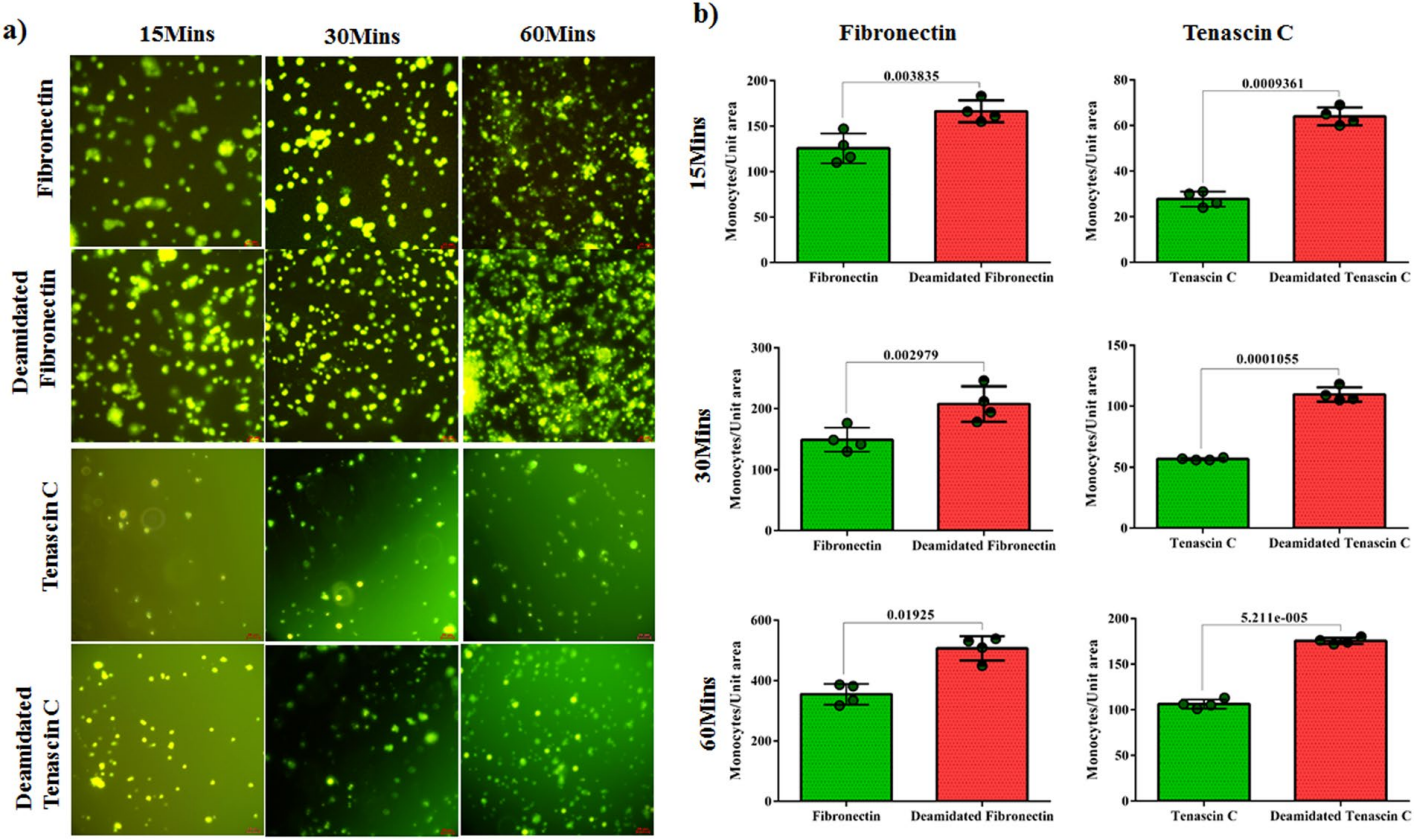
Microscopy images of primary human monocyte adhesion to culture plates coated with fibronectin or tenascin C native proteins (PBS-only treated controls) or the deamidated forms of these ECM components (50 mM TEAB treated). Isolated monocytes were labelled with CFSE dye and the images captured using green fluorescent filter. Scale bar indicates 20 µm. Statistics were calculated from 4 experimental replicates.

We next hypothesized that isoDGR modifications in ECM proteins promote integrin-mediated mononuclear cell adhesion and recruitment into atherosclerotic plaques, leading to arterial dysfunction and disease progression. To test this possibility, we assessed the ability of integrin α_v_β_3_-expressing pre-monocytic cells to adhere to ECM proteins FN and TNC that had been coated onto plastic overnight in the presence or absence of 50 mM tetraethyl ammonium bicarbonate (TEAB) buffer (pH 8.5) to induce asparagine deamidation. We first assessed the extent of protein deamidation induced by TEAB treatment by extracting plate-bound FN and TNC for LC-MS/MS analysis, which revealed extensive FN deamidation at residue 263 in the sequence GNLLQCICTGNGRGEWK and marked TNC deamidation at residue 2118 in the sequence VEGYSGTAGDSMAYHNGR. The percentage of deamidation at each targeted NGR motif was determined by integrating the area of the extracted ion chromatogram (XIC) with the calculated precursor masses of the native and deamidated peptides (+/− 5 ppm mass range). Using this approach, we determined that overall levels of protein deamidation in TEAB-treated plates were ~8% for FN and ~5.3% for TNC, whereas the PBS-treated control plates exhibited <0.5% deamidation of either protein (Fig. 2). We therefore proceeded to test the capacity of these different ECM substrates to bind U937 pre-monocytic cells that had been stimulated with 50 ng TPA (12-*O*-tetradecanoyl Phorbol-13-acetate)^57^ to induce differentiation and upregulation of integrin α_v_β_3_. Western blot analyses indicated that β_3_ integrin expression was increased after 3 h and reached a stable maximum within 12–24 h of TPA treatment, consistent with differentiation of U937 cells towards a macrophage phenotype (Fig. S2a,b). We therefore elected to stimulate U937 cells with 50 ng TPA for 24 h and then incubate for 48 h in fresh RPMI-1640 medium supplemented with 10% FBS prior to use in the cell adhesion assays^58^. Analysis by microscopy revealed that the number of adherent U937 cells counted within a fixed area during the 60 min assay period was increased ~2-fold in the presence of deamidated FN (Fig. 3b) and ~2.5-fold with deamidated TNC (Fig. 4b) compared with unmodified protein controls. Preferential monocyte-macrophage binding to deamidated FN was observed within just 15 min and continued to increase over the entire assay period (Fig. 3b), while increased cell adhesion to deamidated TNC was evident after 30–60 min incubation (Fig. 4a,b). Similar data were obtained when using these same assays to determine the binding capacity of primary human CD14+ blood monocytes, which also displayed preferential adhesion to deamidated FN (~1.3-fold) and deamidated TNC (~2-fold) (Fig. 5b).

### Integrin α_v_β_3_ mediates monocyte-macrophage binding to deamidated ECM Proteins

Our observation that monocyte-macrophages expressing α_v_β_3_ preferentially adhere to deamidated FN and TNC suggested that isoDGR modification of these proteins can enhance integrin binding. We therefore assessed whether the preferential binding of U937 cells to deamidated ECM proteins was attenuated in the presence of the α_v_-selective antagonist cilengitide^59^ and results were further confirmed by blocking of integrins α_v_ and β_3_ using monoclonal antibodies. Primed U937 cells were incubated with or without 5 µg cilengitide overnight and then assessed for ability to bind to unmodified or deamidated ECM proteins via counting of adherent cells (Figs 3a,b and 4a,b). Consistent with our previous findings, U937 cell binding to deamidated FN was increased >2-fold relative to the unmodified control protein, whereas cilengitide treatment impaired cell binding to both the unmodified and deamidated proteins (Fig. 3a,b). Cilengitide-mediated blocking of integrin α_v_ conferred a ~3-fold reduction in U937 cell binding to both native and deamidated FN compare to their native control, although the inhibitory effect declined with increasing assay duration (Fig. 3a,b). Cilengitide also induced a ~4-fold reduction in cell adhesion to native and deamidated TNC protein respect to the native control over the 60 min assay period (Fig. 4a,b). Inhibition of α_v_ and β_3_ using monoclonal blocking antibodies replicated the effect of cilengitide (Figs 3a,b and 4a,b). A maximum of 2.88-fold and 2.86-fold reduction in cell adhesion to both native and deamidated FN was achieved by respective α_v_ and β_3_ monoclonal blocking antibodies while the vales were 3.6-fold and 3.7-fold for TNC protein (Figs 3a,b and 4a,b). Together, these data reveal that deamidation of NGR motifs in ECM proteins FN and TNC enhances monocyte-macrophage adhesion mediated by α_v_β_3_ integrin and likely promotes leukocyte recruitment to human atherosclerotic plaques.

## Discussion

The current report provides evidence of a critical role for isoDGR motifs in mediating leukocyte adhesion to the ECM via interactions with integrin α_v_β_3_ and indicates that protein deamidation may promote atherosclerosis by enhancing monocyte-macrophage recruitment to developing plaques. Integrins are heterodimeric transmembrane molecules that bind to a conserved RGD sequence present in various host peptides^60^, but it has long been recognised that alternative motifs may also be capable of mediating integrin binding. In 1995, the peptide NGRAHA was identified by Koivunen and colleagues as a low-affinity homolog of the RGD ligand for α_v_β_3_ integrin^61^, but it was not until 2006 that Curnis *et al*. reported that the NGR domain of fibronectin can undergo asparagine deamidation to form isoaspartic acid and increase integrin binding^6^. Our data now indicate that deamidation of ECM components can also induce gain-of- function structural changes that increase monocyte-macrophage binding to atherosclerotic plaque proteins.

During early-phase vascular injury, circulating monocytes migrate to the dysfunctional endothelial lining where they differentiate into macrophages (MФ) and produce pro-inflammatory cytokines and chemokines that recruit additional monocytes^33^. Having populated the endothelial lining, MФ then engulf oxidised LDL deposits in the lamina and develop into foam cells which are strongly associated with disease progression^33, 62^. Our data now suggest that deamidation-prone ECM proteins containing NGR motifs are likely to undergo gain-of-function modifications under stress conditions such as local inflammation during vascular injury^63^. We report for the first time that asparagine deamidation of the extracellular matrix proteins FN and TNC in particular can enhance monocyte adhesion via isoDGR interactions with integrin α_v_β_3_. Our initial proteomic analysis of human carotid atherosclerotic plaque tissues demonstrated that both FN and TNC proteins exhibit extensive NGR deamidation *in vivo*, and we further showed that these modifications confer a significant increase in monocyte-macrophage adhesion to ECM *in vitro*. Indeed, our observation that monocyte binding to the ECM was increased up to ~3.5-fold following induction of isoDGR motifs suggests that this mechanism could exert a major influence on the rate of plaque formation in human patients.

FN is a ubiquitous and highly abundant protein in human plasma and ECM. Initially secreted as a soluble disulphide-bonded dimer, FN is subsequently assembled into an insoluble multimeric protein in the ECM and provides structural support via interactions with collagens and fibrin. FN has also been shown to mediate cell surface receptor interactions with the ECM via activation of intracellular cytoskeletal rearrangements and signalling pathways. Consequently, FN is particularly abundant in tissues that are undergoing active regeneration in response to injury and has been strongly implicated in the pathology of atherosclerosis leading to cardiovascular disease^42–44^. Increased expression of FN in the injured vascular wall regulates smooth muscle cell transformation from a contractile phenotype to a synthesizing/proliferative phenotype that may contribute to intimal thickening. In atherosclerotic lesions there is a marked increase in FN co-localization with collagen type III, which is an archetypal feature of the tissue repair process during wound healing, whereas inhibition of FN expression using angiotensin II receptor antagonists is associated with a decrease in intimal thickening. These data suggest that drug targeting of FN structure-function changes may yield therapeutic benefit in human CVD patients.

TNC is highly expressed during embryogenesis but is almost absent in postnatal life and lacking in healthy arterial tissues^53^. However, TNC can be detected in tissues undergoing active remodelling and is up-regulated in atherosclerotic plaques where the protein reportedly contributes to smooth muscle cell migration and proliferation^54^. Sharifi and co-workers used immunohistochemical staining and *in situ* hybridization to demonstrate that TNC co-localizes with macrophages in human coronary atherosclerotic plaques that feature an organized lipid core or areas of rupture^40^, thus indicating that TNC expression and distribution correlate with inflammation. In a separate study, analysis of single nucleotide polymorphisms in human aortic tissues revealed that TNC genetic variants are strongly associated with atherosclerosis and coronary artery disease^55^. Taken together, these data demonstrate that FN and TNC are closely linked with the pathology of atherosclerosis and may represent promising targets for clinical interventions.

Intriguingly, we observed that the extent of TEAB-induced deamidation in fibronectin and tenascin C was <10% higher than the levels of spontaneous NGR deamidation observed in the untreated control. These data are consistent with previous reports that spontaneous NGR deamidation of ECM proteins is highly dependent on flanking residues, secondary/tertiary structure, and overall three-dimensional conformation^64^, which can be influenced by microenvironmental conditions including pH, temperature, and ionic strength^65^. Our data now reveal that these factors are also likely to exert a major influence on human atherosclerotic plaque progression to CVD via effects on monocyte-macrophage recruitment to the affected tissues. Pre-treatment of monocytic cells with the cyclic RGD-based pentapeptide cilengitide blocks α_v_β_3_ integrin binding sites and reduced adhesion to NGR-deamidated fibronectin and tenascin C by 3–4-fold. The specific blocking of α_v_ and β_3_ integrin by monoclonal blocking antibodies reproduce the effect as achieved by RGD-motif specific blocking by cilengitide confirms the NGR-deamidation mediated integrin binding which mimic the RGD-specific integrin binding. Since protein deamidation is a spontaneous reaction the quantity of modified protein present in affected tissues progressively increases with time^9–11^. We used human carotid plaque tissues from elderly donors, hence the samples contained significant amounts of deamidated FN and TNC (acquired over the course of natural ageing). Deamidation of freshly prepared proteins by short-term chemical treatment *in vitro* was predictably lower than can be generated *in vivo*, but still conferred a significant increase in leukocyte adhesion to ECM. It is therefore possible that deamidation of FN and TNC in human tissues *in vivo* leads to even greater enhancement of leukocyte recruitment to carotid plaques than was observed in our assays. Collectively, these findings indicate that deamidation of the NGR domain confers gain-of-function changes in ECM proteins that increase leukocyte binding mediated by α_v_β_3_. Therapeutic targeting of this novel mechanism may represent an effective approach to inhibiting plaque development and delaying/preventing progression to CVD in human patients.

## Conclusion

In this study, we performed proteomic profiling of human carotid atherosclerotic plaque samples and identified that numerous extracellular matrix (ECM) proteins display isoDGR motifs with potential ‘gain-of-function’ properties. IsoDGR is generated by deamidation of the NGR domain in select ECM proteins and mimics the functions of the integrin α_v_β_3_-binding RGD motif. Accordingly, monocyte-macrophage adhesion to ECM proteins was significantly enhanced upon NGR deamidation of the atherosclerosis-associated matrix components fibronectin and tenascin C via a mechanism that required integrin α_v_β_3_. In conclusion, deamidation of asparagine residues in the NGR motif of atherosclerotic plaque proteins likely contributes to disease progression via recruitment of circulating monocytes that adhere to the modified ECM via integrin α_v_β_3_.

## Materials and Methods

### Antibodies and reagents

All reagents and chemicals used in this study were purchased from Sigma (St. Louis, USA) unless otherwise specified. For cell culture, RPMI-1640 medium was purchased from PAA Laboratories GmbH (Austria). Tenascin C protein (Cat. No. CC065) was obtained from EMD Millipore (Billerica, Massachusetts, USA). Cilengitide was purchased from Selleckchem (Houston, USA). Rabbit anti-human integrin α_v_ and β_3_ were from Cell Signaling Technologies, Inc. (Danvers, MA, USA). HRP goat anti-rabbit IgG secondary antibody was from Zymed (San Francisco, California, USA). Sequencing-grade trypsin was from Promega (Madison, MI, USA). Human Pan Monocyte Isolation Kits were purchase from Miltenyi Biotec GmbH (Germany) and 5-Carboxyfluorescein N-Succinimidyl ester (CFSE) fluorescent dye was purchase from Cayman Chemical (Michigan, USA).

### Proteomic profiling of human carotid atherosclerotic plaques

LC-MS/MS-based proteomic profiling of human atherosclerotic plaque tissues was performed as previously described^45^. Briefly, atherosclerotic plaque samples were obtained from n = 38 patients who underwent carotid endarterectomy at University Medical Center Utrecht (Netherlands) between 2002 and 2006^66^. Patients were aged 56–83 and included 32 males and 6 females. The clinical study has been approved by the Institutional Review board of the hospital and written informed consent was obtained from all patients. The experimental protocols were approved by the relevant ethical boards and were conducted in accordance with NTU guidelines. Blood clots were removed from the plaque samples, followed by extensive washing with PBS to remove any soluble materials prior to use in our analyses. Each of the 38 atherosclerotic plaque samples was transferred into lysis buffer (8 M Urea, 50 mM ammonium acetate, pH 6) containing protease inhibitors and then homogenized using a Bullet Blender® (Next Advance, Inc., NY), before pooling and subjecting these to trypsin digestion. The tryptic peptides derived from the atherosclerotic plaque samples were prepared as previously described, with minor modifications^45^. Peptides generated from approximately 1 mg of proteins were fractionated using ERLIC on a HPLC system and then combined into 20 separate fractions^67^. Peptides from each fraction were separated and analyzed in triplicate on a Dionex Ultimate 3000 RSLCnano system coupled to a Q Exactive instrument (Thermo Scientific, San Jose, USA). The raw data are available for download from PeptideAtlas using the dataset identifier PASS00406 (http://www.peptideatlas.org/PASS/PASS00406). Raw data were converted to Mascot generic file (mgf) format using Proteome Discoverer v1.4 (Thermo Scientific, San Jose, USA) with deisotope in MS/MS. The mgf files were searched against the Uniprot human proteome database in Mascot using the following parameters; fixed modification: Carbamidomethyl, variable modification: Deamidation (NQ), Oxidation (M); MS/MS tolerance 5.1 ppm, peptide mass tolerance: 0.02 Da, # of missed cleavage: 2. The Mascot results were exported to an Excel file for further analysis.

### Cell culture

Pro-monocytic U937 cells were cultured in RPMI-1640 medium supplemented with 10% FBS and 1 mM sodium pyruvate. Cells were differentiated by stimulation with 50 ng/ml Phorbol 12-myristate 13-acetate (PMA) (Sigma, St. Louis, MO, USA) for 24 h followed by a further 48 h incubation in fresh culture medium.

### Cell adhesion assay

ECM proteins were coated onto 96-well plates (2 µg protein in 50 µl PBS, pH 6.8) via overnight incubation at 37 °C. Non-adhered ECM proteins were then removed and the wells washed twice with PBS. Deamidation of ECM proteins was induced by addition of 50 mM TEAB buffer (pH 8.5) and overnight incubation at 37 °C. All wells were then washed twice with PBS to remove residual TEAB prior to addition of differentiated U937 cells (1 × 10^4^ cells per well) and incubation at 37 °C for the indicated times. In inhibition experiments, U937 cells were pre-treated with 5 µg cilengitide overnight (1 × 10^4^ cells per condition) prior to use in the adhesion assays. Alternatively, blocking of α_v_ and β_3_ was achieved by incubating U937 cells with the corresponding anti-integrin monoclonal antibodies (1:500 dilution) in 10% FBS-supplemented DMEM at 37 °C for 1 h immediately prior to use in adhesion assays. After incubation, non-adherent cells were removed from the wells by washing twice with PBS. Adherent cells were fixed by addition of 95% ethanol for 15 min at RT. The fixed cells were then stained with crystal violet for 20 min followed by extensive washing with PBS to remove excess dye. Images of the adherent cells were captured using a Nikon EU 2000 inverted microscope. Quantification was performed by cell counting in experimental triplets and the statistical significance was calculated by using nonparametric one-way ANOVA with multiple comparisons test.

### Primary Monocyte Adhesion Assay

Blood samples were obtained after written informed consent from two healthy volunteers recruited via NTU health clinic (Singapore). Experimental protocols were approved by the ethical boards and were conducted in accordance with NTU guidelines. Human peripheral blood mononuclear cells (PBMCs) were isolated from each fresh 15 ml blood sample by density gradient centrifugation over Histopaque-1077 at 400 × g for 20 min. Finally, the human primary monocytes were isolated from PBMCs using monocyte isolation kits according to the manufacturer’s protocol. Isolated monocytes were suspended in PBS and labelled with 10 µM CFSE for 10 min at 37 °C prior to use in the adhesion assays (~3 × 10^4^ cells per well). Images of adherent cells were captured in the green fluorescence channel of a Nikon ECLIPSE Ti-S inverted microscope coupled with a Nikon DS-Ri2 camera. Quantification was performed by cell counting in 4 experimental replicates and nonparametric one-way ANOVA was used for a comparison between groups.

### Western blotting

U937 cells were stimulated with PMA for the indicated times and cell lysate was collected for assessment of β_3_ integrin expression levels. Briefly, cells were lysed in RIPA buffer containing a cocktail of EDTA-free protease inhibitors (Roche, Mannheim, Germany) and phosphatase inhibitors (Roche, Mannheim, Germany). Lysates were clarified by centrifugation (16,000 × g, 30 min) and subjected to Western blotting using an anti-integrin β_3_ antibody (1:1000 dilution). Protein-antibody conjugates were visualized using a chemiluminescence detection kit according to the manufacturer’s protocol (Thermo Scientific).

### ECM protein extraction

ECM proteins were extracted from coated 96-well plates for LC-MS/MS analysis to determine the extent of deamidation. TEAB-treated (deamidated) and PBS-treated (non-deamidated) plates were incubated with 8 M urea and 50 mM ammonium acetate (pH 6.5) on a rotatory shaker for 2 h at 37 °C. Proteins extracted into the urea buffer were then collected in a microcentrifuge tube and precipitated in chilled acetone by incubation at −20 °C overnight. Precipitated proteins were finally subjected to LC-MS/MS analysis of deamidation.

### LC-MS/MS analysis of ECM protein deamidation

Extracted ECM protein samples were reduced, alkylated, and diluted with ammonium acetate to achieve 1 M urea prior to trypsin digestion overnight at 37 °C. The digested protein samples were desalted and dried in a vacuum concentrator. Tryptic peptides were then separated and analysed on a Dionex Ultimate 3000 RSLC-nano system coupled to a LTQ-FT Ultra (Thermo Electron, Bremen, Germany) using a C18 nano-HPLC column with a standard LC-MS/MS gradient. Peptide ions were analysed on LTQ-FT with an ADVANCE™ CaptiveSpray™ Source (Michrom BioResources, USA). Spectra were acquired with XCalibur (version 2.0 SR2). MS/MS spectra were extracted from raw data and converted into mgf files in Proteome Discoverer 1.4 before searching these against the human database in Mascot software (parameters used were; fixed modification: Carbamidomethyl, variable modification: Deamidation (NQ), Oxidation (M); MS/MS tolerance 5.1 ppm, peptide mass tolerance: 0.8 Da, No. of missed cleavage: 2). The Mascot-annotated MS/MS spectra of the targeted deamidated and non-deamidated peptides were confirmed by manual inspection of the search results. The percentage of deamidation at each targeted NGR motif was determined by the area of the extracted ion chromatogram integrated with +/− 5 ppm mass range of the calculated precursor masses of the native and deamidated peptides.

## Electronic supplementary material

Supplementary Data 1

Supplimentary Figures

Supplementary Data 2

## References

[1] Kampoli AM, Tousoulis D, Antoniades C, Siasos G, Stefanadis C (2009). Biomarkers of premature atherosclerosis. Trends Mol Med.

[2] Klingenberg, R., Hasun, M., Corti, R. & Lüscher, T. F. Clinical Manifestations of Atherosclerosis. *Inflammation and Atherosclerosis* 39–58 (2012).

[3] Packard RR, Libby P (2008). Inflammation in atherosclerosis: from vascular biology to biomarker discovery and risk prediction. Clin Chem.

[4] Roger VL (2011). Heart Disease and Stroke Statistics—2011 Update1. About 1. About These Statistics2. American Heart Association’s 2020 Impact Goals3. Cardiovascular Diseases4. Subclinical Atherosclerosis5. Coronary Heart Disease, Acute Coronary Syndrome, and Angina Pectoris6. Stroke (Cerebrovascular Disease) 7. High Blood Pressure8. Congenital Cardiovascular Defects9. Cardiomyopathy and Heart Failure10. Other Cardiovascular Diseases11. Family History and Genetics12. Risk Factor: Smoking/Tobacco Use13. Risk Factor: High Blood Cholesterol and Other Lipids14. Risk Factor: Physical Inactivity15. Risk Factor: Overweight and Obesity16. Risk Factor: Diabetes Mellitus17. End-Stage Renal Disease and Chronic Kidney Disease18. Metabolic Syndrome19. Nutrition20. Quality of Care21. Medical Procedures22. Economic Cost of Cardiovascular Disease23. At-a-Glance Summary Tables 24. Glossary. Circulation.

[5] Roger VL, Go AS, Lloyd-Jones DM, Adams RJ, Berry JD (2011). American Heart Association Statistics Committee and Stroke Statistics Subcommittee. Heart disease and stroke statistics–2011 update: a report from the American Heart Association. Circulation.

[6] Curnis F (2006). Spontaneous formation of L-isoaspartate and gain of function in fibronectin. J Biol Chem.

[7] Imanaka-Yoshida K (2012). Tenascin-C in cardiovascular tissue remodeling: from development to inflammation and repair. Circ J..

[8] Matsui Y, Morimoto J, Uede T (2010). Role of matricellular proteins in cardiac tissue remodeling after myocardial infarction. World J Biol Chem.

[9] Robinson NE, Robinson AB (2001). Molecular clocks. Proceedings of the National Academy of Sciences of the United States of America.

[10] Robinson NE, Robinson AB (2001). Deamidation of human proteins. Proceedings of the National Academy of Sciences of the United States of America.

[11] Truscott RJ, Friedrich MG (2016). The etiology of human age-related cataract. Proteins don’t last forever. Biochimica et biophysica acta.

[12] Ray NJ, Hall D, Carver JA (2016). Deamidation of N76 in human gammaS-crystallin promotes dimer formation. Biochimica et biophysica acta.

[13] Gallart-Palau X (2015). Extracellular vesicles are rapidly purified from human plasma by PRotein Organic Solvent PRecipitation (PROSPR). Scientific reports.

[14] Qin Z, Dimitrijevic A, Aswad DW (2015). Accelerated protein damage in brains of PIMT +/− mice; a possible model for the variability of cognitive decline in human aging. Neurobiology of aging.

[15] Barbariga M (2015). Ceruloplasmin functional changes in Parkinson’s disease-cerebrospinal fluid. Molecular neurodegeneration.

[16] Dan A (2013). Extensive deamidation at asparagine residue 279 accounts for weak immunoreactivity of tau with RD4 antibody in Alzheimer’s disease brain. Acta neuropathologica communications.

[17] Hasegawa M (1992). Protein sequence and mass spectrometric analyses of tau in the Alzheimer’s disease brain. J Biol Chem.

[18] Adav SS, Sze SK (2016). Insight of brain degenerative protein modifications in the pathology of neurodegeneration and dementia by proteomic profiling. Molecular brain.

[19] Patananan AN, Capri J, Whitelegge JP, Clarke SG (2014). Non-repair pathways for minimizing protein isoaspartyl damage in the yeast Saccharomyces cerevisiae. The Journal of biological chemistry.

[20] Sandmeier E, Hunziker P, Kunz B, Sack R, Christen P (1999). Spontaneous deamidation and isomerization of Asn108 in prion peptide 106–126 and in full-length prion protein. Biochemical and biophysical research communications.

[21] Corti A, Curnis F (2011). Isoaspartate-dependent molecular switches for integrin-ligand recognition. J Cell Sci.

[22] Barbariga M (2014). Oxidation-induced Structural Changes of Ceruloplasmin Foster NGR Motif Deamidation That Promotes Integrin Binding and Signaling. Journal of Biological Chemistry.

[23] Weintraub SJ, Deverman BE (2007). Chronoregulation by asparagine deamidation. Sci STKE.

[24] Chuang CY (2014). Oxidation modifies the structure and function of the extracellular matrix generated by human coronary artery endothelial cells. Biochem J.

[25] Spitaleri A (2008). Structural basis for the interaction of isoDGR with the RGD-binding site of alphavbeta3 integrin. J Biol Chem.

[26] Zou M, Zhang L, Xie Y, Xu W (2012). NGR-based strategies for targeting delivery of chemotherapeutics to tumor vasculature. Anticancer Agents Med Chem.

[27] Ghitti M (2012). Molecular dynamics reveal that isoDGR-containing cyclopeptides are true alphavbeta3 antagonists unable to promote integrin allostery and activation. Angew Chem Int Ed Engl.

[28] Frank AO (2010). Conformational control of integrin-subtype selectivity in isoDGR peptide motifs: a biological switch. Angew Chem Int Ed Engl.

[29] Takahashi S (2007). The RGD motif in fibronectin is essential for development but dispensable for fibril assembly. J Cell Biol.

[30] Rizzardi, G. P. & Bordignon, C. NGR and isoDGR are separate moieties binding to different receptors. *Blood* **113**, 5366; author reply 5367, doi:10.1182/blood-2009-02-207068 (2009). 10.1182/blood-2009-02-207068 19470442

[31] Corti A, Curnis F, Arap W, Pasqualini R (2008). The neovasculature homing motif NGR: more than meets the eye. Blood.

[32] Curnis F (2008). Isoaspartate-glycine-arginine: a new tumor vasculature-targeting motif. Cancer Res.

[33] Libby P (1996). Macrophages and atherosclerotic plaque stability. Current opinion in lipidology.

[34] Bartolome RA (2014). An RGD motif present in cadherin 17 induces integrin activation and tumor growth. J Biol Chem.

[35] Bleijerveld OB (2013). Proteomics of plaques and novel sources of potential biomarkers for atherosclerosis. Proteomics. Clinical applications.

[36] Ionita MG (2010). High myeloid-related protein: 8/14 levels are related to an increased risk of cardiovascular events after carotid endarterectomy. Stroke; a journal of cerebral circulation.

[37] Peeters W (2011). Adipocyte fatty acid binding protein in atherosclerotic plaques is associated with local vulnerability and is predictive for the occurrence of adverse cardiovascular events. European heart journal.

[38] Hao P (2014). Deep proteomic profiling of human carotid atherosclerotic plaques using multidimensional LC-MS/MS. Proteomics. Clinical applications.

[39] Schaff M (2011). Novel function of tenascin-C, a matrix protein relevant to atherosclerosis, in platelet recruitment and activation under flow. Arterioscler Thromb Vasc Biol.

[40] Wallner K (1999). Tenascin-C is expressed in macrophage-rich human coronary atherosclerotic plaque. Circulation.

[41] Imanaka-Yoshida K (2012). Tenascin-C in cardiovascular tissue remodeling: from development to inflammation and repair. Circ J.

[42] Santovito D, Weber C (2015). Fibronectin extradomain A: balancing atherosclerotic plaque burden and stability. Thrombosis and haemostasis.

[43] Prakash P, Kulkarni PP, Lentz SR, Chauhan AK (2015). Cellular fibronectin containing extra domain A promotes arterial thrombosis in mice through platelet Toll-like receptor 4. Blood.

[44] Murphy PA, Hynes RO (2014). Alternative splicing of endothelial fibronectin is induced by disturbed hemodynamics and protects against hemorrhage of the vessel wall. Arterioscler Thromb Vasc Biol.

[45] Hao, P., Ren, Y., Alpert, A. J. & Sze, S. K. Detection, evaluation and minimization of nonenzymatic deamidation in proteomic sample preparation. *Mol Cell Proteomics* **10**, O111 009381, doi:10.1074/mcp.O111.009381 (2011). 10.1074/mcp.O111.009381 PMC3205879 21784994

[46] Litteri G (2012). Vascular smooth muscle Emilin-1 is a regulator of arteriolar myogenic response and blood pressure. Arterioscler Thromb Vasc Biol.

[47] Danussi C (2008). Emilin1 deficiency causes structural and functional defects of lymphatic vasculature. Molecular and cellular biology.

[48] Shen C (2009). Emilin1 gene and essential hypertension: a two-stage association study in northern Han Chinese population. BMC medical genetics.

[49] Zacchigna L (2006). Emilin1 links TGF-beta maturation to blood pressure homeostasis. Cell.

[50] Liu X (2004). Elastic fiber homeostasis requires lysyl oxidase-like 1 protein. Nature genetics.

[51] Nave AH (2014). Lysyl oxidases play a causal role in vascular remodeling in clinical and experimental pulmonary arterial hypertension. Arterioscler Thromb Vasc Biol.

[52] Nepomuceno AI, Gibson RJ, Randall SM, Muddiman DC (2014). Accurate identification of deamidated peptides in global proteomics using a quadrupole orbitrap mass spectrometer. Journal of proteome research.

[53] Chiquet-Ehrismann, R. & Tucker, R. P. Tenascins and the importance of adhesion modulation. *Cold Spring Harb Perspect Biol* **3**, doi:10.1101/cshperspect.a004960 (2011). 10.1101/cshperspect.a004960 PMC3101840 21441591

[54] Golledge J, Clancy P, Maguire J, Lincz L, Koblar S (2011). The role of tenascin C in cardiovascular disease. Cardiovasc Res.

[55] Minear MA (2011). Polymorphic variants in tenascin-C (TNC) are associated with atherosclerosis and coronary artery disease. Hum Genet.

[56] Arike L, Peil L (2014). Spectral counting label-free proteomics. Methods in molecular biology (Clifton, N.J.).

[57] Pucillo CE (1993). Interactions of promonocytic U937 cells with proteins of the extracellular matrix. Immunology.

[58] Hass R (1989). TPA-induced differentiation and adhesion of U937 cells: changes in ultrastructure, cytoskeletal organization and expression of cell surface antigens. Eur J Cell Biol.

[59] Mas-Moruno C, Rechenmacher F, Kessler H (2010). Cilengitide: the first anti-angiogenic small molecule drug candidate design, synthesis and clinical evaluation. Anticancer Agents Med Chem.

[60] Katoh K, Mohri H, Ogawa K, Okubo T (1998). Human Plasma Fibronectin Mediates Adhesion of U937 Cells by RGD and CS1. J Thromb Thrombolysis.

[61] Koivunen E, Gay DA, Ruoslahti E (1993). Selection of peptides binding to the alpha 5 beta 1 integrin from phage display library. J Biol Chem.

[62] Dutta P (2012). Myocardial infarction accelerates atherosclerosis. Nature.

[63] Van Agthoven JF (2014). Structural basis for pure antagonism of integrin αVβ3 by a high-affinity form of fibronectin. Nat Struc & Mol Biol..

[64] Serra, A., Gallart-Palau, X., Wei, J. & Sze, S. K. Characterization of Glutamine Deamidation by Long-Length Electrostatic Repulsion-Hydrophilic Interaction Chromatography-Tandem Mass Spectrometry (LERLIC-MS/MS) in Shotgun Proteomics. *Analytical chemistry*, doi:10.1021/acs.analchem.6b02688 (2016). 10.1021/acs.analchem.6b02688 27689507

[65] Robinson NE (2004). Structure-dependent nonenzymatic deamidation of glutaminyl and asparaginyl pentapeptides. The journal of peptide research: official journal of the American Peptide Society.

[66] de Kleijn DP (2010). Local atherosclerotic plaques are a source of prognostic biomarkers for adverse cardiovascular events. Arterioscler Thromb Vasc Biol.

[67] Hao P (2010). Novel application of electrostatic repulsion-hydrophilic interaction chromatography (ERLIC) in shotgun proteomics: comprehensive profiling of rat kidney proteome. Journal of proteome research.

