# Supplementary material for: Monocyte adhesion to atherosclerotic matrix proteins is enhanced by Asn-Gly-Arg deamidation
Source: Sci Rep. 2017 Jul 18;7:5765. doi: 10.1038/s41598-017-06202-2 (PMC5515959; doi:10.1038/s41598-017-06202-2)
Supplement: Supplementary file 1 — Supplementary Data 1 [file 41598_2017_6202_MOESM1_ESM.pdf]

Monocyte adhesion to atherosclerotic matrix proteins is enhanced by Asn-Gly-Arg deamidation

Bamaprasad Dutta[1a], Jung Eun Park[1a], Subodh Kumar[1], Piliang Hao[1], Xavier Gallart-Palau[1], Aida Serra[1], Yan Ren[1], Vitaly Sorokin[2,3], Chuen Neng Lee[2,3], Hee Hwa Ho[4], Dominique de Kleijn[3], and Siu Kwan Sze[1*]

[1]School of Biological Sciences, Nanyang Technological University, 60 Nanyang Drive, Singapore 637551.
[2]Department of Surgery, Yong Loo Lin School of Medicine, National University of Singapore, Singapore 119228.
[3]Department of Cardiac, Thoracic and Vascular Surgery, National University Heart Centre, National University Health System, Singapore 119228.
[4]Department of Cardiology, Tan Tock Seng Hospital, 11 Jalan Tan Tock Seng, Singapore 308433.

[a] These authors contributed equally to this work.

Running Title: IsoDGR enhances monocyte adhesion to ECM proteins

A2M_K.EQAPHCICAN#GR.Q_78.32
A2M_K.EQAPHCICANGR.Q_25.55
A2M_R.VSVQLEASPAFLAVPVEKEQAPHCICANGR.Q_81.54
APOD_K.IPTTFEN#GR.C_36.94
APOD_R.WYEIEKIPTTFEN#GR.C_99.47
APOD_R.WYEIEKIPTTFEN#GR.C_87.09
APOD_R.WYEIEKIPTTFENGR.C_65.46
APOD_R.WYEIEKIPTTFENGR.C_54.48
CRKL_R.MNINGQWEGEVN#GR.K_15.45
DUSP3_R.AADFIDQALAQKN#GR.V_90.75
DUSP3_R.AADFIDQALAQKNGR.V_82.38
EMILIN1_R.AVETAFN#GR.Q_56.24
F9_K.NCELDVTCNIKN#GR.C_117.25
F9_K.NCELDVTCNIKNGRCEQFCK.N_17.62
FBN1_R.DIDECLQN#GR.I_73.28
FBN1_R.DIDECLQNGR.I_41.99
FBN1_R.DIDECLQN#GRICN#N#GR.C_58.50
FBN1_R.DIDECLQN#GRICN#NGR.C_79.97
FBN1_R.DIDECLQ#N#GRICNNGR.C_48.47
FBN1_R.DIDECLQN#GRICNNGR.C_60.40
FBN1_R.DIDECLQN#GRICNNGR.C_58.46
FBN1_R.DIDECLQNGRICNN#GR.C_25.26
FBN1_K.DIN#ECETPGICM#N#GR.C_27.60
FBN1_K.DINECETPGICM#N#GR.C_73.25
FBN1_K.DINECETPGICM#NGR.C_74.80
FBN1_K.DINECETPGICM#NGR.C_34.06
FBN1_K.DINECETPGICMN#GR.C_101.53
FBN1_K.DINECETPGICMN#GR.C_20.31
FBN1_K.DINECETPGICMNGR.C_76.65
FBN1_K.DINECETPGICMNGR.C_57.72
FBN1_R.RPDGEGCVDENECQTKPGICEN#GR.C_92.41
FBN1_R.RPDGEGCVDENECQ#TKPGICENGR.C_24.31
FBN1_R.RPDGEGCVDENECQTKPGICENGR.C_94.09
FBN1_R.RPDGEGCVDENECQTKPGICENGR.C_81.29
FBN1_R.YCKDIN#ECETPGICM#N#GR.C_16.83
FBN1_R.YCKDINECETPGICM#N#GR.C_53.12
FBN1_R.YCKDINECETPGICM#NGR.C_57.16
FBN1_R.YCKDINECETPGICMN#GR.C_64.61
FBN1_R.YCKDINECETPGICMNGR.C_68.75
FBN1_R.YCKDINECETPGICMNGR.C_38.90
FCGBP_R.FAVLQENVAWGN#GR.V_52.75
FGB_K.EDGGGWWYNRCHAAN#PN#GR.Y_24.14
FGB_K.EDGGGWWYNRCHAANPNGR.Y_29.11
FGG_K.EGFGHLSPTGTTEFWLGNEKIHLISTQ#SAIPYALRVELEDWNGR.T_50.31
FGG_K.EGFGHLSPTGTTEFWLGNEKIHLISTQ#SAIPYALRVELEDWNGR.T_18.91
FGG_K.EGFGHLSPTGTTEFWLGNEKIHLISTQSAIPYALRVELEDWNGR.T_52.88
FGG_K.EGFGHLSPTGTTEFWLGNEKIHLISTQSAIPYALRVELEDWNGR.T_51.95
FGG_K.IHLISTQSAIPYALRVELEDWN#GR.T_30.26
FGG_K.IHLISTQSAIPYALRVELEDWNGR.T_96.66
FGG_K.IHLISTQSAIPYALRVELEDWNGR.T_85.82
FGG_K.IHLISTQSAIPYALRVELEDWNGR.T_22.99
FGG_R.VELEDWN#GR.T_74.70
FGG_R.VELEDWNGR.T_67.56

FGG_R.VELEDWN#GRTSTADYAM#FK.V_84.50
FGG_R.VELEDWN#GRTSTADYAMFK.V_121.73
FGG_R.VELEDWN#GRTSTADYAMFK.V_27.53
FGG_R.VELEDWNGRTSTADYAMFK.V_119.50
FGG_R.VELEDWNGRTSTADYAMFK.V_37.83
FN1_R.CTCVGN#GRGEWTCIAYSQLR.D_83.07
FN1_R.CTCVGNGRGEWTCIAYSQLR.D_111.97
FN1_R.CTCVGNGRGEWTCIAYSQLR.D_34.30
FN1_K.DNRGN#LLQCICTGNGR.G_55.68
FN1_K.DNRGN#LLQCICTGN#GRGEWK.C_42.00
FN1_R.EDRVPHSRN#SITLTNLTPGTEYVVSIVALN#GR.E_50.94
FN1_R.EDRVPHSRNSITLTN#LTPGTEYVVSIVALN#GR.E_48.67
FN1_R.GN#LLQCICTGN#GR.G_18.29
FN1_R.GNLLQCICTGN#GR.G_78.25
FN1_R.GNLLQCICTGNGR.G_83.87
FN1_R.GNLLQCICTGNGR.G_44.49
FN1_R.GN#LLQCICTGN#GRGEWK.C_66.81
FN1_R.GNLLQ#CICTGN#GRGEWK.C_24.28
FN1_R.GNLLQ#CICTGN#GRGEWK.C_17.26
FN1_R.GNLLQCICTGN#GRGEWK.C_107.35
FN1_R.GNLLQCICTGN#GRGEWK.C_73.41
FN1_R.GNLLQCICTGNGRGEWK.C_112.25
FN1_R.GNLLQCICTGNGRGEWK.C_93.00
FN1_R.GNLLQCICTGN#GRGEWKCER.H_54.81
FN1_R.GNLLQCICTGNGRGEWKCER.H_52.57
FN1_R.NSITLTN#LTPGTEYVVSIVALN#GR.E_41.32
FN1_R.NSITLTNLTPGTEYVVSIVALN#GR.E_98.67
FN1_R.NSITLTNLTPGTEYVVSIVALN#GR.E_71.68
FN1_R.N#SITLTNLTPGTEYVVSIVALNGR.E_41.62
FN1_R.NSITLTN#LTPGTEYVVSIVALNGR.E_39.07
FN1_R.NSITLTNLTPGTEYVVSIVALNGR.E_112.83
FN1_R.NSITLTNLTPGTEYVVSIVALNGR.E_103.29
FN1_R.NSITLTN#LTPGTEYVVSIVALN#GREESPLLIGQQSTVSDVPR.D_47.29
FN1_R.NSITLTNLTPGTEYVVSIVALN#GREESPLLIGQ#QSTVSDVPR.D_46.43
FN1_R.NSITLTNLTPGTEYVVSIVALN#GREESPLLIGQQSTVSDVPR.D_106.04
FN1_R.NSITLTNLTPGTEYVVSIVALNGREESPLLIGQQ#STVSDVPR.D_21.59
FN1_R.NSITLTNLTPGTEYVVSIVALNGREESPLLIGQQSTVSDVPR.D_80.84
FN1_R.NSITLTNLTPGTEYVVSIVALNGREESPLLIGQQSTVSDVPR.D_30.47
FN1_K.QM#LCTCLGNGVSCQETAVTQTYGGNSNGEPCVLPFTYNGR.T_32.03
FN1_K.QM#LCTCLGNGVSCQETAVTQTYGGNSNGEPCVLPFTYNGR.T_31.92
FN1_K.QMLCTCLGNGVSCQETAVTQTYGGNSN#GEPCVLPFTYNGR.T_42.35
FN1_K.QMLCTCLGNGVSCQETAVTQTYGGNSNGEPCVLPFTYN#GR.T_38.88
FN1_K.QMLCTCLGNGVSCQETAVTQTYGGNSNGEPCVLPFTYN#GR.T_37.18
FN1_K.QMLCTCLGNGVSCQETAVTQTYGGNSN#GEPCVLPFTYNGR.T_26.29
FN1_K.QMLCTCLGNGVSCQETAVTQTYGGNSNGEPCVLPFTYNGR.T_75.08
FN1_K.QMLCTCLGNGVSCQETAVTQTYGGNSNGEPCVLPFTYNGR.T_68.34
FN1_K.TQ#GN#KQM#LCTCLGNGVSCQETAVTQTYGGNSNGEPCVLPFTYNGR.T_33.50
FN1_K.TQ#GNKQM#LCTCLGNGVSCQETAVTQTYGGNSNGEPCVLPFTYNGR.T_35.98
FN1_K.TQGNKQM#LCTCLGNGVSCQETAVTQTYGGNSN#GEPCVLPFTYNGR.T_33.85
FN1_K.TQGNKQM#LCTCLGNGVSCQETAVTQTYGGNSNGEPCVLPFTYNGR.T_47.18
FN1_K.TQGNKQMLCTCLGNGVSCQETAVTQTYGGNSN#GEPCVLPFTYNGR.T_42.98
FN1_K.TQGNKQMLCTCLGNGVSCQETAVTQTYGGNSNGEPCVLPFTYNGR.T_50.54
FNDC1_R.QGYN#GRPNVEGK.V_28.39
FRMD4B_-.RN#AN#GRSPN#N#SDK.T_26.72

HAPLN1_K.SRYDVFCFTSN#FN#GR.F_60.07
HAPLN1_K.SRYDVFCFTSN#FNGR.F_51.48
HAPLN1_K.SRYDVFCFTSN#FNGR.F_36.78
HAPLN1_K.SRYDVFCFTSNFNGR.F_38.87
HAPLN1_R.YDVFCFTSN#FN#GR.F_81.97
HAPLN1_R.YDVFCFTSN#FNGR.F_74.53
HAPLN1_R.YDVFCFTSN#FN#GRFYYLIHPTK.L_42.62
HSPA1B_K.LLQDFFN#GR.D_36.24
HSPA1B_K.LLQDFFNGR.D_61.47
HSPA1B_K.LLQ#DFFN#GRDLN#K.S_16.94
HSPA1B_K.LLQDFFN#GRDLN#K.S_20.16
HSPA1B_K.LLQDFFN#GRDLNK.S_76.44
HSPA1B_K.LLQDFFNGRDLNK.S_77.92
HSPA1B_K.LLQDFFNGRDLNK.S_20.67
HSPA5_K.N#GRVEIIANDQGNR.I_29.83
HSPA5_K.NGRVEIIANDQGNR.I_46.32
HSPA5_K.NGRVEIIANDQGNR.I_29.51
LAMC1_R.ATAESASECLPCDCN#GR.S_67.14
LAMC1_R.ATAESASECLPCDCNGR.S_105.26
LAMC1_R.ATAESASECLPCDCNGR.S_57.93
LAMC1_R.RATAESASECLPCDCN#GR.S_16.83
LAMC1_R.RATAESASECLPCDCNGR.S_57.69
LOXL1_R.LSVGSVYRPNQN#GR.V_34.53
LOXL1_R.LSVGSVYRPNQ#N#GRGLPDLVPDPN#YVQASTYVQR.A_22.57
LOXL1_R.LSVGSVYRPN#QN#GRGLPDLVPDPNYVQASTYVQR.A_51.77
LOXL1_R.LSVGSVYRPNQN#GRGLPDLVPDPN#YVQASTYVQR.A_31.93
LOXL1_R.LSVGSVYRPNQ#N#GRGLPDLVPDPNYVQASTYVQR.A_27.71
LOXL1_R.LSVGSVYRPNQN#GRGLPDLVPDPNYVQASTYVQR.A_37.22
MYOZ1_K.SNGRGGSQ#AGGSGSAGQYGSDQQHHLGSGSGAGGTGGPAGQAGR.G_39.26
MYOZ1_K.SN#GRGGSQAGGSGSAGQYGSDQQHHLGSGSGAGGTGGPAGQAGR.G_23.26
PLEC_K.TLPN#GRDALDGPAAEAEPEHSFDGLR.R_55.59
PLEC_K.TLPNGRDALDGPAAEAEPEHSFDGLR.R_30.07
PLEC_K.TLPN#GRDALDGPAAEAEPEHSFDGLRR.K_62.42
PLEC_K.TLPN#GRDALDGPAAEAEPEHSFDGLRR.K_50.89
PLEK_R.GCVVTSVESNSN#GR.K_30.61
PLEK_R.GCVVTSVESNSNGR.K_66.19
PLEK_R.GCVVTSVESN#SN#GRK.S_31.74
PLEK_R.GCVVTSVESNSN#GRK.S_46.36
PLG_K.EAQLPVIEN#KVCN#RYEFLN#GR.V_21.77
PLG_K.EAQLPVIEN#KVCNRYEFLN#GR.V_39.92
PLG_K.VCN#RYEFLN#GR.V_69.38
PLG_K.VCNRYEFLN#GR.V_48.55
PLG_K.VCNRYEFLNGR.V_43.54
PLG_R.YEFLN#GR.V_50.68
PLG_R.YEFLN#GRVQ#STELCAGHLAGGTDSCQGDSGGPLVCFEK.D_17.87
PLG_R.YEFLNGRVQ#STELCAGHLAGGTDSCQGDSGGPLVCFEK.D_17.87
PPL_R.SLLDLEN#GR.R_53.32
PTMA_K.EVVEEAEN#GR.D_72.49
PTMA_K.EVVEEAENGR.D_68.80
PTMA_K.KEVVEEAENGR.D_34.50
SH3KBP1_R.KEDGGWWEGQIN#GR.R_40.89
SH3KBP1_R.KEDGGWWEGQINGR.R_17.46
SHANK2_-.FM#N#VPGGGAAAVMMTGYNN#GR.C_22.07
SHANK2_-.GMN#VPGGGAAAVMMTGYNN#GR.C_17.52

SYNE1_R.DLQ#DRLSQMN#GRWDR.V_15.81
TBC1D1_R.KQ#N#LDLLEQLQ#VAN#GR.I_22.03
TNC_K.LKVEGYSGTAGDSM#AYHNGR.S_129.83
TNC_K.LKVEGYSGTAGDSM#AYHNGR.S_65.30
TNC_K.LKVEGYSGTAGDSMAYHN#GR.S_38.54
TNC_K.LKVEGYSGTAGDSMAYHNGR.S_144.67
TNC_K.LKVEGYSGTAGDSMAYHNGR.S_131.81
TNC_K.N#GRENFYQNWK.A_25.24
TNC_K.NGRENFYQNWK.A_23.12
TNC_K.NGRENFYQNWK.A_18.85
TNC_K.VEGYSGTAGDSM#AYHNGR.S_119.73
TNC_K.VEGYSGTAGDSMAYHN#GR.S_107.61
TNC_K.VEGYSGTAGDSMAYHNGR.S_182.06
TNC_K.VEGYSGTAGDSMAYHNGR.S_148.35
TNC_R.YKLKVEGYSGTAGDSM#AYHNGR.S_28.22
TNC_R.YKLKVEGYSGTAGDSMAYHNGR.S_79.01
WISP2_R.GALCLLAEDDSSCEVN#GR.L_98.82
WISP2_R.GALCLLAEDDSSCEVN#GR.L_52.02
WISP2_R.GALCLLAEDDSSCEVNGR.L_120.67
WISP2_R.GALCLLAEDDSSCEVNGR.L_83.85

Mascot Search Results

Peptide View

MS/MS Fragmentation of **EQAPHCICANGR**
Found in **P01023** in **uni_human_nr**, A2MG_HUMAN Alpha-2-macroglobulin OS=Homo sapiens GN=A2M PE=1 SV=3

Match to Query 2600: 1412.592732 from(471.871520,3+) intensity(292034.8750) rtinseconds(849) scans(2979) index(1254)
Title: 130809_HPL_Human_Plaque_BR1_TR3_08_Spectrum022864_scans__2979_RTINSECONDS=849
Data file E:\\RAW\\HumanPlaques\\mgf\\T\\1D\\T130809_HPL_Human_Plaque_BR1_TR3_08.mgf

Click mouse within plot area to zoom in by factor of two about that point
Or, Plot from 0 to 1100 Da Full range
Label all possible matches ○ Label matches used for scoring ⊙
Show Y-axis ☐

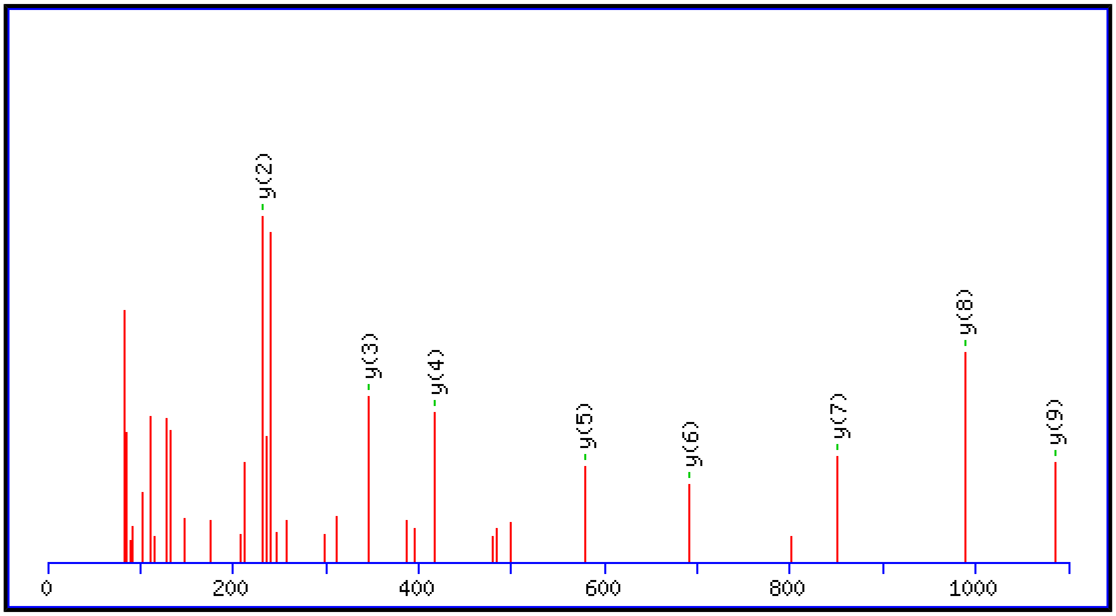

Monoisotopic mass of neutral peptide Mr(calc): 1412.5925
Fixed modifications: Carbamidomethyl (C) (apply to specified residues or termini only)
Variable modifications:
N10 : Deamidated (NQ)
Ions Score: 78 **Expect:** 2.6e-007
Matches : 8/108 fragment ions using 9 most intense peaks (help)

#	b	b^{++}	b*	b^{*++}	b^{0}	b^{0++}	Seq.	y	y^{++}	y*	y^{*++}	#
1	130.0499	65.5286			112.0393	56.5233	E					12
2	258.1084	129.5579	241.0819	121.0446	240.0979	120.5526	Q	1284.5572	642.7822	1267.5307	634.2690	11
3	329.1456	165.0764	312.1190	156.5631	311.1350	156.0711	A	1156.4986	578.7530	1139.4721	570.2397	10
4	426.1983	213.6028	409.1718	205.0895	408.1878	204.5975	P	***1085.4615***	543.2344	1068.4350	534.7211	9
5	563.2572	282.1323	546.2307	273.6190	545.2467	273.1270	H	***988.4087***	494.7080	971.3822	486.1947	8
6	723.2879	362.1476	706.2613	353.6343	705.2773	353.1423	C	***851.3498***	426.1786	834.3233	417.6653	7
7	836.3720	418.6896	819.3454	410.1763	818.3614	409.6843	I	***691.3192***	346.1632	674.2926	337.6500	6

**8**	996.4026	498.7049	979.3761	490.1917	978.3920	489.6997	**C**	***578.2351***	289.6212	561.2086	281.1079	**5**
**9**	1067.4397	534.2235	1050.4132	525.7102	1049.4291	525.2182	**A**	***418.2045***	209.6059	401.1779	201.0926	**4**
**10**	1182.4667	591.7370	1165.4401	583.2237	1164.4561	582.7317	**N**	***347.1674***	174.0873	330.1408	165.5740	**3**
**11**	1239.4881	620.2477	1222.4616	611.7344	1221.4776	611.2424	**G**	***232.1404***	116.5738	215.1139	108.0606	**2**
**12**							**R**	175.1190	88.0631	158.0924	79.5498	**1**

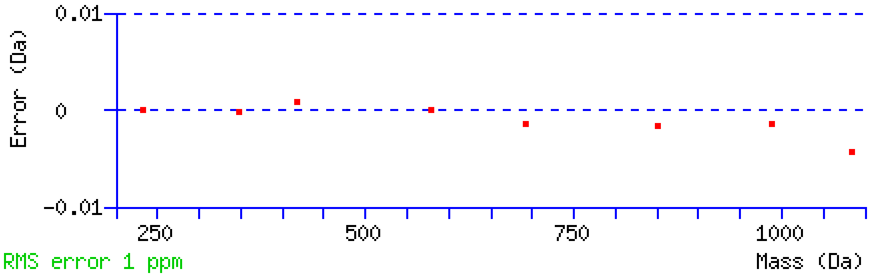

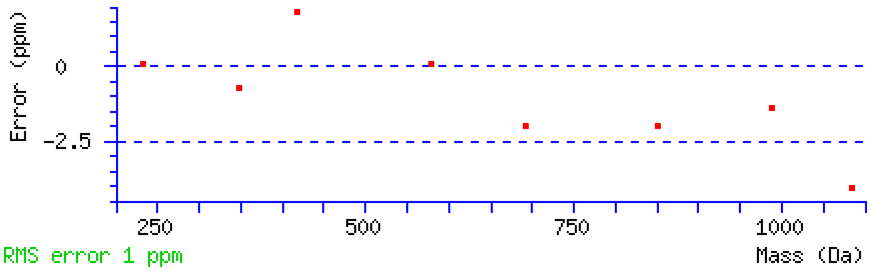

NCBI **BLAST** search of EQAPHCICANGR
(Parameters: blastp, nr protein database, expect=20000, no filter, PAM30)
Other BLAST web gateways

All matches to this query

Score	Mr(calc)	Delta	Sequence
78.3	1412.5925	0.0002	EQAPHCICANGR

Mascot: http://www.matrixscience.com/

Mascot Search Results

Peptide View

MS/MS Fragmentation of **EQAPHCICANGR**
Found in **P01023** in **uni_human_nr**, A2MG_HUMAN Alpha-2-macroglobulin OS=Homo sapiens GN=A2M PE=1 SV=3

Match to Query 3829: 1411.608912 from(471.543580,3+) intensity(39353.8516) rtinseconds(766) scans(2590) index(988)
Title: 130809_HPL_Human_Plaque_BR1_TR3_02_Spectrum025514_scans__2590_RTINSECONDS=766
Data file E:\\RAW\\HumanPlaques\\mgf\\T\\1D\\T130809_HPL_Human_Plaque_BR1_TR3_02.mgf

Click mouse within plot area to zoom in by factor of two about that point
Or, Plot from 50 to 1000 Da Full range
Label all possible matches ○ Label matches used for scoring ⦿
Show Y-axis ☐

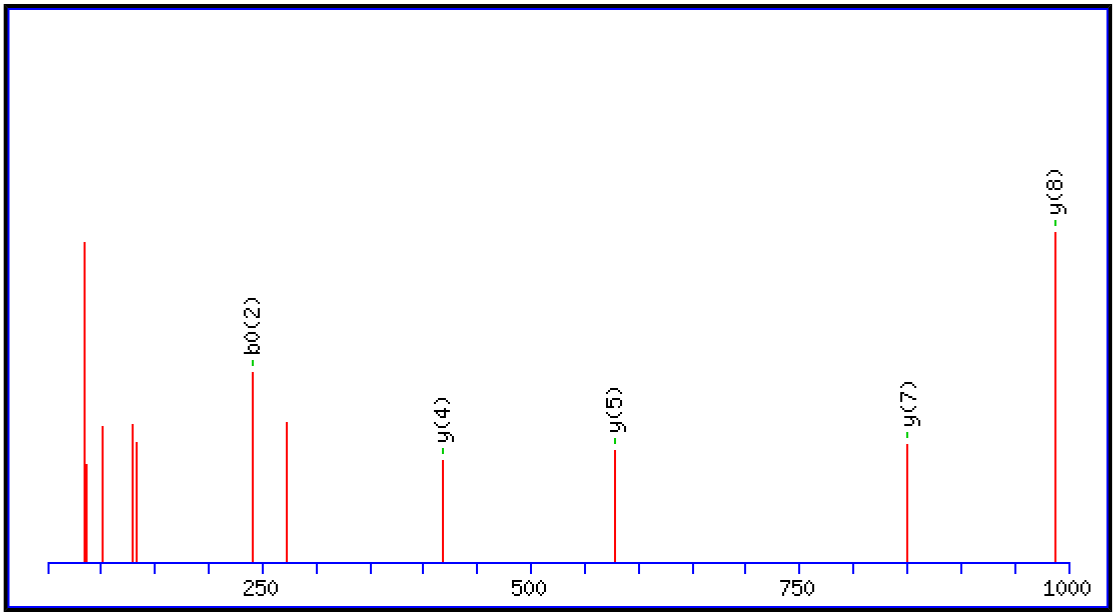

Monoisotopic mass of neutral peptide Mr(calc): 1411.6085
Fixed modifications: Carbamidomethyl (C) (apply to specified residues or termini only)
Ions Score: 26 **Expect:** 0.076
Matches : 5/108 fragment ions using 6 most intense peaks (help)

#	b	b^{++}	b*	b^{*++}	b^0	b^{0++}	Seq.	y	y^{++}	y*	y^{*++}	#
1	130.0499	65.5286			112.0393	56.5233	E					12
2	258.1084	129.5579	241.0819	121.0446	240.0979	120.5526	Q	1283.5732	642.2902	1266.5466	633.7770	11
3	329.1456	165.0764	312.1190	156.5631	311.1350	156.0711	A	1155.5146	578.2609	1138.4881	569.7477	10
4	426.1983	213.6028	409.1718	205.0895	408.1878	204.5975	P	1084.4775	542.7424	1067.4509	534.2291	9
5	563.2572	282.1323	546.2307	273.6190	545.2467	273.1270	H	***987.4247***	494.2160	970.3982	485.7027	8
6	723.2879	362.1476	706.2613	353.6343	705.2773	353.1423	C	***850.3658***	425.6865	833.3393	417.1733	7
7	836.3720	418.6896	819.3454	410.1763	818.3614	409.6843	I	690.3352	345.6712	673.3086	337.1579	6
8	996.4026	498.7049	979.3761	490.1917	978.3920	489.6997	C	***577.2511***	289.1292	560.2246	280.6159	5

**9**	1067.4397	534.2235	1050.4132	525.7102	1049.4291	525.2182	**A**	***417.2205***	209.1139	400.1939	200.6006	**4**
**10**	1181.4826	591.2450	1164.4561	582.7317	1163.4721	582.2397	**N**	346.1833	173.5953	329.1568	165.0820	**3**
**11**	1238.5041	619.7557	1221.4776	611.2424	1220.4935	610.7504	**G**	232.1404	116.5738	215.1139	108.0606	**2**
**12**							**R**	175.1190	88.0631	158.0924	79.5498	**1**

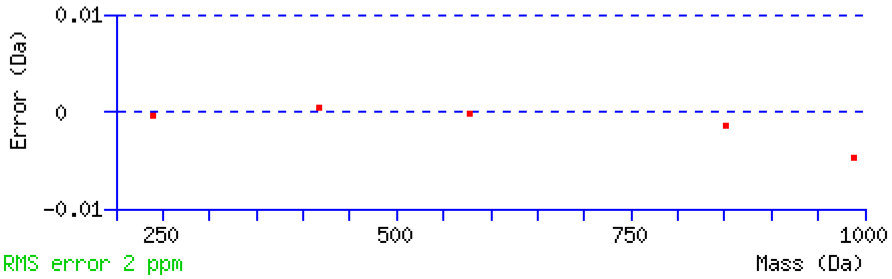

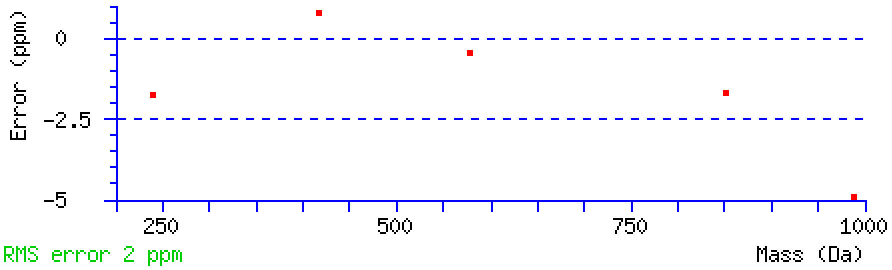

NCBI **BLAST** search of EQAPHCICANGR
(Parameters: blastp, nr protein database, expect=20000, no filter, PAM30)
Other BLAST web gateways

All matches to this query

Score	Mr(calc)	Delta	Sequence
25.6	1411.6085	0.0004	EQAPHCICANGR
3.0	1410.5986	1.0103	MNESRWTEWR

Mascot: http://www.matrixscience.com/

Mascot Search Results

Peptide View

MS/MS Fragmentation of **VSVQLEASPAFLAVPVEKEQAPHCICANGR**
Found in **P01023** in **uni_human_nr**, A2MG_HUMAN Alpha-2-macroglobulin OS=Homo sapiens GN=A2M PE=1 SV=3

Match to Query 19461: 3276.633736 from(820.165710,4+) intensity(4914648.5000) rtinseconds(2303) scans(14636) index(11454)
Title: 130801_HPL_Human_Plaque_BR2_TR1_02_Spectrum035609_scans__14636_RTINSECONDS=2303
Data file E:\\RAW\\HumanPlaques\\mgf\\T\\1D\\T130801_HPL_Human_Plaque_BR2_TR1_02.mgf

Click mouse within plot area to zoom in by factor of two about that point
Or, [Plot from] [0] to [3000] Da [Full range]
Label all possible matches ○ Label matches used for scoring ⊙
Show Y-axis ☐

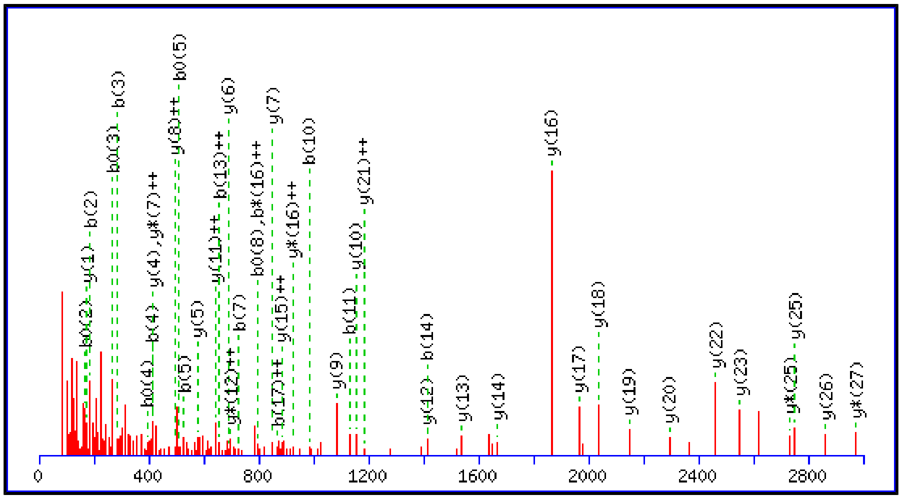

Monoisotopic mass of neutral peptide Mr(calc): 3276.6383
Fixed modifications: Carbamidomethyl (C) (apply to specified residues or termini only)
Ions Score: 82 **Expect:** 5.6e-007
Matches : 44/318 fragment ions using 119 most intense peaks (help)

#	b	b++	b*	b*++	b0	b0++	Seq.	y	y++	y*	y*++	y0	y0++	#
1	100.0757	50.5415					V							30
2	**187.1077**	94.0575			169.0972	85.0522	S	3178.5772	1589.7922	3161.5507	1581.2790	3160.5667	1580.7870	29
3	**286.1761**	143.5917			268.1656	134.5864	V	3091.5452	1546.2762	3074.5186	1537.7630	3073.5346	1537.2709	28
4	**414.2347**	207.6210	397.2082	199.1077	396.2241	198.6157	Q	2992.4768	1496.7420	2975.4502	1488.2288	2974.4662	1487.7367	27
5	**527.3188**	264.1630	510.2922	255.6498	509.3082	255.1577	L	***2864.4182***	1432.7127	2847.3916	1424.1995	2846.4076	1423.7075	26
6	656.3614	328.6843	639.3348	320.1710	638.3508	319.6790	E	***2751.3341***	1376.1707	2734.3076	1367.6574	2733.3236	1367.1654	25
7	**727.3985**	364.2029	710.3719	355.6896	709.3879	355.1976	A	2622.2915	1311.6494	2605.2650	1303.1361	2604.2810	1302.6441	24
8	814.4305	407.7189	797.4040	399.2056	796.4199	398.7136	S	***2551.2544***	1276.1309	2534.2279	1267.6176	2533.2439	1267.1256	23
9	911.4833	456.2453	894.4567	447.7320	893.4727	447.2400	P	***2464.2224***	1232.6148	2447.1958	1224.1016	2446.2118	1223.6096	22
10	**982.5204**	491.7638	965.4938	483.2506	964.5098	482.7585	A	2367.1696	1184.0885	2350.1431	1175.5752	2349.1591	1175.0832	21
11	**1129.5888**	565.2980	1112.5623	556.7848	1111.5782	556.2928	F	***2296.1325***	1148.5699	2279.1060	1140.0566	2278.1220	1139.5646	20
12	1242.6729	621.8401	1225.6463	613.3268	1224.6623	612.8348	L	***2149.0641***	1075.0357	2132.0376	1066.5224	2131.0535	1066.0304	19
13	1313.7100	**657.3586**	1296.6834	648.8454	1295.6994	648.3533	A	***2035.9800***	1018.4937	2018.9535	1009.9804	2017.9695	1009.4884	18
14	**1412.7784**	706.8928	1395.7518	698.3796	1394.7678	697.8876	V	***1964.9429***	982.9751	1947.9164	974.4618	1946.9324	973.9698	17
15	1509.8312	755.4192	1492.8046	746.9059	1491.8206	746.4139	P	***1865.8745***	933.4409	1848.8480	924.9276	1847.8639	924.4356	16
16	1608.8996	804.9534	1591.8730	796.4401	1590.8890	795.9481	V	1768.8218	884.9145	1751.7952	876.4012	1750.8112	875.9092	15
17	1737.9422	**869.4747**	1720.9156	860.9614	1719.9316	860.4694	E	***1669.7533***	835.3803	1652.7268	826.8670	1651.7428	826.3750	14
18	1866.0371	933.5222	1849.0106	925.0089	1848.0266	924.5169	K	***1540.7107***	770.8590	1523.6842	762.3457	1522.7002	761.8537	13
19	1995.0797	998.0435	1978.0532	989.5302	1977.0692	989.0382	E	***1412.6158***	706.8115	1395.5892	698.2983	1394.6052	697.8062	12

**20**	2123.1383	1062.0728	2106.1117	1053.5595	2105.1277	1053.0675	**Q**	1283.5732	642.2902	1266.5466	633.7770			**11**
**21**	2194.1754	1097.5913	2177.1489	1089.0781	2176.1648	1088.5861	**A**	***1155.5146***	578.2609	1138.4881	569.7477			**10**
**22**	2291.2282	1146.1177	2274.2016	1137.6045	2273.2176	1137.1124	**P**	***1084.4775***	542.7424	1067.4509	534.2291			**9**
**23**	2428.2871	1214.6472	2411.2605	1206.1339	2410.2765	1205.6419	**H**	987.4247	494.2160	970.3982	485.7027			**8**
**24**	2588.3177	1294.6625	2571.2912	1286.1492	2570.3072	1285.6572	**C**	***850.3658***	425.6865	833.3393	417.1733			**7**
**25**	2701.4018	1351.2045	2684.3753	1342.6913	2683.3912	1342.1993	**I**	***690.3352***	345.6712	673.3086	337.1579			**6**
**26**	2861.4325	1431.2199	2844.4059	1422.7066	2843.4219	1422.2146	**C**	***577.2511***	289.1292	560.2246	280.6159			**5**
**27**	2932.4696	1466.7384	2915.4430	1458.2251	2914.4590	1457.7331	**A**	***417.2205***	209.1139	400.1939	200.6006			**4**
**28**	3046.5125	1523.7599	3029.4859	1515.2466	3028.5019	1514.7546	**N**	346.1833	173.5953	329.1568	165.0820			**3**
**29**	3103.5340	1552.2706	3086.5074	1543.7573	3085.5234	1543.2653	**G**	232.1404	116.5738	215.1139	108.0606			**2**
**30**							**R**	***175.1190***	88.0631	158.0924	79.5498			**1**

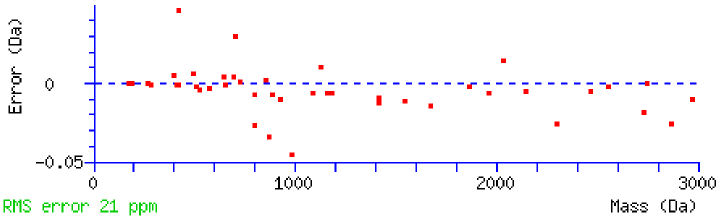

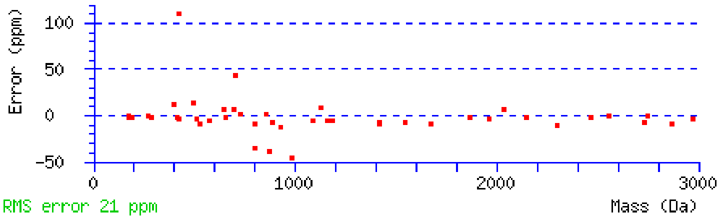

NCBI **BLAST** search of VSVQLEASPAFLAVPVEKEQAPHCICANGR
(Parameters: blastp, nr protein database, expect=20000, no filter, PAM30)
Other BLAST web gateways

All matches to this query

Score	Mr(calc)	Delta	Sequence
81.5	3276.6383	-0.0046	VSVQLEASPAFLAVPVEKEQAPHCICANGR

Mascot: http://www.matrixscience.com/

Mascot Search Results

Peptide View

MS/MS Fragmentation of **IPTTFENGR**
Found in **P05090** in **uni_human_nr**, APOD_HUMAN Apolipoprotein D OS=Homo sapiens GN=APOD PE=1 SV=1

Match to Query 574: 1034.504368 from(518.259460,2+) intensity(789903.0000) rtinseconds(1382) scans(6867) index(4721)
Title: 130809_HPL_Human_Plaque_BR2_TR3_08_Spectrum026219_scans__6867_RTINSECONDS=1382
Data file E:\\RAW\\HumanPlaques\\mgf\\T\\1D\\T130809_HPL_Human_Plaque_BR2_TR3_08.mgf

Click mouse within plot area to zoom in by factor of two about that point
Or, Plot from 50 to 950 Da Full range
Label all possible matches ○ Label matches used for scoring ⦿
Show Y-axis ☐

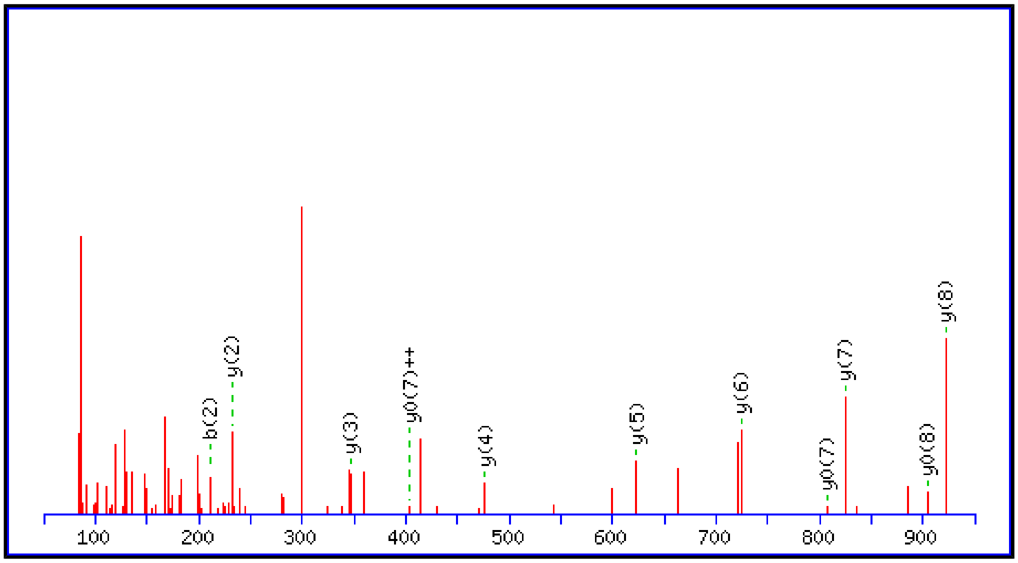

Monoisotopic mass of neutral peptide Mr(calc): 1034.5033
Fixed modifications: Carbamidomethyl (C) (apply to specified residues or termini only)
Variable modifications:
N7 : Deamidated (NQ)
Ions Score: 37 **Expect:** 0.015
Matches : 11/74 fragment ions using 29 most intense peaks (help)

#	b	b^{++}	b*	b^{*++}	b^0	b^{0++}	Seq.	y	y^{++}	y*	y^{*++}	y^0	y^{0++}	#
1	114.0913	57.5493					I							9
2	**211.1441**	106.0757					P	***922.4265***	461.7169	905.3999	453.2036	904.4159	452.7116	8
3	312.1918	156.5995			294.1812	147.5942	T	***825.3737***	413.1905	808.3472	404.6772	807.3632	404.1852	7
4	413.2395	207.1234			395.2289	198.1181	T	***724.3260***	362.6667	707.2995	354.1534	706.3155	353.6614	6
5	560.3079	280.6576			542.2973	271.6523	F	***623.2784***	312.1428	606.2518	303.6295	605.2678	303.1375	5
6	689.3505	345.1789			671.3399	336.1736	E	***476.2100***	238.6086	459.1834	230.0953	458.1994	229.6033	4
7	804.3774	402.6923	787.3509	394.1791	786.3668	393.6871	N	***347.1674***	174.0873	330.1408	165.5740			3
8	861.3989	431.2031	844.3723	422.6898	843.3883	422.1978	G	***232.1404***	116.5738	215.1139	108.0606			2
9							R	175.1190	88.0631	158.0924	79.5498			1

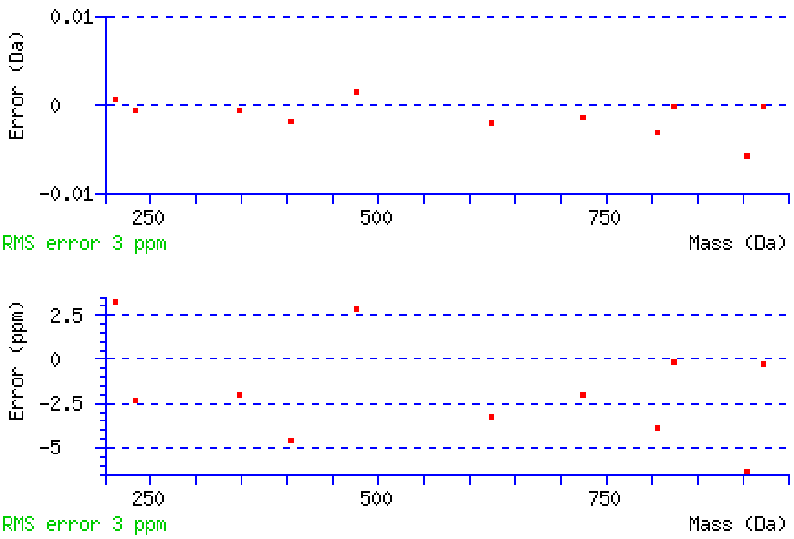

NCBI **BLAST** search of IPTTFENGR
(Parameters: blastp, nr protein database, expect=20000, no filter, PAM30)
Other BLAST web gateways

All matches to this query

Score	Mr(calc)	Delta	Sequence
36.9	1034.5033	0.0011	IPTTFENGR

Mascot: http://www.matrixscience.com/

Mascot Search Results

Peptide View

MS/MS Fragmentation of **WYEIEKIPTTFENGR**
Found in **P05090** in **uni_human_nr**, APOD_HUMAN Apolipoprotein D OS=Homo sapiens GN=APOD PE=1 SV=1

Match to Query 7108: 1882.911042 from(628.644290,3+) intensity(8612535.0000) rtinseconds(2502) scans(14826) index(11293)
Title: 130806_HPL_Human_Plaque_BR1_TR2_07_Spectrum033264_scans__14826_RTINSECONDS=2502
Data file E:\\RAW\\HumanPlaques\\mgf\\T\\1D\\T130806_HPL_Human_Plaque_BR1_TR2_07.mgf

Click mouse within plot area to zoom in by factor of two about that point
Or, Plot from 0 to 1700 Da Full range
Label all possible matches ○ Label matches used for scoring ⊙
Show Y-axis ☐

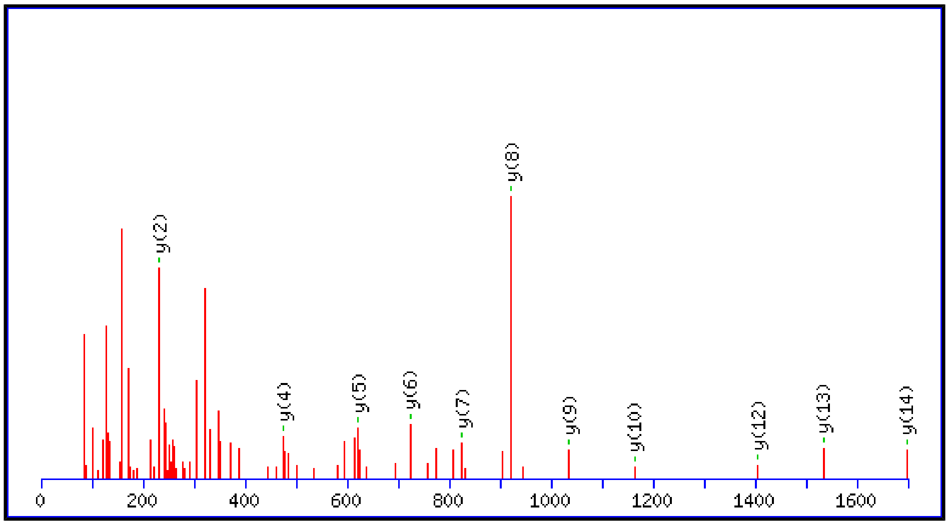

Monoisotopic mass of neutral peptide Mr(calc): 1882.9101
Fixed modifications: Carbamidomethyl (C) (apply to specified residues or termini only)
Variable modifications:
N13 : Deamidated (NQ)
Ions Score: 99 **Expect:** 8.5e-009
Matches : 11/148 fragment ions using 14 most intense peaks (help)

#	b	b^{++}	b*	b^{*++}	b^0	b^{0++}	Seq.	y	y^{++}	y*	y^{*++}	y^0	y^{0++}	#
1	187.0866	94.0469					W							15
2	350.1499	175.5786					Y	***1697.8381***	849.4227	1680.8115	840.9094	1679.8275	840.4174	14
3	479.1925	240.0999			461.1819	231.0946	E	***1534.7748***	767.8910	1517.7482	759.3777	1516.7642	758.8857	13
4	592.2766	296.6419			574.2660	287.6366	I	***1405.7322***	703.3697	1388.7056	694.8564	1387.7216	694.3644	12
5	721.3192	361.1632			703.3086	352.1579	E	1292.6481	646.8277	1275.6216	638.3144	1274.6375	637.8224	11
6	849.4141	425.2107	832.3876	416.6974	831.4036	416.2054	K	***1163.6055***	582.3064	1146.5790	573.7931	1145.5950	573.3011	10
7	962.4982	481.7527	945.4716	473.2395	944.4876	472.7475	I	***1035.5106***	518.2589	1018.4840	509.7456	1017.5000	509.2536	9
8	1059.5510	530.2791	1042.5244	521.7658	1041.5404	521.2738	P	***922.4265***	461.7169	905.3999	453.2036	904.4159	452.7116	8
9	1160.5986	580.8030	1143.5721	572.2897	1142.5881	571.7977	T	***825.3737***	413.1905	808.3472	404.6772	807.3632	404.1852	7
10	1261.6463	631.3268	1244.6198	622.8135	1243.6358	622.3215	T	***724.3260***	362.6667	707.2995	354.1534	706.3155	353.6614	6
11	1408.7147	704.8610	1391.6882	696.3477	1390.7042	695.8557	F	***623.2784***	312.1428	606.2518	303.6295	605.2678	303.1375	5
12	1537.7573	769.3823	1520.7308	760.8690	1519.7468	760.3770	E	***476.2100***	238.6086	459.1834	230.0953	458.1994	229.6033	4
13	1652.7843	826.8958	1635.7577	818.3825	1634.7737	817.8905	N	347.1674	174.0873	330.1408	165.5740			3
14	1709.8057	855.4065	1692.7792	846.8932	1691.7952	846.4012	G	***232.1404***	116.5738	215.1139	108.0606			2
15							R	175.1190	88.0631	158.0924	79.5498			1

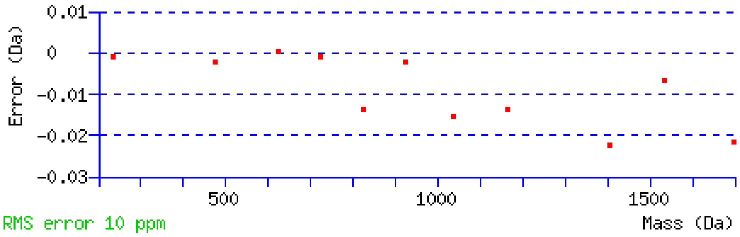

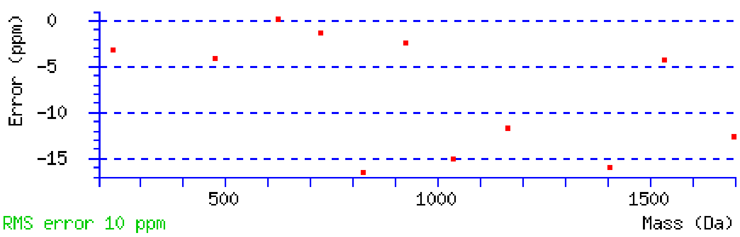

NCBI **BLAST** search of WYEIEKIPTTFENGR
(Parameters: blastp, nr protein database, expect=20000, no filter, PAM30)
Other BLAST web gateways

All matches to this query

Score	Mr(calc)	Delta	Sequence
99.5	1882.9101	0.0009	WYEIEKIPTTFENGR
9.0	1881.9109	1.0002	KLLSSGFDIDTPDDFGR
3.5	1881.9108	1.0002	LEQELFSGGNTGINFEK

Mascot: http://www.matrixscience.com/

Mascot Search Results

Peptide View

MS/MS Fragmentation of **WYEIEKIPTTFENGR**
Found in **P05090** in **uni_human_nr**, APOD_HUMAN Apolipoprotein D OS=Homo sapiens GN=APOD PE=1 SV=1

Match to Query 7798: 1882.908308 from(942.461430,2+) intensity(3155450.7500) rtinseconds(2465) scans(15491) index(12383)
Title: 130806_HPL_Human_Plaque_BR1_TR2_04_Spectrum035797_scans__15491_RTINSECONDS=2465
Data file E:\\RAW\\HumanPlaques\\mgf\\T\\1D\\T130806_HPL_Human_Plaque_BR1_TR2_04.mgf

Click mouse within plot area to zoom in by factor of two about that point
Or, [Plot from] [0] to [1900] Da [Full range]
Label all possible matches ○ Label matches used for scoring ⦿
Show Y-axis ☐

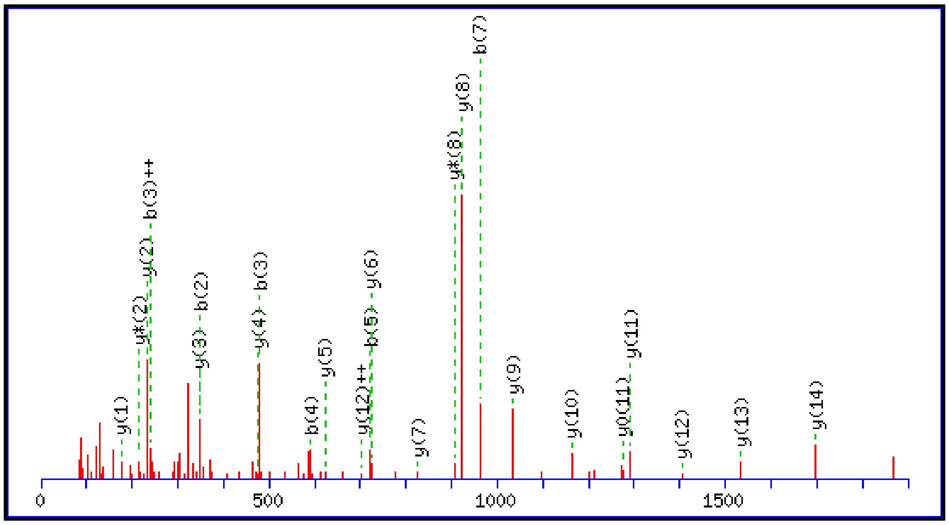

Monoisotopic mass of neutral peptide Mr(calc): 1882.9101
Fixed modifications: Carbamidomethyl (C) (apply to specified residues or termini only)
Variable modifications:
N13 : Deamidated (NQ)
Ions Score: 87 **Expect:** 1.5e-007
Matches : 24/148 fragment ions using 58 most intense peaks (help)

#	b	b^{++}	b*	b^{*++}	b^0	b^{0++}	Seq.	y	y^{++}	y*	y^{*++}	y^0	y^{0++}	#
1	187.0866	94.0469					W							15
2	**350.1499**	175.5786					Y	***1697.8381***	849.4227	1680.8115	840.9094	1679.8275	840.4174	14
3	**479.1925**	**240.0999**			461.1819	231.0946	E	***1534.7748***	767.8910	1517.7482	759.3777	1516.7642	758.8857	13
4	**592.2766**	296.6419			574.2660	287.6366	I	***1405.7322***	703.3697	1388.7056	694.8564	1387.7216	694.3644	12
5	**721.3192**	361.1632			703.3086	352.1579	E	***1292.6481***	646.8277	1275.6216	638.3144	1274.6375	637.8224	11
6	849.4141	425.2107	832.3876	416.6974	831.4036	416.2054	K	***1163.6055***	582.3064	1146.5790	573.7931	1145.5950	573.3011	10
7	**962.4982**	481.7527	945.4716	473.2395	944.4876	472.7475	I	***1035.5106***	518.2589	1018.4840	509.7456	1017.5000	509.2536	9
8	1059.5510	530.2791	1042.5244	521.7658	1041.5404	521.2738	P	***922.4265***	461.7169	905.3999	453.2036	904.4159	452.7116	8
9	1160.5986	580.8030	1143.5721	572.2897	1142.5881	571.7977	T	***825.3737***	413.1905	808.3472	404.6772	807.3632	404.1852	7
10	1261.6463	631.3268	1244.6198	622.8135	1243.6358	622.3215	T	***724.3260***	362.6667	707.2995	354.1534	706.3155	353.6614	6
11	1408.7147	704.8610	1391.6882	696.3477	1390.7042	695.8557	F	***623.2784***	312.1428	606.2518	303.6295	605.2678	303.1375	5
12	1537.7573	769.3823	1520.7308	760.8690	1519.7468	760.3770	E	***476.2100***	238.6086	459.1834	230.0953	458.1994	229.6033	4
13	1652.7843	826.8958	1635.7577	818.3825	1634.7737	817.8905	N	***347.1674***	174.0873	330.1408	165.5740			3
14	1709.8057	855.4065	1692.7792	846.8932	1691.7952	846.4012	G	***232.1404***	116.5738	215.1139	108.0606			2
15							R	***175.1190***	88.0631	158.0924	79.5498			1

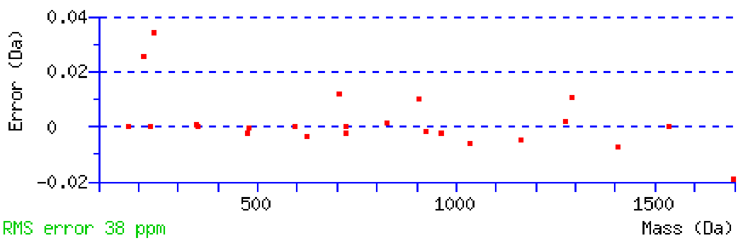

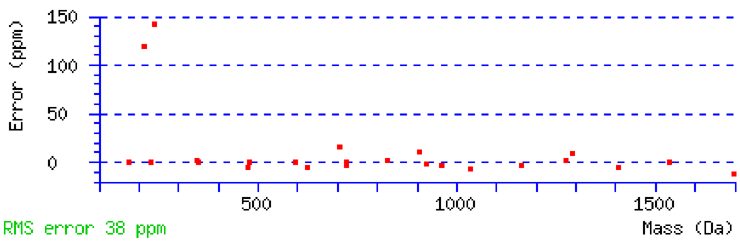

NCBI **BLAST** search of WYEIEKIPTTFENGR
(Parameters: blastp, nr protein database, expect=20000, no filter, PAM30)
Other BLAST web gateways

All matches to this query

Score	Mr(calc)	Delta	Sequence
87.1	1882.9101	-0.0018	WYEIEKIPTTFENGR
6.4	1881.9109	0.9974	KLLSSGFDIDTPDDFGR
0.9	1880.8971	2.0112	MSARQKQIMEIEEQK
0.8	1880.8976	2.0107	QHLEEQRLQQERQR
0.5	1880.8971	2.0112	MSARQKQIMEIEEQK

Mascot: http://www.matrixscience.com/

Mascot Search Results

Peptide View

MS/MS Fragmentation of **WYEIEKIPTTFENGR**
Found in **P05090** in **uni_human_nr**, APOD_HUMAN Apolipoprotein D OS=Homo sapiens GN=APOD PE=1 SV=1

Match to Query 8625: 1881.926128 from(941.970340,2+) intensity(3746826.7500) rtinseconds(2295) scans(14557) index(11382)
Title: 130801_HPL_Human_Plaque_BR2_TR1_02_Spectrum035537_scans__14557_RTINSECONDS=2295
Data file E:\\RAW\\HumanPlaques\\mgf\\T\\1D\\T130801_HPL_Human_Plaque_BR2_TR1_02.mgf

Click mouse within plot area to zoom in by factor of two about that point
Or, Plot from 0 to 1900 Da Full range
Label all possible matches ○ Label matches used for scoring ⦿
Show Y-axis ☐

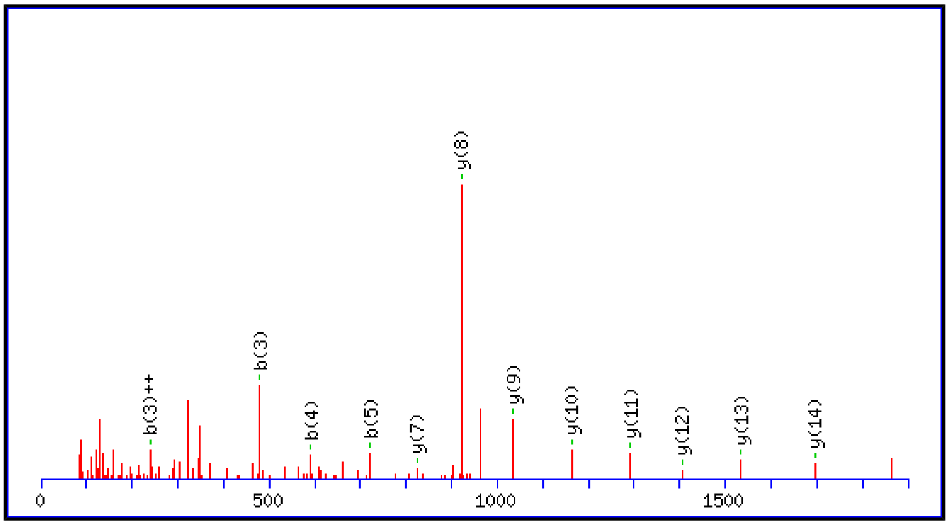

Monoisotopic mass of neutral peptide Mr(calc): 1881.9261
Fixed modifications: Carbamidomethyl (C) (apply to specified residues or termini only)
Ions Score: 65 **Expect:** 2.3e-005
Matches : 12/148 fragment ions using 16 most intense peaks (help)

#	b	b^{++}	b*	b^{*++}	b^0	b^{0++}	Seq.	y	y^{++}	y*	y^{*++}	y^0	y^{0++}	#
1	187.0866	94.0469					W							15
2	350.1499	175.5786					Y	***1696.8541***	848.9307	1679.8275	840.4174	1678.8435	839.9254	14
3	**479.1925**	**240.0999**			461.1819	231.0946	E	***1533.7907***	767.3990	1516.7642	758.8857	1515.7802	758.3937	13
4	**592.2766**	296.6419			574.2660	287.6366	I	***1404.7482***	702.8777	1387.7216	694.3644	1386.7376	693.8724	12
5	**721.3192**	361.1632			703.3086	352.1579	E	***1291.6641***	646.3357	1274.6375	637.8224	1273.6535	637.3304	11
6	849.4141	425.2107	832.3876	416.6974	831.4036	416.2054	K	***1162.6215***	581.8144	1145.5950	573.3011	1144.6109	572.8091	10
7	962.4982	481.7527	945.4716	473.2395	944.4876	472.7475	I	***1034.5265***	517.7669	1017.5000	509.2536	1016.5160	508.7616	9
8	1059.5510	530.2791	1042.5244	521.7658	1041.5404	521.2738	P	***921.4425***	461.2249	904.4159	452.7116	903.4319	452.2196	8
9	1160.5986	580.8030	1143.5721	572.2897	1142.5881	571.7977	T	***824.3897***	412.6985	807.3632	404.1852	806.3791	403.6932	7
10	1261.6463	631.3268	1244.6198	622.8135	1243.6358	622.3215	T	723.3420	362.1747	706.3155	353.6614	705.3315	353.1694	6
11	1408.7147	704.8610	1391.6882	696.3477	1390.7042	695.8557	F	622.2944	311.6508	605.2678	303.1375	604.2838	302.6455	5
12	1537.7573	769.3823	1520.7308	760.8690	1519.7468	760.3770	E	475.2259	238.1166	458.1994	229.6033	457.2154	229.1113	4
13	1651.8003	826.4038	1634.7737	817.8905	1633.7897	817.3985	N	346.1833	173.5953	329.1568	165.0820			3
14	1708.8217	854.9145	1691.7952	846.4012	1690.8112	845.9092	G	232.1404	116.5738	215.1139	108.0606			2
15							R	175.1190	88.0631	158.0924	79.5498			1

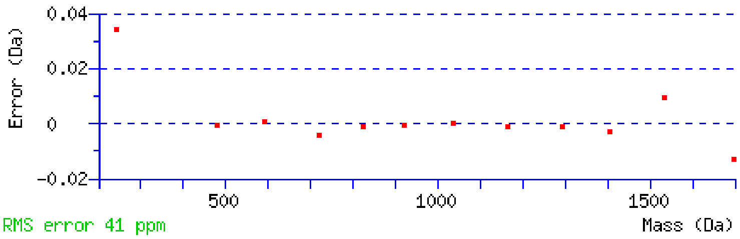

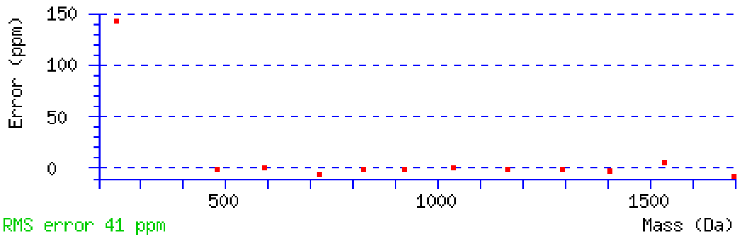

NCBI **BLAST** search of WYEIEKIPTTFENGR
(Parameters: blastp, nr protein database, expect=20000, no filter, PAM30)
Other BLAST web gateways

All matches to this query

Score	Mr(calc)	Delta	Sequence
65.5	1881.9261	0.0000	WYEIEKIPTTFENGR
1.7	1879.9136	2.0125	QHLEEQRLQQERQR
1.0	1880.9156	1.0106	NGLVNASYTKLQEWEK
1.0	1880.9156	1.0106	NGLVNASYTKLQEWEK
0.2	1879.9172	2.0090	MAEQVLPQALYLSNMR

Mascot: http://www.matrixscience.com/

Mascot Search Results

Peptide View

MS/MS Fragmentation of **WYEIEKIPTTFENGR**
Found in **P05090** in **uni_human_nr**, APOD_HUMAN Apolipoprotein D OS=Homo sapiens GN=APOD PE=1 SV=1

Match to Query 8626: 1881.928302 from(628.316710,3+) intensity(57068824.0000) rtinseconds(2298) scans(14583) index(11406)
Title: 130801_HPL_Human_Plaque_BR2_TR1_02_Spectrum035561_scans__14583_RTINSECONDS=2298
Data file E:\\RAW\\HumanPlaques\\mgf\\T\\1D\\T130801_HPL_Human_Plaque_BR2_TR1_02.mgf

Click mouse within plot area to zoom in by factor of two about that point
Or, Plot from 0 to 1700 Da Full range
Label all possible matches ○ Label matches used for scoring ⊙
Show Y-axis ☐

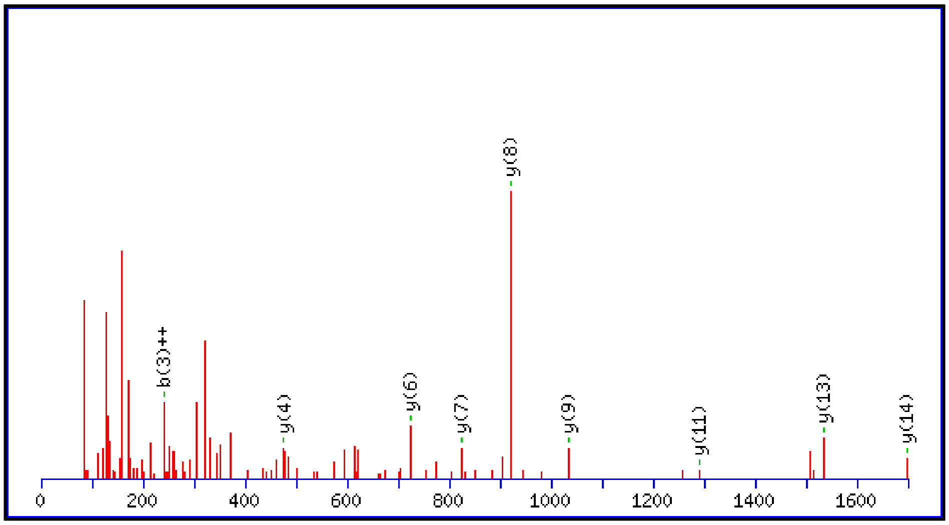

Monoisotopic mass of neutral peptide Mr(calc): 1881.9261
Fixed modifications: Carbamidomethyl (C) (apply to specified residues or termini only)
Ions Score: 54 **Expect:** 0.00031
Matches : 9/148 fragment ions using 14 most intense peaks (help)

#	b	b++	b*	b*++	b0	b0++	Seq.	y	y++	y*	y*++	y0	y0++	#
1	187.0866	94.0469					W							15
2	350.1499	175.5786					Y	***1696.8541***	848.9307	1679.8275	840.4174	1678.8435	839.9254	14
3	479.1925	**240.0999**			461.1819	231.0946	E	***1533.7907***	767.3990	1516.7642	758.8857	1515.7802	758.3937	13
4	592.2766	296.6419			574.2660	287.6366	I	1404.7482	702.8777	1387.7216	694.3644	1386.7376	693.8724	12
5	721.3192	361.1632			703.3086	352.1579	E	***1291.6641***	646.3357	1274.6375	637.8224	1273.6535	637.3304	11
6	849.4141	425.2107	832.3876	416.6974	831.4036	416.2054	K	1162.6215	581.8144	1145.5950	573.3011	1144.6109	572.8091	10
7	962.4982	481.7527	945.4716	473.2395	944.4876	472.7475	I	***1034.5265***	517.7669	1017.5000	509.2536	1016.5160	508.7616	9
8	1059.5510	530.2791	1042.5244	521.7658	1041.5404	521.2738	P	***921.4425***	461.2249	904.4159	452.7116	903.4319	452.2196	8
9	1160.5986	580.8030	1143.5721	572.2897	1142.5881	571.7977	T	***824.3897***	412.6985	807.3632	404.1852	806.3791	403.6932	7
10	1261.6463	631.3268	1244.6198	622.8135	1243.6358	622.3215	T	***723.3420***	362.1747	706.3155	353.6614	705.3315	353.1694	6
11	1408.7147	704.8610	1391.6882	696.3477	1390.7042	695.8557	F	622.2944	311.6508	605.2678	303.1375	604.2838	302.6455	5
12	1537.7573	769.3823	1520.7308	760.8690	1519.7468	760.3770	E	***475.2259***	238.1166	458.1994	229.6033	457.2154	229.1113	4
13	1651.8003	826.4038	1634.7737	817.8905	1633.7897	817.3985	N	346.1833	173.5953	329.1568	165.0820			3
14	1708.8217	854.9145	1691.7952	846.4012	1690.8112	845.9092	G	232.1404	116.5738	215.1139	108.0606			2
15							R	175.1190	88.0631	158.0924	79.5498			1

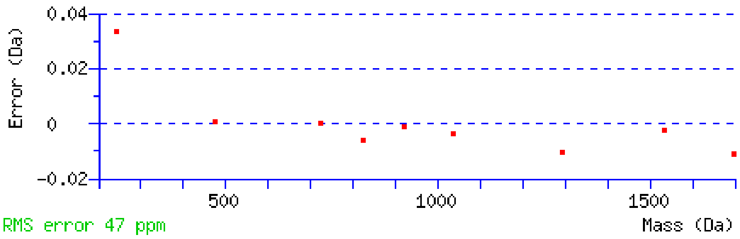

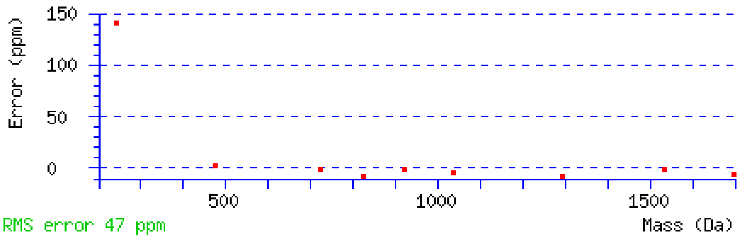

NCBI **BLAST** search of WYEIEKIPTTFENGR
(Parameters: blastp, nr protein database, expect=20000, no filter, PAM30)
Other BLAST web gateways

All matches to this query

Score	Mr(calc)	Delta	Sequence
54.5	1881.9261	0.0022	WYEIEKIPTTFENGR

**Mascot:** http://www.matrixscience.com/

Mascot Search Results

Peptide View

MS/MS Fragmentation of **MNINGQWEGEVNGR**
Found in **P46109** in **uni_human_nr**, CRKL_HUMAN Crk-like protein OS=Homo sapiens GN=CRKL PE=1 SV=1

Match to Query 4416: 1603.705168 from(802.859860,2+) intensity(138766.9531) rtinseconds(2017) scans(11262) index(8427)
Title: 130809_HPL_Human_Plaque_BR1_TR3_10_Spectrum029963_scans__11262_RTINSECONDS=2017
Data file E:\\RAW\\HumanPlaques\\mgf\\T\\1D\\T130809_HPL_Human_Plaque_BR1_TR3_10.mgf

Click mouse within plot area to zoom in by factor of two about that point
Or, [Plot from] [50] to [950] Da [Full range]
Label all possible matches ○ Label matches used for scoring ⊙
Show Y-axis ☐

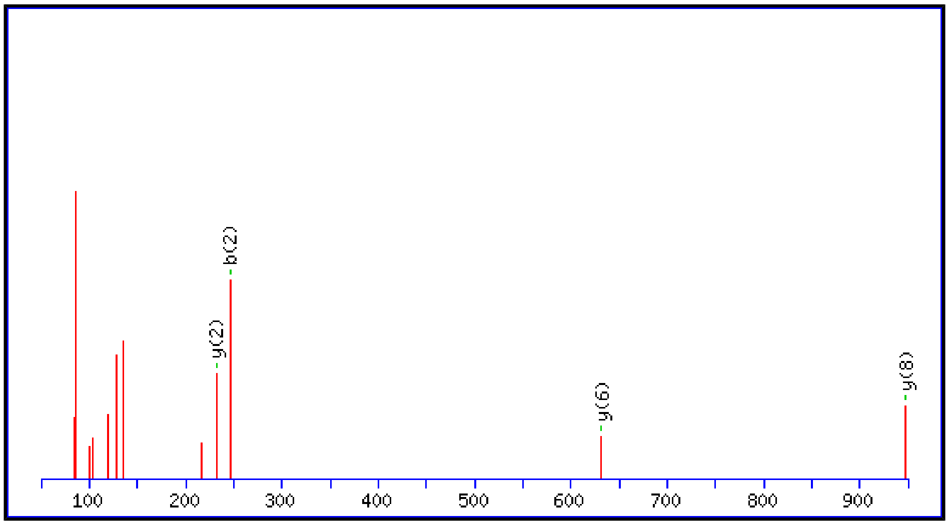

Monoisotopic mass of neutral peptide Mr(calc): 1603.7049
Fixed modifications: Carbamidomethyl (C) (apply to specified residues or termini only)
Variable modifications:
N12 : Deamidated (NQ)
Ions Score: 15 **Expect:** 0.78
Matches : 4/132 fragment ions using 6 most intense peaks (help)

#	b	b^{++}	b*	b^{*++}	b^{0}	b^{0++}	Seq.	y	y^{++}	y*	y^{*++}	y^{0}	y^{0++}	#
1	132.0478	66.5275					M							14
2	**246.0907**	123.5490	229.0641	115.0357			N	1473.6717	737.3395	1456.6451	728.8262	1455.6611	728.3342	13
3	359.1748	180.0910	342.1482	171.5777			I	1359.6288	680.3180	1342.6022	671.8047	1341.6182	671.3127	12
4	473.2177	237.1125	456.1911	228.5992			N	1246.5447	623.7760	1229.5182	615.2627	1228.5341	614.7707	11
5	530.2391	265.6232	513.2126	257.1099			G	1132.5018	566.7545	1115.4752	558.2413	1114.4912	557.7492	10
6	658.2977	329.6525	641.2712	321.1392			Q	1075.4803	538.2438	1058.4538	529.7305	1057.4697	529.2385	9
7	844.3770	422.6922	827.3505	414.1789			W	***947.4217***	474.2145	930.3952	465.7012	929.4112	465.2092	8
8	973.4196	487.2135	956.3931	478.7002	955.4091	478.2082	E	761.3424	381.1748	744.3159	372.6616	743.3319	372.1696	7
9	1030.4411	515.7242	1013.4145	507.2109	1012.4305	506.7189	G	***632.2998***	316.6536	615.2733	308.1403	614.2893	307.6483	6
10	1159.4837	580.2455	1142.4571	571.7322	1141.4731	571.2402	E	575.2784	288.1428	558.2518	279.6295	557.2678	279.1375	5
11	1258.5521	629.7797	1241.5255	621.2664	1240.5415	620.7744	V	446.2358	223.6215	429.2092	215.1083			4
12	1373.5790	687.2932	1356.5525	678.7799	1355.5685	678.2879	N	347.1674	174.0873	330.1408	165.5740			3
13	1430.6005	715.8039	1413.5740	707.2906	1412.5899	706.7986	G	***232.1404***	116.5738	215.1139	108.0606			2
14							R	175.1190	88.0631	158.0924	79.5498			1

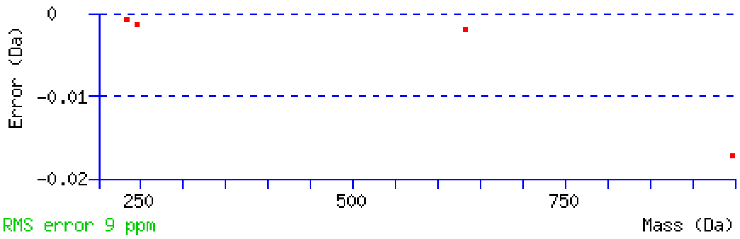

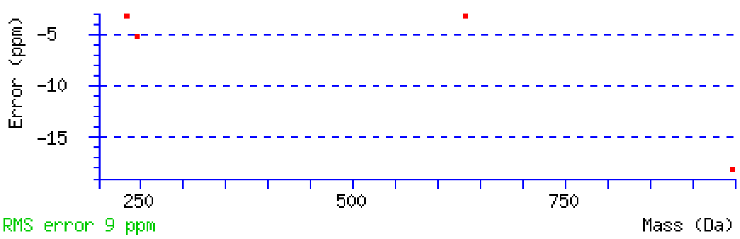

NCBI **BLAST** search of MNINGQWEGEVNGR
(Parameters: blastp, nr protein database, expect=20000, no filter, PAM30)
Other BLAST web gateways

All matches to this query

Score	Mr(calc)	Delta	Sequence
15.4	1603.7049	0.0003	MNINGQWEGEVNGR

Mascot: http://www.matrixscience.com/

Mascot Search Results

Peptide View

MS/MS Fragmentation of **AADFIDQALAQKNGR**
Found in **P51452** in **uni_human_nr**, DUS3_HUMAN Dual specificity protein phosphatase 3 OS=Homo sapiens GN=DUSP3 PE=1 SV=1

Match to Query 4308: 1617.812022 from(540.277950,3+) intensity(667883.1875) rtinseconds(2021) scans(11311) index(8395)
Title: 130809_HPL_Human_Plaque_BR2_TR3_08_Spectrum029893_scans__11311_RTINSECONDS=2021
Data file E:\\RAW\\HumanPlaques\\mgf\\T\\1D\\T130809_HPL_Human_Plaque_BR2_TR3_08.mgf

Click mouse within plot area to zoom in by factor of two about that point
Or, Plot from 0 to 1500 Da Full range
Label all possible matches ○ Label matches used for scoring ⊙
Show Y-axis ☐

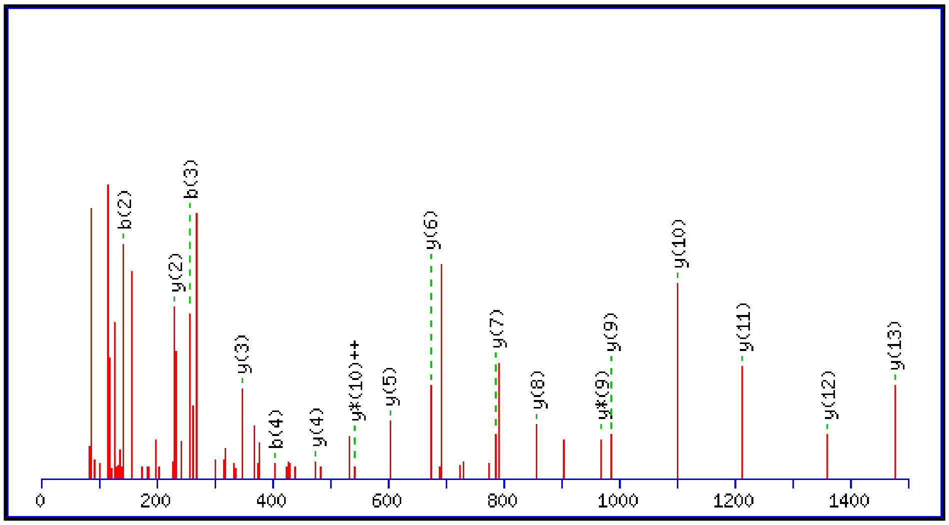

Monoisotopic mass of neutral peptide Mr(calc): 1617.8111
Fixed modifications: Carbamidomethyl (C) (apply to specified residues or termini only)
Variable modifications:
N13 : Deamidated (NQ)
Ions Score: 91 **Expect:** 7e-008
Matches : 17/134 fragment ions using 32 most intense peaks (help)

#	b	b^{++}	b*	b^{*++}	b^0	b^{0++}	Seq.	y	y^{++}	y*	y^{*++}	y^0	y^{0++}	#
1	72.0444	36.5258					A							15
2	**143.0815**	72.0444					A	1547.7812	774.3943	1530.7547	765.8810	1529.7707	765.3890	14
3	**258.1084**	129.5579			240.0979	120.5526	D	***1476.7441***	738.8757	1459.7176	730.3624	1458.7336	729.8704	13
4	**405.1769**	203.0921			387.1663	194.0868	F	***1361.7172***	681.3622	1344.6906	672.8490	1343.7066	672.3570	12
5	518.2609	259.6341			500.2504	250.6288	I	***1214.6488***	607.8280	1197.6222	599.3148	1196.6382	598.8227	11
6	633.2879	317.1476			615.2773	308.1423	D	***1101.5647***	551.2860	1084.5382	542.7727	1083.5541	542.2807	10
7	761.3464	381.1769	744.3199	372.6636	743.3359	372.1716	Q	***986.5378***	493.7725	969.5112	485.2592			9
8	832.3836	416.6954	815.3570	408.1821	814.3730	407.6901	A	***858.4792***	429.7432	841.4526	421.2300			8
9	945.4676	473.2375	928.4411	464.7242	927.4571	464.2322	L	***787.4421***	394.2247	770.4155	385.7114			7
10	1016.5047	508.7560	999.4782	500.2427	998.4942	499.7507	A	***674.3580***	337.6826	657.3315	329.1694			6
11	1144.5633	572.7853	1127.5368	564.2720	1126.5528	563.7800	Q	***603.3209***	302.1641	586.2944	293.6508			5
12	1272.6583	636.8328	1255.6317	628.3195	1254.6477	627.8275	K	***475.2623***	238.1348	458.2358	229.6215			4
13	1387.6852	694.3462	1370.6587	685.8330	1369.6747	685.3410	N	***347.1674***	174.0873	330.1408	165.5740			3
14	1444.7067	722.8570	1427.6801	714.3437	1426.6961	713.8517	G	***232.1404***	116.5738	215.1139	108.0606			2
15							R	175.1190	88.0631	158.0924	79.5498			1

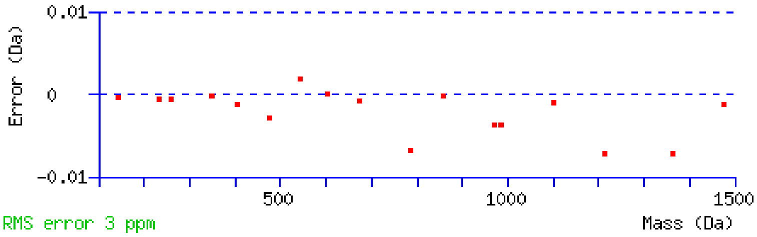

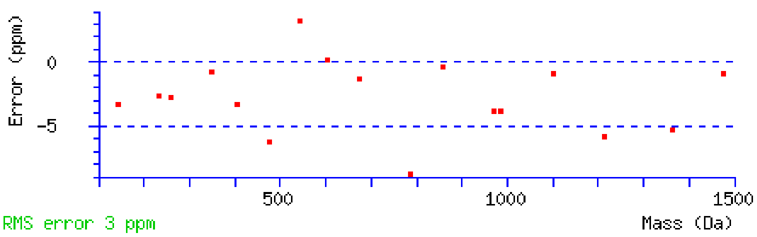

NCBI **BLAST** search of AADFIDQALAQKNGR
(Parameters: blastp, nr protein database, expect=20000, no filter, PAM30)
Other BLAST web gateways

All matches to this query

Score	Mr(calc)	Delta	Sequence	Site Analysis
90.8	1617.8111	0.0010	AADFIDQALAQKNGR	Deamidated N13 99.86%
62.3	1617.8111	0.0010	AADFIDQALAQKNGR	Deamidated Q11 0.14%
20.2	1617.8111	0.0010	AADFIDQALAQKNGR	Deamidated Q7 0.00%

Mascot: http://www.matrixscience.com/

Mascot Search Results

Peptide View

MS/MS Fragmentation of **AADFIDQALAQKNGR**
Found in **P51452** in **uni_human_nr**, DUS3_HUMAN Dual specificity protein phosphatase 3 OS=Homo sapiens GN=DUSP3 PE=1 SV=1

Match to Query 5819: 1616.827302 from(539.949710,3+) intensity(1461600.6250) rtinseconds(1776) scans(10305) index(7673)
Title: 130801_HPL_Human_Plaque_BR1_TR1_02_Spectrum031680_scans__10305_RTINSECONDS=1776
Data file E:\\RAW\\HumanPlaques\\mgf\\T\\1D\\T130801_HPL_Human_Plaque_BR1_TR1_02.mgf

Click mouse within plot area to zoom in by factor of two about that point
Or, [Plot from] [0] to [1600] Da [Full range]
Label all possible matches ○ Label matches used for scoring ⊙
Show Y-axis ☐

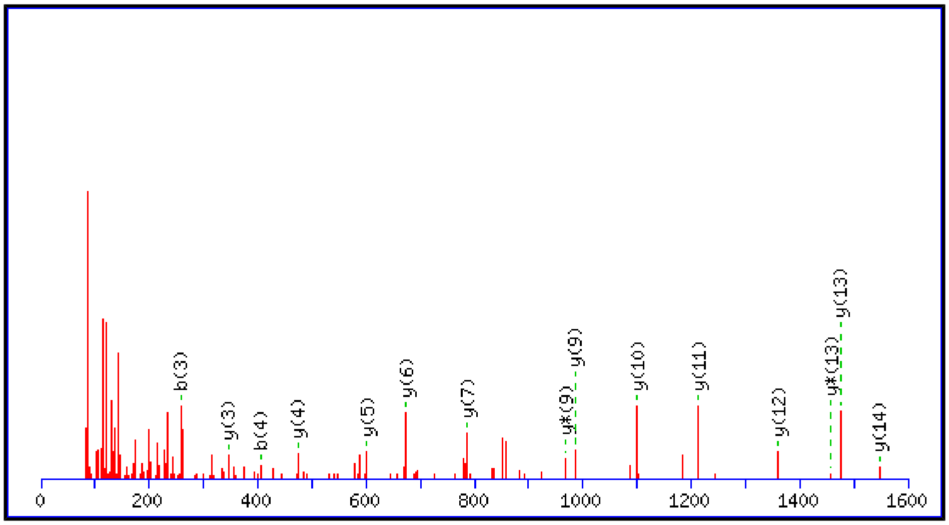

Monoisotopic mass of neutral peptide Mr(calc): 1616.8270
Fixed modifications: Carbamidomethyl (C) (apply to specified residues or termini only)
Ions Score: 82 **Expect:** 5.4e-007
Matches : 15/134 fragment ions using 28 most intense peaks (help)

#	b	b^{++}	b*	b^{*++}	b^0	b^{0++}	Seq.	y	y^{++}	y*	y^{*++}	y^0	y^{0++}	#
1	72.0444	36.5258					A							15
2	143.0815	72.0444					A	***1546.7972***	773.9023	1529.7707	765.3890	1528.7867	764.8970	14
3	**258.1084**	129.5579			240.0979	120.5526	D	***1475.7601***	738.3837	1458.7336	729.8704	1457.7496	729.3784	13
4	**405.1769**	203.0921			387.1663	194.0868	F	***1360.7332***	680.8702	1343.7066	672.3570	1342.7226	671.8649	12
5	518.2609	259.6341			500.2504	250.6288	I	***1213.6648***	607.3360	1196.6382	598.8227	1195.6542	598.3307	11
6	633.2879	317.1476			615.2773	308.1423	D	***1100.5807***	550.7940	1083.5541	542.2807	1082.5701	541.7887	10
7	761.3464	381.1769	744.3199	372.6636	743.3359	372.1716	Q	***985.5538***	493.2805	968.5272	484.7672			9
8	832.3836	416.6954	815.3570	408.1821	814.3730	407.6901	A	857.4952	429.2512	840.4686	420.7380			8
9	945.4676	473.2375	928.4411	464.7242	927.4571	464.2322	L	***786.4581***	393.7327	769.4315	385.2194			7
10	1016.5047	508.7560	999.4782	500.2427	998.4942	499.7507	A	***673.3740***	337.1906	656.3474	328.6774			6
11	1144.5633	572.7853	1127.5368	564.2720	1126.5528	563.7800	Q	***602.3369***	301.6721	585.3103	293.1588			5
12	1272.6583	636.8328	1255.6317	628.3195	1254.6477	627.8275	K	***474.2783***	237.6428	457.2518	229.1295			4
13	1386.7012	693.8542	1369.6747	685.3410	1368.6906	684.8490	N	***346.1833***	173.5953	329.1568	165.0820			3
14	1443.7227	722.3650	1426.6961	713.8517	1425.7121	713.3597	G	232.1404	116.5738	215.1139	108.0606			2
15							R	175.1190	88.0631	158.0924	79.5498			1

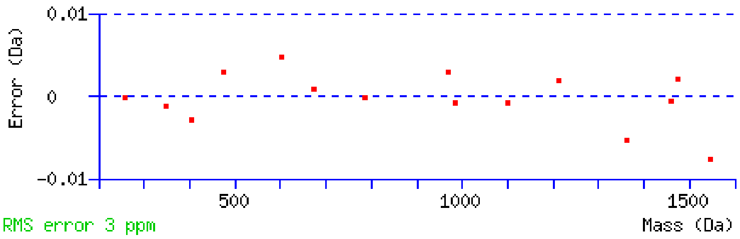

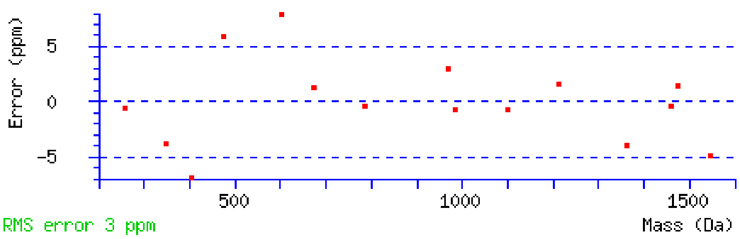

NCBI **BLAST** search of AADFIDQALAQKNGR
(Parameters: blastp, nr protein database, expect=20000, no filter, PAM30)
Other BLAST web gateways

All matches to this query

Score	Mr(calc)	Delta	Sequence
82.4	1616.8270	0.0003	AADFIDQALAQKNGR

Mascot: http://www.matrixscience.com/

Mascot Search Results

Peptide View

MS/MS Fragmentation of **AVETAFNGR**
Found in **Q9Y6C2** in **uni_human_nr**, EMIL1_HUMAN EMILIN-1 OS=Homo sapiens GN=EMILIN1 PE=1 SV=2

Match to Query 301: 964.461768 from(483.238160,2+) intensity(177464.6875) rtinseconds(1218) scans(5255) index(3175)
Title: 130801_HPL_Human_Plaque_BR2_TR1_09_Spectrum023987_scans__5255_RTINSECONDS=1218
Data file E:\\RAW\\HumanPlaques\\mgf\\T\\1D\\T130801_HPL_Human_Plaque_BR2_TR1_09.mgf

Click mouse within plot area to zoom in by factor of two about that point
Or, Plot from 50 to 800 Da Full range
Label all possible matches ○ Label matches used for scoring ⦿
Show Y-axis ☐

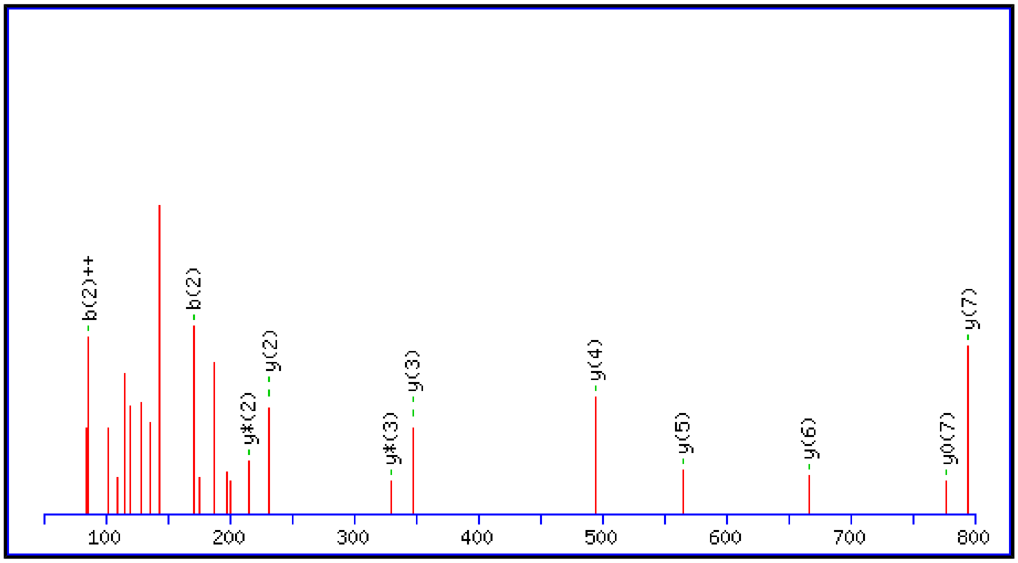

Monoisotopic mass of neutral peptide Mr(calc): 964.4614
Fixed modifications: Carbamidomethyl (C) (apply to specified residues or termini only)
Variable modifications:
N7 : Deamidated (NQ)
Ions Score: 56 **Expect:** 0.00013
Matches : 11/70 fragment ions using 13 most intense peaks (help)

#	b	b^{++}	b*	b^{*++}	b^0	b^{0++}	Seq.	y	y^{++}	y*	y^{*++}	y^0	y^{0++}	#
1	72.0444	36.5258					A							9
2	**171.1128**	**86.0600**					V	894.4316	447.7194	877.4050	439.2062	876.4210	438.7141	8
3	300.1554	150.5813			282.1448	141.5761	E	***795.3632***	398.1852	778.3366	389.6719	777.3526	389.1799	7
4	401.2031	201.1052			383.1925	192.0999	T	***666.3206***	333.6639	649.2940	325.1506	648.3100	324.6586	6
5	472.2402	236.6237			454.2296	227.6185	A	***565.2729***	283.1401	548.2463	274.6268			5
6	619.3086	310.1579			601.2980	301.1527	F	***494.2358***	247.6215	477.2092	239.1082			4
7	734.3355	367.6714	717.3090	359.1581	716.3250	358.6661	N	***347.1674***	174.0873	330.1408	165.5740			3
8	791.3570	396.1821	774.3305	387.6689	773.3464	387.1769	G	***232.1404***	116.5738	215.1139	108.0606			2
9							R	175.1190	88.0631	158.0924	79.5498			1

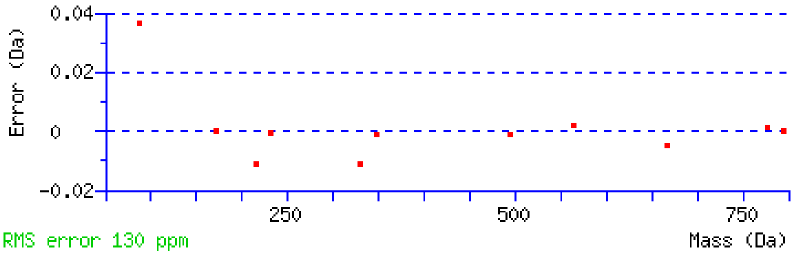

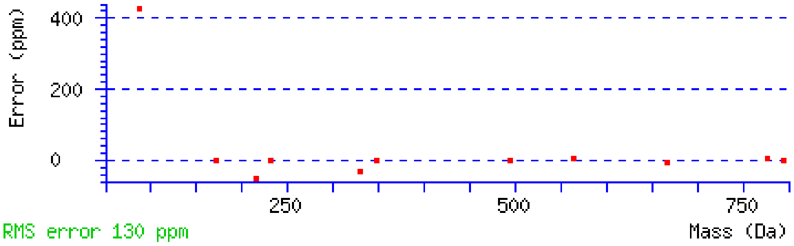

NCBI **BLAST** search of AVETAFNGR
(Parameters: blastp, nr protein database, expect=20000, no filter, PAM30)
Other BLAST web gateways

All matches to this query

Score	Mr(calc)	Delta	Sequence
56.2	964.4614	0.0004	AVETAFNGR
6.8	964.4614	0.0004	GLQEAQYR
4.6	964.4648	-0.0030	AVLQSMSGR
1.1	964.4648	-0.0030	TLNNVTMR
1.1	962.4556	2.0061	TLNQSSSPK
0.7	963.4596	1.0021	TLGHYMAR

Mascot: http://www.matrixscience.com/

Mascot Search Results

Peptide View

MS/MS Fragmentation of **NCELDVTCNIKNGR**
Found in **P00740** in **uni_human_nr**, FA9_HUMAN Coagulation factor IX OS=Homo sapiens GN=F9 PE=1 SV=2

Match to Query 4940: 1692.755082 from(565.258970,3+) intensity(1006666.8125) rtinseconds(1414) scans(6733) index(4487)
Title: 130806_HPL_Human_Plaque_BR2_TR2_10_Spectrum025408_scans__6733_RTINSECONDS=1414
Data file E:\\RAW\\HumanPlaques\\mgf\\T\\1D\\T130806_HPL_Human_Plaque_BR2_TR2_10.mgf

Click mouse within plot area to zoom in by factor of two about that point
Or, [Plot from] [0] to [1600] Da [Full range]
Label all possible matches ○ Label matches used for scoring ⊙
Show Y-axis ☐

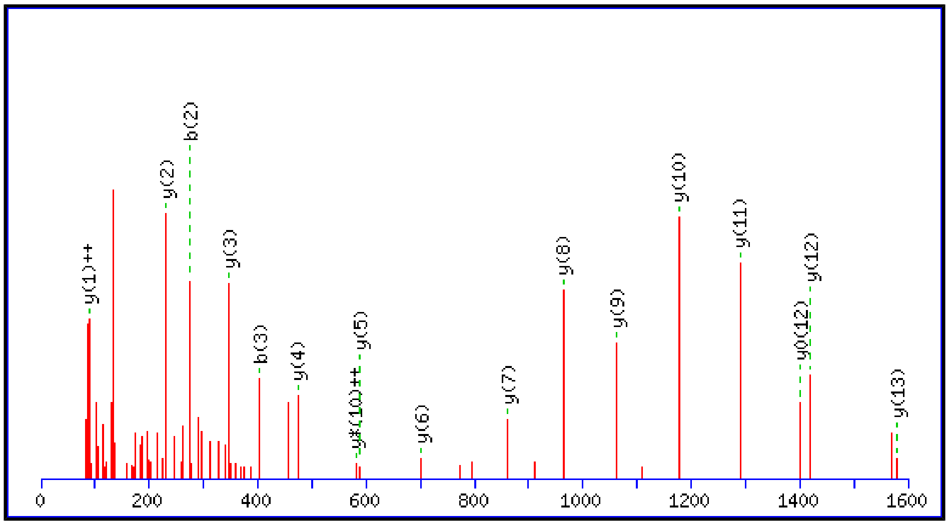

Monoisotopic mass of neutral peptide Mr(calc): 1692.7559
Fixed modifications: Carbamidomethyl (C) (apply to specified residues or termini only)
Variable modifications:
N12 : Deamidated (NQ)
Ions Score: 117 **Expect:** 7.2e-011
Matches : 17/138 fragment ions using 24 most intense peaks (help)

#	b	b^{++}	b*	b^{*++}	b^0	b^{0++}	Seq.	y	y^{++}	y*	y^{*++}	y^0	y^{0++}	#
1	115.0502	58.0287	98.0237	49.5155			N							14
2	**275.0809**	138.0441	258.0543	129.5308			C	***1579.7203***	790.3638	1562.6938	781.8505	1561.7097	781.3585	13
3	**404.1234**	202.5654	387.0969	194.0521	386.1129	193.5601	E	***1419.6897***	710.3485	1402.6631	701.8352	1401.6791	701.3432	12
4	517.2075	259.1074	500.1810	250.5941	499.1969	250.1021	L	***1290.6471***	645.8272	1273.6205	637.3139	1272.6365	636.8219	11
5	632.2345	316.6209	615.2079	308.1076	614.2239	307.6156	D	***1177.5630***	589.2851	1160.5364	580.7719	1159.5524	580.2799	10
6	731.3029	366.1551	714.2763	357.6418	713.2923	357.1498	V	***1062.5361***	531.7717	1045.5095	523.2584	1044.5255	522.7664	9
7	832.3505	416.6789	815.3240	408.1656	814.3400	407.6736	T	***963.4676***	482.2375	946.4411	473.7242	945.4571	473.2322	8
8	992.3812	496.6942	975.3546	488.1810	974.3706	487.6890	C	***862.4200***	431.7136	845.3934	423.2003			7
9	1106.4241	553.7157	1089.3976	545.2024	1088.4136	544.7104	N	***702.3893***	351.6983	685.3628	343.1850			6
10	1219.5082	610.2577	1202.4816	601.7445	1201.4976	601.2524	I	***588.3464***	294.6768	571.3198	286.1636			5
11	1347.6031	674.3052	1330.5766	665.7919	1329.5926	665.2999	K	***475.2623***	238.1348	458.2358	229.6215			4
12	1462.6301	731.8187	1445.6035	723.3054	1444.6195	722.8134	N	***347.1674***	174.0873	330.1408	165.5740			3
13	1519.6516	760.3294	1502.6250	751.8161	1501.6410	751.3241	G	***232.1404***	116.5738	215.1139	108.0606			2
14							R	175.1190	88.0631	158.0924	79.5498			1

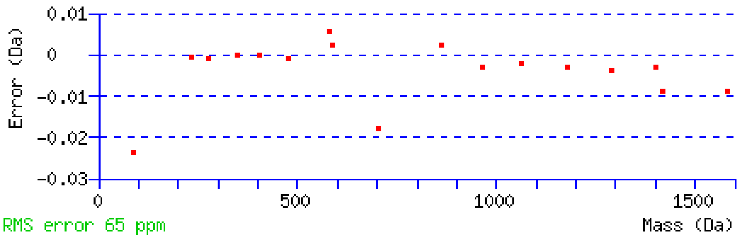

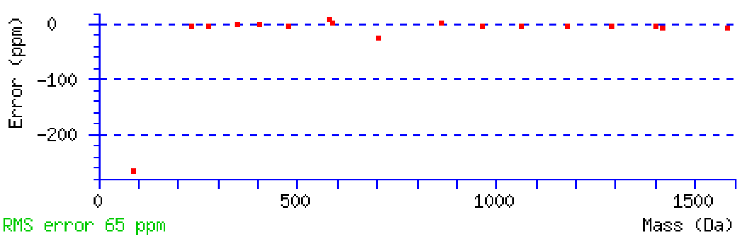

NCBI **BLAST** search of NCELDVTCNIKNGR
(Parameters: blastp, nr protein database, expect=20000, no filter, PAM30)
Other BLAST web gateways

All matches to this query

Score	Mr(calc)	Delta	Sequence	Site Analysis
117.3	1692.7559	-0.0009	NCELDVTCNIKNGR	Deamidated N12 100.00%
68.6	1692.7559	-0.0009	NCELDVTCNIKNGR	Deamidated N9 0.00%
10.8	1690.7468	2.0083	AEEGQDVCLEVETGR	

Mascot: http://www.matrixscience.com/

Mascot Search Results

Peptide View

MS/MS Fragmentation of **NCELDVTCNIKNGRCEQFCK**
Found in **P00740** in **uni_human_nr**, FA9_HUMAN Coagulation factor IX OS=Homo sapiens GN=F9 PE=1 SV=2

Match to Query 12189: 2544.109812 from(849.043880,3+) intensity(37455.6328) rtinseconds(910) scans(3481) index(1710)
Title: 130806_HPL_Human_Plaque_BR1_TR2_18_Spectrum021858_scans__3481_RTINSECONDS=910
Data file E:\\RAW\\HumanPlaques\\mgf\\T\\1D\\T130806_HPL_Human_Plaque_BR1_TR2_18.mgf

Click mouse within plot area to zoom in by factor of two about that point
Or, Plot from 0 to 1400 Da Full range
Label all possible matches ○ Label matches used for scoring ⊙
Show Y-axis ☐

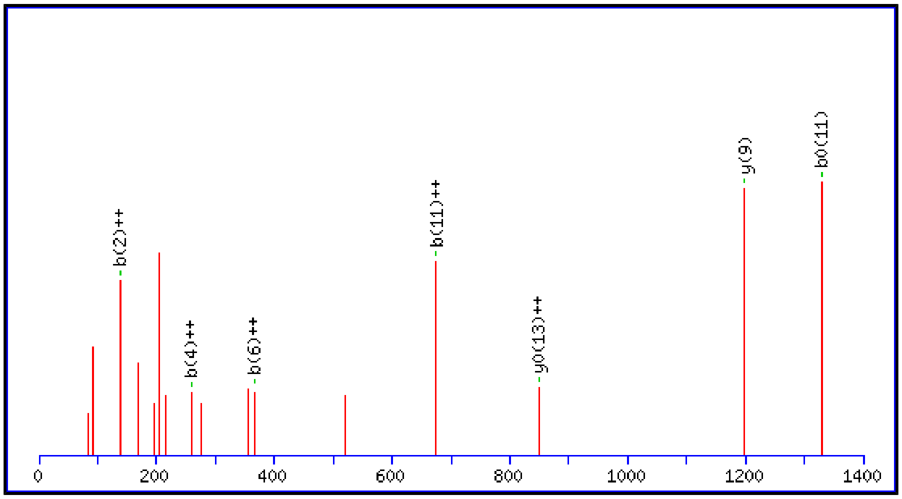

Monoisotopic mass of neutral peptide Mr(calc): 2544.0978
Fixed modifications: Carbamidomethyl (C) (apply to specified residues or termini only)
Ions Score: 18 **Expect:** 0.54
Matches : 7/216 fragment ions using 11 most intense peaks (help)

#	b	b^{++}	b*	b^{*++}	b^{0}	b^{0++}	Seq.	y	y^{++}	y*	y^{*++}	y^{0}	y^{0++}	#
1	115.0502	58.0287	98.0237	49.5155			N							20
2	275.0809	***138.0441***	258.0543	129.5308			C	2431.0621	1216.0347	2414.0356	1207.5214	2413.0516	1207.0294	19
3	404.1234	202.5654	387.0969	194.0521	386.1129	193.5601	E	2271.0315	1136.0194	2254.0049	1127.5061	2253.0209	1127.0141	18
4	517.2075	***259.1074***	500.1810	250.5941	499.1969	250.1021	L	2141.9889	1071.4981	2124.9623	1062.9848	2123.9783	1062.4928	17
5	632.2345	316.6209	615.2079	308.1076	614.2239	307.6156	D	2028.9048	1014.9561	2011.8783	1006.4428	2010.8943	1005.9508	16
6	731.3029	***366.1551***	714.2763	357.6418	713.2923	357.1498	V	1913.8779	957.4426	1896.8513	948.9293	1895.8673	948.4373	15
7	832.3505	416.6789	815.3240	408.1656	814.3400	407.6736	T	1814.8095	907.9084	1797.7829	899.3951	1796.7989	898.9031	14
8	992.3812	496.6942	975.3546	488.1810	974.3706	487.6890	C	1713.7618	857.3845	1696.7352	848.8713	1695.7512	848.3793	13
9	1106.4241	553.7157	1089.3976	545.2024	1088.4136	544.7104	N	1553.7311	777.3692	1536.7046	768.8559	1535.7206	768.3639	12
10	1219.5082	610.2577	1202.4816	601.7445	1201.4976	601.2524	I	1439.6882	720.3477	1422.6617	711.8345	1421.6777	711.3425	11
11	1347.6031	***674.3052***	1330.5766	665.7919	1329.5926	665.2999	K	1326.6042	663.8057	1309.5776	655.2924	1308.5936	654.8004	10
12	1461.6461	731.3267	1444.6195	722.8134	1443.6355	722.3214	N	**1198.5092**	599.7582	1181.4826	591.2450	1180.4986	590.7529	9
13	1518.6675	759.8374	1501.6410	751.3241	1500.6570	750.8321	G	1084.4663	542.7368	1067.4397	534.2235	1066.4557	533.7315	8
14	1674.7686	837.8880	1657.7421	829.3747	1656.7581	828.8827	R	1027.4448	514.2260	1010.4182	505.7128	1009.4342	505.2208	7
15	1834.7993	917.9033	1817.7727	909.3900	1816.7887	908.8980	C	871.3437	436.1755	854.3171	427.6622	853.3331	427.1702	6
16	1963.8419	982.4246	1946.8153	973.9113	1945.8313	973.4193	E	711.3130	356.1602	694.2865	347.6469	693.3025	347.1549	5
17	2091.9005	1046.4539	2074.8739	1037.9406	2073.8899	1037.4486	Q	582.2704	291.6389	565.2439	283.1256			4
18	2238.9689	1119.9881	2221.9423	1111.4748	2220.9583	1110.9828	F	454.2119	227.6096	437.1853	219.0963			3
19	2398.9995	1200.0034	2381.9730	1191.4901	2380.9890	1190.9981	C	307.1435	154.0754	290.1169	145.5621			2

**20**							**K**	147.1128	74.0600	130.0863	65.5468		**1**

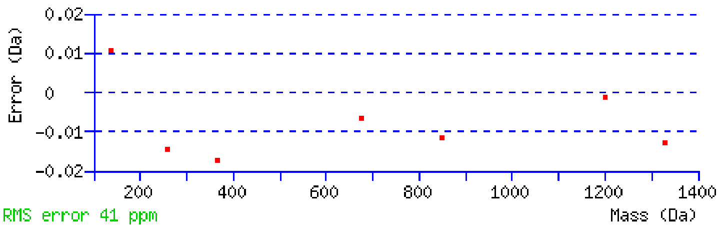

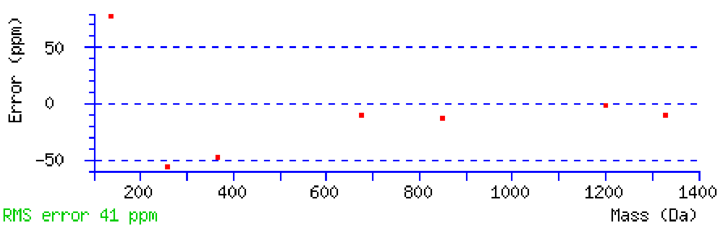

NCBI **BLAST** search of NCELDVTCNIKNGRCEQFCK
(Parameters: blastp, nr protein database, expect=20000, no filter, PAM30)
Other BLAST web gateways

All matches to this query

Score	Mr(calc)	Delta	Sequence
17.6	2544.0978	0.0120	NCELDVTCNIKNGRCEQFCK
0.2	2542.1017	2.0082	EEGTEKIGIWNSNSGLNMTDSNK
0.2	2542.1017	2.0082	EEGTEKIGIWNSNSGLNMTDSNK
0.2	2542.1017	2.0082	EEGTEKIGIWNSNSGLNMTDSNK

Mascot: http://www.matrixscience.com/

Mascot Search Results

Peptide View

MS/MS Fragmentation of **DIDECLQNGR**
Found in **P35555** in **uni_human_nr**, FBN1_HUMAN Fibrillin-1 OS=Homo sapiens GN=FBN1 PE=1 SV=3

Match to Query 1623: 1219.513768 from(610.764160,2+) intensity(1292732.0000) rtinseconds(1416) scans(6546) index(4319)
Title: 130801_HPL_Human_Plaque_BR2_TR1_20_Spectrum023221_scans__6546_RTINSECONDS=1416
Data file E:\\RAW\\HumanPlaques\\mgf\\T\\1D\\T130801_HPL_Human_Plaque_BR2_TR1_20.mgf

Click mouse within plot area to zoom in by factor of two about that point
Or, Plot from 50 to 1000 Da Full range
Label all possible matches ○ Label matches used for scoring ⊙
Show Y-axis ☐

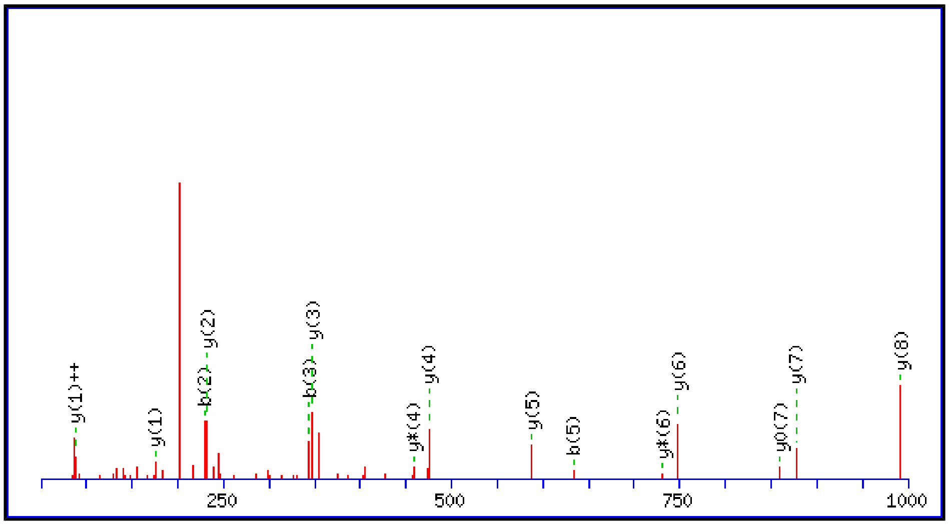

Monoisotopic mass of neutral peptide Mr(calc): 1219.5139
Fixed modifications: Carbamidomethyl (C) (apply to specified residues or termini only)
Variable modifications:
N8 : Deamidated (NQ)
Ions Score: 73 **Expect:** 4.7e-007
Matches : 15/84 fragment ions using 19 most intense peaks (help)

#	b	b++	b*	b*++	b0	b0++	Seq.	y	y++	y*	y*++	y0	y0++	#
1	116.0342	58.5207			98.0237	49.5155	D							10
2	**229.1183**	115.0628			211.1077	106.0575	I	1105.4943	553.2508	1088.4677	544.7375	1087.4837	544.2455	9
3	**344.1452**	172.5763			326.1347	163.5710	D	***992.4102***	496.7087	975.3836	488.1955	974.3996	487.7034	8
4	473.1878	237.0975			455.1773	228.0923	E	***877.3832***	439.1953	860.3567	430.6820	859.3727	430.1900	7
5	**633.2185**	317.1129			615.2079	308.1076	C	***748.3407***	374.6740	731.3141	366.1607			6
6	746.3025	373.6549			728.2920	364.6496	L	***588.3100***	294.6586	571.2835	286.1454			5
7	874.3611	437.6842	857.3346	429.1709	856.3505	428.6789	Q	***475.2259***	238.1166	458.1994	229.6033			4
8	989.3881	495.1977	972.3615	486.6844	971.3775	486.1924	N	***347.1674***	174.0873	330.1408	165.5740			3
9	1046.4095	523.7084	1029.3830	515.1951	1028.3990	514.7031	G	***232.1404***	116.5738	215.1139	108.0606			2
10							R	***175.1190***	88.0631	158.0924	79.5498			1

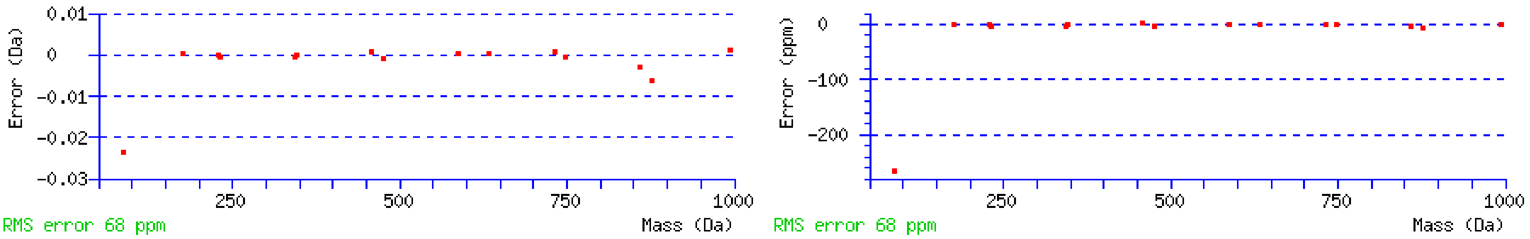

NCBI **BLAST** search of DIDECLQNGR
(Parameters: blastp, nr protein database, expect=20000, no filter, PAM30)
Other BLAST web gateways

All matches to this query

Score	Mr(calc)	Delta	Sequence	Site Analysis
73.3	1219.5139	-0.0001	DIDECLQNGR	Deamidated N8 97.72%
57.0	1219.5139	-0.0001	DIDECLQNGR	Deamidated Q7 2.28%
3.7	1219.5148	-0.0010	ENCMMHLLR	
3.7	1219.5148	-0.0010	ENCMMHLLR	

Mascot: http://www.matrixscience.com/

Mascot Search Results

Peptide View

MS/MS Fragmentation of **DIDECLQNGR**
Found in **P35555** in **uni_human_nr**, FBN1_HUMAN Fibrillin-1 OS=Homo sapiens GN=FBN1 PE=1 SV=3

Match to Query 1451: 1218.530008 from(610.272280,2+) intensity(497201.5938) rtinseconds(1339) scans(6100) index(3836)
Title: 130801_HPL_Human_Plaque_BR1_TR1_17_Spectrum024000_scans__6100_RTINSECONDS=1339
Data file E:\\RAW\\HumanPlaques\\mgf\\T\\1D\\T130801_HPL_Human_Plaque_BR1_TR1_17.mgf

Click mouse within plot area to zoom in by factor of two about that point
Or, [Plot from] [50] to [1000] Da [Full range]
Label all possible matches ○ Label matches used for scoring ⊙
Show Y-axis ☐

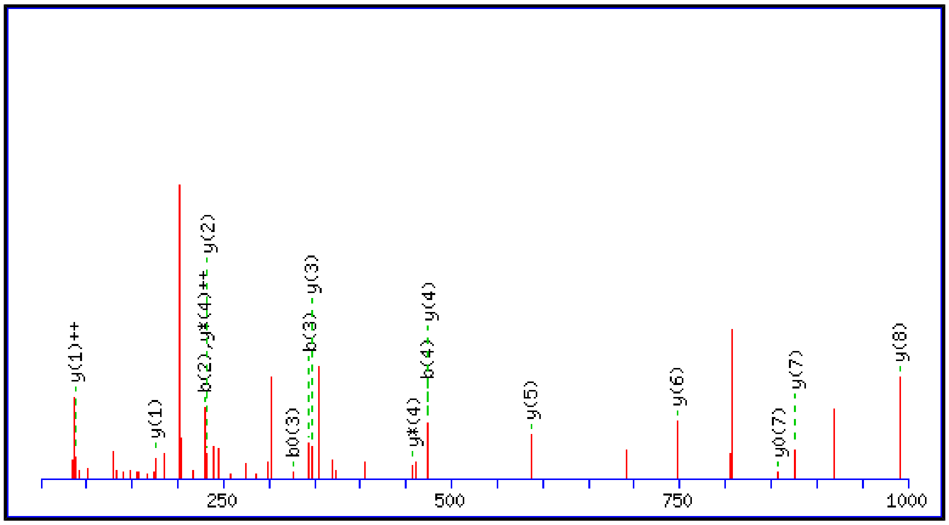

Monoisotopic mass of neutral peptide Mr(calc): 1218.5299
Fixed modifications: Carbamidomethyl (C) (apply to specified residues or termini only)
Ions Score: 42 **Expect:** 0.00085
Matches : 16/84 fragment ions using 40 most intense peaks (help)

#	b	b^{++}	b^{*}	b^{*++}	b^{0}	b^{0++}	Seq.	y	y^{++}	y^{*}	y^{*++}	y^{0}	y^{0++}	#
1	116.0342	58.5207			98.0237	49.5155	D							10
2	**229.1183**	115.0628			211.1077	106.0575	I	1104.5102	552.7588	1087.4837	544.2455	1086.4997	543.7535	9
3	**344.1452**	172.5763			326.1347	163.5710	D	***991.4262***	496.2167	974.3996	487.7034	973.4156	487.2114	8
4	**473.1878**	237.0975			455.1773	228.0923	E	***876.3992***	438.7033	859.3727	430.1900	858.3887	429.6980	7
5	633.2185	317.1129			615.2079	308.1076	C	***747.3566***	374.1820	730.3301	365.6687			6
6	746.3025	373.6549			728.2920	364.6496	L	***587.3260***	294.1666	570.2994	285.6534			5
7	874.3611	437.6842	857.3346	429.1709	856.3505	428.6789	Q	***474.2419***	237.6246	457.2154	229.1113			4
8	988.4040	494.7057	971.3775	486.1924	970.3935	485.7004	N	***346.1833***	173.5953	329.1568	165.0820			3
9	1045.4255	523.2164	1028.3990	514.7031	1027.4149	514.2111	G	***232.1404***	116.5738	215.1139	108.0606			2
10							R	***175.1190***	88.0631	158.0924	79.5498			1

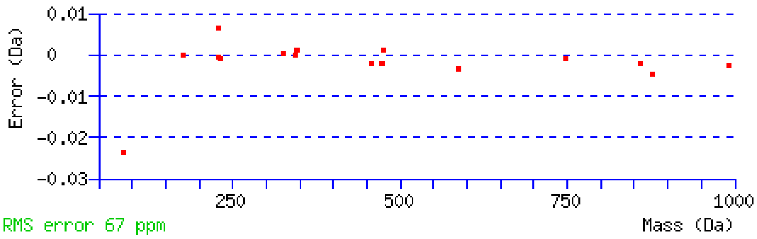

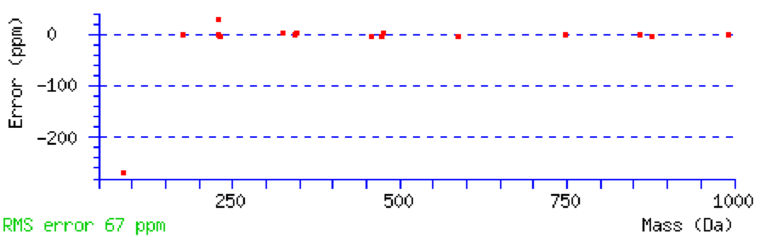

NCBI **BLAST** search of DIDECLQNGR
(Parameters: blastp, nr protein database, expect=20000, no filter, PAM30)
Other BLAST web gateways

All matches to this query

Score	Mr(calc)	Delta	Sequence
42.0	1218.5299	0.0001	DIDECLQNGR

Mascot: http://www.matrixscience.com/

Mascot Search Results

Peptide View

MS/MS Fragmentation of **DIDECLQNGRICNNGR**
Found in **P35555** in **uni_human_nr**, FBN1_HUMAN Fibrillin-1 OS=Homo sapiens GN=FBN1 PE=1 SV=3

Match to Query 7398: 1936.807572 from(646.609800,3+) intensity(123260.1719) rtinseconds(1738) scans(9002) index(6725)
Title: 130806_HPL_Human_Plaque_BR1_TR2_20_Spectrum026593_scans__9002_RTINSECONDS=1738
Data file E:\\RAW\\HumanPlaques\\mgf\\T\\1D\\T130806_HPL_Human_Plaque_BR1_TR2_20.mgf

Click mouse within plot area to zoom in by factor of two about that point
Or, Plot from 0 to 1800 Da Full range
Label all possible matches ○ Label matches used for scoring ⊙
Show Y-axis ☐

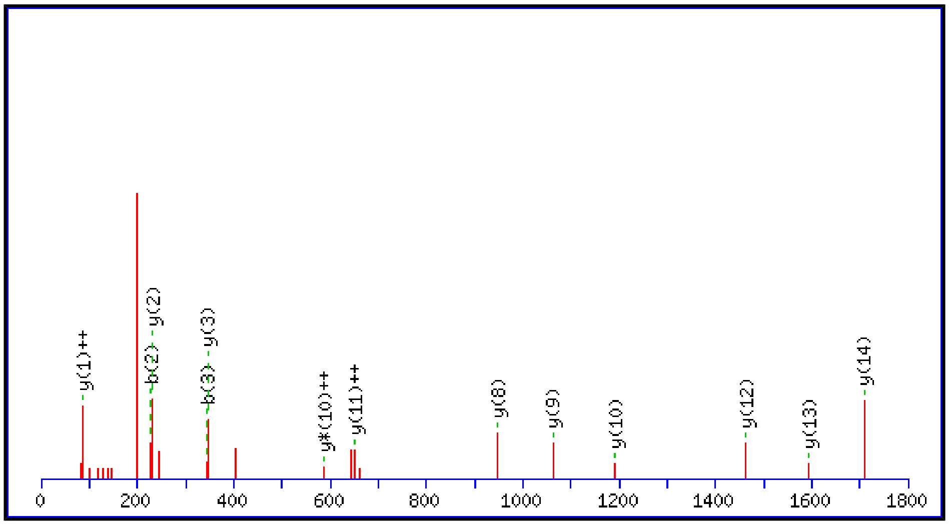

Monoisotopic mass of neutral peptide Mr(calc): 1935.8051
Fixed modifications: Carbamidomethyl (C) (apply to specified residues or termini only)
Variable modifications:
N8 : Deamidated (NQ)
N13 : Deamidated (NQ)
N14 : Deamidated (NQ)
Ions Score: 59 **Expect:** 2e-005
Matches : 13/144 fragment ions using 18 most intense peaks (help)

#	b	b^{++}	b*	b^{*++}	b^0	b^{0++}	Seq.	y	y^{++}	y*	y^{*++}	y^0	y^{0++}	#
1	116.0342	58.5207			98.0237	49.5155	D							16
2	**229.1183**	115.0628			211.1077	106.0575	I	1821.7854	911.3963	1804.7589	902.8831	1803.7749	902.3911	15
3	**344.1452**	172.5763			326.1347	163.5710	D	***1708.7014***	854.8543	1691.6748	846.3410	1690.6908	845.8490	14
4	473.1878	237.0975			455.1773	228.0923	E	***1593.6744***	797.3408	1576.6479	788.8276	1575.6639	788.3356	13
5	633.2185	317.1129			615.2079	308.1076	C	***1464.6318***	732.8196	1447.6053	724.3063			12
6	746.3025	373.6549			728.2920	364.6496	L	1304.6012	652.8042	1287.5746	644.2910			11
7	874.3611	437.6842	857.3346	429.1709	856.3505	428.6789	Q	***1191.5171***	596.2622	1174.4906	587.7489			10
8	989.3881	495.1977	972.3615	486.6844	971.3775	486.1924	N	***1063.4585***	532.2329	1046.4320	523.7196			9
9	1046.4095	523.7084	1029.3830	515.1951	1028.3990	514.7031	G	***948.4316***	474.7194	931.4050	466.2062			8
10	1202.5106	601.7590	1185.4841	593.2457	1184.5001	592.7537	R	891.4101	446.2087	874.3836	437.6954			7
11	1315.5947	658.3010	1298.5681	649.7877	1297.5841	649.2957	I	735.3090	368.1581	718.2825	359.6449			6
12	1475.6253	738.3163	1458.5988	729.8030	1457.6148	729.3110	C	622.2250	311.6161	605.1984	303.1028			5
13	1590.6523	795.8298	1573.6257	787.3165	1572.6417	786.8245	N	462.1943	231.6008	445.1678	223.0875			4
14	1705.6792	853.3433	1688.6527	844.8300	1687.6687	844.3380	N	***347.1674***	174.0873	330.1408	165.5740			3

**15**	1762.7007	881.8540	1745.6741	873.3407	1744.6901	872.8487	**G**	***232.1404***	116.5738	215.1139	108.0606			**2**
**16**							**R**	175.1190	88.0631	158.0924	79.5498			**1**

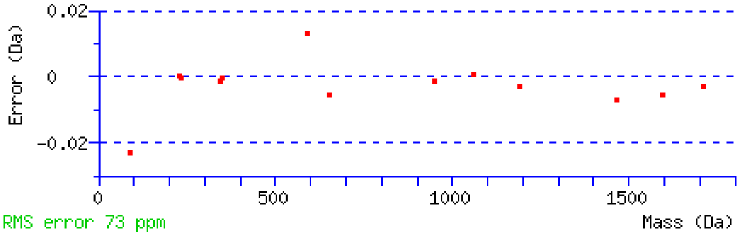

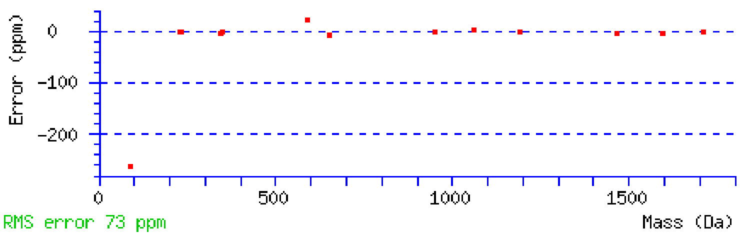

NCBI **BLAST** search of DIDECLQNGRICNNGR
(Parameters: blastp, nr protein database, expect=20000, no filter, PAM30)
Other BLAST web gateways

All matches to this query

Score	Mr(calc)	Delta	Sequence	Site Analysis
58.5	1935.8051	1.0025	DIDECLQNGRICNNGR	Deamidated N8, N13, N14 95.33%
45.1	1935.8051	1.0025	DIDECLQNGRICNNGR	Deamidated Q7, N13, N14 4.38%
33.0	1935.8051	1.0025	DIDECLQNGRICNNGR	Deamidated Q7, N8, N14 0.27%
22.2	1935.8051	1.0025	DIDECLQNGRICNNGR	Deamidated Q7, N8, N13 0.02%
3.4	1935.7970	1.0106	SLDWENPTEREDDSDK	
1.1	1936.8034	0.0041	AEGSSQSWRQQEEEQR	
1.1	1936.8034	0.0041	AEGSSQSWRQQEEEQR	
1.1	1936.8034	0.0041	AEGSSQSWRQQEEEQR	
0.6	1934.7986	2.0090	EAGQGKDMMEEEAPEER	

Mascot: http://www.matrixscience.com/

Mascot Search Results

Peptide View

MS/MS Fragmentation of **DIDECLQNGRICNNGR**
Found in **P35555** in **uni_human_nr**, FBN1_HUMAN Fibrillin-1 OS=Homo sapiens GN=FBN1 PE=1 SV=3

Match to Query 8257: 1934.820162 from(645.947330,3+) intensity(1058828.1250) rtinseconds(1687) scans(8481) index(6125)
Title: 130806_HPL_Human_Plaque_BR2_TR2_20_Spectrum025338_scans__8481_RTINSECONDS=1687
Data file E:\\RAW\\HumanPlaques\\mgf\\T\\1D\\T130806_HPL_Human_Plaque_BR2_TR2_20.mgf

Click mouse within plot area to zoom in by factor of two about that point
Or, Plot from 0 to 1800 Da Full range
Label all possible matches ○ Label matches used for scoring ⊙
Show Y-axis ☐

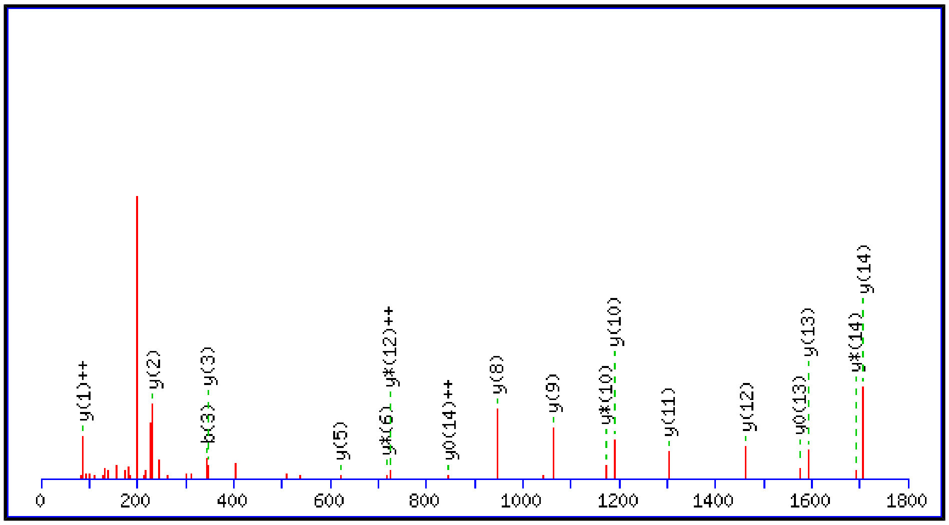

Monoisotopic mass of neutral peptide Mr(calc): 1934.8210
Fixed modifications: Carbamidomethyl (C) (apply to specified residues or termini only)
Variable modifications:
N8 : Deamidated (NQ)
N13 : Deamidated (NQ)
Ions Score: 80 **Expect:** 1.7e-007
Matches : 18/144 fragment ions using 24 most intense peaks (help)

#	b	b^{++}	b*	b^{*++}	b^0	b^{0++}	Seq.	y	y^{++}	y*	y^{*++}	y^0	y^{0++}	#
1	116.0342	58.5207			98.0237	49.5155	D							16
2	229.1183	115.0628			211.1077	106.0575	I	1820.8014	910.9043	1803.7749	902.3911	1802.7908	901.8991	15
3	**344.1452**	172.5763			326.1347	163.5710	D	***1707.7173***	854.3623	1690.6908	845.8490	1689.7068	845.3570	14
4	473.1878	237.0975			455.1773	228.0923	E	***1592.6904***	796.8488	1575.6639	788.3356	1574.6798	787.8436	13
5	633.2185	317.1129			615.2079	308.1076	C	***1463.6478***	732.3275	1446.6213	723.8143			12
6	746.3025	373.6549			728.2920	364.6496	L	***1303.6172***	652.3122	1286.5906	643.7989			11
7	874.3611	437.6842	857.3346	429.1709	856.3505	428.6789	Q	***1190.5331***	595.7702	1173.5065	587.2569			10
8	989.3881	495.1977	972.3615	486.6844	971.3775	486.1924	N	***1062.4745***	531.7409	1045.4480	523.2276			9
9	1046.4095	523.7084	1029.3830	515.1951	1028.3990	514.7031	G	***947.4476***	474.2274	930.4210	465.7142			8
10	1202.5106	601.7590	1185.4841	593.2457	1184.5001	592.7537	R	890.4261	445.7167	873.3996	437.2034			7
11	1315.5947	658.3010	1298.5681	649.7877	1297.5841	649.2957	I	734.3250	367.6661	717.2984	359.1529			6
12	1475.6253	738.3163	1458.5988	729.8030	1457.6148	729.3110	C	***621.2409***	311.1241	604.2144	302.6108			5
13	1590.6523	795.8298	1573.6257	787.3165	1572.6417	786.8245	N	461.2103	231.1088	444.1837	222.5955			4
14	1704.6952	852.8512	1687.6687	844.3380	1686.6846	843.8460	N	***346.1833***	173.5953	329.1568	165.0820			3
15	1761.7167	881.3620	1744.6901	872.8487	1743.7061	872.3567	G	***232.1404***	116.5738	215.1139	108.0606			2

**16**							**R**	175.1190	88.0631	158.0924	79.5498		**1**

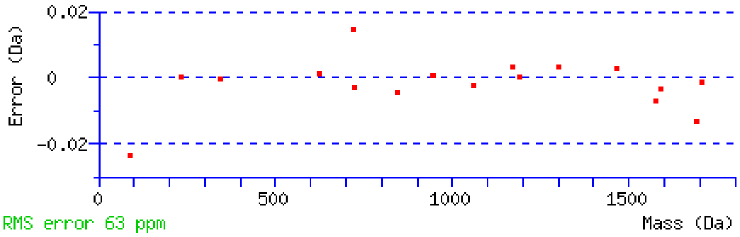

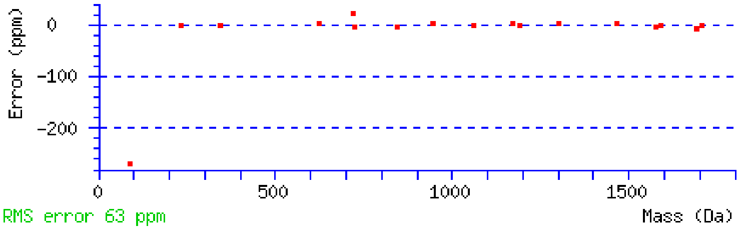

NCBI **BLAST** search of DIDECLQNGRICNNGR
(Parameters: blastp, nr protein database, expect=20000, no filter, PAM30)
Other BLAST web gateways

All matches to this query

Score	Mr(calc)	Delta	Sequence	Site Analysis
80.0	1934.8210	-0.0009	DIDECLQNGRICNNGR	Deamidated N8, N13 90.44%
68.1	1934.8210	-0.0009	DIDECLQNGRICNNGR	Deamidated Q7, N13 5.89%
65.7	1934.8210	-0.0009	DIDECLQNGRICNNGR	Deamidated N8, N14 3.39%
54.6	1934.8210	-0.0009	DIDECLQNGRICNNGR	Deamidated Q7, N14 0.27%
39.9	1934.8210	-0.0009	DIDECLQNGRICNNGR	Deamidated Q7, N8 0.01%
38.3	1934.8210	-0.0009	DIDECLQNGRICNNGR	Deamidated N13, N14 0.01%
0.6	1933.8087	1.0115	RENMYYSQSSMFPHR	

**Mascot:** http://www.matrixscience.com/

Mascot Search Results

Peptide View

MS/MS Fragmentation of **DIDECLQNGRICNNGR**
Found in **P35555** in **uni_human_nr**, FBN1_HUMAN Fibrillin-1 OS=Homo sapiens GN=FBN1 PE=1 SV=3

Match to Query 7548: 1934.820882 from(645.947570,3+) intensity(143663.5938) rtinseconds(1660) scans(8255) index(5772)
Title: 130801_HPL_Human_Plaque_BR1_TR1_18_Spectrum025423_scans__8255_RTINSECONDS=1660
Data file E:\\RAW\\HumanPlaques\\mgf\\T\\1D\\T130801_HPL_Human_Plaque_BR1_TR1_18.mgf

Click mouse within plot area to zoom in by factor of two about that point
Or, [Plot from] [0] to [1800] Da [Full range]
Label all possible matches ○ Label matches used for scoring ⊙
Show Y-axis ☐

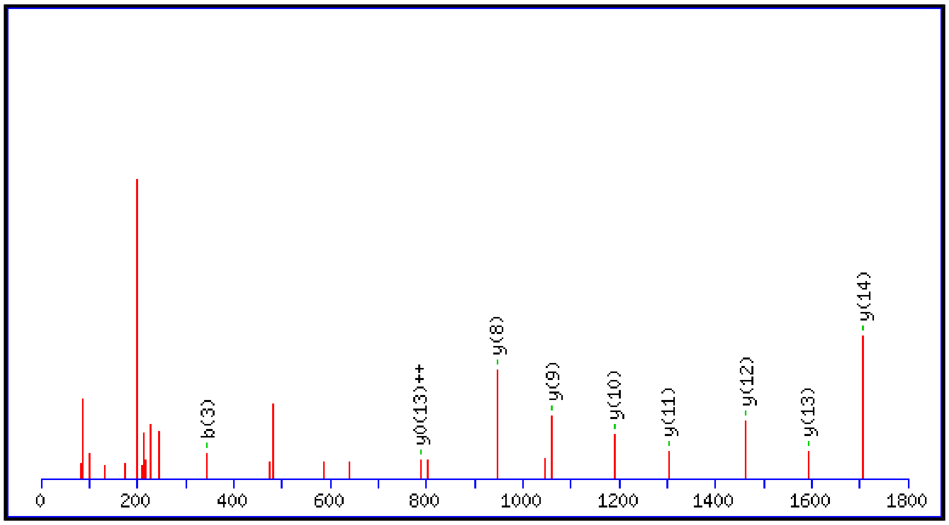

Monoisotopic mass of neutral peptide Mr(calc): 1934.8210
Fixed modifications: Carbamidomethyl (C) (apply to specified residues or termini only)
Variable modifications:
Q7 : Deamidated (NQ)
N8 : Deamidated (NQ)
Ions Score: 48 **Expect:** 0.00025
Matches : 9/144 fragment ions using 13 most intense peaks (help)

#	b	b^{++}	b^{*}	b^{*++}	b^{0}	b^{0++}	Seq.	y	y^{++}	y^{*}	y^{*++}	y^{0}	y^{0++}	#
1	116.0342	58.5207			98.0237	49.5155	D							16
2	229.1183	115.0628			211.1077	106.0575	I	1820.8014	910.9043	1803.7749	902.3911	1802.7908	901.8991	15
3	344.1452	172.5763			326.1347	163.5710	D	***1707.7173***	854.3623	1690.6908	845.8490	1689.7068	845.3570	14
4	473.1878	237.0975			455.1773	228.0923	E	***1592.6904***	796.8488	1575.6639	788.3356	1574.6798	787.8436	13
5	633.2185	317.1129			615.2079	308.1076	C	***1463.6478***	732.3275	1446.6213	723.8143			12
6	746.3025	373.6549			728.2920	364.6496	L	***1303.6172***	652.3122	1286.5906	643.7989			11
7	875.3451	438.1762	858.3186	429.6629	857.3346	429.1709	Q	***1190.5331***	595.7702	1173.5065	587.2569			10
8	990.3721	495.6897	973.3455	487.1764	972.3615	486.6844	N	***1061.4905***	531.2489	1044.4640	522.7356			9
9	1047.3935	524.2004	1030.3670	515.6871	1029.3830	515.1951	G	***946.4636***	473.7354	929.4370	465.2221			8
10	1203.4946	602.2510	1186.4681	593.7377	1185.4841	593.2457	R	889.4421	445.2247	872.4155	436.7114			7
11	1316.5787	658.7930	1299.5522	650.2797	1298.5681	649.7877	I	733.3410	367.1741	716.3144	358.6609			6
12	1476.6094	738.8083	1459.5828	730.2950	1458.5988	729.8030	C	620.2569	310.6321	603.2304	302.1188			5
13	1590.6523	795.8298	1573.6257	787.3165	1572.6417	786.8245	N	460.2263	230.6168	443.1997	222.1035			4
14	1704.6952	852.8512	1687.6687	844.3380	1686.6846	843.8460	N	346.1833	173.5953	329.1568	165.0820			3
15	1761.7167	881.3620	1744.6901	872.8487	1743.7061	872.3567	G	232.1404	116.5738	215.1139	108.0606			2

**16**							**R**	175.1190	88.0631	158.0924	79.5498			**1**

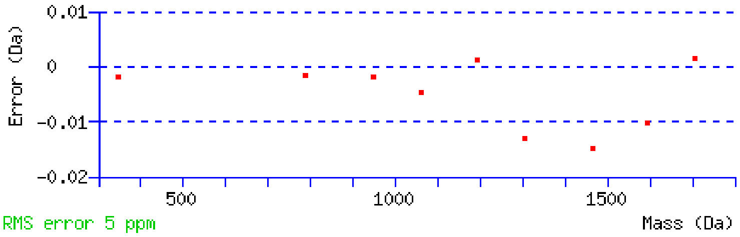

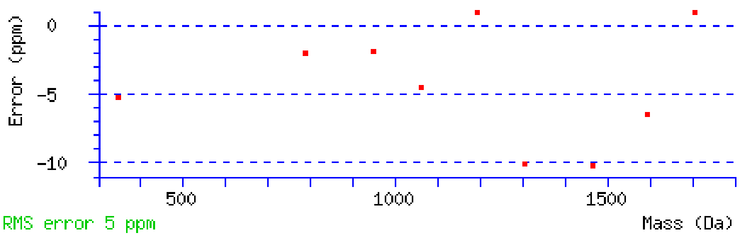

NCBI **BLAST** search of DIDECLQNGRICNNGR
(Parameters: blastp, nr protein database, expect=20000, no filter, PAM30)
Other BLAST web gateways

All matches to this query

Score	Mr(calc)	Delta	Sequence	Site Analysis
48.5	1934.8210	-0.0002	DIDECLQNGRICNNGR	Deamidated Q7, N8 91.64%
34.6	1934.8210	-0.0002	DIDECLQNGRICNNGR	Deamidated Q7, N14 3.80%
34.6	1934.8210	-0.0002	DIDECLQNGRICNNGR	Deamidated Q7, N13 3.80%
22.8	1934.8210	-0.0002	DIDECLQNGRICNNGR	Deamidated N13, N14 0.25%
22.8	1934.8210	-0.0002	DIDECLQNGRICNNGR	Deamidated N8, N14 0.25%
22.8	1934.8210	-0.0002	DIDECLQNGRICNNGR	Deamidated N8, N13 0.25%

**Mascot:** http://www.matrixscience.com/

Mascot Search Results

Peptide View

MS/MS Fragmentation of **DIDECLQNGRICNNGR**
Found in **P35555** in **uni_human_nr**, FBN1_HUMAN Fibrillin-1 OS=Homo sapiens GN=FBN1 PE=1 SV=3

Match to Query 7751: 1933.836522 from(645.619450,3+) intensity(19200392.0000) rtinseconds(1601) scans(8005) index(5523)
Title: 130809_HPL_Human_Plaque_BR2_TR3_17_Spectrum025520_scans__8005_RTINSECONDS=1601
Data file E:\\RAW\\HumanPlaques\\mgf\\T\\1D\\T130809_HPL_Human_Plaque_BR2_TR3_17.mgf

Click mouse within plot area to zoom in by factor of two about that point
Or, Plot from 0 to 1900 Da Full range
Label all possible matches ○ Label matches used for scoring ⦿
Show Y-axis ☐

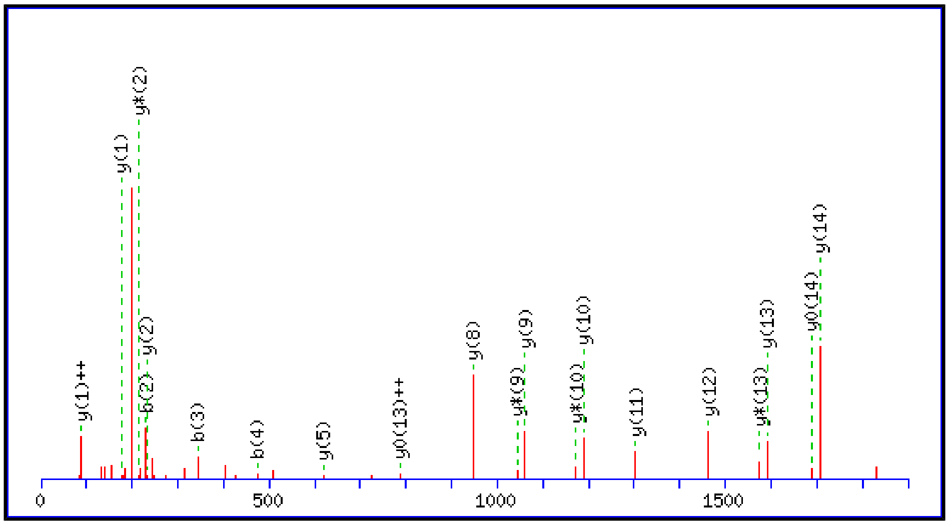

Monoisotopic mass of neutral peptide Mr(calc): 1933.8370
Fixed modifications: Carbamidomethyl (C) (apply to specified residues or termini only)
Variable modifications:
N8 : Deamidated (NQ)
Ions Score: 60 **Expect:** 2.4e-005
Matches : 20/144 fragment ions using 38 most intense peaks (help)

#	b	b^{++}	b*	b^{*++}	b^0	b^{0++}	Seq.	y	y^{++}	y*	y^{*++}	y^0	y^{0++}	#
1	116.0342	58.5207			98.0237	49.5155	D							16
2	**229.1183**	115.0628			211.1077	106.0575	I	1819.8174	910.4123	1802.7908	901.8991	1801.8068	901.4071	15
3	**344.1452**	172.5763			326.1347	163.5710	D	***1706.7333***	853.8703	1689.7068	845.3570	**1688.7228**	844.8650	14
4	**473.1878**	237.0975			455.1773	228.0923	E	***1591.7064***	796.3568	**1574.6798**	787.8436	1573.6958	**787.3515**	13
5	633.2185	317.1129			615.2079	308.1076	C	***1462.6638***	731.8355	1445.6372	723.3223			12
6	746.3025	373.6549			728.2920	364.6496	L	***1302.6331***	651.8202	1285.6066	643.3069			11
7	874.3611	437.6842	857.3346	429.1709	856.3505	428.6789	Q	***1189.5491***	595.2782	**1172.5225**	586.7649			10
8	989.3881	495.1977	972.3615	486.6844	971.3775	486.1924	N	***1061.4905***	531.2489	**1044.4640**	522.7356			9
9	1046.4095	523.7084	1029.3830	515.1951	1028.3990	514.7031	G	***946.4636***	473.7354	929.4370	465.2221			8
10	1202.5106	601.7590	1185.4841	593.2457	1184.5001	592.7537	R	889.4421	445.2247	872.4155	436.7114			7
11	1315.5947	658.3010	1298.5681	649.7877	1297.5841	649.2957	I	733.3410	367.1741	716.3144	358.6609			6
12	1475.6253	738.3163	1458.5988	729.8030	1457.6148	729.3110	C	***620.2569***	310.6321	603.2304	302.1188			5
13	1589.6683	795.3378	1572.6417	786.8245	1571.6577	786.3325	N	460.2263	230.6168	443.1997	222.1035			4
14	1703.7112	852.3592	1686.6846	843.8460	1685.7006	843.3540	N	346.1833	173.5953	329.1568	165.0820			3
15	1760.7327	880.8700	1743.7061	872.3567	1742.7221	871.8647	G	***232.1404***	116.5738	**215.1139**	108.0606			2

**16**							**R**	***175.1190***	88.0631	158.0924	79.5498			**1**

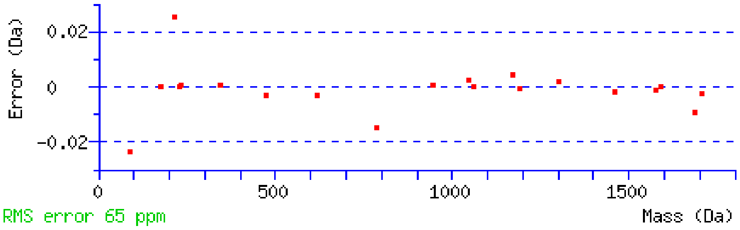

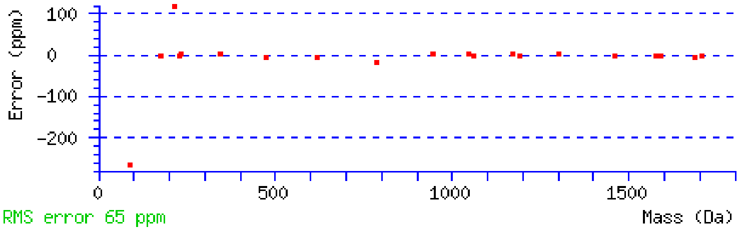

NCBI **BLAST** search of DIDECLQNGRICNNGR
(Parameters: blastp, nr protein database, expect=20000, no filter, PAM30)
Other BLAST web gateways

All matches to this query

Score	Mr(calc)	Delta	Sequence	Site Analysis
60.4	1933.8370	-0.0005	DIDECLQNGRICNNGR	Deamidated N8 92.79%
48.8	1933.8370	-0.0005	DIDECLQNGRICNNGR	Deamidated Q7 6.42%
36.7	1933.8370	-0.0005	DIDECLQNGRICNNGR	Deamidated N14 0.39%
36.7	1933.8370	-0.0005	DIDECLQNGRICNNGR	Deamidated N13 0.39%
1.7	1932.8272	1.0093	VQAMKSPDHNGEDNFAR	
0.4	1932.8247	1.0119	RENMYYSQSSMFPHR	

**Mascot:** http://www.matrixscience.com/

Mascot Search Results

Peptide View

MS/MS Fragmentation of **DIDECLQNGRICNNGR**
Found in **P35555** in **uni_human_nr**, FBN1_HUMAN Fibrillin-1 OS=Homo sapiens GN=FBN1 PE=1 SV=3

Match to Query 7270: 1933.835668 from(967.925110,2+) intensity(3232391.5000) rtinseconds(1595) scans(8053) index(5691)
Title: 130806_HPL_Human_Plaque_BR1_TR2_17_Spectrum026177_scans__8053_RTINSECONDS=1595
Data file E:\\RAW\\HumanPlaques\\mgf\\T\\1D\\T130806_HPL_Human_Plaque_BR1_TR2_17.mgf

Click mouse within plot area to zoom in by factor of two about that point
Or, Plot from 0 to 2000 Da Full range
Label all possible matches ○ Label matches used for scoring ⊙
Show Y-axis ☐

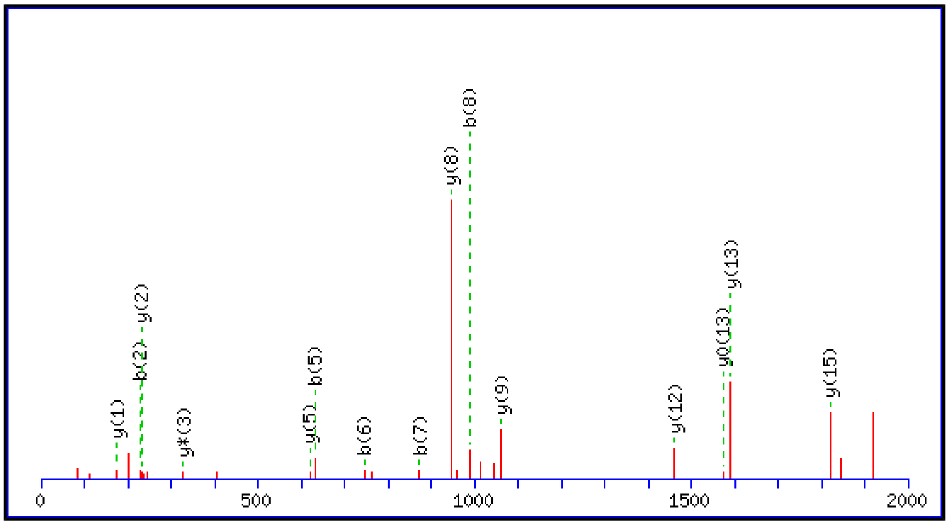

Monoisotopic mass of neutral peptide Mr(calc): 1933.8370
Fixed modifications: Carbamidomethyl (C) (apply to specified residues or termini only)
Variable modifications:
N8 : Deamidated (NQ)
Ions Score: 58 **Expect:** 3.7e-005
Matches : 15/144 fragment ions using 27 most intense peaks (help)

#	b	b^{++}	b*	b^{*++}	b^{0}	b^{0++}	Seq.	y	y^{++}	y*	y^{*++}	y^{0}	y^{0++}	#
1	116.0342	58.5207			98.0237	49.5155	D							16
2	***229.1183***	115.0628			211.1077	106.0575	I	***1819.8174***	910.4123	1802.7908	901.8991	1801.8068	901.4071	15
3	344.1452	172.5763			326.1347	163.5710	D	1706.7333	853.8703	1689.7068	845.3570	1688.7228	844.8650	14
4	473.1878	237.0975			455.1773	228.0923	E	***1591.7064***	796.3568	1574.6798	787.8436	1573.6958	787.3515	13
5	***633.2185***	317.1129			615.2079	308.1076	C	***1462.6638***	731.8355	1445.6372	723.3223			12
6	***746.3025***	373.6549			728.2920	364.6496	L	1302.6331	651.8202	1285.6066	643.3069			11
7	***874.3611***	437.6842	857.3346	429.1709	856.3505	428.6789	Q	1189.5491	595.2782	1172.5225	586.7649			10
8	***989.3881***	495.1977	972.3615	486.6844	971.3775	486.1924	N	***1061.4905***	531.2489	1044.4640	522.7356			9
9	1046.4095	523.7084	1029.3830	515.1951	1028.3990	514.7031	G	***946.4636***	473.7354	929.4370	465.2221			8
10	1202.5106	601.7590	1185.4841	593.2457	1184.5001	592.7537	R	889.4421	445.2247	872.4155	436.7114			7
11	1315.5947	658.3010	1298.5681	649.7877	1297.5841	649.2957	I	733.3410	367.1741	716.3144	358.6609			6
12	1475.6253	738.3163	1458.5988	729.8030	1457.6148	729.3110	C	***620.2569***	310.6321	603.2304	302.1188			5
13	1589.6683	795.3378	1572.6417	786.8245	1571.6577	786.3325	N	460.2263	230.6168	443.1997	222.1035			4
14	1703.7112	852.3592	1686.6846	843.8460	1685.7006	843.3540	N	346.1833	173.5953	329.1568	165.0820			3
15	1760.7327	880.8700	1743.7061	872.3567	1742.7221	871.8647	G	***232.1404***	116.5738	215.1139	108.0606			2

**16**							**R**	***175.1190***	88.0631	158.0924	79.5498			**1**

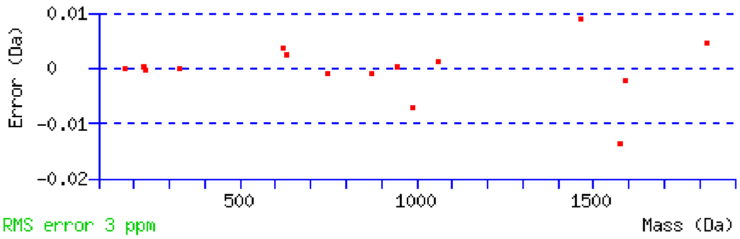

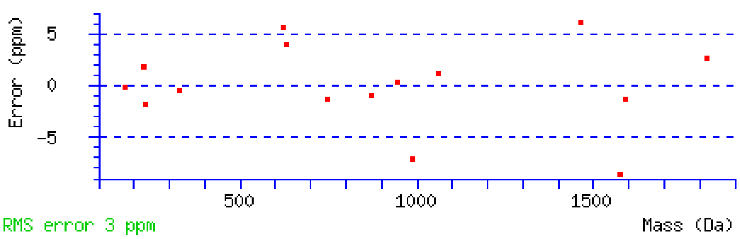

NCBI **BLAST** search of DIDECLQNGRICNNGR
(Parameters: blastp, nr protein database, expect=20000, no filter, PAM30)
Other BLAST web gateways

All matches to this query

Score	Mr(calc)	Delta	Sequence	Site Analysis
58.5	1933.8370	-0.0014	DIDECLQNGRICNNGR	Deamidated N8 96.03%
44.2	1933.8370	-0.0014	DIDECLQNGRICNNGR	Deamidated Q7 3.63%
31.7	1933.8370	-0.0014	DIDECLQNGRICNNGR	Deamidated N13 0.20%
30.0	1933.8370	-0.0014	DIDECLQNGRICNNGR	Deamidated N14 0.14%
0.7	1932.8272	1.0085	VQAMKSPDHNGEDNFAR	
0.4	1933.8402	-0.0045	NDIHLDADDPNSADKHR	

Mascot: http://www.matrixscience.com/

Mascot Search Results

Peptide View

MS/MS Fragmentation of **DIDECLQNGRICNNGR**
Found in **P35555** in **uni_human_nr**, FBN1_HUMAN Fibrillin-1 OS=Homo sapiens GN=FBN1 PE=1 SV=3

Match to Query 8284: 1933.836882 from(645.619570,3+) intensity(205245.7969) rtinseconds(1599) scans(8107) index(5816)
Title: 130806_HPL_Human_Plaque_BR1_TR2_18_Spectrum025964_scans__8107_RTINSECONDS=1599
Data file E:\\RAW\\HumanPlaques\\mgf\\T\\1D\\T130806_HPL_Human_Plaque_BR1_TR2_18.mgf

Click mouse within plot area to zoom in by factor of two about that point
Or, Plot from 0 to 1800 Da Full range
Label all possible matches ○ Label matches used for scoring ⊙
Show Y-axis ☐

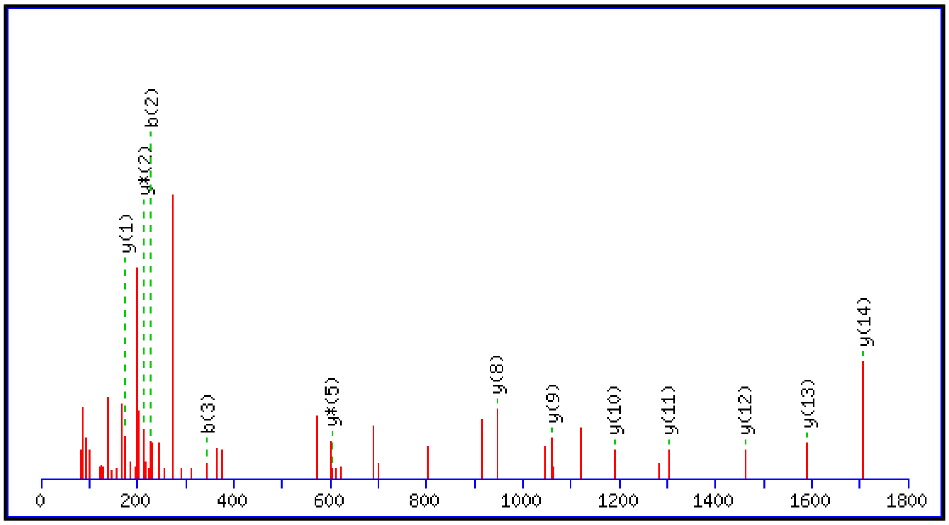

Monoisotopic mass of neutral peptide Mr(calc): 1933.8370
Fixed modifications: Carbamidomethyl (C) (apply to specified residues or termini only)
Variable modifications:
N14 : Deamidated (NQ)
Ions Score: 25 **Expect:** 0.08
Matches : 12/144 fragment ions using 39 most intense peaks (help)

#	b	b^{++}	b*	b^{*++}	b^0	b^{0++}	Seq.	y	y^{++}	y*	y^{*++}	y^0	y^{0++}	#
1	116.0342	58.5207			98.0237	49.5155	D							16
2	**229.1183**	115.0628			211.1077	106.0575	I	1819.8174	910.4123	1802.7908	901.8991	1801.8068	901.4071	15
3	**344.1452**	172.5763			326.1347	163.5710	D	***1706.7333***	853.8703	1689.7068	845.3570	1688.7228	844.8650	14
4	473.1878	237.0975			455.1773	228.0923	E	***1591.7064***	796.3568	1574.6798	787.8436	1573.6958	787.3515	13
5	633.2185	317.1129			615.2079	308.1076	C	***1462.6638***	731.8355	1445.6372	723.3223			12
6	746.3025	373.6549			728.2920	364.6496	L	***1302.6331***	651.8202	1285.6066	643.3069			11
7	874.3611	437.6842	857.3346	429.1709	856.3505	428.6789	Q	***1189.5491***	595.2782	1172.5225	586.7649			10
8	988.4040	494.7057	971.3775	486.1924	970.3935	485.7004	N	***1061.4905***	531.2489	1044.4640	522.7356			9
9	1045.4255	523.2164	1028.3990	514.7031	1027.4149	514.2111	G	***947.4476***	474.2274	930.4210	465.7142			8
10	1201.5266	601.2669	1184.5001	592.7537	1183.5160	592.2617	R	890.4261	445.7167	873.3996	437.2034			7
11	1314.6107	657.8090	1297.5841	649.2957	1296.6001	648.8037	I	734.3250	367.6661	717.2984	359.1529			6
12	1474.6413	737.8243	1457.6148	729.3110	1456.6308	728.8190	C	621.2409	311.1241	604.2144	302.6108			5
13	1588.6843	794.8458	1571.6577	786.3325	1570.6737	785.8405	N	461.2103	231.1088	444.1837	222.5955			4
14	1703.7112	852.3592	1686.6846	843.8460	1685.7006	843.3540	N	347.1674	174.0873	330.1408	165.5740			3
15	1760.7327	880.8700	1743.7061	872.3567	1742.7221	871.8647	G	232.1404	116.5738	215.1139	108.0606			2

16							R	175.1190	88.0631	158.0924	79.5498			1

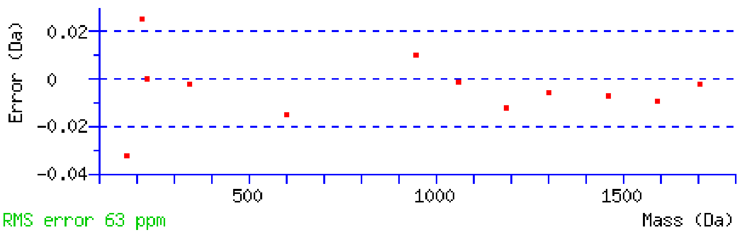

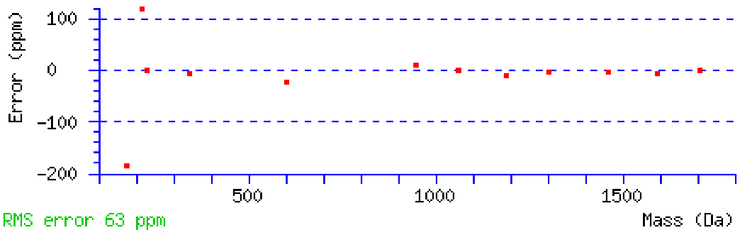

NCBI **BLAST** search of DIDECLQNGRICNNGR
(Parameters: blastp, nr protein database, expect=20000, no filter, PAM30)
Other BLAST web gateways

All matches to this query

Score	Mr(calc)	Delta	Sequence
25.3	1933.8370	-0.0002	DIDECLQNGRICNNGR
25.3	1933.8370	-0.0002	DIDECLQNGRICNNGR
24.1	1933.8370	-0.0002	DIDECLQNGRICNNGR
16.3	1933.8370	-0.0002	DIDECLQNGRICNNGR

Mascot: http://www.matrixscience.com/

Mascot Search Results

Peptide View

MS/MS Fragmentation of **DINECETPGICMNGR**
Found in **P35555** in **uni_human_nr**, FBN1_HUMAN Fibrillin-1 OS=Homo sapiens GN=FBN1 PE=1 SV=3

Match to Query 5929: 1782.679908 from(892.347230,2+) intensity(187371.6406) rtinseconds(1676) scans(8586) index(6357)
Title: 130806_HPL_Human_Plaque_BR1_TR2_20_Spectrum026225_scans__8586_RTINSECONDS=1676
Data file E:\\RAW\\HumanPlaques\\mgf\\T\\1D\\T130806_HPL_Human_Plaque_BR1_TR2_20.mgf

Click mouse within plot area to zoom in by factor of two about that point
Or, [Plot from] [50] to [1050] Da [Full range]
Label all possible matches ○ Label matches used for scoring ⊙
Show Y-axis ☐

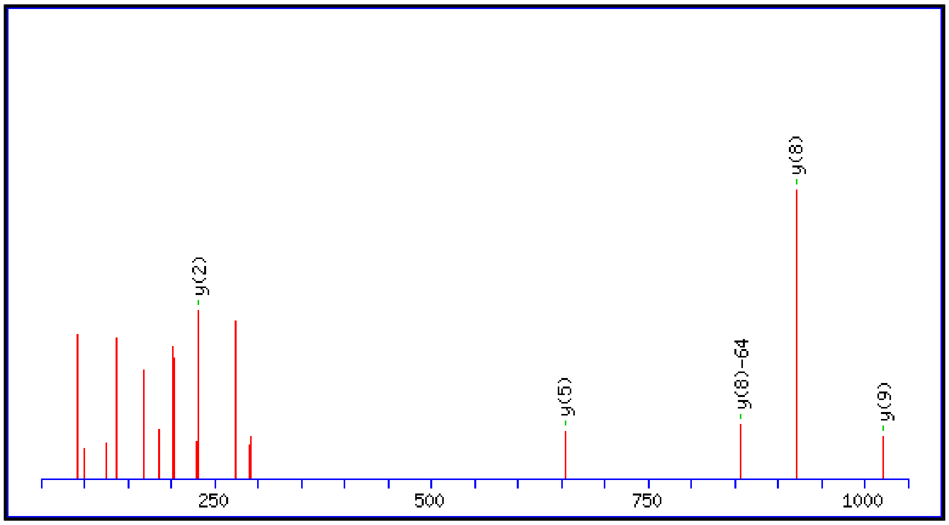

Monoisotopic mass of neutral peptide Mr(calc): 1782.6859
Fixed modifications: Carbamidomethyl (C) (apply to specified residues or termini only)
Variable modifications:
N3 : Deamidated (NQ)
M12 : Oxidation (M), with neutral losses 0.0000(shown in table), 63.9983
N13 : Deamidated (NQ)
Ions Score: 28 **Expect:** 0.005
Matches : 5/222 fragment ions using 6 most intense peaks (help)

#	b	b^{++}	b*	b^{*++}	b^{0}	b^{0++}	Seq.	y	y^{++}	y*	y^{*++}	y^{0}	y^{0++}	#
1	116.0342	58.5207			98.0237	49.5155	D							15
2	229.1183	115.0628			211.1077	106.0575	I	1668.6662	834.8367	1651.6397	826.3235	1650.6557	825.8315	14
3	344.1452	172.5763	327.1187	164.0630	326.1347	163.5710	N	1555.5822	778.2947	1538.5556	769.7814	1537.5716	769.2894	13
4	473.1878	237.0975	456.1613	228.5843	455.1773	228.0923	E	1440.5552	720.7812	1423.5287	712.2680	1422.5446	711.7760	12
5	633.2185	317.1129	616.1919	308.5996	615.2079	308.1076	C	1311.5126	656.2599	1294.4861	647.7467	1293.5021	647.2547	11
6	762.2611	381.6342	745.2345	373.1209	744.2505	372.6289	E	1151.4820	576.2446	1134.4554	567.7313	1133.4714	567.2393	10
7	863.3087	432.1580	846.2822	423.6447	845.2982	423.1527	T	***1022.4394***	511.7233	1005.4128	503.2101	1004.4288	502.7180	9
8	960.3615	480.6844	943.3350	472.1711	942.3509	471.6791	P	***921.3917***	461.1995	904.3652	452.6862			8
9	1017.3830	509.1951	1000.3564	500.6818	999.3724	500.1898	G	824.3389	412.6731	807.3124	404.1598			7
10	1130.4670	565.7372	1113.4405	557.2239	1112.4565	556.7319	I	767.3175	384.1624	750.2909	375.6491			6
11	1290.4977	645.7525	1273.4711	637.2392	1272.4871	636.7472	C	***654.2334***	327.6203	637.2069	319.1071			5
12	1437.5331	719.2702	1420.5065	710.7569	1419.5225	710.2649	M	494.2028	247.6050	477.1762	239.0917			4
13	1552.5600	776.7837	1535.5335	768.2704	1534.5495	767.7784	N	347.1674	174.0873	330.1408	165.5740			3
14	1609.5815	805.2944	1592.5549	796.7811	1591.5709	796.2891	G	***232.1404***	116.5738	215.1139	108.0606			2

15							R	175.1190	88.0631	158.0924	79.5498			1

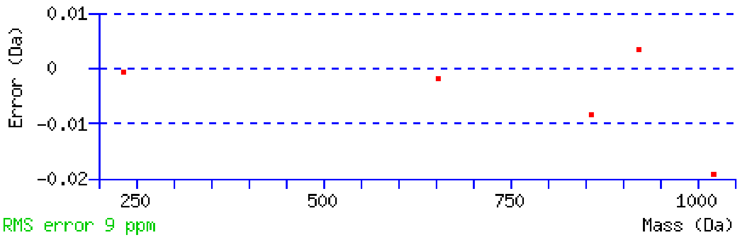

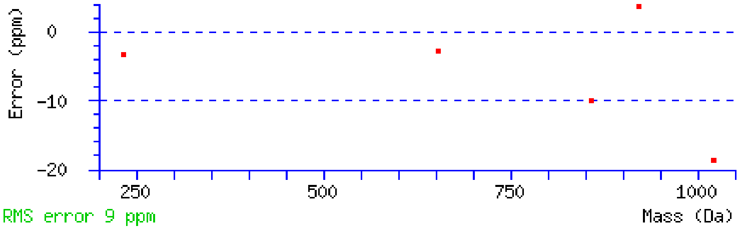

NCBI **BLAST** search of DINECETPGICMNGR
(Parameters: blastp, nr protein database, expect=20000, no filter, PAM30)
Other BLAST web gateways

All matches to this query

Score	Mr(calc)	Delta	Sequence
27.6	1782.6859	-0.0060	DINECETPGICMNGR

Mascot: http://www.matrixscience.com/

Mascot Search Results

Peptide View

MS/MS Fragmentation of **DINECETPGICMNGR**
Found in **P35555** in **uni_human_nr**, FBN1_HUMAN Fibrillin-1 OS=Homo sapiens GN=FBN1 PE=1 SV=3

Match to Query 5049: 1781.700168 from(891.857360,2+) intensity(2438321.2500) rtinseconds(1589) scans(7800) index(5576)
Title: 130801_HPL_Human_Plaque_BR1_TR1_20_Spectrum024711_scans__7800_RTINSECONDS=1589
Data file E:\\RAW\\HumanPlaques\\mgf\\T\\1D\\T130801_HPL_Human_Plaque_BR1_TR1_20.mgf

Click mouse within plot area to zoom in by factor of two about that point
Or, [Plot from] [0] to [1600] Da [Full range]
Label all possible matches ○ Label matches used for scoring ⊙
Show Y-axis ☐

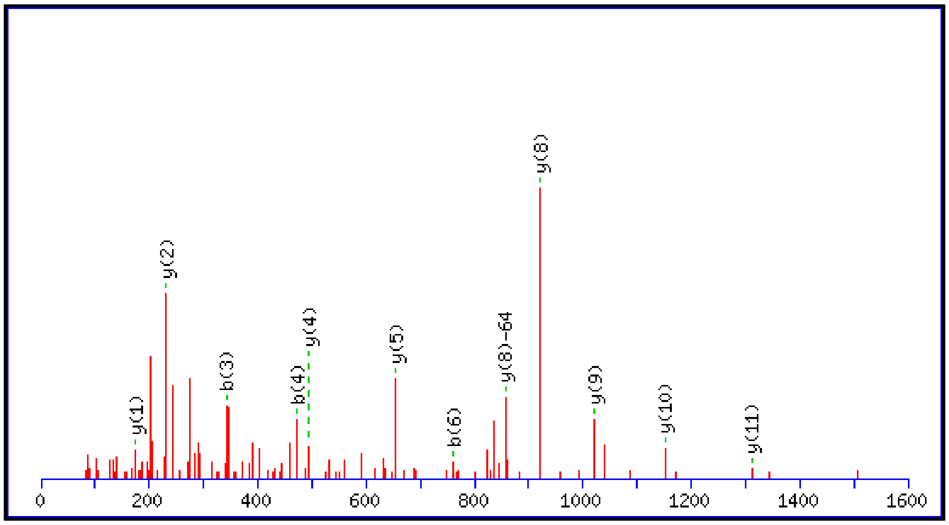

Monoisotopic mass of neutral peptide Mr(calc): 1781.7019
Fixed modifications: Carbamidomethyl (C) (apply to specified residues or termini only)
Variable modifications:
M12 : Oxidation (M), with neutral losses 0.0000(shown in table), 63.9983
N13 : Deamidated (NQ)
Ions Score: 73 **Expect:** 1.9e-007
Matches : 12/222 fragment ions using 13 most intense peaks (help)

#	b	b^{++}	b*	b*$^{++}$	b^{0}	b^{0++}	Seq.	y	y^{++}	y*	y*$^{++}$	y^{0}	y^{0++}	#
1	116.0342	58.5207			98.0237	49.5155	D							15
2	229.1183	115.0628			211.1077	106.0575	I	1667.6822	834.3447	1650.6557	825.8315	1649.6716	825.3395	14
3	**343.1612**	172.0842	326.1347	163.5710	325.1506	163.0790	N	1554.5981	777.8027	1537.5716	769.2894	1536.5876	768.7974	13
4	**472.2038**	236.6055	455.1773	228.0923	454.1932	227.6003	E	1440.5552	720.7812	1423.5287	712.2680	1422.5446	711.7760	12
5	632.2345	316.6209	615.2079	308.1076	614.2239	307.6156	C	***1311.5126***	656.2599	1294.4861	647.7467	1293.5021	647.2547	11
6	**761.2770**	381.1422	744.2505	372.6289	743.2665	372.1369	E	***1151.4820***	576.2446	1134.4554	567.7313	1133.4714	567.2393	10
7	862.3247	431.6660	845.2982	423.1527	844.3142	422.6607	T	***1022.4394***	511.7233	1005.4128	503.2101	1004.4288	502.7180	9
8	959.3775	480.1924	942.3509	471.6791	941.3669	471.1871	P	***921.3917***	461.1995	904.3652	452.6862			8
9	1016.3990	508.7031	999.3724	500.1898	998.3884	499.6978	G	824.3389	412.6731	807.3124	404.1598			7
10	1129.4830	565.2451	1112.4565	556.7319	1111.4725	556.2399	I	767.3175	384.1624	750.2909	375.6491			6
11	1289.5137	645.2605	1272.4871	636.7472	1271.5031	636.2552	C	***654.2334***	327.6203	637.2069	319.1071			5
12	1436.5491	718.7782	1419.5225	710.2649	1418.5385	709.7729	M	***494.2028***	247.6050	477.1762	239.0917			4
13	1551.5760	776.2916	1534.5495	767.7784	1533.5654	767.2864	N	347.1674	174.0873	330.1408	165.5740			3
14	1608.5975	804.8024	1591.5709	796.2891	1590.5869	795.7971	G	***232.1404***	116.5738	215.1139	108.0606			2
15							R	***175.1190***	88.0631	158.0924	79.5498			1

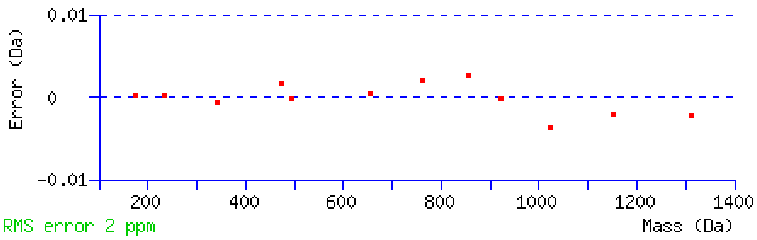

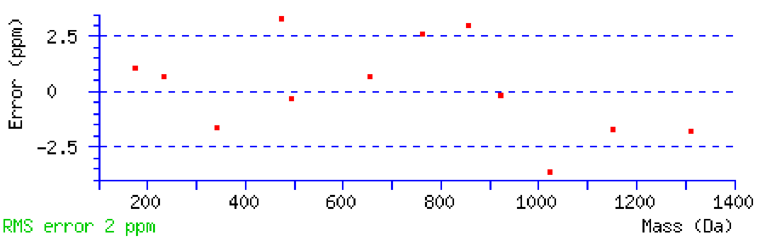

NCBI **BLAST** search of DINECETPGICMNGR
(Parameters: blastp, nr protein database, expect=20000, no filter, PAM30)
Other BLAST web gateways

All matches to this query

Score	Mr(calc)	Delta	Sequence
73.3	1781.7019	-0.0017	DINECETPGICMNGR

**Mascot:** http://www.matrixscience.com/

Mascot Search Results

Peptide View

MS/MS Fragmentation of **DINECETPGICMNGR**
Found in **P35555** in **uni_human_nr**, FBN1_HUMAN Fibrillin-1 OS=Homo sapiens GN=FBN1 PE=1 SV=3

Match to Query 5506: 1780.716888 from(891.365720,2+) intensity(984349.7500) rtinseconds(1513) scans(7226) index(4836)
Title: 130806_HPL_Human_Plaque_BR1_TR2_15_Spectrum025167_scans__7226_RTINSECONDS=1513
Data file E:\\RAW\\HumanPlaques\\mgf\\T\\1D\\T130806_HPL_Human_Plaque_BR1_TR2_15.mgf

Click mouse within plot area to zoom in by factor of two about that point
Or, Plot from 0 to 1400 Da Full range
Label all possible matches ○ Label matches used for scoring ⊙
Show Y-axis ☐

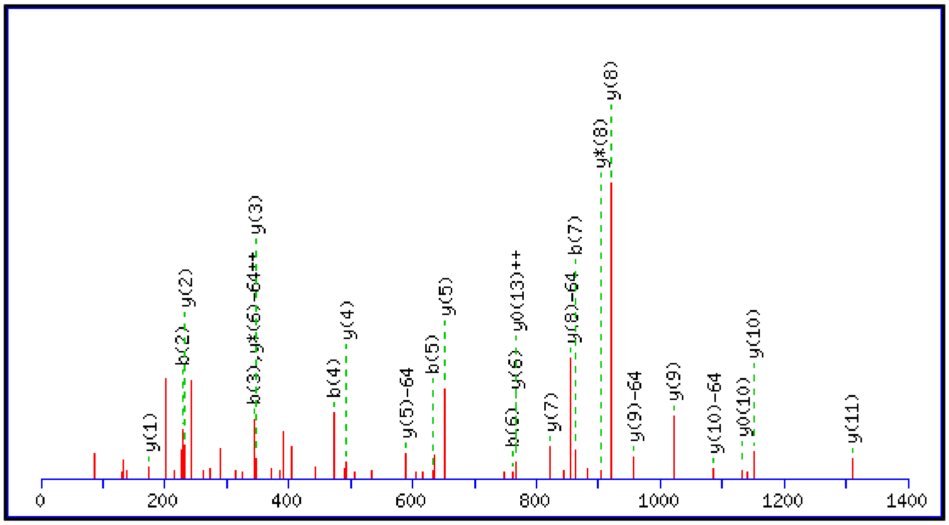

Monoisotopic mass of neutral peptide Mr(calc): 1780.7179
Fixed modifications: Carbamidomethyl (C) (apply to specified residues or termini only)
Variable modifications:
M12 : Oxidation (M), with neutral losses 0.0000(shown in table), 63.9983
Ions Score: 75 **Expect:** 2.8e-007
Matches : 25/222 fragment ions using 42 most intense peaks (help)

#	b	b++	b*	b*++	b0	b0++	Seq.	y	y++	y*	y*++	y0	y0++	#
1	116.0342	58.5207			98.0237	49.5155	D							15
2	**229.1183**	115.0628			211.1077	106.0575	I	1666.6982	833.8527	1649.6716	825.3395	1648.6876	824.8474	14
3	**343.1612**	172.0842	326.1347	163.5710	325.1506	163.0790	N	1553.6141	777.3107	1536.5876	768.7974	1535.6036	768.3054	13
4	**472.2038**	236.6055	455.1773	228.0923	454.1932	227.6003	E	1439.5712	720.2892	1422.5446	711.7760	1421.5606	711.2840	12
5	**632.2345**	316.6209	615.2079	308.1076	614.2239	307.6156	C	***1310.5286***	655.7679	1293.5021	647.2547	1292.5180	646.7627	11
6	**761.2770**	381.1422	744.2505	372.6289	743.2665	372.1369	E	***1150.4980***	575.7526	1133.4714	567.2393	1132.4874	566.7473	10
7	**862.3247**	431.6660	845.2982	423.1527	844.3142	422.6607	T	***1021.4554***	511.2313	1004.4288	502.7180	1003.4448	502.2260	9
8	959.3775	480.1924	942.3509	471.6791	941.3669	471.1871	P	***920.4077***	460.7075	903.3811	452.1942			8
9	1016.3990	508.7031	999.3724	500.1898	998.3884	499.6978	G	***823.3549***	412.1811	806.3284	403.6678			7
10	1129.4830	565.2451	1112.4565	556.7319	1111.4725	556.2399	I	***766.3335***	383.6704	749.3069	375.1571			6
11	1289.5137	645.2605	1272.4871	636.7472	1271.5031	636.2552	C	***653.2494***	327.1283	636.2228	318.6151			5
12	1436.5491	718.7782	1419.5225	710.2649	1418.5385	709.7729	M	***493.2187***	247.1130	476.1922	238.5997			4
13	1550.5920	775.7996	1533.5654	767.2864	1532.5814	766.7944	N	***346.1833***	173.5953	329.1568	165.0820			3
14	1607.6135	804.3104	1590.5869	795.7971	1589.6029	795.3051	G	***232.1404***	116.5738	215.1139	108.0606			2
15							R	***175.1190***	88.0631	158.0924	79.5498			1

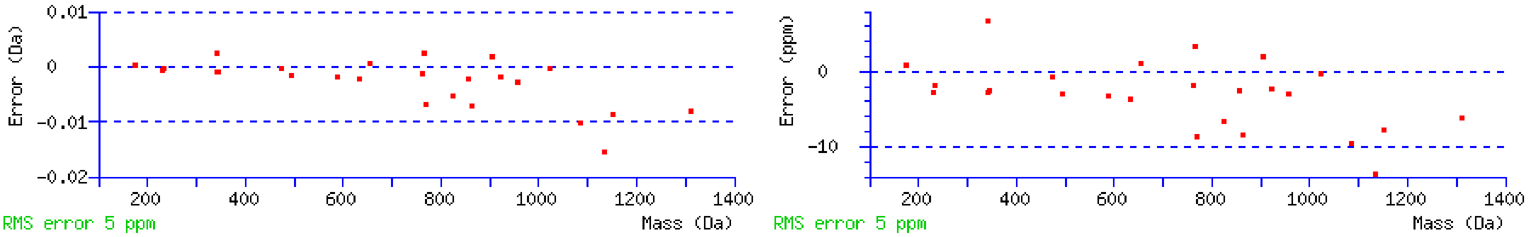

NCBI **BLAST** search of DINECETPGICMNGR
(Parameters: blastp, nr protein database, expect=20000, no filter, PAM30)
Other BLAST web gateways

All matches to this query

Score	Mr(calc)	Delta	Sequence
74.8	1780.7179	-0.0010	DINECETPGICMNGR

Mascot: http://www.matrixscience.com/

Mascot Search Results

Peptide View

MS/MS Fragmentation of **DINECETPGICMNGR**
Found in **P35555** in **uni_human_nr**, FBN1_HUMAN Fibrillin-1 OS=Homo sapiens GN=FBN1 PE=1 SV=3

Match to Query 5706: 1780.717122 from(594.579650,3+) intensity(619573.0625) rtinseconds(1506) scans(7268) index(4872)
Title: 130809_HPL_Human_Plaque_BR2_TR3_16_Spectrum024459_scans__7268_RTINSECONDS=1506
Data file E:\\RAW\\HumanPlaques\\mgf\\T\\1D\\T130809_HPL_Human_Plaque_BR2_TR3_16.mgf

Click mouse within plot area to zoom in by factor of two about that point
Or, Plot from 50 to 950 Da Full range
Label all possible matches ○ Label matches used for scoring ⊙
Show Y-axis ☐

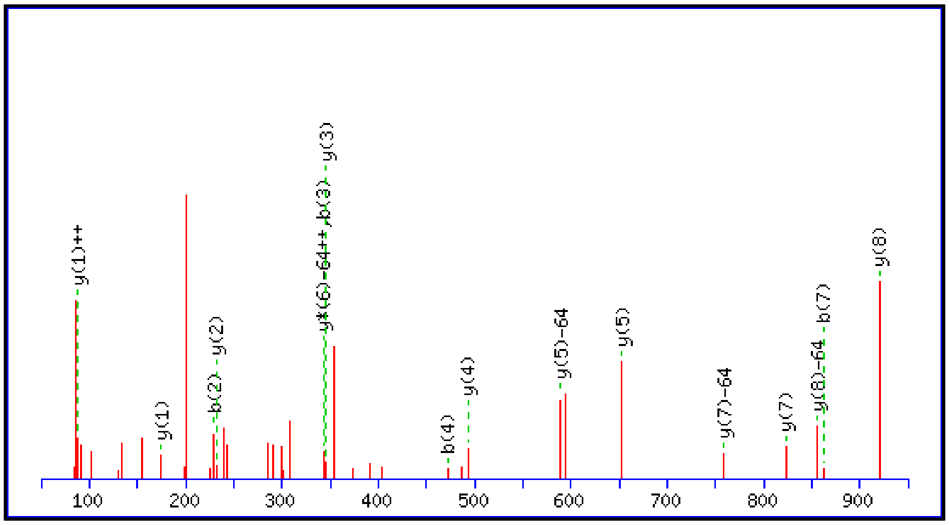

Monoisotopic mass of neutral peptide Mr(calc): 1780.7179
Fixed modifications: Carbamidomethyl (C) (apply to specified residues or termini only)
Variable modifications:
M12 : Oxidation (M), with neutral losses 0.0000(shown in table), 63.9983
Ions Score: 34 **Expect:** 0.0033
Matches : 16/222 fragment ions using 37 most intense peaks (help)

#	b	b^{++}	b*	b^{*++}	b^0	b^{0++}	Seq.	y	y^{++}	y*	y^{*++}	y^0	y^{0++}	#
1	116.0342	58.5207			98.0237	49.5155	D							15
2	***229.1183***	115.0628			211.1077	106.0575	I	1666.6982	833.8527	1649.6716	825.3395	1648.6876	824.8474	14
3	***343.1612***	172.0842	326.1347	163.5710	325.1506	163.0790	N	1553.6141	777.3107	1536.5876	768.7974	1535.6036	768.3054	13
4	***472.2038***	236.6055	455.1773	228.0923	454.1932	227.6003	E	1439.5712	720.2892	1422.5446	711.7760	1421.5606	711.2840	12
5	632.2345	316.6209	615.2079	308.1076	614.2239	307.6156	C	1310.5286	655.7679	1293.5021	647.2547	1292.5180	646.7627	11
6	761.2770	381.1422	744.2505	372.6289	743.2665	372.1369	E	1150.4980	575.7526	1133.4714	567.2393	1132.4874	566.7473	10
7	***862.3247***	431.6660	845.2982	423.1527	844.3142	422.6607	T	1021.4554	511.2313	1004.4288	502.7180	1003.4448	502.2260	9
8	959.3775	480.1924	942.3509	471.6791	941.3669	471.1871	P	***920.4077***	460.7075	903.3811	452.1942			8
9	1016.3990	508.7031	999.3724	500.1898	998.3884	499.6978	G	***823.3549***	412.1811	806.3284	403.6678			7
10	1129.4830	565.2451	1112.4565	556.7319	1111.4725	556.2399	I	766.3335	383.6704	749.3069	375.1571			6
11	1289.5137	645.2605	1272.4871	636.7472	1271.5031	636.2552	C	***653.2494***	327.1283	636.2228	318.6151			5
12	1436.5491	718.7782	1419.5225	710.2649	1418.5385	709.7729	M	***493.2187***	247.1130	476.1922	238.5997			4
13	1550.5920	775.7996	1533.5654	767.2864	1532.5814	766.7944	N	***346.1833***	173.5953	329.1568	165.0820			3
14	1607.6135	804.3104	1590.5869	795.7971	1589.6029	795.3051	G	***232.1404***	116.5738	215.1139	108.0606			2
15							R	***175.1190***	88.0631	158.0924	79.5498			1

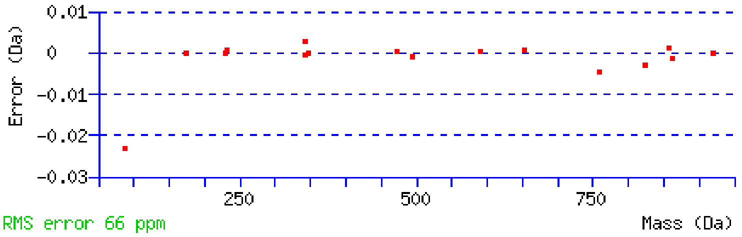

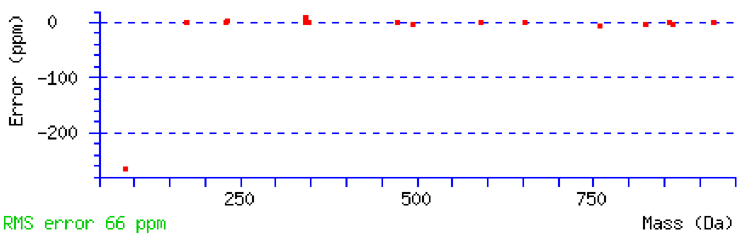

NCBI **BLAST** search of DINECETPGICMNGR
(Parameters: blastp, nr protein database, expect=20000, no filter, PAM30)
Other BLAST web gateways

All matches to this query

Score	Mr(calc)	Delta	Sequence
34.1	1780.7179	-0.0007	DINECETPGICMNGR

Mascot: http://www.matrixscience.com/

Mascot Search Results

Peptide View

MS/MS Fragmentation of **DINECETPGICMNGR**
Found in **P35555** in **uni_human_nr**, FBN1_HUMAN Fibrillin-1 OS=Homo sapiens GN=FBN1 PE=1 SV=3

Match to Query 5473: 1765.706388 from(883.860470,2+) intensity(2866075.5000) rtinseconds(1943) scans(10308) index(7878)
Title: 130809_HPL_Human_Plaque_BR1_TR3_20_Spectrum027504_scans__10308_RTINSECONDS=1943
Data file E:\\RAW\\HumanPlaques\\mgf\\T\\1D\\T130809_HPL_Human_Plaque_BR1_TR3_20.mgf

Click mouse within plot area to zoom in by factor of two about that point
Or, Plot from 0 to 1600 Da Full range
Label all possible matches ○ Label matches used for scoring ⊙
Show Y-axis ☐

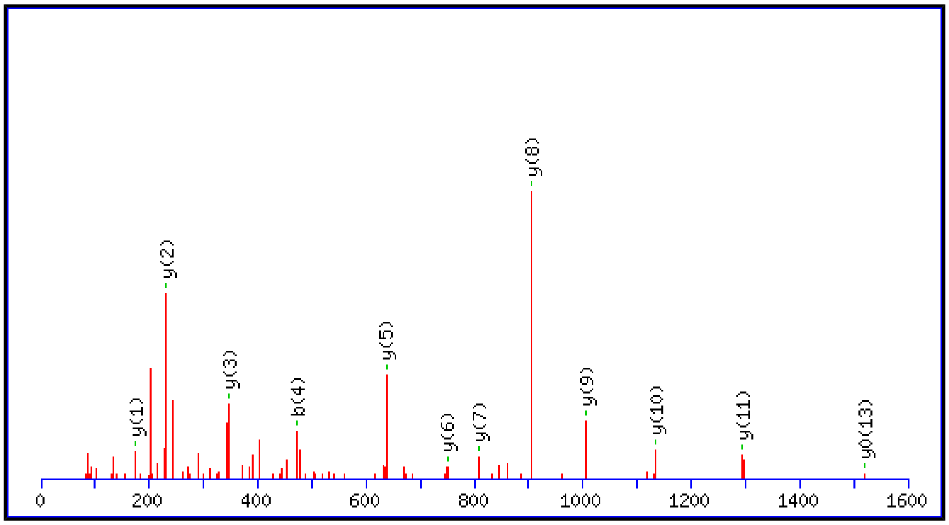

Monoisotopic mass of neutral peptide Mr(calc): 1765.7069
Fixed modifications: Carbamidomethyl (C) (apply to specified residues or termini only)
Variable modifications:
N13 : Deamidated (NQ)
Ions Score: 102 **Expect:** 3.7e-010
Matches : 12/148 fragment ions using 13 most intense peaks (help)

#	b	b^{++}	b*	b^{*++}	b^0	b^{0++}	Seq.	y	y^{++}	y*	y^{*++}	y^0	y^{0++}	#
1	116.0342	58.5207			98.0237	49.5155	D							15
2	229.1183	115.0628			211.1077	106.0575	I	1651.6873	826.3473	1634.6607	817.8340	1633.6767	817.3420	14
3	343.1612	172.0842	326.1347	163.5710	325.1506	163.0790	N	1538.6032	769.8053	1521.5767	761.2920	1520.5927	760.8000	13
4	**472.2038**	236.6055	455.1773	228.0923	454.1932	227.6003	E	1424.5603	712.7838	1407.5338	704.2705	1406.5497	703.7785	12
5	632.2345	316.6209	615.2079	308.1076	614.2239	307.6156	C	***1295.5177***	648.2625	1278.4912	639.7492	1277.5071	639.2572	11
6	761.2770	381.1422	744.2505	372.6289	743.2665	372.1369	E	***1135.4871***	568.2472	1118.4605	559.7339	1117.4765	559.2419	10
7	862.3247	431.6660	845.2982	423.1527	844.3142	422.6607	T	***1006.4445***	503.7259	989.4179	495.2126	988.4339	494.7206	9
8	959.3775	480.1924	942.3509	471.6791	941.3669	471.1871	P	***905.3968***	453.2020	888.3702	444.6888			8
9	1016.3990	508.7031	999.3724	500.1898	998.3884	499.6978	G	***808.3440***	404.6756	791.3175	396.1624			7
10	1129.4830	565.2451	1112.4565	556.7319	1111.4725	556.2399	I	***751.3226***	376.1649	734.2960	367.6516			6
11	1289.5137	645.2605	1272.4871	636.7472	1271.5031	636.2552	C	***638.2385***	319.6229	621.2119	311.1096			5
12	1420.5542	710.7807	1403.5276	702.2674	1402.5436	701.7754	M	478.2078	239.6076	461.1813	231.0943			4
13	1535.5811	768.2942	1518.5545	759.7809	1517.5705	759.2889	N	***347.1674***	174.0873	330.1408	165.5740			3
14	1592.6026	796.8049	1575.5760	788.2916	1574.5920	787.7996	G	***232.1404***	116.5738	215.1139	108.0606			2
15							R	***175.1190***	88.0631	158.0924	79.5498			1

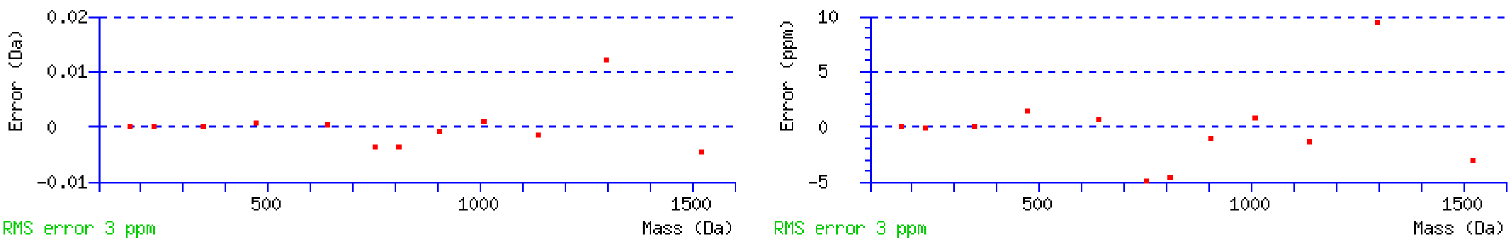

NCBI **BLAST** search of DINECETPGICMNGR
(Parameters: blastp, nr protein database, expect=20000, no filter, PAM30)
Other BLAST web gateways

All matches to this query

Score	Mr(calc)	Delta	Sequence
101.5	1765.7069	-0.0006	DINECETPGICMNGR
0.6	1764.7081	0.9982	RDDASMDRDDNQDGR

Mascot: http://www.matrixscience.com/

Mascot Search Results

Peptide View

MS/MS Fragmentation of **DINECETPGICMNGR**
Found in **P35555** in **uni_human_nr**, FBN1_HUMAN Fibrillin-1 OS=Homo sapiens GN=FBN1 PE=1 SV=3

Match to Query 5474: 1765.709982 from(589.577270,3+) intensity(113877.3594) rtinseconds(1944) scans(10311) index(7881)
Title: 130809_HPL_Human_Plaque_BR1_TR3_20_Spectrum027507_scans__10311_RTINSECONDS=1944
Data file E:\\RAW\\HumanPlaques\\mgf\\T\\1D\\T130809_HPL_Human_Plaque_BR1_TR3_20.mgf

Click mouse within plot area to zoom in by factor of two about that point
Or, Plot from 50 to 950 Da Full range
Label all possible matches ○ Label matches used for scoring ⊙
Show Y-axis ☐

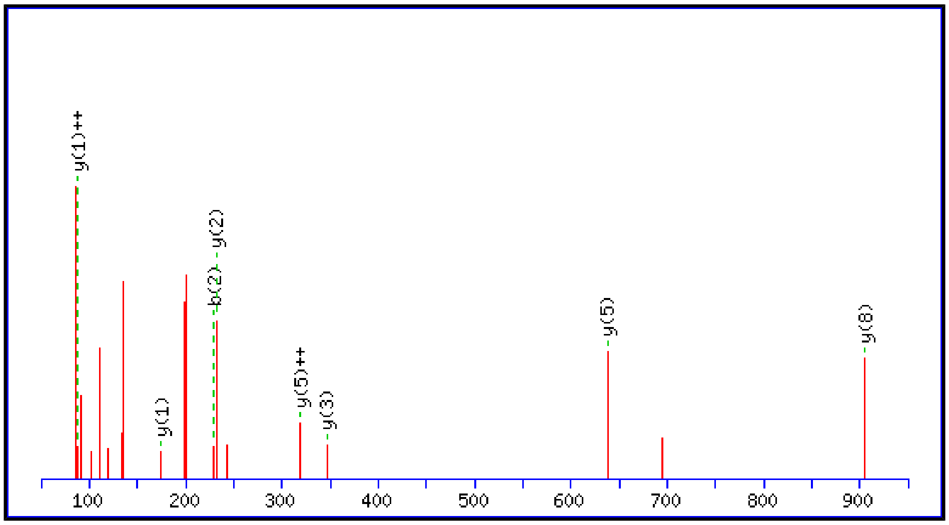

Monoisotopic mass of neutral peptide Mr(calc): 1765.7069
Fixed modifications: Carbamidomethyl (C) (apply to specified residues or termini only)
Variable modifications:
N13 : Deamidated (NQ)
Ions Score: 20 **Expect:** 0.064
Matches : 8/148 fragment ions using 19 most intense peaks (help)

#	b	b^{++}	b*	b^{*++}	b^0	b^{0++}	Seq.	y	y^{++}	y*	y^{*++}	y^0	y^{0++}	#
1	116.0342	58.5207			98.0237	49.5155	D							15
2	**229.1183**	115.0628			211.1077	106.0575	I	1651.6873	826.3473	1634.6607	817.8340	1633.6767	817.3420	14
3	343.1612	172.0842	326.1347	163.5710	325.1506	163.0790	N	1538.6032	769.8053	1521.5767	761.2920	1520.5927	760.8000	13
4	472.2038	236.6055	455.1773	228.0923	454.1932	227.6003	E	1424.5603	712.7838	1407.5338	704.2705	1406.5497	703.7785	12
5	632.2345	316.6209	615.2079	308.1076	614.2239	307.6156	C	1295.5177	648.2625	1278.4912	639.7492	1277.5071	639.2572	11
6	761.2770	381.1422	744.2505	372.6289	743.2665	372.1369	E	1135.4871	568.2472	1118.4605	559.7339	1117.4765	559.2419	10
7	862.3247	431.6660	845.2982	423.1527	844.3142	422.6607	T	1006.4445	503.7259	989.4179	495.2126	988.4339	494.7206	9
8	959.3775	480.1924	942.3509	471.6791	941.3669	471.1871	P	***905.3968***	453.2020	888.3702	444.6888			8
9	1016.3990	508.7031	999.3724	500.1898	998.3884	499.6978	G	808.3440	404.6756	791.3175	396.1624			7
10	1129.4830	565.2451	1112.4565	556.7319	1111.4725	556.2399	I	751.3226	376.1649	734.2960	367.6516			6
11	1289.5137	645.2605	1272.4871	636.7472	1271.5031	636.2552	C	***638.2385***	319.6229	621.2119	311.1096			5
12	1420.5542	710.7807	1403.5276	702.2674	1402.5436	701.7754	M	478.2078	239.6076	461.1813	231.0943			4
13	1535.5811	768.2942	1518.5545	759.7809	1517.5705	759.2889	N	***347.1674***	174.0873	330.1408	165.5740			3
14	1592.6026	796.8049	1575.5760	788.2916	1574.5920	787.7996	G	***232.1404***	116.5738	215.1139	108.0606			2
15							R	***175.1190***	88.0631	158.0924	79.5498			1

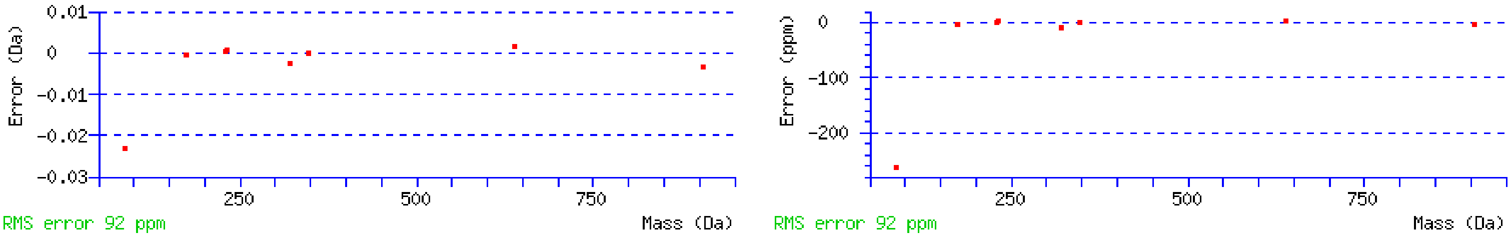

NCBI **BLAST** search of DINECETPGICMNGR
(Parameters: blastp, nr protein database, expect=20000, no filter, PAM30)
Other BLAST web gateways

All matches to this query

Score	Mr(calc)	Delta	Sequence
20.3	1765.7069	0.0030	DINECETPGICMNGR

Mascot: http://www.matrixscience.com/

Mascot Search Results

Peptide View

MS/MS Fragmentation of **DINECETPGICMNGR**
Found in **P35555** in **uni_human_nr**, FBN1_HUMAN Fibrillin-1 OS=Homo sapiens GN=FBN1 PE=1 SV=3

Match to Query 5426: 1764.721768 from(883.368160,2+) intensity(20760472.0000) rtinseconds(1829) scans(9355) index(6637)
Title: 130806_HPL_Human_Plaque_BR1_TR2_15_Spectrum026968_scans__9355_RTINSECONDS=1829
Data file E:\\RAW\\HumanPlaques\\mgf0\\T\\1D\\T130806_HPL_Human_Plaque_BR1_TR2_15.mgf

Click mouse within plot area to zoom in by factor of two about that point
Or, Plot from 0 to 1300 Da Full range
Label all possible matches ○ Label matches used for scoring ⊙
Show Y-axis ☐

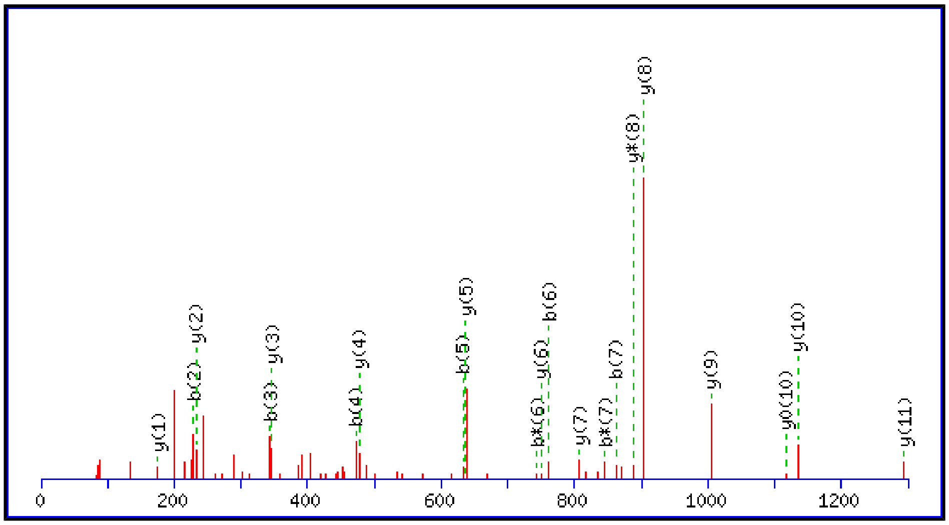

Monoisotopic mass of neutral peptide Mr(calc): 1764.7229
Fixed modifications: Carbamidomethyl (C) (apply to specified residues or termini only)
Ions Score: 77 **Expect:** 2.2e-007
Matches : 21/148 fragment ions using 37 most intense peaks (help)

#	b	b^{++}	b*	b^{*++}	b^0	b^{0++}	Seq.	y	y^{++}	y*	y^{*++}	y^0	y^{0++}	#
1	116.0342	58.5207			98.0237	49.5155	D							15
2	**229.1183**	115.0628			211.1077	106.0575	I	1650.7033	825.8553	1633.6767	817.3420	1632.6927	816.8500	14
3	**343.1612**	172.0842	326.1347	163.5710	325.1506	163.0790	N	1537.6192	769.3132	1520.5927	760.8000	1519.6086	760.3080	13
4	**472.2038**	236.6055	455.1773	228.0923	454.1932	227.6003	E	1423.5763	712.2918	1406.5497	703.7785	1405.5657	703.2865	12
5	**632.2345**	316.6209	615.2079	308.1076	614.2239	307.6156	C	***1294.5337***	647.7705	1277.5071	639.2572	1276.5231	638.7652	11
6	**761.2770**	381.1422	744.2505	372.6289	743.2665	372.1369	E	***1134.5030***	567.7552	1117.4765	559.2419	1116.4925	558.7499	10
7	**862.3247**	431.6660	845.2982	423.1527	844.3142	422.6607	T	***1005.4604***	503.2339	988.4339	494.7206	987.4499	494.2286	9
8	959.3775	480.1924	942.3509	471.6791	941.3669	471.1871	P	***904.4128***	452.7100	887.3862	444.1967			8
9	1016.3990	508.7031	999.3724	500.1898	998.3884	499.6978	G	***807.3600***	404.1836	790.3335	395.6704			7
10	1129.4830	565.2451	1112.4565	556.7319	1111.4725	556.2399	I	***750.3385***	375.6729	733.3120	367.1596			6
11	1289.5137	645.2605	1272.4871	636.7472	1271.5031	636.2552	C	***637.2545***	319.1309	620.2279	310.6176			5
12	1420.5542	710.7807	1403.5276	702.2674	1402.5436	701.7754	M	***477.2238***	239.1156	460.1973	230.6023			4
13	1534.5971	767.8022	1517.5705	759.2889	1516.5865	758.7969	N	***346.1833***	173.5953	329.1568	165.0820			3
14	1591.6185	796.3129	1574.5920	787.7996	1573.6080	787.3076	G	***232.1404***	116.5738	215.1139	108.0606			2
15							R	***175.1190***	88.0631	158.0924	79.5498			1

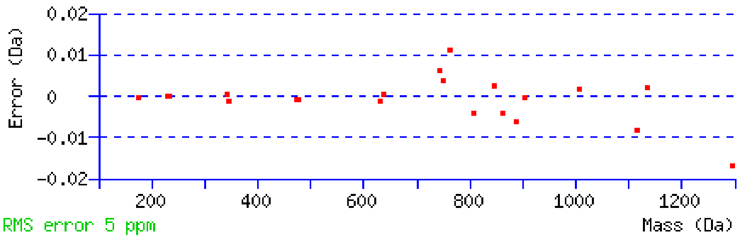

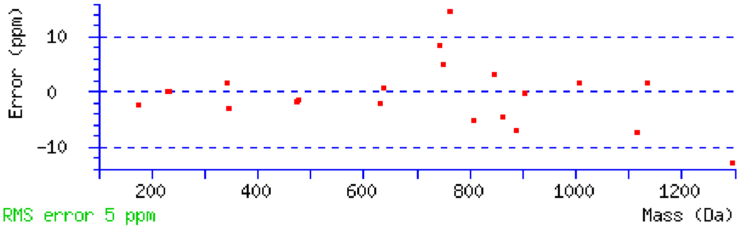

NCBI **BLAST** search of DINECETPGICMNGR
(Parameters: blastp, nr protein database, expect=20000, no filter, PAM30)
Other BLAST web gateways

All matches to this query

Score	Mr(calc)	Delta	Sequence
76.7	1764.7229	-0.0012	DINECETPGICMNGR

Mascot: http://www.matrixscience.com/

Mascot Search Results

Peptide View

MS/MS Fragmentation of **DINECETPGICMNGR**
Found in **P35555** in **uni_human_nr**, FBN1_HUMAN Fibrillin-1 OS=Homo sapiens GN=FBN1 PE=1 SV=3

Match to Query 5136: 1764.723582 from(589.248470,3+) intensity(1112687.2500) rtinseconds(1826) scans(9175) index(6343)
Title: 130801_HPL_Human_Plaque_BR1_TR1_15_Spectrum026496_scans__9175_RTINSECONDS=1826
Data file E:\\RAW\\HumanPlaques\\mgf\\T\\1D\\T130801_HPL_Human_Plaque_BR1_TR1_15.mgf

Click mouse within plot area to zoom in by factor of two about that point
Or, [Plot from] 50 to 1050 Da [Full range]
Label all possible matches ○ Label matches used for scoring ⊙
Show Y-axis ☐

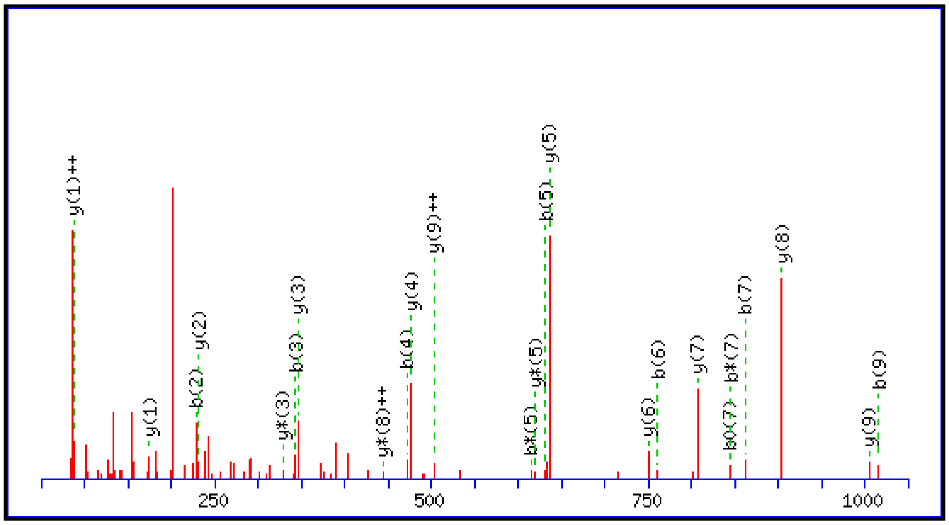

Monoisotopic mass of neutral peptide Mr(calc): 1764.7229
Fixed modifications: Carbamidomethyl (C) (apply to specified residues or termini only)
Ions Score: 58 **Expect:** 1.8e-005
Matches : 24/148 fragment ions using 51 most intense peaks (help)

#	b	b^{++}	b*	b^{*++}	b^0	b^{0++}	Seq.	y	y^{++}	y*	y^{*++}	y^0	y^{0++}	#
1	116.0342	58.5207			98.0237	49.5155	D							15
2	***229.1183***	115.0628			211.1077	106.0575	I	1650.7033	825.8553	1633.6767	817.3420	1632.6927	816.8500	14
3	***343.1612***	172.0842	326.1347	163.5710	325.1506	163.0790	N	1537.6192	769.3132	1520.5927	760.8000	1519.6086	760.3080	13
4	***472.2038***	236.6055	455.1773	228.0923	454.1932	227.6003	E	1423.5763	712.2918	1406.5497	703.7785	1405.5657	703.2865	12
5	***632.2345***	316.6209	615.2079	308.1076	614.2239	307.6156	C	1294.5337	647.7705	1277.5071	639.2572	1276.5231	638.7652	11
6	***761.2770***	381.1422	744.2505	372.6289	743.2665	372.1369	E	1134.5030	567.7552	1117.4765	559.2419	1116.4925	558.7499	10
7	***862.3247***	431.6660	845.2982	423.1527	844.3142	422.6607	T	***1005.4604***	503.2339	988.4339	494.7206	987.4499	494.2286	9
8	959.3775	480.1924	942.3509	471.6791	941.3669	471.1871	P	***904.4128***	452.7100	887.3862	444.1967			8
9	***1016.3990***	508.7031	999.3724	500.1898	998.3884	499.6978	G	***807.3600***	404.1836	790.3335	395.6704			7
10	1129.4830	565.2451	1112.4565	556.7319	1111.4725	556.2399	I	***750.3385***	375.6729	733.3120	367.1596			6
11	1289.5137	645.2605	1272.4871	636.7472	1271.5031	636.2552	C	***637.2545***	319.1309	620.2279	310.6176			5
12	1420.5542	710.7807	1403.5276	702.2674	1402.5436	701.7754	M	***477.2238***	239.1156	460.1973	230.6023			4
13	1534.5971	767.8022	1517.5705	759.2889	1516.5865	758.7969	N	***346.1833***	173.5953	329.1568	165.0820			3
14	1591.6185	796.3129	1574.5920	787.7996	1573.6080	787.3076	G	***232.1404***	116.5738	215.1139	108.0606			2
15							R	***175.1190***	88.0631	158.0924	79.5498			1

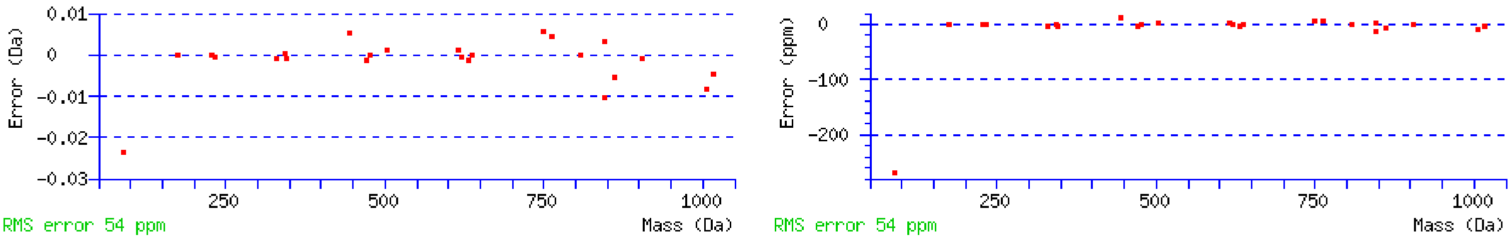

NCBI **BLAST** search of DINECETPGICMNGR
(Parameters: blastp, nr protein database, expect=20000, no filter, PAM30)
Other BLAST web gateways

All matches to this query

Score	Mr(calc)	Delta	Sequence
57.7	1764.7229	0.0007	DINECETPGICMNGR

Mascot: http://www.matrixscience.com/

Mascot Search Results

Peptide View

MS/MS Fragmentation of **RPDGEGCVDENECQTKPGICENGR**
Found in **P35555** in **uni_human_nr**, FBN1_HUMAN Fibrillin-1 OS=Homo sapiens GN=FBN1 PE=1 SV=3

Match to Query 13239: 2777.141776 from(695.292720,4+) intensity(1093211.5000) rtinseconds(1196) scans(5388) index(3379)
Title: 130806_HPL_Human_Plaque_BR1_TR2_18_Spectrum023527_scans__5388_RTINSECONDS=1196
Data file E:\\RAW\\HumanPlaques\\mgf\\T\\1D\\T130806_HPL_Human_Plaque_BR1_TR2_18.mgf

Click mouse within plot area to zoom in by factor of two about that point
Or, Plot from 0 to 2800 Da Full range
Label all possible matches ○ Label matches used for scoring ⊙
Show Y-axis ☐

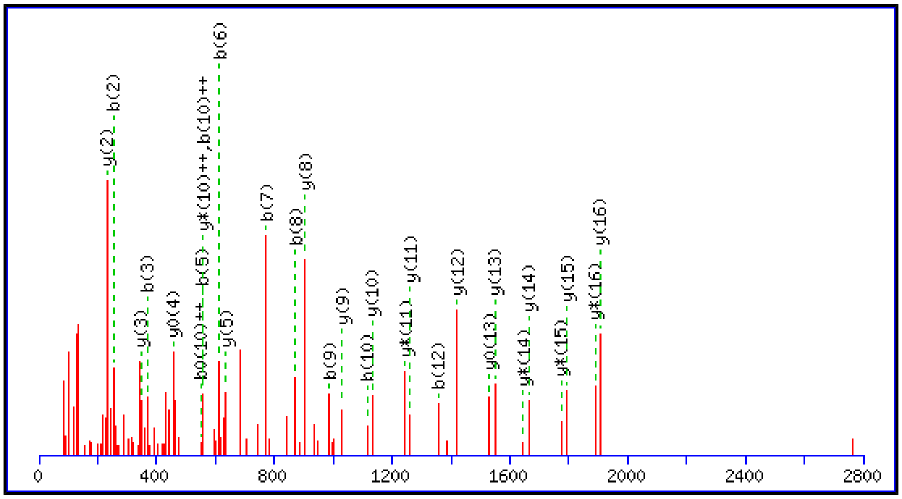


```
Monoisotopic mass of neutral peptide Mr(calc): 2777.1439
Fixed modifications: Carbamidomethyl (C) (apply to specified residues or termini only)
Variable modifications:
N22     : Deamidated (NQ)
Ions Score: 92  Expect: 5.7e-009
Matches : 30/266 fragment ions using 48 most intense peaks   (help)
```

#	b	b^{++}	b*	b^{*++}	b^0	b^{0++}	Seq.	y	y^{++}	y*	y^{*++}	y^0	y^{0++}	#
1	157.1084	79.0578	140.0818	70.5446			R							24
2	***254.1612***	127.5842	237.1346	119.0709			P	2622.0501	1311.5287	2605.0236	1303.0154	2604.0395	1302.5234	23
3	***369.1881***	185.0977	352.1615	176.5844	351.1775	176.0924	D	2524.9973	1263.0023	2507.9708	1254.4890	2506.9868	1253.9970	22
4	426.2096	213.6084	409.1830	205.0951	408.1990	204.6031	G	2409.9704	1205.4888	2392.9439	1196.9756	2391.9598	1196.4836	21
5	***555.2522***	278.1297	538.2256	269.6164	537.2416	269.1244	E	2352.9489	1176.9781	2335.9224	1168.4648	2334.9384	1167.9728	20
6	***612.2736***	306.6404	595.2471	298.1272	594.2631	297.6352	G	2223.9063	1112.4568	2206.8798	1103.9435	2205.8958	1103.4515	19
7	***772.3043***	386.6558	755.2777	378.1425	754.2937	377.6505	C	2166.8849	1083.9461	2149.8583	1075.4328	2148.8743	1074.9408	18
8	***871.3727***	436.1900	854.3461	427.6767	853.3621	427.1847	V	2006.8542	1003.9308	1989.8277	995.4175	1988.8437	994.9255	17
9	***986.3996***	493.7034	969.3731	485.1902	968.3891	484.6982	D	***1907.7858***	954.3965	1890.7593	945.8833	1889.7753	945.3913	16
10	***1115.4422***	**558.2247**	1098.4157	549.7115	1097.4316	549.2195	E	***1792.7589***	896.8831	1775.7323	888.3698	1774.7483	887.8778	15
11	1229.4851	615.2462	1212.4586	606.7329	1211.4746	606.2409	N	***1663.7163***	832.3618	1646.6897	823.8485	1645.7057	823.3565	14
12	***1358.5277***	679.7675	1341.5012	671.2542	1340.5172	670.7622	E	***1549.6734***	775.3403	1532.6468	766.8270	1531.6628	766.3350	13
13	1518.5584	759.7828	1501.5318	751.2696	1500.5478	750.7775	C	***1420.6308***	710.8190	1403.6042	702.3057	1402.6202	701.8137	12
14	1646.6170	823.8121	1629.5904	815.2988	1628.6064	814.8068	Q	***1260.6001***	630.8037	1243.5736	622.2904	1242.5895	621.7984	11
15	1747.6646	874.3360	1730.6381	865.8227	1729.6541	865.3307	T	***1132.5415***	566.7744	1115.5150	558.2611	1114.5310	557.7691	10
16	1875.7596	938.3834	1858.7331	929.8702	1857.7490	929.3782	K	***1031.4939***	516.2506	1014.4673	507.7373	1013.4833	507.2453	9
17	1972.8124	986.9098	1955.7858	978.3965	1954.8018	977.9045	P	***903.3989***	452.2031	886.3723	443.6898	885.3883	443.1978	8
18	2029.8338	1015.4206	2012.8073	1006.9073	2011.8233	1006.4153	G	806.3461	403.6767	789.3196	395.1634	788.3356	394.6714	7

**19**	2142.9179	1071.9626	2125.8913	1063.4493	2124.9073	1062.9573	**I**	749.3247	375.1660	732.2981	366.6527	731.3141	366.1607	**6**
**20**	2302.9485	1151.9779	2285.9220	1143.4646	2284.9380	1142.9726	**C**	***636.2406***	318.6239	619.2141	310.1107	618.2300	309.6187	**5**
**21**	2431.9911	1216.4992	2414.9646	1207.9859	2413.9806	1207.4939	**E**	476.2100	238.6086	459.1834	230.0953	458.1994	229.6033	**4**
**22**	2547.0181	1274.0127	2529.9915	1265.4994	2529.0075	1265.0074	**N**	***347.1674***	174.0873	330.1408	165.5740			**3**
**23**	2604.0395	1302.5234	2587.0130	1294.0101	2586.0290	1293.5181	**G**	***232.1404***	116.5738	215.1139	108.0606			**2**
**24**							**R**	175.1190	88.0631	158.0924	79.5498			**1**

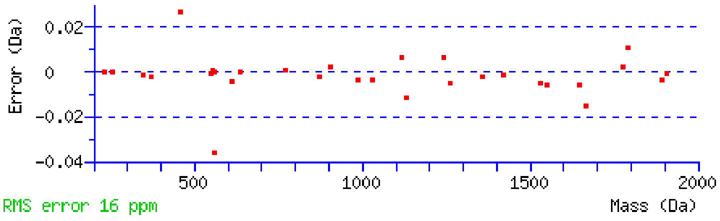

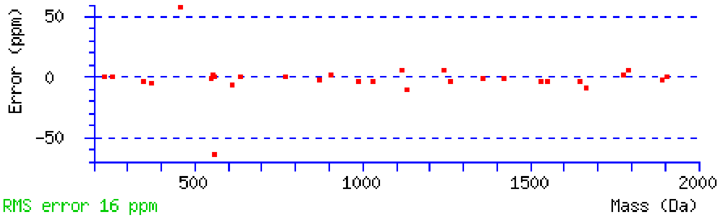

NCBI **BLAST** search of RPDGEGCVDENECQTKPGICENGR
(Parameters: blastp, nr protein database, expect=20000, no filter, PAM30)
Other BLAST web gateways

All matches to this query

Score	Mr(calc)	Delta	Sequence	Site Analysis
92.4	2777.1439	-0.0022	RPDGEGCVDENECQTKPGICENGR	Deamidated N22 99.95%
59.2	2777.1439	-0.0022	RPDGEGCVDENECQTKPGICENGR	Deamidated Q14 0.05%
34.8	2777.1439	-0.0022	RPDGEGCVDENECQTKPGICENGR	Deamidated N11 0.00%

Mascot: http://www.matrixscience.com/

Mascot Search Results

Peptide View

MS/MS Fragmentation of **RPDGEGCVDENECQTKPGICENGR**
Found in **P35555** in **uni_human_nr**, FBN1_HUMAN Fibrillin-1 OS=Homo sapiens GN=FBN1 PE=1 SV=3

Match to Query 11982: 2777.139856 from(695.292240,4+) intensity(230531.3281) rtinseconds(1212) scans(5250) index(3074)
Title: 130801_HPL_Human_Plaque_BR1_TR1_17_Spectrum023238_scans__5250_RTINSECONDS=1212
Data file E:\\RAW\\HumanPlaques\\mgf\\T\\1D\\T130801_HPL_Human_Plaque_BR1_TR1_17.mgf

Click mouse within plot area to zoom in by factor of two about that point
Or, [Plot from] [0] to [2000] Da [Full range]
Label all possible matches ○ Label matches used for scoring ⊙
Show Y-axis ☐

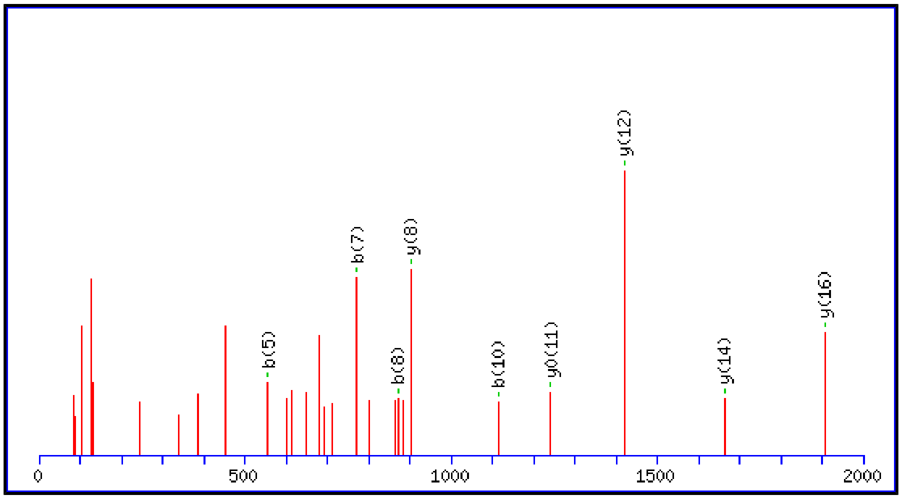

Monoisotopic mass of neutral peptide Mr(calc): 2777.1439
Fixed modifications: Carbamidomethyl (C) (apply to specified residues or termini only)
Variable modifications:
Q14 : Deamidated (NQ)
Ions Score: 24 **Expect:** 0.037
Matches : 9/266 fragment ions using 14 most intense peaks (help)

#	b	b^{++}	b*	b^{*++}	b^0	b^{0++}	Seq.	y	y^{++}	y*	y^{*++}	y^0	y^{0++}	#
1	157.1084	79.0578	140.0818	70.5446			R							24
2	254.1612	127.5842	237.1346	119.0709			P	2622.0501	1311.5287	2605.0236	1303.0154	2604.0395	1302.5234	23
3	369.1881	185.0977	352.1615	176.5844	351.1775	176.0924	D	2524.9973	1263.0023	2507.9708	1254.4890	2506.9868	1253.9970	22
4	426.2096	213.6084	409.1830	205.0951	408.1990	204.6031	G	2409.9704	1205.4888	2392.9439	1196.9756	2391.9598	1196.4836	21
5	***555.2522***	278.1297	538.2256	269.6164	537.2416	269.1244	E	2352.9489	1176.9781	2335.9224	1168.4648	2334.9384	1167.9728	20
6	612.2736	306.6404	595.2471	298.1272	594.2631	297.6352	G	2223.9063	1112.4568	2206.8798	1103.9435	2205.8958	1103.4515	19
7	***772.3043***	386.6558	755.2777	378.1425	754.2937	377.6505	C	2166.8849	1083.9461	2149.8583	1075.4328	2148.8743	1074.9408	18
8	***871.3727***	436.1900	854.3461	427.6767	853.3621	427.1847	V	2006.8542	1003.9308	1989.8277	995.4175	1988.8437	994.9255	17
9	986.3996	493.7034	969.3731	485.1902	968.3891	484.6982	D	***1907.7858***	954.3965	1890.7593	945.8833	1889.7753	945.3913	16
10	***1115.4422***	558.2247	1098.4157	549.7115	1097.4316	549.2195	E	1792.7589	896.8831	1775.7323	888.3698	1774.7483	887.8778	15
11	1229.4851	615.2462	1212.4586	606.7329	1211.4746	606.2409	N	***1663.7163***	832.3618	1646.6897	823.8485	1645.7057	823.3565	14
12	1358.5277	679.7675	1341.5012	671.2542	1340.5172	670.7622	E	1549.6734	775.3403	1532.6468	766.8270	1531.6628	766.3350	13
13	1518.5584	759.7828	1501.5318	751.2696	1500.5478	750.7775	C	***1420.6308***	710.8190	1403.6042	702.3057	1402.6202	701.8137	12
14	1647.6010	824.3041	1630.5744	815.7909	1629.5904	815.2988	Q	1260.6001	630.8037	1243.5736	622.2904	1242.5895	621.7984	11
15	1748.6487	874.8280	1731.6221	866.3147	1730.6381	865.8227	T	1131.5575	566.2824	1114.5310	557.7691	1113.5470	557.2771	10
16	1876.7436	938.8754	1859.7171	930.3622	1858.7331	929.8702	K	1030.5098	515.7586	1013.4833	507.2453	1012.4993	506.7533	9
17	1973.7964	987.4018	1956.7698	978.8886	1955.7858	978.3965	P	***902.4149***	451.7111	885.3883	443.1978	884.4043	442.7058	8
18	2030.8178	1015.9126	2013.7913	1007.3993	2012.8073	1006.9073	G	805.3621	403.1847	788.3356	394.6714	787.3515	394.1794	7

**19**	2143.9019	1072.4546	2126.8754	1063.9413	2125.8913	1063.4493	**I**	748.3406	374.6740	731.3141	366.1607	730.3301	365.6687	**6**
**20**	2303.9326	1152.4699	2286.9060	1143.9566	2285.9220	1143.4646	**C**	635.2566	318.1319	618.2300	309.6187	617.2460	309.1266	**5**
**21**	2432.9752	1216.9912	2415.9486	1208.4779	2414.9646	1207.9859	**E**	475.2259	238.1166	458.1994	229.6033	457.2154	229.1113	**4**
**22**	2547.0181	1274.0127	2529.9915	1265.4994	2529.0075	1265.0074	**N**	346.1833	173.5953	329.1568	165.0820			**3**
**23**	2604.0395	1302.5234	2587.0130	1294.0101	2586.0290	1293.5181	**G**	232.1404	116.5738	215.1139	108.0606			**2**
**24**							**R**	175.1190	88.0631	158.0924	79.5498			**1**

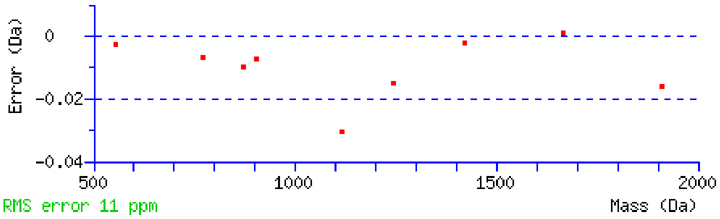

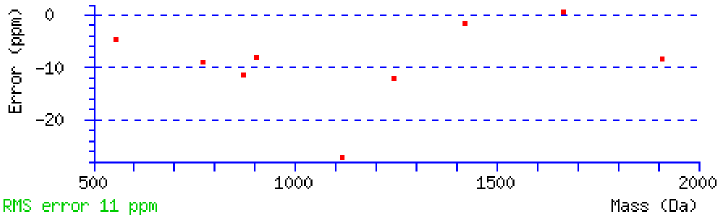

NCBI **BLAST** search of RPDGEGCVDENECQTKPGICENGR
(Parameters: blastp, nr protein database, expect=20000, no filter, PAM30)
Other BLAST web gateways

All matches to this query

Score	Mr(calc)	Delta	Sequence	Site Analysis
24.3	2777.1439	-0.0041	RPDGEGCVDENECQTKPGICENGR	Deamidated Q14 60.49%
19.8	2777.1439	-0.0041	RPDGEGCVDENECQTKPGICENGR	Deamidated N22 21.41%
19.1	2777.1439	-0.0041	RPDGEGCVDENECQTKPGICENGR	Deamidated N11 18.10%

Mascot: http://www.matrixscience.com/

Mascot Search Results

Peptide View

MS/MS Fragmentation of **RPDGEGCVDENECQTKPGICENGR**
Found in **P35555** in **uni_human_nr**, FBN1_HUMAN Fibrillin-1 OS=Homo sapiens GN=FBN1 PE=1 SV=3

Match to Query 11313: 2776.160096 from(695.047300,4+) intensity(4720271.5000) rtinseconds(1145) scans(4623) index(2386)
Title: 130801_HPL_Human_Plaque_BR1_TR1_15_Spectrum022539_scans__4623_RTINSECONDS=1145
Data file E:\\RAW\\HumanPlaques\\mgf\\T\\1D\\T130801_HPL_Human_Plaque_BR1_TR1_15.mgf

Click mouse within plot area to zoom in by factor of two about that point
Or, [Plot from] [0] to [2800] Da [Full range]
Label all possible matches ○ Label matches used for scoring ⊙
Show Y-axis ☐

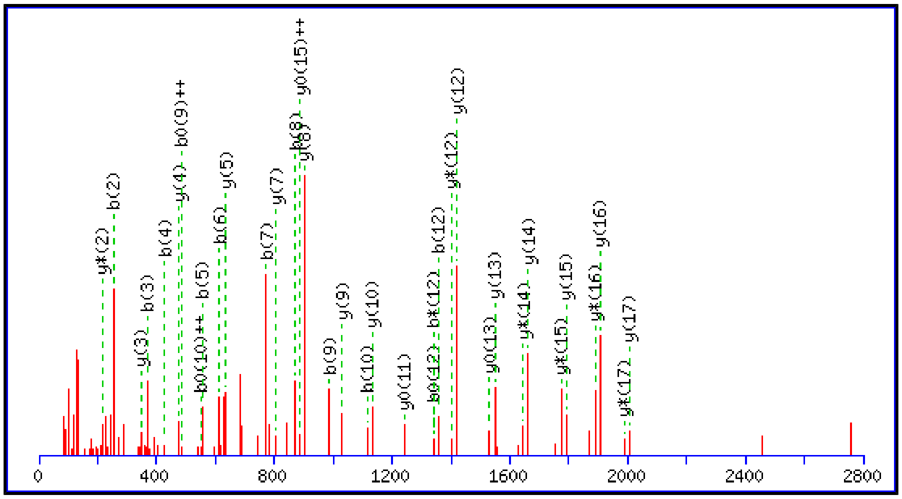

Monoisotopic mass of neutral peptide Mr(calc): 2776.1599
Fixed modifications: Carbamidomethyl (C) (apply to specified residues or termini only)
Ions Score: 94 **Expect:** 5.7e-009
Matches : 36/266 fragment ions using 66 most intense peaks (help)

#	b	b^{++}	b*	b^{*++}	b^0	b^{0++}	Seq.	y	y^{++}	y*	y^{*++}	y^0	y^{0++}	#
1	157.1084	79.0578	140.0818	70.5446			R							24
2	***254.1612***	127.5842	237.1346	119.0709			P	2621.0661	1311.0367	2604.0395	1302.5234	2603.0555	1302.0314	23
3	***369.1881***	185.0977	352.1615	176.5844	351.1775	176.0924	D	2524.0133	1262.5103	2506.9868	1253.9970	2506.0028	1253.5050	22
4	***426.2096***	213.6084	409.1830	205.0951	408.1990	204.6031	G	2408.9864	1204.9968	2391.9598	1196.4836	2390.9758	1195.9915	21
5	***555.2522***	278.1297	538.2256	269.6164	537.2416	269.1244	E	2351.9649	1176.4861	2334.9384	1167.9728	2333.9544	1167.4808	20
6	***612.2736***	306.6404	595.2471	298.1272	594.2631	297.6352	G	2222.9223	1111.9648	2205.8958	1103.4515	2204.9118	1102.9595	19
7	***772.3043***	386.6558	755.2777	378.1425	754.2937	377.6505	C	2165.9009	1083.4541	2148.8743	1074.9408	2147.8903	1074.4488	18
8	***871.3727***	436.1900	854.3461	427.6767	853.3621	427.1847	V	***2005.8702***	1003.4387	1988.8437	994.9255	1987.8597	994.4335	17
9	***986.3996***	493.7034	969.3731	485.1902	968.3891	484.6982	D	***1906.8018***	953.9045	1889.7753	945.3913	1888.7912	944.8993	16
10	***1115.4422***	558.2247	1098.4157	549.7115	1097.4316	549.2195	E	***1791.7749***	896.3911	1774.7483	887.8778	1773.7643	887.3858	15
11	1229.4851	615.2462	1212.4586	606.7329	1211.4746	606.2409	N	***1662.7323***	831.8698	1645.7057	823.3565	1644.7217	822.8645	14
12	***1358.5277***	679.7675	1341.5012	671.2542	1340.5172	670.7622	E	***1548.6893***	774.8483	1531.6628	766.3350	1530.6788	765.8430	13
13	1518.5584	759.7828	1501.5318	751.2696	1500.5478	750.7775	C	***1419.6467***	710.3270	1402.6202	701.8137	1401.6362	701.3217	12
14	1646.6170	823.8121	1629.5904	815.2988	1628.6064	814.8068	Q	1259.6161	630.3117	1242.5895	621.7984	1241.6055	621.3064	11
15	1747.6646	874.3360	1730.6381	865.8227	1729.6541	865.3307	T	***1131.5575***	566.2824	1114.5310	557.7691	1113.5470	557.2771	10
16	1875.7596	938.3834	1858.7331	929.8702	1857.7490	929.3782	K	***1030.5098***	515.7586	1013.4833	507.2453	1012.4993	506.7533	9
17	1972.8124	986.9098	1955.7858	978.3965	1954.8018	977.9045	P	***902.4149***	451.7111	885.3883	443.1978	884.4043	442.7058	8
18	2029.8338	1015.4206	2012.8073	1006.9073	2011.8233	1006.4153	G	***805.3621***	403.1847	788.3356	394.6714	787.3515	394.1794	7
19	2142.9179	1071.9626	2125.8913	1063.4493	2124.9073	1062.9573	I	748.3406	374.6740	731.3141	366.1607	730.3301	365.6687	6

20	2302.9485	1151.9779	2285.9220	1143.4646	2284.9380	1142.9726	C	***635.2566***	318.1319	618.2300	309.6187	617.2460	309.1266	5
21	2431.9911	1216.4992	2414.9646	1207.9859	2413.9806	1207.4939	E	***475.2259***	238.1166	458.1994	229.6033	457.2154	229.1113	4
22	2546.0341	1273.5207	2529.0075	1265.0074	2528.0235	1264.5154	N	***346.1833***	173.5953	329.1568	165.0820			3
23	2603.0555	1302.0314	2586.0290	1293.5181	2585.0450	1293.0261	G	232.1404	116.5738	215.1139	108.0606			2
24							R	175.1190	88.0631	158.0924	79.5498			1

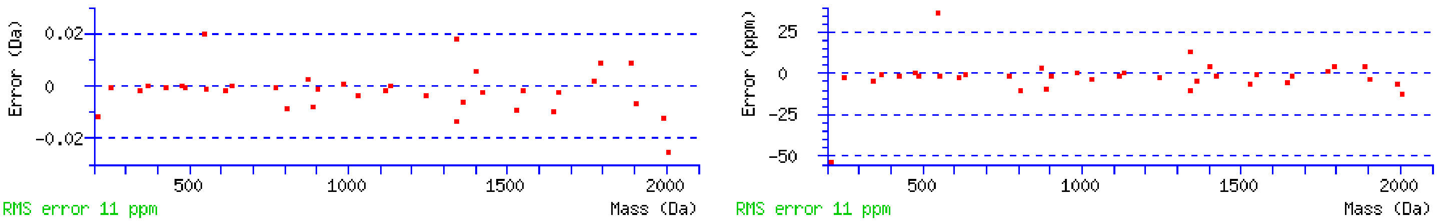

NCBI **BLAST** search of RPDGEGCVDENECQTKPGICENGR
(Parameters: blastp, nr protein database, expect=20000, no filter, PAM30)
Other BLAST web gateways

All matches to this query

Score	Mr(calc)	Delta	Sequence
94.1	2776.1599	0.0002	RPDGEGCVDENECQTKPGICENGR

Mascot: http://www.matrixscience.com/

Mascot Search Results

Peptide View

MS/MS Fragmentation of **RPDGEGCVDENECQTKPGICENGR**
Found in **P35555** in **uni_human_nr**, FBN1_HUMAN Fibrillin-1 OS=Homo sapiens GN=FBN1 PE=1 SV=3

Match to Query 11462: 2776.158282 from(926.393370,3+) intensity(3562521.0000) rtinseconds(1131) scans(4585) index(2399)
Title: 130801_HPL_Human_Plaque_BR1_TR1_14_Spectrum022900_scans__4585_RTINSECONDS=1131
Data file E:\\RAW\\HumanPlaques\\mgf\\T\\1D\\T130801_HPL_Human_Plaque_BR1_TR1_14.mgf

Click mouse within plot area to zoom in by factor of two about that point
Or, Plot from 0 to 2800 Da Full range
Label all possible matches ○ Label matches used for scoring ⊙
Show Y-axis ☐

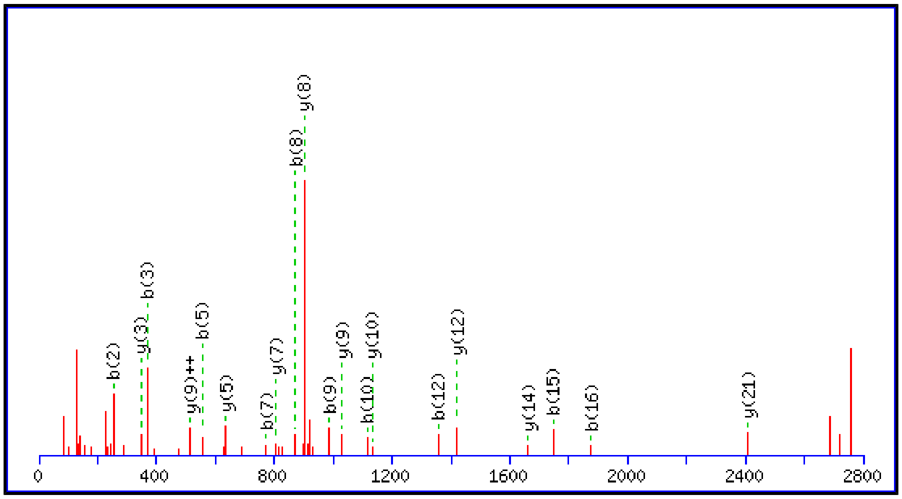

Monoisotopic mass of neutral peptide Mr(calc): 2776.1599
Fixed modifications: Carbamidomethyl (C) (apply to specified residues or termini only)
Ions Score: 81 **Expect:** 1.1e-007
Matches : 20/266 fragment ions using 31 most intense peaks (help)

#	b	b^{++}	b*	b^{*++}	b^0	b^{0++}	Seq.	y	y^{++}	y*	y^{*++}	y^0	y^{0++}	#
1	157.1084	79.0578	140.0818	70.5446			R							24
2	*254.1612*	127.5842	237.1346	119.0709			P	2621.0661	1311.0367	2604.0395	1302.5234	2603.0555	1302.0314	23
3	*369.1881*	185.0977	352.1615	176.5844	351.1775	176.0924	D	2524.0133	1262.5103	2506.9868	1253.9970	2506.0028	1253.5050	22
4	426.2096	213.6084	409.1830	205.0951	408.1990	204.6031	G	*2408.9864*	1204.9968	2391.9598	1196.4836	2390.9758	1195.9915	21
5	*555.2522*	278.1297	538.2256	269.6164	537.2416	269.1244	E	2351.9649	1176.4861	2334.9384	1167.9728	2333.9544	1167.4808	20
6	612.2736	306.6404	595.2471	298.1272	594.2631	297.6352	G	2222.9223	1111.9648	2205.8958	1103.4515	2204.9118	1102.9595	19
7	*772.3043*	386.6558	755.2777	378.1425	754.2937	377.6505	C	2165.9009	1083.4541	2148.8743	1074.9408	2147.8903	1074.4488	18
8	*871.3727*	436.1900	854.3461	427.6767	853.3621	427.1847	V	2005.8702	1003.4387	1988.8437	994.9255	1987.8597	994.4335	17
9	*986.3996*	493.7034	969.3731	485.1902	968.3891	484.6982	D	1906.8018	953.9045	1889.7753	945.3913	1888.7912	944.8993	16
10	*1115.4422*	558.2247	1098.4157	549.7115	1097.4316	549.2195	E	1791.7749	896.3911	1774.7483	887.8778	1773.7643	887.3858	15
11	1229.4851	615.2462	1212.4586	606.7329	1211.4746	606.2409	N	*1662.7323*	831.8698	1645.7057	823.3565	1644.7217	822.8645	14
12	*1358.5277*	679.7675	1341.5012	671.2542	1340.5172	670.7622	E	1548.6893	774.8483	1531.6628	766.3350	1530.6788	765.8430	13
13	1518.5584	759.7828	1501.5318	751.2696	1500.5478	750.7775	C	*1419.6467*	710.3270	1402.6202	701.8137	1401.6362	701.3217	12
14	1646.6170	823.8121	1629.5904	815.2988	1628.6064	814.8068	Q	1259.6161	630.3117	1242.5895	621.7984	1241.6055	621.3064	11
15	*1747.6646*	874.3360	1730.6381	865.8227	1729.6541	865.3307	T	*1131.5575*	566.2824	1114.5310	557.7691	1113.5470	557.2771	10
16	*1875.7596*	938.3834	1858.7331	929.8702	1857.7490	929.3782	K	*1030.5098*	515.7586	1013.4833	507.2453	1012.4993	506.7533	9
17	1972.8124	986.9098	1955.7858	978.3965	1954.8018	977.9045	P	*902.4149*	451.7111	885.3883	443.1978	884.4043	442.7058	8
18	2029.8338	1015.4206	2012.8073	1006.9073	2011.8233	1006.4153	G	*805.3621*	403.1847	788.3356	394.6714	787.3515	394.1794	7
19	2142.9179	1071.9626	2125.8913	1063.4493	2124.9073	1062.9573	I	748.3406	374.6740	731.3141	366.1607	730.3301	365.6687	6

**20**	2302.9485	1151.9779	2285.9220	1143.4646	2284.9380	1142.9726	**C**	***635.2566***	318.1319	618.2300	309.6187	617.2460	309.1266	**5**
**21**	2431.9911	1216.4992	2414.9646	1207.9859	2413.9806	1207.4939	**E**	475.2259	238.1166	458.1994	229.6033	457.2154	229.1113	**4**
**22**	2546.0341	1273.5207	2529.0075	1265.0074	2528.0235	1264.5154	**N**	***346.1833***	173.5953	329.1568	165.0820			**3**
**23**	2603.0555	1302.0314	2586.0290	1293.5181	2585.0450	1293.0261	**G**	232.1404	116.5738	215.1139	108.0606			**2**
**24**							**R**	175.1190	88.0631	158.0924	79.5498			**1**

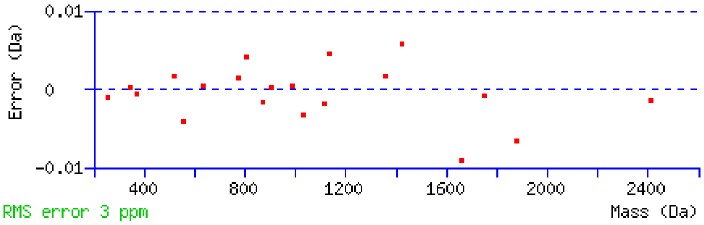

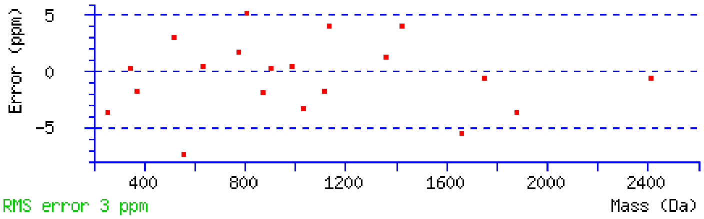

NCBI **BLAST** search of RPDGEGCVDENECQTKPGICENGR
(Parameters: blastp, nr protein database, expect=20000, no filter, PAM30)
Other BLAST web gateways

All matches to this query

Score	Mr(calc)	Delta	Sequence
81.3	2776.1599	-0.0016	RPDGEGCVDENECQTKPGICENGR

**Mascot:** http://www.matrixscience.com/

Mascot Search Results

Peptide View

MS/MS Fragmentation of **YCKDINECETPGICMNGR**
Found in **P35555** in **uni_human_nr**, FBN1_HUMAN Fibrillin-1 OS=Homo sapiens GN=FBN1 PE=1 SV=3

Match to Query 8810: 2233.881912 from(745.634580,3+) intensity(367468.1250) rtinseconds(1414) scans(6719) index(4490)
Title: 130806_HPL_Human_Plaque_BR2_TR2_12_Spectrum025243_scans__6719_RTINSECONDS=1414
Data file E:\\RAW\\HumanPlaques\\mgf\\T\\1D\\T130806_HPL_Human_Plaque_BR2_TR2_12.mgf

Click mouse within plot area to zoom in by factor of two about that point
Or, Plot from 0 to 1100 Da Full range
Label all possible matches ○ Label matches used for scoring ⊙
Show Y-axis ☐

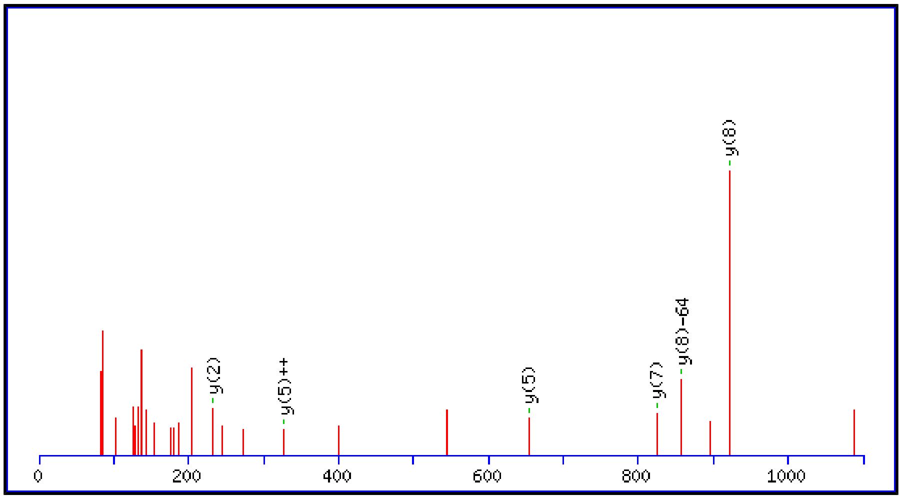

Monoisotopic mass of neutral peptide Mr(calc): 2233.8748
Fixed modifications: Carbamidomethyl (C) (apply to specified residues or termini only)
Variable modifications:
N6 : Deamidated (NQ)
M15 : Oxidation (M), with neutral losses 0.0000(shown in table), 63.9983
N16 : Deamidated (NQ)
Ions Score: 17 **Expect:** 0.099
Matches : 6/270 fragment ions using 13 most intense peaks (help)

#	b	b^{++}	b*	b^{*++}	b^0	b^{0++}	Seq.	y	y^{++}	y*	y^{*++}	y^0	y^{0++}	#
1	164.0706	82.5389					Y							18
2	324.1013	162.5543					C	2071.8188	1036.4130	2054.7922	1027.8998	2053.8082	1027.4077	17
3	452.1962	226.6017	435.1697	218.0885			K	1911.7881	956.3977	1894.7616	947.8844	1893.7776	947.3924	16
4	567.2232	284.1152	550.1966	275.6019	549.2126	275.1099	D	1783.6932	892.3502	1766.6666	883.8369	1765.6826	883.3449	15
5	680.3072	340.6573	663.2807	332.1440	662.2967	331.6520	I	1668.6662	834.8367	1651.6397	826.3235	1650.6557	825.8315	14
6	795.3342	398.1707	778.3076	389.6574	777.3236	389.1654	N	1555.5822	778.2947	1538.5556	769.7814	1537.5716	769.2894	13
7	924.3768	462.6920	907.3502	454.1787	906.3662	453.6867	E	1440.5552	720.7812	1423.5287	712.2680	1422.5446	711.7760	12
8	1084.4074	542.7073	1067.3809	534.1941	1066.3968	533.7021	C	1311.5126	656.2599	1294.4861	647.7467	1293.5021	647.2547	11
9	1213.4500	607.2286	1196.4235	598.7154	1195.4394	598.2234	E	1151.4820	576.2446	1134.4554	567.7313	1133.4714	567.2393	10
10	1314.4977	657.7525	1297.4711	649.2392	1296.4871	648.7472	T	1022.4394	511.7233	1005.4128	503.2101	1004.4288	502.7180	9
11	1411.5504	706.2789	1394.5239	697.7656	1393.5399	697.2736	P	***921.3917***	461.1995	904.3652	452.6862			8
12	1468.5719	734.7896	1451.5454	726.2763	1450.5613	725.7843	G	***824.3389***	412.6731	807.3124	404.1598			7
13	1581.6560	791.3316	1564.6294	782.8183	1563.6454	782.3263	I	767.3175	384.1624	750.2909	375.6491			6
14	1741.6866	871.3469	1724.6601	862.8337	1723.6761	862.3417	C	***654.2334***	327.6203	637.2069	319.1071			5
15	1888.7220	944.8646	1871.6955	936.3514	1870.7115	935.8594	M	494.2028	247.6050	477.1762	239.0917			4
16	2003.7490	1002.3781	1986.7224	993.8648	1985.7384	993.3728	N	347.1674	174.0873	330.1408	165.5740			3
17	2060.7704	1030.8889	2043.7439	1022.3756	2042.7599	1021.8836	G	***232.1404***	116.5738	215.1139	108.0606			2

**18**							**R**	175.1190	88.0631	158.0924	79.5498			**1**

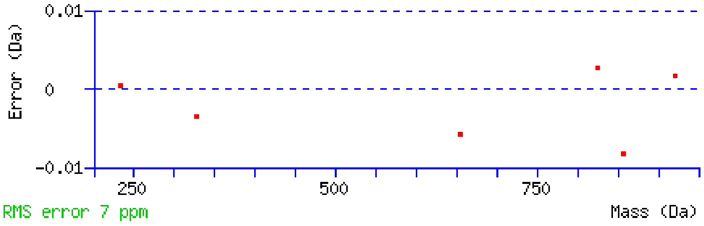

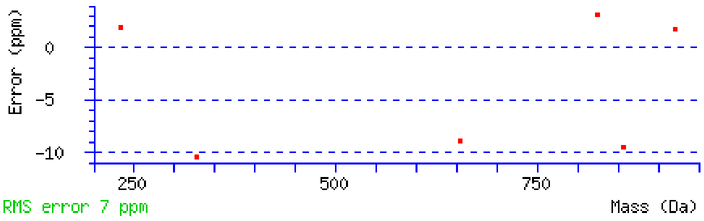

NCBI **BLAST** search of YCKDINECETPGICMNGR
(Parameters: blastp, nr protein database, expect=20000, no filter, PAM30)
Other BLAST web gateways

All matches to this query

Score	Mr(calc)	Delta	Sequence
16.8	2233.8748	0.0071	YCKDINECETPGICMNGR

Mascot: http://www.matrixscience.com/

Mascot Search Results

Peptide View

MS/MS Fragmentation of **YCKDINECETPGICMNGR**
Found in **P35555** in **uni_human_nr**, FBN1_HUMAN Fibrillin-1 OS=Homo sapiens GN=FBN1 PE=1 SV=3

Match to Query 8707: 2232.891132 from(745.304320,3+) intensity(1353067.5000) rtinseconds(1436) scans(6745) index(4439)
Title: 130809_HPL_Human_Plaque_BR1_TR3_15_Spectrum024826_scans__6745_RTINSECONDS=1436
Data file E:\\RAW\\HumanPlaques\\mgf\\T\\1D\\T130809_HPL_Human_Plaque_BR1_TR3_15.mgf

Click mouse within plot area to zoom in by factor of two about that point
Or, [Plot from] [0] to [2400] Da [Full range]
Label all possible matches ○ Label matches used for scoring ⦿
Show Y-axis ☐

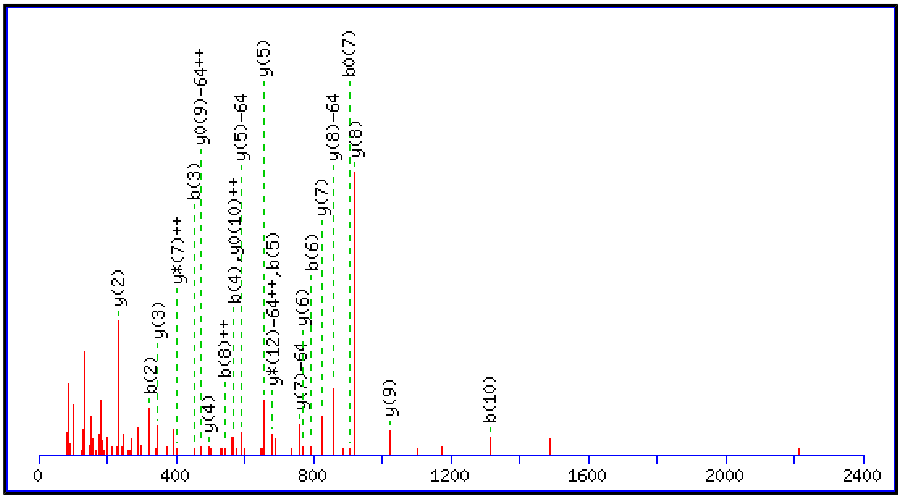

Monoisotopic mass of neutral peptide Mr(calc): 2232.8908
Fixed modifications: Carbamidomethyl (C) (apply to specified residues or termini only)
Variable modifications:
M15 : Oxidation (M), with neutral losses 0.0000(shown in table), 63.9983
N16 : Deamidated (NQ)
Ions Score: 53 **Expect:** 3.6e-005
Matches : 23/270 fragment ions using 44 most intense peaks (help)

#	b	b^{++}	b*	b^{*++}	b^0	b^{0++}	Seq.	y	y^{++}	y*	y^{*++}	y^0	y^{0++}	#
1	164.0706	82.5389					Y							18
2	324.1013	162.5543					C	2070.8348	1035.9210	2053.8082	1027.4077	2052.8242	1026.9157	17
3	452.1962	226.6017	435.1697	218.0885			K	1910.8041	955.9057	1893.7776	947.3924	1892.7935	946.9004	16
4	567.2232	284.1152	550.1966	275.6019	549.2126	275.1099	D	1782.7091	891.8582	1765.6826	883.3449	1764.6986	882.8529	15
5	680.3072	340.6573	663.2807	332.1440	662.2967	331.6520	I	1667.6822	834.3447	1650.6557	825.8315	1649.6716	825.3395	14
6	794.3502	397.6787	777.3236	389.1654	776.3396	388.6734	N	1554.5981	777.8027	1537.5716	769.2894	1536.5876	768.7974	13
7	923.3927	462.2000	906.3662	453.6867	905.3822	453.1947	E	1440.5552	720.7812	1423.5287	712.2680	1422.5446	711.7760	12
8	1083.4234	542.2153	1066.3968	533.7021	1065.4128	533.2101	C	1311.5126	656.2599	1294.4861	647.7467	1293.5021	647.2547	11
9	1212.4660	606.7366	1195.4394	598.2234	1194.4554	597.7313	E	1151.4820	576.2446	1134.4554	567.7313	1133.4714	567.2393	10
10	1313.5137	657.2605	1296.4871	648.7472	1295.5031	648.2552	T	1022.4394	511.7233	1005.4128	503.2101	1004.4288	502.7180	9
11	1410.5664	705.7869	1393.5399	697.2736	1392.5559	696.7816	P	921.3917	461.1995	904.3652	452.6862			8
12	1467.5879	734.2976	1450.5613	725.7843	1449.5773	725.2923	G	824.3389	412.6731	807.3124	404.1598			7
13	1580.6720	790.8396	1563.6454	782.3263	1562.6614	781.8343	I	767.3175	384.1624	750.2909	375.6491			6
14	1740.7026	870.8549	1723.6761	862.3417	1722.6920	861.8497	C	654.2334	327.6203	637.2069	319.1071			5
15	1887.7380	944.3726	1870.7115	935.8594	1869.7274	935.3674	M	494.2028	247.6050	477.1762	239.0917			4
16	2002.7649	1001.8861	1985.7384	993.3728	1984.7544	992.8808	N	347.1674	174.0873	330.1408	165.5740			3
17	2059.7864	1030.3968	2042.7599	1021.8836	2041.7758	1021.3916	G	232.1404	116.5738	215.1139	108.0606			2

18							R	175.1190	88.0631	158.0924	79.5498			1

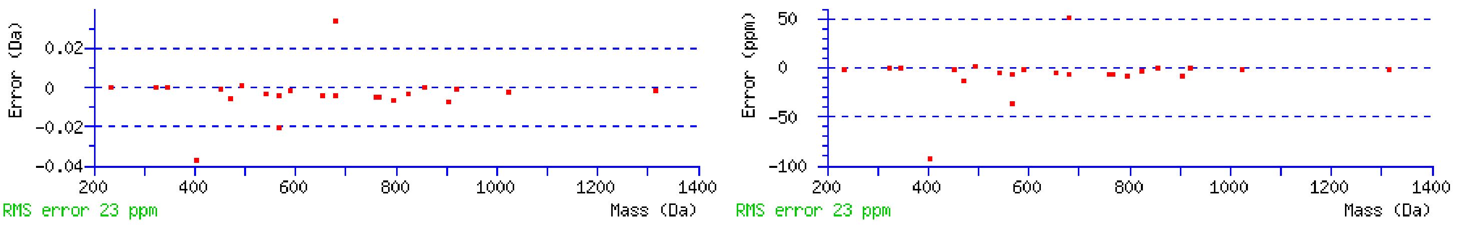

NCBI **BLAST** search of YCKDINECETPGICMNGR
(Parameters: blastp, nr protein database, expect=20000, no filter, PAM30)
Other BLAST web gateways

All matches to this query

Score	Mr(calc)	Delta	Sequence
53.1	2232.8908	0.0003	YCKDINECETPGICMNGR

Mascot: http://www.matrixscience.com/

Mascot Search Results

Peptide View

MS/MS Fragmentation of **YCKDINECETPGICMNGR**
Found in **P35555** in **uni_human_nr**, FBN1_HUMAN Fibrillin-1 OS=Homo sapiens GN=FBN1 PE=1 SV=3

Match to Query 10192: 2231.904372 from(744.975400,3+) intensity(3055518.7500) rtinseconds(1327) scans(6411) index(4299)
Title: 130806_HPL_Human_Plaque_BR1_TR2_07_Spectrum026270_scans__6411_RTINSECONDS=1327
Data file E:\\RAW\\HumanPlaques\\mgf\\T\\1D\\T130806_HPL_Human_Plaque_BR1_TR2_07.mgf

Click mouse within plot area to zoom in by factor of two about that point
Or, Plot from 0 to 2400 Da Full range
Label all possible matches ○ Label matches used for scoring ⊙
Show Y-axis ☐

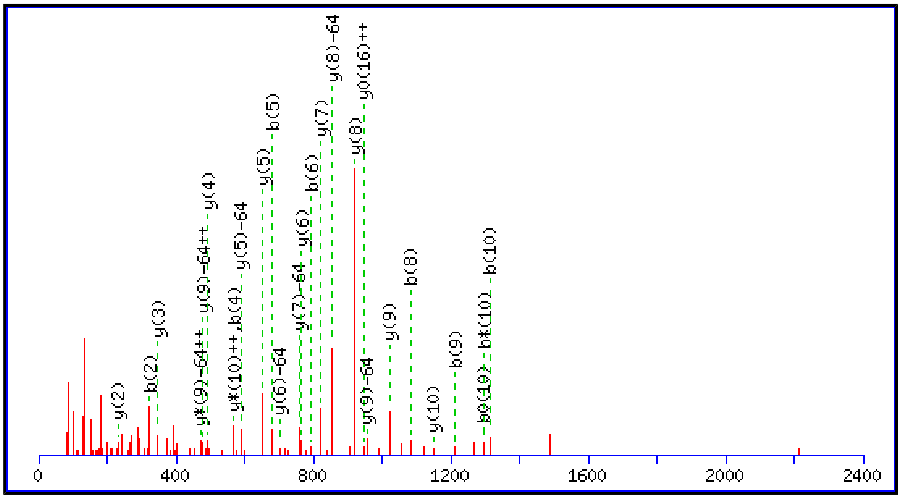

Monoisotopic mass of neutral peptide Mr(calc): 2231.9068
Fixed modifications: Carbamidomethyl (C) (apply to specified residues or termini only)
Variable modifications:
M15 : Oxidation (M), with neutral losses 0.0000(shown in table), 63.9983
Ions Score: 57 **Expect:** 1.8e-005
Matches : 27/270 fragment ions using 51 most intense peaks (help)

#	b	b^{++}	b*	b^{*++}	b^0	b^{0++}	Seq.	y	y^{++}	y*	y^{*++}	y^0	y^{0++}	#
1	164.0706	82.5389					Y							18
2	**324.1013**	162.5543					C	2069.8507	1035.4290	2052.8242	1026.9157	2051.8402	1026.4237	17
3	452.1962	226.6017	435.1697	218.0885			K	1909.8201	955.4137	1892.7935	946.9004	1891.8095	946.4084	16
4	**567.2232**	284.1152	550.1966	275.6019	549.2126	275.1099	D	1781.7251	891.3662	1764.6986	882.8529	1763.7146	882.3609	15
5	**680.3072**	340.6573	663.2807	332.1440	662.2967	331.6520	I	1666.6982	833.8527	1649.6716	825.3395	1648.6876	824.8474	14
6	**794.3502**	397.6787	777.3236	389.1654	776.3396	388.6734	N	1553.6141	777.3107	1536.5876	768.7974	1535.6036	768.3054	13
7	923.3927	462.2000	906.3662	453.6867	905.3822	453.1947	E	1439.5712	720.2892	1422.5446	711.7760	1421.5606	711.2840	12
8	**1083.4234**	542.2153	1066.3968	533.7021	1065.4128	533.2101	C	1310.5286	655.7679	1293.5021	647.2547	1292.5180	646.7627	11
9	**1212.4660**	606.7366	1195.4394	598.2234	1194.4554	597.7313	E	**1150.4980**	575.7526	1133.4714	567.2393	1132.4874	566.7473	10
10	**1313.5137**	657.2605	1296.4871	648.7472	1295.5031	648.2552	T	**1021.4554**	511.2313	1004.4288	502.7180	1003.4448	502.2260	9
11	1410.5664	705.7869	1393.5399	697.2736	1392.5559	696.7816	P	**920.4077**	460.7075	903.3811	452.1942			8
12	1467.5879	734.2976	1450.5613	725.7843	1449.5773	725.2923	G	**823.3549**	412.1811	806.3284	403.6678			7
13	1580.6720	790.8396	1563.6454	782.3263	1562.6614	781.8343	I	**766.3335**	383.6704	749.3069	375.1571			6
14	1740.7026	870.8549	1723.6761	862.3417	1722.6920	861.8497	C	**653.2494**	327.1283	636.2228	318.6151			5
15	1887.7380	944.3726	1870.7115	935.8594	1869.7274	935.3674	M	**493.2187**	247.1130	476.1922	238.5997			4
16	2001.7809	1001.3941	1984.7544	992.8808	1983.7704	992.3888	N	**346.1833**	173.5953	329.1568	165.0820			3
17	2058.8024	1029.9048	2041.7758	1021.3916	2040.7918	1020.8996	G	**232.1404**	116.5738	215.1139	108.0606			2
18							R	175.1190	88.0631	158.0924	79.5498			1

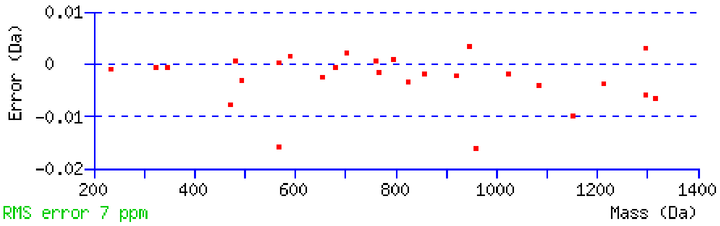

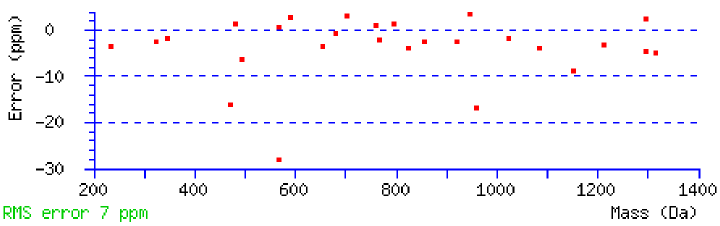

NCBI **BLAST** search of YCKDINECETPGICMNGR
(Parameters: blastp, nr protein database, expect=20000, no filter, PAM30)
Other BLAST web gateways

All matches to this query

Score	Mr(calc)	Delta	Sequence
57.2	2231.9068	-0.0024	YCKDINECETPGICMNGR

**Mascot:** http://www.matrixscience.com/

Mascot Search Results

Peptide View

MS/MS Fragmentation of **YCKDINECETPGICMNGR**
Found in **P35555** in **uni_human_nr**, FBN1_HUMAN Fibrillin-1 OS=Homo sapiens GN=FBN1 PE=1 SV=3

Match to Query 8119: 2216.897802 from(739.973210,3+) intensity(760192.0625) rtinseconds(1673) scans(8242) index(5654)
Title: 130801_HPL_Human_Plaque_BR2_TR1_13_Spectrum025983_scans__8242_RTINSECONDS=1673
Data file E:\\RAW\\HumanPlaques\\mgf\\T\\1D\\T130801_HPL_Human_Plaque_BR2_TR1_13.mgf

Click mouse within plot area to zoom in by factor of two about that point
Or, [Plot from] [0] to [1400] Da [Full range]
Label all possible matches ○ Label matches used for scoring ⊙
Show Y-axis ☐

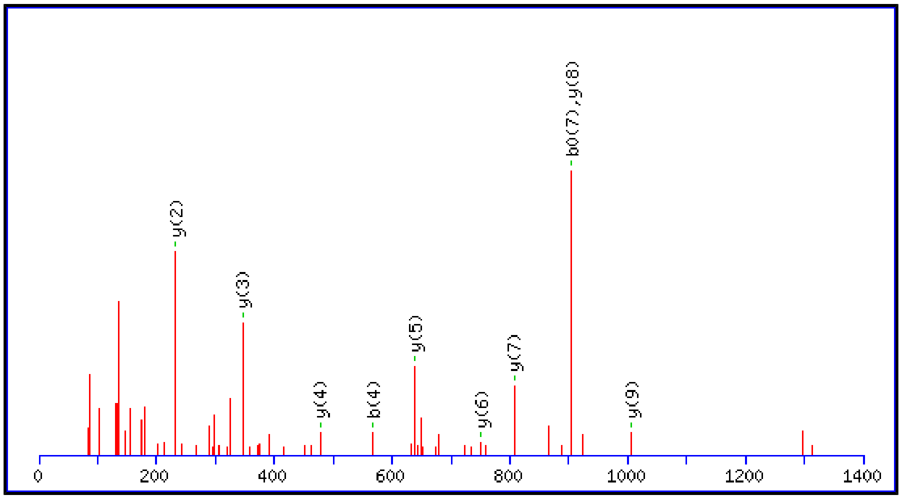

Monoisotopic mass of neutral peptide Mr(calc): 2216.8959
Fixed modifications: Carbamidomethyl (C) (apply to specified residues or termini only)
Variable modifications:
N16 : Deamidated (NQ)
Ions Score: 65 **Expect:** 2.9e-006
Matches : 10/178 fragment ions using 11 most intense peaks (help)

#	b	b^{++}	b*	b^{*++}	b^0	b^{0++}	Seq.	y	y^{++}	y*	y^{*++}	y^0	y^{0++}	#
1	164.0706	82.5389					Y							18
2	324.1013	162.5543					C	2054.8398	1027.9236	2037.8133	1019.4103	2036.8293	1018.9183	17
3	452.1962	226.6017	435.1697	218.0885			K	1894.8092	947.9082	1877.7826	939.3950	1876.7986	938.9030	16
4	**567.2232**	284.1152	550.1966	275.6019	549.2126	275.1099	D	1766.7142	883.8608	1749.6877	875.3475	1748.7037	874.8555	15
5	680.3072	340.6573	663.2807	332.1440	662.2967	331.6520	I	1651.6873	826.3473	1634.6607	817.8340	1633.6767	817.3420	14
6	794.3502	397.6787	777.3236	389.1654	776.3396	388.6734	N	1538.6032	769.8053	1521.5767	761.2920	1520.5927	760.8000	13
7	923.3927	462.2000	906.3662	453.6867	905.3822	453.1947	E	1424.5603	712.7838	1407.5338	704.2705	1406.5497	703.7785	12
8	1083.4234	542.2153	1066.3968	533.7021	1065.4128	533.2101	C	1295.5177	648.2625	1278.4912	639.7492	1277.5071	639.2572	11
9	1212.4660	606.7366	1195.4394	598.2234	1194.4554	597.7313	E	1135.4871	568.2472	1118.4605	559.7339	1117.4765	559.2419	10
10	1313.5137	657.2605	1296.4871	648.7472	1295.5031	648.2552	T	***1006.4445***	503.7259	989.4179	495.2126	988.4339	494.7206	9
11	1410.5664	705.7869	1393.5399	697.2736	1392.5559	696.7816	P	***905.3968***	453.2020	888.3702	444.6888			8
12	1467.5879	734.2976	1450.5613	725.7843	1449.5773	725.2923	G	***808.3440***	404.6756	791.3175	396.1624			7
13	1580.6720	790.8396	1563.6454	782.3263	1562.6614	781.8343	I	***751.3226***	376.1649	734.2960	367.6516			6
14	1740.7026	870.8549	1723.6761	862.3417	1722.6920	861.8497	C	***638.2385***	319.6229	621.2119	311.1096			5
15	1871.7431	936.3752	1854.7165	927.8619	1853.7325	927.3699	M	***478.2078***	239.6076	461.1813	231.0943			4
16	1986.7700	993.8887	1969.7435	985.3754	1968.7595	984.8834	N	***347.1674***	174.0873	330.1408	165.5740			3
17	2043.7915	1022.3994	2026.7649	1013.8861	2025.7809	1013.3941	G	***232.1404***	116.5738	215.1139	108.0606			2
18							R	175.1190	88.0631	158.0924	79.5498			1

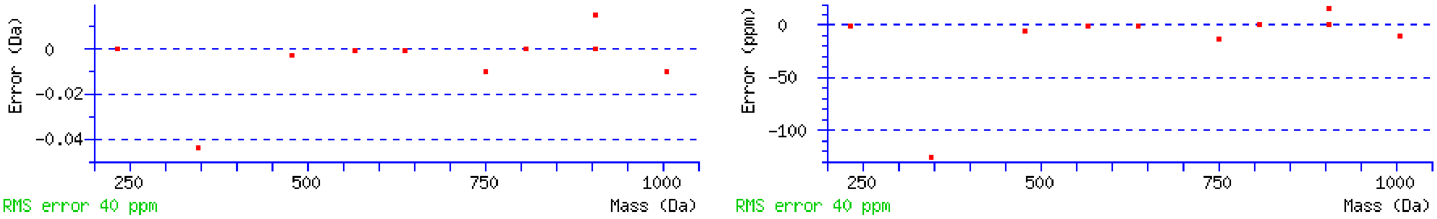

NCBI **BLAST** search of YCKDINECETPGICMNGR
(Parameters: blastp, nr protein database, expect=20000, no filter, PAM30)
Other BLAST web gateways

All matches to this query

Score	Mr(calc)	Delta	Sequence
64.6	2216.8959	0.0019	YCKDINECETPGICMNGR

Mascot: http://www.matrixscience.com/

Mascot Search Results

Peptide View

MS/MS Fragmentation of **YCKDINECETPGICMNGR**
Found in **P35555** in **uni_human_nr**, FBN1_HUMAN Fibrillin-1 OS=Homo sapiens GN=FBN1 PE=1 SV=3

Match to Query 9988: 2215.911042 from(739.644290,3+) intensity(12788484.0000) rtinseconds(1559) scans(8115) index(5743)
Title: 130801_HPL_Human_Plaque_BR1_TR1_06_Spectrum027788_scans__8115_RTINSECONDS=1559
Data file E:\\RAW\\HumanPlaques\\mgf\\T\\1D\\T130801_HPL_Human_Plaque_BR1_TR1_06.mgf

Click mouse within plot area to zoom in by factor of two about that point
Or, Plot from 0 to 1400 Da Full range
Label all possible matches ○ Label matches used for scoring ⊙
Show Y-axis ☐

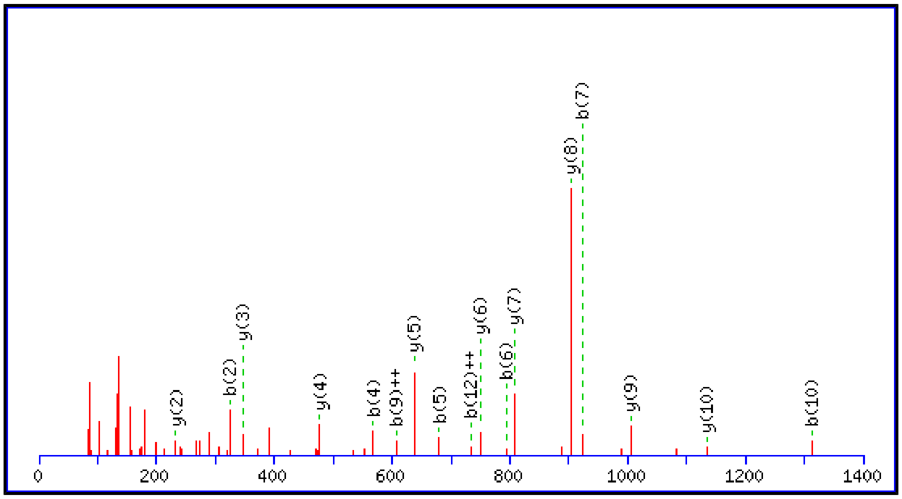

Monoisotopic mass of neutral peptide Mr(calc): 2215.9119
Fixed modifications: Carbamidomethyl (C) (apply to specified residues or termini only)
Ions Score: 69 **Expect:** 1.5e-006
Matches : 17/178 fragment ions using 32 most intense peaks (help)

#	b	b^{++}	b*	b^{*++}	b^0	b^{0++}	Seq.	y	y^{++}	y*	y^{*++}	y^0	y^{0++}	#
1	164.0706	82.5389					Y							18
2	***324.1013***	162.5543					C	2053.8558	1027.4316	2036.8293	1018.9183	2035.8453	1018.4263	17
3	452.1962	226.6017	435.1697	218.0885			K	1893.8252	947.4162	1876.7986	938.9030	1875.8146	938.4109	16
4	***567.2232***	284.1152	550.1966	275.6019	549.2126	275.1099	D	1765.7302	883.3687	1748.7037	874.8555	1747.7197	874.3635	15
5	***680.3072***	340.6573	663.2807	332.1440	662.2967	331.6520	I	1650.7033	825.8553	1633.6767	817.3420	1632.6927	816.8500	14
6	***794.3502***	397.6787	777.3236	389.1654	776.3396	388.6734	N	1537.6192	769.3132	1520.5927	760.8000	1519.6086	760.3080	13
7	***923.3927***	462.2000	906.3662	453.6867	905.3822	453.1947	E	1423.5763	712.2918	1406.5497	703.7785	1405.5657	703.2865	12
8	1083.4234	542.2153	1066.3968	533.7021	1065.4128	533.2101	C	1294.5337	647.7705	1277.5071	639.2572	1276.5231	638.7652	11
9	1212.4660	**606.7366**	1195.4394	598.2234	1194.4554	597.7313	E	***1134.5030***	567.7552	1117.4765	559.2419	1116.4925	558.7499	10
10	***1313.5137***	657.2605	1296.4871	648.7472	1295.5031	648.2552	T	***1005.4604***	503.2339	988.4339	494.7206	987.4499	494.2286	9
11	1410.5664	705.7869	1393.5399	697.2736	1392.5559	696.7816	P	***904.4128***	452.7100	887.3862	444.1967			8
12	1467.5879	**734.2976**	1450.5613	725.7843	1449.5773	725.2923	G	***807.3600***	404.1836	790.3335	395.6704			7
13	1580.6720	790.8396	1563.6454	782.3263	1562.6614	781.8343	I	***750.3385***	375.6729	733.3120	367.1596			6
14	1740.7026	870.8549	1723.6761	862.3417	1722.6920	861.8497	C	***637.2545***	319.1309	620.2279	310.6176			5
15	1871.7431	936.3752	1854.7165	927.8619	1853.7325	927.3699	M	***477.2238***	239.1156	460.1973	230.6023			4
16	1985.7860	993.3966	1968.7595	984.8834	1967.7755	984.3914	N	***346.1833***	173.5953	329.1568	165.0820			3
17	2042.8075	1021.9074	2025.7809	1013.3941	2024.7969	1012.9021	G	***232.1404***	116.5738	215.1139	108.0606			2
18							R	175.1190	88.0631	158.0924	79.5498			1

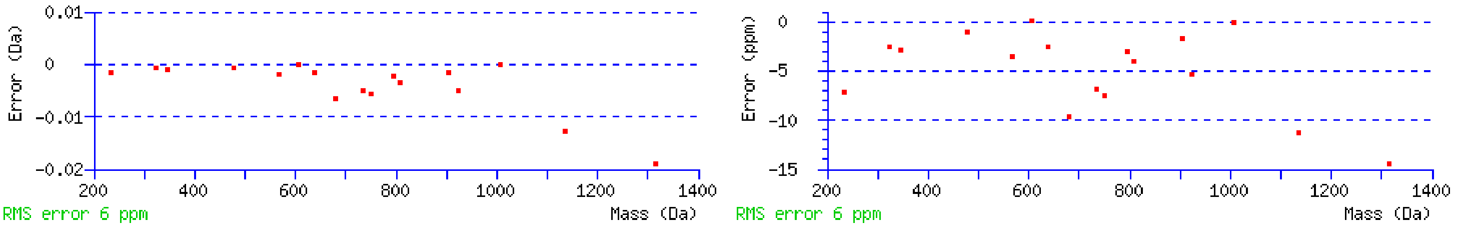

NCBI **BLAST** search of YCKDINECETPGICMNGR
(Parameters: blastp, nr protein database, expect=20000, no filter, PAM30)
Other BLAST web gateways

All matches to this query

Score	Mr(calc)	Delta	Sequence
68.8	2215.9119	-0.0008	YCKDINECETPGICMNGR

Mascot: http://www.matrixscience.com/

Mascot Search Results

Peptide View

MS/MS Fragmentation of **YCKDINECETPGICMNGR**
Found in **P35555** in **uni_human_nr**, FBN1_HUMAN Fibrillin-1 OS=Homo sapiens GN=FBN1 PE=1 SV=3

Match to Query 10404: 2215.910008 from(1108.962280,2+) intensity(894131.5625) rtinseconds(1585) scans(8500) index(6186)
Title: 130809_HPL_Human_Plaque_BR1_TR3_06_Spectrum028540_scans__8500_RTINSECONDS=1585
Data file E:\\RAW\\HumanPlaques\\mgf\\T\\1D\\T130809_HPL_Human_Plaque_BR1_TR3_06.mgf

Click mouse within plot area to zoom in by factor of two about that point
Or, [Plot from] [0] to [1200] Da [Full range]
Label all possible matches ○ Label matches used for scoring ⊙
Show Y-axis ☐

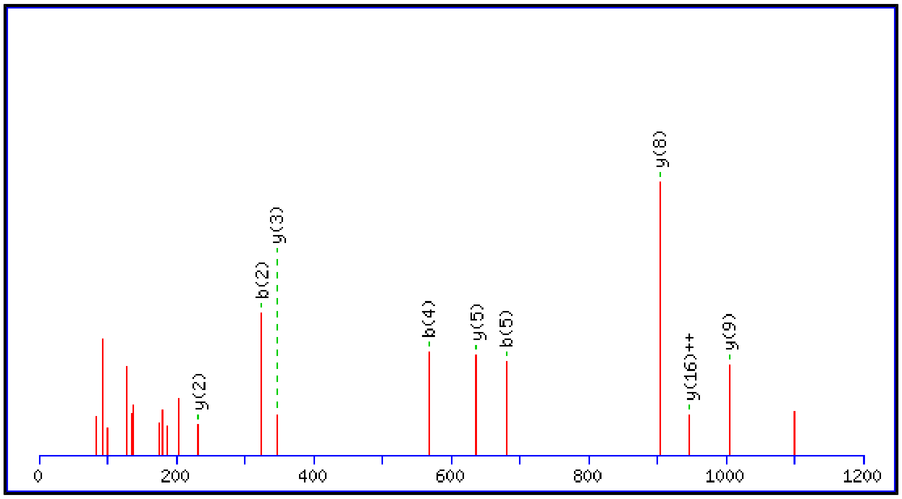

Monoisotopic mass of neutral peptide Mr(calc): 2215.9119
Fixed modifications: Carbamidomethyl (C) (apply to specified residues or termini only)
Ions Score: 39 **Expect:** 0.0014
Matches : 9/178 fragment ions using 13 most intense peaks (help)

#	b	b^{++}	b*	b^{*++}	b^0	b^{0++}	Seq.	y	y^{++}	y*	y^{*++}	y^0	y^{0++}	#
1	164.0706	82.5389					Y							18
2	***324.1013***	162.5543					C	2053.8558	1027.4316	2036.8293	1018.9183	2035.8453	1018.4263	17
3	452.1962	226.6017	435.1697	218.0885			K	1893.8252	947.4162	1876.7986	938.9030	1875.8146	938.4109	16
4	***567.2232***	284.1152	550.1966	275.6019	549.2126	275.1099	D	1765.7302	883.3687	1748.7037	874.8555	1747.7197	874.3635	15
5	***680.3072***	340.6573	663.2807	332.1440	662.2967	331.6520	I	1650.7033	825.8553	1633.6767	817.3420	1632.6927	816.8500	14
6	794.3502	397.6787	777.3236	389.1654	776.3396	388.6734	N	1537.6192	769.3132	1520.5927	760.8000	1519.6086	760.3080	13
7	923.3927	462.2000	906.3662	453.6867	905.3822	453.1947	E	1423.5763	712.2918	1406.5497	703.7785	1405.5657	703.2865	12
8	1083.4234	542.2153	1066.3968	533.7021	1065.4128	533.2101	C	1294.5337	647.7705	1277.5071	639.2572	1276.5231	638.7652	11
9	1212.4660	606.7366	1195.4394	598.2234	1194.4554	597.7313	E	1134.5030	567.7552	1117.4765	559.2419	1116.4925	558.7499	10
10	1313.5137	657.2605	1296.4871	648.7472	1295.5031	648.2552	T	***1005.4604***	503.2339	988.4339	494.7206	987.4499	494.2286	9
11	1410.5664	705.7869	1393.5399	697.2736	1392.5559	696.7816	P	***904.4128***	452.7100	887.3862	444.1967			8
12	1467.5879	734.2976	1450.5613	725.7843	1449.5773	725.2923	G	807.3600	404.1836	790.3335	395.6704			7
13	1580.6720	790.8396	1563.6454	782.3263	1562.6614	781.8343	I	750.3385	375.6729	733.3120	367.1596			6
14	1740.7026	870.8549	1723.6761	862.3417	1722.6920	861.8497	C	***637.2545***	319.1309	620.2279	310.6176			5
15	1871.7431	936.3752	1854.7165	927.8619	1853.7325	927.3699	M	477.2238	239.1156	460.1973	230.6023			4
16	1985.7860	993.3966	1968.7595	984.8834	1967.7755	984.3914	N	***346.1833***	173.5953	329.1568	165.0820			3
17	2042.8075	1021.9074	2025.7809	1013.3941	2024.7969	1012.9021	G	***232.1404***	116.5738	215.1139	108.0606			2
18							R	175.1190	88.0631	158.0924	79.5498			1

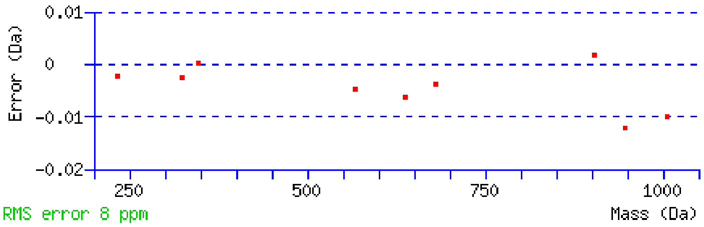

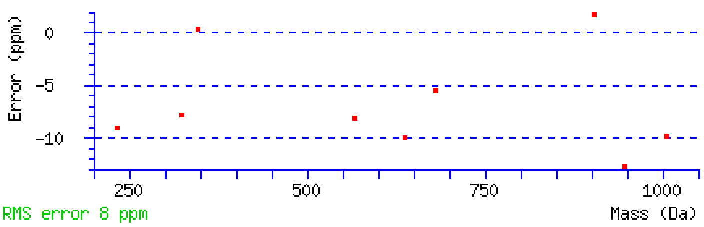

NCBI **BLAST** search of YCKDINECETPGICMNGR
(Parameters: blastp, nr protein database, expect=20000, no filter, PAM30)
Other BLAST web gateways

All matches to this query

Score	Mr(calc)	Delta	Sequence
38.9	2215.9119	-0.0019	YCKDINECETPGICMNGR

**Mascot:** http://www.matrixscience.com/

Mascot Search Results

Peptide View

MS/MS Fragmentation of **FAVLQENVAWGNGR**
Found in **Q9Y6R7** in **uni_human_nr**, FCGBP_HUMAN IgGFc-binding protein OS=Homo sapiens GN=FCGBP PE=1 SV=3

Match to Query 4303: 1561.775128 from(781.894840,2+) intensity(1496454.0000) rtinseconds(2498) scans(14607) index(11303)
Title: 130801_HPL_Human_Plaque_BR2_TR1_06_Spectrum032797_scans__14607_RTINSECONDS=2498
Data file E:\\RAW\\HumanPlaques\\mgf\\T\\1D\\T130801_HPL_Human_Plaque_BR2_TR1_06.mgf

Click mouse within plot area to zoom in by factor of two about that point
Or, Plot from 0 to 1500 Da Full range
Label all possible matches ○ Label matches used for scoring ⊙
Show Y-axis ☐

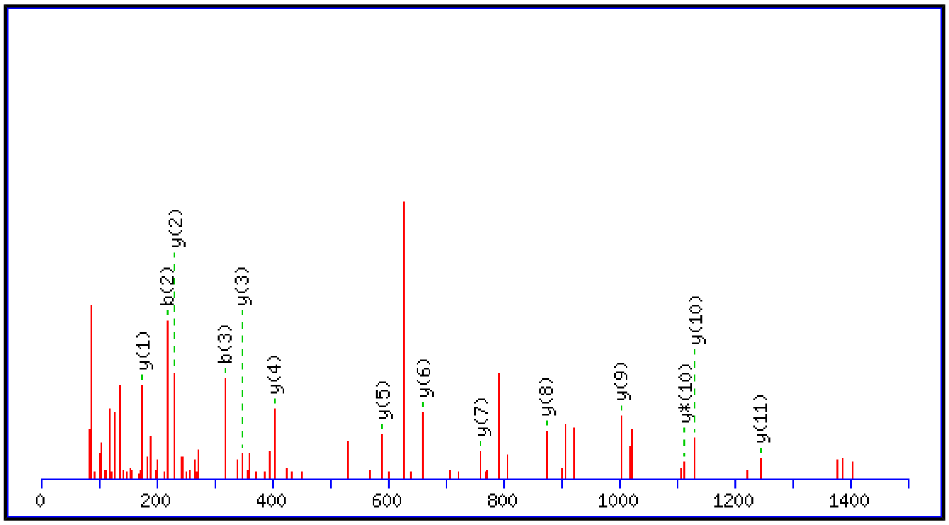

Monoisotopic mass of neutral peptide Mr(calc): 1560.7685
Fixed modifications: Carbamidomethyl (C) (apply to specified residues or termini only)
Variable modifications:
N12 : Deamidated (NQ)
Ions Score: 53 **Expect:** 0.00041
Matches : 14/122 fragment ions using 44 most intense peaks (help)

#	b	b^{++}	b*	b^{*++}	b^0	b^{0++}	Seq.	y	y^{++}	y*	y^{*++}	y^0	y^{0++}	#
1	148.0757	74.5415					F							14
2	**219.1128**	110.0600					A	1414.7074	707.8573	1397.6808	699.3440	1396.6968	698.8520	13
3	**318.1812**	159.5942					V	1343.6702	672.3388	1326.6437	663.8255	1325.6597	663.3335	12
4	431.2653	216.1363					L	***1244.6018***	622.8046	1227.5753	614.2913	1226.5913	613.7993	11
5	559.3239	280.1656	542.2973	271.6523			Q	***1131.5178***	566.2625	1114.4912	557.7492	1113.5072	557.2572	10
6	688.3665	344.6869	671.3399	336.1736	670.3559	335.6816	E	***1003.4592***	502.2332	986.4326	493.7200	985.4486	493.2279	9
7	802.4094	401.7083	785.3828	393.1951	784.3988	392.7030	N	***874.4166***	437.7119	857.3900	429.1987			8
8	901.4778	451.2425	884.4512	442.7293	883.4672	442.2373	V	***760.3737***	380.6905	743.3471	372.1772			7
9	972.5149	486.7611	955.4884	478.2478	954.5043	477.7558	A	***661.3053***	331.1563	644.2787	322.6430			6
10	1158.5942	579.8007	1141.5677	571.2875	1140.5837	570.7955	W	***590.2681***	295.6377	573.2416	287.1244			5
11	1215.6157	608.3115	1198.5891	599.7982	1197.6051	599.3062	G	***404.1888***	202.5980	387.1623	194.0848			4
12	1330.6426	665.8250	1313.6161	657.3117	1312.6321	656.8197	N	***347.1674***	174.0873	330.1408	165.5740			3
13	1387.6641	694.3357	1370.6375	685.8224	1369.6535	685.3304	G	***232.1404***	116.5738	215.1139	108.0606			2
14							R	***175.1190***	88.0631	158.0924	79.5498			1

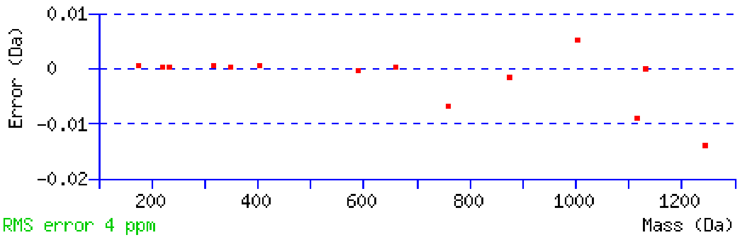

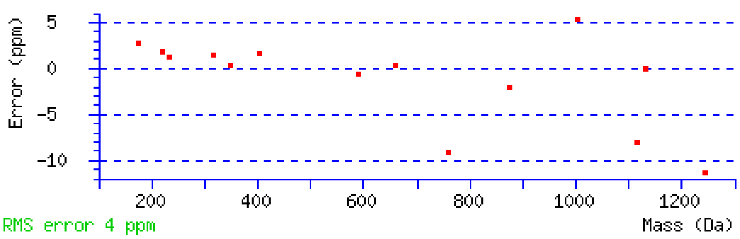

NCBI **BLAST** search of FAVLQENVAWGNGR
(Parameters: blastp, nr protein database, expect=20000, no filter, PAM30)
Other BLAST web gateways

All matches to this query

Score	Mr(calc)	Delta	Sequence	Site Analysis
52.8	1560.7685	1.0067	FAVLQENVAWGNGR	Deamidated N12 99.99%
13.3	1560.7685	1.0067	FAVLQENVAWGNGR	Deamidated N7 0.01%
3.8	1560.7685	1.0067	FAVLQENVAWGNGR	Deamidated Q5 0.00%

**Mascot:** http://www.matrixscience.com/

Mascot Search Results

Peptide View

MS/MS Fragmentation of **EDGGGWWYNRCHAANPNGR**
Found in **P02675** in **uni_human_nr**, FIBB_HUMAN Fibrinogen beta chain OS=Homo sapiens GN=FGB PE=1 SV=2

Match to Query 8994: 2217.906696 from(555.483950,4+) intensity(105656.2188) rtinseconds(1757) scans(8875) index(6210)
Title: 130806_HPL_Human_Plaque_BR2_TR2_15_Spectrum026327_scans__8875_RTINSECONDS=1757
Data file E:\\RAW\\HumanPlaques\\mgf\\T\\1D\\T130806_HPL_Human_Plaque_BR2_TR2_15.mgf

Click mouse within plot area to zoom in by factor of two about that point
Or, Plot from 0 to 1900 Da Full range
Label all possible matches ○ Label matches used for scoring ⊙
Show Y-axis ☐

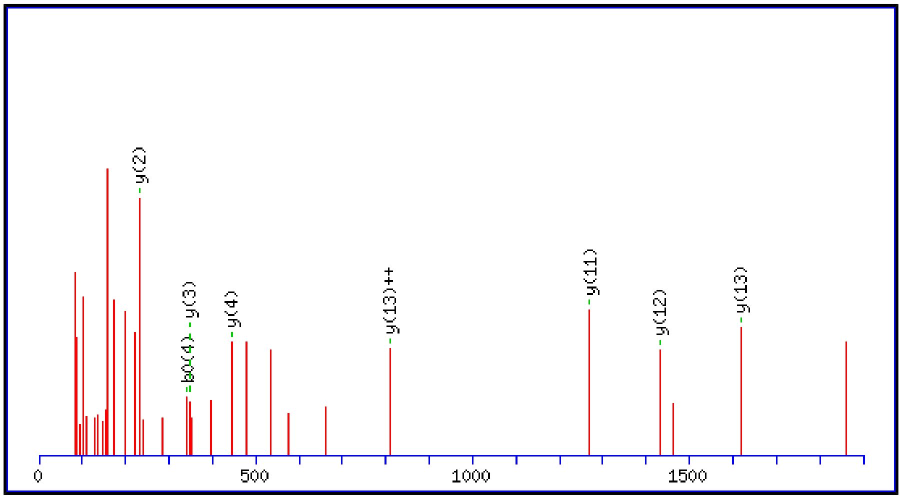

Monoisotopic mass of neutral peptide Mr(calc): 2217.9035
Fixed modifications: Carbamidomethyl (C) (apply to specified residues or termini only)
Variable modifications:
N15 : Deamidated (NQ)
N17 : Deamidated (NQ)
Ions Score: 24 **Expect:** 0.042
Matches : 8/166 fragment ions using 17 most intense peaks (help)

#	b	b^{++}	b*	b^{*++}	b^0	b^{0++}	Seq.	y	y^{++}	y*	y^{*++}	y^0	y^{0++}	#
1	130.0499	65.5286			112.0393	56.5233	E							19
2	245.0768	123.0420			227.0662	114.0368	D	2089.8682	1045.4377	2072.8416	1036.9245	2071.8576	1036.4324	18
3	302.0983	151.5528			284.0877	142.5475	G	1974.8412	987.9243	1957.8147	979.4110			17
4	359.1197	180.0635			341.1092	171.0582	G	1917.8198	959.4135	1900.7932	950.9003			16
5	416.1412	208.5742			398.1306	199.5690	G	1860.7983	930.9028	1843.7718	922.3895			15
6	602.2205	301.6139			584.2100	292.6086	W	1803.7768	902.3921	1786.7503	893.8788			14
7	788.2998	394.6536			770.2893	385.6483	W	***1617.6975***	809.3524	1600.6710	800.8391			13
8	951.3632	476.1852			933.3526	467.1799	Y	***1431.6182***	716.3127	1414.5917	707.7995			12
9	1065.4061	533.2067	1048.3795	524.6934	1047.3955	524.2014	N	***1268.5549***	634.7811	1251.5283	626.2678			11
10	1221.5072	611.2572	1204.4806	602.7440	1203.4966	602.2520	R	1154.5120	577.7596	1137.4854	569.2463			10
11	1381.5378	691.2726	1364.5113	682.7593	1363.5273	682.2673	C	998.4109	499.7091	981.3843	491.1958			9
12	1518.5968	759.8020	1501.5702	751.2887	1500.5862	750.7967	H	838.3802	419.6937	821.3537	411.1805			8
13	1589.6339	795.3206	1572.6073	786.8073	1571.6233	786.3153	A	701.3213	351.1643	684.2947	342.6510			7
14	1660.6710	830.8391	1643.6444	822.3259	1642.6604	821.8338	A	630.2842	315.6457	613.2576	307.1325			6
15	1775.6979	888.3526	1758.6714	879.8393	1757.6874	879.3473	N	559.2471	280.1272	542.2205	271.6139			5
16	1872.7507	936.8790	1855.7241	928.3657	1854.7401	927.8737	P	***444.2201***	222.6137	427.1936	214.1004			4
17	1987.7776	994.3925	1970.7511	985.8792	1969.7671	985.3872	N	***347.1674***	174.0873	330.1408	165.5740			3

**18**	2044.7991	1022.9032	2027.7726	1014.3899	2026.7885	1013.8979	**G**	*232.1404*	116.5738	215.1139	108.0606			**2**
**19**							**R**	175.1190	88.0631	158.0924	79.5498			**1**

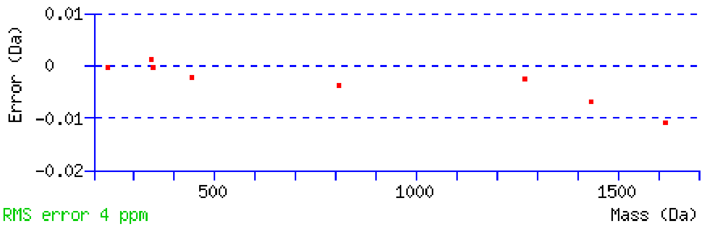

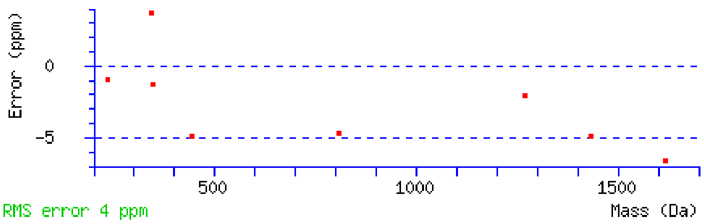

NCBI **BLAST** search of EDGGGWWYNRCHAANPNGR
(Parameters: blastp, nr protein database, expect=20000, no filter, PAM30)
Other BLAST web gateways

All matches to this query

Score	Mr(calc)	Delta	Sequence	Site Analysis
24.1	2217.9035	0.0032	EDGGGWWYNRCHAANPNGR	Deamidated N15, N17 47.63%
24.1	2217.9035	0.0032	EDGGGWWYNRCHAANPNGR	Deamidated N9, N17 47.63%
14.1	2217.9035	0.0032	EDGGGWWYNRCHAANPNGR	Deamidated N9, N15 4.73%

Mascot: http://www.matrixscience.com/

Mascot Search Results

Peptide View

MS/MS Fragmentation of **EDGGGWWYNRCHAANPNGR**
Found in **P02675** in **uni_human_nr**, FIBB_HUMAN Fibrinogen beta chain OS=Homo sapiens GN=FGB PE=1 SV=2

Match to Query 11110: 2215.937456 from(554.991640,4+) intensity(1238908.0000) rtinseconds(1404) scans(6941) index(4640)
Title: 130801_HPL_Human_Plaque_BR2_TR1_03_Spectrum027582_scans__6941_RTINSECONDS=1404
Data file E:\\RAW\\HumanPlaques\\mgf\\T\\1D\\T130801_HPL_Human_Plaque_BR2_TR1_03.mgf

Click mouse within plot area to zoom in by factor of two about that point
Or, [Plot from] [0] to [2400] Da [Full range]
Label all possible matches ○ Label matches used for scoring ⊙
Show Y-axis ☐

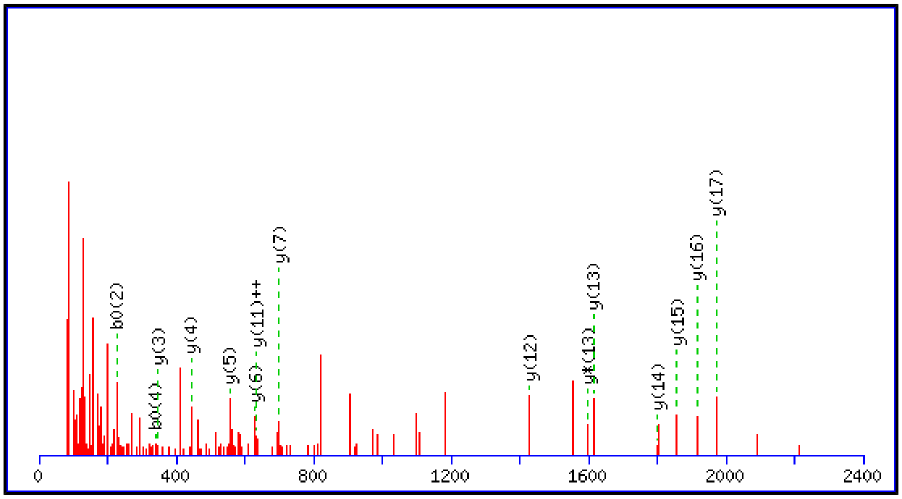

Monoisotopic mass of neutral peptide Mr(calc): 2215.9355
Fixed modifications: Carbamidomethyl (C) (apply to specified residues or termini only)
Ions Score: 29 **Expect:** 0.027
Matches : 15/166 fragment ions using 61 most intense peaks (help)

#	b	b^{++}	b*	b^{*++}	b^0	b^{0++}	Seq.	y	y^{++}	y*	y^{*++}	y^0	y^{0++}	#
1	130.0499	65.5286			112.0393	56.5233	E							19
2	245.0768	123.0420			227.0662	114.0368	D	2087.9002	1044.4537	2070.8736	1035.9404	2069.8896	1035.4484	18
3	302.0983	151.5528			284.0877	142.5475	G	***1972.8732***	986.9402	1955.8467	978.4270			17
4	359.1197	180.0635			341.1092	171.0582	G	***1915.8517***	958.4295	1898.8252	949.9162			16
5	416.1412	208.5742			398.1306	199.5690	G	***1858.8303***	929.9188	1841.8037	921.4055			15
6	602.2205	301.6139			584.2100	292.6086	W	***1801.8088***	901.4080	1784.7823	892.8948			14
7	788.2998	394.6536			770.2893	385.6483	W	***1615.7295***	808.3684	1598.7030	799.8551			13
8	951.3632	476.1852			933.3526	467.1799	Y	***1429.6502***	715.3287	1412.6236	706.8155			12
9	1065.4061	533.2067	1048.3795	524.6934	1047.3955	524.2014	N	1266.5869	633.7971	1249.5603	625.2838			11
10	1221.5072	611.2572	1204.4806	602.7440	1203.4966	602.2520	R	1152.5439	576.7756	1135.5174	568.2623			10
11	1381.5378	691.2726	1364.5113	682.7593	1363.5273	682.2673	C	996.4428	498.7250	979.4163	490.2118			9
12	1518.5968	759.8020	1501.5702	751.2887	1500.5862	750.7967	H	836.4122	418.7097	819.3856	410.1965			8
13	1589.6339	795.3206	1572.6073	786.8073	1571.6233	786.3153	A	***699.3533***	350.1803	682.3267	341.6670			7
14	1660.6710	830.8391	1643.6444	822.3259	1642.6604	821.8338	A	***628.3161***	314.6617	611.2896	306.1484			6
15	1774.7139	887.8606	1757.6874	879.3473	1756.7033	878.8553	N	***557.2790***	279.1432	540.2525	270.6299			5
16	1871.7667	936.3870	1854.7401	927.8737	1853.7561	927.3817	P	***443.2361***	222.1217	426.2096	213.6084			4
17	1985.8096	993.4084	1968.7831	984.8952	1967.7990	984.4032	N	***346.1833***	173.5953	329.1568	165.0820			3
18	2042.8311	1021.9192	2025.8045	1013.4059	2024.8205	1012.9139	G	232.1404	116.5738	215.1139	108.0606			2
19							R	175.1190	88.0631	158.0924	79.5498			1

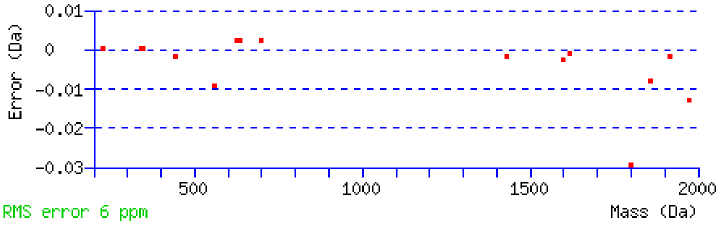

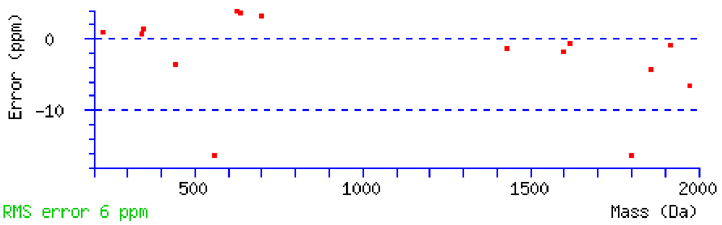

NCBI **BLAST** search of EDGGGWWYNRCHAANPNGR
(Parameters: blastp, nr protein database, expect=20000, no filter, PAM30)
Other BLAST web gateways

All matches to this query

Score	Mr(calc)	Delta	Sequence
29.1	2215.9355	0.0020	EDGGGWWYNRCHAANPNGR

**Mascot:** http://www.matrixscience.com/

Mascot Search Results

Peptide View

MS/MS Fragmentation of **EGFGHLSPTGTTEFWLGNEKIHLISTQSAIPYALRVELEDWNGR**
Found in **P02679** in **uni_human_nr**, FIBG_HUMAN Fibrinogen gamma chain OS=Homo sapiens GN=FGG PE=1 SV=3

Match to Query 23881: 4971.487370 from(995.304750,5+) intensity(5309084.0000) rtinseconds(3346) scans(23026) index(18926)
Title: 130806_HPL_Human_Plaque_BR1_TR2_01_Spectrum043298_scans__23026_RTINSECONDS=3346
Data file E:\\RAW\\HumanPlaques\\mgf\\T\\1D\\T130806_HPL_Human_Plaque_BR1_TR2_01.mgf

Click mouse within plot area to zoom in by factor of two about that point
Or, Plot from 0 to 5000 Da Full range
Label all possible matches ○ Label matches used for scoring ⊙
Show Y-axis ☐

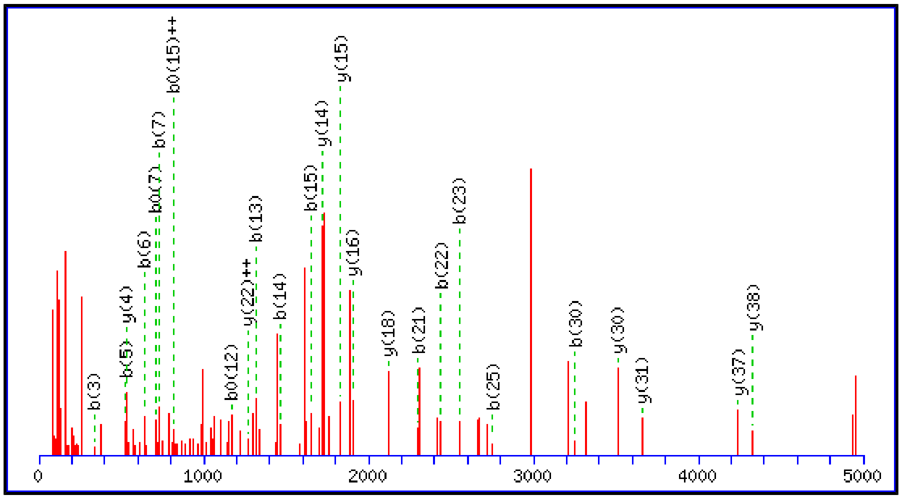


```
Monoisotopic mass of neutral peptide Mr(calc): 4969.4675
Fixed modifications: Carbamidomethyl (C) (apply to specified residues or termini only)
Variable modifications:
Q27     : Deamidated (NQ)
Ions Score: 50  Expect: 0.00042
Matches : 25/474 fragment ions using 53 most intense peaks   (help)
```

#	b	b++	b*	b*++	b0	b0++	Seq.	y	y++	y*	y*++	y0	y0++	#
1	130.0499	65.5286			112.0393	56.5233	E							44
2	187.0713	94.0393			169.0608	85.0340	G	4841.4322	2421.2197	4824.4056	2412.7065	4823.4216	2412.2145	43
3	***334.1397***	167.5735			316.1292	158.5682	F	4784.4107	2392.7090	4767.3842	2384.1957	4766.4002	2383.7037	42
4	391.1612	196.0842			373.1506	187.0790	G	4637.3423	2319.1748	4620.3158	2310.6615	4619.3318	2310.1695	41
5	***528.2201***	264.6137			510.2096	255.6084	H	4580.3209	2290.6641	4563.2943	2282.1508	4562.3103	2281.6588	40
6	***641.3042***	321.1557			623.2936	312.1504	L	4443.2619	2222.1346	4426.2354	2213.6213	4425.2514	2213.1293	39
7	***728.3362***	364.6717			710.3257	355.6665	S	***4330.1779***	2165.5926	4313.1513	2157.0793	4312.1673	2156.5873	38
8	825.3890	413.1981			807.3784	404.1928	P	***4243.1459***	2122.0766	4226.1193	2113.5633	4225.1353	2113.0713	37
9	926.4367	463.7220			908.4261	454.7167	T	4146.0931	2073.5502	4129.0665	2065.0369	4128.0825	2064.5449	36
10	983.4581	492.2327			965.4476	483.2274	G	4045.0454	2023.0263	4028.0189	2014.5131	4027.0348	2014.0211	35
11	1084.5058	542.7565			1066.4952	533.7513	T	3988.0239	1994.5156	3970.9974	1986.0023	3970.0134	1985.5103	34
12	1185.5535	593.2804			1167.5429	584.2751	T	3886.9763	1943.9918	3869.9497	1935.4785	3868.9657	1934.9865	33
13	***1314.5961***	657.8017			1296.5855	648.7964	E	3785.9286	1893.4679	3768.9020	1884.9547	3767.9180	1884.4626	32
14	***1461.6645***	731.3359			1443.6539	722.3306	F	***3656.8860***	1828.9466	3639.8594	1820.4334	3638.8754	1819.9414	31
15	***1647.7438***	824.3755			1629.7332	815.3703	W	***3509.8176***	1755.4124	3492.7910	1746.8992	3491.8070	1746.4071	30
16	1760.8279	880.9176			1742.8173	871.9123	L	3323.7383	1662.3728	3306.7117	1653.8595	3305.7277	1653.3675	29
17	1817.8493	909.4283			1799.8388	900.4230	G	3210.6542	1605.8307	3193.6277	1597.3175	3192.6436	1596.8255	28
18	1931.8923	966.4498	1914.8657	957.9365	1913.8817	957.4445	N	3153.6327	1577.3200	3136.6062	1568.8067	3135.6222	1568.3147	27

19	2060.9348	1030.9711	2043.9083	1022.4578	2042.9243	1021.9658	E	3039.5898	1520.2985	3022.5633	1511.7853	3021.5792	1511.2933	26
20	2189.0298	1095.0185	2172.0033	1086.5053	2171.0192	1086.0133	K	2910.5472	1455.7772	2893.5207	1447.2640	2892.5367	1446.7720	25
21	***2302.1139***	1151.5606	2285.0873	1143.0473	2284.1033	1142.5553	I	2782.4523	1391.7298	2765.4257	1383.2165	2764.4417	1382.7245	24
22	***2439.1728***	1220.0900	2422.1462	1211.5768	2421.1622	1211.0847	H	2669.3682	1335.1877	2652.3416	1326.6745	2651.3576	1326.1825	23
23	***2552.2569***	1276.6321	2535.2303	1268.1188	2534.2463	1267.6268	L	2532.3093	1266.6583	2515.2827	1258.1450	2514.2987	1257.6530	22
24	2665.3409	1333.1741	2648.3144	1324.6608	2647.3303	1324.1688	I	2419.2252	1210.1162	2402.1987	1201.6030	2401.2147	1201.1110	21
25	***2752.3729***	1376.6901	2735.3464	1368.1768	2734.3624	1367.6848	S	2306.1412	1153.5742	2289.1146	1145.0609	2288.1306	1144.5689	20
26	2853.4206	1427.2139	2836.3941	1418.7007	2835.4101	1418.2087	T	2219.1091	1110.0582	2202.0826	1101.5449	2201.0986	1101.0529	19
27	2982.4632	1491.7352	2965.4367	1483.2220	2964.4527	1482.7300	Q	***2118.0614***	1059.5344	2101.0349	1051.0211	2100.0509	1050.5291	18
28	3069.4952	1535.2513	3052.4687	1526.7380	3051.4847	1526.2460	S	1989.0189	995.0131	1971.9923	986.4998	1971.0083	986.0078	17
29	3140.5324	1570.7698	3123.5058	1562.2565	3122.5218	1561.7645	A	***1901.9868***	951.4970	1884.9603	942.9838	1883.9763	942.4918	16
30	***3253.6164***	1627.3118	3236.5899	1618.7986	3235.6059	1618.3066	I	***1830.9497***	915.9785	1813.9232	907.4652	1812.9391	906.9732	15
31	3350.6692	1675.8382	3333.6426	1667.3250	3332.6586	1666.8329	P	***1717.8656***	859.4365	1700.8391	850.9232	1699.8551	850.4312	14
32	3513.7325	1757.3699	3496.7060	1748.8566	3495.7220	1748.3646	Y	1620.8129	810.9101	1603.7863	802.3968	1602.8023	801.9048	13
33	3584.7696	1792.8885	3567.7431	1784.3752	3566.7591	1783.8832	A	1457.7496	729.3784	1440.7230	720.8651	1439.7390	720.3731	12
34	3697.8537	1849.4305	3680.8271	1840.9172	3679.8431	1840.4252	L	1386.7124	693.8599	1369.6859	685.3466	1368.7019	684.8546	11
35	3853.9548	1927.4810	3836.9283	1918.9678	3835.9442	1918.4758	R	1273.6284	637.3178	1256.6018	628.8046	1255.6178	628.3125	10
36	3953.0232	1977.0152	3935.9967	1968.5020	3935.0127	1968.0100	V	1117.5273	559.2673	1100.5007	550.7540	1099.5167	550.2620	9
37	4082.0658	2041.5365	4065.0393	2033.0233	4064.0552	2032.5313	E	1018.4588	509.7331	1001.4323	501.2198	1000.4483	500.7278	8
38	4195.1499	2098.0786	4178.1233	2089.5653	4177.1393	2089.0733	L	889.4163	445.2118	872.3897	436.6985	871.4057	436.2065	7
39	4324.1925	2162.5999	4307.1659	2154.0866	4306.1819	2153.5946	E	776.3322	388.6697	759.3056	380.1565	758.3216	379.6645	6
40	4439.2194	2220.1133	4422.1929	2211.6001	4421.2088	2211.1081	D	647.2896	324.1484	630.2631	315.6352	629.2790	315.1432	5
41	4625.2987	2313.1530	4608.2722	2304.6397	4607.2882	2304.1477	W	***532.2627***	266.6350	515.2361	258.1217			4
42	4739.3417	2370.1745	4722.3151	2361.6612	4721.3311	2361.1692	N	346.1833	173.5953	329.1568	165.0820			3
43	4796.3631	2398.6852	4779.3366	2390.1719	4778.3526	2389.6799	G	232.1404	116.5738	215.1139	108.0606			2
44							R	175.1190	88.0631	158.0924	79.5498			1

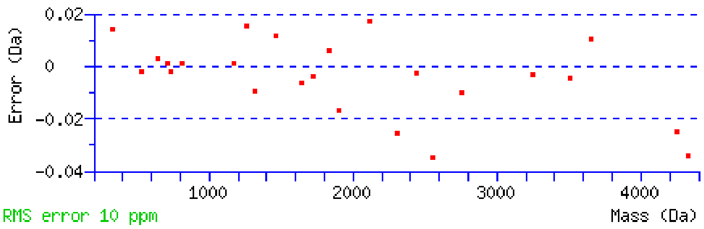

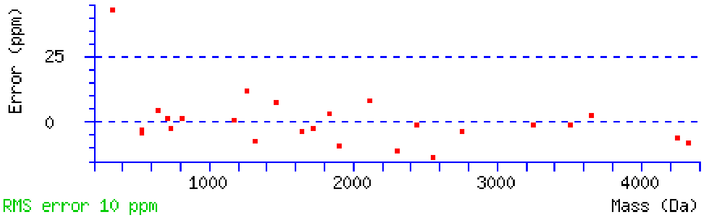

NCBI **BLAST** search of EGFGHLSPTGTTEFWLGNEKIHLISTQSAIPYALRVELEDWNGR
(Parameters: blastp, nr protein database, expect=20000, no filter, PAM30)
Other BLAST web gateways

All matches to this query

Score	Mr(calc)	Delta	Sequence	Site Analysis
50.3	4969.4675	2.0199	EGFGHLSPTGTTEFWLGNEKIHLISTQSAIPYALRVELEDWNGR	Deamidated Q27 95.64%
36.0	4969.4675	2.0199	EGFGHLSPTGTTEFWLGNEKIHLISTQSAIPYALRVELEDWNGR	Deamidated N18 3.58%
29.4	4969.4675	2.0199	EGFGHLSPTGTTEFWLGNEKIHLISTQSAIPYALRVELEDWNGR	Deamidated N42 0.78%

Mascot: http://www.matrixscience.com/

Mascot Search Results

Peptide View

MS/MS Fragmentation of **EGFGHLSPTGTTEFWLGNEKIHLISTQSAIPYALRVELEDWNGR**
Found in **P02679** in **uni_human_nr**, FIBG_HUMAN Fibrinogen gamma chain OS=Homo sapiens GN=FGG PE=1 SV=3

Match to Query 23083: 4971.484764 from(829.588070,6+) intensity(14187161.0000) rtinseconds(3338) scans(22434) index(18122)
Title: 130801_HPL_Human_Plaque_BR1_TR1_01_Spectrum041770_scans__22434_RTINSECONDS=3338
Data file E:\\RAW\\HumanPlaques\\mgf\\T\\1D\\T130801_HPL_Human_Plaque_BR1_TR1_01.mgf

Click mouse within plot area to zoom in by factor of two about that point
Or, Plot from 0 to 3400 Da Full range
Label all possible matches ○ Label matches used for scoring ⊙
Show Y-axis ☐

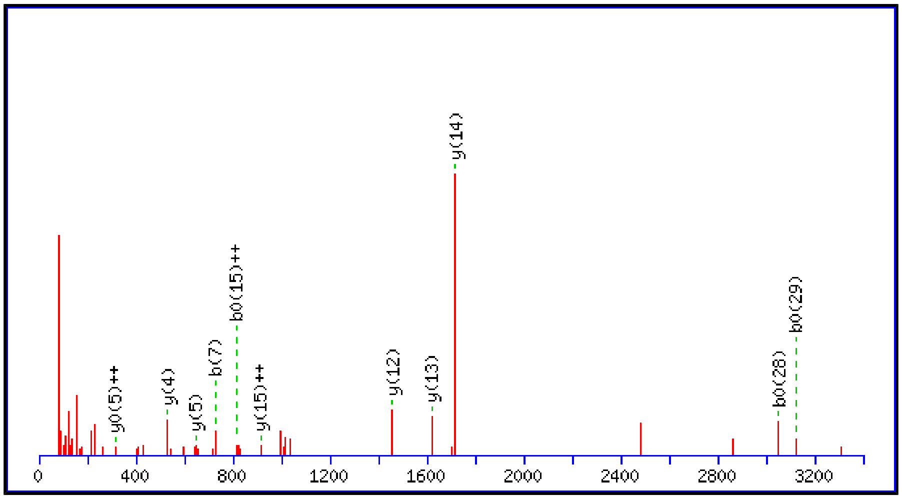

Monoisotopic mass of neutral peptide Mr(calc): 4969.4675
Fixed modifications: Carbamidomethyl (C) (apply to specified residues or termini only)
Variable modifications:
Q27 : Deamidated (NQ)
Ions Score: 19 **Expect:** 0.6
Matches : 11/474 fragment ions using 18 most intense peaks (help)

#	b	b^{++}	b*	b^{*++}	b^0	b^{0++}	Seq.	y	y^{++}	y*	y^{*++}	y^0	y^{0++}	#
1	130.0499	65.5286			112.0393	56.5233	E							44
2	187.0713	94.0393			169.0608	85.0340	G	4841.4322	2421.2197	4824.4056	2412.7065	4823.4216	2412.2145	43
3	334.1397	167.5735			316.1292	158.5682	F	4784.4107	2392.7090	4767.3842	2384.1957	4766.4002	2383.7037	42
4	391.1612	196.0842			373.1506	187.0790	G	4637.3423	2319.1748	4620.3158	2310.6615	4619.3318	2310.1695	41
5	528.2201	264.6137			510.2096	255.6084	H	4580.3209	2290.6641	4563.2943	2282.1508	4562.3103	2281.6588	40
6	641.3042	321.1557			623.2936	312.1504	L	4443.2619	2222.1346	4426.2354	2213.6213	4425.2514	2213.1293	39
7	728.3362	364.6717			710.3257	355.6665	S	4330.1779	2165.5926	4313.1513	2157.0793	4312.1673	2156.5873	38
8	825.3890	413.1981			807.3784	404.1928	P	4243.1459	2122.0766	4226.1193	2113.5633	4225.1353	2113.0713	37
9	926.4367	463.7220			908.4261	454.7167	T	4146.0931	2073.5502	4129.0665	2065.0369	4128.0825	2064.5449	36
10	983.4581	492.2327			965.4476	483.2274	G	4045.0454	2023.0263	4028.0189	2014.5131	4027.0348	2014.0211	35
11	1084.5058	542.7565			1066.4952	533.7513	T	3988.0239	1994.5156	3970.9974	1986.0023	3970.0134	1985.5103	34
12	1185.5535	593.2804			1167.5429	584.2751	T	3886.9763	1943.9918	3869.9497	1935.4785	3868.9657	1934.9865	33
13	1314.5961	657.8017			1296.5855	648.7964	E	3785.9286	1893.4679	3768.9020	1884.9547	3767.9180	1884.4626	32
14	1461.6645	731.3359			1443.6539	722.3306	F	3656.8860	1828.9466	3639.8594	1820.4334	3638.8754	1819.9414	31
15	1647.7438	824.3755			1629.7332	815.3703	W	3509.8176	1755.4124	3492.7910	1746.8992	3491.8070	1746.4071	30
16	1760.8279	880.9176			1742.8173	871.9123	L	3323.7383	1662.3728	3306.7117	1653.8595	3305.7277	1653.3675	29
17	1817.8493	909.4283			1799.8388	900.4230	G	3210.6542	1605.8307	3193.6277	1597.3175	3192.6436	1596.8255	28
18	1931.8923	966.4498	1914.8657	957.9365	1913.8817	957.4445	N	3153.6327	1577.3200	3136.6062	1568.8067	3135.6222	1568.3147	27

**19**	2060.9348	1030.9711	2043.9083	1022.4578	2042.9243	1021.9658	**E**	3039.5898	1520.2985	3022.5633	1511.7853	3021.5792	1511.2933	**26**
**20**	2189.0298	1095.0185	2172.0033	1086.5053	2171.0192	1086.0133	**K**	2910.5472	1455.7772	2893.5207	1447.2640	2892.5367	1446.7720	**25**
**21**	2302.1139	1151.5606	2285.0873	1143.0473	2284.1033	1142.5553	**I**	2782.4523	1391.7298	2765.4257	1383.2165	2764.4417	1382.7245	**24**
**22**	2439.1728	1220.0900	2422.1462	1211.5768	2421.1622	1211.0847	**H**	2669.3682	1335.1877	2652.3416	1326.6745	2651.3576	1326.1825	**23**
**23**	2552.2569	1276.6321	2535.2303	1268.1188	2534.2463	1267.6268	**L**	2532.3093	1266.6583	2515.2827	1258.1450	2514.2987	1257.6530	**22**
**24**	2665.3409	1333.1741	2648.3144	1324.6608	2647.3303	1324.1688	**I**	2419.2252	1210.1162	2402.1987	1201.6030	2401.2147	1201.1110	**21**
**25**	2752.3729	1376.6901	2735.3464	1368.1768	2734.3624	1367.6848	**S**	2306.1412	1153.5742	2289.1146	1145.0609	2288.1306	1144.5689	**20**
**26**	2853.4206	1427.2139	2836.3941	1418.7007	2835.4101	1418.2087	**T**	2219.1091	1110.0582	2202.0826	1101.5449	2201.0986	1101.0529	**19**
**27**	2982.4632	1491.7352	2965.4367	1483.2220	2964.4527	1482.7300	**Q**	2118.0614	1059.5344	2101.0349	1051.0211	2100.0509	1050.5291	**18**
**28**	3069.4952	1535.2513	3052.4687	1526.7380	3051.4847	1526.2460	**S**	1989.0189	995.0131	1971.9923	986.4998	1971.0083	986.0078	**17**
**29**	3140.5324	1570.7698	3123.5058	1562.2565	3122.5218	1561.7645	**A**	1901.9868	951.4970	1884.9603	942.9838	1883.9763	942.4918	**16**
**30**	3253.6164	1627.3118	3236.5899	1618.7986	3235.6059	1618.3066	**I**	1830.9497	915.9785	1813.9232	907.4652	1812.9391	906.9732	**15**
**31**	3350.6692	1675.8382	3333.6426	1667.3250	3332.6586	1666.8329	**P**	***1717.8656***	859.4365	1700.8391	850.9232	1699.8551	850.4312	**14**
**32**	3513.7325	1757.3699	3496.7060	1748.8566	3495.7220	1748.3646	**Y**	***1620.8129***	810.9101	1603.7863	802.3968	1602.8023	801.9048	**13**
**33**	3584.7696	1792.8885	3567.7431	1784.3752	3566.7591	1783.8832	**A**	***1457.7496***	729.3784	1440.7230	720.8651	1439.7390	720.3731	**12**
**34**	3697.8537	1849.4305	3680.8271	1840.9172	3679.8431	1840.4252	**L**	1386.7124	693.8599	1369.6859	685.3466	1368.7019	684.8546	**11**
**35**	3853.9548	1927.4810	3836.9283	1918.9678	3835.9442	1918.4758	**R**	1273.6284	637.3178	1256.6018	628.8046	1255.6178	628.3125	**10**
**36**	3953.0232	1977.0152	3935.9967	1968.5020	3935.0127	1968.0100	**V**	1117.5273	559.2673	1100.5007	550.7540	1099.5167	550.2620	**9**
**37**	4082.0658	2041.5365	4065.0393	2033.0233	4064.0552	2032.5313	**E**	1018.4588	509.7331	1001.4323	501.2198	1000.4483	500.7278	**8**
**38**	4195.1499	2098.0786	4178.1233	2089.5653	4177.1393	2089.0733	**L**	889.4163	445.2118	872.3897	436.6985	871.4057	436.2065	**7**
**39**	4324.1925	2162.5999	4307.1659	2154.0866	4306.1819	2153.5946	**E**	776.3322	388.6697	759.3056	380.1565	758.3216	379.6645	**6**
**40**	4439.2194	2220.1133	4422.1929	2211.6001	4421.2088	2211.1081	**D**	***647.2896***	324.1484	630.2631	315.6352	629.2790	315.1432	**5**
**41**	4625.2987	2313.1530	4608.2722	2304.6397	4607.2882	2304.1477	**W**	***532.2627***	266.6350	515.2361	258.1217			**4**
**42**	4739.3417	2370.1745	4722.3151	2361.6612	4721.3311	2361.1692	**N**	346.1833	173.5953	329.1568	165.0820			**3**
**43**	4796.3631	2398.6852	4779.3366	2390.1719	4778.3526	2389.6799	**G**	232.1404	116.5738	215.1139	108.0606			**2**
**44**							**R**	175.1190	88.0631	158.0924	79.5498			**1**

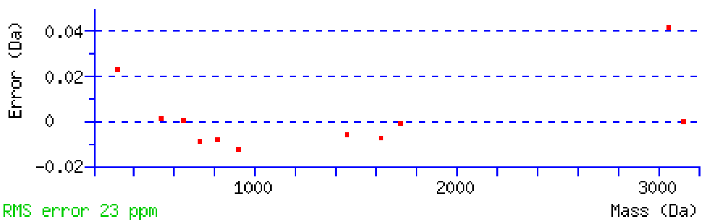

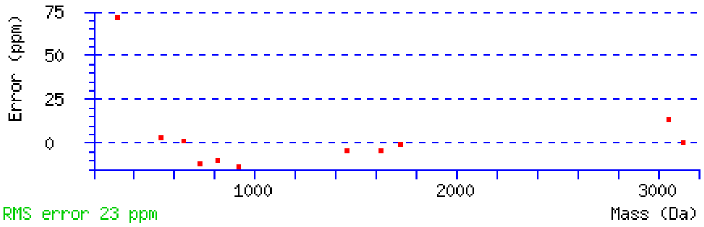

NCBI **BLAST** search of EGFGHLSPTGTTEFWLGNEKIHLISTQSAIPYALRVELEDWNGR
(Parameters: blastp, nr protein database, expect=20000, no filter, PAM30)
Other BLAST web gateways

All matches to this query

Score	Mr(calc)	Delta	Sequence
18.9	4969.4675	2.0173	EGFGHLSPTGTTEFWLGNEKIHLISTQSAIPYALRVELEDWNGR
18.9	4969.4675	2.0173	EGFGHLSPTGTTEFWLGNEKIHLISTQSAIPYALRVELEDWNGR
0.2	4971.4977	-0.0130	IYLESEHGSPLTPRVGIPSGLGAECPSQPPLHGIHIADNNPFNLLR
0.2	4971.4977	-0.0130	IYLESEHGSPLTPRVGIPSGLGAECPSQPPLHGIHIADNNPFNLLR
0.2	4971.4977	-0.0130	IYLESEHGSPLTPRVGIPSGLGAECPSQPPLHGIHIADNNPFNLLR

Mascot: http://www.matrixscience.com/

Mascot Search Results

Peptide View

MS/MS Fragmentation of **EGFGHLSPTGTTEFWLGNEKIHLISTQSAIPYALRVELEDWNGR**
Found in **P02679** in **uni_human_nr**, FIBG_HUMAN Fibrinogen gamma chain OS=Homo sapiens GN=FGG PE=1 SV=3

Match to Query 23517: 4968.483684 from(829.087890,6+) intensity(2524746.7500) rtinseconds(3375) scans(23536) index(19496)
Title: 130806_HPL_Human_Plaque_BR2_TR2_02_Spectrum044329_scans__23536_RTINSECONDS=3375
Data file E:\\RAW\\HumanPlaques\\mgf\\T\\1D\\T130806_HPL_Human_Plaque_BR2_TR2_02.mgf

Click mouse within plot area to zoom in by factor of two about that point
Or, [Plot from] 0 to 3200 Da [Full range]
Label all possible matches ○ Label matches used for scoring ⊙
Show Y-axis ☐

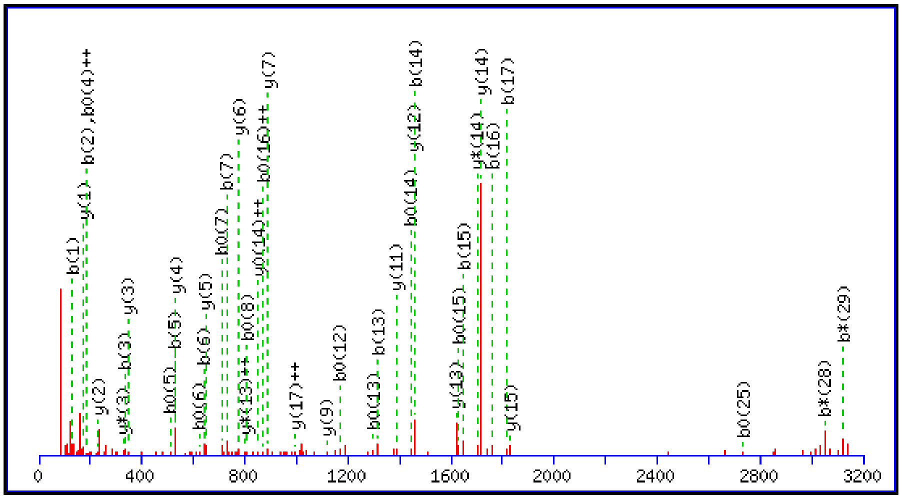

Monoisotopic mass of neutral peptide Mr(calc): 4968.4835
Fixed modifications: Carbamidomethyl (C) (apply to specified residues or termini only)
Ions Score: 53 **Expect:** 0.00026
Matches : 42/474 fragment ions using 110 most intense peaks (help)

#	b	b^{++}	b*	b^{*++}	b^0	b^{0++}	Seq.	y	y^{++}	y*	y^{*++}	y^0	y^{0++}	#
1	130.0499	65.5286			112.0393	56.5233	E							44
2	187.0713	94.0393			169.0608	85.0340	G	4840.4482	2420.7277	4823.4216	2412.2145	4822.4376	2411.7224	43
3	334.1397	167.5735			316.1292	158.5682	F	4783.4267	2392.2170	4766.4002	2383.7037	4765.4162	2383.2117	42
4	391.1612	196.0842			373.1506	187.0790	G	4636.3583	2318.6828	4619.3318	2310.1695	4618.3477	2309.6775	41
5	528.2201	264.6137			510.2096	255.6084	H	4579.3368	2290.1721	4562.3103	2281.6588	4561.3263	2281.1668	40
6	641.3042	321.1557			623.2936	312.1504	L	4442.2779	2221.6426	4425.2514	2213.1293	4424.2674	2212.6373	39
7	728.3362	364.6717			710.3257	355.6665	S	4329.1939	2165.1006	4312.1673	2156.5873	4311.1833	2156.0953	38
8	825.3890	413.1981			807.3784	404.1928	P	4242.1618	2121.5846	4225.1353	2113.0713	4224.1513	2112.5793	37
9	926.4367	463.7220			908.4261	454.7167	T	4145.1091	2073.0582	4128.0825	2064.5449	4127.0985	2064.0529	36
10	983.4581	492.2327			965.4476	483.2274	G	4044.0614	2022.5343	4027.0348	2014.0211	4026.0508	2013.5291	35
11	1084.5058	542.7565			1066.4952	533.7513	T	3987.0399	1994.0236	3970.0134	1985.5103	3969.0294	1985.0183	34
12	1185.5535	593.2804			1167.5429	584.2751	T	3885.9923	1943.4998	3868.9657	1934.9865	3867.9817	1934.4945	33
13	1314.5961	657.8017			1296.5855	648.7964	E	3784.9446	1892.9759	3767.9180	1884.4626	3766.9340	1883.9706	32
14	1461.6645	731.3359			1443.6539	722.3306	F	3655.9020	1828.4546	3638.8754	1819.9414	3637.8914	1819.4493	31
15	1647.7438	824.3755			1629.7332	815.3703	W	3508.8336	1754.9204	3491.8070	1746.4071	3490.8230	1745.9151	30
16	1760.8279	880.9176			1742.8173	871.9123	L	3322.7543	1661.8808	3305.7277	1653.3675	3304.7437	1652.8755	29
17	1817.8493	909.4283			1799.8388	900.4230	G	3209.6702	1605.3387	3192.6436	1596.8255	3191.6596	1596.3334	28
18	1931.8923	966.4498	1914.8657	957.9365	1913.8817	957.4445	N	3152.6487	1576.8280	3135.6222	1568.3147	3134.6382	1567.8227	27
19	2060.9348	1030.9711	2043.9083	1022.4578	2042.9243	1021.9658	E	3038.6058	1519.8065	3021.5792	1511.2933	3020.5952	1510.8013	26

20	2189.0298	1095.0185	2172.0033	1086.5053	2171.0192	1086.0133	K	2909.5632	1455.2852	2892.5367	1446.7720	2891.5526	1446.2800	25
21	2302.1139	1151.5606	2285.0873	1143.0473	2284.1033	1142.5553	I	2781.4682	1391.2378	2764.4417	1382.7245	2763.4577	1382.2325	24
22	2439.1728	1220.0900	2422.1462	1211.5768	2421.1622	1211.0847	H	2668.3842	1334.6957	2651.3576	1326.1825	2650.3736	1325.6904	23
23	2552.2569	1276.6321	2535.2303	1268.1188	2534.2463	1267.6268	L	2531.3253	1266.1663	2514.2987	1257.6530	2513.3147	1257.1610	22
24	2665.3409	1333.1741	2648.3144	1324.6608	2647.3303	1324.1688	I	2418.2412	1209.6242	2401.2147	1201.1110	2400.2306	1200.6190	21
25	2752.3729	1376.6901	2735.3464	1368.1768	2734.3624	1367.6848	S	2305.1571	1153.0822	2288.1306	1144.5689	2287.1466	1144.0769	20
26	2853.4206	1427.2139	2836.3941	1418.7007	2835.4101	1418.2087	T	2218.1251	1109.5662	2201.0986	1101.0529	2200.1145	1100.5609	19
27	2981.4792	1491.2432	2964.4527	1482.7300	2963.4686	1482.2380	Q	2117.0774	1059.0424	2100.0509	1050.5291	2099.0669	1050.0371	18
28	3068.5112	1534.7593	3051.4847	1526.2460	3050.5007	1525.7540	S	1989.0189	995.0131	1971.9923	986.4998	1971.0083	986.0078	17
29	3139.5483	1570.2778	3122.5218	1561.7645	3121.5378	1561.2725	A	1901.9868	951.4970	1884.9603	942.9838	1883.9763	942.4918	16
30	3252.6324	1626.8198	3235.6059	1618.3066	3234.6218	1617.8146	I	***1830.9497***	915.9785	1813.9232	907.4652	1812.9391	906.9732	15
31	3349.6852	1675.3462	3332.6586	1666.8329	3331.6746	1666.3409	P	***1717.8656***	859.4365	1700.8391	850.9232	1699.8551	850.4312	14
32	3512.7485	1756.8779	3495.7220	1748.3646	3494.7379	1747.8726	Y	***1620.8129***	810.9101	1603.7863	802.3968	1602.8023	801.9048	13
33	3583.7856	1792.3964	3566.7591	1783.8832	3565.7750	1783.3912	A	***1457.7496***	729.3784	1440.7230	720.8651	1439.7390	720.3731	12
34	3696.8697	1848.9385	3679.8431	1840.4252	3678.8591	1839.9332	L	***1386.7124***	693.8599	1369.6859	685.3466	1368.7019	684.8546	11
35	3852.9708	1926.9890	3835.9442	1918.4758	3834.9602	1917.9837	R	1273.6284	637.3178	1256.6018	628.8046	1255.6178	628.3125	10
36	3952.0392	1976.5232	3935.0127	1968.0100	3934.0286	1967.5180	V	***1117.5273***	559.2673	1100.5007	550.7540	1099.5167	550.2620	9
37	4081.0818	2041.0445	4064.0552	2032.5313	4063.0712	2032.0393	E	1018.4588	509.7331	1001.4323	501.2198	1000.4483	500.7278	8
38	4194.1659	2097.5866	4177.1393	2089.0733	4176.1553	2088.5813	L	***889.4163***	445.2118	872.3897	436.6985	871.4057	436.2065	7
39	4323.2085	2162.1079	4306.1819	2153.5946	4305.1979	2153.1026	E	***776.3322***	388.6697	759.3056	380.1565	758.3216	379.6645	6
40	4438.2354	2219.6213	4421.2088	2211.1081	4420.2248	2210.6161	D	***647.2896***	324.1484	630.2631	315.6352	629.2790	315.1432	5
41	4624.3147	2312.6610	4607.2882	2304.1477	4606.3041	2303.6557	W	***532.2627***	266.6350	515.2361	258.1217			4
42	4738.3576	2369.6825	4721.3311	2361.1692	4720.3471	2360.6772	N	***346.1833***	173.5953	329.1568	165.0820			3
43	4795.3791	2398.1932	4778.3526	2389.6799	4777.3685	2389.1879	G	***232.1404***	116.5738	215.1139	108.0606			2
44							R	***175.1190***	88.0631	158.0924	79.5498			1

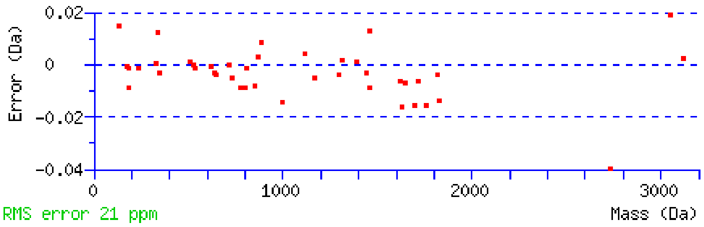

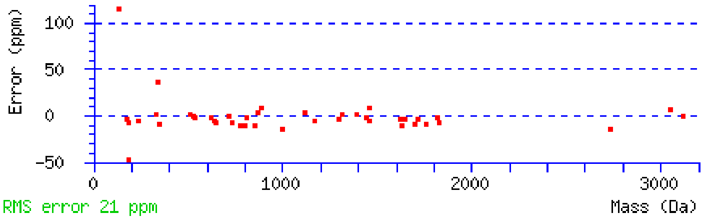

NCBI **BLAST** search of EGFGHLSPTGTTEFWLGNEKIHLISTQSAIPYALRVELEDWNGR
(Parameters: blastp, nr protein database, expect=20000, no filter, PAM30)
Other BLAST web gateways

All matches to this query

Score	Mr(calc)	Delta	Sequence
52.9	4968.4835	0.0002	EGFGHLSPTGTTEFWLGNEKIHLISTQSAIPYALRVELEDWNGR

Mascot: http://www.matrixscience.com/

Mascot Search Results

Peptide View

MS/MS Fragmentation of **EGFGHLSPTGTTEFWLGNEKIHLISTQSAIPYALRVELEDWNGR**
Found in **P02679** in **uni_human_nr**, FIBG_HUMAN Fibrinogen gamma chain OS=Homo sapiens GN=FGG PE=1 SV=3

Match to Query 23518: 4968.488120 from(994.704900,5+) intensity(1797980.0000) rtinseconds(3373) scans(23522) index(19484)
Title: 130806_HPL_Human_Plaque_BR2_TR2_02_Spectrum044317_scans__23522_RTINSECONDS=3373
Data file E:\\RAW\\HumanPlaques\\mgf\\T\\1D\\T130806_HPL_Human_Plaque_BR2_TR2_02.mgf

Click mouse within plot area to zoom in by factor of two about that point
Or, Plot from 0 to 5500 Da Full range
Label all possible matches ○ Label matches used for scoring ⊙
Show Y-axis ☐

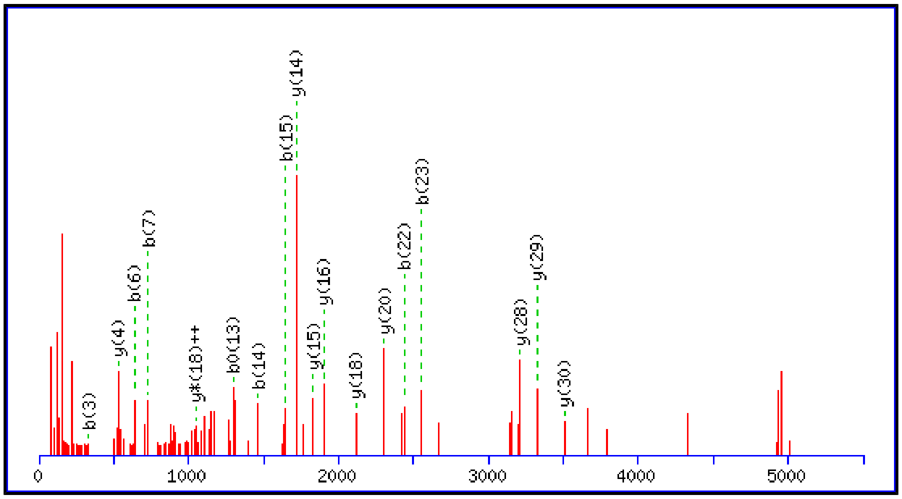

Monoisotopic mass of neutral peptide Mr(calc): 4968.4835
Fixed modifications: Carbamidomethyl (C) (apply to specified residues or termini only)
Ions Score: 52 **Expect:** 0.00031
Matches : 18/474 fragment ions using 30 most intense peaks (help)

#	b	b^{++}	b^{*}	b^{*++}	b^{0}	b^{0++}	Seq.	y	y^{++}	y^{*}	y^{*++}	y^{0}	y^{0++}	#
1	130.0499	65.5286			112.0393	56.5233	E							44
2	187.0713	94.0393			169.0608	85.0340	G	4840.4482	2420.7277	4823.4216	2412.2145	4822.4376	2411.7224	43
3	***334.1397***	167.5735			316.1292	158.5682	F	4783.4267	2392.2170	4766.4002	2383.7037	4765.4162	2383.2117	42
4	391.1612	196.0842			373.1506	187.0790	G	4636.3583	2318.6828	4619.3318	2310.1695	4618.3477	2309.6775	41
5	528.2201	264.6137			510.2096	255.6084	H	4579.3368	2290.1721	4562.3103	2281.6588	4561.3263	2281.1668	40
6	***641.3042***	321.1557			623.2936	312.1504	L	4442.2779	2221.6426	4425.2514	2213.1293	4424.2674	2212.6373	39
7	***728.3362***	364.6717			710.3257	355.6665	S	4329.1939	2165.1006	4312.1673	2156.5873	4311.1833	2156.0953	38
8	825.3890	413.1981			807.3784	404.1928	P	4242.1618	2121.5846	4225.1353	2113.0713	4224.1513	2112.5793	37
9	926.4367	463.7220			908.4261	454.7167	T	4145.1091	2073.0582	4128.0825	2064.5449	4127.0985	2064.0529	36
10	983.4581	492.2327			965.4476	483.2274	G	4044.0614	2022.5343	4027.0348	2014.0211	4026.0508	2013.5291	35
11	1084.5058	542.7565			1066.4952	533.7513	T	3987.0399	1994.0236	3970.0134	1985.5103	3969.0294	1985.0183	34
12	1185.5535	593.2804			1167.5429	584.2751	T	3885.9923	1943.4998	3868.9657	1934.9865	3867.9817	1934.4945	33
13	1314.5961	657.8017			1296.5855	648.7964	E	3784.9446	1892.9759	3767.9180	1884.4626	3766.9340	1883.9706	32
14	***1461.6645***	731.3359			1443.6539	722.3306	F	3655.9020	1828.4546	3638.8754	1819.9414	3637.8914	1819.4493	31
15	***1647.7438***	824.3755			1629.7332	815.3703	W	***3508.8336***	1754.9204	3491.8070	1746.4071	3490.8230	1745.9151	30
16	1760.8279	880.9176			1742.8173	871.9123	L	***3322.7543***	1661.8808	3305.7277	1653.3675	3304.7437	1652.8755	29
17	1817.8493	909.4283			1799.8388	900.4230	G	***3209.6702***	1605.3387	3192.6436	1596.8255	3191.6596	1596.3334	28
18	1931.8923	966.4498	1914.8657	957.9365	1913.8817	957.4445	N	3152.6487	1576.8280	3135.6222	1568.3147	3134.6382	1567.8227	27
19	2060.9348	1030.9711	2043.9083	1022.4578	2042.9243	1021.9658	E	3038.6058	1519.8065	3021.5792	1511.2933	3020.5952	1510.8013	26

**20**	2189.0298	1095.0185	2172.0033	1086.5053	2171.0192	1086.0133	**K**	2909.5632	1455.2852	2892.5367	1446.7720	2891.5526	1446.2800	**25**
**21**	2302.1139	1151.5606	2285.0873	1143.0473	2284.1033	1142.5553	**I**	2781.4682	1391.2378	2764.4417	1382.7245	2763.4577	1382.2325	**24**
**22**	***2439.1728***	1220.0900	2422.1462	1211.5768	2421.1622	1211.0847	**H**	2668.3842	1334.6957	2651.3576	1326.1825	2650.3736	1325.6904	**23**
**23**	***2552.2569***	1276.6321	2535.2303	1268.1188	2534.2463	1267.6268	**L**	2531.3253	1266.1663	2514.2987	1257.6530	2513.3147	1257.1610	**22**
**24**	2665.3409	1333.1741	2648.3144	1324.6608	2647.3303	1324.1688	**I**	2418.2412	1209.6242	2401.2147	1201.1110	2400.2306	1200.6190	**21**
**25**	2752.3729	1376.6901	2735.3464	1368.1768	2734.3624	1367.6848	**S**	***2305.1571***	1153.0822	2288.1306	1144.5689	2287.1466	1144.0769	**20**
**26**	2853.4206	1427.2139	2836.3941	1418.7007	2835.4101	1418.2087	**T**	2218.1251	1109.5662	2201.0986	1101.0529	2200.1145	1100.5609	**19**
**27**	2981.4792	1491.2432	2964.4527	1482.7300	2963.4686	1482.2380	**Q**	***2117.0774***	1059.0424	2100.0509	1050.5291	2099.0669	1050.0371	**18**
**28**	3068.5112	1534.7593	3051.4847	1526.2460	3050.5007	1525.7540	**S**	1989.0189	995.0131	1971.9923	986.4998	1971.0083	986.0078	**17**
**29**	3139.5483	1570.2778	3122.5218	1561.7645	3121.5378	1561.2725	**A**	***1901.9868***	951.4970	1884.9603	942.9838	1883.9763	942.4918	**16**
**30**	3252.6324	1626.8198	3235.6059	1618.3066	3234.6218	1617.8146	**I**	***1830.9497***	915.9785	1813.9232	907.4652	1812.9391	906.9732	**15**
**31**	3349.6852	1675.3462	3332.6586	1666.8329	3331.6746	1666.3409	**P**	***1717.8656***	859.4365	1700.8391	850.9232	1699.8551	850.4312	**14**
**32**	3512.7485	1756.8779	3495.7220	1748.3646	3494.7379	1747.8726	**Y**	1620.8129	810.9101	1603.7863	802.3968	1602.8023	801.9048	**13**
**33**	3583.7856	1792.3964	3566.7591	1783.8832	3565.7750	1783.3912	**A**	1457.7496	729.3784	1440.7230	720.8651	1439.7390	720.3731	**12**
**34**	3696.8697	1848.9385	3679.8431	1840.4252	3678.8591	1839.9332	**L**	1386.7124	693.8599	1369.6859	685.3466	1368.7019	684.8546	**11**
**35**	3852.9708	1926.9890	3835.9442	1918.4758	3834.9602	1917.9837	**R**	1273.6284	637.3178	1256.6018	628.8046	1255.6178	628.3125	**10**
**36**	3952.0392	1976.5232	3935.0127	1968.0100	3934.0286	1967.5180	**V**	1117.5273	559.2673	1100.5007	550.7540	1099.5167	550.2620	**9**
**37**	4081.0818	2041.0445	4064.0552	2032.5313	4063.0712	2032.0393	**E**	1018.4588	509.7331	1001.4323	501.2198	1000.4483	500.7278	**8**
**38**	4194.1659	2097.5866	4177.1393	2089.0733	4176.1553	2088.5813	**L**	889.4163	445.2118	872.3897	436.6985	871.4057	436.2065	**7**
**39**	4323.2085	2162.1079	4306.1819	2153.5946	4305.1979	2153.1026	**E**	776.3322	388.6697	759.3056	380.1565	758.3216	379.6645	**6**
**40**	4438.2354	2219.6213	4421.2088	2211.1081	4420.2248	2210.6161	**D**	647.2896	324.1484	630.2631	315.6352	629.2790	315.1432	**5**
**41**	4624.3147	2312.6610	4607.2882	2304.1477	4606.3041	2303.6557	**W**	***532.2627***	266.6350	515.2361	258.1217			**4**
**42**	4738.3576	2369.6825	4721.3311	2361.1692	4720.3471	2360.6772	**N**	346.1833	173.5953	329.1568	165.0820			**3**
**43**	4795.3791	2398.1932	4778.3526	2389.6799	4777.3685	2389.1879	**G**	232.1404	116.5738	215.1139	108.0606			**2**
**44**							**R**	175.1190	88.0631	158.0924	79.5498			**1**

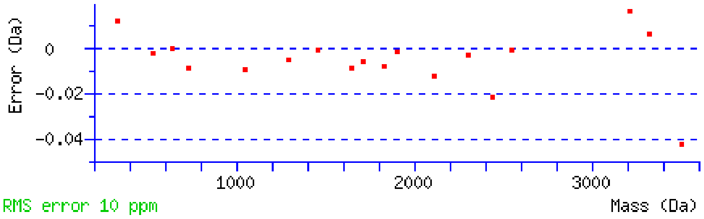

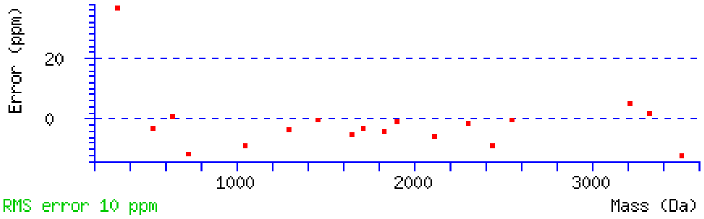

NCBI **BLAST** search of EGFGHLSPTGTTEFWLGNEKIHLISTQSAIPYALRVELEDWNGR
(Parameters: blastp, nr protein database, expect=20000, no filter, PAM30)
Other BLAST web gateways

All matches to this query

Score	Mr(calc)	Delta	Sequence
52.0	4968.4835	0.0046	EGFGHLSPTGTTEFWLGNEKIHLISTQSAIPYALRVELEDWNGR

**Mascot:** http://www.matrixscience.com/

Mascot Search Results

Peptide View

MS/MS Fragmentation of **IHLISTQSAIPYALRVELEDWNGR**
Found in **P02679** in **uni_human_nr**, FIBG_HUMAN Fibrinogen gamma chain OS=Homo sapiens GN=FGG PE=1 SV=3

Match to Query 16382: 2781.438456 from(696.366890,4+) intensity(0.0000) rtinseconds(3071) scans(20267) index(16663)
Title: 130809_HPL_Human_Plaque_BR2_TR3_03_Spectrum040451_scans__20267_RTINSECONDS=3071
Data file E:\\RAW\\HumanPlaques\\mgf\\T\\1D\\T130809_HPL_Human_Plaque_BR2_TR3_03.mgf

Click mouse within plot area to zoom in by factor of two about that point
Or, [Plot from] [0] to [2000] Da [Full range]
Label all possible matches ○ Label matches used for scoring ⊙
Show Y-axis ☐

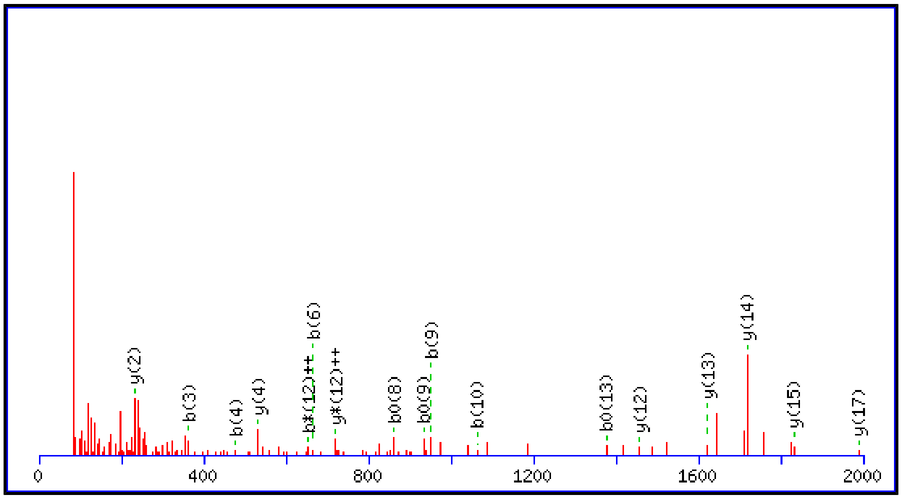

Monoisotopic mass of neutral peptide Mr(calc): 2781.4449
Fixed modifications: Carbamidomethyl (C) (apply to specified residues or termini only)
Variable modifications:
N22 : Deamidated (NQ)
Ions Score: 30 **Expect:** 0.064
Matches : 17/248 fragment ions using 35 most intense peaks (help)

#	b	b^{++}	b*	b^{*++}	b^0	b^{0++}	Seq.	y	y^{++}	y*	y^{*++}	y^0	y^{0++}	#
1	114.0913	57.5493					I							24
2	251.1503	126.0788					H	2669.3682	1335.1877	2652.3416	1326.6745	2651.3576	1326.1825	23
3	***364.2343***	182.6208					L	2532.3093	1266.6583	2515.2827	1258.1450	2514.2987	1257.6530	22
4	***477.3184***	239.1628					I	2419.2252	1210.1162	2402.1987	1201.6030	2401.2147	1201.1110	21
5	564.3504	282.6788			546.3398	273.6736	S	2306.1412	1153.5742	2289.1146	1145.0609	2288.1306	1144.5689	20
6	***665.3981***	333.2027			647.3875	324.1974	T	2219.1091	1110.0582	2202.0826	1101.5449	2201.0986	1101.0529	19
7	793.4567	397.2320	776.4301	388.7187	775.4461	388.2267	Q	2118.0614	1059.5344	2101.0349	1051.0211	2100.0509	1050.5291	18
8	880.4887	440.7480	863.4621	432.2347	862.4781	431.7427	S	***1990.0029***	995.5051	1972.9763	986.9918	1971.9923	986.4998	17
9	***951.5258***	476.2665	934.4993	467.7533	933.5152	467.2613	A	1902.9708	951.9891	1885.9443	943.4758	1884.9603	942.9838	16
10	***1064.6099***	532.8086	1047.5833	524.2953	1046.5993	523.8033	I	***1831.9337***	916.4705	1814.9072	907.9572	1813.9232	907.4652	15
11	1161.6626	581.3350	1144.6361	572.8217	1143.6521	572.3297	P	***1718.8497***	859.9285	1701.8231	851.4152	1700.8391	850.9232	14
12	1324.7260	662.8666	1307.6994	654.3533	1306.7154	653.8613	Y	***1621.7969***	811.4021	1604.7703	802.8888	1603.7863	802.3968	13
13	1395.7631	698.3852	1378.7365	689.8719	1377.7525	689.3799	A	***1458.7336***	729.8704	1441.7070	721.3571	1440.7230	720.8651	12
14	1508.8471	754.9272	1491.8206	746.4139	1490.8366	745.9219	L	1387.6965	694.3519	1370.6699	685.8386	1369.6859	685.3466	11
15	1664.9483	832.9778	1647.9217	824.4645	1646.9377	823.9725	R	1274.6124	637.8098	1257.5858	629.2966	1256.6018	628.8046	10
16	1764.0167	882.5120	1746.9901	873.9987	1746.0061	873.5067	V	1118.5113	559.7593	1101.4847	551.2460	1100.5007	550.7540	9
17	1893.0593	947.0333	1876.0327	938.5200	1875.0487	938.0280	E	1019.4429	510.2251	1002.4163	501.7118	1001.4323	501.2198	8
18	2006.1433	1003.5753	1989.1168	995.0620	1988.1328	994.5700	L	890.4003	445.7038	873.3737	437.1905	872.3897	436.6985	7

**19**	2135.1859	1068.0966	2118.1594	1059.5833	2117.1754	1059.0913	**E**	777.3162	389.1617	760.2897	380.6485	759.3056	380.1565	**6**
**20**	2250.2129	1125.6101	2233.1863	1117.0968	2232.2023	1116.6048	**D**	648.2736	324.6404	631.2471	316.1272	630.2630	315.6352	**5**
**21**	2436.2922	1218.6497	2419.2656	1210.1364	2418.2816	1209.6444	**W**	***533.2467***	267.1270	516.2201	258.6137			**4**
**22**	2551.3191	1276.1632	2534.2926	1267.6499	2533.3086	1267.1579	**N**	347.1674	174.0873	330.1408	165.5740			**3**
**23**	2608.3406	1304.6739	2591.3140	1296.1607	2590.3300	1295.6686	**G**	***232.1404***	116.5738	215.1139	108.0606			**2**
**24**							**R**	175.1190	88.0631	158.0924	79.5498			**1**

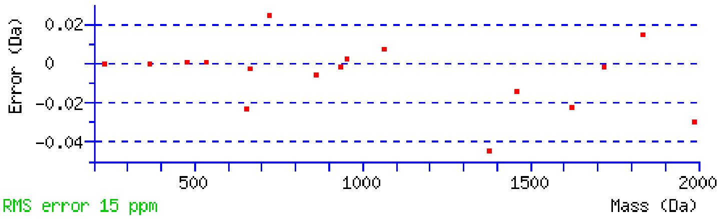

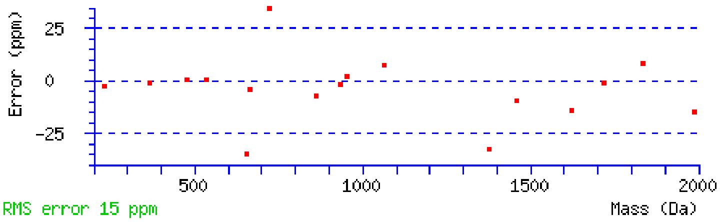

NCBI **BLAST** search of IHLISTQSAIPYALRVELEDWNGR
(Parameters: blastp, nr protein database, expect=20000, no filter, PAM30)
Other BLAST web gateways

All matches to this query

Score	Mr(calc)	Delta	Sequence
30.3	2781.4449	-0.0065	IHLISTQSAIPYALRVELEDWNGR

Mascot: http://www.matrixscience.com/

Mascot Search Results

Peptide View

MS/MS Fragmentation of **IHLISTQSAIPYALRVELEDWNGR**
Found in **P02679** in **uni_human_nr**, FIBG_HUMAN Fibrinogen gamma chain OS=Homo sapiens GN=FGG PE=1 SV=3

Match to Query 16838: 2780.463642 from(927.828490,3+) intensity(22824230.0000) rtinseconds(2879) scans(19863) index(16264)
Title: 130809_HPL_Human_Plaque_BR2_TR3_02_Spectrum041117_scans__19863_RTINSECONDS=2879
Data file E:\\RAW\\HumanPlaques\\mgf\\T\\1D\\T130809_HPL_Human_Plaque_BR2_TR3_02.mgf

Click mouse within plot area to zoom in by factor of two about that point
Or, [Plot from] 0 to 2800 Da [Full range]
Label all possible matches ○ Label matches used for scoring ⊙
Show Y-axis ☐

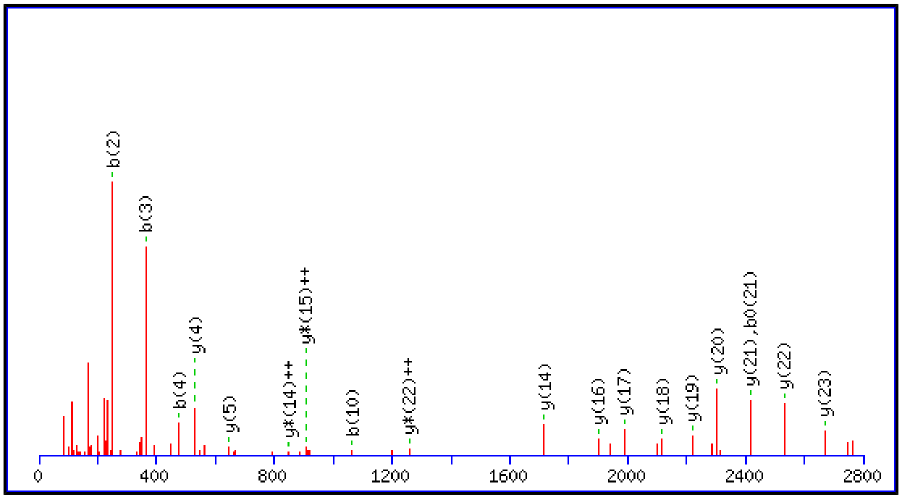

Monoisotopic mass of neutral peptide Mr(calc): 2780.4609
Fixed modifications: Carbamidomethyl (C) (apply to specified residues or termini only)
Ions Score: 97 **Expect:** 1.2e-008
Matches : 19/248 fragment ions using 20 most intense peaks (help)

#	b	b^{++}	b*	b^{*++}	b^0	b^{0++}	Seq.	y	y^{++}	y*	y^{*++}	y^0	y^{0++}	#
1	114.0913	57.5493					I							24
2	251.1503	126.0788					H	2668.3842	1334.6957	2651.3576	1326.1825	2650.3736	1325.6904	23
3	364.2343	182.6208					L	2531.3253	1266.1663	2514.2987	1257.6530	2513.3147	1257.1610	22
4	477.3184	239.1628					I	2418.2412	1209.6242	2401.2147	1201.1110	2400.2306	1200.6190	21
5	564.3504	282.6788			546.3398	273.6736	S	2305.1571	1153.0822	2288.1306	1144.5689	2287.1466	1144.0769	20
6	665.3981	333.2027			647.3875	324.1974	T	2218.1251	1109.5662	2201.0986	1101.0529	2200.1145	1100.5609	19
7	793.4567	397.2320	776.4301	388.7187	775.4461	388.2267	Q	2117.0774	1059.0424	2100.0509	1050.5291	2099.0669	1050.0371	18
8	880.4887	440.7480	863.4621	432.2347	862.4781	431.7427	S	1989.0189	995.0131	1971.9923	986.4998	1971.0083	986.0078	17
9	951.5258	476.2665	934.4993	467.7533	933.5152	467.2613	A	1901.9868	951.4970	1884.9603	942.9838	1883.9763	942.4918	16
10	1064.6099	532.8086	1047.5833	524.2953	1046.5993	523.8033	I	1830.9497	915.9785	1813.9232	907.4652	1812.9391	906.9732	15
11	1161.6626	581.3350	1144.6361	572.8217	1143.6521	572.3297	P	1717.8656	859.4365	1700.8391	850.9232	1699.8551	850.4312	14
12	1324.7260	662.8666	1307.6994	654.3533	1306.7154	653.8613	Y	1620.8129	810.9101	1603.7863	802.3968	1602.8023	801.9048	13
13	1395.7631	698.3852	1378.7365	689.8719	1377.7525	689.3799	A	1457.7496	729.3784	1440.7230	720.8651	1439.7390	720.3731	12
14	1508.8471	754.9272	1491.8206	746.4139	1490.8366	745.9219	L	1386.7124	693.8599	1369.6859	685.3466	1368.7019	684.8546	11
15	1664.9483	832.9778	1647.9217	824.4645	1646.9377	823.9725	R	1273.6284	637.3178	1256.6018	628.8046	1255.6178	628.3125	10
16	1764.0167	882.5120	1746.9901	873.9987	1746.0061	873.5067	V	1117.5273	559.2673	1100.5007	550.7540	1099.5167	550.2620	9
17	1893.0593	947.0333	1876.0327	938.5200	1875.0487	938.0280	E	1018.4588	509.7331	1001.4323	501.2198	1000.4483	500.7278	8
18	2006.1433	1003.5753	1989.1168	995.0620	1988.1328	994.5700	L	889.4163	445.2118	872.3897	436.6985	871.4057	436.2065	7
19	2135.1859	1068.0966	2118.1594	1059.5833	2117.1754	1059.0913	E	776.3322	388.6697	759.3056	380.1565	758.3216	379.6645	6

**20**	2250.2129	1125.6101	2233.1863	1117.0968	2232.2023	1116.6048	**D**	***647.2896***	324.1484	630.2630	315.6352	629.2790	315.1432	**5**
**21**	2436.2922	1218.6497	2419.2656	1210.1364	2418.2816	1209.6444	**W**	***532.2627***	266.6350	515.2361	258.1217			**4**
**22**	2550.3351	1275.6712	2533.3086	1267.1579	2532.3245	1266.6659	**N**	346.1833	173.5953	329.1568	165.0820			**3**
**23**	2607.3566	1304.1819	2590.3300	1295.6686	2589.3460	1295.1766	**G**	232.1404	116.5738	215.1139	108.0606			**2**
**24**							**R**	175.1190	88.0631	158.0924	79.5498			**1**

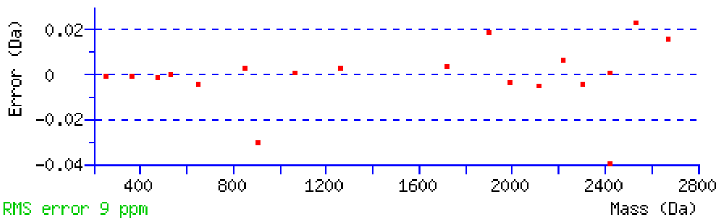

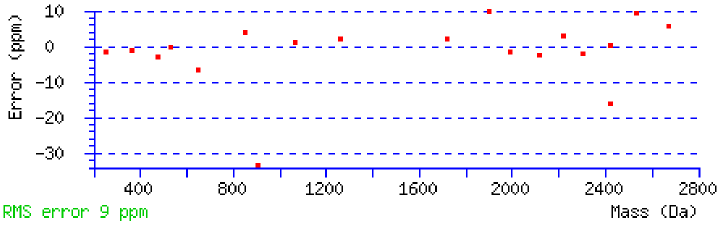

NCBI **BLAST** search of IHLISTQSAIPYALRVELEDWNGR
(Parameters: blastp, nr protein database, expect=20000, no filter, PAM30)
Other BLAST web gateways

All matches to this query

Score	Mr(calc)	Delta	Sequence
96.7	2780.4609	0.0027	IHLISTQSAIPYALRVELEDWNGR
7.2	2780.4677	-0.0040	HILLRPTMQTLAPEVKTMTRDGEK
6.9	2780.4677	-0.0040	HILLRPTMQTLAPEVKTMTRDGEK

Mascot: http://www.matrixscience.com/

Mascot Search Results

Peptide View

MS/MS Fragmentation of **IHLISTQSAIPYALRVELEDWNGR**
Found in **P02679** in **uni_human_nr**, FIBG_HUMAN Fibrinogen gamma chain OS=Homo sapiens GN=FGG PE=1 SV=3

Match to Query 16814: 2780.461376 from(696.122620,4+) intensity(48521944.0000) rtinseconds(2907) scans(19975) index(16367)
Title: 130806_HPL_Human_Plaque_BR2_TR2_02_Spectrum041200_scans__19975_RTINSECONDS=2907
Data file E:\\RAW\\HumanPlaques\\mgf\\T\\1D\\T130806_HPL_Human_Plaque_BR2_TR2_02.mgf

Click mouse within plot area to zoom in by factor of two about that point
Or, [Plot from] [0] to [2000] Da [Full range]
Label all possible matches ○ Label matches used for scoring ⦿
Show Y-axis ☐

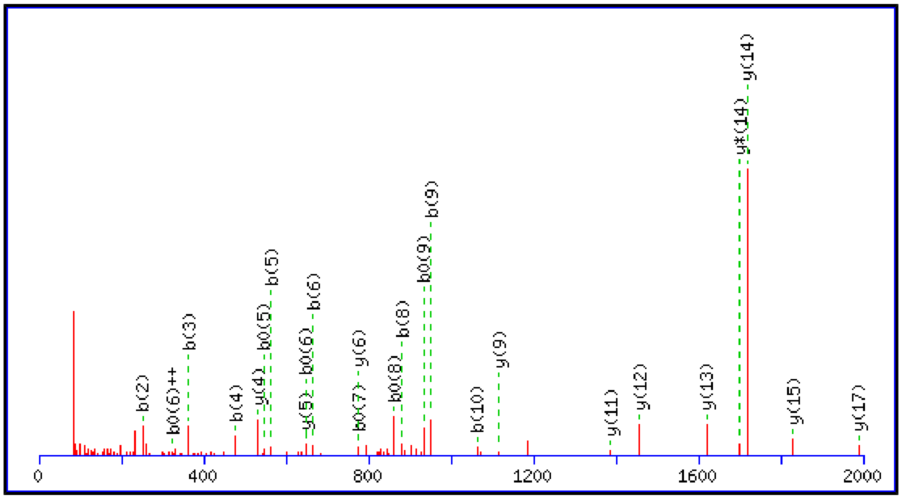

Monoisotopic mass of neutral peptide Mr(calc): 2780.4609
Fixed modifications: Carbamidomethyl (C) (apply to specified residues or termini only)
Ions Score: 86 **Expect:** 1.6e-007
Matches : 25/248 fragment ions using 39 most intense peaks (help)

#	b	b^{++}	b*	b^{*++}	b^0	b^{0++}	Seq.	y	y^{++}	y*	y^{*++}	y^0	y^{0++}	#
1	114.0913	57.5493					I							24
2	251.1503	126.0788					H	2668.3842	1334.6957	2651.3576	1326.1825	2650.3736	1325.6904	23
3	364.2343	182.6208					L	2531.3253	1266.1663	2514.2987	1257.6530	2513.3147	1257.1610	22
4	477.3184	239.1628					I	2418.2412	1209.6242	2401.2147	1201.1110	2400.2306	1200.6190	21
5	564.3504	282.6788			546.3398	273.6736	S	2305.1571	1153.0822	2288.1306	1144.5689	2287.1466	1144.0769	20
6	665.3981	333.2027			647.3875	324.1974	T	2218.1251	1109.5662	2201.0986	1101.0529	2200.1145	1100.5609	19
7	793.4567	397.2320	776.4301	388.7187	775.4461	388.2267	Q	2117.0774	1059.0424	2100.0509	1050.5291	2099.0669	1050.0371	18
8	880.4887	440.7480	863.4621	432.2347	862.4781	431.7427	S	1989.0189	995.0131	1971.9923	986.4998	1971.0083	986.0078	17
9	951.5258	476.2665	934.4993	467.7533	933.5152	467.2613	A	1901.9868	951.4970	1884.9603	942.9838	1883.9763	942.4918	16
10	1064.6099	532.8086	1047.5833	524.2953	1046.5993	523.8033	I	1830.9497	915.9785	1813.9232	907.4652	1812.9391	906.9732	15
11	1161.6626	581.3350	1144.6361	572.8217	1143.6521	572.3297	P	1717.8656	859.4365	1700.8391	850.9232	1699.8551	850.4312	14
12	1324.7260	662.8666	1307.6994	654.3533	1306.7154	653.8613	Y	1620.8129	810.9101	1603.7863	802.3968	1602.8023	801.9048	13
13	1395.7631	698.3852	1378.7365	689.8719	1377.7525	689.3799	A	1457.7496	729.3784	1440.7230	720.8651	1439.7390	720.3731	12
14	1508.8471	754.9272	1491.8206	746.4139	1490.8366	745.9219	L	1386.7124	693.8599	1369.6859	685.3466	1368.7019	684.8546	11
15	1664.9483	832.9778	1647.9217	824.4645	1646.9377	823.9725	R	1273.6284	637.3178	1256.6018	628.8046	1255.6178	628.3125	10
16	1764.0167	882.5120	1746.9901	873.9987	1746.0061	873.5067	V	1117.5273	559.2673	1100.5007	550.7540	1099.5167	550.2620	9
17	1893.0593	947.0333	1876.0327	938.5200	1875.0487	938.0280	E	1018.4588	509.7331	1001.4323	501.2198	1000.4483	500.7278	8
18	2006.1433	1003.5753	1989.1168	995.0620	1988.1328	994.5700	L	889.4163	445.2118	872.3897	436.6985	871.4057	436.2065	7
19	2135.1859	1068.0966	2118.1594	1059.5833	2117.1754	1059.0913	E	776.3322	388.6697	759.3056	380.1565	758.3216	379.6645	6

**20**	2250.2129	1125.6101	2233.1863	1117.0968	2232.2023	1116.6048	**D**	***647.2896***	324.1484	630.2630	315.6352	629.2790	315.1432	**5**
**21**	2436.2922	1218.6497	2419.2656	1210.1364	2418.2816	1209.6444	**W**	***532.2627***	266.6350	515.2361	258.1217			**4**
**22**	2550.3351	1275.6712	2533.3086	1267.1579	2532.3245	1266.6659	**N**	346.1833	173.5953	329.1568	165.0820			**3**
**23**	2607.3566	1304.1819	2590.3300	1295.6686	2589.3460	1295.1766	**G**	232.1404	116.5738	215.1139	108.0606			**2**
**24**							**R**	175.1190	88.0631	158.0924	79.5498			**1**

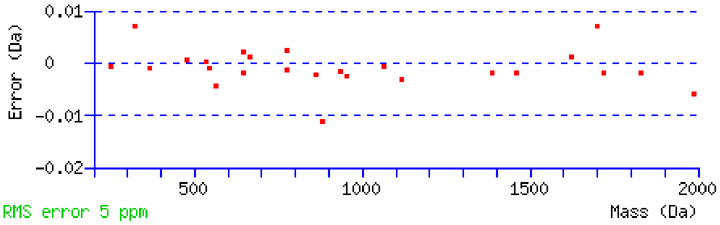

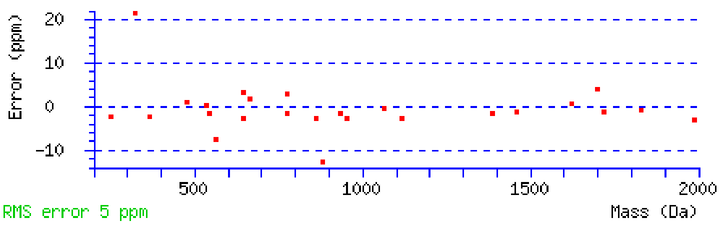

NCBI **BLAST** search of IHLISTQSAIPYALRVELEDWNGR
(Parameters: blastp, nr protein database, expect=20000, no filter, PAM30)
Other BLAST web gateways

All matches to this query

Score	Mr(calc)	Delta	Sequence
85.8	2780.4609	0.0005	IHLISTQSAIPYALRVELEDWNGR

**Mascot:** http://www.matrixscience.com/

Mascot Search Results

Peptide View

MS/MS Fragmentation of **IHLISTQSAIPYALRVELEDWNGR**
Found in **P02679** in **uni_human_nr**, FIBG_HUMAN Fibrinogen gamma chain OS=Homo sapiens GN=FGG PE=1 SV=3

Match to Query 16815: 2780.468848 from(1391.241700,2+) intensity(1615435.0000) rtinseconds(2906) scans(19968) index(16361)
Title: 130806_HPL_Human_Plaque_BR2_TR2_02_Spectrum041194_scans__19968_RTINSECONDS=2906
Data file E:\\RAW\\HumanPlaques\\mgf\\T\\1D\\T130806_HPL_Human_Plaque_BR2_TR2_02.mgf

Click mouse within plot area to zoom in by factor of two about that point
Or, Plot from 0 to 2800 Da Full range
Label all possible matches ○ Label matches used for scoring ⊙
Show Y-axis ☐

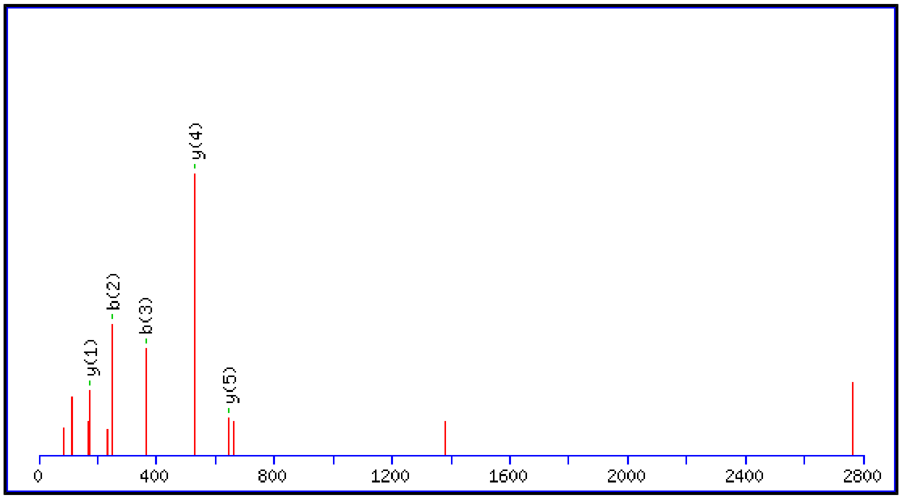

Monoisotopic mass of neutral peptide Mr(calc): 2780.4609
Fixed modifications: Carbamidomethyl (C) (apply to specified residues or termini only)
Ions Score: 23 **Expect:** 0.26
Matches : 5/248 fragment ions using 7 most intense peaks (help)

#	b	b^{++}	b*	b^{*++}	b^0	b^{0++}	Seq.	y	y^{++}	y*	y^{*++}	y^0	y^{0++}	#
1	114.0913	57.5493					I							24
2	***251.1503***	126.0788					H	2668.3842	1334.6957	2651.3576	1326.1825	2650.3736	1325.6904	23
3	***364.2343***	182.6208					L	2531.3253	1266.1663	2514.2987	1257.6530	2513.3147	1257.1610	22
4	477.3184	239.1628					I	2418.2412	1209.6242	2401.2147	1201.1110	2400.2306	1200.6190	21
5	564.3504	282.6788			546.3398	273.6736	S	2305.1571	1153.0822	2288.1306	1144.5689	2287.1466	1144.0769	20
6	665.3981	333.2027			647.3875	324.1974	T	2218.1251	1109.5662	2201.0986	1101.0529	2200.1145	1100.5609	19
7	793.4567	397.2320	776.4301	388.7187	775.4461	388.2267	Q	2117.0774	1059.0424	2100.0509	1050.5291	2099.0669	1050.0371	18
8	880.4887	440.7480	863.4621	432.2347	862.4781	431.7427	S	1989.0189	995.0131	1971.9923	986.4998	1971.0083	986.0078	17
9	951.5258	476.2665	934.4993	467.7533	933.5152	467.2613	A	1901.9868	951.4970	1884.9603	942.9838	1883.9763	942.4918	16
10	1064.6099	532.8086	1047.5833	524.2953	1046.5993	523.8033	I	1830.9497	915.9785	1813.9232	907.4652	1812.9391	906.9732	15
11	1161.6626	581.3350	1144.6361	572.8217	1143.6521	572.3297	P	1717.8656	859.4365	1700.8391	850.9232	1699.8551	850.4312	14
12	1324.7260	662.8666	1307.6994	654.3533	1306.7154	653.8613	Y	1620.8129	810.9101	1603.7863	802.3968	1602.8023	801.9048	13
13	1395.7631	698.3852	1378.7365	689.8719	1377.7525	689.3799	A	1457.7496	729.3784	1440.7230	720.8651	1439.7390	720.3731	12
14	1508.8471	754.9272	1491.8206	746.4139	1490.8366	745.9219	L	1386.7124	693.8599	1369.6859	685.3466	1368.7019	684.8546	11
15	1664.9483	832.9778	1647.9217	824.4645	1646.9377	823.9725	R	1273.6284	637.3178	1256.6018	628.8046	1255.6178	628.3125	10
16	1764.0167	882.5120	1746.9901	873.9987	1746.0061	873.5067	V	1117.5273	559.2673	1100.5007	550.7540	1099.5167	550.2620	9
17	1893.0593	947.0333	1876.0327	938.5200	1875.0487	938.0280	E	1018.4588	509.7331	1001.4323	501.2198	1000.4483	500.7278	8
18	2006.1433	1003.5753	1989.1168	995.0620	1988.1328	994.5700	L	889.4163	445.2118	872.3897	436.6985	871.4057	436.2065	7
19	2135.1859	1068.0966	2118.1594	1059.5833	2117.1754	1059.0913	E	776.3322	388.6697	759.3056	380.1565	758.3216	379.6645	6

**20**	2250.2129	1125.6101	2233.1863	1117.0968	2232.2023	1116.6048	**D**	***647.2896***	324.1484	630.2630	315.6352	629.2790	315.1432	**5**
**21**	2436.2922	1218.6497	2419.2656	1210.1364	2418.2816	1209.6444	**W**	***532.2627***	266.6350	515.2361	258.1217			**4**
**22**	2550.3351	1275.6712	2533.3086	1267.1579	2532.3245	1266.6659	**N**	346.1833	173.5953	329.1568	165.0820			**3**
**23**	2607.3566	1304.1819	2590.3300	1295.6686	2589.3460	1295.1766	**G**	232.1404	116.5738	215.1139	108.0606			**2**
**24**							**R**	***175.1190***	88.0631	158.0924	79.5498			**1**

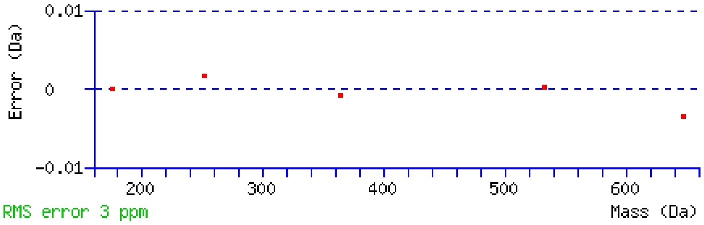

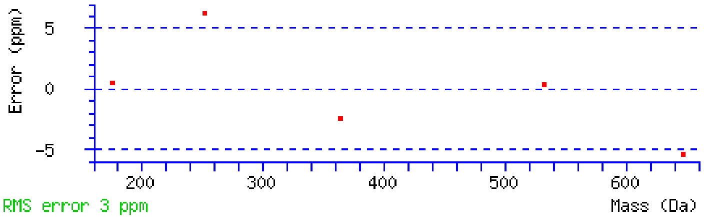

NCBI **BLAST** search of IHLISTQSAIPYALRVELEDWNGR
(Parameters: blastp, nr protein database, expect=20000, no filter, PAM30)
Other BLAST web gateways

All matches to this query

Score	Mr(calc)	Delta	Sequence
23.0	2780.4609	0.0079	IHLISTQSAIPYALRVELEDWNGR
6.3	2778.4525	2.0163	HLLSGGTWTQAGQGQLRQVLSRDIR
6.3	2778.4525	2.0163	HLLSGGTWTQAGQGQLRQVLSRDIR
6.3	2778.4525	2.0163	HLLSGGTWTQAGQGQLRQVLSRDIR
5.3	2780.4685	0.0003	SYLVFWTVSNLMAFLIMVVVYLR
5.3	2780.4685	0.0003	SYLVFWTVSNLMAFLIMVVVYLR
4.4	2778.4511	2.0177	TEQLVRLESNAVIENKTIAHNLQR
4.4	2778.4511	2.0177	TEQLVRLESNAVIENKTIAHNLQR
3.7	2779.4585	1.0103	VFVWDLDETIIIFHSLLTGTFASR
3.6	2780.4820	-0.0132	QALAVGNKIVLHTEQKQQLFAATNR

Mascot: http://www.matrixscience.com/

Mascot Search Results

Peptide View

MS/MS Fragmentation of **VELEDWNGR**
Found in **P02679** in **uni_human_nr**, FIBG_HUMAN Fibrinogen gamma chain OS=Homo sapiens GN=FGG PE=1 SV=3

Match to Query 726: 1117.504128 from(559.759340,2+) intensity(19995396.0000) rtinseconds(1862) scans(9366) index(6465)
Title: 130801_HPL_Human_Plaque_BR1_TR1_16_Spectrum026193_scans__9366_RTINSECONDS=1862
Data file E:\\RAW\\HumanPlaques\\mgf\\T\\1D\\T130801_HPL_Human_Plaque_BR1_TR1_16.mgf

Click mouse within plot area to zoom in by factor of two about that point
Or, Plot from 50 to 1050 Da Full range
Label all possible matches ○ Label matches used for scoring ⦿
Show Y-axis ☐

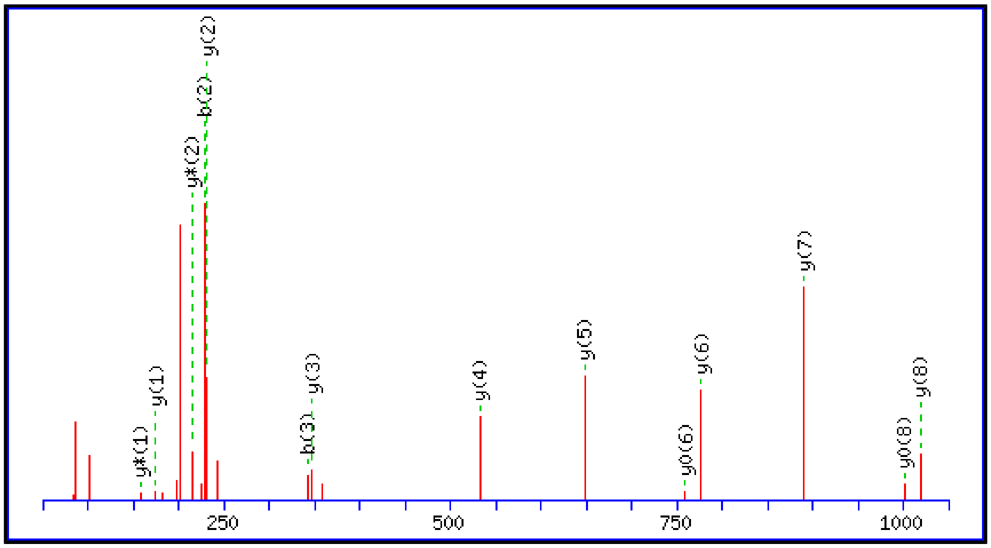

Monoisotopic mass of neutral peptide Mr(calc): 1117.5040
Fixed modifications: Carbamidomethyl (C) (apply to specified residues or termini only)
Variable modifications:
N7 : Deamidated (NQ)
Ions Score: 75 **Expect:** 8.1e-007
Matches : 14/74 fragment ions using 19 most intense peaks (help)

#	b	b^{++}	b*	b^{*++}	b^0	b^{0++}	Seq.	y	y^{++}	y*	y^{*++}	y^0	y^{0++}	#
1	100.0757	50.5415					V							9
2	**229.1183**	115.0628			211.1077	106.0575	E	***1019.4429***	510.2251	1002.4163	501.7118	1001.4323	501.2198	8
3	**342.2023**	171.6048			324.1918	162.5995	L	***890.4003***	445.7038	873.3737	437.1905	872.3897	436.6985	7
4	471.2449	236.1261			453.2344	227.1208	E	***777.3162***	389.1617	760.2897	380.6485	759.3056	380.1565	6
5	586.2719	293.6396			568.2613	284.6343	D	***648.2736***	324.6404	631.2471	316.1272	630.2631	315.6352	5
6	772.3512	386.6792			754.3406	377.6740	W	***533.2467***	267.1270	516.2201	258.6137			4
7	887.3781	444.1927	870.3516	435.6794	869.3676	435.1874	N	***347.1674***	174.0873	330.1408	165.5740			3
8	944.3996	472.7034	927.3731	464.1902	926.3890	463.6982	G	***232.1404***	116.5738	215.1139	108.0606			2
9							R	***175.1190***	88.0631	158.0924	79.5498			1

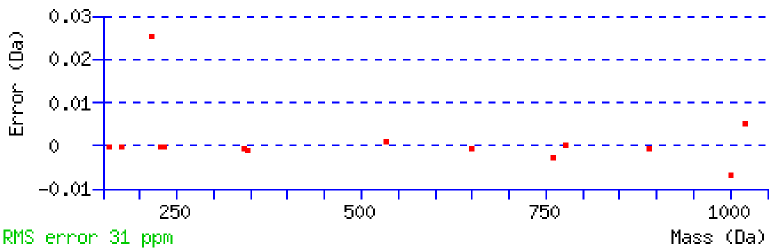

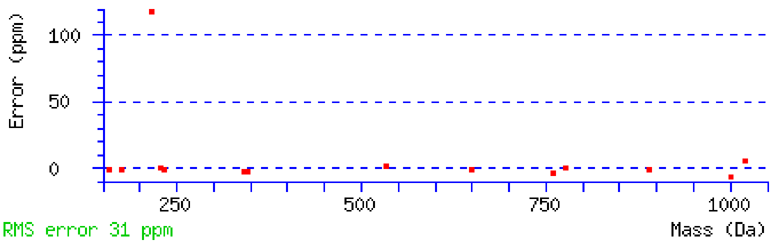

NCBI **BLAST** search of VELEDWNGR
(Parameters: blastp, nr protein database, expect=20000, no filter, PAM30)
Other BLAST web gateways

All matches to this query

Score	Mr(calc)	Delta	Sequence
74.7	1117.5040	0.0001	VELEDWNGR
9.9	1115.5029	2.0012	NPQQNMNIR
6.0	1115.4956	2.0086	GPDSQRQDGR
3.7	1117.5000	0.0041	VGATDPNSGSGR
2.9	1115.5029	2.0012	NPQQNMNIR
2.9	1115.5029	2.0012	NPQQNMNIR
2.1	1115.4956	2.0086	GPDSQRQDGR
2.0	1117.5053	-0.0012	GRWANDGWR
0.4	1116.5056	0.9985	VIQGCCGNIP

Mascot: http://www.matrixscience.com/

Mascot Search Results

Peptide View

MS/MS Fragmentation of **VELEDWNGR**
Found in **P02679** in **uni_human_nr**, FIBG_HUMAN Fibrinogen gamma chain OS=Homo sapiens GN=FGG PE=1 SV=3

Match to Query 873: 1116.520108 from(559.267330,2+) intensity(25080110.0000) rtinseconds(1700) scans(8606) index(6060)
Title: 130801_HPL_Human_Plaque_BR2_TR1_09_Spectrum026872_scans__8606_RTINSECONDS=1700
Data file E:\\RAW\\HumanPlaques\\mgf\\T\\1D\\T130801_HPL_Human_Plaque_BR2_TR1_09.mgf

Click mouse within plot area to zoom in by factor of two about that point
Or, Plot from 50 to 1050 Da Full range
Label all possible matches ○ Label matches used for scoring ⦿
Show Y-axis ☐

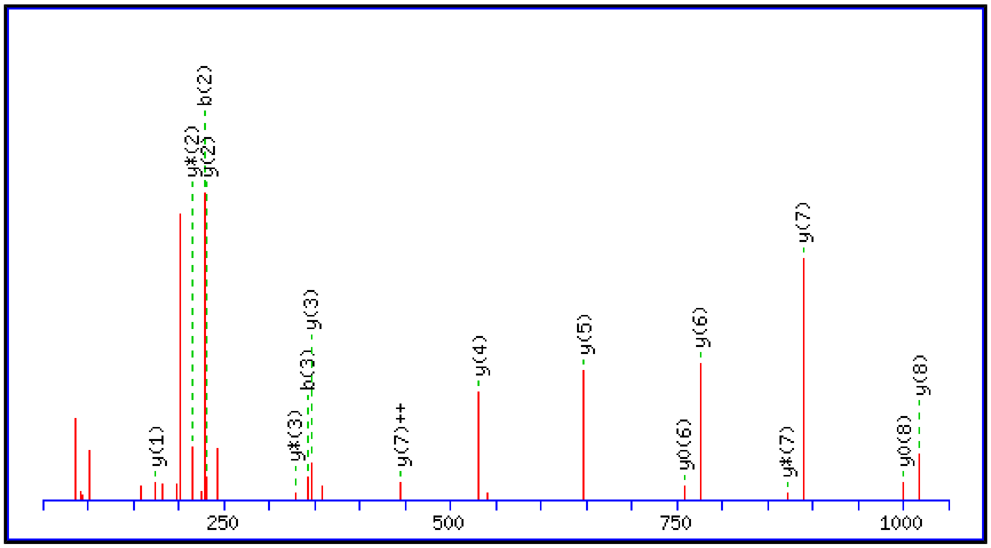

Monoisotopic mass of neutral peptide Mr(calc): 1116.5200
Fixed modifications: Carbamidomethyl (C) (apply to specified residues or termini only)
Ions Score: 68 **Expect:** 5e-006
Matches : 16/74 fragment ions using 24 most intense peaks (help)

#	b	b^{++}	b*	b^{*++}	b^0	b^{0++}	Seq.	y	y^{++}	y*	y^{*++}	y^0	y^{0++}	#
1	100.0757	50.5415					V							9
2	**229.1183**	115.0628			211.1077	106.0575	E	***1018.4588***	509.7331	1001.4323	501.2198	1000.4483	500.7278	8
3	**342.2023**	171.6048			324.1918	162.5995	L	***889.4163***	445.2118	872.3897	436.6985	871.4057	436.2065	7
4	471.2449	236.1261			453.2344	227.1208	E	***776.3322***	388.6697	759.3056	380.1565	758.3216	379.6645	6
5	586.2719	293.6396			568.2613	284.6343	D	***647.2896***	324.1484	630.2631	315.6352	629.2790	315.1432	5
6	772.3512	386.6792			754.3406	377.6740	W	***532.2627***	266.6350	515.2361	258.1217			4
7	886.3941	443.7007	869.3676	435.1874	868.3836	434.6954	N	***346.1833***	173.5953	329.1568	165.0820			3
8	943.4156	472.2114	926.3890	463.6982	925.4050	463.2061	G	***232.1404***	116.5738	215.1139	108.0606			2
9							R	***175.1190***	88.0631	158.0924	79.5498			1

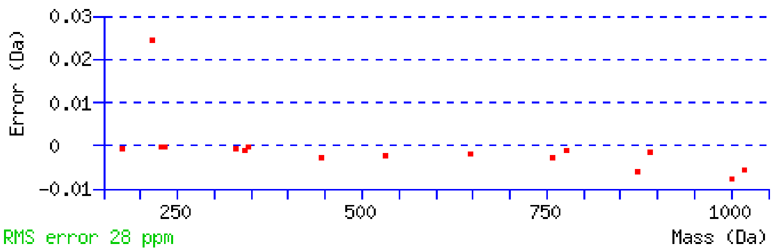

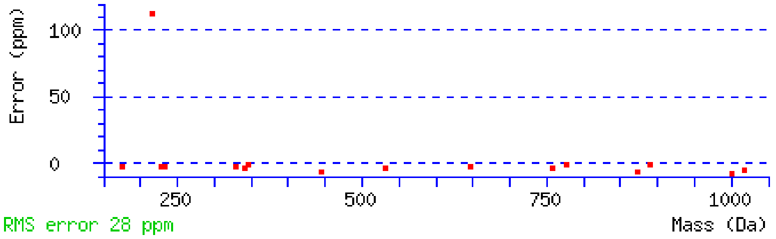

NCBI **BLAST** search of VELEDWNGR
(Parameters: blastp, nr protein database, expect=20000, no filter, PAM30)
Other BLAST web gateways

All matches to this query

Score	Mr(calc)	Delta	Sequence
67.6	1116.5200	0.0001	VELEDWNGR
13.2	1114.5116	2.0085	RGTPGDADGGGR
12.5	1116.5161	0.0040	MPSEYTYVK
3.4	1115.5142	1.0059	NHMSVSNVGR
2.2	1116.5160	0.0041	VGATDPNSGSGR
0.6	1116.5161	0.0040	TFMQGEFLK
0.4	1115.5207	0.9994	GNLEIGGDAGGR
0.3	1116.5199	0.0002	AEEIEHYAR

Mascot: http://www.matrixscience.com/

Mascot Search Results

Peptide View

MS/MS Fragmentation of **VELEDWNGRTSTADYAMFK**
Found in **P02679** in **uni_human_nr**, FIBG_HUMAN Fibrinogen gamma chain OS=Homo sapiens GN=FGG PE=1 SV=3

Match to Query 9058: 2248.994712 from(750.672180,3+) intensity(880264.6875) rtinseconds(2393) scans(13381) index(10107)
Title: 130809_HPL_Human_Plaque_BR2_TR3_14_Spectrum030776_scans__13381_RTINSECONDS=2393
Data file E:\\RAW\\HumanPlaques\\mgf\\T\\1D\\T130809_HPL_Human_Plaque_BR2_TR3_14.mgf

Click mouse within plot area to zoom in by factor of two about that point
Or, [Plot from] [0] to [2200] Da [Full range]
Label all possible matches ○ Label matches used for scoring ⦿
Show Y-axis ☐

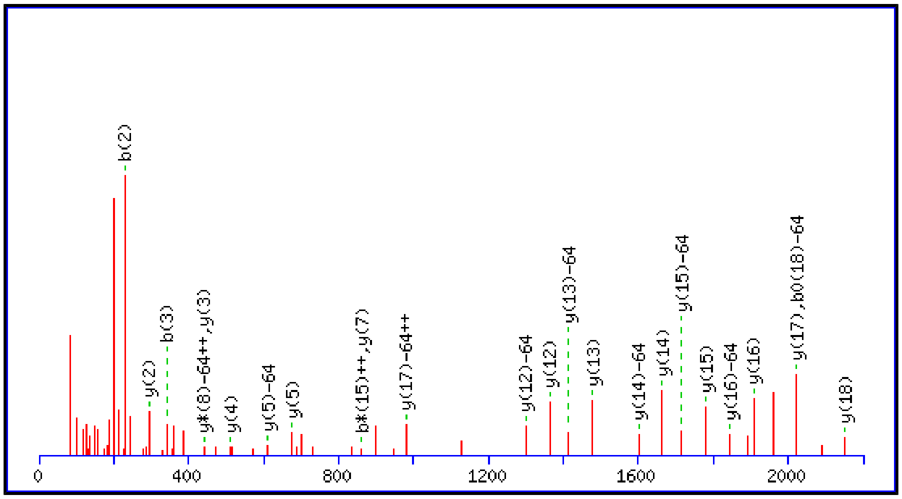

Monoisotopic mass of neutral peptide Mr(calc): 2248.9946
Fixed modifications: Carbamidomethyl (C) (apply to specified residues or termini only)
Variable modifications:
N7 : Deamidated (NQ)
M17 : Oxidation (M), with neutral losses 0.0000(shown in table), 63.9983
Ions Score: 85 **Expect:** 1.5e-007
Matches : 24/294 fragment ions using 33 most intense peaks (help)

#	b	b^{++}	b*	b^{*++}	b^0	b^{0++}	Seq.	y	y^{++}	y*	y^{*++}	y^0	y^{0++}	#
1	100.0757	50.5415					V							19
2	**229.1183**	115.0628			211.1077	106.0575	E	***2150.9335***	1075.9704	2133.9070	1067.4571	2132.9230	1066.9651	18
3	**342.2023**	171.6048			324.1918	162.5995	L	***2021.8909***	1011.4491	2004.8644	1002.9358	2003.8804	1002.4438	17
4	471.2449	236.1261			453.2344	227.1208	E	***1908.8069***	954.9071	1891.7803	946.3938	1890.7963	945.9018	16
5	586.2719	293.6396			568.2613	284.6343	D	***1779.7643***	890.3858	1762.7377	881.8725	1761.7537	881.3805	15
6	772.3512	386.6792			754.3406	377.6740	W	***1664.7373***	832.8723	1647.7108	824.3590	1646.7268	823.8670	14
7	887.3781	444.1927	870.3516	435.6794	869.3676	435.1874	N	***1478.6580***	739.8326	1461.6315	731.3194	1460.6475	730.8274	13
8	944.3996	472.7034	927.3731	464.1902	926.3890	463.6982	G	***1363.6311***	682.3192	1346.6045	673.8059	1345.6205	673.3139	12
9	1100.5007	550.7540	1083.4742	542.2407	1082.4901	541.7487	R	1306.6096	653.8084	1289.5831	645.2952	1288.5991	644.8032	11
10	1201.5484	601.2778	1184.5218	592.7646	1183.5378	592.2726	T	1150.5085	575.7579	1133.4820	567.2446	1132.4979	566.7526	10
11	1288.5804	644.7938	1271.5539	636.2806	1270.5699	635.7886	S	1049.4608	525.2341	1032.4343	516.7208	1031.4503	516.2288	9
12	1389.6281	695.3177	1372.6016	686.8044	1371.6175	686.3124	T	962.4288	481.7180	945.4022	473.2048	944.4182	472.7128	8
13	1460.6652	730.8362	1443.6387	722.3230	1442.6546	721.8310	A	***861.3811***	431.1942	844.3546	422.6809	843.3706	422.1889	7
14	1575.6922	788.3497	1558.6656	779.8364	1557.6816	779.3444	D	790.3440	395.6756	773.3175	387.1624	772.3334	386.6704	6
15	1738.7555	869.8814	1721.7289	861.3681	1720.7449	860.8761	Y	***675.3171***	338.1622	658.2905	329.6489			5
16	1809.7926	905.3999	1792.7661	896.8867	1791.7820	896.3947	A	***512.2537***	256.6305	495.2272	248.1172			4
17	1956.8280	978.9176	1939.8015	970.4044	1938.8174	969.9124	M	***441.2166***	221.1119	424.1901	212.5987			3

**18**	2103.8964	1052.4518	2086.8699	1043.9386	2085.8858	1043.4466	**F**	*294.1812*	147.5942	277.1547	139.0810			**2**
**19**							**K**	147.1128	74.0600	130.0863	65.5468			**1**

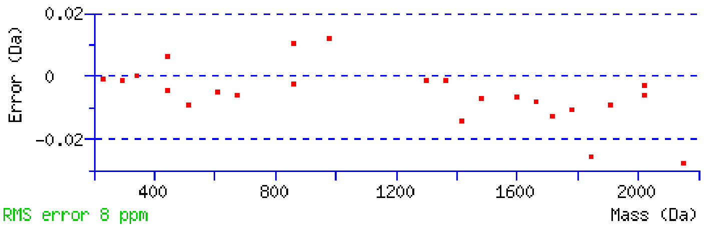

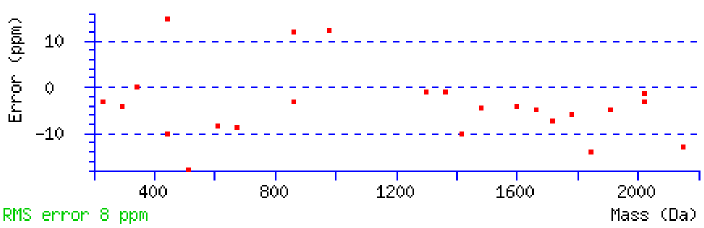

NCBI **BLAST** search of VELEDWNGRTSTADYAMFK
(Parameters: blastp, nr protein database, expect=20000, no filter, PAM30)
Other BLAST web gateways

All matches to this query

Score	Mr(calc)	Delta	Sequence
84.5	2248.9946	0.0001	VELEDWNGRTSTADYAMFK
2.3	2249.0052	-0.0105	GGARDLLEEACDQCASQLEK
0.3	2248.9915	0.0033	RGKEMVDYMANYMEGIEGR

Mascot: http://www.matrixscience.com/

Mascot Search Results

Peptide View

MS/MS Fragmentation of **VELEDWNGRTSTADYAMFK**
Found in **P02679** in **uni_human_nr**, FIBG_HUMAN Fibrinogen gamma chain OS=Homo sapiens GN=FGG PE=1 SV=3

Match to Query 8780: 2232.998802 from(745.340210,3+) intensity(6672243.0000) rtinseconds(2636) scans(15159) index(11527)
Title: 130806_HPL_Human_Plaque_BR1_TR2_12_Spectrum032646_scans__15159_RTINSECONDS=2636
Data file E:\\RAW\\HumanPlaques\\mgf\\T\\1D\\T130806_HPL_Human_Plaque_BR1_TR2_12.mgf

Click mouse within plot area to zoom in by factor of two about that point
Or, Plot from 0 to 2200 Da Full range
Label all possible matches ○ Label matches used for scoring ⊙
Show Y-axis ☐

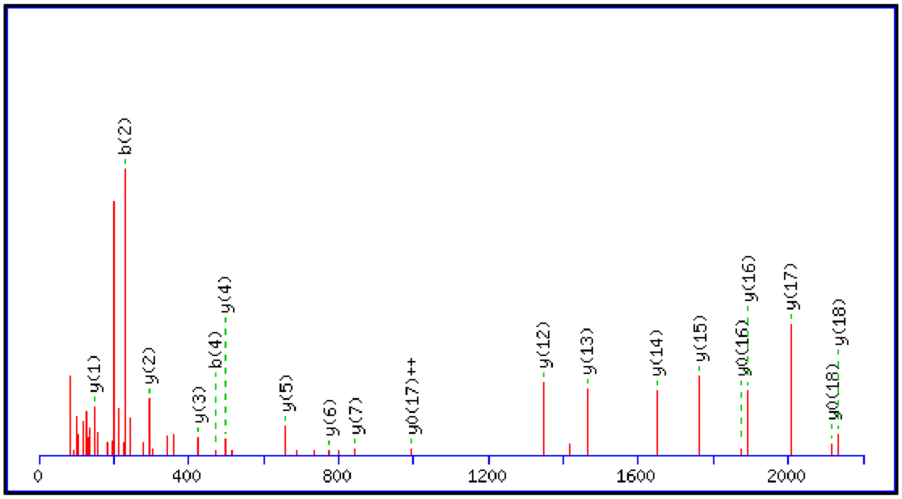

Monoisotopic mass of neutral peptide Mr(calc): 2232.9997
Fixed modifications: Carbamidomethyl (C) (apply to specified residues or termini only)
Variable modifications:
N7 : Deamidated (NQ)
Ions Score: 122 **Expect:** 3e-011
Matches : 19/192 fragment ions using 27 most intense peaks (help)

#	b	b^{++}	b*	b^{*++}	b^0	b^{0++}	Seq.	y	y^{++}	y*	y^{*++}	y^0	y^{0++}	#
1	100.0757	50.5415					V							19
2	**229.1183**	115.0628			211.1077	106.0575	E	***2134.9386***	1067.9729	2117.9121	1059.4597	2116.9280	1058.9677	18
3	342.2023	171.6048			324.1918	162.5995	L	***2005.8960***	1003.4516	1988.8695	994.9384	1987.8855	994.4464	17
4	**471.2449**	236.1261			453.2344	227.1208	E	***1892.8120***	946.9096	1875.7854	938.3963	1874.8014	937.9043	16
5	586.2719	293.6396			568.2613	284.6343	D	***1763.7694***	882.3883	1746.7428	873.8750	1745.7588	873.3830	15
6	772.3512	386.6792			754.3406	377.6740	W	***1648.7424***	824.8748	1631.7159	816.3616	1630.7319	815.8696	14
7	887.3781	444.1927	870.3516	435.6794	869.3676	435.1874	N	***1462.6631***	731.8352	1445.6366	723.3219	1444.6525	722.8299	13
8	944.3996	472.7034	927.3731	464.1902	926.3890	463.6982	G	***1347.6362***	674.3217	1330.6096	665.8084	1329.6256	665.3164	12
9	1100.5007	550.7540	1083.4742	542.2407	1082.4901	541.7487	R	1290.6147	645.8110	1273.5882	637.2977	1272.6041	636.8057	11
10	1201.5484	601.2778	1184.5218	592.7646	1183.5378	592.2726	T	1134.5136	567.7604	1117.4870	559.2472	1116.5030	558.7552	10
11	1288.5804	644.7938	1271.5539	636.2806	1270.5699	635.7886	S	1033.4659	517.2366	1016.4394	508.7233	1015.4553	508.2313	9
12	1389.6281	695.3177	1372.6016	686.8044	1371.6175	686.3124	T	946.4339	473.7206	929.4073	465.2073	928.4233	464.7153	8
13	1460.6652	730.8362	1443.6387	722.3230	1442.6546	721.8310	A	***845.3862***	423.1967	828.3597	414.6835	827.3756	414.1915	7
14	1575.6922	788.3497	1558.6656	779.8364	1557.6816	779.3444	D	***774.3491***	387.6782	757.3225	379.1649	756.3385	378.6729	6
15	1738.7555	869.8814	1721.7289	861.3681	1720.7449	860.8761	Y	***659.3221***	330.1647	642.2956	321.6514			5
16	1809.7926	905.3999	1792.7661	896.8867	1791.7820	896.3947	A	***496.2588***	248.6330	479.2323	240.1198			4
17	1940.8331	970.9202	1923.8065	962.4069	1922.8225	961.9149	M	***425.2217***	213.1145	408.1952	204.6012			3
18	2087.9015	1044.4544	2070.8749	1035.9411	2069.8909	1035.4491	F	***294.1812***	147.5942	277.1547	139.0810			2

**19**							**K**	***147.1128***	74.0600	130.0863	65.5468			**1**

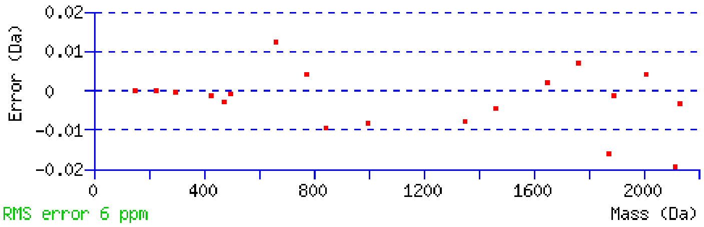

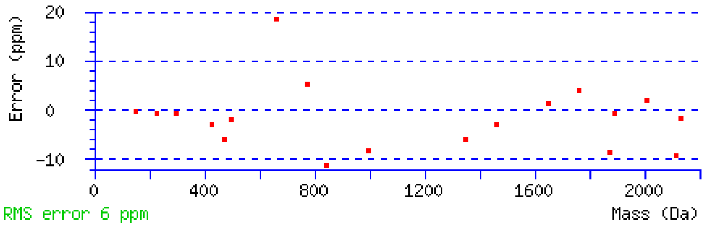

NCBI **BLAST** search of VELEDWNGRTSTADYAMFK
(Parameters: blastp, nr protein database, expect=20000, no filter, PAM30)
Other BLAST web gateways

All matches to this query

Score	Mr(calc)	Delta	Sequence
121.7	2232.9997	-0.0009	VELEDWNGRTSTADYAMFK
0.7	2232.9957	0.0031	VEDENALSIFETNCHSGSPK

**Mascot:** http://www.matrixscience.com/

Mascot Search Results

Peptide View

MS/MS Fragmentation of **VELEDWNGRTSTADYAMFK**
Found in **P02679** in **uni_human_nr**, FIBG_HUMAN Fibrinogen gamma chain OS=Homo sapiens GN=FGG PE=1 SV=3

Match to Query 8781: 2233.004008 from(1117.509280,2+) intensity(304648.5938) rtinseconds(2638) scans(15170) index(11537)
Title: 130806_HPL_Human_Plaque_BR1_TR2_12_Spectrum032656_scans__15170_RTINSECONDS=2638
Data file E:\\RAW\\HumanPlaques\\mgf\\T\\1D\\T130806_HPL_Human_Plaque_BR1_TR2_12.mgf

Click mouse within plot area to zoom in by factor of two about that point
Or, [Plot from] 0 to 1700 Da [Full range]
Label all possible matches ○ Label matches used for scoring ⊙
Show Y-axis ☐

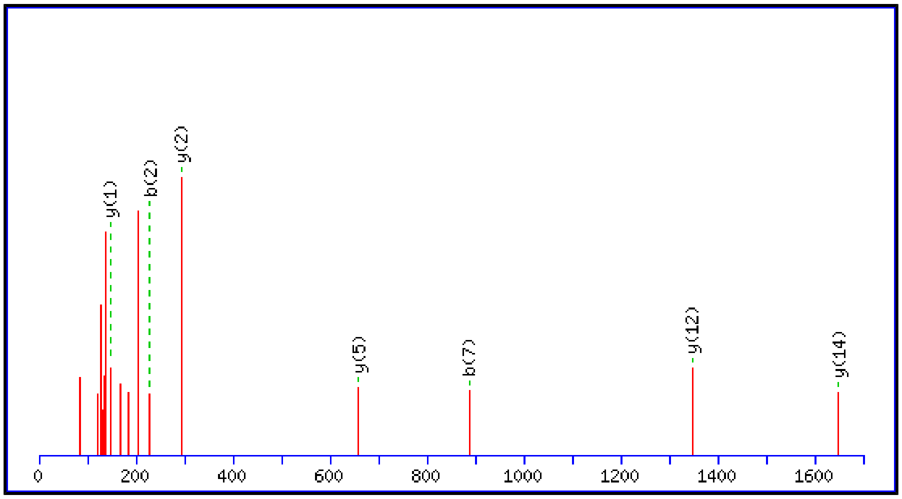

Monoisotopic mass of neutral peptide Mr(calc): 2232.9997
Fixed modifications: Carbamidomethyl (C) (apply to specified residues or termini only)
Variable modifications:
N7 : Deamidated (NQ)
Ions Score: 28 **Expect:** 0.083
Matches : 7/192 fragment ions using 11 most intense peaks (help)

#	b	b^{++}	b*	b^{*++}	b^{0}	b^{0++}	Seq.	y	y^{++}	y*	y^{*++}	y^{0}	y^{0++}	#
1	100.0757	50.5415					V							19
2	**229.1183**	115.0628			211.1077	106.0575	E	2134.9386	1067.9729	2117.9121	1059.4597	2116.9280	1058.9677	18
3	342.2023	171.6048			324.1918	162.5995	L	2005.8960	1003.4516	1988.8695	994.9384	1987.8855	994.4464	17
4	471.2449	236.1261			453.2344	227.1208	E	1892.8120	946.9096	1875.7854	938.3963	1874.8014	937.9043	16
5	586.2719	293.6396			568.2613	284.6343	D	1763.7694	882.3883	1746.7428	873.8750	1745.7588	873.3830	15
6	772.3512	386.6792			754.3406	377.6740	W	***1648.7424***	824.8748	1631.7159	816.3616	1630.7319	815.8696	14
7	**887.3781**	444.1927	870.3516	435.6794	869.3676	435.1874	N	1462.6631	731.8352	1445.6366	723.3219	1444.6525	722.8299	13
8	944.3996	472.7034	927.3731	464.1902	926.3890	463.6982	G	***1347.6362***	674.3217	1330.6096	665.8084	1329.6256	665.3164	12
9	1100.5007	550.7540	1083.4742	542.2407	1082.4901	541.7487	R	1290.6147	645.8110	1273.5882	637.2977	1272.6041	636.8057	11
10	1201.5484	601.2778	1184.5218	592.7646	1183.5378	592.2726	T	1134.5136	567.7604	1117.4870	559.2472	1116.5030	558.7552	10
11	1288.5804	644.7938	1271.5539	636.2806	1270.5699	635.7886	S	1033.4659	517.2366	1016.4394	508.7233	1015.4553	508.2313	9
12	1389.6281	695.3177	1372.6016	686.8044	1371.6175	686.3124	T	946.4339	473.7206	929.4073	465.2073	928.4233	464.7153	8
13	1460.6652	730.8362	1443.6387	722.3230	1442.6546	721.8310	A	845.3862	423.1967	828.3597	414.6835	827.3756	414.1915	7
14	1575.6922	788.3497	1558.6656	779.8364	1557.6816	779.3444	D	774.3491	387.6782	757.3225	379.1649	756.3385	378.6729	6
15	1738.7555	869.8814	1721.7289	861.3681	1720.7449	860.8761	Y	***659.3221***	330.1647	642.2956	321.6514			5
16	1809.7926	905.3999	1792.7661	896.8867	1791.7820	896.3947	A	496.2588	248.6330	479.2323	240.1198			4
17	1940.8331	970.9202	1923.8065	962.4069	1922.8225	961.9149	M	425.2217	213.1145	408.1952	204.6012			3
18	2087.9015	1044.4544	2070.8749	1035.9411	2069.8909	1035.4491	F	***294.1812***	147.5942	277.1547	139.0810			2
19							K	147.1128	74.0600	130.0863	65.5468			1

**19**							**K**	***147.1128***	74.0600	130.0863	65.5468			**1**

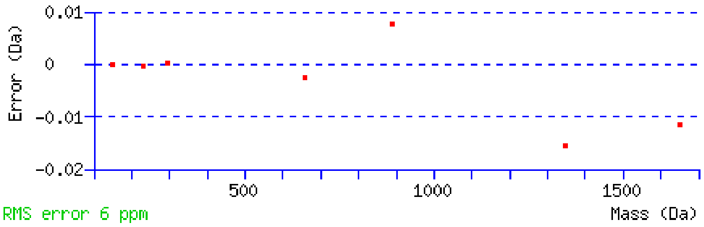

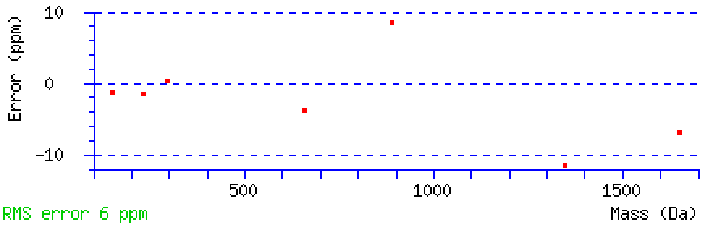

NCBI **BLAST** search of VELEDWNGRTSTADYAMFK
(Parameters: blastp, nr protein database, expect=20000, no filter, PAM30)
Other BLAST web gateways

All matches to this query

Score	Mr(calc)	Delta	Sequence
27.5	2232.9997	0.0043	VELEDWNGRTSTADYAMFK
1.6	2233.0051	-0.0011	HRRIHTGEKPHMCDDCGK

Mascot: http://www.matrixscience.com/

Mascot Search Results

Peptide View

MS/MS Fragmentation of **VELEDWNGRTSTADYAMFK**
Found in **P02679** in **uni_human_nr**, FIBG_HUMAN Fibrinogen gamma chain OS=Homo sapiens GN=FGG PE=1 SV=3

Match to Query 10578: 2232.015702 from(745.012510,3+) intensity(3327254.5000) rtinseconds(2450) scans(14803) index(11624)
Title: 130809_HPL_Human_Plaque_BR1_TR3_05_Spectrum034189_scans__14803_RTINSECONDS=2450
Data file E:\\RAW\\HumanPlaques\\mgf\\T\\1D\\T130809_HPL_Human_Plaque_BR1_TR3_05.mgf

Click mouse within plot area to zoom in by factor of two about that point
Or, Plot from 0 to 2200 Da Full range
Label all possible matches ○ Label matches used for scoring ⦿
Show Y-axis ☐

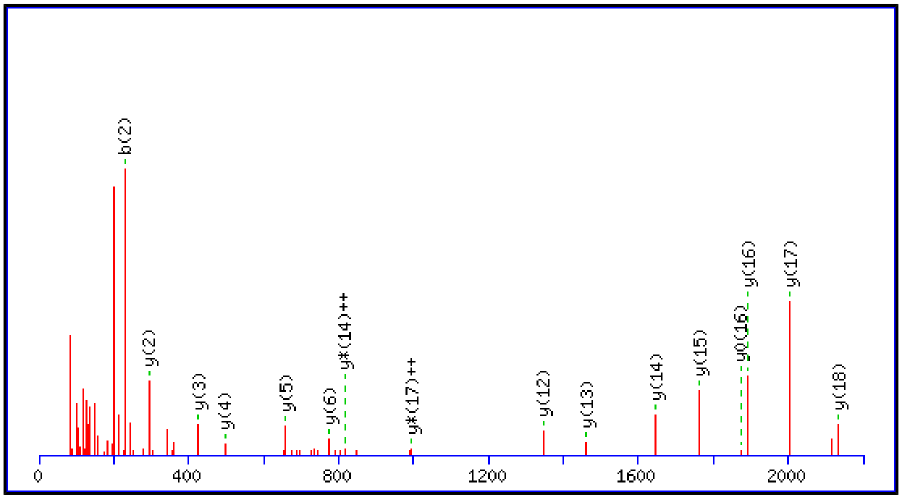

Monoisotopic mass of neutral peptide Mr(calc): 2232.0157
Fixed modifications: Carbamidomethyl (C) (apply to specified residues or termini only)
Ions Score: 120 **Expect:** 6.4e-011
Matches : 16/192 fragment ions using 17 most intense peaks (help)

#	b	b^{++}	b*	b*$^{++}$	b^{0}	b^{0++}	Seq.	y	y^{++}	y*	y*$^{++}$	y^{0}	y^{0++}	#
1	100.0757	50.5415					V							19
2	**229.1183**	115.0628			211.1077	106.0575	E	***2133.9546***	1067.4809	2116.9280	1058.9677	2115.9440	1058.4757	18
3	342.2023	171.6048			324.1918	162.5995	L	***2004.9120***	1002.9596	1987.8855	994.4464	1986.9014	993.9544	17
4	471.2449	236.1261			453.2344	227.1208	E	***1891.8279***	946.4176	1874.8014	937.9043	1873.8174	937.4123	16
5	586.2719	293.6396			568.2613	284.6343	D	***1762.7853***	881.8963	1745.7588	873.3830	1744.7748	872.8910	15
6	772.3512	386.6792			754.3406	377.6740	W	***1647.7584***	824.3828	1630.7319	815.8696	1629.7478	815.3776	14
7	886.3941	443.7007	869.3676	435.1874	868.3836	434.6954	N	***1461.6791***	731.3432	1444.6525	722.8299	1443.6685	722.3379	13
8	943.4156	472.2114	926.3890	463.6982	925.4050	463.2061	G	***1347.6362***	674.3217	1330.6096	665.8084	1329.6256	665.3164	12
9	1099.5167	550.2620	1082.4901	541.7487	1081.5061	541.2567	R	1290.6147	645.8110	1273.5882	637.2977	1272.6041	636.8057	11
10	1200.5644	600.7858	1183.5378	592.2726	1182.5538	591.7805	T	1134.5136	567.7604	1117.4870	559.2472	1116.5030	558.7552	10
11	1287.5964	644.3018	1270.5699	635.7886	1269.5858	635.2966	S	1033.4659	517.2366	1016.4394	508.7233	1015.4553	508.2313	9
12	1388.6441	694.8257	1371.6175	686.3124	1370.6335	685.8204	T	946.4339	473.7206	929.4073	465.2073	928.4233	464.7153	8
13	1459.6812	730.3442	1442.6546	721.8310	1441.6706	721.3390	A	845.3862	423.1967	828.3597	414.6835	827.3756	414.1915	7
14	1574.7081	787.8577	1557.6816	779.3444	1556.6976	778.8524	D	***774.3491***	387.6782	757.3225	379.1649	756.3385	378.6729	6
15	1737.7715	869.3894	1720.7449	860.8761	1719.7609	860.3841	Y	***659.3221***	330.1647	642.2956	321.6514			5
16	1808.8086	904.9079	1791.7820	896.3947	1790.7980	895.9026	A	***496.2588***	248.6330	479.2323	240.1198			4
17	1939.8491	970.4282	1922.8225	961.9149	1921.8385	961.4229	M	***425.2217***	213.1145	408.1952	204.6012			3
18	2086.9175	1043.9624	2069.8909	1035.4491	2068.9069	1034.9571	F	***294.1812***	147.5942	277.1547	139.0810			2
19							K	147.1128	74.0600	130.0863	65.5468			1

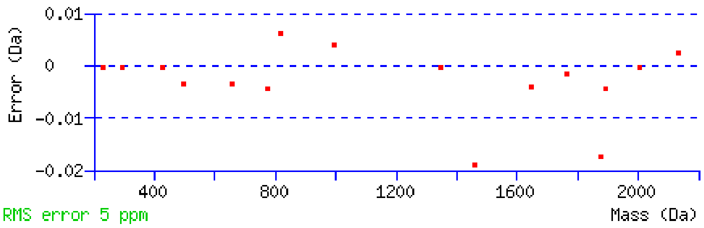

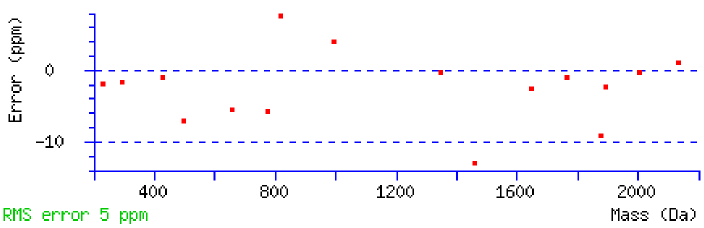

NCBI **BLAST** search of VELEDWNGRTSTADYAMFK
(Parameters: blastp, nr protein database, expect=20000, no filter, PAM30)
Other BLAST web gateways

All matches to this query

Score	Mr(calc)	Delta	Sequence
119.5	2232.0157	-0.0000	VELEDWNGRTSTADYAMFK

Mascot: http://www.matrixscience.com/

Mascot Search Results

Peptide View

MS/MS Fragmentation of **VELEDWNGRTSTADYAMFK**
Found in **P02679** in **uni_human_nr**, FIBG_HUMAN Fibrinogen gamma chain OS=Homo sapiens GN=FGG PE=1 SV=3

Match to Query 10924: 2232.015468 from(1117.015010,2+) intensity(658890.8125) rtinseconds(2437) scans(14905) index(11856)
Title: 130809_HPL_Human_Plaque_BR2_TR3_05_Spectrum034512_scans__14905_RTINSECONDS=2437
Data file E:\\RAW\\HumanPlaques\\mgf\\T\\1D\\T130809_HPL_Human_Plaque_BR2_TR3_05.mgf

Click mouse within plot area to zoom in by factor of two about that point
Or, [Plot from] [0] to [1200] Da [Full range]
Label all possible matches ○ Label matches used for scoring ⊙
Show Y-axis ☐

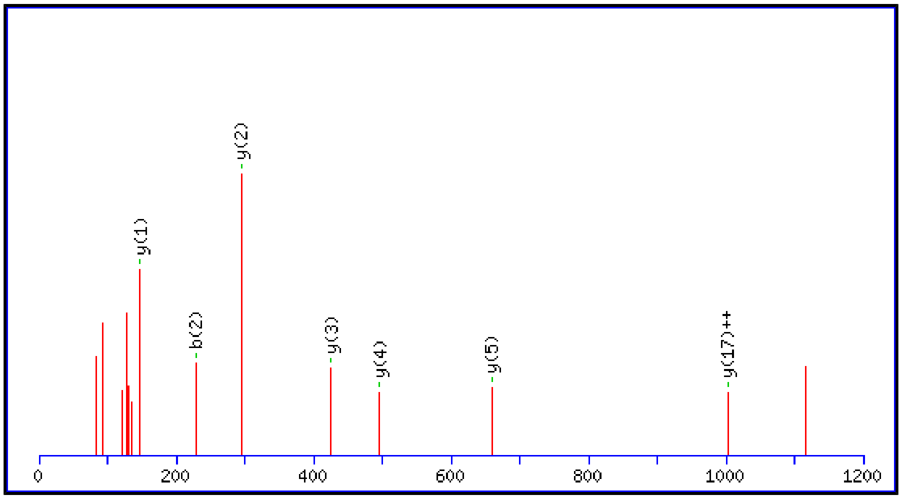

Monoisotopic mass of neutral peptide Mr(calc): 2232.0157
Fixed modifications: Carbamidomethyl (C) (apply to specified residues or termini only)
Ions Score: 38 **Expect:** 0.0091
Matches : 7/192 fragment ions using 8 most intense peaks (help)

#	b	b^{++}	b*	b*$^{++}$	b^{0}	b^{0++}	Seq.	y	y^{++}	y*	y*$^{++}$	y^{0}	y^{0++}	#
1	100.0757	50.5415					V							19
2	**229.1183**	115.0628			211.1077	106.0575	E	2133.9546	1067.4809	2116.9280	1058.9677	2115.9440	1058.4757	18
3	342.2023	171.6048			324.1918	162.5995	L	2004.9120	1002.9596	1987.8855	994.4464	1986.9014	993.9544	17
4	471.2449	236.1261			453.2344	227.1208	E	1891.8279	946.4176	1874.8014	937.9043	1873.8174	937.4123	16
5	586.2719	293.6396			568.2613	284.6343	D	1762.7853	881.8963	1745.7588	873.3830	1744.7748	872.8910	15
6	772.3512	386.6792			754.3406	377.6740	W	1647.7584	824.3828	1630.7319	815.8696	1629.7478	815.3776	14
7	886.3941	443.7007	869.3676	435.1874	868.3836	434.6954	N	1461.6791	731.3432	1444.6525	722.8299	1443.6685	722.3379	13
8	943.4156	472.2114	926.3890	463.6982	925.4050	463.2061	G	1347.6362	674.3217	1330.6096	665.8084	1329.6256	665.3164	12
9	1099.5167	550.2620	1082.4901	541.7487	1081.5061	541.2567	R	1290.6147	645.8110	1273.5882	637.2977	1272.6041	636.8057	11
10	1200.5644	600.7858	1183.5378	592.2726	1182.5538	591.7805	T	1134.5136	567.7604	1117.4870	559.2472	1116.5030	558.7552	10
11	1287.5964	644.3018	1270.5699	635.7886	1269.5858	635.2966	S	1033.4659	517.2366	1016.4394	508.7233	1015.4553	508.2313	9
12	1388.6441	694.8257	1371.6175	686.3124	1370.6335	685.8204	T	946.4339	473.7206	929.4073	465.2073	928.4233	464.7153	8
13	1459.6812	730.3442	1442.6546	721.8310	1441.6706	721.3390	A	845.3862	423.1967	828.3597	414.6835	827.3756	414.1915	7
14	1574.7081	787.8577	1557.6816	779.3444	1556.6976	778.8524	D	774.3491	387.6782	757.3225	379.1649	756.3385	378.6729	6
15	1737.7715	869.3894	1720.7449	860.8761	1719.7609	860.3841	Y	***659.3221***	330.1647	642.2956	321.6514			5
16	1808.8086	904.9079	1791.7820	896.3947	1790.7980	895.9026	A	***496.2588***	248.6330	479.2323	240.1198			4
17	1939.8491	970.4282	1922.8225	961.9149	1921.8385	961.4229	M	***425.2217***	213.1145	408.1952	204.6012			3
18	2086.9175	1043.9624	2069.8909	1035.4491	2068.9069	1034.9571	F	***294.1812***	147.5942	277.1547	139.0810			2
19							K	***147.1128***	74.0600	130.0863	65.5468			1

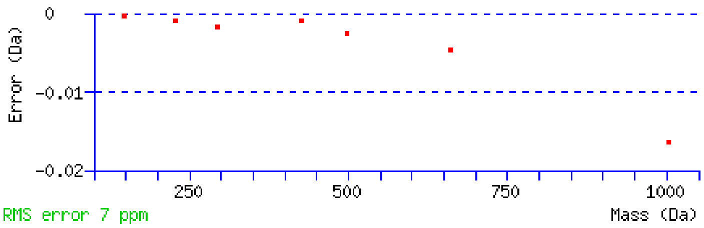

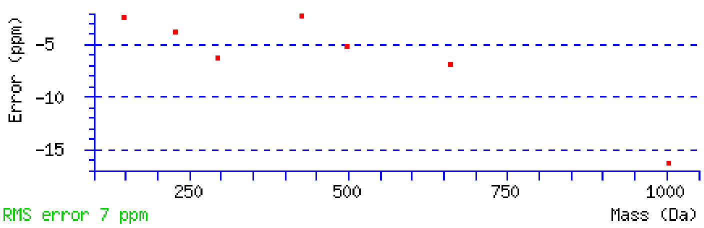

NCBI **BLAST** search of VELEDWNGRTSTADYAMFK
(Parameters: blastp, nr protein database, expect=20000, no filter, PAM30)
Other BLAST web gateways

All matches to this query

Score	Mr(calc)	Delta	Sequence
37.8	2232.0157	-0.0002	VELEDWNGRTSTADYAMFK
6.8	2231.0085	1.0069	ENLKGEGMSQAATICKSNFK
6.8	2232.0224	-0.0070	MNLKGEGMSQAATICKSNFK
6.8	2232.0224	-0.0070	MNLKGEGMSQAATICKSNFK
6.8	2232.0224	-0.0070	MNLKGEGMSQAATICKSNFK
6.8	2232.0224	-0.0070	MNLKGEGMSQAATICKSNFK
6.8	2231.0085	1.0069	QNLKGEGMSQAATICKSNFK
1.4	2229.9987	2.0168	ANEMNISPASLASLYEEDFK
1.4	2231.0144	1.0011	FHWGRENQRGSEHTVNFK
1.4	2232.0222	-0.0068	GYPTYTNVEVVNDGAESAAFK

Mascot: http://www.matrixscience.com/

Mascot Search Results

Peptide View

MS/MS Fragmentation of **CTCVGNGRGEWTCIAYSQLR**
Found in **P02751** in **uni_human_nr**, FINC_HUMAN Fibronectin OS=Homo sapiens GN=FN1 PE=1 SV=4

Match to Query 13495: 2388.035472 from(797.019100,3+) intensity(2500728.5000) rtinseconds(2295) scans(14567) index(11697)
Title: 130806_HPL_Human_Plaque_BR1_TR2_03_Spectrum035636_scans__14567_RTINSECONDS=2295
Data file E:\\RAW\\HumanPlaques\\mgf\\T\\1D\\T130806_HPL_Human_Plaque_BR1_TR2_03.mgf

Click mouse within plot area to zoom in by factor of two about that point
Or, Plot from 0 to 2400 Da Full range
Label all possible matches ○ Label matches used for scoring ⊙
Show Y-axis ☐

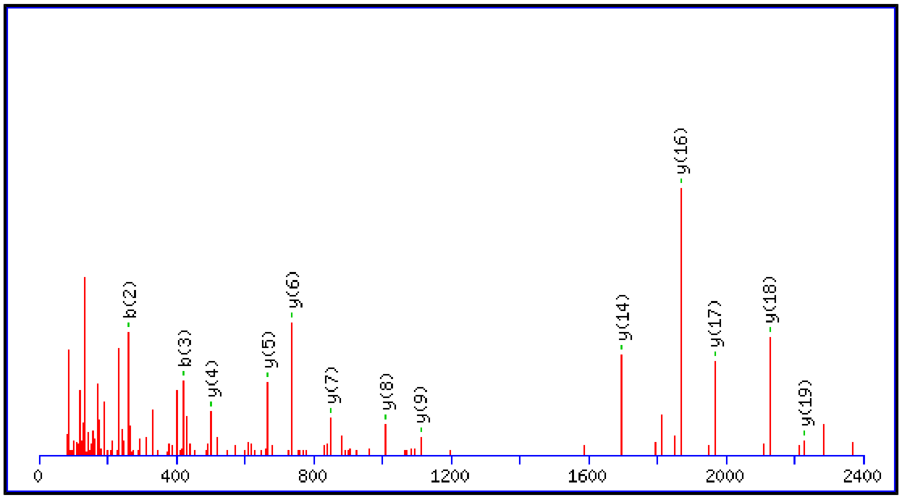

Monoisotopic mass of neutral peptide Mr(calc): 2388.0409
Fixed modifications: Carbamidomethyl (C) (apply to specified residues or termini only)
Variable modifications:
N6 : Deamidated (NQ)
Ions Score: 83 **Expect:** 1.5e-007
Matches : 13/210 fragment ions using 19 most intense peaks (help)

#	b	b^{++}	b^{*}	b^{*++}	b^{0}	b^{0++}	Seq.	y	y^{++}	y^{*}	y^{*++}	y^{0}	y^{0++}	#
1	161.0379	81.0226					C							20
2	**262.0856**	131.5464			244.0750	122.5412	T	***2229.0176***	1115.0124	2211.9910	1106.4991	2211.0070	1106.0071	19
3	**422.1163**	211.5618			404.1057	202.5565	C	***2127.9699***	1064.4886	2110.9433	1055.9753	2109.9593	1055.4833	18
4	521.1847	261.0960			503.1741	252.0907	V	***1967.9392***	984.4732	1950.9127	975.9600	1949.9287	975.4680	17
5	578.2061	289.6067			560.1956	280.6014	G	***1868.8708***	934.9390	1851.8443	926.4258	1850.8602	925.9338	16
6	693.2331	347.1202	676.2065	338.6069	675.2225	338.1149	N	1811.8493	906.4283	1794.8228	897.9150	1793.8388	897.4230	15
7	750.2545	375.6309	733.2280	367.1176	732.2440	366.6256	G	***1696.8224***	848.9148	1679.7959	840.4016	1678.8118	839.9096	14
8	906.3556	453.6815	889.3291	445.1682	888.3451	444.6762	R	1639.8009	820.4041	1622.7744	811.8908	1621.7904	811.3988	13
9	963.3771	482.1922	946.3506	473.6789	945.3665	473.1869	G	1483.6998	742.3536	1466.6733	733.8403	1465.6893	733.3483	12
10	1092.4197	546.7135	1075.3932	538.2002	1074.4091	537.7082	E	1426.6784	713.8428	1409.6518	705.3295	1408.6678	704.8375	11
11	1278.4990	639.7531	1261.4725	631.2399	1260.4885	630.7479	W	1297.6358	649.3215	1280.6092	640.8082	1279.6252	640.3162	10
12	1379.5467	690.2770	1362.5201	681.7637	1361.5361	681.2717	T	***1111.5565***	556.2819	1094.5299	547.7686	1093.5459	547.2766	9
13	1539.5773	770.2923	1522.5508	761.7790	1521.5668	761.2870	C	***1010.5088***	505.7580	993.4822	497.2448	992.4982	496.7527	8
14	1652.6614	826.8343	1635.6349	818.3211	1634.6508	817.8291	I	***850.4781***	425.7427	833.4516	417.2294	832.4676	416.7374	7
15	1723.6985	862.3529	1706.6720	853.8396	1705.6880	853.3476	A	***737.3941***	369.2007	720.3675	360.6874	719.3835	360.1954	6
16	1886.7619	943.8846	1869.7353	935.3713	1868.7513	934.8793	Y	***666.3570***	333.6821	649.3304	325.1688	648.3464	324.6768	5
17	1973.7939	987.4006	1956.7673	978.8873	1955.7833	978.3953	S	***503.2936***	252.1504	486.2671	243.6372	485.2831	243.1452	4
18	2101.8525	1051.4299	2084.8259	1042.9166	2083.8419	1042.4246	Q	416.2616	208.6344	399.2350	200.1212			3

**19**	2214.9365	1107.9719	2197.9100	1099.4586	2196.9260	1098.9666	**L**	288.2030	144.6051	271.1765	136.0919			**2**
**20**							**R**	175.1190	88.0631	158.0924	79.5498			**1**

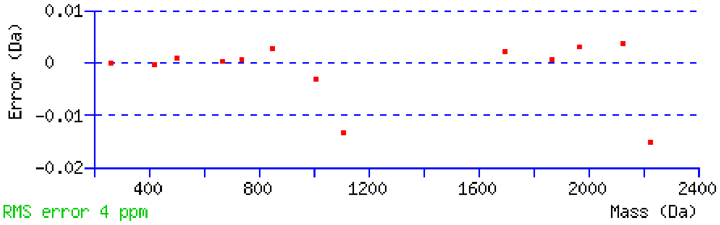

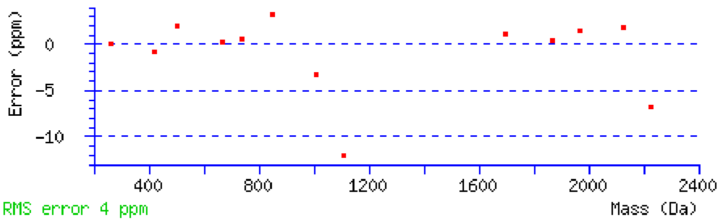

NCBI **BLAST** search of CTCVGNGRGEWTCIAYSQLR
(Parameters: blastp, nr protein database, expect=20000, no filter, PAM30)
Other BLAST web gateways

All matches to this query

Score	Mr(calc)	Delta	Sequence	Site Analysis
83.1	2388.0409	-0.0055	CTCVGNGRGEWTCIAYSQLR	Deamidated N6 100.00%
10.1	2388.0409	-0.0055	CTCVGNGRGEWTCIAYSQLR	Deamidated Q18 0.00%

Mascot: http://www.matrixscience.com/

Mascot Search Results

Peptide View

MS/MS Fragmentation of **CTCVGNGRGEWTCIAYSQLR**
Found in **P02751** in **uni_human_nr**, FINC_HUMAN Fibronectin OS=Homo sapiens GN=FN1 PE=1 SV=4

Match to Query 13958: 2387.054052 from(796.691960,3+) intensity(78002072.0000) rtinseconds(1933) scans(12127) index(9378)
Title: 130809_HPL_Human_Plaque_BR2_TR3_02_Spectrum034231_scans__12127_RTINSECONDS=1933
Data file E:\\RAW\\HumanPlaques\\mgf\\T\\1D\\T130809_HPL_Human_Plaque_BR2_TR3_02.mgf

Click mouse within plot area to zoom in by factor of two about that point
Or, [Plot from] [0] to [2400] Da [Full range]
Label all possible matches ○ Label matches used for scoring ⊙
Show Y-axis ☐

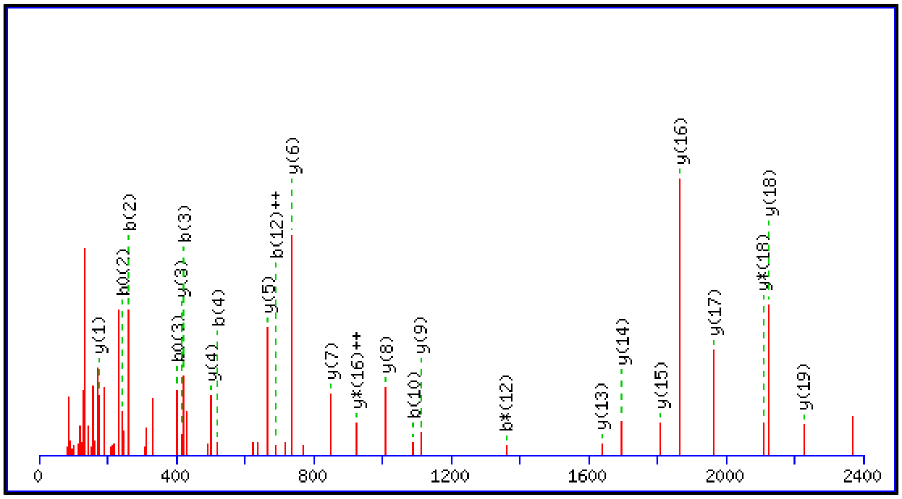

Monoisotopic mass of neutral peptide Mr(calc): 2387.0569
Fixed modifications: Carbamidomethyl (C) (apply to specified residues or termini only)
Ions Score: 112 **Expect:** 2.7e-010
Matches : 25/210 fragment ions using 42 most intense peaks (help)

#	b	b++	b*	b*++	b0	b0++	Seq.	y	y++	y*	y*++	y0	y0++	#
1	161.0379	81.0226					C							20
2	**262.0856**	131.5464			244.0750	122.5412	T	***2228.0335***	1114.5204	2211.0070	1106.0071	2210.0230	1105.5151	19
3	**422.1163**	211.5618			404.1057	202.5565	C	***2126.9859***	1063.9966	2109.9593	1055.4833	2108.9753	1054.9913	18
4	**521.1847**	261.0960			503.1741	252.0907	V	***1966.9552***	983.9812	1949.9287	975.4680	1948.9446	974.9760	17
5	578.2061	289.6067			560.1956	280.6014	G	***1867.8868***	934.4470	1850.8602	925.9338	1849.8762	925.4418	16
6	692.2491	346.6282	675.2225	338.1149	674.2385	337.6229	N	***1810.8653***	905.9363	1793.8388	897.4230	1792.8548	896.9310	15
7	749.2705	375.1389	732.2440	366.6256	731.2600	366.1336	G	***1696.8224***	848.9148	1679.7959	840.4016	1678.8118	839.9096	14
8	905.3716	453.1895	888.3451	444.6762	887.3611	444.1842	R	***1639.8009***	820.4041	1622.7744	811.8908	1621.7904	811.3988	13
9	962.3931	481.7002	945.3665	473.1869	944.3825	472.6949	G	1483.6998	742.3536	1466.6733	733.8403	1465.6893	733.3483	12
10	**1091.4357**	546.2215	1074.4091	537.7082	1073.4251	537.2162	E	1426.6784	713.8428	1409.6518	705.3295	1408.6678	704.8375	11
11	1277.5150	639.2611	1260.4885	630.7479	1259.5044	630.2559	W	1297.6358	649.3215	1280.6092	640.8082	1279.6252	640.3162	10
12	1378.5627	**689.7850**	1361.5361	681.2717	1360.5521	680.7797	T	***1111.5565***	556.2819	1094.5299	547.7686	1093.5459	547.2766	9
13	1538.5933	769.8003	1521.5668	761.2870	1520.5828	760.7950	C	***1010.5088***	505.7580	993.4822	497.2448	992.4982	496.7527	8
14	1651.6774	826.3423	1634.6508	817.8291	1633.6668	817.3371	I	***850.4781***	425.7427	833.4516	417.2294	832.4676	416.7374	7
15	1722.7145	861.8609	1705.6880	853.3476	1704.7039	852.8556	A	***737.3941***	369.2007	720.3675	360.6874	719.3835	360.1954	6
16	1885.7778	943.3926	1868.7513	934.8793	1867.7673	934.3873	Y	***666.3570***	333.6821	649.3304	325.1688	648.3464	324.6768	5
17	1972.8099	986.9086	1955.7833	978.3953	1954.7993	977.9033	S	***503.2936***	252.1504	486.2671	243.6372	485.2831	243.1452	4
18	2100.8684	1050.9379	2083.8419	1042.4246	2082.8579	1041.9326	Q	***416.2616***	208.6344	399.2350	200.1212			3
19	2213.9525	1107.4799	2196.9260	1098.9666	2195.9419	1098.4746	L	288.2030	144.6051	271.1765	136.0919			2

**20**							**R**	***175.1190***	88.0631	158.0924	79.5498				**1**

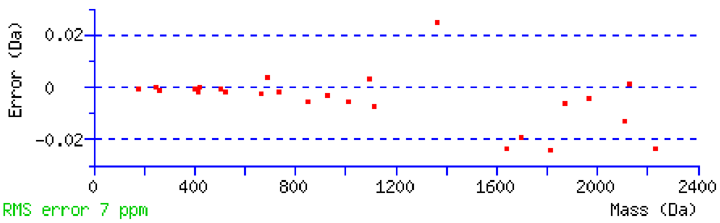

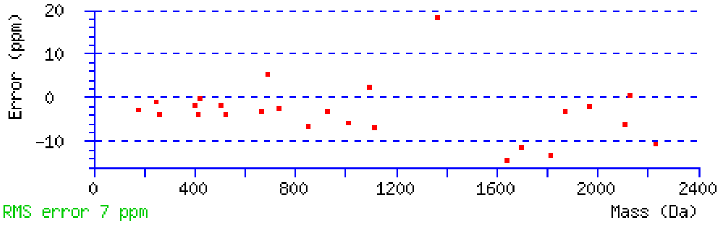

NCBI **BLAST** search of CTCVGNGRGEWTCIAYSQLR
(Parameters: blastp, nr protein database, expect=20000, no filter, PAM30)
Other BLAST web gateways

All matches to this query

Score	Mr(calc)	Delta	Sequence
112.0	2387.0569	-0.0029	CTCVGNGRGEWTCIAYSQLR

**Mascot:** http://www.matrixscience.com/

Mascot Search Results

Peptide View

MS/MS Fragmentation of **CTCVGNGRGEWTCIAYSQLR**
Found in **P02751** in **uni_human_nr**, FINC_HUMAN Fibronectin OS=Homo sapiens GN=FN1 PE=1 SV=4

Match to Query 13415: 2387.061868 from(1194.538210,2+) intensity(4408756.0000) rtinseconds(1950) scans(11628) index(8779)
Title: 130801_HPL_Human_Plaque_BR2_TR1_02_Spectrum032934_scans__11628_RTINSECONDS=1950
Data file E:\\RAW\\HumanPlaques\\mgf\\T\\1D\\T130801_HPL_Human_Plaque_BR2_TR1_02.mgf

Click mouse within plot area to zoom in by factor of two about that point
Or, Plot from 0 to 2400 Da Full range
Label all possible matches ○ Label matches used for scoring ⊙
Show Y-axis ☐

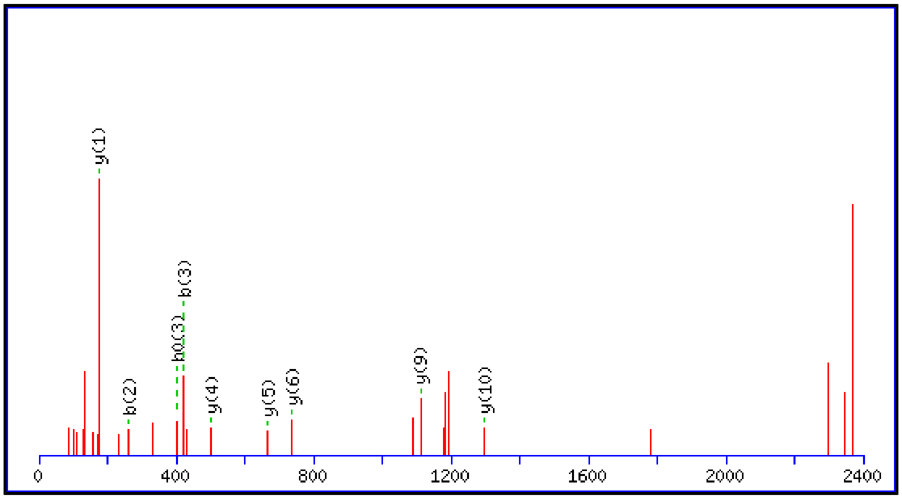

Monoisotopic mass of neutral peptide Mr(calc): 2387.0569
Fixed modifications: Carbamidomethyl (C) (apply to specified residues or termini only)
Ions Score: 34 **Expect:** 0.017
Matches : 9/210 fragment ions using 17 most intense peaks (help)

#	b	b++	b*	b*++	b0	b0++	Seq.	y	y++	y*	y*++	y0	y0++	#
1	161.0379	81.0226					C							20
2	**262.0856**	131.5464			244.0750	122.5412	T	2228.0335	1114.5204	2211.0070	1106.0071	2210.0230	1105.5151	19
3	**422.1163**	211.5618			404.1057	202.5565	C	2126.9859	1063.9966	2109.9593	1055.4833	2108.9753	1054.9913	18
4	521.1847	261.0960			503.1741	252.0907	V	1966.9552	983.9812	1949.9287	975.4680	1948.9446	974.9760	17
5	578.2061	289.6067			560.1956	280.6014	G	1867.8868	934.4470	1850.8602	925.9338	1849.8762	925.4418	16
6	692.2491	346.6282	675.2225	338.1149	674.2385	337.6229	N	1810.8653	905.9363	1793.8388	897.4230	1792.8548	896.9310	15
7	749.2705	375.1389	732.2440	366.6256	731.2600	366.1336	G	1696.8224	848.9148	1679.7959	840.4016	1678.8118	839.9096	14
8	905.3716	453.1895	888.3451	444.6762	887.3611	444.1842	R	1639.8009	820.4041	1622.7744	811.8908	1621.7904	811.3988	13
9	962.3931	481.7002	945.3665	473.1869	944.3825	472.6949	G	1483.6998	742.3536	1466.6733	733.8403	1465.6893	733.3483	12
10	1091.4357	546.2215	1074.4091	537.7082	1073.4251	537.2162	E	1426.6784	713.8428	1409.6518	705.3295	1408.6678	704.8375	11
11	1277.5150	639.2611	1260.4885	630.7479	1259.5044	630.2559	W	***1297.6358***	649.3215	1280.6092	640.8082	1279.6252	640.3162	10
12	1378.5627	689.7850	1361.5361	681.2717	1360.5521	680.7797	T	***1111.5565***	556.2819	1094.5299	547.7686	1093.5459	547.2766	9
13	1538.5933	769.8003	1521.5668	761.2870	1520.5828	760.7950	C	1010.5088	505.7580	993.4822	497.2448	992.4982	496.7527	8
14	1651.6774	826.3423	1634.6508	817.8291	1633.6668	817.3371	I	850.4781	425.7427	833.4516	417.2294	832.4676	416.7374	7
15	1722.7145	861.8609	1705.6880	853.3476	1704.7039	852.8556	A	***737.3941***	369.2007	720.3675	360.6874	719.3835	360.1954	6
16	1885.7778	943.3926	1868.7513	934.8793	1867.7673	934.3873	Y	***666.3570***	333.6821	649.3304	325.1688	648.3464	324.6768	5
17	1972.8099	986.9086	1955.7833	978.3953	1954.7993	977.9033	S	***503.2936***	252.1504	486.2671	243.6372	485.2831	243.1452	4
18	2100.8684	1050.9379	2083.8419	1042.4246	2082.8579	1041.9326	Q	416.2616	208.6344	399.2350	200.1212			3
19	2213.9525	1107.4799	2196.9260	1098.9666	2195.9419	1098.4746	L	288.2030	144.6051	271.1765	136.0919			2

**20**							**R**	***175.1190***	88.0631	158.0924	79.5498			**1**

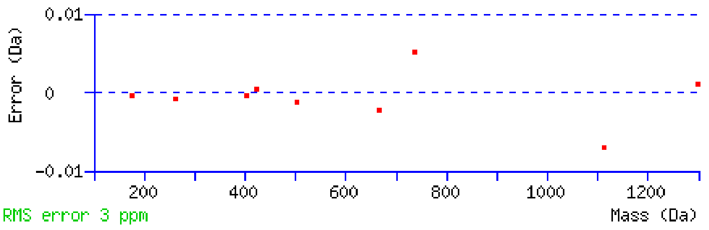

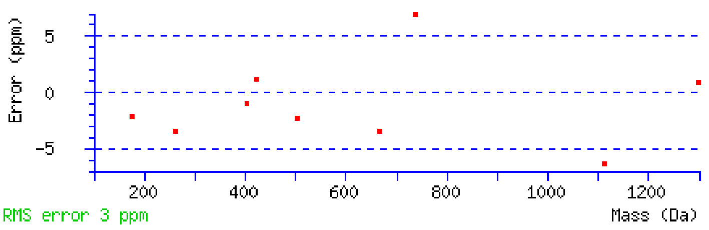

NCBI **BLAST** search of CTCVGNGRGEWTCIAYSQLR
(Parameters: blastp, nr protein database, expect=20000, no filter, PAM30)
Other BLAST web gateways

All matches to this query

Score	Mr(calc)	Delta	Sequence
34.3	2387.0569	0.0050	CTCVGNGRGEWTCIAYSQLR
2.2	2387.0674	-0.0056	MASSGMADSANHLPFFFGNITR
2.2	2387.0674	-0.0056	MASSGMADSANHLPFFFGNITR
1.7	2387.0634	-0.0016	VCGLCGNFDDIAVNDFATRSR
1.7	2387.0634	-0.0016	VCGLCGNFDDIAVNDFATRSR
1.7	2387.0699	-0.0080	EQQQLSDVCYRQASQLEFR
1.7	2387.0636	-0.0017	MQFMKYMVYPQTFALNADR
1.7	2387.0636	-0.0017	MQFMKYMVYPQTFALNADR
1.7	2387.0636	-0.0017	MQFMKYMVYPQTFALNADR
1.7	2387.0699	-0.0080	QQQQLSDVCYRQASQLEFR

Mascot: http://www.matrixscience.com/

Mascot Search Results

Peptide View

MS/MS Fragmentation of **DNRGNLLQCICTGNGR**
Found in **P02751** in **uni_human_nr**, FINC_HUMAN Fibronectin OS=Homo sapiens GN=FN1 PE=1 SV=4

Match to Query 5968: 1847.836992 from(616.952940,3+) intensity(291876.9063) rtinseconds(1956) scans(10328) index(7461)
Title: 130801_HPL_Human_Plaque_BR2_TR1_10_Spectrum028016_scans__10328_RTINSECONDS=1956
Data file E:\\RAW\\HumanPlaques\\mgf\\T\\1D\\T130801_HPL_Human_Plaque_BR2_TR1_10.mgf

Click mouse within plot area to zoom in by factor of two about that point
Or, Plot from 50 to 1100 Da Full range
Label all possible matches ○ Label matches used for scoring ◉
Show Y-axis ☐

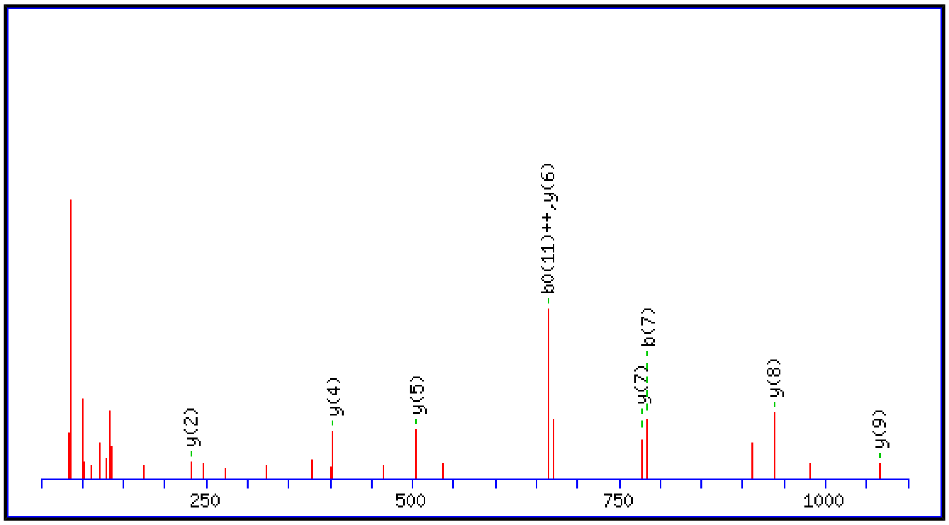


```
Monoisotopic mass of neutral peptide Mr(calc): 1847.8367
Fixed modifications: Carbamidomethyl (C) (apply to specified residues or termini only)
Variable modifications:
N5      : Deamidated (NQ)
Ions Score: 56  Expect: 0.00012
Matches : 9/170 fragment ions using 10 most intense peaks   (help)
```

#	b	b^{++}	b*	b^{*++}	b^0	b^{0++}	Seq.	y	y^{++}	y*	y^{*++}	y^0	y^{0++}	#
1	116.0342	58.5207			98.0237	49.5155	D							16
2	230.0771	115.5422	213.0506	107.0289	212.0666	106.5369	N	1733.8170	867.4121	1716.7904	858.8989	1715.8064	858.4069	15
3	386.1783	193.5928	369.1517	185.0795	368.1677	184.5875	R	1619.7741	810.3907	1602.7475	801.8774	1601.7635	801.3854	14
4	443.1997	222.1035	426.1732	213.5902	425.1892	213.0982	G	1463.6730	732.3401	1446.6464	723.8268	1445.6624	723.3348	13
5	558.2267	279.6170	541.2001	271.1037	540.2161	270.6117	N	1406.6515	703.8294	1389.6249	695.3161	1388.6409	694.8241	12
6	671.3107	336.1590	654.2842	327.6457	653.3002	327.1537	L	1291.6246	646.3159	1274.5980	637.8026	1273.6140	637.3106	11
7	**784.3948**	392.7010	767.3682	384.1878	766.3842	383.6958	L	1178.5405	589.7739	1161.5139	581.2606	1160.5299	580.7686	10
8	912.4534	456.7303	895.4268	448.2170	894.4428	447.7250	Q	***1065.4564***	533.2319	1048.4299	524.7186	1047.4459	524.2266	9
9	1072.4840	536.7456	1055.4575	528.2324	1054.4735	527.7404	C	***937.3978***	469.2026	920.3713	460.6893	919.3873	460.1973	8
10	1185.5681	593.2877	1168.5415	584.7744	1167.5575	584.2824	I	***777.3672***	389.1872	760.3406	380.6740	759.3566	380.1820	7
11	1345.5987	673.3030	1328.5722	664.7897	1327.5882	664.2977	C	***664.2831***	332.6452	647.2566	324.1319	646.2726	323.6399	6
12	1446.6464	723.8268	1429.6199	715.3136	1428.6358	714.8216	T	***504.2525***	252.6299	487.2259	244.1166	486.2419	243.6246	5
13	1503.6679	752.3376	1486.6413	743.8243	1485.6573	743.3323	G	***403.2048***	202.1060	386.1783	193.5928			4
14	1617.7108	809.3590	1600.6843	800.8458	1599.7002	800.3538	N	346.1833	173.5953	329.1568	165.0820			3
15	1674.7323	837.8698	1657.7057	829.3565	1656.7217	828.8645	G	***232.1404***	116.5738	215.1139	108.0606			2

16							R	175.1190	88.0631	158.0924	79.5498		1

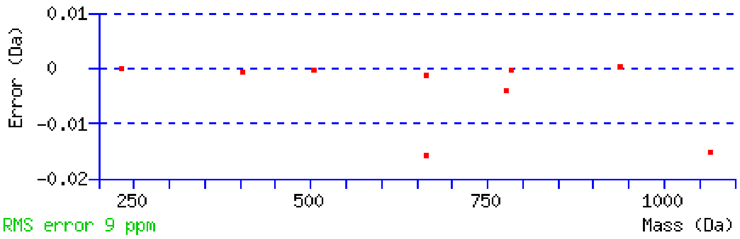

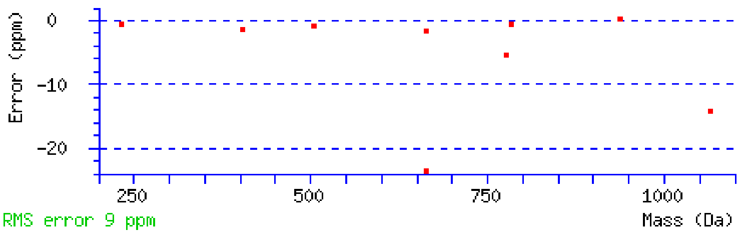

NCBI **BLAST** search of DNRGNLLQCICTGNGR
(Parameters: blastp, nr protein database, expect=20000, no filter, PAM30)
Other BLAST web gateways

All matches to this query

Score	Mr(calc)	Delta	Sequence	Site Analysis
55.7	1847.8367	0.0003	DNRGNLLQCICTGNGR	Deamidated N5 49.44%
55.7	1847.8367	0.0003	DNRGNLLQCICTGNGR	Deamidated N2 49.44%
36.7	1847.8367	0.0003	DNRGNLLQCICTGNGR	Deamidated Q8 0.63%

Mascot: http://www.matrixscience.com/

Mascot Search Results

Peptide View

MS/MS Fragmentation of **DNRGNLLQCICTGNGRGEWK**
Found in **P02751** in **uni_human_nr**, FINC_HUMAN Fibronectin OS=Homo sapiens GN=FN1 PE=1 SV=4

Match to Query 8801: 2349.059016 from(588.272030,4+) intensity(335275.2500) rtinseconds(2279) scans(12331) index(8907)
Title: 130801_HPL_Human_Plaque_BR1_TR1_13_Spectrum029377_scans__12331_RTINSECONDS=2279
Data file E:\\RAW\\HumanPlaques\\mgf\\T\\1D\\T130801_HPL_Human_Plaque_BR1_TR1_13.mgf

Click mouse within plot area to zoom in by factor of two about that point
Or, Plot from 0 to 1600 Da Full range
Label all possible matches ○ Label matches used for scoring ⊙
Show Y-axis ☐

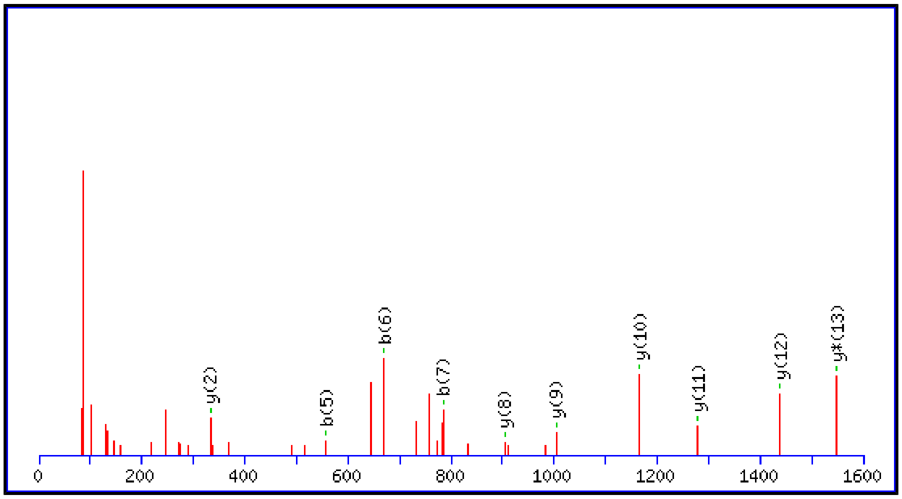

Monoisotopic mass of neutral peptide Mr(calc): 2349.0590
Fixed modifications: Carbamidomethyl (C) (apply to specified residues or termini only)
Variable modifications:
N5 : Deamidated (NQ)
N14 : Deamidated (NQ)
Ions Score: 42 **Expect:** 0.0034
Matches : 10/222 fragment ions using 13 most intense peaks (help)

#	b	b^{++}	b*	b^{*++}	b^0	b^{0++}	Seq.	y	y^{++}	y*	y^{*++}	y^0	y^{0++}	#
1	116.0342	58.5207			98.0237	49.5155	D							20
2	230.0771	115.5422	213.0506	107.0289	212.0666	106.5369	N	2235.0393	1118.0233	2218.0128	1109.5100	2217.0288	1109.0180	19
3	386.1783	193.5928	369.1517	185.0795	368.1677	184.5875	R	2120.9964	1061.0018	2103.9699	1052.4886	2102.9859	1051.9966	18
4	443.1997	222.1035	426.1732	213.5902	425.1892	213.0982	G	1964.8953	982.9513	1947.8688	974.4380	1946.8847	973.9460	17
5	***558.2267***	279.6170	541.2001	271.1037	540.2161	270.6117	N	1907.8738	954.4406	1890.8473	945.9273	1889.8633	945.4353	16
6	***671.3107***	336.1590	654.2842	327.6457	653.3002	327.1537	L	1792.8469	896.9271	1775.8204	888.4138	1774.8363	887.9218	15
7	***784.3948***	392.7010	767.3682	384.1878	766.3842	383.6958	L	1679.7628	840.3851	1662.7363	831.8718	1661.7523	831.3798	14
8	912.4534	456.7303	895.4268	448.2170	894.4428	447.7250	Q	1566.6788	783.8430	1549.6522	775.3298	1548.6682	774.8377	13
9	1072.4840	536.7456	1055.4575	528.2324	1054.4735	527.7404	C	***1438.6202***	719.8137	1421.5936	711.3005	1420.6096	710.8085	12
10	1185.5681	593.2877	1168.5415	584.7744	1167.5575	584.2824	I	***1278.5895***	639.7984	1261.5630	631.2851	1260.5790	630.7931	11
11	1345.5987	673.3030	1328.5722	664.7897	1327.5882	664.2977	C	***1165.5055***	583.2564	1148.4789	574.7431	1147.4949	574.2511	10
12	1446.6464	723.8268	1429.6199	715.3136	1428.6358	714.8216	T	***1005.4748***	503.2411	988.4483	494.7278	987.4643	494.2358	9
13	1503.6679	752.3376	1486.6413	743.8243	1485.6573	743.3323	G	***904.4272***	452.7172	887.4006	444.2039	886.4166	443.7119	8
14	1618.6948	809.8510	1601.6683	801.3378	1600.6843	800.8458	N	847.4057	424.2065	830.3791	415.6932	829.3951	415.2012	7
15	1675.7163	838.3618	1658.6897	829.8485	1657.7057	829.3565	G	732.3787	366.6930	715.3522	358.1797	714.3682	357.6877	6
16	1831.8174	916.4123	1814.7908	907.8991	1813.8068	907.4071	R	675.3573	338.1823	658.3307	329.6690	657.3467	329.1770	5
17	1888.8389	944.9231	1871.8123	936.4098	1870.8283	935.9178	G	519.2562	260.1317	502.2296	251.6185	501.2456	251.1264	4

**18**	2017.8814	1009.4444	2000.8549	1000.9311	1999.8709	1000.4391	**E**	462.2347	231.6210	445.2082	223.1077	444.2241	222.6157	**3**
**19**	2203.9608	1102.4840	2186.9342	1093.9707	2185.9502	1093.4787	**W**	***333.1921***	167.0997	316.1656	158.5864			**2**
**20**							**K**	147.1128	74.0600	130.0863	65.5468			**1**

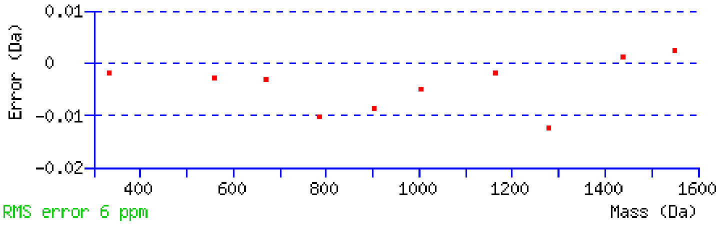

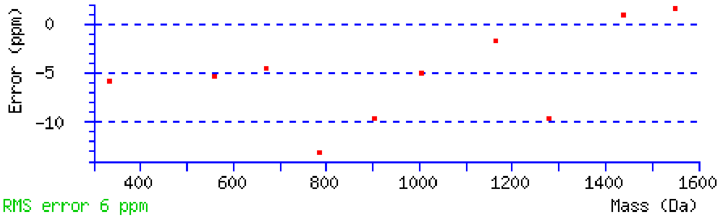

NCBI **BLAST** search of DNRGNLLQCICTGNGRGEWK
(Parameters: blastp, nr protein database, expect=20000, no filter, PAM30)
Other BLAST web gateways

All matches to this query

Score	Mr(calc)	Delta	Sequence	Site Analysis
42.0	2349.0590	0.0000	DNRGNLLQCICTGNGRGEWK	Deamidated N5, N14 48.79%
42.0	2349.0590	0.0000	DNRGNLLQCICTGNGRGEWK	Deamidated N2, N14 48.79%
28.9	2349.0590	0.0000	DNRGNLLQCICTGNGRGEWK	Deamidated Q8, N14 2.41%
4.7	2349.0590	0.0000	DNRGNLLQCICTGNGRGEWK	Deamidated N2, Q8 0.01%
4.0	2349.0590	0.0000	DNRGNLLQCICTGNGRGEWK	Deamidated N5, Q8 0.01%

Mascot: http://www.matrixscience.com/

Mascot Search Results

Peptide View

MS/MS Fragmentation of **EDRVPHSRNSITLTNLTPGTEYVVSIVALNGR**
Found in **P02751** in **uni_human_nr**, FINC_HUMAN Fibronectin OS=Homo sapiens GN=FN1 PE=1 SV=4

Match to Query 18517: 3509.813470 from(702.969970,5+) intensity(196140.3125) rtinseconds(3014) scans(18986) index(15255)
Title: 130806_HPL_Human_Plaque_BR1_TR2_05_Spectrum038051_scans__18986_RTINSECONDS=3014
Data file E:\\RAW\\HumanPlaques\\mgf\\T\\1D\\T130806_HPL_Human_Plaque_BR1_TR2_05.mgf

Click mouse within plot area to zoom in by factor of two about that point
Or, Plot from 0 to 2600 Da Full range
Label all possible matches ○ Label matches used for scoring ⊙
Show Y-axis ☐

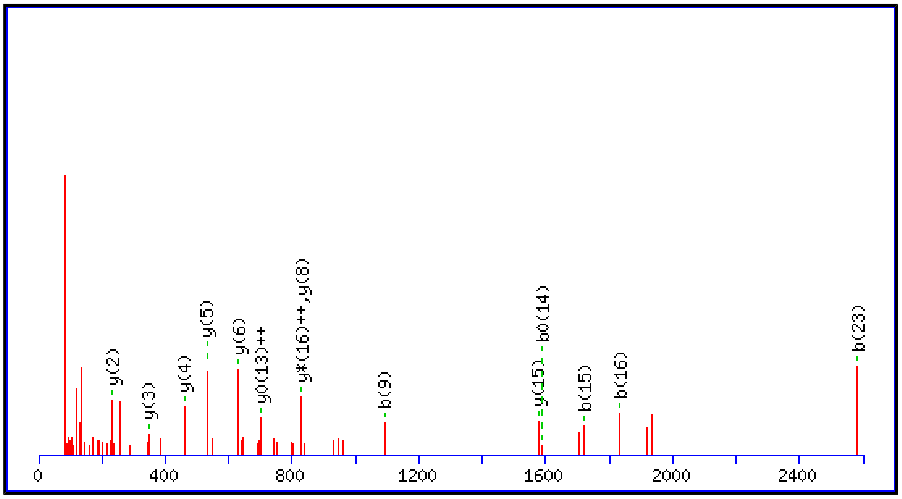

Monoisotopic mass of neutral peptide Mr(calc): 3509.8114
Fixed modifications: Carbamidomethyl (C) (apply to specified residues or termini only)
Variable modifications:
N9 : Deamidated (NQ)
N30 : Deamidated (NQ)
Ions Score: 51 **Expect:** 0.00046
Matches : 14/354 fragment ions using 16 most intense peaks (help)

#	b	b^{++}	b*	b^{*++}	b^0	b^{0++}	Seq.	y	y^{++}	y*	y^{*++}	y^0	y^{0++}	#
1	130.0499	65.5286			112.0393	56.5233	E							32
2	245.0768	123.0420			227.0662	114.0368	D	3381.7761	1691.3917	3364.7496	1682.8784	3363.7655	1682.3864	31
3	401.1779	201.0926	384.1514	192.5793	383.1674	192.0873	R	3266.7492	1633.8782	3249.7226	1625.3649	3248.7386	1624.8729	30
4	500.2463	250.6268	483.2198	242.1135	482.2358	241.6215	V	3110.6481	1555.8277	3093.6215	1547.3144	3092.6375	1546.8224	29
5	597.2991	299.1532	580.2726	290.6399	579.2885	290.1479	P	3011.5796	1506.2935	2994.5531	1497.7802	2993.5691	1497.2882	28
6	734.3580	367.6826	717.3315	359.1694	716.3474	358.6774	H	2914.5269	1457.7671	2897.5003	1449.2538	2896.5163	1448.7618	27
7	821.3900	411.1987	804.3635	402.6854	803.3795	402.1934	S	2777.4680	1389.2376	2760.4414	1380.7243	2759.4574	1380.2323	26
8	977.4912	489.2492	960.4646	480.7359	959.4806	480.2439	R	2690.4359	1345.7216	2673.4094	1337.2083	2672.4254	1336.7163	25
9	***1092.5181***	546.7627	1075.4915	538.2494	1074.5075	537.7574	N	2534.3348	1267.6711	2517.3083	1259.1578	2516.3243	1258.6658	24
10	1179.5501	590.2787	1162.5236	581.7654	1161.5396	581.2734	S	2419.3079	1210.1576	2402.2813	1201.6443	2401.2973	1201.1523	23
11	1292.6342	646.8207	1275.6076	638.3075	1274.6236	637.8154	I	2332.2759	1166.6416	2315.2493	1158.1283	2314.2653	1157.6363	22
12	1393.6819	697.3446	1376.6553	688.8313	1375.6713	688.3393	T	2219.1918	1110.0995	2202.1652	1101.5863	2201.1812	1101.0943	21
13	1506.7659	753.8866	1489.7394	745.3733	1488.7554	744.8813	L	2118.1441	1059.5757	2101.1176	1051.0624	2100.1335	1050.5704	20
14	1607.8136	804.4104	1590.7871	795.8972	1589.8030	795.4052	T	2005.0600	1003.0337	1988.0335	994.5204	1987.0495	994.0284	19
15	***1721.8565***	861.4319	1704.8300	852.9186	1703.8460	852.4266	N	1904.0124	952.5098	1886.9858	943.9965	1886.0018	943.5045	18
16	***1834.9406***	917.9739	1817.9141	909.4607	1816.9300	908.9687	L	1789.9694	895.4884	1772.9429	886.9751	1771.9589	886.4831	17
17	1935.9883	968.4978	1918.9617	959.9845	1917.9777	959.4925	T	1676.8854	838.9463	1659.8588	830.4331	1658.8748	829.9410	16

**18**	2033.0410	1017.0242	2016.0145	1008.5109	2015.0305	1008.0189	**P**	***1575.8377***	788.4225	1558.8112	779.9092	1557.8271	779.4172	**15**
**19**	2090.0625	1045.5349	2073.0360	1037.0216	2072.0519	1036.5296	**G**	1478.7849	739.8961	1461.7584	731.3828	1460.7744	730.8908	**14**
**20**	2191.1102	1096.0587	2174.0836	1087.5455	2173.0996	1087.0534	**T**	1421.7635	711.3854	1404.7369	702.8721	1403.7529	702.3801	**13**
**21**	2320.1528	1160.5800	2303.1262	1152.0668	2302.1422	1151.5747	**E**	1320.7158	660.8615	1303.6892	652.3483	1302.7052	651.8563	**12**
**22**	2483.2161	1242.1117	2466.1896	1233.5984	2465.2055	1233.1064	**Y**	1191.6732	596.3402	1174.6467	587.8270	1173.6626	587.3350	**11**
**23**	***2582.2845***	1291.6459	2565.2580	1283.1326	2564.2740	1282.6406	**V**	1028.6099	514.8086	1011.5833	506.2953	1010.5993	505.8033	**10**
**24**	2681.3529	1341.1801	2664.3264	1332.6668	2663.3424	1332.1748	**V**	929.5415	465.2744	912.5149	456.7611	911.5309	456.2691	**9**
**25**	2768.3850	1384.6961	2751.3584	1376.1828	2750.3744	1375.6908	**S**	***830.4730***	415.7402	813.4465	407.2269	812.4625	406.7349	**8**
**26**	2881.4690	1441.2382	2864.4425	1432.7249	2863.4585	1432.2329	**I**	743.4410	372.2241	726.4145	363.7109			**7**
**27**	2980.5374	1490.7724	2963.5109	1482.2591	2962.5269	1481.7671	**V**	***630.3570***	315.6821	613.3304	307.1688			**6**
**28**	3051.5746	1526.2909	3034.5480	1517.7776	3033.5640	1517.2856	**A**	***531.2885***	266.1479	514.2620	257.6346			**5**
**29**	3164.6586	1582.8329	3147.6321	1574.3197	3146.6481	1573.8277	**L**	***460.2514***	230.6293	443.2249	222.1161			**4**
**30**	3279.6856	1640.3464	3262.6590	1631.8331	3261.6750	1631.3411	**N**	***347.1674***	174.0873	330.1408	165.5740			**3**
**31**	3336.7070	1668.8572	3319.6805	1660.3439	3318.6965	1659.8519	**G**	***232.1404***	116.5738	215.1139	108.0606			**2**
**32**							**R**	175.1190	88.0631	158.0924	79.5498			**1**

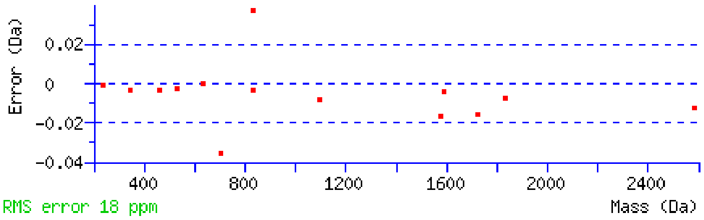

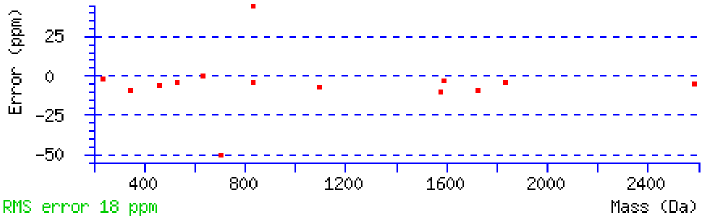

NCBI **BLAST** search of EDRVPHSRNSITLTNLTPGTEYVVSIVALNGR
(Parameters: blastp, nr protein database, expect=20000, no filter, PAM30)
Other BLAST web gateways

All matches to this query

Score	Mr(calc)	Delta	Sequence	Site Analysis
50.9	3509.8114	0.0021	EDRVPHSRNSITLTNLTPGTEYVVSIVALNGR	Deamidated N9, N30 79.36%
45.1	3509.8114	0.0021	EDRVPHSRNSITLTNLTPGTEYVVSIVALNGR	Deamidated N15, N30 20.64%
31.3	3508.8274	0.9861	EDRVPHSRNSITLTNLTPGTEYVVSIVALNGR	
5.1	3508.8274	0.9861	EDRVPHSRNSITLTNLTPGTEYVVSIVALNGR	
1.1	3508.8274	0.9861	EDRVPHSRNSITLTNLTPGTEYVVSIVALNGR	

Mascot: http://www.matrixscience.com/

Mascot Search Results

Peptide View

MS/MS Fragmentation of **EDRVPHSRNSITLTNLTPGTEYVVSIVALNGR**
Found in **P02751** in **uni_human_nr**, FINC_HUMAN Fibronectin OS=Homo sapiens GN=FN1 PE=1 SV=4

Match to Query 16507: 3509.804920 from(702.968260,5+) intensity(106178.4531) rtinseconds(2985) scans(17854) index(13618)
Title: 130809_HPL_Human_Plaque_BR1_TR3_08_Spectrum035228_scans__17854_RTINSECONDS=2985
Data file E:\\RAW\\HumanPlaques\\mgf\\T\\1D\\T130809_HPL_Human_Plaque_BR1_TR3_08.mgf

Click mouse within plot area to zoom in by factor of two about that point
Or, [Plot from] [0] to [2000] Da [Full range]
Label all possible matches ○ Label matches used for scoring ⊙
Show Y-axis ☐

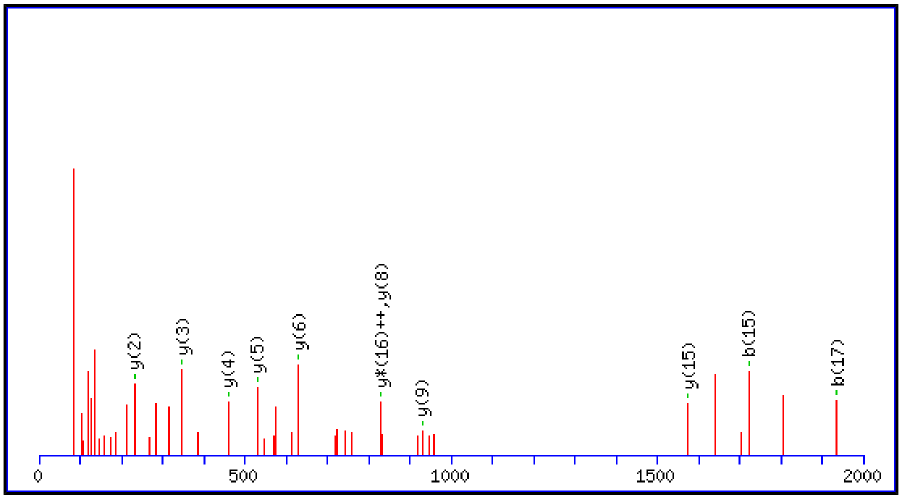

Monoisotopic mass of neutral peptide Mr(calc): 3509.8114
Fixed modifications: Carbamidomethyl (C) (apply to specified residues or termini only)
Variable modifications:
N15 : Deamidated (NQ)
N30 : Deamidated (NQ)
Ions Score: 49 **Expect:** 0.00088
Matches : 11/354 fragment ions using 14 most intense peaks (help)

#	b	b^{++}	b^{*}	b^{*++}	b^{0}	b^{0++}	Seq.	y	y^{++}	y^{*}	y^{*++}	y^{0}	y^{0++}	#
1	130.0499	65.5286			112.0393	56.5233	E							32
2	245.0768	123.0420			227.0662	114.0368	D	3381.7761	1691.3917	3364.7496	1682.8784	3363.7655	1682.3864	31
3	401.1779	201.0926	384.1514	192.5793	383.1674	192.0873	R	3266.7492	1633.8782	3249.7226	1625.3649	3248.7386	1624.8729	30
4	500.2463	250.6268	483.2198	242.1135	482.2358	241.6215	V	3110.6481	1555.8277	3093.6215	1547.3144	3092.6375	1546.8224	29
5	597.2991	299.1532	580.2726	290.6399	579.2885	290.1479	P	3011.5796	1506.2935	2994.5531	1497.7802	2993.5691	1497.2882	28
6	734.3580	367.6826	717.3315	359.1694	716.3474	358.6774	H	2914.5269	1457.7671	2897.5003	1449.2538	2896.5163	1448.7618	27
7	821.3900	411.1987	804.3635	402.6854	803.3795	402.1934	S	2777.4680	1389.2376	2760.4414	1380.7243	2759.4574	1380.2323	26
8	977.4912	489.2492	960.4646	480.7359	959.4806	480.2439	R	2690.4359	1345.7216	2673.4094	1337.2083	2672.4254	1336.7163	25
9	1091.5341	546.2707	1074.5075	537.7574	1073.5235	537.2654	N	2534.3348	1267.6711	2517.3083	1259.1578	2516.3243	1258.6658	24
10	1178.5661	589.7867	1161.5396	581.2734	1160.5555	580.7814	S	2420.2919	1210.6496	2403.2654	1202.1363	2402.2813	1201.6443	23
11	1291.6502	646.3287	1274.6236	637.8154	1273.6396	637.3234	I	2333.2599	1167.1336	2316.2333	1158.6203	2315.2493	1158.1283	22
12	1392.6979	696.8526	1375.6713	688.3393	1374.6873	687.8473	T	2220.1758	1110.5915	2203.1493	1102.0783	2202.1652	1101.5863	21
13	1505.7819	753.3946	1488.7554	744.8813	1487.7713	744.3893	L	2119.1281	1060.0677	2102.1016	1051.5544	2101.1176	1051.0624	20
14	1606.8296	803.9184	1589.8030	795.4052	1588.8190	794.9132	T	2006.0441	1003.5257	1989.0175	995.0124	1988.0335	994.5204	19
15	1721.8565	861.4319	1704.8300	852.9186	1703.8460	852.4266	N	1904.9964	953.0018	1887.9698	944.4886	1886.9858	943.9965	18
16	1834.9406	917.9739	1817.9141	909.4607	1816.9300	908.9687	L	1789.9694	895.4884	1772.9429	886.9751	1771.9589	886.4831	17
17	1935.9883	968.4978	1918.9617	959.9845	1917.9777	959.4925	T	1676.8854	838.9463	1659.8588	830.4331	1658.8748	829.9410	16

**18**	2033.0410	1017.0242	2016.0145	1008.5109	2015.0305	1008.0189	**P**	***1575.8377***	788.4225	1558.8112	779.9092	1557.8271	779.4172	**15**
**19**	2090.0625	1045.5349	2073.0360	1037.0216	2072.0519	1036.5296	**G**	1478.7849	739.8961	1461.7584	731.3828	1460.7744	730.8908	**14**
**20**	2191.1102	1096.0587	2174.0836	1087.5455	2173.0996	1087.0534	**T**	1421.7635	711.3854	1404.7369	702.8721	1403.7529	702.3801	**13**
**21**	2320.1528	1160.5800	2303.1262	1152.0668	2302.1422	1151.5747	**E**	1320.7158	660.8615	1303.6892	652.3483	1302.7052	651.8563	**12**
**22**	2483.2161	1242.1117	2466.1896	1233.5984	2465.2055	1233.1064	**Y**	1191.6732	596.3402	1174.6467	587.8270	1173.6626	587.3350	**11**
**23**	2582.2845	1291.6459	2565.2580	1283.1326	2564.2740	1282.6406	**V**	1028.6099	514.8086	1011.5833	506.2953	1010.5993	505.8033	**10**
**24**	2681.3529	1341.1801	2664.3264	1332.6668	2663.3424	1332.1748	**V**	***929.5415***	465.2744	912.5149	456.7611	911.5309	456.2691	**9**
**25**	2768.3850	1384.6961	2751.3584	1376.1828	2750.3744	1375.6908	**S**	***830.4730***	415.7402	813.4465	407.2269	812.4625	406.7349	**8**
**26**	2881.4690	1441.2382	2864.4425	1432.7249	2863.4585	1432.2329	**I**	743.4410	372.2241	726.4145	363.7109			**7**
**27**	2980.5374	1490.7724	2963.5109	1482.2591	2962.5269	1481.7671	**V**	***630.3570***	315.6821	613.3304	307.1688			**6**
**28**	3051.5746	1526.2909	3034.5480	1517.7776	3033.5640	1517.2856	**A**	***531.2885***	266.1479	514.2620	257.6346			**5**
**29**	3164.6586	1582.8329	3147.6321	1574.3197	3146.6481	1573.8277	**L**	***460.2514***	230.6293	443.2249	222.1161			**4**
**30**	3279.6856	1640.3464	3262.6590	1631.8331	3261.6750	1631.3411	**N**	***347.1674***	174.0873	330.1408	165.5740			**3**
**31**	3336.7070	1668.8572	3319.6805	1660.3439	3318.6965	1659.8519	**G**	***232.1404***	116.5738	215.1139	108.0606			**2**
**32**							**R**	175.1190	88.0631	158.0924	79.5498			**1**

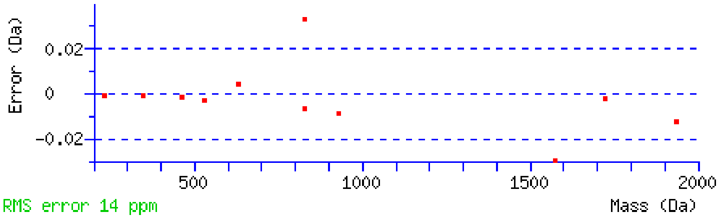

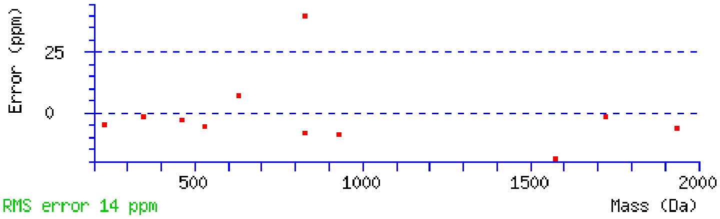

NCBI **BLAST** search of EDRVPHSRNSITLTNLTPGTEYVVSIVALNGR
(Parameters: blastp, nr protein database, expect=20000, no filter, PAM30)
Other BLAST web gateways

All matches to this query

Score	Mr(calc)	Delta	Sequence	Site Analysis
48.7	3509.8114	-0.0065	EDRVPHSRNSITLTNLTPGTEYVVSIVALNGR	Deamidated N15, N30 35.79%
48.7	3509.8114	-0.0065	EDRVPHSRNSITLTNLTPGTEYVVSIVALNGR	Deamidated N9, N30 35.79%

Mascot: http://www.matrixscience.com/

Mascot Search Results

Peptide View

MS/MS Fragmentation of **GNLLQCICTGNGR**
Found in **P02751** in **uni_human_nr**, FINC_HUMAN Fibronectin OS=Homo sapiens GN=FN1 PE=1 SV=4

Match to Query 2992: 1463.652068 from(732.833310,2+) intensity(122796.8984) rtinseconds(2314) scans(12772) index(9512)
Title: 130806_HPL_Human_Plaque_BR2_TR2_13_Spectrum030145_scans__12772_RTINSECONDS=2314
Data file E:\\RAW\\HumanPlaques\\mgf\\T\\1D\\T130806_HPL_Human_Plaque_BR2_TR2_13.mgf

Click mouse within plot area to zoom in by factor of two about that point
Or, Plot from 50 to 950 Da Full range
Label all possible matches ○ Label matches used for scoring ⦿
Show Y-axis ☐

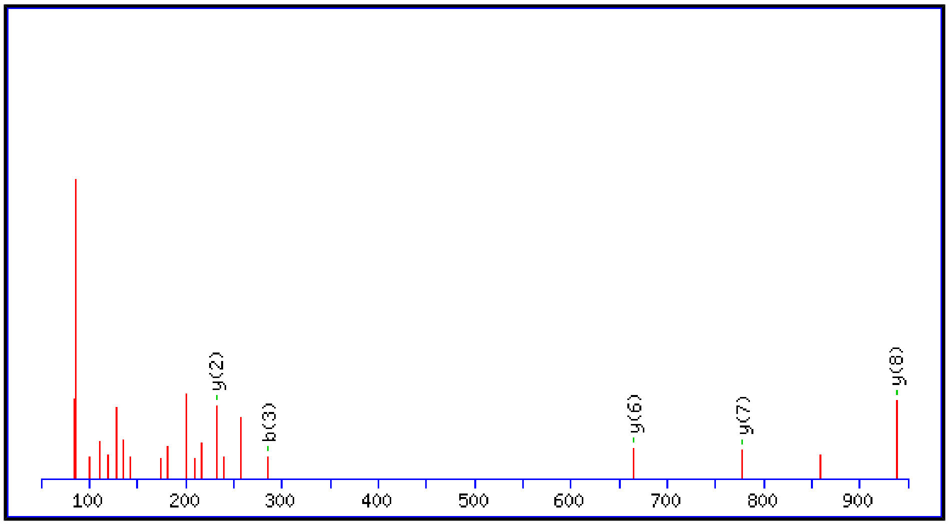

Monoisotopic mass of neutral peptide Mr(calc): 1463.6497
Fixed modifications: Carbamidomethyl (C) (apply to specified residues or termini only)
Variable modifications:
N2 : Deamidated (NQ)
N11 : Deamidated (NQ)
Ions Score: 18 **Expect:** 0.53
Matches : 5/118 fragment ions using 9 most intense peaks (help)

#	b	b^{++}	b*	b^{*++}	b^0	b^{0++}	Seq.	y	y^{++}	y*	y^{*++}	y^0	y^{0++}	#
1	58.0287	29.5180					G							13
2	173.0557	87.0315	156.0291	78.5182			N	1407.6355	704.3214	1390.6090	695.8081	1389.6249	695.3161	12
3	**286.1397**	143.5735	269.1132	135.0602			L	1292.6086	646.8079	1275.5820	638.2946	1274.5980	637.8026	11
4	399.2238	200.1155	382.1973	191.6023			L	1179.5245	590.2659	1162.4980	581.7526	1161.5139	581.2606	10
5	527.2824	264.1448	510.2558	255.6316			Q	1066.4404	533.7239	1049.4139	525.2106	1048.4299	524.7186	9
6	687.3130	344.1602	670.2865	335.6469			C	***938.3819***	469.6946	921.3553	461.1813	920.3713	460.6893	8
7	800.3971	400.7022	783.3706	392.1889			I	***778.3512***	389.6792	761.3247	381.1660	760.3406	380.6740	7
8	960.4278	480.7175	943.4012	472.2042			C	***665.2672***	333.1372	648.2406	324.6239	647.2566	324.1319	6
9	1061.4754	531.2414	1044.4489	522.7281	1043.4649	522.2361	T	505.2365	253.1219	488.2100	244.6086	487.2259	244.1166	5
10	1118.4969	559.7521	1101.4703	551.2388	1100.4863	550.7468	G	404.1888	202.5980	387.1623	194.0848			4
11	1233.5238	617.2656	1216.4973	608.7523	1215.5133	608.2603	N	347.1674	174.0873	330.1408	165.5740			3
12	1290.5453	645.7763	1273.5188	637.2630	1272.5347	636.7710	G	***232.1404***	116.5738	215.1139	108.0606			2
13							R	175.1190	88.0631	158.0924	79.5498			1

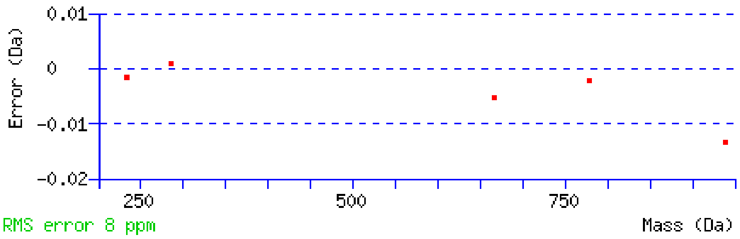

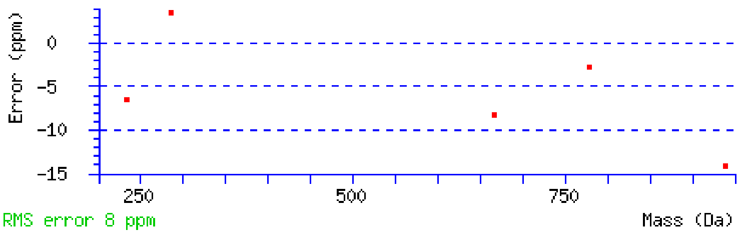

NCBI **BLAST** search of GNLLQCICTGNGR
(Parameters: blastp, nr protein database, expect=20000, no filter, PAM30)
Other BLAST web gateways

All matches to this query

Score	Mr(calc)	Delta	Sequence
18.3	1463.6497	0.0024	GNLLQCICTGNGR
15.3	1463.6497	0.0024	GNLLQCICTGNGR

Mascot: http://www.matrixscience.com/

Mascot Search Results

Peptide View

MS/MS Fragmentation of **GNLLQCICTGNGR**
Found in **P02751** in **uni_human_nr**, FINC_HUMAN Fibronectin OS=Homo sapiens GN=FN1 PE=1 SV=4

Match to Query 3399: 1462.664888 from(732.339720,2+) intensity(3667816.5000) rtinseconds(2081) scans(11852) index(8779)
Title: 130806_HPL_Human_Plaque_BR1_TR2_07_Spectrum030750_scans__11852_RTINSECONDS=2081
Data file E:\\RAW\\HumanPlaques\\mgf\\T\\1D\\T130806_HPL_Human_Plaque_BR1_TR2_07.mgf

Click mouse within plot area to zoom in by factor of two about that point
Or, Plot from 0 to 1200 Da Full range
Label all possible matches ○ Label matches used for scoring ⦿
Show Y-axis ☐

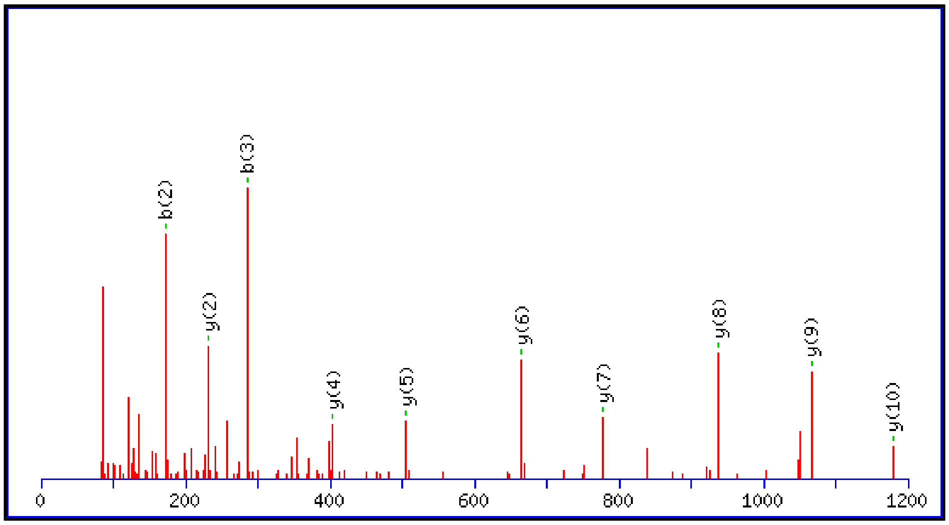

Monoisotopic mass of neutral peptide Mr(calc): 1462.6657
Fixed modifications: Carbamidomethyl (C) (apply to specified residues or termini only)
Variable modifications:
N11 : Deamidated (NQ)
Ions Score: 78 **Expect:** 6.5e-007
Matches : 10/118 fragment ions using 11 most intense peaks (help)

#	b	b^{++}	b*	b^{*++}	b^0	b^{0++}	Seq.	y	y^{++}	y*	y^{*++}	y^0	y^{0++}	#
1	58.0287	29.5180					G							13
2	**172.0717**	86.5395	155.0451	78.0262			N	1406.6515	703.8294	1389.6249	695.3161	1388.6409	694.8241	12
3	**285.1557**	143.0815	268.1292	134.5682			L	1292.6086	646.8079	1275.5820	638.2946	1274.5980	637.8026	11
4	398.2398	199.6235	381.2132	191.1103			L	***1179.5245***	590.2659	1162.4980	581.7526	1161.5139	581.2606	10
5	526.2984	263.6528	509.2718	255.1396			Q	***1066.4404***	533.7239	1049.4139	525.2106	1048.4299	524.7186	9
6	686.3290	343.6681	669.3025	335.1549			C	***938.3819***	469.6946	921.3553	461.1813	920.3713	460.6893	8
7	799.4131	400.2102	782.3865	391.6969			I	***778.3512***	389.6792	761.3247	381.1660	760.3406	380.6740	7
8	959.4437	480.2255	942.4172	471.7122			C	***665.2672***	333.1372	648.2406	324.6239	647.2566	324.1319	6
9	1060.4914	530.7493	1043.4649	522.2361	1042.4808	521.7441	T	***505.2365***	253.1219	488.2100	244.6086	487.2259	244.1166	5
10	1117.5129	559.2601	1100.4863	550.7468	1099.5023	550.2548	G	***404.1888***	202.5980	387.1623	194.0848			4
11	1232.5398	616.7735	1215.5133	608.2603	1214.5293	607.7683	N	347.1674	174.0873	330.1408	165.5740			3
12	1289.5613	645.2843	1272.5347	636.7710	1271.5507	636.2790	G	***232.1404***	116.5738	215.1139	108.0606			2
13							R	175.1190	88.0631	158.0924	79.5498			1

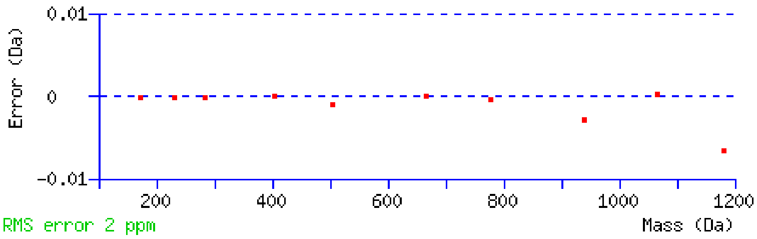

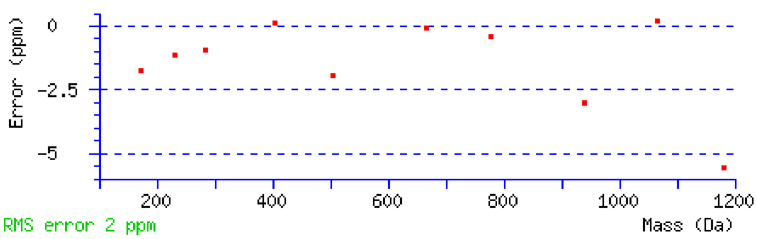

NCBI **BLAST** search of GNLLQCICTGNGR
(Parameters: blastp, nr protein database, expect=20000, no filter, PAM30)
Other BLAST web gateways

All matches to this query

Score	Mr(calc)	Delta	Sequence	Site Analysis
78.3	1462.6657	-0.0008	GNLLQCICTGNGR	Deamidated N11 100.00%
11.1	1462.6657	-0.0008	GNLLQCICTGNGR	Deamidated Q5 0.00%

Mascot: http://www.matrixscience.com/

Mascot Search Results

Peptide View

MS/MS Fragmentation of **GNLLQCICTGNGR**
Found in **P02751** in **uni_human_nr**, FINC_HUMAN Fibronectin OS=Homo sapiens GN=FN1 PE=1 SV=4

Match to Query 4104: 1461.679288 from(731.846920,2+) intensity(225957232.0000) rtinseconds(1757) scans(9968) index(7285)
Title: 130801_HPL_Human_Plaque_BR2_TR1_02_Spectrum031440_scans__9968_RTINSECONDS=1757
Data file E:\\RAW\\HumanPlaques\\mgf\\T\\1D\\T130801_HPL_Human_Plaque_BR2_TR1_02.mgf

Click mouse within plot area to zoom in by factor of two about that point
Or, Plot from 0 to 1200 Da Full range
Label all possible matches ○ Label matches used for scoring ⊙
Show Y-axis ☐

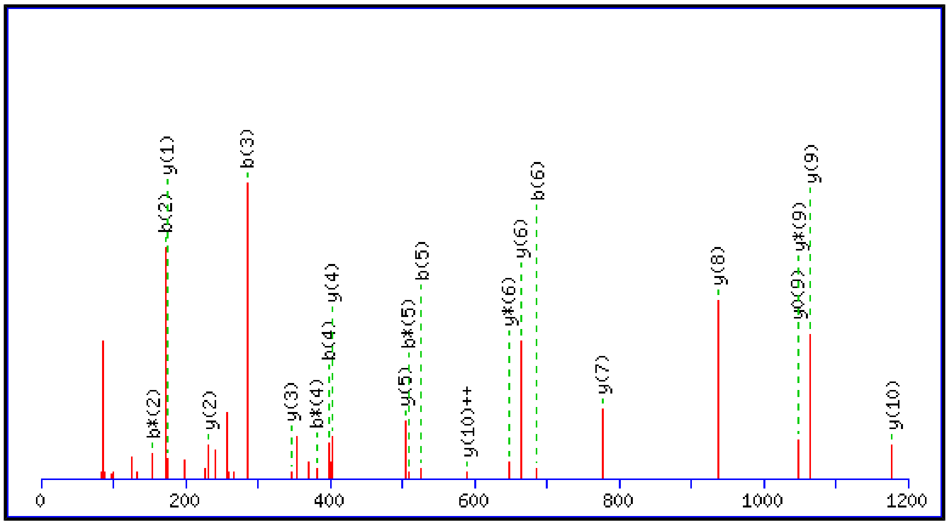

Monoisotopic mass of neutral peptide Mr(calc): 1461.6817
Fixed modifications: Carbamidomethyl (C) (apply to specified residues or termini only)
Ions Score: 84 **Expect:** 2.2e-007
Matches : 22/118 fragment ions using 33 most intense peaks (help)

#	b	b^{++}	b*	b^{*++}	b^{0}	b^{0++}	Seq.	y	y^{++}	y*	y^{*++}	y^{0}	y^{0++}	#
1	58.0287	29.5180					G							13
2	**172.0717**	86.5395	155.0451	78.0262			N	1405.6675	703.3374	1388.6409	694.8241	1387.6569	694.3321	12
3	**285.1557**	143.0815	268.1292	134.5682			L	1291.6246	646.3159	1274.5980	637.8026	1273.6140	637.3106	11
4	**398.2398**	199.6235	381.2132	191.1103			L	***1178.5405***	589.7739	1161.5139	581.2606	1160.5299	580.7686	10
5	**526.2984**	263.6528	509.2718	255.1396			Q	***1065.4564***	533.2319	1048.4299	524.7186	1047.4459	524.2266	9
6	**686.3290**	343.6681	669.3025	335.1549			C	***937.3978***	469.2026	920.3713	460.6893	919.3873	460.1973	8
7	799.4131	400.2102	782.3865	391.6969			I	***777.3672***	389.1872	760.3406	380.6740	759.3566	380.1820	7
8	959.4437	480.2255	942.4172	471.7122			C	***664.2831***	332.6452	647.2566	324.1319	646.2726	323.6399	6
9	1060.4914	530.7493	1043.4649	522.2361	1042.4808	521.7441	T	***504.2525***	252.6299	487.2259	244.1166	486.2419	243.6246	5
10	1117.5129	559.2601	1100.4863	550.7468	1099.5023	550.2548	G	***403.2048***	202.1060	386.1783	193.5928			4
11	1231.5558	616.2815	1214.5293	607.7683	1213.5452	607.2763	N	***346.1833***	173.5953	329.1568	165.0820			3
12	1288.5773	644.7923	1271.5507	636.2790	1270.5667	635.7870	G	***232.1404***	116.5738	215.1139	108.0606			2
13							R	***175.1190***	88.0631	158.0924	79.5498			1

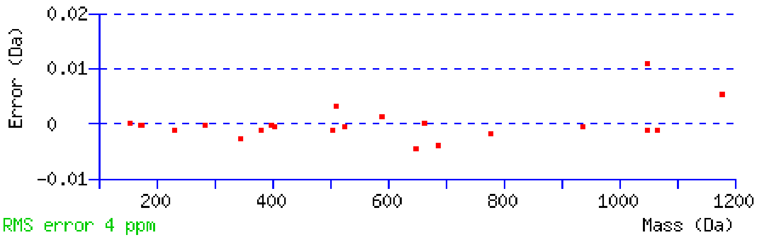

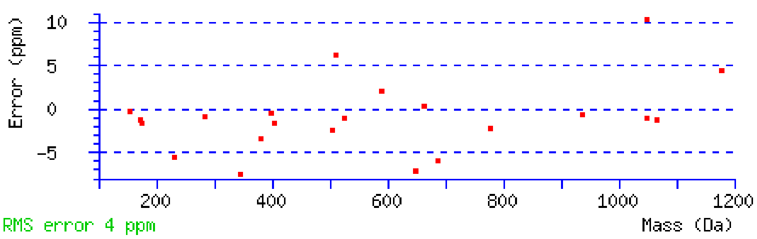

NCBI **BLAST** search of GNLLQCICTGNGR
(Parameters: blastp, nr protein database, expect=20000, no filter, PAM30)
Other BLAST web gateways

All matches to this query

Score	Mr(calc)	Delta	Sequence
83.9	1461.6817	-0.0024	GNLLQCICTGNGR
11.0	1461.6783	0.0010	QRLQMYNSQHR
0.8	1461.6735	0.0057	NGIPYLNQEEER
0.8	1461.6735	0.0057	NGIPYLNQEEER
0.5	1461.6857	-0.0064	QRICSFTTFMR
0.5	1461.6783	0.0010	QRLQMYNSQHR
0.5	1461.6783	0.0010	QRLQMYNSQHR

Mascot: http://www.matrixscience.com/

Mascot Search Results

Peptide View

MS/MS Fragmentation of **GNLLQCICTGNGR**
Found in **P02751** in **uni_human_nr**, FINC_HUMAN Fibronectin OS=Homo sapiens GN=FN1 PE=1 SV=4

Match to Query 4157: 1461.683232 from(488.235020,3+) intensity(7707136.5000) rtinseconds(1758) scans(10166) index(7551)
Title: 130801_HPL_Human_Plaque_BR1_TR1_02_Spectrum031558_scans__10166_RTINSECONDS=1758
Data file E:\\RAW\\HumanPlaques\\mgf\\T\\1D\\T130801_HPL_Human_Plaque_BR1_TR1_02.mgf

Click mouse within plot area to zoom in by factor of two about that point
Or, Plot from 50 to 1050 Da Full range
Label all possible matches ○ Label matches used for scoring ⊙
Show Y-axis ☐

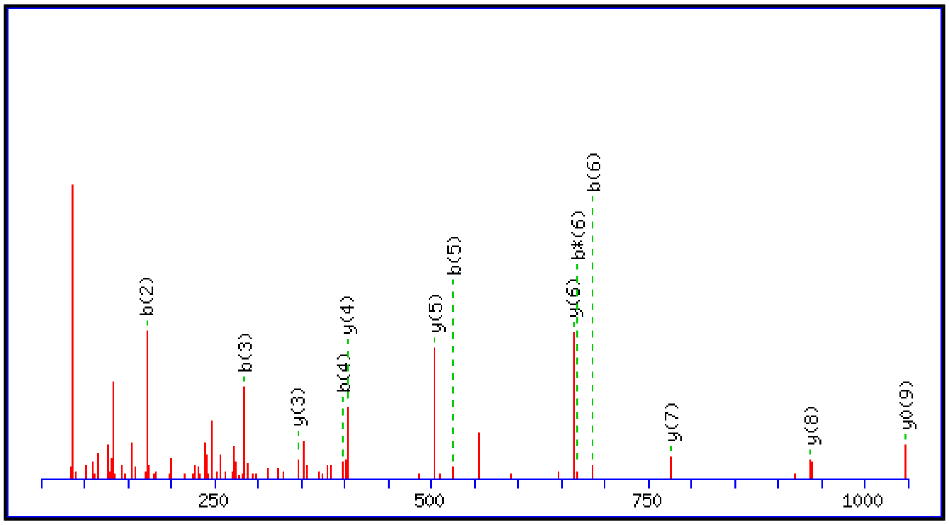

Monoisotopic mass of neutral peptide Mr(calc): 1461.6817
Fixed modifications: Carbamidomethyl (C) (apply to specified residues or termini only)
Ions Score: 44 **Expect:** 0.0021
Matches : 13/118 fragment ions using 24 most intense peaks (help)

#	b	b++	b*	b*++	b0	b0++	Seq.	y	y++	y*	y*++	y0	y0++	#
1	58.0287	29.5180					G							13
2	***172.0717***	86.5395	155.0451	78.0262			N	1405.6675	703.3374	1388.6409	694.8241	1387.6569	694.3321	12
3	***285.1557***	143.0815	268.1292	134.5682			L	1291.6246	646.3159	1274.5980	637.8026	1273.6140	637.3106	11
4	***398.2398***	199.6235	381.2132	191.1103			L	1178.5405	589.7739	1161.5139	581.2606	1160.5299	580.7686	10
5	***526.2984***	263.6528	509.2718	255.1396			Q	1065.4564	533.2319	1048.4299	524.7186	1047.4459	524.2266	9
6	***686.3290***	343.6681	669.3025	335.1549			C	***937.3978***	469.2026	920.3713	460.6893	919.3873	460.1973	8
7	799.4131	400.2102	782.3865	391.6969			I	***777.3672***	389.1872	760.3406	380.6740	759.3566	380.1820	7
8	959.4437	480.2255	942.4172	471.7122			C	***664.2831***	332.6452	647.2566	324.1319	646.2726	323.6399	6
9	1060.4914	530.7493	1043.4649	522.2361	1042.4808	521.7441	T	***504.2525***	252.6299	487.2259	244.1166	486.2419	243.6246	5
10	1117.5129	559.2601	1100.4863	550.7468	1099.5023	550.2548	G	***403.2048***	202.1060	386.1783	193.5928			4
11	1231.5558	616.2815	1214.5293	607.7683	1213.5452	607.2763	N	***346.1833***	173.5953	329.1568	165.0820			3
12	1288.5773	644.7923	1271.5507	636.2790	1270.5667	635.7870	G	232.1404	116.5738	215.1139	108.0606			2
13							R	175.1190	88.0631	158.0924	79.5498			1

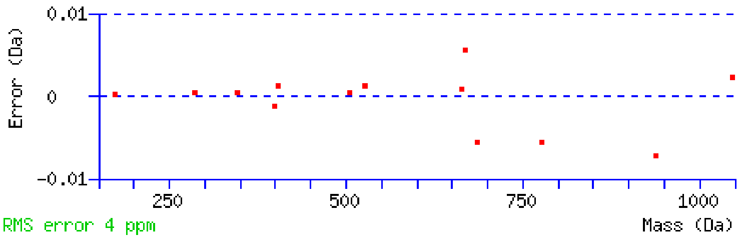

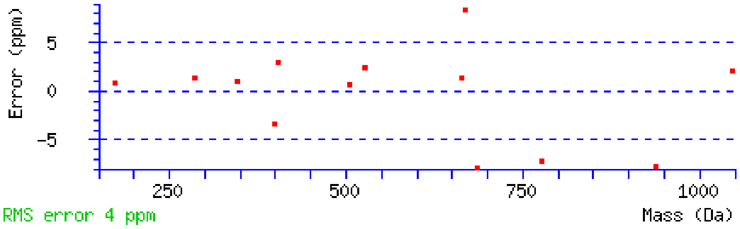

NCBI **BLAST** search of GNLLQCICTGNGR
(Parameters: blastp, nr protein database, expect=20000, no filter, PAM30)
Other BLAST web gateways

All matches to this query

Score	Mr(calc)	Delta	Sequence
44.5	1461.6817	0.0016	GNLLQCICTGNGR

Mascot: http://www.matrixscience.com/

Mascot Search Results

Peptide View

MS/MS Fragmentation of **GNLLQCICTGNGRGEWK**
Found in **P02751** in **uni_human_nr**, FINC_HUMAN Fibronectin OS=Homo sapiens GN=FN1 PE=1 SV=4

Match to Query 7478: 1963.888152 from(655.636660,3+) intensity(570374.1875) rtinseconds(2474) scans(14361) index(11049)
Title: 130809_HPL_Human_Plaque_BR2_TR3_09_Spectrum032376_scans__14361_RTINSECONDS=2474
Data file E:\\RAW\\HumanPlaques\\mgf\\T\\1D\\T130809_HPL_Human_Plaque_BR2_TR3_09.mgf

Click mouse within plot area to zoom in by factor of two about that point
Or, [Plot from] [0] to [1700] Da [Full range]
Label all possible matches ○ Label matches used for scoring ⊙
Show Y-axis ☐

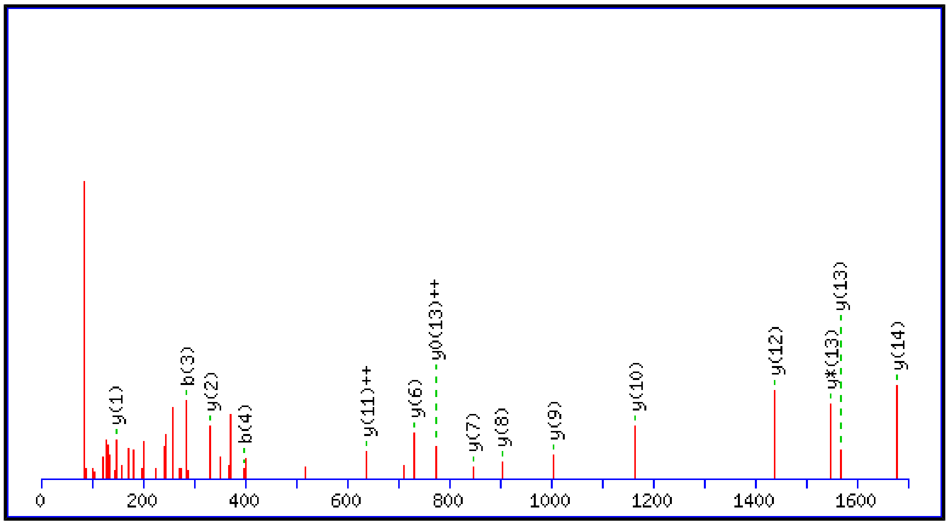

Monoisotopic mass of neutral peptide Mr(calc): 1963.8880
Fixed modifications: Carbamidomethyl (C) (apply to specified residues or termini only)
Variable modifications:
N2 : Deamidated (NQ)
N11 : Deamidated (NQ)
Ions Score: 67 **Expect:** 9.7e-006
Matches : 15/170 fragment ions using 27 most intense peaks (help)

#	b	b^{++}	b*	b^{*++}	b^{0}	b^{0++}	Seq.	y	y^{++}	y*	y^{*++}	y^{0}	y^{0++}	#
1	58.0287	29.5180					G							17
2	173.0557	87.0315	156.0291	78.5182			N	1907.8738	954.4406	1890.8473	945.9273	1889.8633	945.4353	16
3	**286.1397**	143.5735	269.1132	135.0602			L	1792.8469	896.9271	1775.8204	888.4138	1774.8363	887.9218	15
4	**399.2238**	200.1155	382.1973	191.6023			L	***1679.7628***	840.3851	1662.7363	831.8718	1661.7523	831.3798	14
5	527.2824	264.1448	510.2558	255.6316			Q	***1566.6788***	783.8430	1549.6522	775.3298	1548.6682	774.8377	13
6	687.3130	344.1602	670.2865	335.6469			C	***1438.6202***	719.8137	1421.5936	711.3005	1420.6096	710.8085	12
7	800.3971	400.7022	783.3706	392.1889			I	1278.5895	639.7984	1261.5630	631.2851	1260.5790	630.7931	11
8	960.4278	480.7175	943.4012	472.2042			C	***1165.5055***	583.2564	1148.4789	574.7431	1147.4949	574.2511	10
9	1061.4754	531.2414	1044.4489	522.7281	1043.4649	522.2361	T	***1005.4748***	503.2411	988.4483	494.7278	987.4643	494.2358	9
10	1118.4969	559.7521	1101.4703	551.2388	1100.4863	550.7468	G	***904.4272***	452.7172	887.4006	444.2039	886.4166	443.7119	8
11	1233.5238	617.2656	1216.4973	608.7523	1215.5133	608.2603	N	***847.4057***	424.2065	830.3791	415.6932	829.3951	415.2012	7
12	1290.5453	645.7763	1273.5188	637.2630	1272.5347	636.7710	G	***732.3787***	366.6930	715.3522	358.1797	714.3682	357.6877	6
13	1446.6464	723.8268	1429.6199	715.3136	1428.6358	714.8216	R	675.3573	338.1823	658.3307	329.6690	657.3467	329.1770	5
14	1503.6679	752.3376	1486.6413	743.8243	1485.6573	743.3323	G	519.2562	260.1317	502.2296	251.6185	501.2456	251.1264	4
15	1632.7105	816.8589	1615.6839	808.3456	1614.6999	807.8536	E	462.2347	231.6210	445.2082	223.1077	444.2241	222.6157	3

**16**	1818.7898	909.8985	1801.7632	901.3853	1800.7792	900.8932	**W**	***333.1921***	167.0997	316.1656	158.5864			**2**
**17**							**K**	***147.1128***	74.0600	130.0863	65.5468			**1**

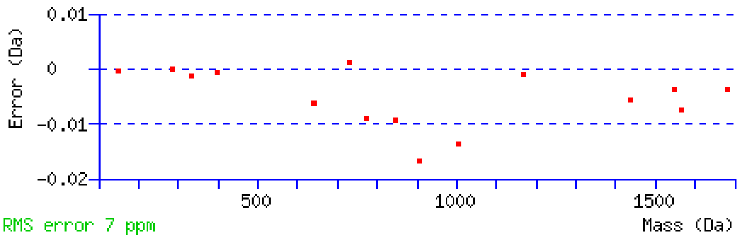

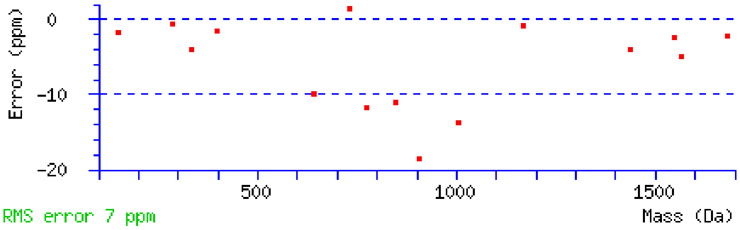

NCBI **BLAST** search of GNLLQCICTGNGRGEWK
(Parameters: blastp, nr protein database, expect=20000, no filter, PAM30)
Other BLAST web gateways

All matches to this query

Score	Mr(calc)	Delta	Sequence	Site Analysis
66.8	1963.8880	0.0001	GNLLQCICTGNGRGEWK	Deamidated N2, N11 99.93%
35.0	1963.8880	0.0001	GNLLQCICTGNGRGEWK	Deamidated Q5, N11 0.07%
11.8	1963.8880	0.0001	GNLLQCICTGNGRGEWK	Deamidated N2, Q5 0.00%

Mascot: http://www.matrixscience.com/

Mascot Search Results

Peptide View

MS/MS Fragmentation of **GNLLQCICTGNGRGEWK**
Found in **P02751** in **uni_human_nr**, FINC_HUMAN Fibronectin OS=Homo sapiens GN=FN1 PE=1 SV=4

Match to Query 8061: 1964.885142 from(655.968990,3+) intensity(593385.1250) rtinseconds(2423) scans(14099) index(10734)
Title: 130806_HPL_Human_Plaque_BR2_TR2_07_Spectrum032410_scans__14099_RTINSECONDS=2423
Data file E:\\RAW\\HumanPlaques\\mgf\\T\\1D\\T130806_HPL_Human_Plaque_BR2_TR2_07.mgf

Click mouse within plot area to zoom in by factor of two about that point
Or, Plot from 0 to 1700 Da Full range
Label all possible matches ○ Label matches used for scoring ⊙
Show Y-axis ☐

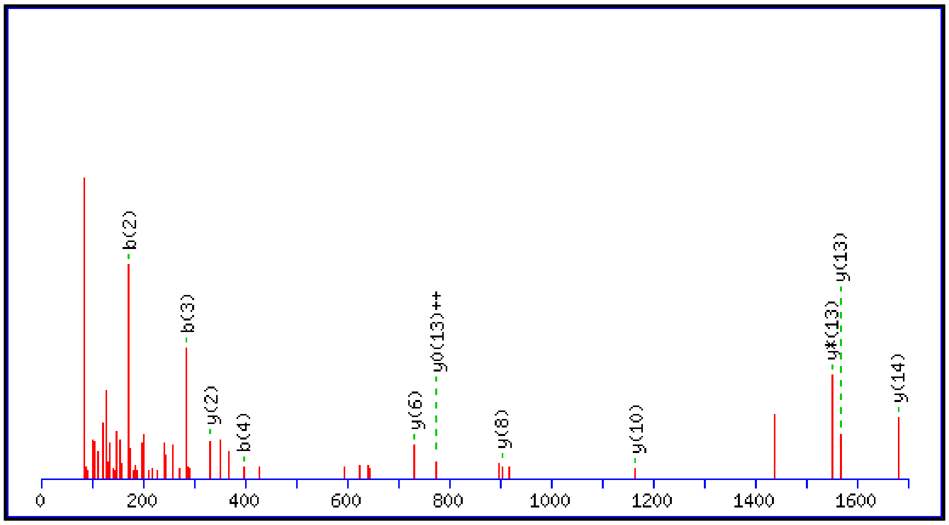

Monoisotopic mass of neutral peptide Mr(calc): 1963.8880
Fixed modifications: Carbamidomethyl (C) (apply to specified residues or termini only)
Variable modifications:
Q5 : Deamidated (NQ)
N11 : Deamidated (NQ)
Ions Score: 24 **Expect:** 0.17
Matches : 11/170 fragment ions using 24 most intense peaks (help)

#	b	b^{++}	b*	b^{*++}	b^0	b^{0++}	Seq.	y	y^{++}	y*	y^{*++}	y^0	y^{0++}	#
1	58.0287	29.5180					G							17
2	**172.0717**	86.5395	155.0451	78.0262			N	1907.8738	954.4406	1890.8473	945.9273	1889.8633	945.4353	16
3	**285.1557**	143.0815	268.1292	134.5682			L	1793.8309	897.4191	1776.8044	888.9058	1775.8204	888.4138	15
4	**398.2398**	199.6235	381.2132	191.1103			L	***1680.7469***	840.8771	1663.7203	832.3638	1662.7363	831.8718	14
5	527.2824	264.1448	510.2558	255.6316			Q	***1567.6628***	784.3350	1550.6362	775.8218	1549.6522	775.3298	13
6	687.3130	344.1602	670.2865	335.6469			C	1438.6202	719.8137	1421.5936	711.3005	1420.6096	710.8085	12
7	800.3971	400.7022	783.3706	392.1889			I	1278.5895	639.7984	1261.5630	631.2851	1260.5790	630.7931	11
8	960.4278	480.7175	943.4012	472.2042			C	***1165.5055***	583.2564	1148.4789	574.7431	1147.4949	574.2511	10
9	1061.4754	531.2414	1044.4489	522.7281	1043.4649	522.2361	T	1005.4748	503.2411	988.4483	494.7278	987.4643	494.2358	9
10	1118.4969	559.7521	1101.4703	551.2388	1100.4863	550.7468	G	***904.4272***	452.7172	887.4006	444.2039	886.4166	443.7119	8
11	1233.5238	617.2656	1216.4973	608.7523	1215.5133	608.2603	N	847.4057	424.2065	830.3791	415.6932	829.3951	415.2012	7
12	1290.5453	645.7763	1273.5188	637.2630	1272.5347	636.7710	G	***732.3787***	366.6930	715.3522	358.1797	714.3682	357.6877	6
13	1446.6464	723.8268	1429.6199	715.3136	1428.6358	714.8216	R	675.3573	338.1823	658.3307	329.6690	657.3467	329.1770	5
14	1503.6679	752.3376	1486.6413	743.8243	1485.6573	743.3323	G	519.2562	260.1317	502.2296	251.6185	501.2456	251.1264	4
15	1632.7105	816.8589	1615.6839	808.3456	1614.6999	807.8536	E	462.2347	231.6210	445.2082	223.1077	444.2241	222.6157	3

**16**	1818.7898	909.8985	1801.7632	901.3853	1800.7792	900.8932	**W**	*333.1921*	167.0997	316.1656	158.5864			**2**
**17**							**K**	147.1128	74.0600	130.0863	65.5468			**1**

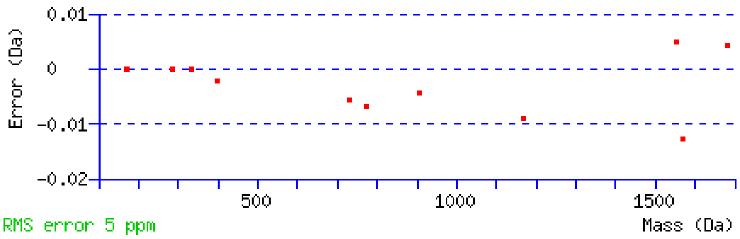

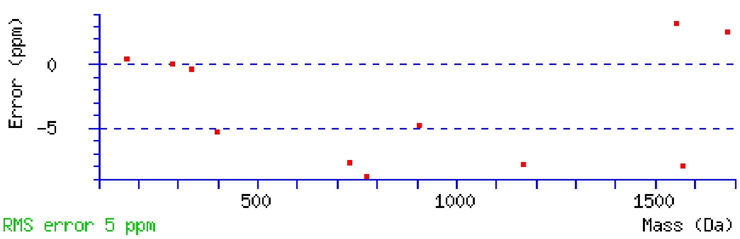

NCBI **BLAST** search of GNLLQCICTGNGRGEWK
(Parameters: blastp, nr protein database, expect=20000, no filter, PAM30)
Other BLAST web gateways

All matches to this query

Score	Mr(calc)	Delta	Sequence
24.3	1963.8880	0.9971	GNLLQCICTGNGRGEWK

Mascot: http://www.matrixscience.com/

Mascot Search Results

Peptide View

MS/MS Fragmentation of **GNLLQCICTGNGRGEWK**
Found in **P02751** in **uni_human_nr**, FINC_HUMAN Fibronectin OS=Homo sapiens GN=FN1 PE=1 SV=4

Match to Query 6523: 1963.882548 from(982.948550,2+) intensity(233518.5156) rtinseconds(2539) scans(14406) index(10752)
Title: 130801_HPL_Human_Plaque_BR1_TR1_10_Spectrum031716_scans__14406_RTINSECONDS=2539
Data file E:\\RAW\\HumanPlaques\\mgf\\T\\1D\\T130801_HPL_Human_Plaque_BR1_TR1_10.mgf

Click mouse within plot area to zoom in by factor of two about that point
Or, [Plot from] 50 to 950 Da [Full range]
Label all possible matches ○ Label matches used for scoring ⊙
Show Y-axis ☐

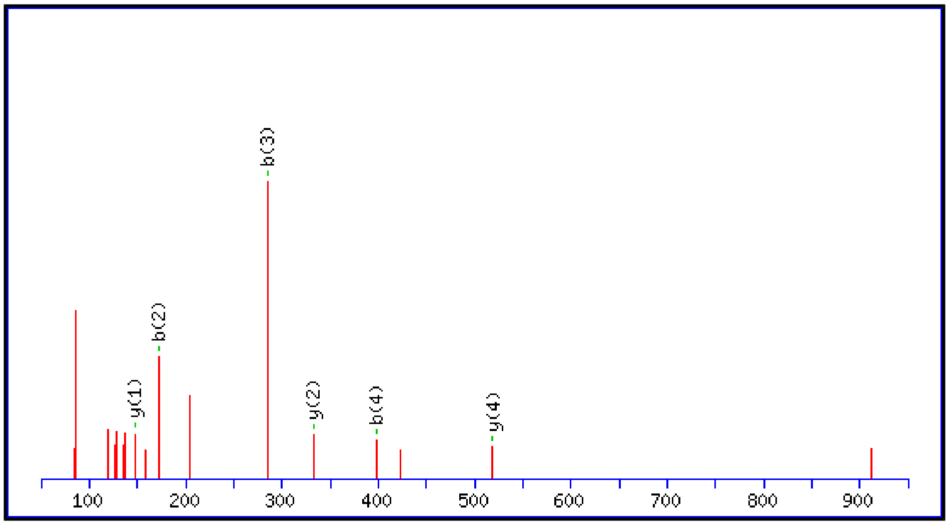

Monoisotopic mass of neutral peptide Mr(calc): 1963.8880
Fixed modifications: Carbamidomethyl (C) (apply to specified residues or termini only)
Variable modifications:
Q5 : Deamidated (NQ)
N11 : Deamidated (NQ)
Ions Score: 17 **Expect:** 0.83
Matches : 6/170 fragment ions using 13 most intense peaks (help)

#	b	b++	b*	b*++	b0	b0++	Seq.	y	y++	y*	y*++	y0	y0++	#
1	58.0287	29.5180					G							17
2	***172.0717***	86.5395	155.0451	78.0262			N	1907.8738	954.4406	1890.8473	945.9273	1889.8633	945.4353	16
3	***285.1557***	143.0815	268.1292	134.5682			L	1793.8309	897.4191	1776.8044	888.9058	1775.8204	888.4138	15
4	***398.2398***	199.6235	381.2132	191.1103			L	1680.7469	840.8771	1663.7203	832.3638	1662.7363	831.8718	14
5	527.2824	264.1448	510.2558	255.6316			Q	1567.6628	784.3350	1550.6362	775.8218	1549.6522	775.3298	13
6	687.3130	344.1602	670.2865	335.6469			C	1438.6202	719.8137	1421.5936	711.3005	1420.6096	710.8085	12
7	800.3971	400.7022	783.3706	392.1889			I	1278.5895	639.7984	1261.5630	631.2851	1260.5790	630.7931	11
8	960.4278	480.7175	943.4012	472.2042			C	1165.5055	583.2564	1148.4789	574.7431	1147.4949	574.2511	10
9	1061.4754	531.2414	1044.4489	522.7281	1043.4649	522.2361	T	1005.4748	503.2411	988.4483	494.7278	987.4643	494.2358	9
10	1118.4969	559.7521	1101.4703	551.2388	1100.4863	550.7468	G	904.4272	452.7172	887.4006	444.2039	886.4166	443.7119	8
11	1233.5238	617.2656	1216.4973	608.7523	1215.5133	608.2603	N	847.4057	424.2065	830.3791	415.6932	829.3951	415.2012	7
12	1290.5453	645.7763	1273.5188	637.2630	1272.5347	636.7710	G	732.3787	366.6930	715.3522	358.1797	714.3682	357.6877	6
13	1446.6464	723.8268	1429.6199	715.3136	1428.6358	714.8216	R	675.3573	338.1823	658.3307	329.6690	657.3467	329.1770	5
14	1503.6679	752.3376	1486.6413	743.8243	1485.6573	743.3323	G	***519.2562***	260.1317	502.2296	251.6185	501.2456	251.1264	4
15	1632.7105	816.8589	1615.6839	808.3456	1614.6999	807.8536	E	462.2347	231.6210	445.2082	223.1077	444.2241	222.6157	3

**16**	1818.7898	909.8985	1801.7632	901.3853	1800.7792	900.8932	**W**	***333.1921***	167.0997	316.1656	158.5864			**2**
**17**							**K**	***147.1128***	74.0600	130.0863	65.5468			**1**

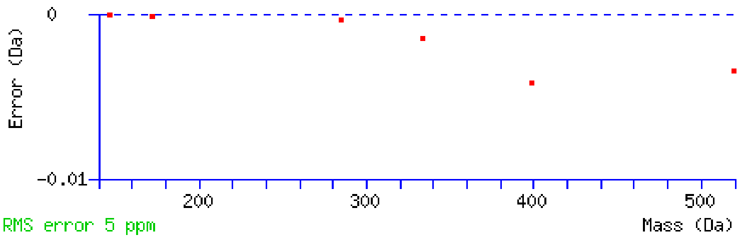

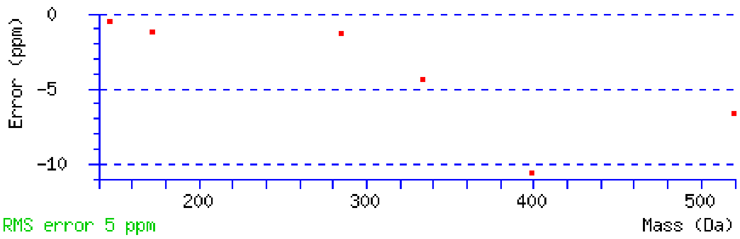

NCBI **BLAST** search of GNLLQCICTGNGRGEWK
(Parameters: blastp, nr protein database, expect=20000, no filter, PAM30)
Other BLAST web gateways

All matches to this query

Score	Mr(calc)	Delta	Sequence
17.3	1963.8880	-0.0055	GNLLQCICTGNGRGEWK
2.2	1963.8727	0.0098	QRLMQCDLEEEENVR

**Mascot:** http://www.matrixscience.com/

Mascot Search Results

Peptide View

MS/MS Fragmentation of **GNLLQCICTGNGRGEWK**
Found in **P02751** in **uni_human_nr**, FINC_HUMAN Fibronectin OS=Homo sapiens GN=FN1 PE=1 SV=4

Match to Query 8657: 1962.903288 from(982.458920,2+) intensity(18874392.0000) rtinseconds(2200) scans(13137) index(10184)
Title: 130801_HPL_Human_Plaque_BR2_TR1_03_Spectrum033126_scans__13137_RTINSECONDS=2200
Data file E:\\RAW\\HumanPlaques\\mgf\\T\\1D\\T130801_HPL_Human_Plaque_BR2_TR1_03.mgf

Click mouse within plot area to zoom in by factor of two about that point
Or, Plot from 0 to 2000 Da Full range
Label all possible matches ○ Label matches used for scoring ⦿
Show Y-axis ☐

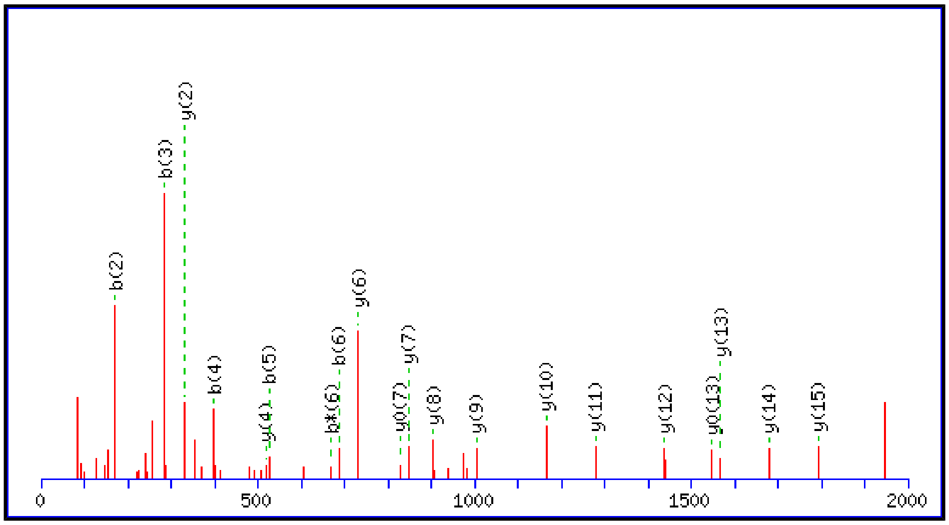

Monoisotopic mass of neutral peptide Mr(calc): 1962.9040
Fixed modifications: Carbamidomethyl (C) (apply to specified residues or termini only)
Variable modifications:
N11 : Deamidated (NQ)
Ions Score: 107 **Expect:** 1.1e-009
Matches : 20/170 fragment ions using 28 most intense peaks (help)

#	b	b^{++}	b*	b^{*++}	b^0	b^{0++}	Seq.	y	y^{++}	y*	y^{*++}	y^0	y^{0++}	#
1	58.0287	29.5180					G							17
2	**172.0717**	86.5395	155.0451	78.0262			N	1906.8898	953.9486	1889.8633	945.4353	1888.8793	944.9433	16
3	**285.1557**	143.0815	268.1292	134.5682			L	***1792.8469***	896.9271	1775.8204	888.4138	1774.8363	887.9218	15
4	**398.2398**	199.6235	381.2132	191.1103			L	***1679.7628***	840.3851	1662.7363	831.8718	1661.7523	831.3798	14
5	**526.2984**	263.6528	509.2718	255.1396			Q	***1566.6788***	783.8430	1549.6522	775.3298	1548.6682	774.8377	13
6	**686.3290**	343.6681	669.3025	335.1549			C	***1438.6202***	719.8137	1421.5936	711.3005	1420.6096	710.8085	12
7	799.4131	400.2102	782.3865	391.6969			I	***1278.5895***	639.7984	1261.5630	631.2851	1260.5790	630.7931	11
8	959.4437	480.2255	942.4172	471.7122			C	***1165.5055***	583.2564	1148.4789	574.7431	1147.4949	574.2511	10
9	1060.4914	530.7493	1043.4649	522.2361	1042.4808	521.7441	T	***1005.4748***	503.2411	988.4483	494.7278	987.4643	494.2358	9
10	1117.5129	559.2601	1100.4863	550.7468	1099.5023	550.2548	G	***904.4272***	452.7172	887.4006	444.2039	886.4166	443.7119	8
11	1232.5398	616.7735	1215.5133	608.2603	1214.5293	607.7683	N	***847.4057***	424.2065	830.3791	415.6932	829.3951	415.2012	7
12	1289.5613	645.2843	1272.5347	636.7710	1271.5507	636.2790	G	***732.3787***	366.6930	715.3522	358.1797	714.3682	357.6877	6
13	1445.6624	723.3348	1428.6358	714.8216	1427.6518	714.3296	R	675.3573	338.1823	658.3307	329.6690	657.3467	329.1770	5
14	1502.6839	751.8456	1485.6573	743.3323	1484.6733	742.8403	G	***519.2562***	260.1317	502.2296	251.6185	501.2456	251.1264	4
15	1631.7265	816.3669	1614.6999	807.8536	1613.7159	807.3616	E	462.2347	231.6210	445.2082	223.1077	444.2241	222.6157	3

**16**	1817.8058	909.4065	1800.7792	900.8932	1799.7952	900.4012	**W**	*333.1921*	167.0997	316.1656	158.5864			**2**
**17**							**K**	147.1128	74.0600	130.0863	65.5468			**1**

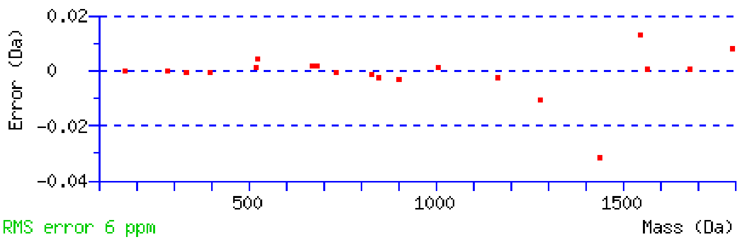

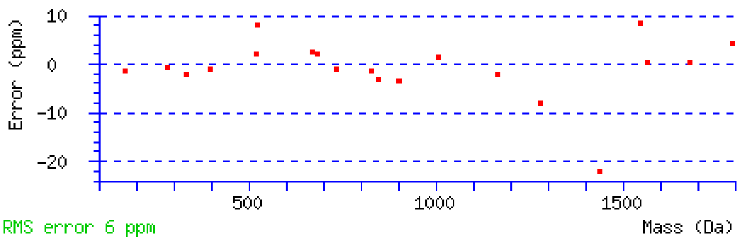

NCBI **BLAST** search of GNLLQCICTGNGRGEWK
(Parameters: blastp, nr protein database, expect=20000, no filter, PAM30)
Other BLAST web gateways

All matches to this query

Score	Mr(calc)	Delta	Sequence	Site Analysis
107.3	1962.9040	-0.0007	GNLLQCICTGNGRGEWK	Deamidated N11 100.00%
25.3	1962.9040	-0.0007	GNLLQCICTGNGRGEWK	Deamidated Q5 0.00%

Mascot: http://www.matrixscience.com/

Mascot Search Results

Peptide View

MS/MS Fragmentation of **GNLLQCICTGNGRGEWK**
Found in **P02751** in **uni_human_nr**, FINC_HUMAN Fibronectin OS=Homo sapiens GN=FN1 PE=1 SV=4

Match to Query 9066: 1962.903762 from(655.308530,3+) intensity(119695248.0000) rtinseconds(2190) scans(13640) index(10792)
Title: 130809_HPL_Human_Plaque_BR2_TR3_03_Spectrum034580_scans__13640_RTINSECONDS=2190
Data file E:\\RAW\\HumanPlaques\\mgf\\T\\1D\\T130809_HPL_Human_Plaque_BR2_TR3_03.mgf

Click mouse within plot area to zoom in by factor of two about that point
Or, Plot from 0 to 1700 Da Full range
Label all possible matches ○ Label matches used for scoring ⊙
Show Y-axis ☐

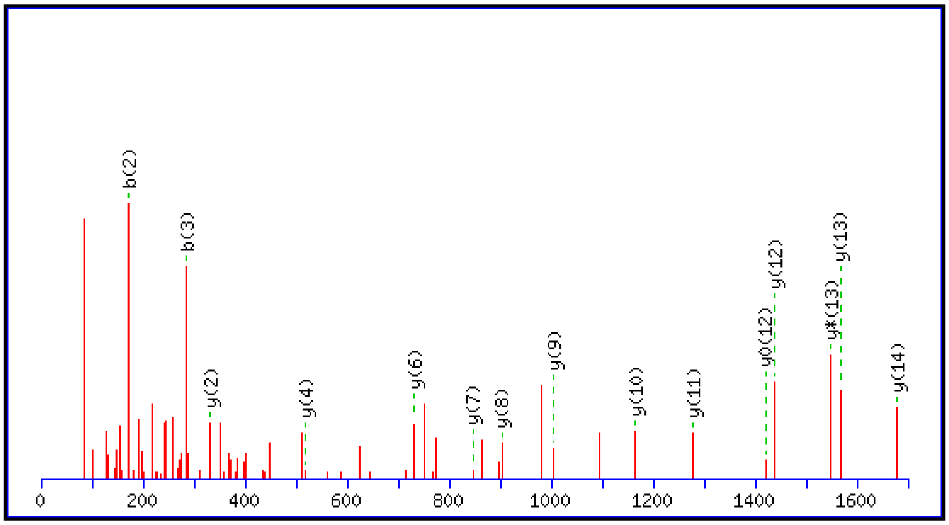

Monoisotopic mass of neutral peptide Mr(calc): 1962.9040
Fixed modifications: Carbamidomethyl (C) (apply to specified residues or termini only)
Variable modifications:
N11 : Deamidated (NQ)
Ions Score: 73 **Expect:** 2.5e-006
Matches : 15/170 fragment ions using 29 most intense peaks (help)

#	b	b++	b*	b*++	b^0	b^{0++}	Seq.	y	y++	y*	y*++	y^0	y^{0++}	#
1	58.0287	29.5180					G							17
2	172.0717	86.5395	155.0451	78.0262			N	1906.8898	953.9486	1889.8633	945.4353	1888.8793	944.9433	16
3	285.1557	143.0815	268.1292	134.5682			L	1792.8469	896.9271	1775.8204	888.4138	1774.8363	887.9218	15
4	398.2398	199.6235	381.2132	191.1103			L	1679.7628	840.3851	1662.7363	831.8718	1661.7523	831.3798	14
5	526.2984	263.6528	509.2718	255.1396			Q	1566.6788	783.8430	1549.6522	775.3298	1548.6682	774.8377	13
6	686.3290	343.6681	669.3025	335.1549			C	1438.6202	719.8137	1421.5936	711.3005	1420.6096	710.8085	12
7	799.4131	400.2102	782.3865	391.6969			I	1278.5895	639.7984	1261.5630	631.2851	1260.5790	630.7931	11
8	959.4437	480.2255	942.4172	471.7122			C	1165.5055	583.2564	1148.4789	574.7431	1147.4949	574.2511	10
9	1060.4914	530.7493	1043.4649	522.2361	1042.4808	521.7441	T	1005.4748	503.2411	988.4483	494.7278	987.4643	494.2358	9
10	1117.5129	559.2601	1100.4863	550.7468	1099.5023	550.2548	G	904.4272	452.7172	887.4006	444.2039	886.4166	443.7119	8
11	1232.5398	616.7735	1215.5133	608.2603	1214.5293	607.7683	N	847.4057	424.2065	830.3791	415.6932	829.3951	415.2012	7
12	1289.5613	645.2843	1272.5347	636.7710	1271.5507	636.2790	G	732.3787	366.6930	715.3522	358.1797	714.3682	357.6877	6
13	1445.6624	723.3348	1428.6358	714.8216	1427.6518	714.3296	R	675.3573	338.1823	658.3307	329.6690	657.3467	329.1770	5
14	1502.6839	751.8456	1485.6573	743.3323	1484.6733	742.8403	G	519.2562	260.1317	502.2296	251.6185	501.2456	251.1264	4
15	1631.7265	816.3669	1614.6999	807.8536	1613.7159	807.3616	E	462.2347	231.6210	445.2082	223.1077	444.2241	222.6157	3

**16**	1817.8058	909.4065	1800.7792	900.8932	1799.7952	900.4012	**W**	*333.1921*	167.0997	316.1656	158.5864			**2**
**17**							**K**	147.1128	74.0600	130.0863	65.5468			**1**

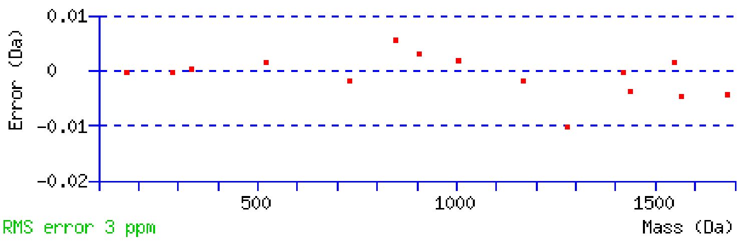

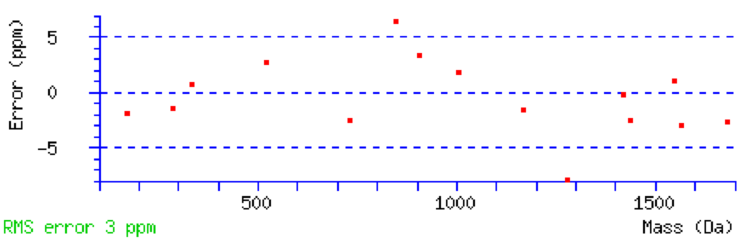

NCBI **BLAST** search of GNLLQCICTGNGRGEWK
(Parameters: blastp, nr protein database, expect=20000, no filter, PAM30)
Other BLAST web gateways

All matches to this query

Score	Mr(calc)	Delta	Sequence	Site Analysis
73.4	1962.9040	-0.0002	GNLLQCICTGNGRGEWK	Deamidated N11 100.00%
12.7	1962.9040	-0.0002	GNLLQCICTGNGRGEWK	Deamidated Q5 0.00%

Mascot: http://www.matrixscience.com/

Mascot Search Results

Peptide View

MS/MS Fragmentation of **GNLLQCICTGNGRGEWK**
Found in **P02751** in **uni_human_nr**, FINC_HUMAN Fibronectin OS=Homo sapiens GN=FN1 PE=1 SV=4

Match to Query 10052: 1961.916848 from(981.965700,2+) intensity(10108480.0000) rtinseconds(1882) scans(11550) index(8876)
Title: 130806_HPL_Human_Plaque_BR2_TR2_02_Spectrum033709_scans__11550_RTINSECONDS=1882
Data file E:\\RAW\\HumanPlaques\\mgf\\T\\1D\\T130806_HPL_Human_Plaque_BR2_TR2_02.mgf

Click mouse within plot area to zoom in by factor of two about that point
Or, Plot from 0 to 2000 Da Full range
Label all possible matches ○ Label matches used for scoring ⊙
Show Y-axis ☐

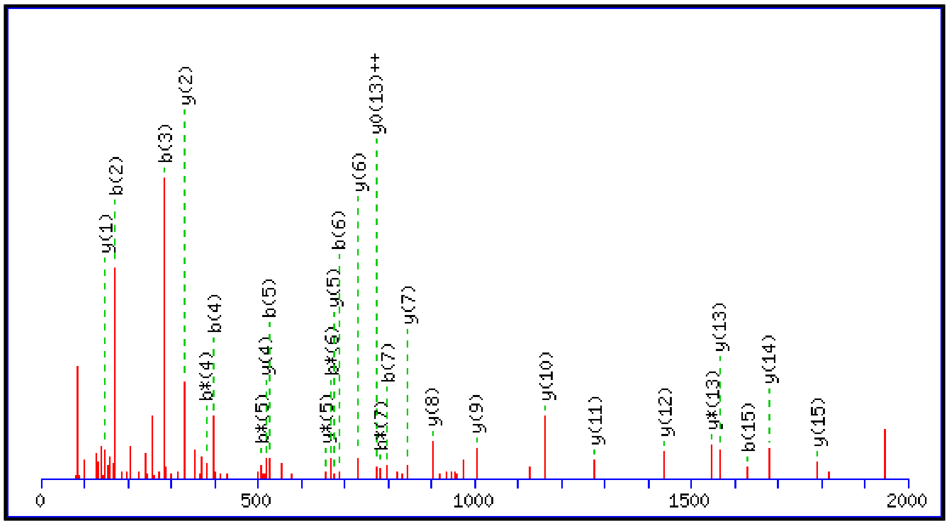

Monoisotopic mass of neutral peptide Mr(calc): 1961.9200
Fixed modifications: Carbamidomethyl (C) (apply to specified residues or termini only)
Ions Score: 112 **Expect:** 4e-010
Matches : 28/170 fragment ions using 48 most intense peaks (help)

#	b	b^{++}	b*	b*$^{++}$	b^{0}	b^{0++}	Seq.	y	y^{++}	y*	y*$^{++}$	y^{0}	y^{0++}	#
1	58.0287	29.5180					G							17
2	**172.0717**	86.5395	155.0451	78.0262			N	1905.9058	953.4565	1888.8793	944.9433	1887.8952	944.4513	16
3	**285.1557**	143.0815	268.1292	134.5682			L	***1791.8629***	896.4351	1774.8363	887.9218	1773.8523	887.4298	15
4	**398.2398**	199.6235	381.2132	191.1103			L	***1678.7788***	839.8930	1661.7523	831.3798	1660.7683	830.8878	14
5	**526.2984**	263.6528	509.2718	255.1396			Q	***1565.6948***	783.3510	1548.6682	774.8377	1547.6842	774.3457	13
6	**686.3290**	343.6681	669.3025	335.1549			C	***1437.6362***	719.3217	1420.6096	710.8085	1419.6256	710.3164	12
7	**799.4131**	400.2102	782.3865	391.6969			I	***1277.6055***	639.3064	1260.5790	630.7931	1259.5950	630.3011	11
8	959.4437	480.2255	942.4172	471.7122			C	***1164.5215***	582.7644	1147.4949	574.2511	1146.5109	573.7591	10
9	1060.4914	530.7493	1043.4649	522.2361	1042.4808	521.7441	T	***1004.4908***	502.7490	987.4643	494.2358	986.4803	493.7438	9
10	1117.5129	559.2601	1100.4863	550.7468	1099.5023	550.2548	G	***903.4431***	452.2252	886.4166	443.7119	885.4326	443.2199	8
11	1231.5558	616.2815	1214.5293	607.7683	1213.5452	607.2763	N	***846.4217***	423.7145	829.3951	415.2012	828.4111	414.7092	7
12	1288.5773	644.7923	1271.5507	636.2790	1270.5667	635.7870	G	***732.3787***	366.6930	715.3522	358.1797	714.3682	357.6877	6
13	1444.6784	722.8428	1427.6518	714.3296	1426.6678	713.8375	R	***675.3573***	338.1823	658.3307	329.6690	657.3467	329.1770	5
14	1501.6998	751.3536	1484.6733	742.8403	1483.6893	742.3483	G	***519.2562***	260.1317	502.2296	251.6185	501.2456	251.1264	4
15	**1630.7424**	815.8749	1613.7159	807.3616	1612.7319	806.8696	E	462.2347	231.6210	445.2082	223.1077	444.2241	222.6157	3
16	1816.8217	908.9145	1799.7952	900.4012	1798.8112	899.9092	W	***333.1921***	167.0997	316.1656	158.5864			2

17							K	*147.1128*	74.0600	130.0863	65.5468			1

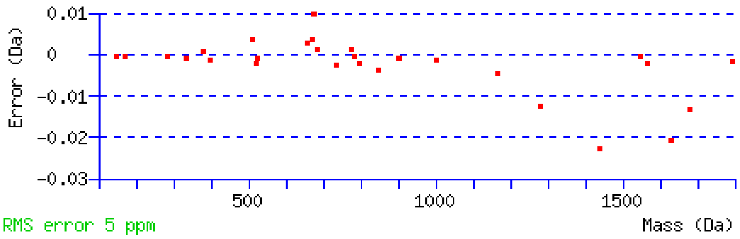

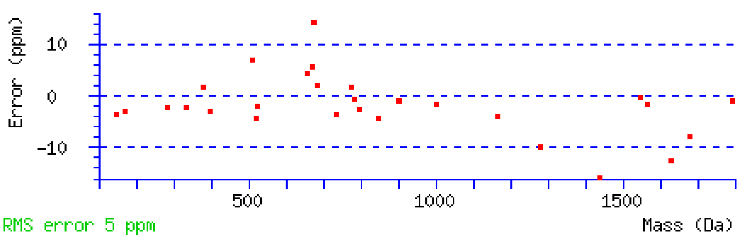

NCBI **BLAST** search of GNLLQCICTGNGRGEWK
(Parameters: blastp, nr protein database, expect=20000, no filter, PAM30)
Other BLAST web gateways

All matches to this query

Score	Mr(calc)	Delta	Sequence
112.3	1961.9200	-0.0032	GNLLQCICTGNGRGEWK
1.1	1961.9178	-0.0009	NGLKQDEPGQAGSQKSSTK

Mascot: http://www.matrixscience.com/

Mascot Search Results

Peptide View

MS/MS Fragmentation of **GNLLQCICTGNGRGEWK**
Found in **P02751** in **uni_human_nr**, FINC_HUMAN Fibronectin OS=Homo sapiens GN=FN1 PE=1 SV=4

Match to Query 10084: 1961.916462 from(654.979430,3+) intensity(160983184.0000) rtinseconds(1856) scans(11481) index(8796)
Title: 130809_HPL_Human_Plaque_BR2_TR3_02_Spectrum033649_scans__11481_RTINSECONDS=1856
Data file E:\\RAW\\HumanPlaques\\mgf\\T\\1D\\T130809_HPL_Human_Plaque_BR2_TR3_02.mgf

Click mouse within plot area to zoom in by factor of two about that point
Or, [Plot from] [0] to [1800] Da [Full range]
Label all possible matches ○ Label matches used for scoring ⦿
Show Y-axis ☐

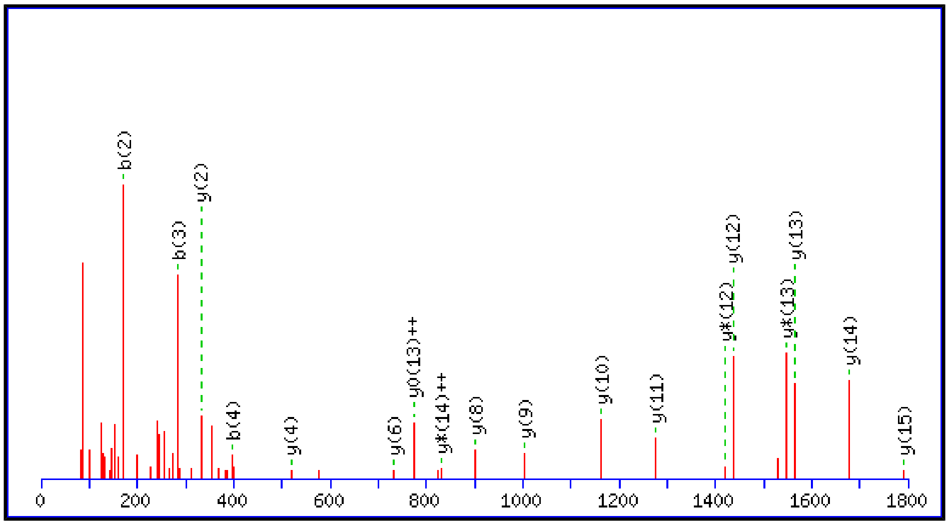

Monoisotopic mass of neutral peptide Mr(calc): 1961.9200
Fixed modifications: Carbamidomethyl (C) (apply to specified residues or termini only)
Ions Score: 93 **Expect:** 3.3e-008
Matches : 18/170 fragment ions using 25 most intense peaks (help)

#	b	b^{++}	b*	b^{*++}	b^0	b^{0++}	Seq.	y	y^{++}	y*	y^{*++}	y^0	y^{0++}	#
1	58.0287	29.5180					G							17
2	**172.0717**	86.5395	155.0451	78.0262			N	1905.9058	953.4565	1888.8793	944.9433	1887.8952	944.4513	16
3	**285.1557**	143.0815	268.1292	134.5682			L	***1791.8629***	896.4351	1774.8363	887.9218	1773.8523	887.4298	15
4	**398.2398**	199.6235	381.2132	191.1103			L	***1678.7788***	839.8930	1661.7523	831.3798	1660.7683	830.8878	14
5	526.2984	263.6528	509.2718	255.1396			Q	***1565.6948***	783.3510	1548.6682	774.8377	1547.6842	774.3457	13
6	686.3290	343.6681	669.3025	335.1549			C	***1437.6362***	719.3217	1420.6096	710.8085	1419.6256	710.3164	12
7	799.4131	400.2102	782.3865	391.6969			I	***1277.6055***	639.3064	1260.5790	630.7931	1259.5950	630.3011	11
8	959.4437	480.2255	942.4172	471.7122			C	***1164.5215***	582.7644	1147.4949	574.2511	1146.5109	573.7591	10
9	1060.4914	530.7493	1043.4649	522.2361	1042.4808	521.7441	T	***1004.4908***	502.7490	987.4643	494.2358	986.4803	493.7438	9
10	1117.5129	559.2601	1100.4863	550.7468	1099.5023	550.2548	G	***903.4431***	452.2252	886.4166	443.7119	885.4326	443.2199	8
11	1231.5558	616.2815	1214.5293	607.7683	1213.5452	607.2763	N	846.4217	423.7145	829.3951	415.2012	828.4111	414.7092	7
12	1288.5773	644.7923	1271.5507	636.2790	1270.5667	635.7870	G	***732.3787***	366.6930	715.3522	358.1797	714.3682	357.6877	6
13	1444.6784	722.8428	1427.6518	714.3296	1426.6678	713.8375	R	675.3573	338.1823	658.3307	329.6690	657.3467	329.1770	5
14	1501.6998	751.3536	1484.6733	742.8403	1483.6893	742.3483	G	***519.2562***	260.1317	502.2296	251.6185	501.2456	251.1264	4
15	1630.7424	815.8749	1613.7159	807.3616	1612.7319	806.8696	E	462.2347	231.6210	445.2082	223.1077	444.2241	222.6157	3
16	1816.8217	908.9145	1799.7952	900.4012	1798.8112	899.9092	W	***333.1921***	167.0997	316.1656	158.5864			2

17							K	147.1128	74.0600	130.0863	65.5468			1

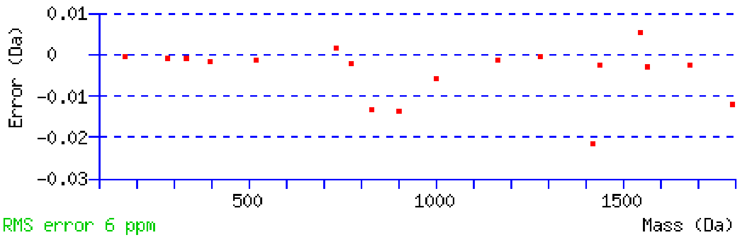

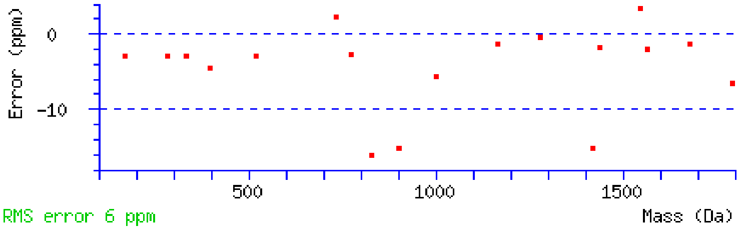

NCBI **BLAST** search of GNLLQCICTGNGRGEWK
(Parameters: blastp, nr protein database, expect=20000, no filter, PAM30)
Other BLAST web gateways

All matches to this query

Score	Mr(calc)	Delta	Sequence
93.0	1961.9200	-0.0035	GNLLQCICTGNGRGEWK
1.2	1961.9178	-0.0013	NGLKQDEPGQAGSQKSSTK

Mascot: http://www.matrixscience.com/

Mascot Search Results

Peptide View

MS/MS Fragmentation of **GNLLQCICTGNGRGEWKCER**
Found in **P02751** in **uni_human_nr**, FINC_HUMAN Fibronectin OS=Homo sapiens GN=FN1 PE=1 SV=4

Match to Query 13072: 2408.078336 from(603.026860,4+) intensity(2229472.2500) rtinseconds(1968) scans(11501) index(8733)
Title: 130801_HPL_Human_Plaque_BR1_TR1_03_Spectrum031983_scans__11501_RTINSECONDS=1968
Data file E:\\RAW\\HumanPlaques\\mgf\\T\\1D\\T130801_HPL_Human_Plaque_BR1_TR1_03.mgf

Click mouse within plot area to zoom in by factor of two about that point
Or, Plot from 0 to 2200 Da Full range
Label all possible matches ○ Label matches used for scoring ⦿
Show Y-axis ☐

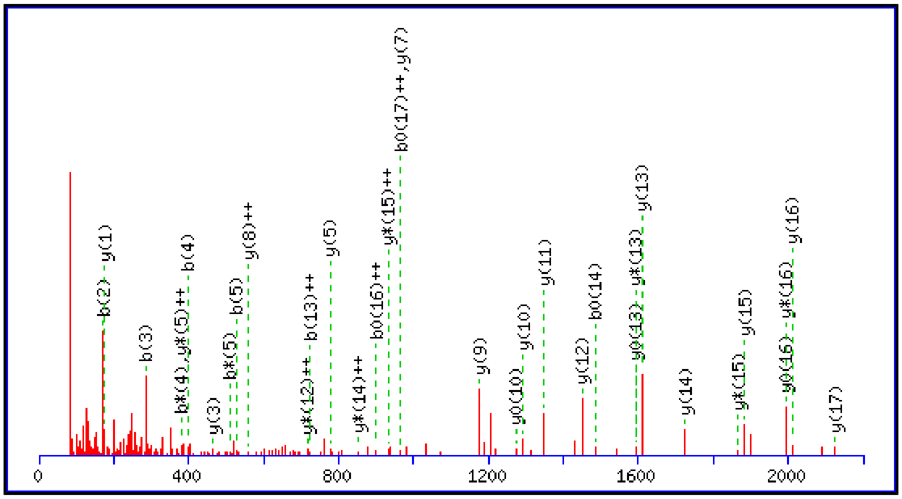

Monoisotopic mass of neutral peptide Mr(calc): 2408.0784
Fixed modifications: Carbamidomethyl (C) (apply to specified residues or termini only)
Variable modifications:
N11 : Deamidated (NQ)
Ions Score: 55 **Expect:** 0.00015
Matches : 34/208 fragment ions using 88 most intense peaks (help)

#	b	b^{++}	b^{*}	b^{*++}	b^{0}	b^{0++}	Seq.	y	y^{++}	y^{*}	y^{*++}	y^{0}	y^{0++}	#
1	58.0287	29.5180					G							20
2	**172.0717**	86.5395	155.0451	78.0262			N	2352.0642	1176.5357	2335.0376	1168.0225	2334.0536	1167.5304	19
3	**285.1557**	143.0815	268.1292	134.5682			L	2238.0213	1119.5143	2220.9947	1111.0010	2220.0107	1110.5090	18
4	**398.2398**	199.6235	381.2132	191.1103			L	***2124.9372***	1062.9722	2107.9106	1054.4590	2106.9266	1053.9670	17
5	**526.2984**	263.6528	509.2718	255.1396			Q	***2011.8531***	1006.4302	1994.8266	997.9169	1993.8426	997.4249	16
6	686.3290	343.6681	669.3025	335.1549			C	***1883.7946***	942.4009	1866.7680	933.8876	1865.7840	933.3956	15
7	799.4131	400.2102	782.3865	391.6969			I	***1723.7639***	862.3856	1706.7374	853.8723	1705.7533	853.3803	14
8	959.4437	480.2255	942.4172	471.7122			C	***1610.6798***	805.8436	1593.6533	797.3303	1592.6693	796.8383	13
9	1060.4914	530.7493	1043.4649	522.2361	1042.4808	521.7441	T	***1450.6492***	725.8282	1433.6226	717.3150	1432.6386	716.8229	12
10	1117.5129	559.2601	1100.4863	550.7468	1099.5023	550.2548	G	***1349.6015***	675.3044	1332.5750	666.7911	1331.5909	666.2991	11
11	1232.5398	616.7735	1215.5133	608.2603	1214.5293	607.7683	N	***1292.5800***	646.7937	1275.5535	638.2804	1274.5695	637.7884	10
12	1289.5613	645.2843	1272.5347	636.7710	1271.5507	636.2790	G	***1177.5531***	589.2802	1160.5266	580.7669	1159.5425	580.2749	9
13	1445.6624	723.3348	1428.6358	714.8216	1427.6518	714.3296	R	1120.5316	560.7695	1103.5051	552.2562	1102.5211	551.7642	8
14	1502.6839	751.8456	1485.6573	743.3323	1484.6733	742.8403	G	***964.4305***	482.7189	947.4040	474.2056	946.4200	473.7136	7
15	1631.7265	816.3669	1614.6999	807.8536	1613.7159	807.3616	E	907.4091	454.2082	890.3825	445.6949	889.3985	445.2029	6
16	1817.8058	909.4065	1800.7792	900.8932	1799.7952	900.4012	W	***778.3665***	389.6869	761.3399	381.1736	760.3559	380.6816	5
17	1945.9007	973.4540	1928.8742	964.9407	1927.8902	964.4487	K	592.2872	296.6472	575.2606	288.1339	574.2766	287.6419	4
18	2105.9314	1053.4693	2088.9048	1044.9561	2087.9208	1044.4640	C	***464.1922***	232.5997	447.1656	224.0865	446.1816	223.5945	3

**19**	2234.9740	1117.9906	2217.9474	1109.4773	2216.9634	1108.9853	**E**	304.1615	152.5844	287.1350	144.0711	286.1510	143.5791	**2**
**20**							**R**	***175.1190***	88.0631	158.0924	79.5498			**1**

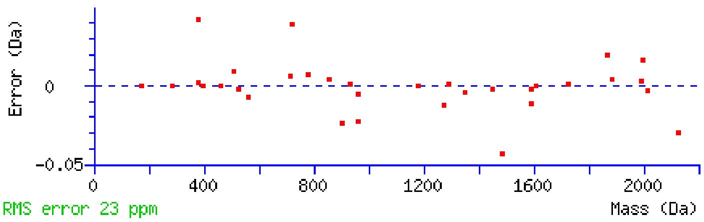

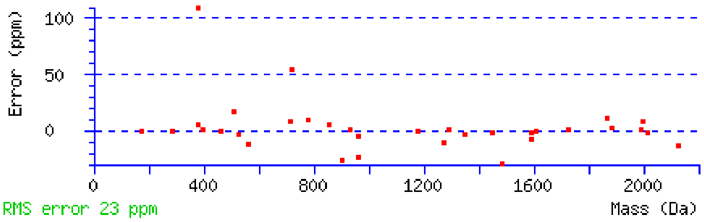

NCBI **BLAST** search of GNLLQCICTGNGRGEWKCER
(Parameters: blastp, nr protein database, expect=20000, no filter, PAM30)
Other BLAST web gateways

All matches to this query

Score	Mr(calc)	Delta	Sequence	Site Analysis
54.8	2408.0784	-0.0000	GNLLQCICTGNGRGEWKCER	Deamidated N11 100.00%
5.3	2408.0784	-0.0000	GNLLQCICTGNGRGEWKCER	Deamidated Q5 0.00%

**Mascot:** http://www.matrixscience.com/

Mascot Search Results

Peptide View

MS/MS Fragmentation of **GNLLQCICTGNGRGEWKCER**
Found in **P02751** in **uni_human_nr**, FINC_HUMAN Fibronectin OS=Homo sapiens GN=FN1 PE=1 SV=4

Match to Query 13496: 2407.083402 from(803.368410,3+) intensity(2074839.3750) rtinseconds(1655) scans(9304) index(6778)
Title: 130801_HPL_Human_Plaque_BR1_TR1_02_Spectrum030785_scans__9304_RTINSECONDS=1655
Data file E:\\RAW\\HumanPlaques\\mgf\\T\\1D\\T130801_HPL_Human_Plaque_BR1_TR1_02.mgf

Click mouse within plot area to zoom in by factor of two about that point
Or, [Plot from] 0 to 2400 Da [Full range]
Label all possible matches ○ Label matches used for scoring ⦿
Show Y-axis ☐

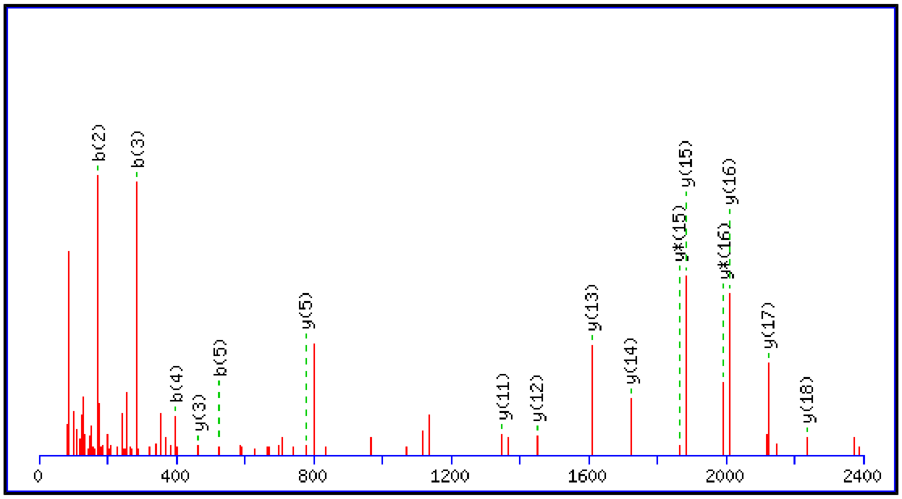

Monoisotopic mass of neutral peptide Mr(calc): 2407.0943
Fixed modifications: Carbamidomethyl (C) (apply to specified residues or termini only)
Ions Score: 53 **Expect:** 0.00029
Matches : 16/208 fragment ions using 33 most intense peaks (help)

#	b	b++	b*	b*++	b0	b0++	Seq.	y	y++	y*	y*++	y0	y0++	#
1	58.0287	29.5180					G							20
2	**172.0717**	86.5395	155.0451	78.0262			N	2351.0802	1176.0437	2334.0536	1167.5304	2333.0696	1167.0384	19
3	**285.1557**	143.0815	268.1292	134.5682			L	***2237.0372***	1119.0223	2220.0107	1110.5090	2219.0267	1110.0170	18
4	**398.2398**	199.6235	381.2132	191.1103			L	***2123.9532***	1062.4802	2106.9266	1053.9670	2105.9426	1053.4749	17
5	**526.2984**	263.6528	509.2718	255.1396			Q	***2010.8691***	1005.9382	1993.8426	997.4249	1992.8585	996.9329	16
6	686.3290	343.6681	669.3025	335.1549			C	***1882.8105***	941.9089	1865.7840	933.3956	1864.8000	932.9036	15
7	799.4131	400.2102	782.3865	391.6969			I	***1722.7799***	861.8936	1705.7533	853.3803	1704.7693	852.8883	14
8	959.4437	480.2255	942.4172	471.7122			C	***1609.6958***	805.3515	1592.6693	796.8383	1591.6853	796.3463	13
9	1060.4914	530.7493	1043.4649	522.2361	1042.4808	521.7441	T	***1449.6652***	725.3362	1432.6386	716.8229	1431.6546	716.3309	12
10	1117.5129	559.2601	1100.4863	550.7468	1099.5023	550.2548	G	***1348.6175***	674.8124	1331.5909	666.2991	1330.6069	665.8071	11
11	1231.5558	616.2815	1214.5293	607.7683	1213.5452	607.2763	N	1291.5960	646.3017	1274.5695	637.7884	1273.5855	637.2964	10
12	1288.5773	644.7923	1271.5507	636.2790	1270.5667	635.7870	G	1177.5531	589.2802	1160.5266	580.7669	1159.5425	580.2749	9
13	1444.6784	722.8428	1427.6518	714.3296	1426.6678	713.8375	R	1120.5316	560.7695	1103.5051	552.2562	1102.5211	551.7642	8
14	1501.6998	751.3536	1484.6733	742.8403	1483.6893	742.3483	G	964.4305	482.7189	947.4040	474.2056	946.4200	473.7136	7
15	1630.7424	815.8749	1613.7159	807.3616	1612.7319	806.8696	E	907.4091	454.2082	890.3825	445.6949	889.3985	445.2029	6
16	1816.8217	908.9145	1799.7952	900.4012	1798.8112	899.9092	W	***778.3665***	389.6869	761.3399	381.1736	760.3559	380.6816	5
17	1944.9167	972.9620	1927.8902	964.4487	1926.9061	963.9567	K	592.2872	296.6472	575.2606	288.1339	574.2766	287.6419	4
18	2104.9474	1052.9773	2087.9208	1044.4640	2086.9368	1043.9720	C	***464.1922***	232.5997	447.1656	224.0865	446.1816	223.5945	3
19	2233.9900	1117.4986	2216.9634	1108.9853	2215.9794	1108.4933	E	304.1615	152.5844	287.1350	144.0711	286.1510	143.5791	2

**20**							**R**	175.1190	88.0631	158.0924	79.5498		**1**

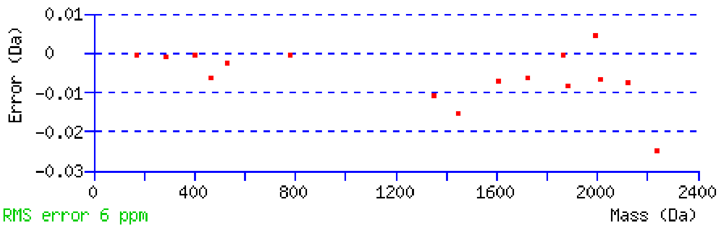

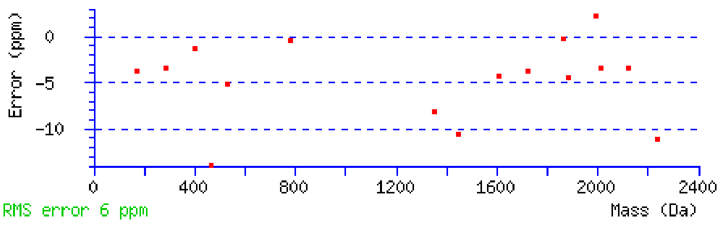

NCBI **BLAST** search of GNLLQCICTGNGRGEWKCER
(Parameters: blastp, nr protein database, expect=20000, no filter, PAM30)
Other BLAST web gateways

All matches to this query

Score	Mr(calc)	Delta	Sequence
52.6	2407.0943	-0.0109	GNLLQCICTGNGRGEWKCER

Mascot: http://www.matrixscience.com/

Mascot Search Results

Peptide View

MS/MS Fragmentation of **NSITLTNLTPGTEYVVSIVALNGR**
Found in **P02751** in **uni_human_nr**, FINC_HUMAN Fibronectin OS=Homo sapiens GN=FN1 PE=1 SV=4

Match to Query 11823: 2533.325832 from(845.449220,3+) intensity(82577.0000) rtinseconds(3909) scans(24109) index(18617)
Title: 130809_HPL_Human_Plaque_BR1_TR3_09_Spectrum040406_scans__24109_RTINSECONDS=3909
Data file E:\\RAW\\HumanPlaques\\mgf\\T\\1D\\T130809_HPL_Human_Plaque_BR1_TR3_09.mgf

Click mouse within plot area to zoom in by factor of two about that point
Or, [Plot from] [0] to [2200] Da [Full range]
Label all possible matches ○ Label matches used for scoring ⊙
Show Y-axis ☐

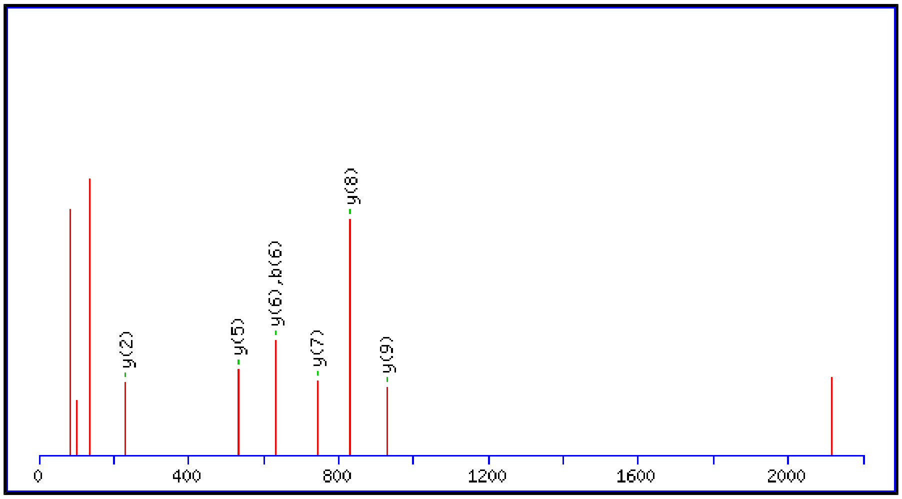

Monoisotopic mass of neutral peptide Mr(calc): 2533.3275
Fixed modifications: Carbamidomethyl (C) (apply to specified residues or termini only)
Variable modifications:
N7 : Deamidated (NQ)
N22 : Deamidated (NQ)
Ions Score: 41 **Expect:** 0.0052
Matches : 7/260 fragment ions using 8 most intense peaks (help)

#	b	b^{++}	b*	b^{*++}	b^0	b^{0++}	Seq.	y	y^{++}	y*	y^{*++}	y^0	y^{0++}	#
1	115.0502	58.0287	98.0237	49.5155			N							24
2	202.0822	101.5448	185.0557	93.0315	184.0717	92.5395	S	2420.2919	1210.6496	2403.2654	1202.1363	2402.2813	1201.6443	23
3	315.1663	158.0868	298.1397	149.5735	297.1557	149.0815	I	2333.2599	1167.1336	2316.2333	1158.6203	2315.2493	1158.1283	22
4	416.2140	208.6106	399.1874	200.0974	398.2034	199.6053	T	2220.1758	1110.5915	2203.1493	1102.0783	2202.1652	1101.5863	21
5	529.2980	265.1527	512.2715	256.6394	511.2875	256.1474	L	2119.1281	1060.0677	2102.1016	1051.5544	2101.1176	1051.0624	20
6	**630.3457**	315.6765	613.3192	307.1632	612.3352	306.6712	T	2006.0441	1003.5257	1989.0175	995.0124	1988.0335	994.5204	19
7	745.3727	373.1900	728.3461	364.6767	727.3621	364.1847	N	1904.9964	953.0018	1887.9698	944.4886	1886.9858	943.9965	18
8	858.4567	429.7320	841.4302	421.2187	840.4462	420.7267	L	1789.9694	895.4884	1772.9429	886.9751	1771.9589	886.4831	17
9	959.5044	480.2558	942.4779	471.7426	941.4938	471.2506	T	1676.8854	838.9463	1659.8588	830.4331	1658.8748	829.9410	16
10	1056.5572	528.7822	1039.5306	520.2689	1038.5466	519.7769	P	1575.8377	788.4225	1558.8112	779.9092	1557.8271	779.4172	15
11	1113.5786	557.2930	1096.5521	548.7797	1095.5681	548.2877	G	1478.7849	739.8961	1461.7584	731.3828	1460.7744	730.8908	14
12	1214.6263	607.8168	1197.5998	599.3035	1196.6157	598.8115	T	1421.7635	711.3854	1404.7369	702.8721	1403.7529	702.3801	13
13	1343.6689	672.3381	1326.6424	663.8248	1325.6583	663.3328	E	1320.7158	660.8615	1303.6892	652.3483	1302.7052	651.8563	12
14	1506.7322	753.8698	1489.7057	745.3565	1488.7217	744.8645	Y	1191.6732	596.3402	1174.6467	587.8270	1173.6626	587.3350	11
15	1605.8006	803.4040	1588.7741	794.8907	1587.7901	794.3987	V	1028.6099	514.8086	1011.5833	506.2953	1010.5993	505.8033	10
16	1704.8691	852.9382	1687.8425	844.4249	1686.8585	843.9329	V	***929.5415***	465.2744	912.5149	456.7611	911.5309	456.2691	9
17	1791.9011	896.4542	1774.8745	887.9409	1773.8905	887.4489	S	***830.4730***	415.7402	813.4465	407.2269	812.4625	406.7349	8

**18**	1904.9852	952.9962	1887.9586	944.4829	1886.9746	943.9909	**I**	***743.4410***	372.2241	726.4145	363.7109			**7**
**19**	2004.0536	1002.5304	1987.0270	994.0171	1986.0430	993.5251	**V**	***630.3570***	315.6821	613.3304	307.1688			**6**
**20**	2075.0907	1038.0490	2058.0641	1029.5357	2057.0801	1029.0437	**A**	***531.2885***	266.1479	514.2620	257.6346			**5**
**21**	2188.1747	1094.5910	2171.1482	1086.0777	2170.1642	1085.5857	**L**	460.2514	230.6293	443.2249	222.1161			**4**
**22**	2303.2017	1152.1045	2286.1751	1143.5912	2285.1911	1143.0992	**N**	347.1674	174.0873	330.1408	165.5740			**3**
**23**	2360.2232	1180.6152	2343.1966	1172.1019	2342.2126	1171.6099	**G**	***232.1404***	116.5738	215.1139	108.0606			**2**
**24**							**R**	175.1190	88.0631	158.0924	79.5498			**1**

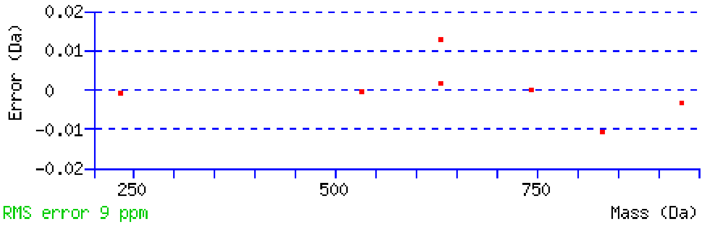

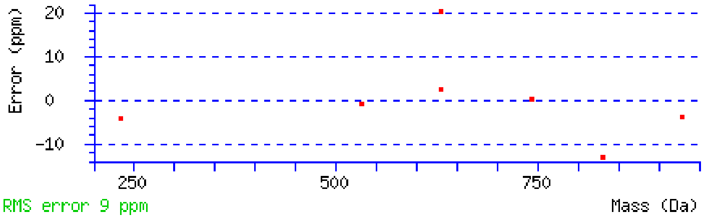

NCBI **BLAST** search of NSITLTNLTPGTEYVVSIVALNGR
(Parameters: blastp, nr protein database, expect=20000, no filter, PAM30)
Other BLAST web gateways

All matches to this query

Score	Mr(calc)	Delta	Sequence	Site Analysis
41.3	2533.3275	-0.0017	NSITLTNLTPGTEYVVSIVALNGR	Deamidated N7, N22 50.00%
41.3	2533.3275	-0.0017	NSITLTNLTPGTEYVVSIVALNGR	Deamidated N1, N22 50.00%
6.5	2533.3210	0.0048	VIKPTSSNTAQGRFDTSILPICK	
6.5	2533.3210	0.0048	VIKPTSSNTAQGRFDTSILPICK	
0.7	2533.3297	-0.0039	HCLTSIYRPFVDKALKQMGLR	
0.1	2532.3114	1.0145	DTILVCLDCCIKIVNLQGRLK	
0.1	2533.3362	-0.0104	LALRLYTCQEWAVVKVQAQVR	

Mascot: http://www.matrixscience.com/

Mascot Search Results

Peptide View

MS/MS Fragmentation of **NSITLTNLTPGTEYVVSIVALNGR**
Found in **P02751** in **uni_human_nr**, FINC_HUMAN Fibronectin OS=Homo sapiens GN=FN1 PE=1 SV=4

Match to Query 14847: 2532.342868 from(1267.178710,2+) intensity(3762859.2500) rtinseconds(3509) scans(24236) index(20118)
Title: 130809_HPL_Human_Plaque_BR1_TR3_02_Spectrum044644_scans__24236_RTINSECONDS=3509
Data file E:\\RAW\\HumanPlaques\\mgf\\T\\1D\\T130809_HPL_Human_Plaque_BR1_TR3_02.mgf

Click mouse within plot area to zoom in by factor of two about that point
Or, Plot from 0 to 1700 Da Full range
Label all possible matches Label matches used for scoring
Show Y-axis

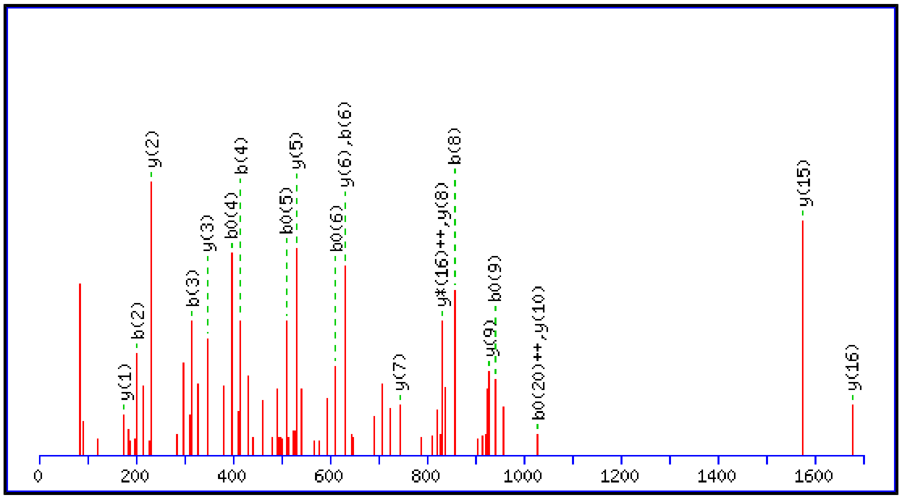

Monoisotopic mass of neutral peptide Mr(calc): 2532.3435
Fixed modifications: Carbamidomethyl (C) (apply to specified residues or termini only)
Variable modifications:
N22 : Deamidated (NQ)
Ions Score: 99 **Expect:** 7.7e-009
Matches : 22/260 fragment ions using 21 most intense peaks (help)

#	b	b++	b*	b*++	b0	b0++	Seq.	y	y++	y*	y*++	y0	y0++	#
1	115.0502	58.0287	98.0237	49.5155			N							24
2	**202.0822**	101.5448	185.0557	93.0315	184.0717	92.5395	S	2419.3079	1210.1576	2402.2813	1201.6443	2401.2973	1201.1523	23
3	**315.1663**	158.0868	298.1397	149.5735	297.1557	149.0815	I	2332.2759	1166.6416	2315.2493	1158.1283	2314.2653	1157.6363	22
4	**416.2140**	208.6106	399.1874	200.0974	398.2034	199.6053	T	2219.1918	1110.0995	2202.1652	1101.5863	2201.1812	1101.0943	21
5	529.2980	265.1527	512.2715	256.6394	511.2875	256.1474	L	2118.1441	1059.5757	2101.1176	1051.0624	2100.1335	1050.5704	20
6	**630.3457**	315.6765	613.3192	307.1632	612.3352	306.6712	T	2005.0600	1003.0337	1988.0335	994.5204	1987.0495	994.0284	19
7	744.3886	372.6980	727.3621	364.1847	726.3781	363.6927	N	1904.0124	952.5098	1886.9858	943.9965	1886.0018	943.5045	18
8	**857.4727**	429.2400	840.4462	420.7267	839.4621	420.2347	L	1789.9694	895.4884	1772.9429	886.9751	1771.9589	886.4831	17
9	958.5204	479.7638	941.4938	471.2506	940.5098	470.7585	T	***1676.8854***	838.9463	1659.8588	830.4331	1658.8748	829.9410	16
10	1055.5732	528.2902	1038.5466	519.7769	1037.5626	519.2849	P	***1575.8377***	788.4225	1558.8112	779.9092	1557.8271	779.4172	15
11	1112.5946	556.8009	1095.5681	548.2877	1094.5840	547.7957	G	1478.7849	739.8961	1461.7584	731.3828	1460.7744	730.8908	14
12	1213.6423	607.3248	1196.6157	598.8115	1195.6317	598.3195	T	1421.7635	711.3854	1404.7369	702.8721	1403.7529	702.3801	13
13	1342.6849	671.8461	1325.6583	663.3328	1324.6743	662.8408	E	1320.7158	660.8615	1303.6892	652.3483	1302.7052	651.8563	12
14	1505.7482	753.3777	1488.7217	744.8645	1487.7377	744.3725	Y	1191.6732	596.3402	1174.6467	587.8270	1173.6626	587.3350	11
15	1604.8166	802.9120	1587.7901	794.3987	1586.8061	793.9067	V	***1028.6099***	514.8086	1011.5833	506.2953	1010.5993	505.8033	10
16	1703.8850	852.4462	1686.8585	843.9329	1685.8745	843.4409	V	***929.5415***	465.2744	912.5149	456.7611	911.5309	456.2691	9
17	1790.9171	895.9622	1773.8905	887.4489	1772.9065	886.9569	S	***830.4730***	415.7402	813.4465	407.2269	812.4625	406.7349	8
18	1904.0011	952.5042	1886.9746	943.9909	1885.9906	943.4989	I	***743.4410***	372.2241	726.4145	363.7109			7

**19**	2003.0696	1002.0384	1986.0430	993.5251	1985.0590	993.0331	**V**	***630.3570***	315.6821	613.3304	307.1688			**6**
**20**	2074.1067	1037.5570	2057.0801	1029.0437	2056.0961	1028.5517	**A**	***531.2885***	266.1479	514.2620	257.6346			**5**
**21**	2187.1907	1094.0990	2170.1642	1085.5857	2169.1802	1085.0937	**L**	460.2514	230.6293	443.2249	222.1161			**4**
**22**	2302.2177	1151.6125	2285.1911	1143.0992	2284.2071	1142.6072	**N**	***347.1674***	174.0873	330.1408	165.5740			**3**
**23**	2359.2391	1180.1232	2342.2126	1171.6099	2341.2286	1171.1179	**G**	***232.1404***	116.5738	215.1139	108.0606			**2**
**24**							**R**	***175.1190***	88.0631	158.0924	79.5498			**1**

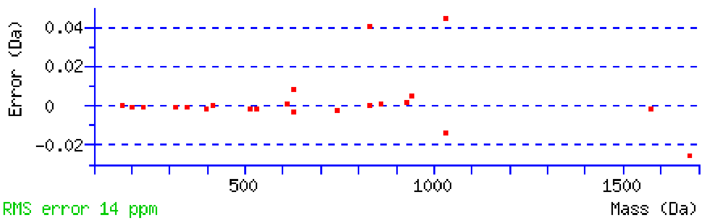

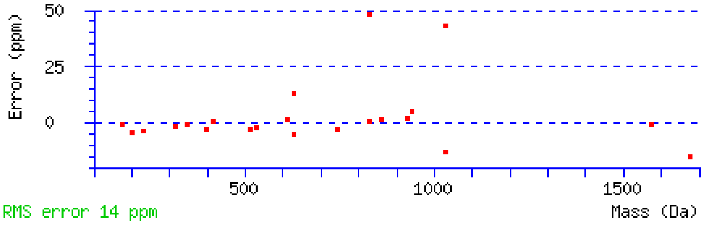

NCBI **BLAST** search of NSITLTNLTPGTEYVVSIVALNGR
(Parameters: blastp, nr protein database, expect=20000, no filter, PAM30)
Other BLAST web gateways

All matches to this query

Score	Mr(calc)	Delta	Sequence	Site Analysis
98.7	2532.3435	-0.0007	NSITLTNLTPGTEYVVSIVALNGR	Deamidated N22 100.00%
10.0	2532.3435	-0.0007	NSITLTNLTPGTEYVVSIVALNGR	Deamidated N7 0.00%
3.3	2531.3344	1.0085	SLLGDSAPTLHLNKGTPSQSPVVGR	
0.7	2532.3408	0.0021	ASRPAALGGPARAEQAATATAPADKAL	
0.6	2532.3522	-0.0094	LALRLYTCQEWAVVKVQAQVR	
0.2	2532.3509	-0.0080	LEKFSSALQPGTLLDLLQVEGMK	

Mascot: http://www.matrixscience.com/

Mascot Search Results

Peptide View

MS/MS Fragmentation of **NSITLTNLTPGTEYVVSIVALNGR**
Found in **P02751** in **uni_human_nr**, FINC_HUMAN Fibronectin OS=Homo sapiens GN=FN1 PE=1 SV=4

Match to Query 12758: 2532.342372 from(845.121400,3+) intensity(1041584.8750) rtinseconds(3517) scans(22032) index(17489)
Title: 130809_HPL_Human_Plaque_BR1_TR3_06_Spectrum039843_scans__22032_RTINSECONDS=3517
Data file E:\\RAW\\HumanPlaques\\mgf\\T\\1D\\T130809_HPL_Human_Plaque_BR1_TR3_06.mgf

Click mouse within plot area to zoom in by factor of two about that point
Or, Plot from 0 to 1600 Da Full range
Label all possible matches ○ Label matches used for scoring ⊙
Show Y-axis ☐

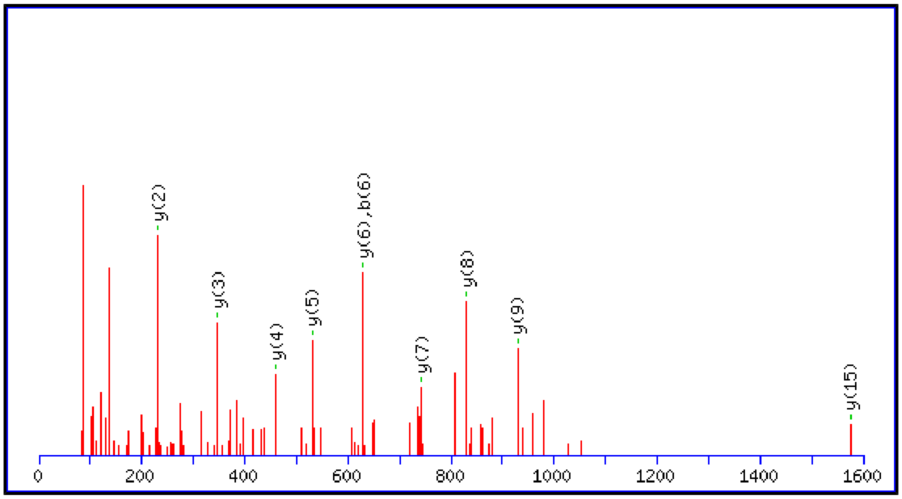

Monoisotopic mass of neutral peptide Mr(calc): 2532.3435
Fixed modifications: Carbamidomethyl (C) (apply to specified residues or termini only)
Variable modifications:
N22 : Deamidated (NQ)
Ions Score: 72 **Expect:** 3.9e-006
Matches : 10/260 fragment ions using 11 most intense peaks (help)

#	b	b^{++}	b^{*}	b^{*++}	b^{0}	b^{0++}	Seq.	y	y^{++}	y^{*}	y^{*++}	y^{0}	y^{0++}	#
1	115.0502	58.0287	98.0237	49.5155			N							24
2	202.0822	101.5448	185.0557	93.0315	184.0717	92.5395	S	2419.3079	1210.1576	2402.2813	1201.6443	2401.2973	1201.1523	23
3	315.1663	158.0868	298.1397	149.5735	297.1557	149.0815	I	2332.2759	1166.6416	2315.2493	1158.1283	2314.2653	1157.6363	22
4	416.2140	208.6106	399.1874	200.0974	398.2034	199.6053	T	2219.1918	1110.0995	2202.1652	1101.5863	2201.1812	1101.0943	21
5	529.2980	265.1527	512.2715	256.6394	511.2875	256.1474	L	2118.1441	1059.5757	2101.1176	1051.0624	2100.1335	1050.5704	20
6	**630.3457**	315.6765	613.3192	307.1632	612.3352	306.6712	T	2005.0600	1003.0337	1988.0335	994.5204	1987.0495	994.0284	19
7	744.3886	372.6980	727.3621	364.1847	726.3781	363.6927	N	1904.0124	952.5098	1886.9858	943.9965	1886.0018	943.5045	18
8	857.4727	429.2400	840.4462	420.7267	839.4621	420.2347	L	1789.9694	895.4884	1772.9429	886.9751	1771.9589	886.4831	17
9	958.5204	479.7638	941.4938	471.2506	940.5098	470.7585	T	1676.8854	838.9463	1659.8588	830.4331	1658.8748	829.9410	16
10	1055.5732	528.2902	1038.5466	519.7769	1037.5626	519.2849	P	***1575.8377***	788.4225	1558.8112	779.9092	1557.8271	779.4172	15
11	1112.5946	556.8009	1095.5681	548.2877	1094.5840	547.7957	G	1478.7849	739.8961	1461.7584	731.3828	1460.7744	730.8908	14
12	1213.6423	607.3248	1196.6157	598.8115	1195.6317	598.3195	T	1421.7635	711.3854	1404.7369	702.8721	1403.7529	702.3801	13
13	1342.6849	671.8461	1325.6583	663.3328	1324.6743	662.8408	E	1320.7158	660.8615	1303.6892	652.3483	1302.7052	651.8563	12
14	1505.7482	753.3777	1488.7217	744.8645	1487.7377	744.3725	Y	1191.6732	596.3402	1174.6467	587.8270	1173.6626	587.3350	11
15	1604.8166	802.9120	1587.7901	794.3987	1586.8061	793.9067	V	1028.6099	514.8086	1011.5833	506.2953	1010.5993	505.8033	10
16	1703.8850	852.4462	1686.8585	843.9329	1685.8745	843.4409	V	***929.5415***	465.2744	912.5149	456.7611	911.5309	456.2691	9
17	1790.9171	895.9622	1773.8905	887.4489	1772.9065	886.9569	S	***830.4730***	415.7402	813.4465	407.2269	812.4625	406.7349	8
18	1904.0011	952.5042	1886.9746	943.9909	1885.9906	943.4989	I	***743.4410***	372.2241	726.4145	363.7109			7

**19**	2003.0696	1002.0384	1986.0430	993.5251	1985.0590	993.0331	**V**	***630.3570***	315.6821	613.3304	307.1688			**6**
**20**	2074.1067	1037.5570	2057.0801	1029.0437	2056.0961	1028.5517	**A**	***531.2885***	266.1479	514.2620	257.6346			**5**
**21**	2187.1907	1094.0990	2170.1642	1085.5857	2169.1802	1085.0937	**L**	***460.2514***	230.6293	443.2249	222.1161			**4**
**22**	2302.2177	1151.6125	2285.1911	1143.0992	2284.2071	1142.6072	**N**	***347.1674***	174.0873	330.1408	165.5740			**3**
**23**	2359.2391	1180.1232	2342.2126	1171.6099	2341.2286	1171.1179	**G**	***232.1404***	116.5738	215.1139	108.0606			**2**
**24**							**R**	175.1190	88.0631	158.0924	79.5498			**1**

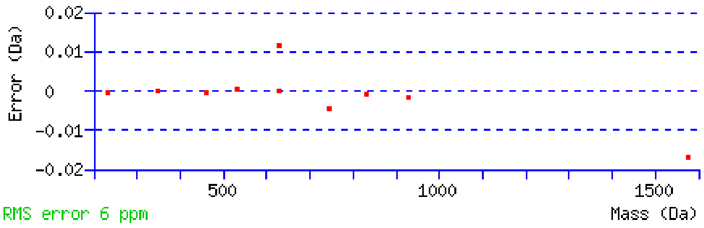

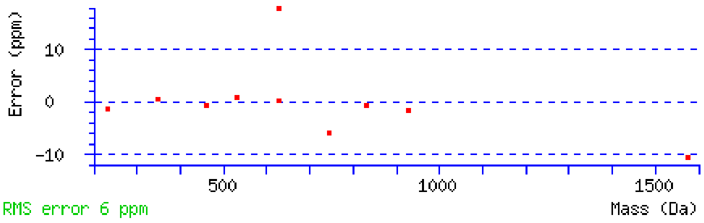

NCBI **BLAST** search of NSITLTNLTPGTEYVVSIVALNGR
(Parameters: blastp, nr protein database, expect=20000, no filter, PAM30)
Other BLAST web gateways

All matches to this query

Score	Mr(calc)	Delta	Sequence
71.7	2532.3435	-0.0011	NSITLTNLTPGTEYVVSIVALNGR
0.5	2532.3370	0.0054	VIKPTSSNTAQGRFDTSILPICK

Mascot: http://www.matrixscience.com/

Mascot Search Results

Peptide View

MS/MS Fragmentation of **NSITLTNLTPGTEYVVSIVALNGR**
Found in **P02751** in **uni_human_nr**, FINC_HUMAN Fibronectin OS=Homo sapiens GN=FN1 PE=1 SV=4

Match to Query 12550: 2532.343092 from(845.121640,3+) intensity(814495.3750) rtinseconds(3827) scans(23938) index(19028)
Title: 130801_HPL_Human_Plaque_BR2_TR1_05_Spectrum041093_scans__23938_RTINSECONDS=3827
Data file E:\\RAW\\HumanPlaques\\mgf\\T\\1D\\T130801_HPL_Human_Plaque_BR2_TR1_05.mgf

Click mouse within plot area to zoom in by factor of two about that point
Or, Plot from 0 to 1600 Da Full range
Label all possible matches ○ Label matches used for scoring ⊙
Show Y-axis ☐

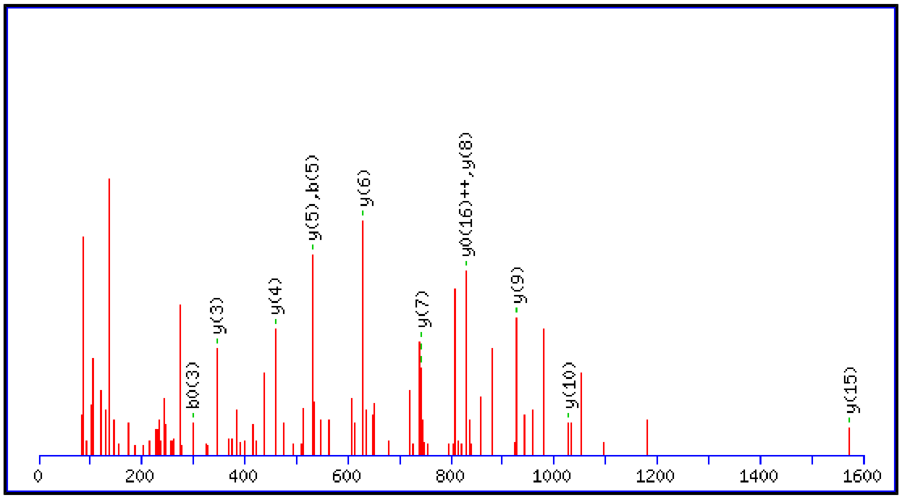

Monoisotopic mass of neutral peptide Mr(calc): 2532.3435
Fixed modifications: Carbamidomethyl (C) (apply to specified residues or termini only)
Variable modifications:
N1 : Deamidated (NQ)
Ions Score: 42 **Expect:** 0.0039
Matches : 12/260 fragment ions using 23 most intense peaks (help)

#	b	b^{++}	b*	b^{*++}	b^{0}	b^{0++}	Seq.	y	y^{++}	y*	y^{*++}	y^{0}	y^{0++}	#
1	116.0342	58.5207	99.0077	50.0075			N							24
2	203.0662	102.0368	186.0397	93.5235	185.0557	93.0315	S	2418.3239	1209.6656	2401.2973	1201.1523	2400.3133	1200.6603	23
3	316.1503	158.5788	299.1238	150.0655	298.1397	149.5735	I	2331.2918	1166.1496	2314.2653	1157.6363	2313.2813	1157.1443	22
4	417.1980	209.1026	400.1714	200.5894	399.1874	200.0974	T	2218.2078	1109.6075	2201.1812	1101.0943	2200.1972	1100.6022	21
5	530.2821	265.6447	513.2555	257.1314	512.2715	256.6394	L	2117.1601	1059.0837	2100.1335	1050.5704	2099.1495	1050.0784	20
6	631.3297	316.1685	614.3032	307.6552	613.3192	307.1632	T	2004.0760	1002.5417	1987.0495	994.0284	1986.0655	993.5364	19
7	745.3727	373.1900	728.3461	364.6767	727.3621	364.1847	N	1903.0284	952.0178	1886.0018	943.5045	1885.0178	943.0125	18
8	858.4567	429.7320	841.4302	421.2187	840.4462	420.7267	L	1788.9854	894.9964	1771.9589	886.4831	1770.9749	885.9911	17
9	959.5044	480.2558	942.4779	471.7426	941.4938	471.2506	T	1675.9014	838.4543	1658.8748	829.9410	1657.8908	829.4490	16
10	1056.5572	528.7822	1039.5306	520.2689	1038.5466	519.7769	P	*1574.8537*	787.9305	1557.8271	779.4172	1556.8431	778.9252	15
11	1113.5786	557.2930	1096.5521	548.7797	1095.5681	548.2877	G	1477.8009	739.4041	1460.7744	730.8908	1459.7904	730.3988	14
12	1214.6263	607.8168	1197.5998	599.3035	1196.6157	598.8115	T	1420.7795	710.8934	1403.7529	702.3801	1402.7689	701.8881	13
13	1343.6689	672.3381	1326.6424	663.8248	1325.6583	663.3328	E	1319.7318	660.3695	1302.7052	651.8563	1301.7212	651.3642	12
14	1506.7322	753.8698	1489.7057	745.3565	1488.7217	744.8645	Y	1190.6892	595.8482	1173.6626	587.3350	1172.6786	586.8429	11
15	1605.8006	803.4040	1588.7741	794.8907	1587.7901	794.3987	V	*1027.6259*	514.3166	1010.5993	505.8033	1009.6153	505.3113	10
16	1704.8691	852.9382	1687.8425	844.4249	1686.8585	843.9329	V	*928.5574*	464.7824	911.5309	456.2691	910.5469	455.7771	9
17	1791.9011	896.4542	1774.8745	887.9409	1773.8905	887.4489	S	*829.4890*	415.2482	812.4625	406.7349	811.4785	406.2429	8
18	1904.9852	952.9962	1887.9586	944.4829	1886.9746	943.9909	I	*742.4570*	371.7321	725.4305	363.2189			7

**19**	2004.0536	1002.5304	1987.0270	994.0171	1986.0430	993.5251	**V**	***629.3729***	315.1901	612.3464	306.6768			**6**
**20**	2075.0907	1038.0490	2058.0641	1029.5357	2057.0801	1029.0437	**A**	***530.3045***	265.6559	513.2780	257.1426			**5**
**21**	2188.1747	1094.5910	2171.1482	1086.0777	2170.1642	1085.5857	**L**	***459.2674***	230.1373	442.2409	221.6241			**4**
**22**	2302.2177	1151.6125	2285.1911	1143.0992	2284.2071	1142.6072	**N**	***346.1833***	173.5953	329.1568	165.0820			**3**
**23**	2359.2391	1180.1232	2342.2126	1171.6099	2341.2286	1171.1179	**G**	232.1404	116.5738	215.1139	108.0606			**2**
**24**							**R**	175.1190	88.0631	158.0924	79.5498			**1**

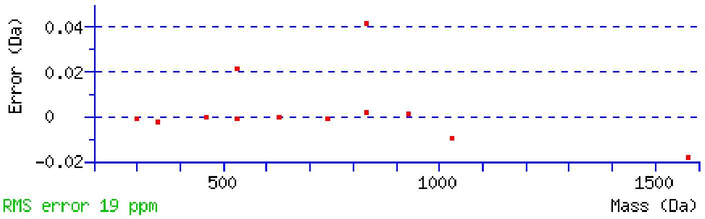

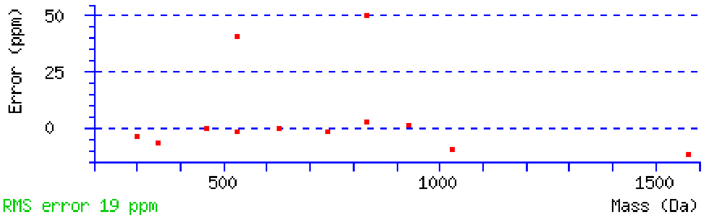

NCBI **BLAST** search of NSITLTNLTPGTEYVVSIVALNGR
(Parameters: blastp, nr protein database, expect=20000, no filter, PAM30)
Other BLAST web gateways

All matches to this query

Score	Mr(calc)	Delta	Sequence	Site Analysis
41.6	2532.3435	-0.0004	NSITLTNLTPGTEYVVSIVALNGR	Deamidated N1 50.06%
39.1	2532.3435	-0.0004	NSITLTNLTPGTEYVVSIVALNGR	Deamidated N7 27.83%

Mascot: http://www.matrixscience.com/

Mascot Search Results

Peptide View

MS/MS Fragmentation of **NSITLTNLTPGTEYVVSIVALNGR**
Found in **P02751** in **uni_human_nr**, FINC_HUMAN Fibronectin OS=Homo sapiens GN=FN1 PE=1 SV=4

Match to Query 12550: 2532.343092 from(845.121640,3+) intensity(814495.3750) rtinseconds(3827) scans(23938) index(19028)
Title: 130801_HPL_Human_Plaque_BR2_TR1_05_Spectrum041093_scans__23938_RTINSECONDS=3827
Data file E:\\RAW\\HumanPlaques\\mgf\\T\\1D\\T130801_HPL_Human_Plaque_BR2_TR1_05.mgf

Click mouse within plot area to zoom in by factor of two about that point
Or, Plot from 0 to 1600 Da Full range
Label all possible matches Label matches used for scoring
Show Y-axis

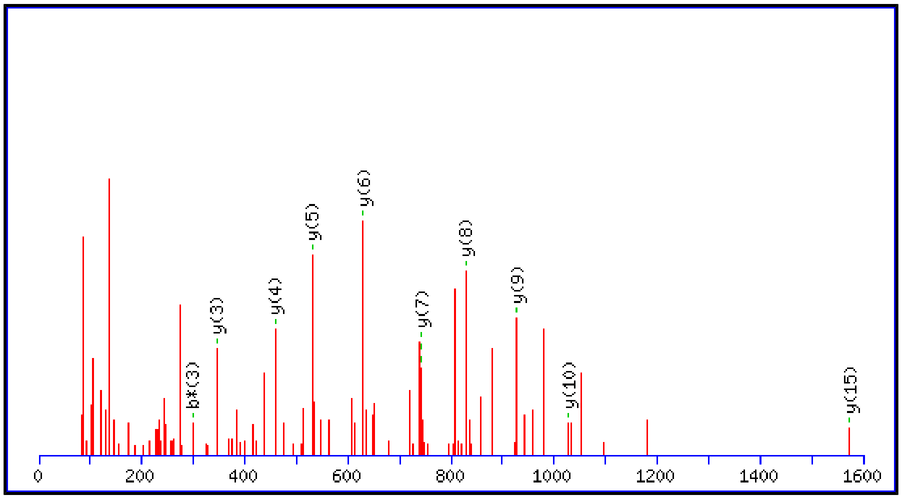

Monoisotopic mass of neutral peptide Mr(calc): 2532.3435
Fixed modifications: Carbamidomethyl (C) (apply to specified residues or termini only)
Variable modifications:
N7 : Deamidated (NQ)
Ions Score: 39 **Expect:** 0.0071
Matches : 10/260 fragment ions using 23 most intense peaks (help)

#	b	b^{++}	b*	b^{*++}	b^0	b^{0++}	Seq.	y	y^{++}	y*	y^{*++}	y^0	y^{0++}	#
1	115.0502	58.0287	98.0237	49.5155			N							24
2	202.0822	101.5448	185.0557	93.0315	184.0717	92.5395	S	2419.3079	1210.1576	2402.2813	1201.6443	2401.2973	1201.1523	23
3	315.1663	158.0868	298.1397	149.5735	297.1557	149.0815	I	2332.2759	1166.6416	2315.2493	1158.1283	2314.2653	1157.6363	22
4	416.2140	208.6106	399.1874	200.0974	398.2034	199.6053	T	2219.1918	1110.0995	2202.1652	1101.5863	2201.1812	1101.0943	21
5	529.2980	265.1527	512.2715	256.6394	511.2875	256.1474	L	2118.1441	1059.5757	2101.1176	1051.0624	2100.1335	1050.5704	20
6	630.3457	315.6765	613.3192	307.1632	612.3352	306.6712	T	2005.0600	1003.0337	1988.0335	994.5204	1987.0495	994.0284	19
7	745.3727	373.1900	728.3461	364.6767	727.3621	364.1847	N	1904.0124	952.5098	1886.9858	943.9965	1886.0018	943.5045	18
8	858.4567	429.7320	841.4302	421.2187	840.4462	420.7267	L	1788.9854	894.9964	1771.9589	886.4831	1770.9749	885.9911	17
9	959.5044	480.2558	942.4779	471.7426	941.4938	471.2506	T	1675.9014	838.4543	1658.8748	829.9410	1657.8908	829.4490	16
10	1056.5572	528.7822	1039.5306	520.2689	1038.5466	519.7769	P	***1574.8537***	787.9305	1557.8271	779.4172	1556.8431	778.9252	15
11	1113.5786	557.2930	1096.5521	548.7797	1095.5681	548.2877	G	1477.8009	739.4041	1460.7744	730.8908	1459.7904	730.3988	14
12	1214.6263	607.8168	1197.5998	599.3035	1196.6157	598.8115	T	1420.7795	710.8934	1403.7529	702.3801	1402.7689	701.8881	13
13	1343.6689	672.3381	1326.6424	663.8248	1325.6583	663.3328	E	1319.7318	660.3695	1302.7052	651.8563	1301.7212	651.3642	12
14	1506.7322	753.8698	1489.7057	745.3565	1488.7217	744.8645	Y	1190.6892	595.8482	1173.6626	587.3350	1172.6786	586.8429	11
15	1605.8006	803.4040	1588.7741	794.8907	1587.7901	794.3987	V	***1027.6259***	514.3166	1010.5993	505.8033	1009.6153	505.3113	10
16	1704.8691	852.9382	1687.8425	844.4249	1686.8585	843.9329	V	***928.5574***	464.7824	911.5309	456.2691	910.5469	455.7771	9
17	1791.9011	896.4542	1774.8745	887.9409	1773.8905	887.4489	S	***829.4890***	415.2482	812.4625	406.7349	811.4785	406.2429	8
18	1904.9852	952.9962	1887.9586	944.4829	1886.9746	943.9909	I	***742.4570***	371.7321	725.4305	363.2189			7

**19**	2004.0536	1002.5304	1987.0270	994.0171	1986.0430	993.5251	**V**	***629.3729***	315.1901	612.3464	306.6768			**6**
**20**	2075.0907	1038.0490	2058.0641	1029.5357	2057.0801	1029.0437	**A**	***530.3045***	265.6559	513.2780	257.1426			**5**
**21**	2188.1747	1094.5910	2171.1482	1086.0777	2170.1642	1085.5857	**L**	***459.2674***	230.1373	442.2409	221.6241			**4**
**22**	2302.2177	1151.6125	2285.1911	1143.0992	2284.2071	1142.6072	**N**	***346.1833***	173.5953	329.1568	165.0820			**3**
**23**	2359.2391	1180.1232	2342.2126	1171.6099	2341.2286	1171.1179	**G**	232.1404	116.5738	215.1139	108.0606			**2**
**24**							**R**	175.1190	88.0631	158.0924	79.5498			**1**

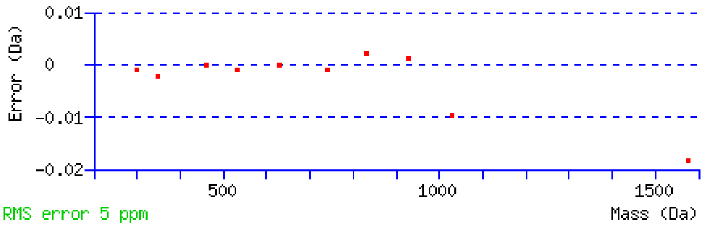

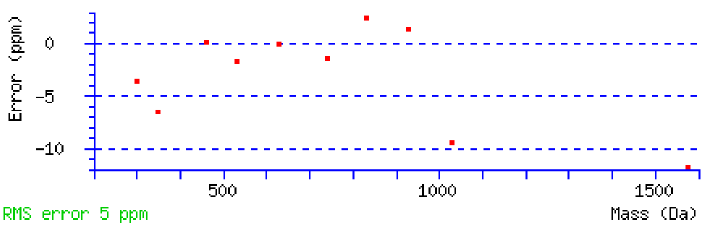

NCBI **BLAST** search of NSITLTNLTPGTEYVVSIVALNGR
(Parameters: blastp, nr protein database, expect=20000, no filter, PAM30)
Other BLAST web gateways

All matches to this query

Score	Mr(calc)	Delta	Sequence	Site Analysis
39.1	2532.3435	-0.0004	NSITLTNLTPGTEYVVSIVALNGR	Deamidated N7 35.79%
39.1	2532.3435	-0.0004	NSITLTNLTPGTEYVVSIVALNGR	Deamidated N1 35.79%

Mascot: http://www.matrixscience.com/

Mascot Search Results

Peptide View

MS/MS Fragmentation of **NSITLTNLTPGTEYVVSIVALNGR**
Found in **P02751** in **uni_human_nr**, FINC_HUMAN Fibronectin OS=Homo sapiens GN=FN1 PE=1 SV=4

Match to Query 14719: 2531.360208 from(1266.687380,2+) intensity(10694606.0000) rtinseconds(3420) scans(23631) index(19605)
Title: 130806_HPL_Human_Plaque_BR1_TR2_02_Spectrum044309_scans__23631_RTINSECONDS=3420
Data file E:\\RAW\\HumanPlaques\\mgf\\T\\1D\\T130806_HPL_Human_Plaque_BR1_TR2_02.mgf

Click mouse within plot area to zoom in by factor of two about that point
Or, [Plot from] [0] to [1700] Da [Full range]
Label all possible matches ○ Label matches used for scoring ⊙
Show Y-axis ☐

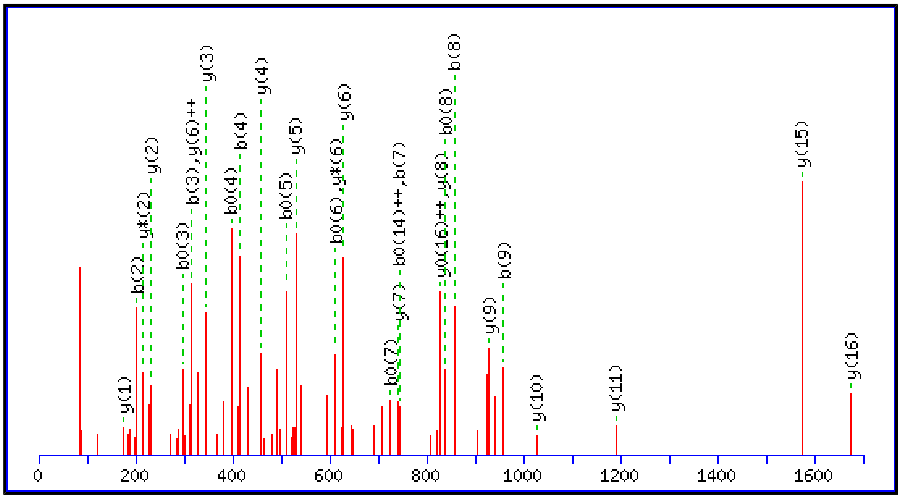

Monoisotopic mass of neutral peptide Mr(calc): 2531.3595
Fixed modifications: Carbamidomethyl (C) (apply to specified residues or termini only)
Ions Score: 113 **Expect:** 2.2e-010
Matches : 30/260 fragment ions using 32 most intense peaks (help)

#	b	b^{++}	b*	b^{*++}	b^0	b^{0++}	Seq.	y	y^{++}	y*	y^{*++}	y^0	y^{0++}	#
1	115.0502	58.0287	98.0237	49.5155			N							24
2	**202.0822**	101.5448	185.0557	93.0315	184.0717	92.5395	S	2418.3239	1209.6656	2401.2973	1201.1523	2400.3133	1200.6603	23
3	**315.1663**	158.0868	298.1397	149.5735	297.1557	149.0815	I	2331.2918	1166.1496	2314.2653	1157.6363	2313.2813	1157.1443	22
4	**416.2140**	208.6106	399.1874	200.0974	398.2034	199.6053	T	2218.2078	1109.6075	2201.1812	1101.0943	2200.1972	1100.6022	21
5	529.2980	265.1527	512.2715	256.6394	511.2875	256.1474	L	2117.1601	1059.0837	2100.1335	1050.5704	2099.1495	1050.0784	20
6	630.3457	315.6765	613.3192	307.1632	612.3352	306.6712	T	2004.0760	1002.5417	1987.0495	994.0284	1986.0655	993.5364	19
7	**744.3886**	372.6980	727.3621	364.1847	726.3781	363.6927	N	1903.0284	952.0178	1886.0018	943.5045	1885.0178	943.0125	18
8	**857.4727**	429.2400	840.4462	420.7267	839.4621	420.2347	L	1788.9854	894.9964	1771.9589	886.4831	1770.9749	885.9911	17
9	**958.5204**	479.7638	941.4938	471.2506	940.5098	470.7585	T	***1675.9014***	838.4543	1658.8748	829.9410	1657.8908	829.4490	16
10	1055.5732	528.2902	1038.5466	519.7769	1037.5626	519.2849	P	***1574.8537***	787.9305	1557.8271	779.4172	1556.8431	778.9252	15
11	1112.5946	556.8009	1095.5681	548.2877	1094.5840	547.7957	G	1477.8009	739.4041	1460.7744	730.8908	1459.7904	730.3988	14
12	1213.6423	607.3248	1196.6157	598.8115	1195.6317	598.3195	T	1420.7795	710.8934	1403.7529	702.3801	1402.7689	701.8881	13
13	1342.6849	671.8461	1325.6583	663.3328	1324.6743	662.8408	E	1319.7318	660.3695	1302.7052	651.8563	1301.7212	651.3642	12
14	1505.7482	753.3777	1488.7217	744.8645	1487.7377	744.3725	Y	***1190.6892***	595.8482	1173.6626	587.3350	1172.6786	586.8429	11
15	1604.8166	802.9120	1587.7901	794.3987	1586.8061	793.9067	V	***1027.6259***	514.3166	1010.5993	505.8033	1009.6153	505.3113	10
16	1703.8850	852.4462	1686.8585	843.9329	1685.8745	843.4409	V	***928.5574***	464.7824	911.5309	456.2691	910.5469	455.7771	9
17	1790.9171	895.9622	1773.8905	887.4489	1772.9065	886.9569	S	***829.4890***	415.2482	812.4625	406.7349	811.4785	406.2429	8
18	1904.0011	952.5042	1886.9746	943.9909	1885.9906	943.4989	I	***742.4570***	371.7321	725.4305	363.2189			7
19	2003.0696	1002.0384	1986.0430	993.5251	1985.0590	993.0331	V	***629.3729***	315.1901	612.3464	306.6768			6

**20**	2074.1067	1037.5570	2057.0801	1029.0437	2056.0961	1028.5517	**A**	***530.3045***	265.6559	513.2780	257.1426			**5**
**21**	2187.1907	1094.0990	2170.1642	1085.5857	2169.1802	1085.0937	**L**	***459.2674***	230.1373	442.2409	221.6241			**4**
**22**	2301.2337	1151.1205	2284.2071	1142.6072	2283.2231	1142.1152	**N**	***346.1833***	173.5953	329.1568	165.0820			**3**
**23**	2358.2551	1179.6312	2341.2286	1171.1179	2340.2446	1170.6259	**G**	***232.1404***	116.5738	215.1139	108.0606			**2**
**24**							**R**	***175.1190***	88.0631	158.0924	79.5498			**1**

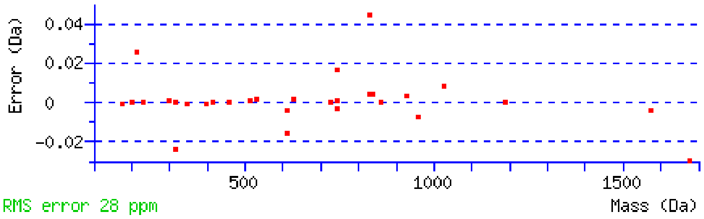

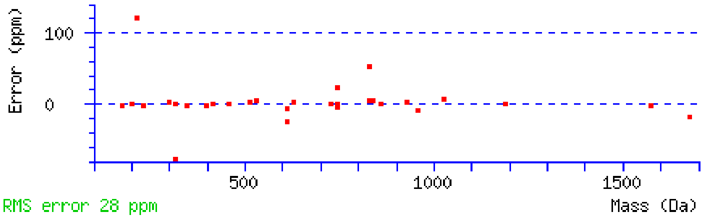

NCBI **BLAST** search of NSITLTNLTPGTEYVVSIVALNGR
(Parameters: blastp, nr protein database, expect=20000, no filter, PAM30)
Other BLAST web gateways

All matches to this query

Score	Mr(calc)	Delta	Sequence
112.8	2531.3595	0.0007	NSITLTNLTPGTEYVVSIVALNGR
0.5	2531.3682	-0.0080	LALRLYTCQEWAVVKVQAQVR

Mascot: http://www.matrixscience.com/

Mascot Search Results

Peptide View

MS/MS Fragmentation of **NSITLTNLTPGTEYVVSIVALNGR**
Found in **P02751** in **uni_human_nr**, FINC_HUMAN Fibronectin OS=Homo sapiens GN=FN1 PE=1 SV=4

Match to Query 14840: 2531.359452 from(844.793760,3+) intensity(22561322.0000) rtinseconds(3435) scans(23689) index(19659)
Title: 130809_HPL_Human_Plaque_BR1_TR3_02_Spectrum044185_scans__23689_RTINSECONDS=3435
Data file E:\\RAW\\HumanPlaques\\mgf\\T\\1D\\T130809_HPL_Human_Plaque_BR1_TR3_02.mgf

Click mouse within plot area to zoom in by factor of two about that point
Or, Plot from 0 to 1700 Da Full range
Label all possible matches ○ Label matches used for scoring ⊙
Show Y-axis ☐

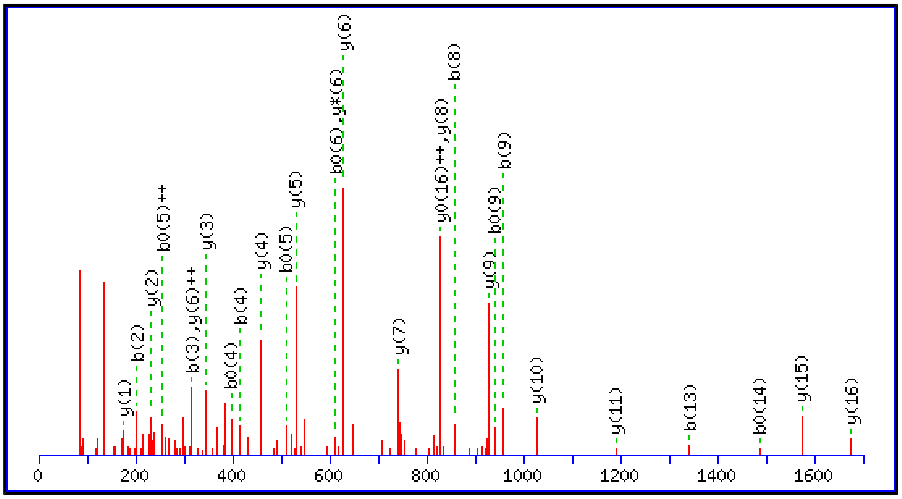

Monoisotopic mass of neutral peptide Mr(calc): 2531.3595
Fixed modifications: Carbamidomethyl (C) (apply to specified residues or termini only)
Ions Score: 103 **Expect:** 2e-009
Matches : 28/260 fragment ions using 34 most intense peaks (help)

#	b	b++	b*	b*++	b0	b0++	Seq.	y	y++	y*	y*++	y0	y0++	#
1	115.0502	58.0287	98.0237	49.5155			N							24
2	**202.0822**	101.5448	185.0557	93.0315	184.0717	92.5395	S	2418.3239	1209.6656	2401.2973	1201.1523	2400.3133	1200.6603	23
3	**315.1663**	158.0868	298.1397	149.5735	297.1557	149.0815	I	2331.2918	1166.1496	2314.2653	1157.6363	2313.2813	1157.1443	22
4	**416.2140**	208.6106	399.1874	200.0974	398.2034	199.6053	T	2218.2078	1109.6075	2201.1812	1101.0943	2200.1972	1100.6022	21
5	529.2980	265.1527	512.2715	256.6394	511.2875	256.1474	L	2117.1601	1059.0837	2100.1335	1050.5704	2099.1495	1050.0784	20
6	630.3457	315.6765	613.3192	307.1632	612.3352	306.6712	T	2004.0760	1002.5417	1987.0495	994.0284	1986.0655	993.5364	19
7	744.3886	372.6980	727.3621	364.1847	726.3781	363.6927	N	1903.0284	952.0178	1886.0018	943.5045	1885.0178	943.0125	18
8	**857.4727**	429.2400	840.4462	420.7267	839.4621	420.2347	L	1788.9854	894.9964	1771.9589	886.4831	1770.9749	885.9911	17
9	**958.5204**	479.7638	941.4938	471.2506	940.5098	470.7585	T	***1675.9014***	838.4543	1658.8748	829.9410	1657.8908	829.4490	16
10	1055.5732	528.2902	1038.5466	519.7769	1037.5626	519.2849	P	***1574.8537***	787.9305	1557.8271	779.4172	1556.8431	778.9252	15
11	1112.5946	556.8009	1095.5681	548.2877	1094.5840	547.7957	G	1477.8009	739.4041	1460.7744	730.8908	1459.7904	730.3988	14
12	1213.6423	607.3248	1196.6157	598.8115	1195.6317	598.3195	T	1420.7795	710.8934	1403.7529	702.3801	1402.7689	701.8881	13
13	**1342.6849**	671.8461	1325.6583	663.3328	1324.6743	662.8408	E	1319.7318	660.3695	1302.7052	651.8563	1301.7212	651.3642	12
14	1505.7482	753.3777	1488.7217	744.8645	1487.7377	744.3725	Y	***1190.6892***	595.8482	1173.6626	587.3350	1172.6786	586.8429	11
15	1604.8166	802.9120	1587.7901	794.3987	1586.8061	793.9067	V	***1027.6259***	514.3166	1010.5993	505.8033	1009.6153	505.3113	10
16	1703.8850	852.4462	1686.8585	843.9329	1685.8745	843.4409	V	***928.5574***	464.7824	911.5309	456.2691	910.5469	455.7771	9
17	1790.9171	895.9622	1773.8905	887.4489	1772.9065	886.9569	S	***829.4890***	415.2482	812.4625	406.7349	811.4785	406.2429	8
18	1904.0011	952.5042	1886.9746	943.9909	1885.9906	943.4989	I	***742.4570***	371.7321	725.4305	363.2189			7
19	2003.0696	1002.0384	1986.0430	993.5251	1985.0590	993.0331	V	***629.3729***	315.1901	612.3464	306.6768			6

**20**	2074.1067	1037.5570	2057.0801	1029.0437	2056.0961	1028.5517	**A**	***530.3045***	265.6559	513.2780	257.1426			**5**
**21**	2187.1907	1094.0990	2170.1642	1085.5857	2169.1802	1085.0937	**L**	***459.2674***	230.1373	442.2409	221.6241			**4**
**22**	2301.2337	1151.1205	2284.2071	1142.6072	2283.2231	1142.1152	**N**	***346.1833***	173.5953	329.1568	165.0820			**3**
**23**	2358.2551	1179.6312	2341.2286	1171.1179	2340.2446	1170.6259	**G**	***232.1404***	116.5738	215.1139	108.0606			**2**
**24**							**R**	***175.1190***	88.0631	158.0924	79.5498			**1**

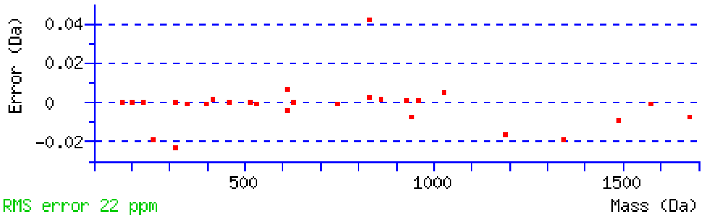

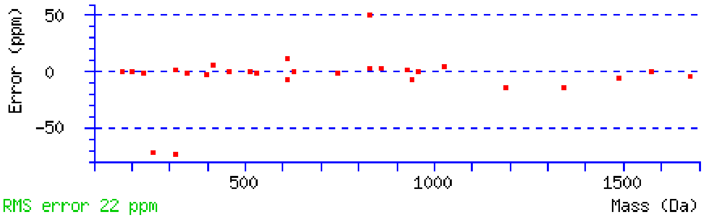

NCBI **BLAST** search of NSITLTNLTPGTEYVVSIVALNGR
(Parameters: blastp, nr protein database, expect=20000, no filter, PAM30)
Other BLAST web gateways

All matches to this query

Score	Mr(calc)	Delta	Sequence
103.3	2531.3595	-0.0001	NSITLTNLTPGTEYVVSIVALNGR
0.6	2531.3682	-0.0088	LALRLYTCQEWAVVKVQAQVR

Mascot: http://www.matrixscience.com/

Mascot Search Results

Peptide View

MS/MS Fragmentation of **NSITLTNLTPGTEYVVSIVALNGREESPLLIGQQSTVSDVPR**
Found in **P02751** in **uni_human_nr**, FINC_HUMAN Fibronectin OS=Homo sapiens GN=FN1 PE=1 SV=4

Match to Query 18731: 4471.339056 from(1118.842040,4+) intensity(688823.1875) rtinseconds(3653) scans(22315) index(17353)
Title: 130809_HPL_Human_Plaque_BR2_TR3_08_Spectrum038851_scans__22315_RTINSECONDS=3653
Data file E:\\RAW\\HumanPlaques\\mgf\\T\\1D\\T130809_HPL_Human_Plaque_BR2_TR3_08.mgf

Click mouse within plot area to zoom in by factor of two about that point
Or, Plot from 0 to 1300 Da Full range
Label all possible matches ○ Label matches used for scoring ⊙
Show Y-axis ☐

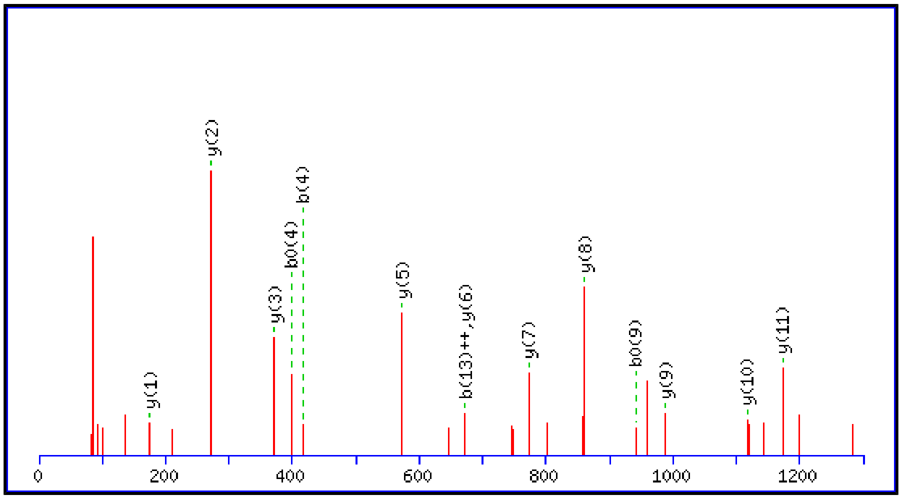

Monoisotopic mass of neutral peptide Mr(calc): 4469.3177
Fixed modifications: Carbamidomethyl (C) (apply to specified residues or termini only)
Variable modifications:
N7 : Deamidated (NQ)
N22 : Deamidated (NQ)
Ions Score: 47 **Expect:** 0.00046
Matches : 14/484 fragment ions using 25 most intense peaks (help)

#	b	b^{++}	b*	b^{*++}	b^{0}	b^{0++}	Seq.	y	y^{++}	y*	y^{*++}	y^{0}	y^{0++}	#
1	115.0502	58.0287	98.0237	49.5155			N							42
2	202.0822	101.5448	185.0557	93.0315	184.0717	92.5395	S	4356.2821	2178.6447	4339.2555	2170.1314	4338.2715	2169.6394	41
3	315.1663	158.0868	298.1397	149.5735	297.1557	149.0815	I	4269.2500	2135.1287	4252.2235	2126.6154	4251.2395	2126.1234	40
4	**416.2140**	208.6106	399.1874	200.0974	398.2034	199.6053	T	4156.1660	2078.5866	4139.1394	2070.0734	4138.1554	2069.5813	39
5	529.2980	265.1527	512.2715	256.6394	511.2875	256.1474	L	4055.1183	2028.0628	4038.0918	2019.5495	4037.1077	2019.0575	38
6	630.3457	315.6765	613.3192	307.1632	612.3352	306.6712	T	3942.0342	1971.5208	3925.0077	1963.0075	3924.0237	1962.5155	37
7	745.3727	373.1900	728.3461	364.6767	727.3621	364.1847	N	3840.9866	1920.9969	3823.9600	1912.4836	3822.9760	1911.9916	36
8	858.4567	429.7320	841.4302	421.2187	840.4462	420.7267	L	3725.9596	1863.4834	3708.9331	1854.9702	3707.9490	1854.4782	35
9	959.5044	480.2558	942.4779	471.7426	941.4938	471.2506	T	3612.8756	1806.9414	3595.8490	1798.4281	3594.8650	1797.9361	34
10	1056.5572	528.7822	1039.5306	520.2689	1038.5466	519.7769	P	3511.8279	1756.4176	3494.8013	1747.9043	3493.8173	1747.4123	33
11	1113.5786	557.2930	1096.5521	548.7797	1095.5681	548.2877	G	3414.7751	1707.8912	3397.7486	1699.3779	3396.7645	1698.8859	32
12	1214.6263	607.8168	1197.5998	599.3035	1196.6157	598.8115	T	3357.7536	1679.3805	3340.7271	1670.8672	3339.7431	1670.3752	31
13	1343.6689	**672.3381**	1326.6424	663.8248	1325.6583	663.3328	E	3256.7060	1628.8566	3239.6794	1620.3433	3238.6954	1619.8513	30
14	1506.7322	753.8698	1489.7057	745.3565	1488.7217	744.8645	Y	3127.6634	1564.3353	3110.6368	1555.8220	3109.6528	1555.3300	29
15	1605.8006	803.4040	1588.7741	794.8907	1587.7901	794.3987	V	2964.6000	1482.8037	2947.5735	1474.2904	2946.5895	1473.7984	28
16	1704.8691	852.9382	1687.8425	844.4249	1686.8585	843.9329	V	2865.5316	1433.2695	2848.5051	1424.7562	2847.5211	1424.2642	27
17	1791.9011	896.4542	1774.8745	887.9409	1773.8905	887.4489	S	2766.4632	1383.7352	2749.4367	1375.2220	2748.4526	1374.7300	26

18	1904.9852	952.9962	1887.9586	944.4829	1886.9746	943.9909	I	2679.4312	1340.2192	2662.4046	1331.7060	2661.4206	1331.2139	25
19	2004.0536	1002.5304	1987.0270	994.0171	1986.0430	993.5251	V	2566.3471	1283.6772	2549.3206	1275.1639	2548.3366	1274.6719	24
20	2075.0907	1038.0490	2058.0641	1029.5357	2057.0801	1029.0437	A	2467.2787	1234.1430	2450.2522	1225.6297	2449.2681	1225.1377	23
21	2188.1747	1094.5910	2171.1482	1086.0777	2170.1642	1085.5857	L	2396.2416	1198.6244	2379.2150	1190.1112	2378.2310	1189.6192	22
22	2303.2017	1152.1045	2286.1751	1143.5912	2285.1911	1143.0992	N	2283.1575	1142.0824	2266.1310	1133.5691	2265.1470	1133.0771	21
23	2360.2232	1180.6152	2343.1966	1172.1019	2342.2126	1171.6099	G	2168.1306	1084.5689	2151.1040	1076.0557	2150.1200	1075.5636	20
24	2516.3243	1258.6658	2499.2977	1250.1525	2498.3137	1249.6605	R	2111.1091	1056.0582	2094.0826	1047.5449	2093.0986	1047.0529	19
25	2645.3669	1323.1871	2628.3403	1314.6738	2627.3563	1314.1818	E	1955.0080	978.0076	1937.9815	969.4944	1936.9974	969.0024	18
26	2774.4094	1387.7084	2757.3829	1379.1951	2756.3989	1378.7031	E	1825.9654	913.4863	1808.9389	904.9731	1807.9549	904.4811	17
27	2861.4415	1431.2244	2844.4149	1422.7111	2843.4309	1422.2191	S	1696.9228	848.9651	1679.8963	840.4518	1678.9123	839.9598	16
28	2958.4942	1479.7508	2941.4677	1471.2375	2940.4837	1470.7455	P	1609.8908	805.4490	1592.8642	796.9358	1591.8802	796.4438	15
29	3071.5783	1536.2928	3054.5518	1527.7795	3053.5677	1527.2875	L	1512.8380	756.9227	1495.8115	748.4094	1494.8275	747.9174	14
30	3184.6624	1592.8348	3167.6358	1584.3215	3166.6518	1583.8295	L	1399.7540	700.3806	1382.7274	691.8673	1381.7434	691.3753	13
31	3297.7464	1649.3769	3280.7199	1640.8636	3279.7359	1640.3716	I	1286.6699	643.8386	1269.6434	635.3253	1268.6593	634.8333	12
32	3354.7679	1677.8876	3337.7413	1669.3743	3336.7573	1668.8823	G	1173.5858	587.2966	1156.5593	578.7833	1155.5753	578.2913	11
33	3482.8265	1741.9169	3465.7999	1733.4036	3464.8159	1732.9116	Q	1116.5644	558.7858	1099.5378	550.2726	1098.5538	549.7805	10
34	3610.8851	1805.9462	3593.8585	1797.4329	3592.8745	1796.9409	Q	988.5058	494.7565	971.4793	486.2433	970.4952	485.7513	9
35	3697.9171	1849.4622	3680.8905	1840.9489	3679.9065	1840.4569	S	860.4472	430.7272	843.4207	422.2140	842.4367	421.7220	8
36	3798.9648	1899.9860	3781.9382	1891.4727	3780.9542	1890.9807	T	773.4152	387.2112	756.3886	378.6980	755.4046	378.2060	7
37	3898.0332	1949.5202	3881.0066	1941.0070	3880.0226	1940.5149	V	672.3675	336.6874	655.3410	328.1741	654.3570	327.6821	6
38	3985.0652	1993.0362	3968.0387	1984.5230	3967.0546	1984.0310	S	573.2991	287.1532	556.2726	278.6399	555.2885	278.1479	5
39	4100.0921	2050.5497	4083.0656	2042.0364	4082.0816	2041.5444	D	486.2671	243.6372	469.2405	235.1239	468.2565	234.6319	4
40	4199.1606	2100.0839	4182.1340	2091.5706	4181.1500	2091.0786	V	371.2401	186.1237	354.2136	177.6104			3
41	4296.2133	2148.6103	4279.1868	2140.0970	4278.2028	2139.6050	P	272.1717	136.5895	255.1452	128.0762			2
42							R	175.1190	88.0631	158.0924	79.5498			1

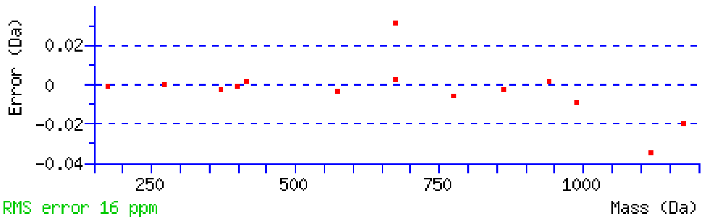

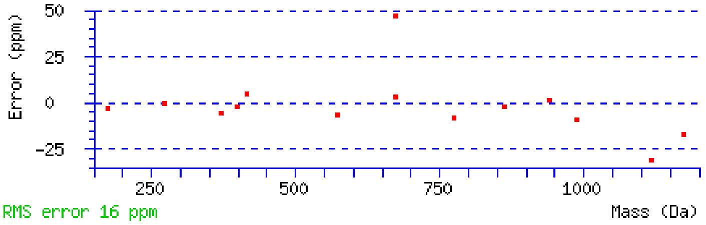

NCBI **BLAST** search of NSITLTNLTPGTEYVVSIVALNGREESPLLIGQQSTVSDVPR
(Parameters: blastp, nr protein database, expect=20000, no filter, PAM30)
Other BLAST web gateways

All matches to this query

Score	Mr(calc)	Delta	Sequence	Site Analysis
47.3	4469.3177	2.0214	NSITLTNLTPGTEYVVSIVALNGREESPLLIGQQSTVSDVPR	Deamidated N7, N22 32.86%
46.4	4469.3177	2.0214	NSITLTNLTPGTEYVVSIVALNGREESPLLIGQQSTVSDVPR	Deamidated N22, Q33 26.96%
43.6	4469.3177	2.0214	NSITLTNLTPGTEYVVSIVALNGREESPLLIGQQSTVSDVPR	Deamidated N1, N22 14.18%
43.6	4469.3177	2.0214	NSITLTNLTPGTEYVVSIVALNGREESPLLIGQQSTVSDVPR	Deamidated N1, N7 14.18%
38.6	4469.3177	2.0214	NSITLTNLTPGTEYVVSIVALNGREESPLLIGQQSTVSDVPR	Deamidated Q33, Q34 4.42%
38.6	4469.3177	2.0214	NSITLTNLTPGTEYVVSIVALNGREESPLLIGQQSTVSDVPR	Deamidated N22, Q34 4.42%
33.5	4469.3177	2.0214	NSITLTNLTPGTEYVVSIVALNGREESPLLIGQQSTVSDVPR	Deamidated N7, Q33 1.36%
33.1	4469.3177	2.0214	NSITLTNLTPGTEYVVSIVALNGREESPLLIGQQSTVSDVPR	Deamidated N1, Q33 1.25%
24.8	4469.3177	2.0214	NSITLTNLTPGTEYVVSIVALNGREESPLLIGQQSTVSDVPR	Deamidated N7, Q34 0.19%
24.4	4469.3177	2.0214	NSITLTNLTPGTEYVVSIVALNGREESPLLIGQQSTVSDVPR	Deamidated N1, Q34 0.17%

Mascot: http://www.matrixscience.com/

Mascot Search Results

Peptide View

MS/MS Fragmentation of **NSITLTNLTPGTEYVVSIVALNGREESPLLIGQQSTVSDVPR**
Found in **P02751** in **uni_human_nr**, FINC_HUMAN Fibronectin OS=Homo sapiens GN=FN1 PE=1 SV=4

Match to Query 18731: 4471.339056 from(1118.842040,4+) intensity(688823.1875) rtinseconds(3653) scans(22315) index(17353)
Title: 130809_HPL_Human_Plaque_BR2_TR3_08_Spectrum038851_scans__22315_RTINSECONDS=3653
Data file E:\\RAW\\HumanPlaques\\mgf\\T\\1D\\T130809_HPL_Human_Plaque_BR2_TR3_08.mgf

Click mouse within plot area to zoom in by factor of two about that point
Or, Plot from 0 to 1300 Da Full range
Label all possible matches Label matches used for scoring
Show Y-axis

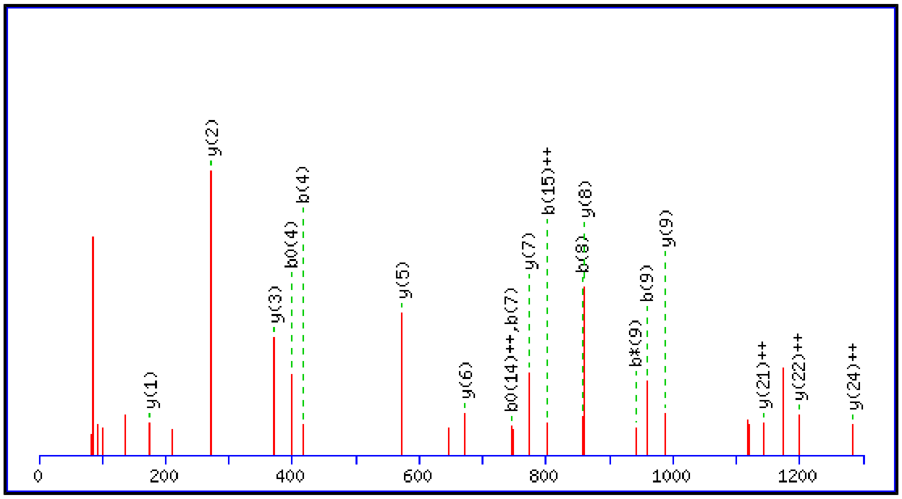

Monoisotopic mass of neutral peptide Mr(calc): 4469.3177
Fixed modifications: Carbamidomethyl (C) (apply to specified residues or termini only)
Variable modifications:
N22 : Deamidated (NQ)
Q33 : Deamidated (NQ)
Ions Score: 46 **Expect:** 0.00056
Matches : 19/484 fragment ions using 25 most intense peaks (help)

#	b	b++	b*	b*++	b0	b0++	Seq.	y	y++	y*	y*++	y0	y0++	#
1	115.0502	58.0287	98.0237	49.5155			N							42
2	202.0822	101.5448	185.0557	93.0315	184.0717	92.5395	S	4356.2821	2178.6447	4339.2555	2170.1314	4338.2715	2169.6394	41
3	315.1663	158.0868	298.1397	149.5735	297.1557	149.0815	I	4269.2500	2135.1287	4252.2235	2126.6154	4251.2395	2126.1234	40
4	***416.2140***	208.6106	399.1874	200.0974	398.2034	199.6053	T	4156.1660	2078.5866	4139.1394	2070.0734	4138.1554	2069.5813	39
5	529.2980	265.1527	512.2715	256.6394	511.2875	256.1474	L	4055.1183	2028.0628	4038.0918	2019.5495	4037.1077	2019.0575	38
6	630.3457	315.6765	613.3192	307.1632	612.3352	306.6712	T	3942.0342	1971.5208	3925.0077	1963.0075	3924.0237	1962.5155	37
7	***744.3886***	372.6980	727.3621	364.1847	726.3781	363.6927	N	3840.9866	1920.9969	3823.9600	1912.4836	3822.9760	1911.9916	36
8	***857.4727***	429.2400	840.4462	420.7267	839.4621	420.2347	L	3726.9436	1863.9755	3709.9171	1855.4622	3708.9331	1854.9702	35
9	***958.5204***	479.7638	941.4938	471.2506	940.5098	470.7585	T	3613.8596	1807.4334	3596.8330	1798.9201	3595.8490	1798.4281	34
10	1055.5732	528.2902	1038.5466	519.7769	1037.5626	519.2849	P	3512.8119	1756.9096	3495.7853	1748.3963	3494.8013	1747.9043	33
11	1112.5946	556.8009	1095.5681	548.2877	1094.5840	547.7957	G	3415.7591	1708.3832	3398.7326	1699.8699	3397.7486	1699.3779	32
12	1213.6423	607.3248	1196.6157	598.8115	1195.6317	598.3195	T	3358.7377	1679.8725	3341.7111	1671.3592	3340.7271	1670.8672	31
13	1342.6849	671.8461	1325.6583	663.3328	1324.6743	662.8408	E	3257.6900	1629.3486	3240.6634	1620.8354	3239.6794	1620.3433	30
14	1505.7482	753.3777	1488.7217	744.8645	1487.7377	744.3725	Y	3128.6474	1564.8273	3111.6208	1556.3141	3110.6368	1555.8220	29
15	1604.8166	**802.9120**	1587.7901	794.3987	1586.8061	793.9067	V	2965.5841	1483.2957	2948.5575	1474.7824	2947.5735	1474.2904	28
16	1703.8850	852.4462	1686.8585	843.9329	1685.8745	843.4409	V	2866.5156	1433.7615	2849.4891	1425.2482	2848.5051	1424.7562	27
17	1790.9171	895.9622	1773.8905	887.4489	1772.9065	886.9569	S	2767.4472	1384.2273	2750.4207	1375.7140	2749.4367	1375.2220	26

**18**	1904.0011	952.5042	1886.9746	943.9909	1885.9906	943.4989	**I**	2680.4152	1340.7112	2663.3887	1332.1980	2662.4046	1331.7060	**25**
**19**	2003.0696	1002.0384	1986.0430	993.5251	1985.0590	993.0331	**V**	2567.3311	1284.1692	2550.3046	1275.6559	2549.3206	1275.1639	**24**
**20**	2074.1067	1037.5570	2057.0801	1029.0437	2056.0961	1028.5517	**A**	2468.2627	1234.6350	2451.2362	1226.1217	2450.2522	1225.6297	**23**
**21**	2187.1907	1094.0990	2170.1642	1085.5857	2169.1802	1085.0937	**L**	2397.2256	1199.1164	2380.1991	1190.6032	2379.2150	1190.1112	**22**
**22**	2302.2177	1151.6125	2285.1911	1143.0992	2284.2071	1142.6072	**N**	2284.1415	1142.5744	2267.1150	1134.0611	2266.1310	1133.5691	**21**
**23**	2359.2391	1180.1232	2342.2126	1171.6099	2341.2286	1171.1179	**G**	2169.1146	1085.0609	2152.0881	1076.5477	2151.1040	1076.0557	**20**
**24**	2515.3402	1258.1738	2498.3137	1249.6605	2497.3297	1249.1685	**R**	2112.0931	1056.5502	2095.0666	1048.0369	2094.0826	1047.5449	**19**
**25**	2644.3828	1322.6951	2627.3563	1314.1818	2626.3723	1313.6898	**E**	1955.9920	978.4997	1938.9655	969.9864	1937.9815	969.4944	**18**
**26**	2773.4254	1387.2164	2756.3989	1378.7031	2755.4149	1378.2111	**E**	1826.9494	913.9784	1809.9229	905.4651	1808.9389	904.9731	**17**
**27**	2860.4575	1430.7324	2843.4309	1422.2191	2842.4469	1421.7271	**S**	1697.9068	849.4571	1680.8803	840.9438	1679.8963	840.4518	**16**
**28**	2957.5102	1479.2588	2940.4837	1470.7455	2939.4997	1470.2535	**P**	1610.8748	805.9410	1593.8483	797.4278	1592.8642	796.9358	**15**
**29**	3070.5943	1535.8008	3053.5677	1527.2875	3052.5837	1526.7955	**L**	1513.8221	757.4147	1496.7955	748.9014	1495.8115	748.4094	**14**
**30**	3183.6784	1592.3428	3166.6518	1583.8295	3165.6678	1583.3375	**L**	1400.7380	700.8726	1383.7114	692.3594	1382.7274	691.8673	**13**
**31**	3296.7624	1648.8848	3279.7359	1640.3716	3278.7519	1639.8796	**I**	1287.6539	644.3306	1270.6274	635.8173	1269.6434	635.3253	**12**
**32**	3353.7839	1677.3956	3336.7573	1668.8823	3335.7733	1668.3903	**G**	1174.5699	587.7886	1157.5433	579.2753	1156.5593	578.7833	**11**
**33**	3482.8265	1741.9169	3465.7999	1733.4036	3464.8159	1732.9116	**Q**	1117.5484	559.2778	1100.5218	550.7646	1099.5378	550.2726	**10**
**34**	3610.8851	1805.9462	3593.8585	1797.4329	3592.8745	1796.9409	**Q**	***988.5058***	494.7565	971.4793	486.2433	970.4952	485.7513	**9**
**35**	3697.9171	1849.4622	3680.8905	1840.9489	3679.9065	1840.4569	**S**	***860.4472***	430.7272	843.4207	422.2140	842.4367	421.7220	**8**
**36**	3798.9648	1899.9860	3781.9382	1891.4727	3780.9542	1890.9807	**T**	***773.4152***	387.2112	756.3886	378.6980	755.4046	378.2060	**7**
**37**	3898.0332	1949.5202	3881.0066	1941.0070	3880.0226	1940.5149	**V**	***672.3675***	336.6874	655.3410	328.1741	654.3570	327.6821	**6**
**38**	3985.0652	1993.0362	3968.0387	1984.5230	3967.0546	1984.0310	**S**	***573.2991***	287.1532	556.2726	278.6399	555.2885	278.1479	**5**
**39**	4100.0921	2050.5497	4083.0656	2042.0364	4082.0816	2041.5444	**D**	486.2671	243.6372	469.2405	235.1239	468.2565	234.6319	**4**
**40**	4199.1606	2100.0839	4182.1340	2091.5706	4181.1500	2091.0786	**V**	***371.2401***	186.1237	354.2136	177.6104			**3**
**41**	4296.2133	2148.6103	4279.1868	2140.0970	4278.2028	2139.6050	**P**	***272.1717***	136.5895	255.1452	128.0762			**2**
**42**							**R**	***175.1190***	88.0631	158.0924	79.5498			**1**

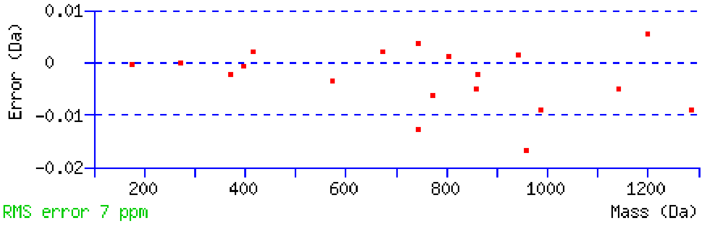

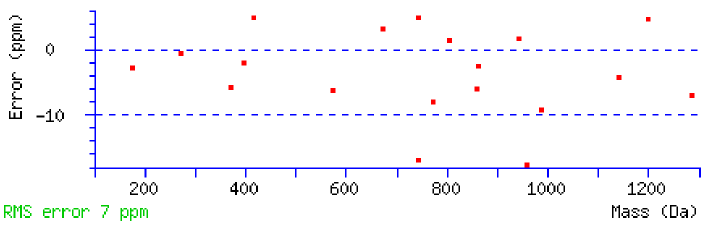

NCBI **BLAST** search of NSITLTNLTPGTEYVVSIVALNGREESPLLIGQQSTVSDVPR
(Parameters: blastp, nr protein database, expect=20000, no filter, PAM30)
Other BLAST web gateways

All matches to this query

Score	Mr(calc)	Delta	Sequence	Site Analysis
46.4	4469.3177	2.0214	NSITLTNLTPGTEYVVSIVALNGREESPLLIGQQSTVSDVPR	Deamidated N22, Q33 32.67%
44.0	4469.3177	2.0214	NSITLTNLTPGTEYVVSIVALNGREESPLLIGQQSTVSDVPR	Deamidated N7, N22 18.63%
43.6	4469.3177	2.0214	NSITLTNLTPGTEYVVSIVALNGREESPLLIGQQSTVSDVPR	Deamidated N1, N22 17.19%
43.6	4469.3177	2.0214	NSITLTNLTPGTEYVVSIVALNGREESPLLIGQQSTVSDVPR	Deamidated N1, N7 17.19%
38.6	4469.3177	2.0214	NSITLTNLTPGTEYVVSIVALNGREESPLLIGQQSTVSDVPR	Deamidated Q33, Q34 5.36%
38.6	4469.3177	2.0214	NSITLTNLTPGTEYVVSIVALNGREESPLLIGQQSTVSDVPR	Deamidated N22, Q34 5.36%
33.5	4469.3177	2.0214	NSITLTNLTPGTEYVVSIVALNGREESPLLIGQQSTVSDVPR	Deamidated N7, Q33 1.65%
33.1	4469.3177	2.0214	NSITLTNLTPGTEYVVSIVALNGREESPLLIGQQSTVSDVPR	Deamidated N1, Q33 1.51%
24.8	4469.3177	2.0214	NSITLTNLTPGTEYVVSIVALNGREESPLLIGQQSTVSDVPR	Deamidated N7, Q34 0.23%
24.4	4469.3177	2.0214	NSITLTNLTPGTEYVVSIVALNGREESPLLIGQQSTVSDVPR	Deamidated N1, Q34 0.21%

Mascot: http://www.matrixscience.com/

Mascot Search Results

Peptide View

MS/MS Fragmentation of **NSITLTNLTPGTEYVVSIVALNGREESPLLIGQQSTVSDVPR**
Found in **P02751** in **uni_human_nr**, FINC_HUMAN Fibronectin OS=Homo sapiens GN=FN1 PE=1 SV=4

Match to Query 18277: 4468.334176 from(1118.090820,4+) intensity(905530.4375) rtinseconds(3650) scans(22060) index(16811)
Title: 130801_HPL_Human_Plaque_BR1_TR1_08_Spectrum038087_scans__22060_RTINSECONDS=3650
Data file E:\\RAW\\HumanPlaques\\mgf\\T\\1D\\T130801_HPL_Human_Plaque_BR1_TR1_08.mgf

Click mouse within plot area to zoom in by factor of two about that point
Or, [Plot from] [0] to [3800] Da [Full range]
Label all possible matches ○ Label matches used for scoring ⊙
Show Y-axis ☐

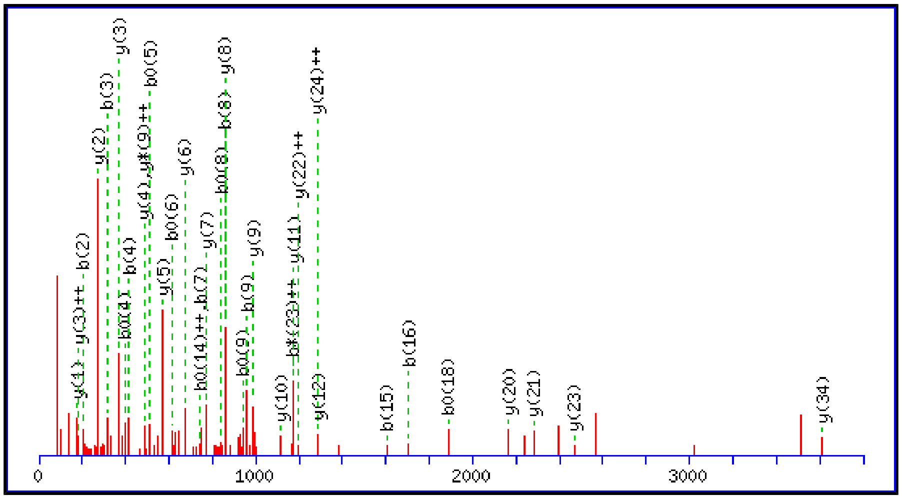


```
Monoisotopic mass of neutral peptide Mr(calc): 4468.3337
Fixed modifications: Carbamidomethyl (C) (apply to specified residues or termini only)
Variable modifications:
N22     : Deamidated (NQ)
Ions Score: 106  Expect: 6.9e-010
Matches : 36/484 fragment ions using 51 most intense peaks   (help)
```

#	b	b^{++}	b*	b^{*++}	b^0	b^{0++}	Seq.	y	y^{++}	y*	y^{*++}	y^0	y^{0++}	#
1	115.0502	58.0287	98.0237	49.5155			N							42
2	***202.0822***	101.5448	185.0557	93.0315	184.0717	92.5395	S	4355.2981	2178.1527	4338.2715	2169.6394	4337.2875	2169.1474	41
3	***315.1663***	158.0868	298.1397	149.5735	297.1557	149.0815	I	4268.2660	2134.6367	4251.2395	2126.1234	4250.2555	2125.6314	40
4	***416.2140***	208.6106	399.1874	200.0974	398.2034	199.6053	T	4155.1820	2078.0946	4138.1554	2069.5813	4137.1714	2069.0893	39
5	529.2980	265.1527	512.2715	256.6394	511.2875	256.1474	L	4054.1343	2027.5708	4037.1077	2019.0575	4036.1237	2018.5655	38
6	630.3457	315.6765	613.3192	307.1632	612.3352	306.6712	T	3941.0502	1971.0287	3924.0237	1962.5155	3923.0397	1962.0235	37
7	***744.3886***	372.6980	727.3621	364.1847	726.3781	363.6927	N	3840.0025	1920.5049	3822.9760	1911.9916	3821.9920	1911.4996	36
8	***857.4727***	429.2400	840.4462	420.7267	839.4621	420.2347	L	3725.9596	1863.4834	3708.9331	1854.9702	3707.9490	1854.4782	35
9	***958.5204***	479.7638	941.4938	471.2506	940.5098	470.7585	T	***3612.8756***	1806.9414	3595.8490	1798.4281	3594.8650	1797.9361	34
10	1055.5732	528.2902	1038.5466	519.7769	1037.5626	519.2849	P	3511.8279	1756.4176	3494.8013	1747.9043	3493.8173	1747.4123	33
11	1112.5946	556.8009	1095.5681	548.2877	1094.5840	547.7957	G	3414.7751	1707.8912	3397.7486	1699.3779	3396.7645	1698.8859	32
12	1213.6423	607.3248	1196.6157	598.8115	1195.6317	598.3195	T	3357.7536	1679.3805	3340.7271	1670.8672	3339.7431	1670.3752	31
13	1342.6849	671.8461	1325.6583	663.3328	1324.6743	662.8408	E	3256.7060	1628.8566	3239.6794	1620.3433	3238.6954	1619.8513	30
14	1505.7482	753.3777	1488.7217	744.8645	1487.7377	744.3725	Y	3127.6634	1564.3353	3110.6368	1555.8220	3109.6528	1555.3300	29
15	***1604.8166***	802.9120	1587.7901	794.3987	1586.8061	793.9067	V	2964.6000	1482.8037	2947.5735	1474.2904	2946.5895	1473.7984	28
16	***1703.8850***	852.4462	1686.8585	843.9329	1685.8745	843.4409	V	2865.5316	1433.2695	2848.5051	1424.7562	2847.5211	1424.2642	27
17	1790.9171	895.9622	1773.8905	887.4489	1772.9065	886.9569	S	2766.4632	1383.7352	2749.4367	1375.2220	2748.4526	1374.7300	26
18	1904.0011	952.5042	1886.9746	943.9909	1885.9906	943.4989	I	2679.4312	1340.2192	2662.4046	1331.7060	2661.4206	1331.2139	25

19	2003.0696	1002.0384	1986.0430	993.5251	1985.0590	993.0331	V	2566.3471	1283.6772	2549.3206	1275.1639	2548.3366	1274.6719	24
20	2074.1067	1037.5570	2057.0801	1029.0437	2056.0961	1028.5517	A	2467.2787	1234.1430	2450.2522	1225.6297	2449.2681	1225.1377	23
21	2187.1907	1094.0990	2170.1642	1085.5857	2169.1802	1085.0937	L	2396.2416	1198.6244	2379.2150	1190.1112	2378.2310	1189.6192	22
22	2302.2177	1151.6125	2285.1911	1143.0992	2284.2071	1142.6072	N	2283.1575	1142.0824	2266.1310	1133.5691	2265.1470	1133.0771	21
23	2359.2391	1180.1232	2342.2126	1171.6099	2341.2286	1171.1179	G	2168.1306	1084.5689	2151.1040	1076.0557	2150.1200	1075.5636	20
24	2515.3402	1258.1738	2498.3137	1249.6605	2497.3297	1249.1685	R	2111.1091	1056.0582	2094.0826	1047.5449	2093.0986	1047.0529	19
25	2644.3828	1322.6951	2627.3563	1314.1818	2626.3723	1313.6898	E	1955.0080	978.0076	1937.9815	969.4944	1936.9974	969.0024	18
26	2773.4254	1387.2164	2756.3989	1378.7031	2755.4149	1378.2111	E	1825.9654	913.4863	1808.9389	904.9731	1807.9549	904.4811	17
27	2860.4575	1430.7324	2843.4309	1422.2191	2842.4469	1421.7271	S	1696.9228	848.9651	1679.8963	840.4518	1678.9123	839.9598	16
28	2957.5102	1479.2588	2940.4837	1470.7455	2939.4997	1470.2535	P	1609.8908	805.4490	1592.8642	796.9358	1591.8802	796.4438	15
29	3070.5943	1535.8008	3053.5677	1527.2875	3052.5837	1526.7955	L	1512.8380	756.9227	1495.8115	748.4094	1494.8275	747.9174	14
30	3183.6784	1592.3428	3166.6518	1583.8295	3165.6678	1583.3375	L	1399.7540	700.3806	1382.7274	691.8673	1381.7434	691.3753	13
31	3296.7624	1648.8848	3279.7359	1640.3716	3278.7519	1639.8796	I	1286.6699	643.8386	1269.6434	635.3253	1268.6593	634.8333	12
32	3353.7839	1677.3956	3336.7573	1668.8823	3335.7733	1668.3903	G	1173.5858	587.2966	1156.5593	578.7833	1155.5753	578.2913	11
33	3481.8425	1741.4249	3464.8159	1732.9116	3463.8319	1732.4196	Q	1116.5644	558.7858	1099.5378	550.2726	1098.5538	549.7805	10
34	3609.9010	1805.4542	3592.8745	1796.9409	3591.8905	1796.4489	Q	988.5058	494.7565	971.4793	486.2433	970.4952	485.7513	9
35	3696.9331	1848.9702	3679.9065	1840.4569	3678.9225	1839.9649	S	860.4472	430.7272	843.4207	422.2140	842.4367	421.7220	8
36	3797.9807	1899.4940	3780.9542	1890.9807	3779.9702	1890.4887	T	773.4152	387.2112	756.3886	378.6980	755.4046	378.2060	7
37	3897.0492	1949.0282	3880.0226	1940.5149	3879.0386	1940.0229	V	672.3675	336.6874	655.3410	328.1741	654.3570	327.6821	6
38	3984.0812	1992.5442	3967.0546	1984.0310	3966.0706	1983.5389	S	573.2991	287.1532	556.2726	278.6399	555.2885	278.1479	5
39	4099.1081	2050.0577	4082.0816	2041.5444	4081.0976	2041.0524	D	486.2671	243.6372	469.2405	235.1239	468.2565	234.6319	4
40	4198.1765	2099.5919	4181.1500	2091.0786	4180.1660	2090.5866	V	371.2401	186.1237	354.2136	177.6104			3
41	4295.2293	2148.1183	4278.2028	2139.6050	4277.2187	2139.1130	P	272.1717	136.5895	255.1452	128.0762			2
42							R	175.1190	88.0631	158.0924	79.5498			1

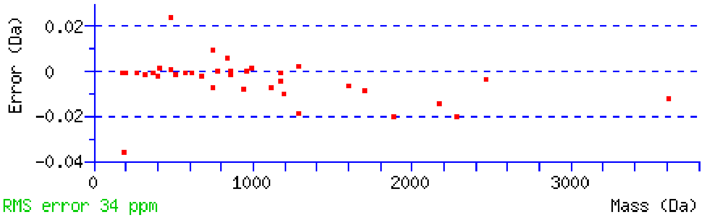

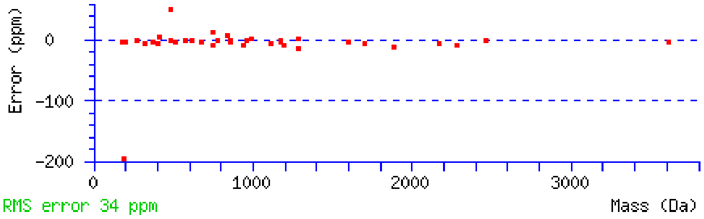

NCBI **BLAST** search of NSITLTNLTPGTEYVVSIVALNGREESPLLIGQQSTVSDVPR
(Parameters: blastp, nr protein database, expect=20000, no filter, PAM30)
Other BLAST web gateways

All matches to this query

Score	Mr(calc)	Delta	Sequence	Site Analysis
106.0	4468.3337	0.0005	NSITLTNLTPGTEYVVSIVALNGREESPLLIGQQSTVSDVPR	Deamidated N22 99.91%
76.5	4467.3497	0.9845	NSITLTNLTPGTEYVVSIVALNGREESPLLIGQQSTVSDVPR	
74.9	4468.3337	0.0005	NSITLTNLTPGTEYVVSIVALNGREESPLLIGQQSTVSDVPR	Deamidated Q33 0.08%
66.8	4468.3337	0.0005	NSITLTNLTPGTEYVVSIVALNGREESPLLIGQQSTVSDVPR	Deamidated Q34 0.01%
50.9	4468.3337	0.0005	NSITLTNLTPGTEYVVSIVALNGREESPLLIGQQSTVSDVPR	Deamidated N7 0.00%
39.1	4468.3337	0.0005	NSITLTNLTPGTEYVVSIVALNGREESPLLIGQQSTVSDVPR	Deamidated N1 0.00%

Mascot: http://www.matrixscience.com/

Mascot Search Results

Peptide View

MS/MS Fragmentation of **NSITLTNLTPGTEYVVSIVALNGREESPLLIGQQSTVSDVPR**
Found in **P02751** in **uni_human_nr**, FINC_HUMAN Fibronectin OS=Homo sapiens GN=FN1 PE=1 SV=4

Match to Query 19153: 4470.354696 from(1118.595950,4+) intensity(318403.0313) rtinseconds(3643) scans(22397) index(17446)
Title: 130801_HPL_Human_Plaque_BR1_TR1_07_Spectrum039149_scans__22397_RTINSECONDS=3643
Data file E:\\RAW\\HumanPlaques\\mgf\\T\\1D\\T130801_HPL_Human_Plaque_BR1_TR1_07.mgf

Click mouse within plot area to zoom in by factor of two about that point
Or, [Plot from] [0] to [1200] Da [Full range]
Label all possible matches ○ Label matches used for scoring ⊙
Show Y-axis ☐

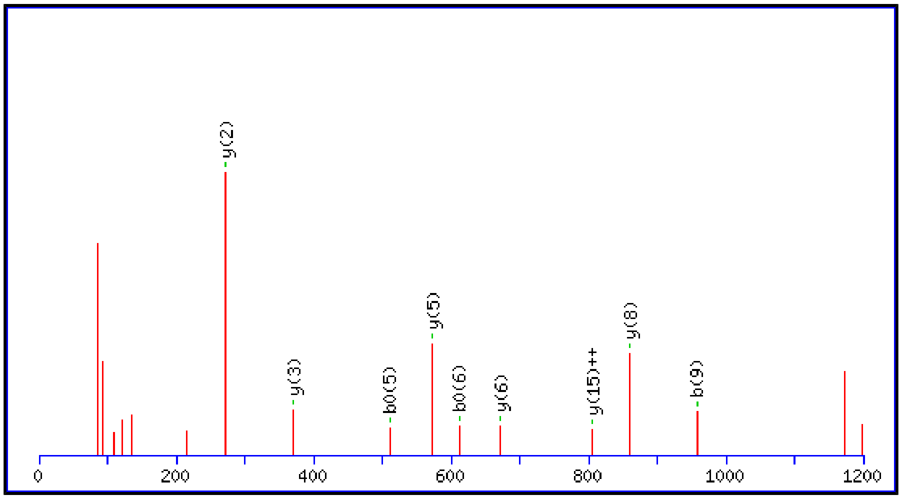

Monoisotopic mass of neutral peptide Mr(calc): 4468.3337
Fixed modifications: Carbamidomethyl (C) (apply to specified residues or termini only)
Variable modifications:
Q34 : Deamidated (NQ)
Ions Score: 22 **Expect:** 0.15
Matches : 9/484 fragment ions using 15 most intense peaks (help)

#	b	b^{++}	b*	b^{*++}	b^{0}	b^{0++}	Seq.	y	y^{++}	y*	y^{*++}	y^{0}	y^{0++}	#
1	115.0502	58.0287	98.0237	49.5155			N							42
2	202.0822	101.5448	185.0557	93.0315	184.0717	92.5395	S	4355.2981	2178.1527	4338.2715	2169.6394	4337.2875	2169.1474	41
3	315.1663	158.0868	298.1397	149.5735	297.1557	149.0815	I	4268.2660	2134.6367	4251.2395	2126.1234	4250.2555	2125.6314	40
4	416.2140	208.6106	399.1874	200.0974	398.2034	199.6053	T	4155.1820	2078.0946	4138.1554	2069.5813	4137.1714	2069.0893	39
5	529.2980	265.1527	512.2715	256.6394	511.2875	256.1474	L	4054.1343	2027.5708	4037.1077	2019.0575	4036.1237	2018.5655	38
6	630.3457	315.6765	613.3192	307.1632	612.3352	306.6712	T	3941.0502	1971.0287	3924.0237	1962.5155	3923.0397	1962.0235	37
7	744.3886	372.6980	727.3621	364.1847	726.3781	363.6927	N	3840.0025	1920.5049	3822.9760	1911.9916	3821.9920	1911.4996	36
8	857.4727	429.2400	840.4462	420.7267	839.4621	420.2347	L	3725.9596	1863.4834	3708.9331	1854.9702	3707.9490	1854.4782	35
9	958.5204	479.7638	941.4938	471.2506	940.5098	470.7585	T	3612.8756	1806.9414	3595.8490	1798.4281	3594.8650	1797.9361	34
10	1055.5732	528.2902	1038.5466	519.7769	1037.5626	519.2849	P	3511.8279	1756.4176	3494.8013	1747.9043	3493.8173	1747.4123	33
11	1112.5946	556.8009	1095.5681	548.2877	1094.5840	547.7957	G	3414.7751	1707.8912	3397.7486	1699.3779	3396.7645	1698.8859	32
12	1213.6423	607.3248	1196.6157	598.8115	1195.6317	598.3195	T	3357.7536	1679.3805	3340.7271	1670.8672	3339.7431	1670.3752	31
13	1342.6849	671.8461	1325.6583	663.3328	1324.6743	662.8408	E	3256.7060	1628.8566	3239.6794	1620.3433	3238.6954	1619.8513	30
14	1505.7482	753.3777	1488.7217	744.8645	1487.7377	744.3725	Y	3127.6634	1564.3353	3110.6368	1555.8220	3109.6528	1555.3300	29
15	1604.8166	802.9120	1587.7901	794.3987	1586.8061	793.9067	V	2964.6000	1482.8037	2947.5735	1474.2904	2946.5895	1473.7984	28
16	1703.8850	852.4462	1686.8585	843.9329	1685.8745	843.4409	V	2865.5316	1433.2695	2848.5051	1424.7562	2847.5211	1424.2642	27
17	1790.9171	895.9622	1773.8905	887.4489	1772.9065	886.9569	S	2766.4632	1383.7352	2749.4367	1375.2220	2748.4526	1374.7300	26
18	1904.0011	952.5042	1886.9746	943.9909	1885.9906	943.4989	I	2679.4312	1340.2192	2662.4046	1331.7060	2661.4206	1331.2139	25

**19**	2003.0696	1002.0384	1986.0430	993.5251	1985.0590	993.0331	**V**	2566.3471	1283.6772	2549.3206	1275.1639	2548.3366	1274.6719	**24**
**20**	2074.1067	1037.5570	2057.0801	1029.0437	2056.0961	1028.5517	**A**	2467.2787	1234.1430	2450.2522	1225.6297	2449.2681	1225.1377	**23**
**21**	2187.1907	1094.0990	2170.1642	1085.5857	2169.1802	1085.0937	**L**	2396.2416	1198.6244	2379.2150	1190.1112	2378.2310	1189.6192	**22**
**22**	2301.2337	1151.1205	2284.2071	1142.6072	2283.2231	1142.1152	**N**	2283.1575	1142.0824	2266.1310	1133.5691	2265.1470	1133.0771	**21**
**23**	2358.2551	1179.6312	2341.2286	1171.1179	2340.2446	1170.6259	**G**	2169.1146	1085.0609	2152.0881	1076.5477	2151.1040	1076.0557	**20**
**24**	2514.3562	1257.6818	2497.3297	1249.1685	2496.3457	1248.6765	**R**	2112.0931	1056.5502	2095.0666	1048.0369	2094.0826	1047.5449	**19**
**25**	2643.3988	1322.2030	2626.3723	1313.6898	2625.3883	1313.1978	**E**	1955.9920	978.4997	1938.9655	969.9864	1937.9815	969.4944	**18**
**26**	2772.4414	1386.7243	2755.4149	1378.2111	2754.4309	1377.7191	**E**	1826.9494	913.9784	1809.9229	905.4651	1808.9389	904.9731	**17**
**27**	2859.4734	1430.2404	2842.4469	1421.7271	2841.4629	1421.2351	**S**	1697.9068	849.4571	1680.8803	840.9438	1679.8963	840.4518	**16**
**28**	2956.5262	1478.7667	2939.4997	1470.2535	2938.5156	1469.7615	**P**	1610.8748	805.9410	1593.8483	797.4278	1592.8642	796.9358	**15**
**29**	3069.6103	1535.3088	3052.5837	1526.7955	3051.5997	1526.3035	**L**	1513.8221	757.4147	1496.7955	748.9014	1495.8115	748.4094	**14**
**30**	3182.6943	1591.8508	3165.6678	1583.3375	3164.6838	1582.8455	**L**	1400.7380	700.8726	1383.7114	692.3594	1382.7274	691.8673	**13**
**31**	3295.7784	1648.3928	3278.7519	1639.8796	3277.7678	1639.3876	**I**	1287.6539	644.3306	1270.6274	635.8173	1269.6434	635.3253	**12**
**32**	3352.7999	1676.9036	3335.7733	1668.3903	3334.7893	1667.8983	**G**	1174.5699	587.7886	1157.5433	579.2753	1156.5593	578.7833	**11**
**33**	3480.8584	1740.9329	3463.8319	1732.4196	3462.8479	1731.9276	**Q**	1117.5484	559.2778	1100.5218	550.7646	1099.5378	550.2726	**10**
**34**	3609.9010	1805.4542	3592.8745	1796.9409	3591.8905	1796.4489	**Q**	989.4898	495.2485	972.4633	486.7353	971.4793	486.2433	**9**
**35**	3696.9331	1848.9702	3679.9065	1840.4569	3678.9225	1839.9649	**S**	***860.4472***	430.7272	843.4207	422.2140	842.4367	421.7220	**8**
**36**	3797.9807	1899.4940	3780.9542	1890.9807	3779.9702	1890.4887	**T**	773.4152	387.2112	756.3886	378.6980	755.4046	378.2060	**7**
**37**	3897.0492	1949.0282	3880.0226	1940.5149	3879.0386	1940.0229	**V**	***672.3675***	336.6874	655.3410	328.1741	654.3570	327.6821	**6**
**38**	3984.0812	1992.5442	3967.0546	1984.0310	3966.0706	1983.5389	**S**	***573.2991***	287.1532	556.2726	278.6399	555.2885	278.1479	**5**
**39**	4099.1081	2050.0577	4082.0816	2041.5444	4081.0976	2041.0524	**D**	486.2671	243.6372	469.2405	235.1239	468.2565	234.6319	**4**
**40**	4198.1765	2099.5919	4181.1500	2091.0786	4180.1660	2090.5866	**V**	***371.2401***	186.1237	354.2136	177.6104			**3**
**41**	4295.2293	2148.1183	4278.2028	2139.6050	4277.2187	2139.1130	**P**	***272.1717***	136.5895	255.1452	128.0762			**2**
**42**							**R**	175.1190	88.0631	158.0924	79.5498			**1**

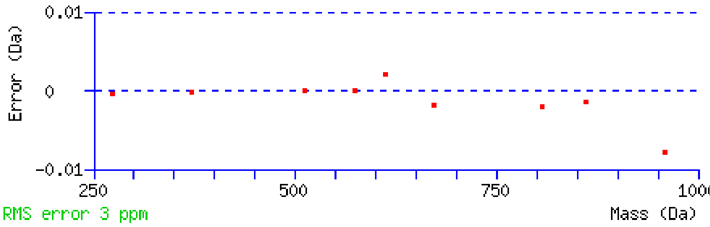

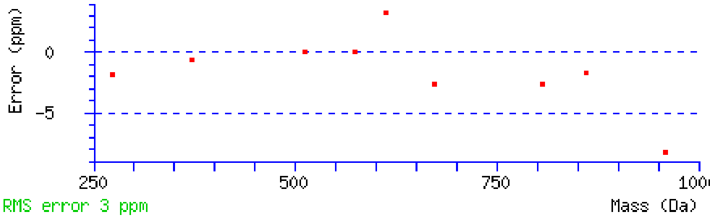

NCBI **BLAST** search of NSITLTNLTPGTEYVVSIVALNGREESPLLIGQQSTVSDVPR
(Parameters: blastp, nr protein database, expect=20000, no filter, PAM30)
Other BLAST web gateways

All matches to this query

Score	Mr(calc)	Delta	Sequence
21.6	4468.3337	2.0210	NSITLTNLTPGTEYVVSIVALNGREESPLLIGQQSTVSDVPR
21.6	4468.3337	2.0210	NSITLTNLTPGTEYVVSIVALNGREESPLLIGQQSTVSDVPR
20.0	4468.3337	2.0210	NSITLTNLTPGTEYVVSIVALNGREESPLLIGQQSTVSDVPR
12.8	4468.3337	2.0210	NSITLTNLTPGTEYVVSIVALNGREESPLLIGQQSTVSDVPR
12.8	4468.3337	2.0210	NSITLTNLTPGTEYVVSIVALNGREESPLLIGQQSTVSDVPR
1.9	4470.3645	-0.0098	GSEAIATLSLSNNQLSYLPPSLPPSLERLHLQNNLISKVPR
1.9	4470.3645	-0.0098	GSEAIATLSLSNNQLSYLPPSLPPSLERLHLQNNLISKVPR
1.9	4470.3645	-0.0098	GSEAIATLSLSNNQLSYLPPSLPPSLERLHLQNNLISKVPR
1.9	4470.3645	-0.0098	GSEAIATLSLSNNQLSYLPPSLPPSLERLHLQNNLISKVPR
1.9	4470.3645	-0.0098	GSEAIATLSLSNNQLSYLPPSLPPSLERLHLQNNLISKVPR

Mascot: http://www.matrixscience.com/

Mascot Search Results

Peptide View

MS/MS Fragmentation of **NSITLTNLTPGTEYVVSIVALNGREESPLLIGQQSTVSDVPR**
Found in **P02751** in **uni_human_nr**, FINC_HUMAN Fibronectin OS=Homo sapiens GN=FN1 PE=1 SV=4

Match to Query 22330: 4467.349816 from(1117.844730,4+) intensity(1484667.1250) rtinseconds(3623) scans(24220) index(19962)
Title: 130806_HPL_Human_Plaque_BR1_TR2_03_Spectrum043901_scans__24220_RTINSECONDS=3623
Data file E:\\RAW\\HumanPlaques\\mgf\\T\\1D\\T130806_HPL_Human_Plaque_BR1_TR2_03.mgf

Click mouse within plot area to zoom in by factor of two about that point
Or, [Plot from] [0] to [3600] Da [Full range]
Label all possible matches ○ Label matches used for scoring ⊙
Show Y-axis ☐

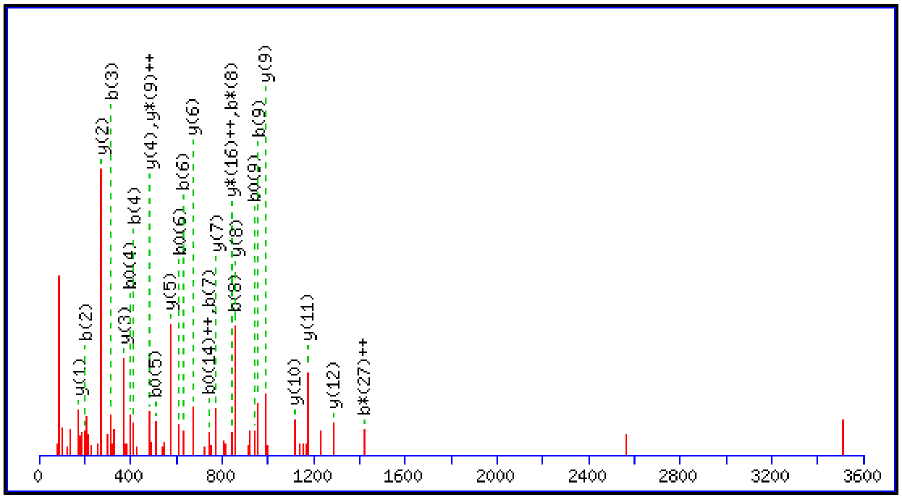

Monoisotopic mass of neutral peptide Mr(calc): 4467.3497
Fixed modifications: Carbamidomethyl (C) (apply to specified residues or termini only)
Ions Score: 81 **Expect:** 2.1e-007
Matches : 28/484 fragment ions using 38 most intense peaks (help)

#	b	b^{++}	b*	b^{*++}	b^0	b^{0++}	Seq.	y	y^{++}	y*	y^{*++}	y^0	y^{0++}	#
1	115.0502	58.0287	98.0237	49.5155			N							42
2	***202.0822***	101.5448	185.0557	93.0315	184.0717	92.5395	S	4354.3140	2177.6607	4337.2875	2169.1474	4336.3035	2168.6554	41
3	***315.1663***	158.0868	298.1397	149.5735	297.1557	149.0815	I	4267.2820	2134.1446	4250.2555	2125.6314	4249.2714	2125.1394	40
4	***416.2140***	208.6106	399.1874	200.0974	398.2034	199.6053	T	4154.1979	2077.6026	4137.1714	2069.0893	4136.1874	2068.5973	39
5	529.2980	265.1527	512.2715	256.6394	511.2875	256.1474	L	4053.1503	2027.0788	4036.1237	2018.5655	4035.1397	2018.0735	38
6	***630.3457***	315.6765	613.3192	307.1632	612.3352	306.6712	T	3940.0662	1970.5367	3923.0397	1962.0235	3922.0556	1961.5315	37
7	***744.3886***	372.6980	727.3621	364.1847	726.3781	363.6927	N	3839.0185	1920.0129	3821.9920	1911.4996	3821.0080	1911.0076	36
8	***857.4727***	429.2400	840.4462	420.7267	839.4621	420.2347	L	3724.9756	1862.9914	3707.9490	1854.4782	3706.9650	1853.9862	35
9	***958.5204***	479.7638	941.4938	471.2506	940.5098	470.7585	T	3611.8915	1806.4494	3594.8650	1797.9361	3593.8810	1797.4441	34
10	1055.5732	528.2902	1038.5466	519.7769	1037.5626	519.2849	P	3510.8439	1755.9256	3493.8173	1747.4123	3492.8333	1746.9203	33
11	1112.5946	556.8009	1095.5681	548.2877	1094.5840	547.7957	G	3413.7911	1707.3992	3396.7645	1698.8859	3395.7805	1698.3939	32
12	1213.6423	607.3248	1196.6157	598.8115	1195.6317	598.3195	T	3356.7696	1678.8885	3339.7431	1670.3752	3338.7591	1669.8832	31
13	1342.6849	671.8461	1325.6583	663.3328	1324.6743	662.8408	E	3255.7219	1628.3646	3238.6954	1619.8513	3237.7114	1619.3593	30
14	1505.7482	753.3777	1488.7217	744.8645	1487.7377	744.3725	Y	3126.6794	1563.8433	3109.6528	1555.3300	3108.6688	1554.8380	29
15	1604.8166	802.9120	1587.7901	794.3987	1586.8061	793.9067	V	2963.6160	1482.3117	2946.5895	1473.7984	2945.6055	1473.3064	28
16	1703.8850	852.4462	1686.8585	843.9329	1685.8745	843.4409	V	2864.5476	1432.7774	2847.5211	1424.2642	2846.5370	1423.7722	27
17	1790.9171	895.9622	1773.8905	887.4489	1772.9065	886.9569	S	2765.4792	1383.2432	2748.4526	1374.7300	2747.4686	1374.2380	26
18	1904.0011	952.5042	1886.9746	943.9909	1885.9906	943.4989	I	2678.4472	1339.7272	2661.4206	1331.2139	2660.4366	1330.7219	25
19	2003.0696	1002.0384	1986.0430	993.5251	1985.0590	993.0331	V	2565.3631	1283.1852	2548.3366	1274.6719	2547.3525	1274.1799	24

20	2074.1067	1037.5570	2057.0801	1029.0437	2056.0961	1028.5517	A	2466.2947	1233.6510	2449.2681	1225.1377	2448.2841	1224.6457	23
21	2187.1907	1094.0990	2170.1642	1085.5857	2169.1802	1085.0937	L	2395.2576	1198.1324	2378.2310	1189.6192	2377.2470	1189.1271	22
22	2301.2337	1151.1205	2284.2071	1142.6072	2283.2231	1142.1152	N	2282.1735	1141.5904	2265.1470	1133.0771	2264.1629	1132.5851	21
23	2358.2551	1179.6312	2341.2286	1171.1179	2340.2446	1170.6259	G	2168.1306	1084.5689	2151.1040	1076.0557	2150.1200	1075.5636	20
24	2514.3562	1257.6818	2497.3297	1249.1685	2496.3457	1248.6765	R	2111.1091	1056.0582	2094.0826	1047.5449	2093.0986	1047.0529	19
25	2643.3988	1322.2030	2626.3723	1313.6898	2625.3883	1313.1978	E	1955.0080	978.0076	1937.9815	969.4944	1936.9974	969.0024	18
26	2772.4414	1386.7243	2755.4149	1378.2111	2754.4309	1377.7191	E	1825.9654	913.4863	1808.9389	904.9731	1807.9549	904.4811	17
27	2859.4734	1430.2404	2842.4469	1421.7271	2841.4629	1421.2351	S	1696.9228	848.9651	1679.8963	840.4518	1678.9123	839.9598	16
28	2956.5262	1478.7667	2939.4997	1470.2535	2938.5156	1469.7615	P	1609.8908	805.4490	1592.8642	796.9358	1591.8802	796.4438	15
29	3069.6103	1535.3088	3052.5837	1526.7955	3051.5997	1526.3035	L	1512.8380	756.9227	1495.8115	748.4094	1494.8275	747.9174	14
30	3182.6943	1591.8508	3165.6678	1583.3375	3164.6838	1582.8455	L	1399.7540	700.3806	1382.7274	691.8673	1381.7434	691.3753	13
31	3295.7784	1648.3928	3278.7519	1639.8796	3277.7678	1639.3876	I	***1286.6699***	643.8386	1269.6434	635.3253	1268.6593	634.8333	12
32	3352.7999	1676.9036	3335.7733	1668.3903	3334.7893	1667.8983	G	***1173.5858***	587.2966	1156.5593	578.7833	1155.5753	578.2913	11
33	3480.8584	1740.9329	3463.8319	1732.4196	3462.8479	1731.9276	Q	***1116.5644***	558.7858	1099.5378	550.2726	1098.5538	549.7805	10
34	3608.9170	1804.9621	3591.8905	1796.4489	3590.9065	1795.9569	Q	***988.5058***	494.7565	971.4793	486.2433	970.4952	485.7513	9
35	3695.9490	1848.4782	3678.9225	1839.9649	3677.9385	1839.4729	S	***860.4472***	430.7272	843.4207	422.2140	842.4367	421.7220	8
36	3796.9967	1899.0020	3779.9702	1890.4887	3778.9862	1889.9967	T	***773.4152***	387.2112	756.3886	378.6980	755.4046	378.2060	7
37	3896.0651	1948.5362	3879.0386	1940.0229	3878.0546	1939.5309	V	***672.3675***	336.6874	655.3410	328.1741	654.3570	327.6821	6
38	3983.0972	1992.0522	3966.0706	1983.5389	3965.0866	1983.0469	S	***573.2991***	287.1532	556.2726	278.6399	555.2885	278.1479	5
39	4098.1241	2049.5657	4081.0976	2041.0524	4080.1135	2040.5604	D	***486.2671***	243.6372	469.2405	235.1239	468.2565	234.6319	4
40	4197.1925	2099.0999	4180.1660	2090.5866	4179.1820	2090.0946	V	***371.2401***	186.1237	354.2136	177.6104			3
41	4294.2453	2147.6263	4277.2187	2139.1130	4276.2347	2138.6210	P	***272.1717***	136.5895	255.1452	128.0762			2
42							R	***175.1190***	88.0631	158.0924	79.5498			1

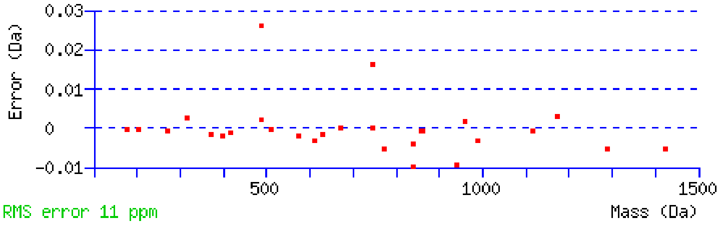

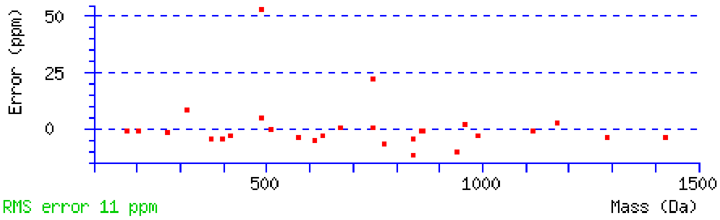

NCBI **BLAST** search of NSITLTNLTPGTEYVVSIVALNGREESPLLIGQQSTVSDVPR
(Parameters: blastp, nr protein database, expect=20000, no filter, PAM30)
Other BLAST web gateways

All matches to this query

Score	Mr(calc)	Delta	Sequence
80.8	4467.3497	0.0001	NSITLTNLTPGTEYVVSIVALNGREESPLLIGQQSTVSDVPR

Mascot: http://www.matrixscience.com/

Mascot Search Results

Peptide View

MS/MS Fragmentation of **NSITLTNLTPGTEYVVSIVALNGREESPLLIGQQSTVSDVPR**
Found in **P02751** in **uni_human_nr**, FINC_HUMAN Fibronectin OS=Homo sapiens GN=FN1 PE=1 SV=4

Match to Query 18278: 4468.337172 from(1490.453000,3+) intensity(333788.7813) rtinseconds(3647) scans(22038) index(16795)
Title: 130801_HPL_Human_Plaque_BR1_TR1_08_Spectrum038071_scans__22038_RTINSECONDS=3647
Data file E:\\RAW\\HumanPlaques\\mgf\\T\\1D\\T130801_HPL_Human_Plaque_BR1_TR1_08.mgf

Click mouse within plot area to zoom in by factor of two about that point
Or, Plot from 0 to 3400 Da Full range
Label all possible matches ○ Label matches used for scoring ⊙
Show Y-axis ☐

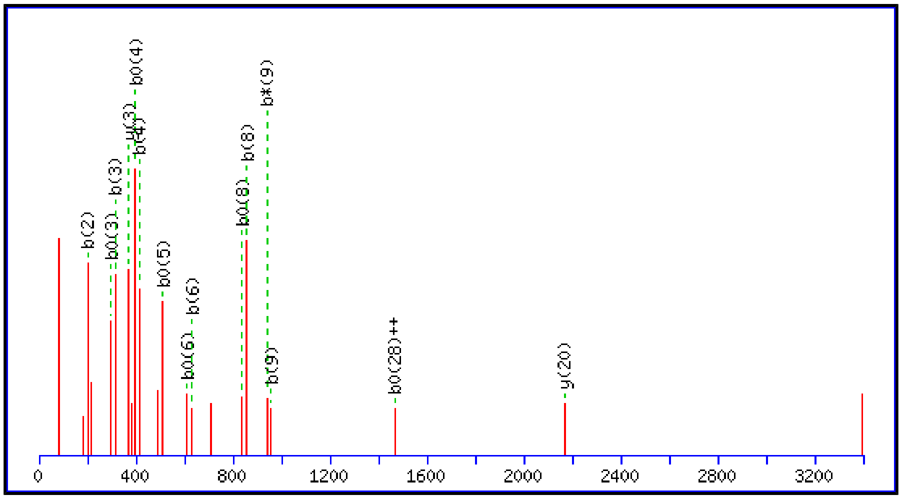

Monoisotopic mass of neutral peptide Mr(calc): 4467.3497
Fixed modifications: Carbamidomethyl (C) (apply to specified residues or termini only)
Ions Score: 30 **Expect:** 0.024
Matches : 15/484 fragment ions using 21 most intense peaks (help)

#	b	b^{++}	b*	b^{*++}	b^0	b^{0++}	Seq.	y	y^{++}	y*	y^{*++}	y^0	y^{0++}	#
1	115.0502	58.0287	98.0237	49.5155			N							42
2	***202.0822***	101.5448	185.0557	93.0315	184.0717	92.5395	S	4354.3140	2177.6607	4337.2875	2169.1474	4336.3035	2168.6554	41
3	***315.1663***	158.0868	298.1397	149.5735	297.1557	149.0815	I	4267.2820	2134.1446	4250.2555	2125.6314	4249.2714	2125.1394	40
4	***416.2140***	208.6106	399.1874	200.0974	398.2034	199.6053	T	4154.1979	2077.6026	4137.1714	2069.0893	4136.1874	2068.5973	39
5	529.2980	265.1527	512.2715	256.6394	511.2875	256.1474	L	4053.1503	2027.0788	4036.1237	2018.5655	4035.1397	2018.0735	38
6	***630.3457***	315.6765	613.3192	307.1632	612.3352	306.6712	T	3940.0662	1970.5367	3923.0397	1962.0235	3922.0556	1961.5315	37
7	744.3886	372.6980	727.3621	364.1847	726.3781	363.6927	N	3839.0185	1920.0129	3821.9920	1911.4996	3821.0080	1911.0076	36
8	***857.4727***	429.2400	840.4462	420.7267	839.4621	420.2347	L	3724.9756	1862.9914	3707.9490	1854.4782	3706.9650	1853.9862	35
9	***958.5204***	479.7638	941.4938	471.2506	940.5098	470.7585	T	3611.8915	1806.4494	3594.8650	1797.9361	3593.8810	1797.4441	34
10	1055.5732	528.2902	1038.5466	519.7769	1037.5626	519.2849	P	3510.8439	1755.9256	3493.8173	1747.4123	3492.8333	1746.9203	33
11	1112.5946	556.8009	1095.5681	548.2877	1094.5840	547.7957	G	3413.7911	1707.3992	3396.7645	1698.8859	3395.7805	1698.3939	32
12	1213.6423	607.3248	1196.6157	598.8115	1195.6317	598.3195	T	3356.7696	1678.8885	3339.7431	1670.3752	3338.7591	1669.8832	31
13	1342.6849	671.8461	1325.6583	663.3328	1324.6743	662.8408	E	3255.7219	1628.3646	3238.6954	1619.8513	3237.7114	1619.3593	30
14	1505.7482	753.3777	1488.7217	744.8645	1487.7377	744.3725	Y	3126.6794	1563.8433	3109.6528	1555.3300	3108.6688	1554.8380	29
15	1604.8166	802.9120	1587.7901	794.3987	1586.8061	793.9067	V	2963.6160	1482.3117	2946.5895	1473.7984	2945.6055	1473.3064	28
16	1703.8850	852.4462	1686.8585	843.9329	1685.8745	843.4409	V	2864.5476	1432.7774	2847.5211	1424.2642	2846.5370	1423.7722	27
17	1790.9171	895.9622	1773.8905	887.4489	1772.9065	886.9569	S	2765.4792	1383.2432	2748.4526	1374.7300	2747.4686	1374.2380	26
18	1904.0011	952.5042	1886.9746	943.9909	1885.9906	943.4989	I	2678.4472	1339.7272	2661.4206	1331.2139	2660.4366	1330.7219	25
19	2003.0696	1002.0384	1986.0430	993.5251	1985.0590	993.0331	V	2565.3631	1283.1852	2548.3366	1274.6719	2547.3525	1274.1799	24

20	2074.1067	1037.5570	2057.0801	1029.0437	2056.0961	1028.5517	A	2466.2947	1233.6510	2449.2681	1225.1377	2448.2841	1224.6457	23
21	2187.1907	1094.0990	2170.1642	1085.5857	2169.1802	1085.0937	L	2395.2576	1198.1324	2378.2310	1189.6192	2377.2470	1189.1271	22
22	2301.2337	1151.1205	2284.2071	1142.6072	2283.2231	1142.1152	N	2282.1735	1141.5904	2265.1470	1133.0771	2264.1629	1132.5851	21
23	2358.2551	1179.6312	2341.2286	1171.1179	2340.2446	1170.6259	G	**2168.1306**	1084.5689	2151.1040	1076.0557	2150.1200	1075.5636	20
24	2514.3562	1257.6818	2497.3297	1249.1685	2496.3457	1248.6765	R	2111.1091	1056.0582	2094.0826	1047.5449	2093.0986	1047.0529	19
25	2643.3988	1322.2030	2626.3723	1313.6898	2625.3883	1313.1978	E	1955.0080	978.0076	1937.9815	969.4944	1936.9974	969.0024	18
26	2772.4414	1386.7243	2755.4149	1378.2111	2754.4309	1377.7191	E	1825.9654	913.4863	1808.9389	904.9731	1807.9549	904.4811	17
27	2859.4734	1430.2404	2842.4469	1421.7271	2841.4629	1421.2351	S	1696.9228	848.9651	1679.8963	840.4518	1678.9123	839.9598	16
28	2956.5262	1478.7667	2939.4997	1470.2535	2938.5156	1469.7615	P	1609.8908	805.4490	1592.8642	796.9358	1591.8802	796.4438	15
29	3069.6103	1535.3088	3052.5837	1526.7955	3051.5997	1526.3035	L	1512.8380	756.9227	1495.8115	748.4094	1494.8275	747.9174	14
30	3182.6943	1591.8508	3165.6678	1583.3375	3164.6838	1582.8455	L	1399.7540	700.3806	1382.7274	691.8673	1381.7434	691.3753	13
31	3295.7784	1648.3928	3278.7519	1639.8796	3277.7678	1639.3876	I	1286.6699	643.8386	1269.6434	635.3253	1268.6593	634.8333	12
32	3352.7999	1676.9036	3335.7733	1668.3903	3334.7893	1667.8983	G	1173.5858	587.2966	1156.5593	578.7833	1155.5753	578.2913	11
33	3480.8584	1740.9329	3463.8319	1732.4196	3462.8479	1731.9276	Q	1116.5644	558.7858	1099.5378	550.2726	1098.5538	549.7805	10
34	3608.9170	1804.9621	3591.8905	1796.4489	3590.9065	1795.9569	Q	988.5058	494.7565	971.4793	486.2433	970.4952	485.7513	9
35	3695.9490	1848.4782	3678.9225	1839.9649	3677.9385	1839.4729	S	860.4472	430.7272	843.4207	422.2140	842.4367	421.7220	8
36	3796.9967	1899.0020	3779.9702	1890.4887	3778.9862	1889.9967	T	773.4152	387.2112	756.3886	378.6980	755.4046	378.2060	7
37	3896.0651	1948.5362	3879.0386	1940.0229	3878.0546	1939.5309	V	672.3675	336.6874	655.3410	328.1741	654.3570	327.6821	6
38	3983.0972	1992.0522	3966.0706	1983.5389	3965.0866	1983.0469	S	573.2991	287.1532	556.2726	278.6399	555.2885	278.1479	5
39	4098.1241	2049.5657	4081.0976	2041.0524	4080.1135	2040.5604	D	486.2671	243.6372	469.2405	235.1239	468.2565	234.6319	4
40	4197.1925	2099.0999	4180.1660	2090.5866	4179.1820	2090.0946	V	**371.2401**	186.1237	354.2136	177.6104			3
41	4294.2453	2147.6263	4277.2187	2139.1130	4276.2347	2138.6210	P	272.1717	136.5895	255.1452	128.0762			2
42							R	175.1190	88.0631	158.0924	79.5498			1

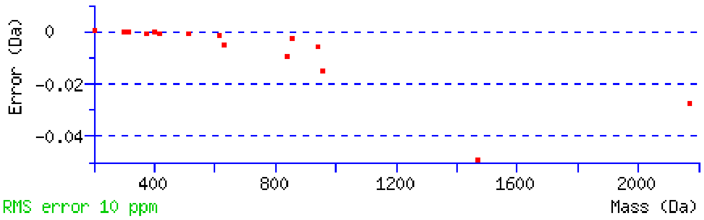

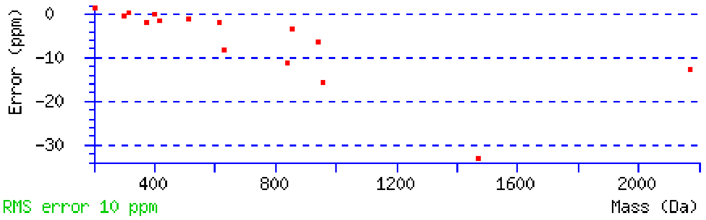

NCBI **BLAST** search of NSITLTNLTPGTEYVVSIVALNGREESPLLIGQQSTVSDVPR
(Parameters: blastp, nr protein database, expect=20000, no filter, PAM30)
Other BLAST web gateways

All matches to this query

Score	Mr(calc)	Delta	Sequence
30.5	4467.3497	0.9875	NSITLTNLTPGTEYVVSIVALNGREESPLLIGQQSTVSDVPR
28.6	4468.3337	0.0035	NSITLTNLTPGTEYVVSIVALNGREESPLLIGQQSTVSDVPR
28.6	4468.3337	0.0035	NSITLTNLTPGTEYVVSIVALNGREESPLLIGQQSTVSDVPR
28.6	4468.3337	0.0035	NSITLTNLTPGTEYVVSIVALNGREESPLLIGQQSTVSDVPR
12.6	4468.3337	0.0035	NSITLTNLTPGTEYVVSIVALNGREESPLLIGQQSTVSDVPR

Mascot: http://www.matrixscience.com/

Mascot Search Results

Peptide View

MS/MS Fragmentation of **QMLCTCLGNGVSCQETAVTQTYGGNSNGEPCVLPFTYNGR**
Found in **P02751** in **uni_human_nr**, FINC_HUMAN Fibronectin OS=Homo sapiens GN=FN1 PE=1 SV=4

Match to Query 17469: 4456.953162 from(1486.658330,3+) intensity(198830.7813) rtinseconds(3518) scans(20619) index(15546)
Title: 130809_HPL_Human_Plaque_BR1_TR3_15_Spectrum035933_scans__20619_RTINSECONDS=3518
Data file E:\\RAW\\HumanPlaques\\mgf\\T\\1D\\T130809_HPL_Human_Plaque_BR1_TR3_15.mgf

Click mouse within plot area to zoom in by factor of two about that point
Or, Plot from 0 to 1400 Da Full range
Label all possible matches ○ Label matches used for scoring ⊙
Show Y-axis ☐

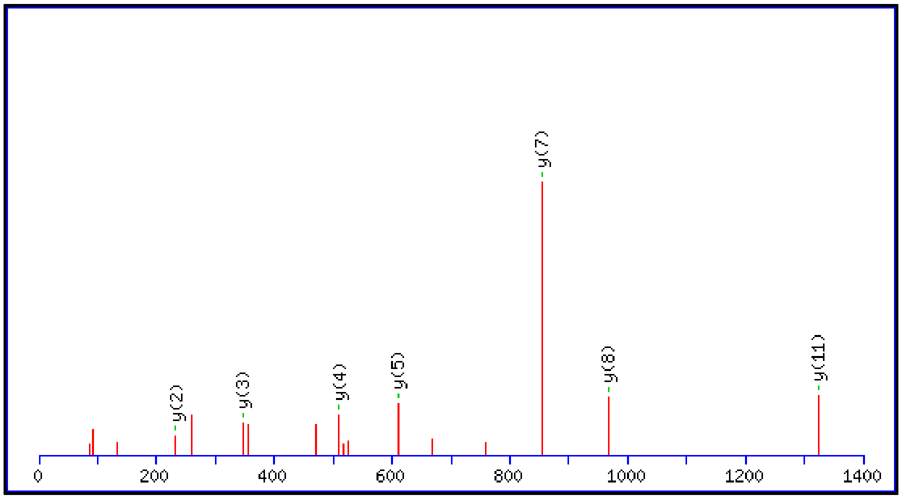

Monoisotopic mass of neutral peptide Mr(calc): 4455.9287
Fixed modifications: Carbamidomethyl (C) (apply to specified residues or termini only)
Variable modifications:
M2 : Oxidation (M), with neutral losses 0.0000(shown in table), 63.9983
Ions Score: 32 **Expect:** 0.015
Matches : 7/680 fragment ions using 16 most intense peaks (help)

#	b	b++	b*	b*++	b0	b0++	Seq.	y	y++	y*	y*++	y0	y0++	#
1	129.0659	65.0366	112.0393	56.5233			Q							40
2	276.1013	138.5543	259.0747	130.0410			M	4328.8774	2164.9423	4311.8508	2156.4291	4310.8668	2155.9370	39
3	389.1853	195.0963	372.1588	186.5830			L	4181.8420	2091.4246	4164.8154	2082.9114	4163.8314	2082.4193	38
4	549.2160	275.1116	532.1894	266.5983			C	4068.7579	2034.8826	4051.7314	2026.3693	4050.7474	2025.8773	37
5	650.2636	325.6355	633.2371	317.1222	632.2531	316.6302	T	3908.7273	1954.8673	3891.7007	1946.3540	3890.7167	1945.8620	36
6	810.2943	405.6508	793.2677	397.1375	792.2837	396.6455	C	3807.6796	1904.3434	3790.6530	1895.8302	3789.6690	1895.3381	35
7	923.3784	462.1928	906.3518	453.6795	905.3678	453.1875	L	3647.6489	1824.3281	3630.6224	1815.8148	3629.6384	1815.3228	34
8	980.3998	490.7035	963.3733	482.1903	962.3893	481.6983	G	3534.5649	1767.7861	3517.5383	1759.2728	3516.5543	1758.7808	33
9	1094.4428	547.7250	1077.4162	539.2117	1076.4322	538.7197	N	3477.5434	1739.2753	3460.5169	1730.7621	3459.5328	1730.2701	32
10	1151.4642	576.2357	1134.4377	567.7225	1133.4536	567.2305	G	3363.5005	1682.2539	3346.4739	1673.7406	3345.4899	1673.2486	31
11	1250.5326	625.7700	1233.5061	617.2567	1232.5221	616.7647	V	3306.4790	1653.7431	3289.4525	1645.2299	3288.4685	1644.7379	30
12	1337.5647	669.2860	1320.5381	660.7727	1319.5541	660.2807	S	3207.4106	1604.2089	3190.3841	1595.6957	3189.4000	1595.2037	29
13	1497.5953	749.3013	1480.5688	740.7880	1479.5847	740.2960	C	3120.3786	1560.6929	3103.3520	1552.1797	3102.3680	1551.6876	28
14	1625.6539	813.3306	1608.6273	804.8173	1607.6433	804.3253	Q	2960.3479	1480.6776	2943.3214	1472.1643	2942.3374	1471.6723	27
15	1754.6965	877.8519	1737.6699	869.3386	1736.6859	868.8466	E	2832.2894	1416.6483	2815.2628	1408.1350	2814.2788	1407.6430	26
16	1855.7442	928.3757	1838.7176	919.8624	1837.7336	919.3704	T	2703.2468	1352.1270	2686.2202	1343.6137	2685.2362	1343.1217	25
17	1926.7813	963.8943	1909.7547	955.3810	1908.7707	954.8890	A	2602.1991	1301.6032	2585.1725	1293.0899	2584.1885	1292.5979	24
18	2025.8497	1013.4285	2008.8231	1004.9152	2007.8391	1004.4232	V	2531.1620	1266.0846	2514.1354	1257.5713	2513.1514	1257.0793	23

**19**	2126.8974	1063.9523	2109.8708	1055.4390	2108.8868	1054.9470	**T**	2432.0936	1216.5504	2415.0670	1208.0371	2414.0830	1207.5451	**22**
**20**	2254.9559	1127.9816	2237.9294	1119.4683	2236.9454	1118.9763	**Q**	2331.0459	1166.0266	2314.0193	1157.5133	2313.0353	1157.0213	**21**
**21**	2356.0036	1178.5054	2338.9771	1169.9922	2337.9931	1169.5002	**T**	2202.9873	1101.9973	2185.9607	1093.4840	2184.9767	1092.9920	**20**
**22**	2519.0669	1260.0371	2502.0404	1251.5238	2501.0564	1251.0318	**Y**	2101.9396	1051.4734	2084.9131	1042.9602	2083.9291	1042.4682	**19**
**23**	2576.0884	1288.5478	2559.0619	1280.0346	2558.0778	1279.5426	**G**	1938.8763	969.9418	1921.8497	961.4285	1920.8657	960.9365	**18**
**24**	2633.1099	1317.0586	2616.0833	1308.5453	2615.0993	1308.0533	**G**	1881.8548	941.4310	1864.8283	932.9178	1863.8443	932.4258	**17**
**25**	2747.1528	1374.0800	2730.1263	1365.5668	2729.1422	1365.0748	**N**	1824.8334	912.9203	1807.8068	904.4070	1806.8228	903.9150	**16**
**26**	2834.1848	1417.5961	2817.1583	1409.0828	2816.1743	1408.5908	**S**	1710.7904	855.8989	1693.7639	847.3856	1692.7799	846.8936	**15**
**27**	2948.2278	1474.6175	2931.2012	1466.1042	2930.2172	1465.6122	**N**	1623.7584	812.3828	1606.7319	803.8696	1605.7478	803.3776	**14**
**28**	3005.2492	1503.1282	2988.2227	1494.6150	2987.2387	1494.1230	**G**	1509.7155	755.3614	1492.6889	746.8481	1491.7049	746.3561	**13**
**29**	3134.2918	1567.6495	3117.2653	1559.1363	3116.2813	1558.6443	**E**	1452.6940	726.8506	1435.6675	718.3374	1434.6834	717.8454	**12**
**30**	3231.3446	1616.1759	3214.3180	1607.6627	3213.3340	1607.1706	**P**	***1323.6514***	662.3293	1306.6249	653.8161	1305.6409	653.3241	**11**
**31**	3391.3752	1696.1913	3374.3487	1687.6780	3373.3647	1687.1860	**C**	1226.5987	613.8030	1209.5721	605.2897	1208.5881	604.7977	**10**
**32**	3490.4436	1745.7255	3473.4171	1737.2122	3472.4331	1736.7202	**V**	1066.5680	533.7876	1049.5415	525.2744	1048.5574	524.7824	**9**
**33**	3603.5277	1802.2675	3586.5012	1793.7542	3585.5171	1793.2622	**L**	***967.4996***	484.2534	950.4730	475.7402	949.4890	475.2482	**8**
**34**	3700.5805	1850.7939	3683.5539	1842.2806	3682.5699	1841.7886	**P**	***854.4155***	427.7114	837.3890	419.1981	836.4050	418.7061	**7**
**35**	3847.6489	1924.3281	3830.6223	1915.8148	3829.6383	1915.3228	**F**	757.3628	379.1850	740.3362	370.6717	739.3522	370.1797	**6**
**36**	3948.6966	1974.8519	3931.6700	1966.3386	3930.6860	1965.8466	**T**	***610.2944***	305.6508	593.2678	297.1375	592.2838	296.6455	**5**
**37**	4111.7599	2056.3836	4094.7333	2047.8703	4093.7493	2047.3783	**Y**	***509.2467***	255.1270	492.2201	246.6137			**4**
**38**	4225.8028	2113.4050	4208.7763	2104.8918	4207.7923	2104.3998	**N**	***346.1833***	173.5953	329.1568	165.0820			**3**
**39**	4282.8243	2141.9158	4265.7977	2133.4025	4264.8137	2132.9105	**G**	***232.1404***	116.5738	215.1139	108.0606			**2**
**40**							**R**	175.1190	88.0631	158.0924	79.5498			**1**

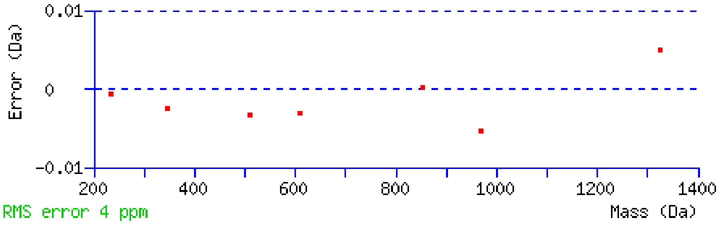

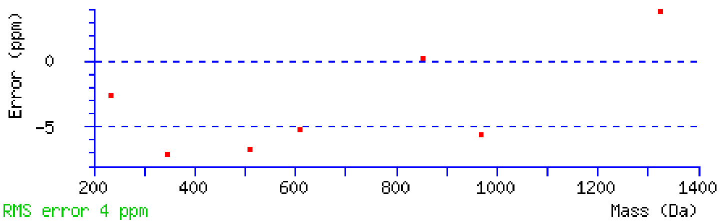

NCBI **BLAST** search of QMLCTCLGNGVSCQETAVTQTYGGNSNGEPCVLPFTYNGR
(Parameters: blastp, nr protein database, expect=20000, no filter, PAM30)
Other BLAST web gateways

All matches to this query

Score	Mr(calc)	Delta	Sequence
32.0	4455.9287	1.0244	QMLCTCLGNGVSCQETAVTQTYGGNSNGEPCVLPFTYNGR

Mascot: http://www.matrixscience.com/

Mascot Search Results

Peptide View

MS/MS Fragmentation of **QMLCTCLGNGVSCQETAVTQTYGGNSNGEPCVLPFTYNGR**
Found in **P02751** in **uni_human_nr**, FINC_HUMAN Fibronectin OS=Homo sapiens GN=FN1 PE=1 SV=4

Match to Query 18657: 4456.954776 from(1115.245970,4+) intensity(609333.8125) rtinseconds(3063) scans(18388) index(14031)
Title: 130809_HPL_Human_Plaque_BR1_TR3_08_Spectrum035641_scans__18388_RTINSECONDS=3063
Data file E:\\RAW\\HumanPlaques\\mgf\\T\\1D\\T130809_HPL_Human_Plaque_BR1_TR3_08.mgf

Click mouse within plot area to zoom in by factor of two about that point
Or, Plot from 0 to 1900 Da Full range
Label all possible matches ○ Label matches used for scoring ⊙
Show Y-axis ☐

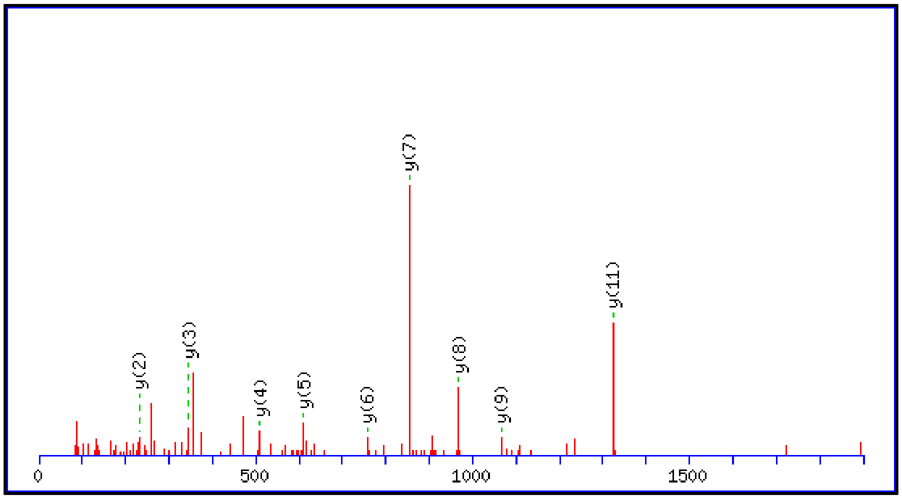

Monoisotopic mass of neutral peptide Mr(calc): 4455.9287
Fixed modifications: Carbamidomethyl (C) (apply to specified residues or termini only)
Variable modifications:
M2 : Oxidation (M), with neutral losses 0.0000(shown in table), 63.9983
Ions Score: 32 **Expect:** 0.015
Matches : 9/680 fragment ions using 28 most intense peaks (help)

#	b	b^{++}	b*	b^{*++}	b^0	b^{0++}	Seq.	y	y^{++}	y*	y^{*++}	y^0	y^{0++}	#
1	129.0659	65.0366	112.0393	56.5233			Q							40
2	276.1013	138.5543	259.0747	130.0410			M	4328.8774	2164.9423	4311.8508	2156.4291	4310.8668	2155.9370	39
3	389.1853	195.0963	372.1588	186.5830			L	4181.8420	2091.4246	4164.8154	2082.9114	4163.8314	2082.4193	38
4	549.2160	275.1116	532.1894	266.5983			C	4068.7579	2034.8826	4051.7314	2026.3693	4050.7474	2025.8773	37
5	650.2636	325.6355	633.2371	317.1222	632.2531	316.6302	T	3908.7273	1954.8673	3891.7007	1946.3540	3890.7167	1945.8620	36
6	810.2943	405.6508	793.2677	397.1375	792.2837	396.6455	C	3807.6796	1904.3434	3790.6530	1895.8302	3789.6690	1895.3381	35
7	923.3784	462.1928	906.3518	453.6795	905.3678	453.1875	L	3647.6489	1824.3281	3630.6224	1815.8148	3629.6384	1815.3228	34
8	980.3998	490.7035	963.3733	482.1903	962.3893	481.6983	G	3534.5649	1767.7861	3517.5383	1759.2728	3516.5543	1758.7808	33
9	1094.4428	547.7250	1077.4162	539.2117	1076.4322	538.7197	N	3477.5434	1739.2753	3460.5169	1730.7621	3459.5328	1730.2701	32
10	1151.4642	576.2357	1134.4377	567.7225	1133.4536	567.2305	G	3363.5005	1682.2539	3346.4739	1673.7406	3345.4899	1673.2486	31
11	1250.5326	625.7700	1233.5061	617.2567	1232.5221	616.7647	V	3306.4790	1653.7431	3289.4525	1645.2299	3288.4685	1644.7379	30
12	1337.5647	669.2860	1320.5381	660.7727	1319.5541	660.2807	S	3207.4106	1604.2089	3190.3841	1595.6957	3189.4000	1595.2037	29
13	1497.5953	749.3013	1480.5688	740.7880	1479.5847	740.2960	C	3120.3786	1560.6929	3103.3520	1552.1797	3102.3680	1551.6876	28
14	1625.6539	813.3306	1608.6273	804.8173	1607.6433	804.3253	Q	2960.3479	1480.6776	2943.3214	1472.1643	2942.3374	1471.6723	27
15	1754.6965	877.8519	1737.6699	869.3386	1736.6859	868.8466	E	2832.2894	1416.6483	2815.2628	1408.1350	2814.2788	1407.6430	26
16	1855.7442	928.3757	1838.7176	919.8624	1837.7336	919.3704	T	2703.2468	1352.1270	2686.2202	1343.6137	2685.2362	1343.1217	25
17	1926.7813	963.8943	1909.7547	955.3810	1908.7707	954.8890	A	2602.1991	1301.6032	2585.1725	1293.0899	2584.1885	1292.5979	24
18	2025.8497	1013.4285	2008.8231	1004.9152	2007.8391	1004.4232	V	2531.1620	1266.0846	2514.1354	1257.5713	2513.1514	1257.0793	23

**19**	2126.8974	1063.9523	2109.8708	1055.4390	2108.8868	1054.9470	**T**	2432.0936	1216.5504	2415.0670	1208.0371	2414.0830	1207.5451	**22**
**20**	2254.9559	1127.9816	2237.9294	1119.4683	2236.9454	1118.9763	**Q**	2331.0459	1166.0266	2314.0193	1157.5133	2313.0353	1157.0213	**21**
**21**	2356.0036	1178.5054	2338.9771	1169.9922	2337.9931	1169.5002	**T**	2202.9873	1101.9973	2185.9607	1093.4840	2184.9767	1092.9920	**20**
**22**	2519.0669	1260.0371	2502.0404	1251.5238	2501.0564	1251.0318	**Y**	2101.9396	1051.4734	2084.9131	1042.9602	2083.9291	1042.4682	**19**
**23**	2576.0884	1288.5478	2559.0619	1280.0346	2558.0778	1279.5426	**G**	1938.8763	969.9418	1921.8497	961.4285	1920.8657	960.9365	**18**
**24**	2633.1099	1317.0586	2616.0833	1308.5453	2615.0993	1308.0533	**G**	1881.8548	941.4310	1864.8283	932.9178	1863.8443	932.4258	**17**
**25**	2747.1528	1374.0800	2730.1263	1365.5668	2729.1422	1365.0748	**N**	1824.8334	912.9203	1807.8068	904.4070	1806.8228	903.9150	**16**
**26**	2834.1848	1417.5961	2817.1583	1409.0828	2816.1743	1408.5908	**S**	1710.7904	855.8989	1693.7639	847.3856	1692.7799	846.8936	**15**
**27**	2948.2278	1474.6175	2931.2012	1466.1042	2930.2172	1465.6122	**N**	1623.7584	812.3828	1606.7319	803.8696	1605.7478	803.3776	**14**
**28**	3005.2492	1503.1282	2988.2227	1494.6150	2987.2387	1494.1230	**G**	1509.7155	755.3614	1492.6889	746.8481	1491.7049	746.3561	**13**
**29**	3134.2918	1567.6495	3117.2653	1559.1363	3116.2813	1558.6443	**E**	1452.6940	726.8506	1435.6675	718.3374	1434.6834	717.8454	**12**
**30**	3231.3446	1616.1759	3214.3180	1607.6627	3213.3340	1607.1706	**P**	***1323.6514***	662.3293	1306.6249	653.8161	1305.6409	653.3241	**11**
**31**	3391.3752	1696.1913	3374.3487	1687.6780	3373.3647	1687.1860	**C**	1226.5987	613.8030	1209.5721	605.2897	1208.5881	604.7977	**10**
**32**	3490.4436	1745.7255	3473.4171	1737.2122	3472.4331	1736.7202	**V**	***1066.5680***	533.7876	1049.5415	525.2744	1048.5574	524.7824	**9**
**33**	3603.5277	1802.2675	3586.5012	1793.7542	3585.5171	1793.2622	**L**	***967.4996***	484.2534	950.4730	475.7402	949.4890	475.2482	**8**
**34**	3700.5805	1850.7939	3683.5539	1842.2806	3682.5699	1841.7886	**P**	***854.4155***	427.7114	837.3890	419.1981	836.4050	418.7061	**7**
**35**	3847.6489	1924.3281	3830.6223	1915.8148	3829.6383	1915.3228	**F**	***757.3628***	379.1850	740.3362	370.6717	739.3522	370.1797	**6**
**36**	3948.6966	1974.8519	3931.6700	1966.3386	3930.6860	1965.8466	**T**	***610.2944***	305.6508	593.2678	297.1375	592.2838	296.6455	**5**
**37**	4111.7599	2056.3836	4094.7333	2047.8703	4093.7493	2047.3783	**Y**	***509.2467***	255.1270	492.2201	246.6137			**4**
**38**	4225.8028	2113.4050	4208.7763	2104.8918	4207.7923	2104.3998	**N**	***346.1833***	173.5953	329.1568	165.0820			**3**
**39**	4282.8243	2141.9158	4265.7977	2133.4025	4264.8137	2132.9105	**G**	***232.1404***	116.5738	215.1139	108.0606			**2**
**40**							**R**	175.1190	88.0631	158.0924	79.5498			**1**

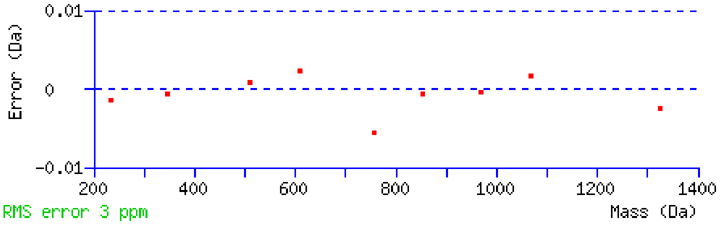

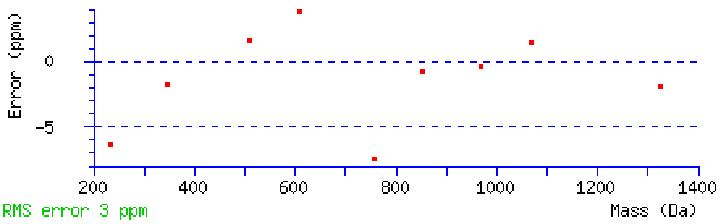

NCBI **BLAST** search of QMLCTCLGNGVSCQETAVTQTYGGNSNGEPCVLPFTYNGR
(Parameters: blastp, nr protein database, expect=20000, no filter, PAM30)
Other BLAST web gateways

All matches to this query

Score	Mr(calc)	Delta	Sequence
31.9	4455.9287	1.0260	QMLCTCLGNGVSCQETAVTQTYGGNSNGEPCVLPFTYNGR

Mascot: http://www.matrixscience.com/

Mascot Search Results

Peptide View

MS/MS Fragmentation of **QMLCTCLGNGVSCQETAVTQTYGGNSNGEPCVLPFTYNGR**
Found in **P02751** in **uni_human_nr**, FINC_HUMAN Fibronectin OS=Homo sapiens GN=FN1 PE=1 SV=4

Match to Query 17805: 4440.911292 from(1481.311040,3+) intensity(210279.1406) rtinseconds(3097) scans(18317) index(13958)
Title: 130806_HPL_Human_Plaque_BR1_TR2_11_Spectrum034994_scans__18317_RTINSECONDS=3097
Data file E:\\RAW\\HumanPlaques\\mgf\\T\\1D\\T130806_HPL_Human_Plaque_BR1_TR2_11.mgf

Click mouse within plot area to zoom in by factor of two about that point
Or, Plot from 0 to 1400 Da Full range
Label all possible matches ○ Label matches used for scoring ⊙
Show Y-axis ☐

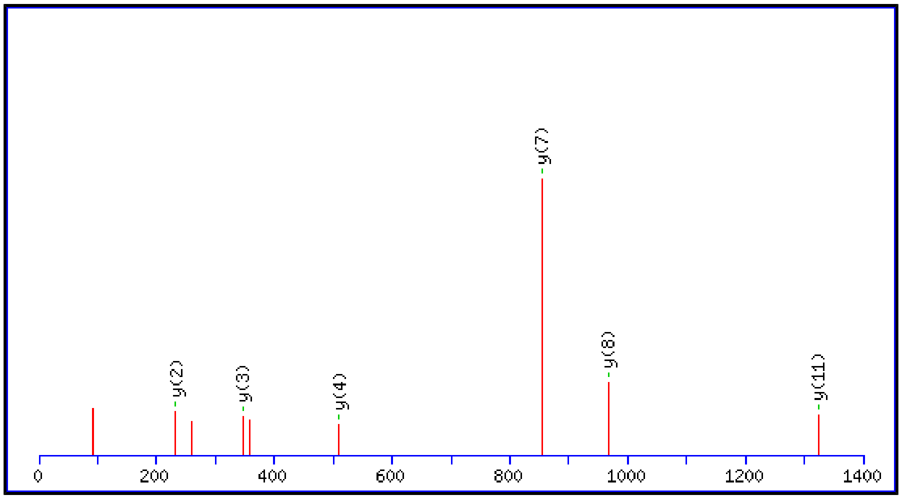

Monoisotopic mass of neutral peptide Mr(calc): 4440.9178
Fixed modifications: Carbamidomethyl (C) (apply to specified residues or termini only)
Variable modifications:
N27 : Deamidated (NQ)
Ions Score: 42 **Expect:** 0.001
Matches : 6/452 fragment ions using 7 most intense peaks (help)

#	b	b^{++}	b*	b^{*++}	b^0	b^{0++}	Seq.	y	y^{++}	y*	y^{*++}	y^0	y^{0++}	#
1	129.0659	65.0366	112.0393	56.5233			Q							40
2	260.1063	130.5568	243.0798	122.0435			M	4313.8665	2157.4369	4296.8399	2148.9236	4295.8559	2148.4316	39
3	373.1904	187.0988	356.1639	178.5856			L	4182.8260	2091.9166	4165.7994	2083.4034	4164.8154	2082.9114	38
4	533.2211	267.1142	516.1945	258.6009			C	4069.7419	2035.3746	4052.7154	2026.8613	4051.7314	2026.3693	37
5	634.2687	317.6380	617.2422	309.1247	616.2582	308.6327	T	3909.7113	1955.3593	3892.6847	1946.8460	3891.7007	1946.3540	36
6	794.2994	397.6533	777.2728	389.1401	776.2888	388.6480	C	3808.6636	1904.8354	3791.6371	1896.3222	3790.6530	1895.8302	35
7	907.3834	454.1954	890.3569	445.6821	889.3729	445.1901	L	3648.6330	1824.8201	3631.6064	1816.3068	3630.6224	1815.8148	34
8	964.4049	482.7061	947.3784	474.1928	946.3943	473.7008	G	3535.5489	1768.2781	3518.5223	1759.7648	3517.5383	1759.2728	33
9	1078.4478	539.7276	1061.4213	531.2143	1060.4373	530.7223	N	3478.5274	1739.7674	3461.5009	1731.2541	3460.5169	1730.7621	32
10	1135.4693	568.2383	1118.4428	559.7250	1117.4587	559.2330	G	3364.4845	1682.7459	3347.4580	1674.2326	3346.4739	1673.7406	31
11	1234.5377	617.7725	1217.5112	609.2592	1216.5271	608.7672	V	3307.4630	1654.2352	3290.4365	1645.7219	3289.4525	1645.2299	30
12	1321.5697	661.2885	1304.5432	652.7752	1303.5592	652.2832	S	3208.3946	1604.7009	3191.3681	1596.1877	3190.3841	1595.6957	29
13	1481.6004	741.3038	1464.5738	732.7906	1463.5898	732.2986	C	3121.3626	1561.1849	3104.3360	1552.6717	3103.3520	1552.1797	28
14	1609.6590	805.3331	1592.6324	796.8198	1591.6484	796.3278	Q	2961.3319	1481.1696	2944.3054	1472.6563	2943.3214	1472.1643	27
15	1738.7016	869.8544	1721.6750	861.3411	1720.6910	860.8491	E	2833.2734	1417.1403	2816.2468	1408.6270	2815.2628	1408.1350	26
16	1839.7492	920.3783	1822.7227	911.8650	1821.7387	911.3730	T	2704.2308	1352.6190	2687.2042	1344.1058	2686.2202	1343.6137	25
17	1910.7864	955.8968	1893.7598	947.3835	1892.7758	946.8915	A	2603.1831	1302.0952	2586.1565	1293.5819	2585.1725	1293.0899	24
18	2009.8548	1005.4310	1992.8282	996.9177	1991.8442	996.4257	V	2532.1460	1266.5766	2515.1194	1258.0634	2514.1354	1257.5713	23

**19**	2110.9024	1055.9549	2093.8759	1047.4416	2092.8919	1046.9496	**T**	2433.0776	1217.0424	2416.0510	1208.5291	2415.0670	1208.0371	**22**
**20**	2238.9610	1119.9842	2221.9345	1111.4709	2220.9505	1110.9789	**Q**	2332.0299	1166.5186	2315.0033	1158.0053	2314.0193	1157.5133	**21**
**21**	2340.0087	1170.5080	2322.9822	1161.9947	2321.9981	1161.5027	**T**	2203.9713	1102.4893	2186.9448	1093.9760	2185.9607	1093.4840	**20**
**22**	2503.0720	1252.0397	2486.0455	1243.5264	2485.0615	1243.0344	**Y**	2102.9236	1051.9655	2085.8971	1043.4522	2084.9131	1042.9602	**19**
**23**	2560.0935	1280.5504	2543.0669	1272.0371	2542.0829	1271.5451	**G**	1939.8603	970.4338	1922.8338	961.9205	1921.8497	961.4285	**18**
**24**	2617.1150	1309.0611	2600.0884	1300.5478	2599.1044	1300.0558	**G**	1882.8388	941.9231	1865.8123	933.4098	1864.8283	932.9178	**17**
**25**	2731.1579	1366.0826	2714.1313	1357.5693	2713.1473	1357.0773	**N**	1825.8174	913.4123	1808.7908	904.8991	1807.8068	904.4070	**16**
**26**	2818.1899	1409.5986	2801.1634	1401.0853	2800.1794	1400.5933	**S**	1711.7744	856.3909	1694.7479	847.8776	1693.7639	847.3856	**15**
**27**	2933.2169	1467.1121	2916.1903	1458.5988	2915.2063	1458.1068	**N**	1624.7424	812.8748	1607.7159	804.3616	1606.7319	803.8696	**14**
**28**	2990.2383	1495.6228	2973.2118	1487.1095	2972.2278	1486.6175	**G**	1509.7155	755.3614	1492.6889	746.8481	1491.7049	746.3561	**13**
**29**	3119.2809	1560.1441	3102.2544	1551.6308	3101.2704	1551.1388	**E**	1452.6940	726.8506	1435.6675	718.3374	1434.6834	717.8454	**12**
**30**	3216.3337	1608.6705	3199.3071	1600.1572	3198.3231	1599.6652	**P**	***1323.6514***	662.3293	1306.6249	653.8161	1305.6409	653.3241	**11**
**31**	3376.3643	1688.6858	3359.3378	1680.1725	3358.3538	1679.6805	**C**	1226.5987	613.8030	1209.5721	605.2897	1208.5881	604.7977	**10**
**32**	3475.4327	1738.2200	3458.4062	1729.7067	3457.4222	1729.2147	**V**	1066.5680	533.7876	1049.5415	525.2744	1048.5574	524.7824	**9**
**33**	3588.5168	1794.7620	3571.4903	1786.2488	3570.5062	1785.7568	**L**	***967.4996***	484.2534	950.4730	475.7402	949.4890	475.2482	**8**
**34**	3685.5696	1843.2884	3668.5430	1834.7751	3667.5590	1834.2831	**P**	***854.4155***	427.7114	837.3890	419.1981	836.4050	418.7061	**7**
**35**	3832.6380	1916.8226	3815.6114	1908.3094	3814.6274	1907.8173	**F**	757.3628	379.1850	740.3362	370.6717	739.3522	370.1797	**6**
**36**	3933.6857	1967.3465	3916.6591	1958.8332	3915.6751	1958.3412	**T**	610.2944	305.6508	593.2678	297.1375	592.2838	296.6455	**5**
**37**	4096.7490	2048.8781	4079.7224	2040.3649	4078.7384	2039.8729	**Y**	***509.2467***	255.1270	492.2201	246.6137			**4**
**38**	4210.7919	2105.8996	4193.7654	2097.3863	4192.7814	2096.8943	**N**	***346.1833***	173.5953	329.1568	165.0820			**3**
**39**	4267.8134	2134.4103	4250.7868	2125.8971	4249.8028	2125.4050	**G**	***232.1404***	116.5738	215.1139	108.0606			**2**
**40**							**R**	175.1190	88.0631	158.0924	79.5498			**1**

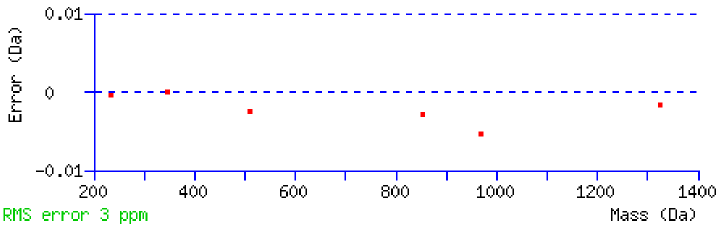

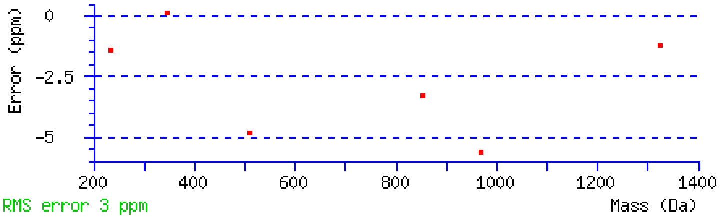

NCBI **BLAST** search of QMLCTCLGNGVSCQETAVTQTYGGNSNGEPCVLPFTYNGR
(Parameters: blastp, nr protein database, expect=20000, no filter, PAM30)
Other BLAST web gateways

All matches to this query

Score	Mr(calc)	Delta	Sequence	Site Analysis
42.4	4440.9178	-0.0065	QMLCTCLGNGVSCQETAVTQTYGGNSNGEPCVLPFTYNGR	Deamidated N27 14.72%
42.4	4440.9178	-0.0065	QMLCTCLGNGVSCQETAVTQTYGGNSNGEPCVLPFTYNGR	Deamidated N25 14.72%
42.4	4440.9178	-0.0065	QMLCTCLGNGVSCQETAVTQTYGGNSNGEPCVLPFTYNGR	Deamidated Q20 14.72%
42.4	4440.9178	-0.0065	QMLCTCLGNGVSCQETAVTQTYGGNSNGEPCVLPFTYNGR	Deamidated Q14 14.72%
42.4	4440.9178	-0.0065	QMLCTCLGNGVSCQETAVTQTYGGNSNGEPCVLPFTYNGR	Deamidated N9 14.72%
42.4	4440.9178	-0.0065	QMLCTCLGNGVSCQETAVTQTYGGNSNGEPCVLPFTYNGR	Deamidated Q1 14.72%

Mascot: http://www.matrixscience.com/

Mascot Search Results

Peptide View

MS/MS Fragmentation of **QMLCTCLGNGVSCQETAVTQTYGGNSNGEPCVLPFTYNGR**
Found in **P02751** in **uni_human_nr**, FINC_HUMAN Fibronectin OS=Homo sapiens GN=FN1 PE=1 SV=4

Match to Query 17377: 4440.925182 from(1481.315670,3+) intensity(225400.6563) rtinseconds(3107) scans(17964) index(13644)
Title: 130801_HPL_Human_Plaque_BR2_TR1_12_Spectrum034122_scans__17964_RTINSECONDS=3107
Data file E:\\RAW\\HumanPlaques\\mgf\\T\\1D\\T130801_HPL_Human_Plaque_BR2_TR1_12.mgf

Click mouse within plot area to zoom in by factor of two about that point
Or, Plot from 200 to 1000 Da Full range
Label all possible matches ○ Label matches used for scoring ⊙
Show Y-axis ☐

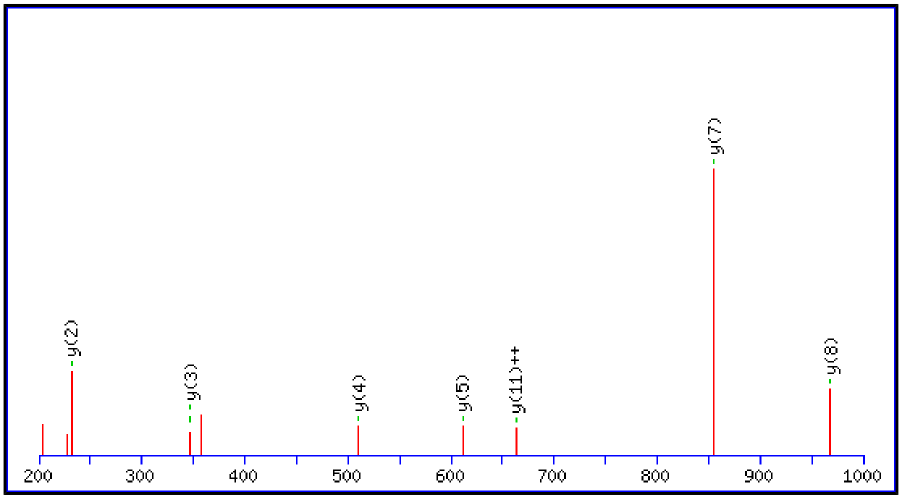

Monoisotopic mass of neutral peptide Mr(calc): 4440.9178
Fixed modifications: Carbamidomethyl (C) (apply to specified residues or termini only)
Variable modifications:
N38 : Deamidated (NQ)
Ions Score: 39 **Expect:** 0.0028
Matches : 7/452 fragment ions using 9 most intense peaks (help)

#	b	b^{++}	b*	b^{*++}	b^0	b^{0++}	Seq.	y	y^{++}	y*	y^{*++}	y^0	y^{0++}	#
1	129.0659	65.0366	112.0393	56.5233			Q							40
2	260.1063	130.5568	243.0798	122.0435			M	4313.8665	2157.4369	4296.8399	2148.9236	4295.8559	2148.4316	39
3	373.1904	187.0988	356.1639	178.5856			L	4182.8260	2091.9166	4165.7994	2083.4034	4164.8154	2082.9114	38
4	533.2211	267.1142	516.1945	258.6009			C	4069.7419	2035.3746	4052.7154	2026.8613	4051.7314	2026.3693	37
5	634.2687	317.6380	617.2422	309.1247	616.2582	308.6327	T	3909.7113	1955.3593	3892.6847	1946.8460	3891.7007	1946.3540	36
6	794.2994	397.6533	777.2728	389.1401	776.2888	388.6480	C	3808.6636	1904.8354	3791.6371	1896.3222	3790.6530	1895.8302	35
7	907.3834	454.1954	890.3569	445.6821	889.3729	445.1901	L	3648.6330	1824.8201	3631.6064	1816.3068	3630.6224	1815.8148	34
8	964.4049	482.7061	947.3784	474.1928	946.3943	473.7008	G	3535.5489	1768.2781	3518.5223	1759.7648	3517.5383	1759.2728	33
9	1078.4478	539.7276	1061.4213	531.2143	1060.4373	530.7223	N	3478.5274	1739.7674	3461.5009	1731.2541	3460.5169	1730.7621	32
10	1135.4693	568.2383	1118.4428	559.7250	1117.4587	559.2330	G	3364.4845	1682.7459	3347.4580	1674.2326	3346.4739	1673.7406	31
11	1234.5377	617.7725	1217.5112	609.2592	1216.5271	608.7672	V	3307.4630	1654.2352	3290.4365	1645.7219	3289.4525	1645.2299	30
12	1321.5697	661.2885	1304.5432	652.7752	1303.5592	652.2832	S	3208.3946	1604.7009	3191.3681	1596.1877	3190.3841	1595.6957	29
13	1481.6004	741.3038	1464.5738	732.7906	1463.5898	732.2986	C	3121.3626	1561.1849	3104.3360	1552.6717	3103.3520	1552.1797	28
14	1609.6590	805.3331	1592.6324	796.8198	1591.6484	796.3278	Q	2961.3319	1481.1696	2944.3054	1472.6563	2943.3214	1472.1643	27
15	1738.7016	869.8544	1721.6750	861.3411	1720.6910	860.8491	E	2833.2734	1417.1403	2816.2468	1408.6270	2815.2628	1408.1350	26
16	1839.7492	920.3783	1822.7227	911.8650	1821.7387	911.3730	T	2704.2308	1352.6190	2687.2042	1344.1058	2686.2202	1343.6137	25
17	1910.7864	955.8968	1893.7598	947.3835	1892.7758	946.8915	A	2603.1831	1302.0952	2586.1565	1293.5819	2585.1725	1293.0899	24
18	2009.8548	1005.4310	1992.8282	996.9177	1991.8442	996.4257	V	2532.1460	1266.5766	2515.1194	1258.0634	2514.1354	1257.5713	23

**19**	2110.9024	1055.9549	2093.8759	1047.4416	2092.8919	1046.9496	**T**	2433.0776	1217.0424	2416.0510	1208.5291	2415.0670	1208.0371	**22**
**20**	2238.9610	1119.9842	2221.9345	1111.4709	2220.9505	1110.9789	**Q**	2332.0299	1166.5186	2315.0033	1158.0053	2314.0193	1157.5133	**21**
**21**	2340.0087	1170.5080	2322.9822	1161.9947	2321.9981	1161.5027	**T**	2203.9713	1102.4893	2186.9448	1093.9760	2185.9607	1093.4840	**20**
**22**	2503.0720	1252.0397	2486.0455	1243.5264	2485.0615	1243.0344	**Y**	2102.9236	1051.9655	2085.8971	1043.4522	2084.9131	1042.9602	**19**
**23**	2560.0935	1280.5504	2543.0669	1272.0371	2542.0829	1271.5451	**G**	1939.8603	970.4338	1922.8338	961.9205	1921.8497	961.4285	**18**
**24**	2617.1150	1309.0611	2600.0884	1300.5478	2599.1044	1300.0558	**G**	1882.8388	941.9231	1865.8123	933.4098	1864.8283	932.9178	**17**
**25**	2731.1579	1366.0826	2714.1313	1357.5693	2713.1473	1357.0773	**N**	1825.8174	913.4123	1808.7908	904.8991	1807.8068	904.4070	**16**
**26**	2818.1899	1409.5986	2801.1634	1401.0853	2800.1794	1400.5933	**S**	1711.7744	856.3909	1694.7479	847.8776	1693.7639	847.3856	**15**
**27**	2932.2328	1466.6201	2915.2063	1458.1068	2914.2223	1457.6148	**N**	1624.7424	812.8748	1607.7159	804.3616	1606.7319	803.8696	**14**
**28**	2989.2543	1495.1308	2972.2278	1486.6175	2971.2437	1486.1255	**G**	1510.6995	755.8534	1493.6729	747.3401	1492.6889	746.8481	**13**
**29**	3118.2969	1559.6521	3101.2704	1551.1388	3100.2863	1550.6468	**E**	1453.6780	727.3427	1436.6515	718.8294	1435.6675	718.3374	**12**
**30**	3215.3497	1608.1785	3198.3231	1599.6652	3197.3391	1599.1732	**P**	1324.6354	662.8214	1307.6089	654.3081	1306.6249	653.8161	**11**
**31**	3375.3803	1688.1938	3358.3538	1679.6805	3357.3697	1679.1885	**C**	1227.5827	614.2950	1210.5561	605.7817	1209.5721	605.2897	**10**
**32**	3474.4487	1737.7280	3457.4222	1729.2147	3456.4382	1728.7227	**V**	1067.5520	534.2796	1050.5255	525.7664	1049.5415	525.2744	**9**
**33**	3587.5328	1794.2700	3570.5062	1785.7568	3569.5222	1785.2648	**L**	***968.4836***	484.7454	951.4571	476.2322	950.4730	475.7402	**8**
**34**	3684.5856	1842.7964	3667.5590	1834.2831	3666.5750	1833.7911	**P**	***855.3995***	428.2034	838.3730	419.6901	837.3890	419.1981	**7**
**35**	3831.6540	1916.3306	3814.6274	1907.8173	3813.6434	1907.3253	**F**	758.3468	379.6770	741.3202	371.1638	740.3362	370.6717	**6**
**36**	3932.7016	1966.8545	3915.6751	1958.3412	3914.6911	1957.8492	**T**	***611.2784***	306.1428	594.2518	297.6295	593.2678	297.1375	**5**
**37**	4095.7650	2048.3861	4078.7384	2039.8729	4077.7544	2039.3808	**Y**	***510.2307***	255.6190	493.2041	247.1057			**4**
**38**	4210.7919	2105.8996	4193.7654	2097.3863	4192.7814	2096.8943	**N**	***347.1674***	174.0873	330.1408	165.5740			**3**
**39**	4267.8134	2134.4103	4250.7868	2125.8971	4249.8028	2125.4050	**G**	***232.1404***	116.5738	215.1139	108.0606			**2**
**40**							**R**	175.1190	88.0631	158.0924	79.5498			**1**

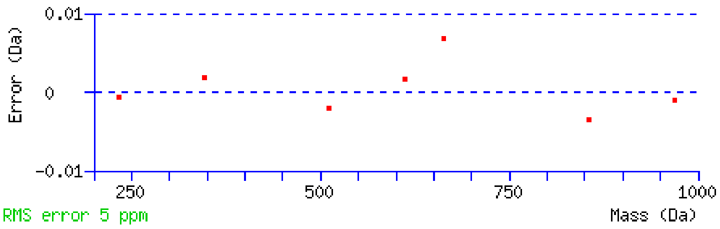

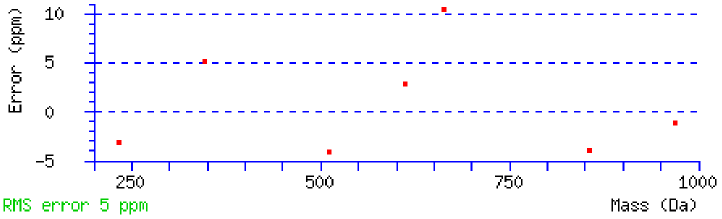

NCBI **BLAST** search of QMLCTCLGNGVSCQETAVTQTYGGNSNGEPCVLPFTYNGR
(Parameters: blastp, nr protein database, expect=20000, no filter, PAM30)
Other BLAST web gateways

All matches to this query

Score	Mr(calc)	Delta	Sequence
38.9	4440.9178	0.0074	QMLCTCLGNGVSCQETAVTQTYGGNSNGEPCVLPFTYNGR
5.0	4438.9173	2.0079	NQEAFKHLYFEKFPGYYDTMDAGYMDEEGYLYVMSR
5.0	4438.9173	2.0079	NQEAFKHLYFEKFPGYYDTMDAGYMDEEGYLYVMSR
5.0	4438.9173	2.0079	NQEAFKHLYFEKFPGYYDTMDAGYMDEEGYLYVMSR

Mascot: http://www.matrixscience.com/

Mascot Search Results

Peptide View

MS/MS Fragmentation of **QMLCTCLGNGVSCQETAVTQTYGGNSNGEPCVLPFTYNGR**
Found in **P02751** in **uni_human_nr**, FINC_HUMAN Fibronectin OS=Homo sapiens GN=FN1 PE=1 SV=4

Match to Query 16874: 4440.904976 from(1111.233520,4+) intensity(196461.0781) rtinseconds(3129) scans(17786) index(13260)
Title: 130801_HPL_Human_Plaque_BR2_TR1_15_Spectrum033022_scans__17786_RTINSECONDS=3129
Data file E:\\RAW\\HumanPlaques\\mgf\\T\\1D\\T130801_HPL_Human_Plaque_BR2_TR1_15.mgf

Click mouse within plot area to zoom in by factor of two about that point
Or, Plot from 0 to 1400 Da Full range
Label all possible matches ○ Label matches used for scoring ⊙
Show Y-axis ☐

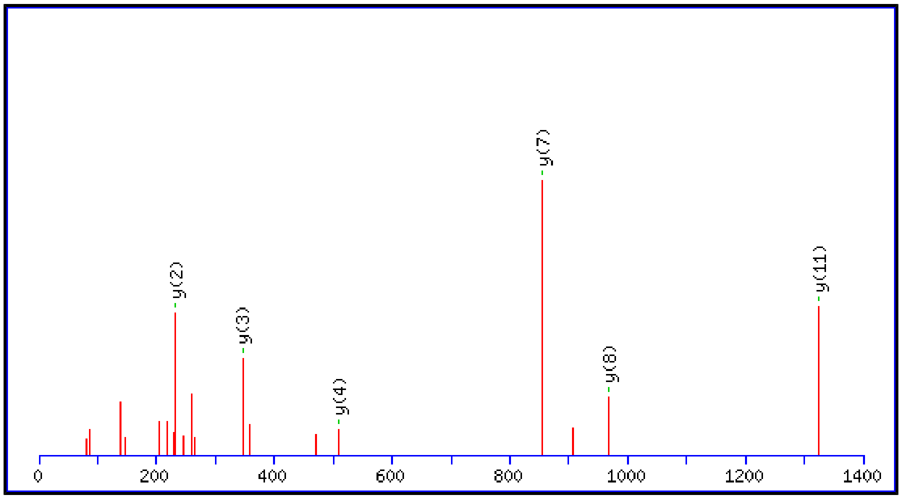

Monoisotopic mass of neutral peptide Mr(calc): 4440.9178
Fixed modifications: Carbamidomethyl (C) (apply to specified residues or termini only)
Variable modifications:
N38 : Deamidated (NQ)
Ions Score: 37 **Expect:** 0.0033
Matches : 6/452 fragment ions using 8 most intense peaks (help)

#	b	b^{++}	b^{*}	b^{*++}	b^{0}	b^{0++}	Seq.	y	y^{++}	y^{*}	y^{*++}	y^{0}	y^{0++}	#
1	129.0659	65.0366	112.0393	56.5233			Q							40
2	260.1063	130.5568	243.0798	122.0435			M	4313.8665	2157.4369	4296.8399	2148.9236	4295.8559	2148.4316	39
3	373.1904	187.0988	356.1639	178.5856			L	4182.8260	2091.9166	4165.7994	2083.4034	4164.8154	2082.9114	38
4	533.2211	267.1142	516.1945	258.6009			C	4069.7419	2035.3746	4052.7154	2026.8613	4051.7314	2026.3693	37
5	634.2687	317.6380	617.2422	309.1247	616.2582	308.6327	T	3909.7113	1955.3593	3892.6847	1946.8460	3891.7007	1946.3540	36
6	794.2994	397.6533	777.2728	389.1401	776.2888	388.6480	C	3808.6636	1904.8354	3791.6371	1896.3222	3790.6530	1895.8302	35
7	907.3834	454.1954	890.3569	445.6821	889.3729	445.1901	L	3648.6330	1824.8201	3631.6064	1816.3068	3630.6224	1815.8148	34
8	964.4049	482.7061	947.3784	474.1928	946.3943	473.7008	G	3535.5489	1768.2781	3518.5223	1759.7648	3517.5383	1759.2728	33
9	1078.4478	539.7276	1061.4213	531.2143	1060.4373	530.7223	N	3478.5274	1739.7674	3461.5009	1731.2541	3460.5169	1730.7621	32
10	1135.4693	568.2383	1118.4428	559.7250	1117.4587	559.2330	G	3364.4845	1682.7459	3347.4580	1674.2326	3346.4739	1673.7406	31
11	1234.5377	617.7725	1217.5112	609.2592	1216.5271	608.7672	V	3307.4630	1654.2352	3290.4365	1645.7219	3289.4525	1645.2299	30
12	1321.5697	661.2885	1304.5432	652.7752	1303.5592	652.2832	S	3208.3946	1604.7009	3191.3681	1596.1877	3190.3841	1595.6957	29
13	1481.6004	741.3038	1464.5738	732.7906	1463.5898	732.2986	C	3121.3626	1561.1849	3104.3360	1552.6717	3103.3520	1552.1797	28
14	1609.6590	805.3331	1592.6324	796.8198	1591.6484	796.3278	Q	2961.3319	1481.1696	2944.3054	1472.6563	2943.3214	1472.1643	27
15	1738.7016	869.8544	1721.6750	861.3411	1720.6910	860.8491	E	2833.2734	1417.1403	2816.2468	1408.6270	2815.2628	1408.1350	26
16	1839.7492	920.3783	1822.7227	911.8650	1821.7387	911.3730	T	2704.2308	1352.6190	2687.2042	1344.1058	2686.2202	1343.6137	25
17	1910.7864	955.8968	1893.7598	947.3835	1892.7758	946.8915	A	2603.1831	1302.0952	2586.1565	1293.5819	2585.1725	1293.0899	24
18	2009.8548	1005.4310	1992.8282	996.9177	1991.8442	996.4257	V	2532.1460	1266.5766	2515.1194	1258.0634	2514.1354	1257.5713	23

**19**	2110.9024	1055.9549	2093.8759	1047.4416	2092.8919	1046.9496	**T**	2433.0776	1217.0424	2416.0510	1208.5291	2415.0670	1208.0371	**22**
**20**	2238.9610	1119.9842	2221.9345	1111.4709	2220.9505	1110.9789	**Q**	2332.0299	1166.5186	2315.0033	1158.0053	2314.0193	1157.5133	**21**
**21**	2340.0087	1170.5080	2322.9822	1161.9947	2321.9981	1161.5027	**T**	2203.9713	1102.4893	2186.9448	1093.9760	2185.9607	1093.4840	**20**
**22**	2503.0720	1252.0397	2486.0455	1243.5264	2485.0615	1243.0344	**Y**	2102.9236	1051.9655	2085.8971	1043.4522	2084.9131	1042.9602	**19**
**23**	2560.0935	1280.5504	2543.0669	1272.0371	2542.0829	1271.5451	**G**	1939.8603	970.4338	1922.8338	961.9205	1921.8497	961.4285	**18**
**24**	2617.1150	1309.0611	2600.0884	1300.5478	2599.1044	1300.0558	**G**	1882.8388	941.9231	1865.8123	933.4098	1864.8283	932.9178	**17**
**25**	2731.1579	1366.0826	2714.1313	1357.5693	2713.1473	1357.0773	**N**	1825.8174	913.4123	1808.7908	904.8991	1807.8068	904.4070	**16**
**26**	2818.1899	1409.5986	2801.1634	1401.0853	2800.1794	1400.5933	**S**	1711.7744	856.3909	1694.7479	847.8776	1693.7639	847.3856	**15**
**27**	2932.2328	1466.6201	2915.2063	1458.1068	2914.2223	1457.6148	**N**	1624.7424	812.8748	1607.7159	804.3616	1606.7319	803.8696	**14**
**28**	2989.2543	1495.1308	2972.2278	1486.6175	2971.2437	1486.1255	**G**	1510.6995	755.8534	1493.6729	747.3401	1492.6889	746.8481	**13**
**29**	3118.2969	1559.6521	3101.2704	1551.1388	3100.2863	1550.6468	**E**	1453.6780	727.3427	1436.6515	718.8294	1435.6675	718.3374	**12**
**30**	3215.3497	1608.1785	3198.3231	1599.6652	3197.3391	1599.1732	**P**	***1324.6354***	662.8214	1307.6089	654.3081	1306.6249	653.8161	**11**
**31**	3375.3803	1688.1938	3358.3538	1679.6805	3357.3697	1679.1885	**C**	1227.5827	614.2950	1210.5561	605.7817	1209.5721	605.2897	**10**
**32**	3474.4487	1737.7280	3457.4222	1729.2147	3456.4382	1728.7227	**V**	1067.5520	534.2796	1050.5255	525.7664	1049.5415	525.2744	**9**
**33**	3587.5328	1794.2700	3570.5062	1785.7568	3569.5222	1785.2648	**L**	***968.4836***	484.7454	951.4571	476.2322	950.4730	475.7402	**8**
**34**	3684.5856	1842.7964	3667.5590	1834.2831	3666.5750	1833.7911	**P**	***855.3995***	428.2034	838.3730	419.6901	837.3890	419.1981	**7**
**35**	3831.6540	1916.3306	3814.6274	1907.8173	3813.6434	1907.3253	**F**	758.3468	379.6770	741.3202	371.1638	740.3362	370.6717	**6**
**36**	3932.7016	1966.8545	3915.6751	1958.3412	3914.6911	1957.8492	**T**	611.2784	306.1428	594.2518	297.6295	593.2678	297.1375	**5**
**37**	4095.7650	2048.3861	4078.7384	2039.8729	4077.7544	2039.3808	**Y**	***510.2307***	255.6190	493.2041	247.1057			**4**
**38**	4210.7919	2105.8996	4193.7654	2097.3863	4192.7814	2096.8943	**N**	***347.1674***	174.0873	330.1408	165.5740			**3**
**39**	4267.8134	2134.4103	4250.7868	2125.8971	4249.8028	2125.4050	**G**	***232.1404***	116.5738	215.1139	108.0606			**2**
**40**							**R**	175.1190	88.0631	158.0924	79.5498			**1**

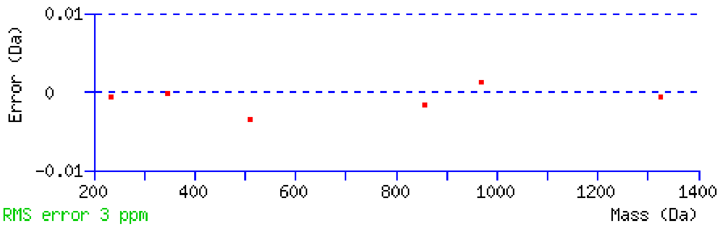

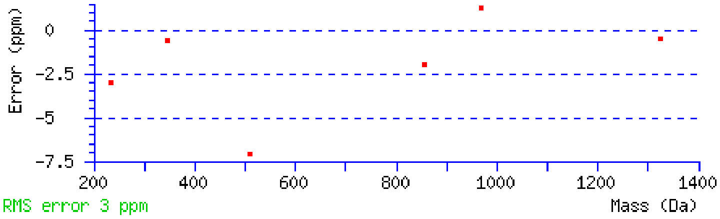

NCBI **BLAST** search of QMLCTCLGNGVSCQETAVTQTYGGNSNGEPCVLPFTYNGR
(Parameters: blastp, nr protein database, expect=20000, no filter, PAM30)
Other BLAST web gateways

All matches to this query

Score	Mr(calc)	Delta	Sequence
37.2	4440.9178	-0.0128	QMLCTCLGNGVSCQETAVTQTYGGNSNGEPCVLPFTYNGR
0.8	4438.9173	1.9877	NQEAFKHLYFEKFPGYYDTMDAGYMDEEGYLYVMSR
0.8	4438.9173	1.9877	NQEAFKHLYFEKFPGYYDTMDAGYMDEEGYLYVMSR
0.8	4438.9173	1.9877	NQEAFKHLYFEKFPGYYDTMDAGYMDEEGYLYVMSR

Mascot: http://www.matrixscience.com/

Mascot Search Results

Peptide View

MS/MS Fragmentation of **QMLCTCLGNGVSCQETAVTQTYGGNSNGEPCVLPFTYNGR**
Found in **P02751** in **uni_human_nr**, FINC_HUMAN Fibronectin OS=Homo sapiens GN=FN1 PE=1 SV=4

Match to Query 19418: 4442.938656 from(1111.741940,4+) intensity(314869.7813) rtinseconds(3504) scans(21638) index(16965)
Title: 130806_HPL_Human_Plaque_BR1_TR2_07_Spectrum038936_scans__21638_RTINSECONDS=3504
Data file E:\\RAW\\HumanPlaques\\mgf\\T\\1D\\T130806_HPL_Human_Plaque_BR1_TR2_07.mgf

Click mouse within plot area to zoom in by factor of two about that point
Or, Plot from 0 to 1400 Da Full range
Label all possible matches ○ Label matches used for scoring ⊙
Show Y-axis ☐

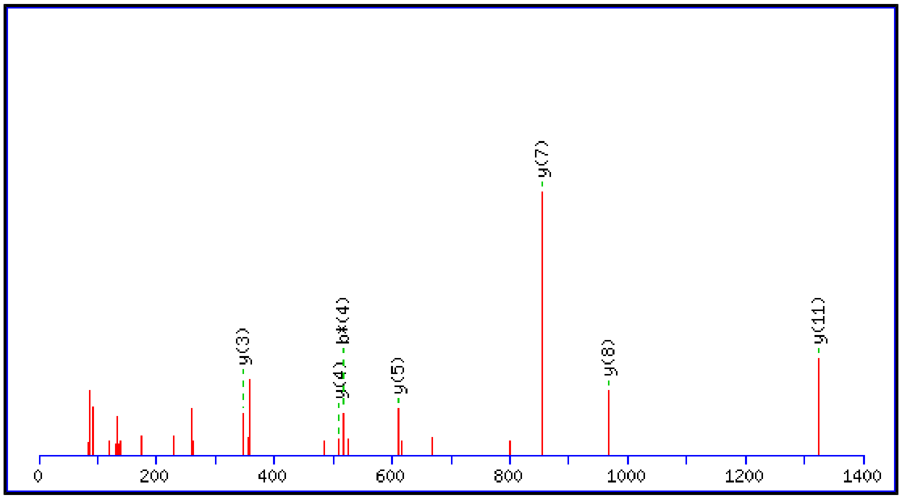

Monoisotopic mass of neutral peptide Mr(calc): 4440.9178
Fixed modifications: Carbamidomethyl (C) (apply to specified residues or termini only)
Variable modifications:
N27 : Deamidated (NQ)
Ions Score: 26 **Expect:** 0.054
Matches : 7/452 fragment ions using 15 most intense peaks (help)

#	b	b^{++}	b*	b^{*++}	b^{0}	b^{0++}	Seq.	y	y^{++}	y*	y^{*++}	y^{0}	y^{0++}	#
1	129.0659	65.0366	112.0393	56.5233			Q							40
2	260.1063	130.5568	243.0798	122.0435			M	4313.8665	2157.4369	4296.8399	2148.9236	4295.8559	2148.4316	39
3	373.1904	187.0988	356.1639	178.5856			L	4182.8260	2091.9166	4165.7994	2083.4034	4164.8154	2082.9114	38
4	533.2211	267.1142	516.1945	258.6009			C	4069.7419	2035.3746	4052.7154	2026.8613	4051.7314	2026.3693	37
5	634.2687	317.6380	617.2422	309.1247	616.2582	308.6327	T	3909.7113	1955.3593	3892.6847	1946.8460	3891.7007	1946.3540	36
6	794.2994	397.6533	777.2728	389.1401	776.2888	388.6480	C	3808.6636	1904.8354	3791.6371	1896.3222	3790.6530	1895.8302	35
7	907.3834	454.1954	890.3569	445.6821	889.3729	445.1901	L	3648.6330	1824.8201	3631.6064	1816.3068	3630.6224	1815.8148	34
8	964.4049	482.7061	947.3784	474.1928	946.3943	473.7008	G	3535.5489	1768.2781	3518.5223	1759.7648	3517.5383	1759.2728	33
9	1078.4478	539.7276	1061.4213	531.2143	1060.4373	530.7223	N	3478.5274	1739.7674	3461.5009	1731.2541	3460.5169	1730.7621	32
10	1135.4693	568.2383	1118.4428	559.7250	1117.4587	559.2330	G	3364.4845	1682.7459	3347.4580	1674.2326	3346.4739	1673.7406	31
11	1234.5377	617.7725	1217.5112	609.2592	1216.5271	608.7672	V	3307.4630	1654.2352	3290.4365	1645.7219	3289.4525	1645.2299	30
12	1321.5697	661.2885	1304.5432	652.7752	1303.5592	652.2832	S	3208.3946	1604.7009	3191.3681	1596.1877	3190.3841	1595.6957	29
13	1481.6004	741.3038	1464.5738	732.7906	1463.5898	732.2986	C	3121.3626	1561.1849	3104.3360	1552.6717	3103.3520	1552.1797	28
14	1609.6590	805.3331	1592.6324	796.8198	1591.6484	796.3278	Q	2961.3319	1481.1696	2944.3054	1472.6563	2943.3214	1472.1643	27
15	1738.7016	869.8544	1721.6750	861.3411	1720.6910	860.8491	E	2833.2734	1417.1403	2816.2468	1408.6270	2815.2628	1408.1350	26
16	1839.7492	920.3783	1822.7227	911.8650	1821.7387	911.3730	T	2704.2308	1352.6190	2687.2042	1344.1058	2686.2202	1343.6137	25
17	1910.7864	955.8968	1893.7598	947.3835	1892.7758	946.8915	A	2603.1831	1302.0952	2586.1565	1293.5819	2585.1725	1293.0899	24
18	2009.8548	1005.4310	1992.8282	996.9177	1991.8442	996.4257	V	2532.1460	1266.5766	2515.1194	1258.0634	2514.1354	1257.5713	23

**19**	2110.9024	1055.9549	2093.8759	1047.4416	2092.8919	1046.9496	**T**	2433.0776	1217.0424	2416.0510	1208.5291	2415.0670	1208.0371	**22**
**20**	2238.9610	1119.9842	2221.9345	1111.4709	2220.9505	1110.9789	**Q**	2332.0299	1166.5186	2315.0033	1158.0053	2314.0193	1157.5133	**21**
**21**	2340.0087	1170.5080	2322.9822	1161.9947	2321.9981	1161.5027	**T**	2203.9713	1102.4893	2186.9448	1093.9760	2185.9607	1093.4840	**20**
**22**	2503.0720	1252.0397	2486.0455	1243.5264	2485.0615	1243.0344	**Y**	2102.9236	1051.9655	2085.8971	1043.4522	2084.9131	1042.9602	**19**
**23**	2560.0935	1280.5504	2543.0669	1272.0371	2542.0829	1271.5451	**G**	1939.8603	970.4338	1922.8338	961.9205	1921.8497	961.4285	**18**
**24**	2617.1150	1309.0611	2600.0884	1300.5478	2599.1044	1300.0558	**G**	1882.8388	941.9231	1865.8123	933.4098	1864.8283	932.9178	**17**
**25**	2731.1579	1366.0826	2714.1313	1357.5693	2713.1473	1357.0773	**N**	1825.8174	913.4123	1808.7908	904.8991	1807.8068	904.4070	**16**
**26**	2818.1899	1409.5986	2801.1634	1401.0853	2800.1794	1400.5933	**S**	1711.7744	856.3909	1694.7479	847.8776	1693.7639	847.3856	**15**
**27**	2933.2169	1467.1121	2916.1903	1458.5988	2915.2063	1458.1068	**N**	1624.7424	812.8748	1607.7159	804.3616	1606.7319	803.8696	**14**
**28**	2990.2383	1495.6228	2973.2118	1487.1095	2972.2278	1486.6175	**G**	1509.7155	755.3614	1492.6889	746.8481	1491.7049	746.3561	**13**
**29**	3119.2809	1560.1441	3102.2544	1551.6308	3101.2704	1551.1388	**E**	1452.6940	726.8506	1435.6675	718.3374	1434.6834	717.8454	**12**
**30**	3216.3337	1608.6705	3199.3071	1600.1572	3198.3231	1599.6652	**P**	***1323.6514***	662.3293	1306.6249	653.8161	1305.6409	653.3241	**11**
**31**	3376.3643	1688.6858	3359.3378	1680.1725	3358.3538	1679.6805	**C**	1226.5987	613.8030	1209.5721	605.2897	1208.5881	604.7977	**10**
**32**	3475.4327	1738.2200	3458.4062	1729.7067	3457.4222	1729.2147	**V**	1066.5680	533.7876	1049.5415	525.2744	1048.5574	524.7824	**9**
**33**	3588.5168	1794.7620	3571.4903	1786.2488	3570.5062	1785.7568	**L**	***967.4996***	484.2534	950.4730	475.7402	949.4890	475.2482	**8**
**34**	3685.5696	1843.2884	3668.5430	1834.7751	3667.5590	1834.2831	**P**	***854.4155***	427.7114	837.3890	419.1981	836.4050	418.7061	**7**
**35**	3832.6380	1916.8226	3815.6114	1908.3094	3814.6274	1907.8173	**F**	757.3628	379.1850	740.3362	370.6717	739.3522	370.1797	**6**
**36**	3933.6857	1967.3465	3916.6591	1958.8332	3915.6751	1958.3412	**T**	***610.2944***	305.6508	593.2678	297.1375	592.2838	296.6455	**5**
**37**	4096.7490	2048.8781	4079.7224	2040.3649	4078.7384	2039.8729	**Y**	***509.2467***	255.1270	492.2201	246.6137			**4**
**38**	4210.7919	2105.8996	4193.7654	2097.3863	4192.7814	2096.8943	**N**	***346.1833***	173.5953	329.1568	165.0820			**3**
**39**	4267.8134	2134.4103	4250.7868	2125.8971	4249.8028	2125.4050	**G**	232.1404	116.5738	215.1139	108.0606			**2**
**40**							**R**	175.1190	88.0631	158.0924	79.5498			**1**

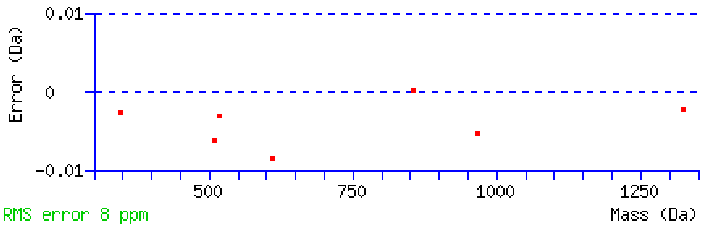

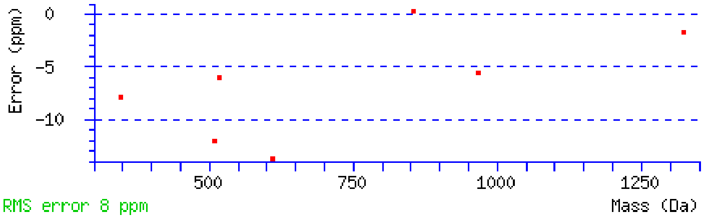

NCBI **BLAST** search of QMLCTCLGNGVSCQETAVTQTYGGNSNGEPCVLPFTYNGR
(Parameters: blastp, nr protein database, expect=20000, no filter, PAM30)
Other BLAST web gateways

All matches to this query

Score	Mr(calc)	Delta	Sequence	Site Analysis
26.3	4440.9178	2.0208	QMLCTCLGNGVSCQETAVTQTYGGNSNGEPCVLPFTYNGR	Deamidated N27 16.84%
26.3	4440.9178	2.0208	QMLCTCLGNGVSCQETAVTQTYGGNSNGEPCVLPFTYNGR	Deamidated N25 16.84%
26.3	4440.9178	2.0208	QMLCTCLGNGVSCQETAVTQTYGGNSNGEPCVLPFTYNGR	Deamidated Q20 16.84%
26.3	4440.9178	2.0208	QMLCTCLGNGVSCQETAVTQTYGGNSNGEPCVLPFTYNGR	Deamidated Q14 16.84%
26.3	4440.9178	2.0208	QMLCTCLGNGVSCQETAVTQTYGGNSNGEPCVLPFTYNGR	Deamidated N9 16.84%
26.0	4440.9178	2.0208	QMLCTCLGNGVSCQETAVTQTYGGNSNGEPCVLPFTYNGR	Deamidated Q1 15.75%
0.9	4442.9547	-0.0160	DNSKNVFFSPMSMSCALAMVYMGAKGNTAAQMAQILSFNK	
0.9	4442.9547	-0.0160	DNSKNVFFSPMSMSCALAMVYMGAKGNTAAQMAQILSFNK	

Mascot: http://www.matrixscience.com/

Mascot Search Results

Peptide View

MS/MS Fragmentation of **QMLCTCLGNGVSCQETAVTQTYGGNSNGEPCVLPFTYNGR**
Found in **P02751** in **uni_human_nr**, FINC_HUMAN Fibronectin OS=Homo sapiens GN=FN1 PE=1 SV=4

Match to Query 18685: 4439.938632 from(1480.986820,3+) intensity(2434001.5000) rtinseconds(3046) scans(18312) index(13995)
Title: 130806_HPL_Human_Plaque_BR1_TR2_08_Spectrum035687_scans__18312_RTINSECONDS=3046
Data file E:\\RAW\\HumanPlaques\\mgf\\T\\1D\\T130806_HPL_Human_Plaque_BR1_TR2_08.mgf

Click mouse within plot area to zoom in by factor of two about that point
Or, Plot from 0 to 1400 Da Full range
Label all possible matches ○ Label matches used for scoring ⦿
Show Y-axis ☐

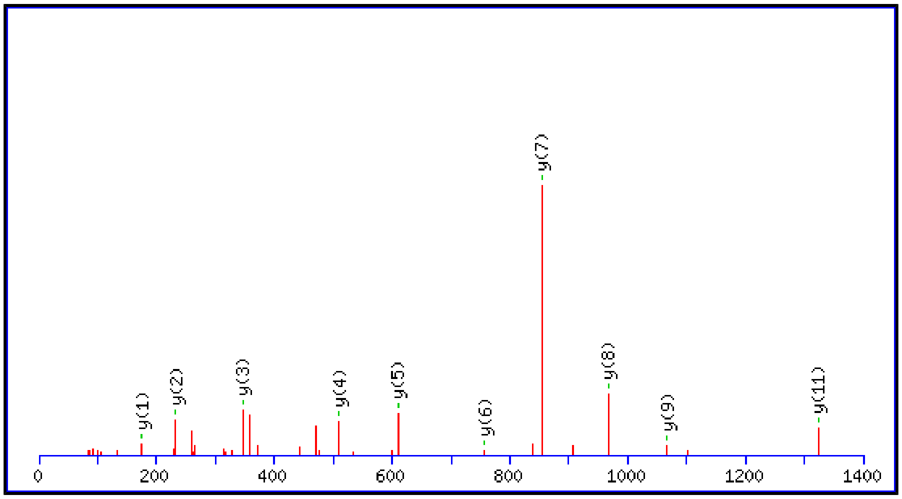

Monoisotopic mass of neutral peptide Mr(calc): 4439.9338
Fixed modifications: Carbamidomethyl (C) (apply to specified residues or termini only)
Ions Score: 75 **Expect:** 8.6e-007
Matches : 10/452 fragment ions using 12 most intense peaks (help)

#	b	b^{++}	b*	b^{*++}	b^0	b^{0++}	Seq.	y	y^{++}	y*	y^{*++}	y^0	y^{0++}	#
1	129.0659	65.0366	112.0393	56.5233			Q							40
2	260.1063	130.5568	243.0798	122.0435			M	4312.8825	2156.9449	4295.8559	2148.4316	4294.8719	2147.9396	39
3	373.1904	187.0988	356.1639	178.5856			L	4181.8420	2091.4246	4164.8154	2082.9114	4163.8314	2082.4193	38
4	533.2211	267.1142	516.1945	258.6009			C	4068.7579	2034.8826	4051.7314	2026.3693	4050.7474	2025.8773	37
5	634.2687	317.6380	617.2422	309.1247	616.2582	308.6327	T	3908.7273	1954.8673	3891.7007	1946.3540	3890.7167	1945.8620	36
6	794.2994	397.6533	777.2728	389.1401	776.2888	388.6480	C	3807.6796	1904.3434	3790.6530	1895.8302	3789.6690	1895.3381	35
7	907.3834	454.1954	890.3569	445.6821	889.3729	445.1901	L	3647.6489	1824.3281	3630.6224	1815.8148	3629.6384	1815.3228	34
8	964.4049	482.7061	947.3784	474.1928	946.3943	473.7008	G	3534.5649	1767.7861	3517.5383	1759.2728	3516.5543	1758.7808	33
9	1078.4478	539.7276	1061.4213	531.2143	1060.4373	530.7223	N	3477.5434	1739.2753	3460.5169	1730.7621	3459.5328	1730.2701	32
10	1135.4693	568.2383	1118.4428	559.7250	1117.4587	559.2330	G	3363.5005	1682.2539	3346.4739	1673.7406	3345.4899	1673.2486	31
11	1234.5377	617.7725	1217.5112	609.2592	1216.5271	608.7672	V	3306.4790	1653.7431	3289.4525	1645.2299	3288.4685	1644.7379	30
12	1321.5697	661.2885	1304.5432	652.7752	1303.5592	652.2832	S	3207.4106	1604.2089	3190.3841	1595.6957	3189.4000	1595.2037	29
13	1481.6004	741.3038	1464.5738	732.7906	1463.5898	732.2986	C	3120.3786	1560.6929	3103.3520	1552.1797	3102.3680	1551.6876	28
14	1609.6590	805.3331	1592.6324	796.8198	1591.6484	796.3278	Q	2960.3479	1480.6776	2943.3214	1472.1643	2942.3374	1471.6723	27
15	1738.7016	869.8544	1721.6750	861.3411	1720.6910	860.8491	E	2832.2894	1416.6483	2815.2628	1408.1350	2814.2788	1407.6430	26
16	1839.7492	920.3783	1822.7227	911.8650	1821.7387	911.3730	T	2703.2468	1352.1270	2686.2202	1343.6137	2685.2362	1343.1217	25
17	1910.7864	955.8968	1893.7598	947.3835	1892.7758	946.8915	A	2602.1991	1301.6032	2585.1725	1293.0899	2584.1885	1292.5979	24
18	2009.8548	1005.4310	1992.8282	996.9177	1991.8442	996.4257	V	2531.1620	1266.0846	2514.1354	1257.5713	2513.1514	1257.0793	23
19	2110.9024	1055.9549	2093.8759	1047.4416	2092.8919	1046.9496	T	2432.0936	1216.5504	2415.0670	1208.0371	2414.0830	1207.5451	22

20	2238.9610	1119.9842	2221.9345	1111.4709	2220.9505	1110.9789	Q	2331.0459	1166.0266	2314.0193	1157.5133	2313.0353	1157.0213	21
21	2340.0087	1170.5080	2322.9822	1161.9947	2321.9981	1161.5027	T	2202.9873	1101.9973	2185.9607	1093.4840	2184.9767	1092.9920	20
22	2503.0720	1252.0397	2486.0455	1243.5264	2485.0615	1243.0344	Y	2101.9396	1051.4734	2084.9131	1042.9602	2083.9291	1042.4682	19
23	2560.0935	1280.5504	2543.0669	1272.0371	2542.0829	1271.5451	G	1938.8763	969.9418	1921.8497	961.4285	1920.8657	960.9365	18
24	2617.1150	1309.0611	2600.0884	1300.5478	2599.1044	1300.0558	G	1881.8548	941.4310	1864.8283	932.9178	1863.8443	932.4258	17
25	2731.1579	1366.0826	2714.1313	1357.5693	2713.1473	1357.0773	N	1824.8334	912.9203	1807.8068	904.4070	1806.8228	903.9150	16
26	2818.1899	1409.5986	2801.1634	1401.0853	2800.1794	1400.5933	S	1710.7904	855.8989	1693.7639	847.3856	1692.7799	846.8936	15
27	2932.2328	1466.6201	2915.2063	1458.1068	2914.2223	1457.6148	N	1623.7584	812.3828	1606.7319	803.8696	1605.7478	803.3776	14
28	2989.2543	1495.1308	2972.2278	1486.6175	2971.2437	1486.1255	G	1509.7155	755.3614	1492.6889	746.8481	1491.7049	746.3561	13
29	3118.2969	1559.6521	3101.2704	1551.1388	3100.2863	1550.6468	E	1452.6940	726.8506	1435.6675	718.3374	1434.6834	717.8454	12
30	3215.3497	1608.1785	3198.3231	1599.6652	3197.3391	1599.1732	P	1323.6514	662.3293	1306.6249	653.8161	1305.6409	653.3241	11
31	3375.3803	1688.1938	3358.3538	1679.6805	3357.3697	1679.1885	C	1226.5987	613.8030	1209.5721	605.2897	1208.5881	604.7977	10
32	3474.4487	1737.7280	3457.4222	1729.2147	3456.4382	1728.7227	V	1066.5680	533.7876	1049.5415	525.2744	1048.5574	524.7824	9
33	3587.5328	1794.2700	3570.5062	1785.7568	3569.5222	1785.2648	L	967.4996	484.2534	950.4730	475.7402	949.4890	475.2482	8
34	3684.5856	1842.7964	3667.5590	1834.2831	3666.5750	1833.7911	P	854.4155	427.7114	837.3890	419.1981	836.4050	418.7061	7
35	3831.6540	1916.3306	3814.6274	1907.8173	3813.6434	1907.3253	F	757.3628	379.1850	740.3362	370.6717	739.3522	370.1797	6
36	3932.7016	1966.8545	3915.6751	1958.3412	3914.6911	1957.8492	T	610.2944	305.6508	593.2678	297.1375	592.2838	296.6455	5
37	4095.7650	2048.3861	4078.7384	2039.8729	4077.7544	2039.3808	Y	509.2467	255.1270	492.2201	246.6137			4
38	4209.8079	2105.4076	4192.7814	2096.8943	4191.7973	2096.4023	N	346.1833	173.5953	329.1568	165.0820			3
39	4266.8294	2133.9183	4249.8028	2125.4050	4248.8188	2124.9130	G	232.1404	116.5738	215.1139	108.0606			2
40							R	175.1190	88.0631	158.0924	79.5498			1

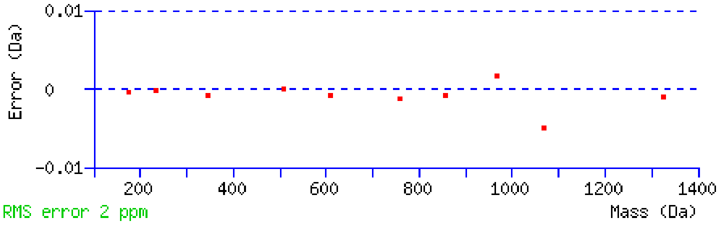

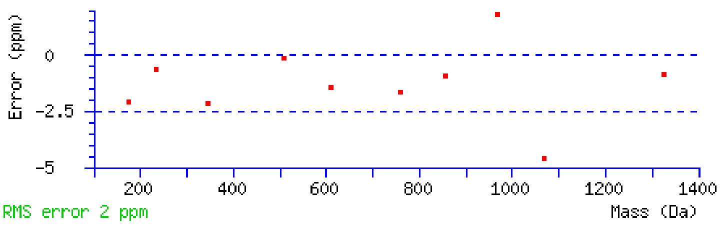

NCBI **BLAST** search of QMLCTCLGNGVSCQETAVTQTYGGNSNGEPCVLPFTYNGR
(Parameters: blastp, nr protein database, expect=20000, no filter, PAM30)
Other BLAST web gateways

All matches to this query

Score	Mr(calc)	Delta	Sequence
75.1	4439.9338	0.0048	QMLCTCLGNGVSCQETAVTQTYGGNSNGEPCVLPFTYNGR

Mascot: http://www.matrixscience.com/

Mascot Search Results

Peptide View

MS/MS Fragmentation of **QMLCTCLGNGVSCQETAVTQTYGGNSNGEPCVLPFTYNGR**
Found in **P02751** in **uni_human_nr**, FINC_HUMAN Fibronectin OS=Homo sapiens GN=FN1 PE=1 SV=4

Match to Query 19085: 4439.932816 from(1110.990480,4+) intensity(4664387.0000) rtinseconds(3040) scans(18351) index(14134)
Title: 130801_HPL_Human_Plaque_BR1_TR1_07_Spectrum035837_scans__18351_RTINSECONDS=3040
Data file E:\\RAW\\HumanPlaques\\mgf\\T\\1D\\T130801_HPL_Human_Plaque_BR1_TR1_07.mgf

Click mouse within plot area to zoom in by factor of two about that point
Or, Plot from 0 to 3800 Da Full range
Label all possible matches ○ Label matches used for scoring ⊙
Show Y-axis ☐

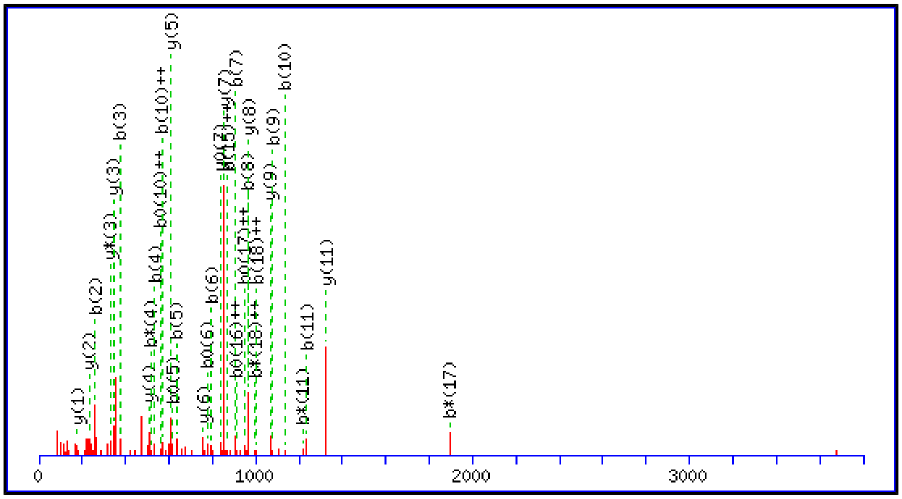

Monoisotopic mass of neutral peptide Mr(calc): 4439.9338
Fixed modifications: Carbamidomethyl (C) (apply to specified residues or termini only)
Ions Score: 68 **Expect:** 3.6e-006
Matches : 34/452 fragment ions using 64 most intense peaks (help)

#	b	b^{++}	b*	b^{*++}	b^0	b^{0++}	Seq.	y	y^{++}	y*	y^{*++}	y^0	y^{0++}	#
1	129.0659	65.0366	112.0393	56.5233			Q							40
2	***260.1063***	130.5568	243.0798	122.0435			M	4312.8825	2156.9449	4295.8559	2148.4316	4294.8719	2147.9396	39
3	***373.1904***	187.0988	356.1639	178.5856			L	4181.8420	2091.4246	4164.8154	2082.9114	4163.8314	2082.4193	38
4	***533.2211***	267.1142	516.1945	258.6009			C	4068.7579	2034.8826	4051.7314	2026.3693	4050.7474	2025.8773	37
5	***634.2687***	317.6380	617.2422	309.1247	616.2582	308.6327	T	3908.7273	1954.8673	3891.7007	1946.3540	3890.7167	1945.8620	36
6	***794.2994***	397.6533	777.2728	389.1401	776.2888	388.6480	C	3807.6796	1904.3434	3790.6530	1895.8302	3789.6690	1895.3381	35
7	***907.3834***	454.1954	890.3569	445.6821	889.3729	445.1901	L	3647.6489	1824.3281	3630.6224	1815.8148	3629.6384	1815.3228	34
8	***964.4049***	482.7061	947.3784	474.1928	946.3943	473.7008	G	3534.5649	1767.7861	3517.5383	1759.2728	3516.5543	1758.7808	33
9	***1078.4478***	539.7276	1061.4213	531.2143	1060.4373	530.7223	N	3477.5434	1739.2753	3460.5169	1730.7621	3459.5328	1730.2701	32
10	***1135.4693***	**568.2383**	1118.4428	559.7250	1117.4587	559.2330	G	3363.5005	1682.2539	3346.4739	1673.7406	3345.4899	1673.2486	31
11	***1234.5377***	617.7725	1217.5112	609.2592	1216.5271	608.7672	V	3306.4790	1653.7431	3289.4525	1645.2299	3288.4685	1644.7379	30
12	1321.5697	661.2885	1304.5432	652.7752	1303.5592	652.2832	S	3207.4106	1604.2089	3190.3841	1595.6957	3189.4000	1595.2037	29
13	1481.6004	741.3038	1464.5738	732.7906	1463.5898	732.2986	C	3120.3786	1560.6929	3103.3520	1552.1797	3102.3680	1551.6876	28
14	1609.6590	805.3331	1592.6324	796.8198	1591.6484	796.3278	Q	2960.3479	1480.6776	2943.3214	1472.1643	2942.3374	1471.6723	27
15	1738.7016	**869.8544**	1721.6750	861.3411	1720.6910	860.8491	E	2832.2894	1416.6483	2815.2628	1408.1350	2814.2788	1407.6430	26
16	1839.7492	920.3783	1822.7227	911.8650	1821.7387	911.3730	T	2703.2468	1352.1270	2686.2202	1343.6137	2685.2362	1343.1217	25
17	1910.7864	955.8968	1893.7598	947.3835	1892.7758	946.8915	A	2602.1991	1301.6032	2585.1725	1293.0899	2584.1885	1292.5979	24
18	2009.8548	**1005.4310**	1992.8282	996.9177	1991.8442	996.4257	V	2531.1620	1266.0846	2514.1354	1257.5713	2513.1514	1257.0793	23
19	2110.9024	1055.9549	2093.8759	1047.4416	2092.8919	1046.9496	T	2432.0936	1216.5504	2415.0670	1208.0371	2414.0830	1207.5451	22

**20**	2238.9610	1119.9842	2221.9345	1111.4709	2220.9505	1110.9789	**Q**	2331.0459	1166.0266	2314.0193	1157.5133	2313.0353	1157.0213	**21**
**21**	2340.0087	1170.5080	2322.9822	1161.9947	2321.9981	1161.5027	**T**	2202.9873	1101.9973	2185.9607	1093.4840	2184.9767	1092.9920	**20**
**22**	2503.0720	1252.0397	2486.0455	1243.5264	2485.0615	1243.0344	**Y**	2101.9396	1051.4734	2084.9131	1042.9602	2083.9291	1042.4682	**19**
**23**	2560.0935	1280.5504	2543.0669	1272.0371	2542.0829	1271.5451	**G**	1938.8763	969.9418	1921.8497	961.4285	1920.8657	960.9365	**18**
**24**	2617.1150	1309.0611	2600.0884	1300.5478	2599.1044	1300.0558	**G**	1881.8548	941.4310	1864.8283	932.9178	1863.8443	932.4258	**17**
**25**	2731.1579	1366.0826	2714.1313	1357.5693	2713.1473	1357.0773	**N**	1824.8334	912.9203	1807.8068	904.4070	1806.8228	903.9150	**16**
**26**	2818.1899	1409.5986	2801.1634	1401.0853	2800.1794	1400.5933	**S**	1710.7904	855.8989	1693.7639	847.3856	1692.7799	846.8936	**15**
**27**	2932.2328	1466.6201	2915.2063	1458.1068	2914.2223	1457.6148	**N**	1623.7584	812.3828	1606.7319	803.8696	1605.7478	803.3776	**14**
**28**	2989.2543	1495.1308	2972.2278	1486.6175	2971.2437	1486.1255	**G**	1509.7155	755.3614	1492.6889	746.8481	1491.7049	746.3561	**13**
**29**	3118.2969	1559.6521	3101.2704	1551.1388	3100.2863	1550.6468	**E**	1452.6940	726.8506	1435.6675	718.3374	1434.6834	717.8454	**12**
**30**	3215.3497	1608.1785	3198.3231	1599.6652	3197.3391	1599.1732	**P**	***1323.6514***	662.3293	1306.6249	653.8161	1305.6409	653.3241	**11**
**31**	3375.3803	1688.1938	3358.3538	1679.6805	3357.3697	1679.1885	**C**	1226.5987	613.8030	1209.5721	605.2897	1208.5881	604.7977	**10**
**32**	3474.4487	1737.7280	3457.4222	1729.2147	3456.4382	1728.7227	**V**	***1066.5680***	533.7876	1049.5415	525.2744	1048.5574	524.7824	**9**
**33**	3587.5328	1794.2700	3570.5062	1785.7568	3569.5222	1785.2648	**L**	***967.4996***	484.2534	950.4730	475.7402	949.4890	475.2482	**8**
**34**	3684.5856	1842.7964	3667.5590	1834.2831	3666.5750	1833.7911	**P**	***854.4155***	427.7114	837.3890	419.1981	836.4050	418.7061	**7**
**35**	3831.6540	1916.3306	3814.6274	1907.8173	3813.6434	1907.3253	**F**	***757.3628***	379.1850	740.3362	370.6717	739.3522	370.1797	**6**
**36**	3932.7016	1966.8545	3915.6751	1958.3412	3914.6911	1957.8492	**T**	***610.2944***	305.6508	593.2678	297.1375	592.2838	296.6455	**5**
**37**	4095.7650	2048.3861	4078.7384	2039.8729	4077.7544	2039.3808	**Y**	***509.2467***	255.1270	492.2201	246.6137			**4**
**38**	4209.8079	2105.4076	4192.7814	2096.8943	4191.7973	2096.4023	**N**	***346.1833***	173.5953	329.1568	165.0820			**3**
**39**	4266.8294	2133.9183	4249.8028	2125.4050	4248.8188	2124.9130	**G**	***232.1404***	116.5738	215.1139	108.0606			**2**
**40**							**R**	***175.1190***	88.0631	158.0924	79.5498			**1**

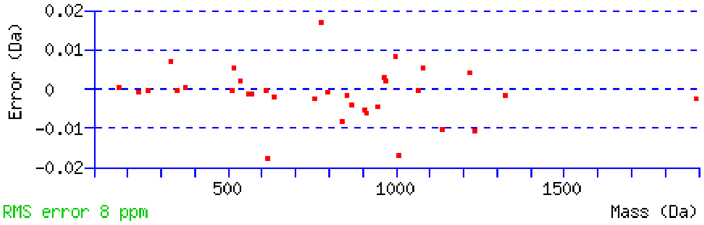

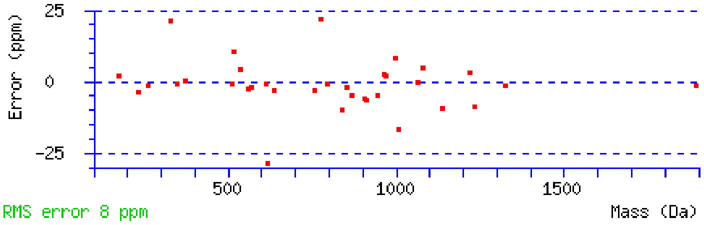

NCBI **BLAST** search of QMLCTCLGNGVSCQETAVTQTYGGNSNGEPCVLPFTYNGR
(Parameters: blastp, nr protein database, expect=20000, no filter, PAM30)
Other BLAST web gateways

All matches to this query

Score	Mr(calc)	Delta	Sequence
68.3	4439.9338	-0.0010	QMLCTCLGNGVSCQETAVTQTYGGNSNGEPCVLPFTYNGR

Mascot: http://www.matrixscience.com/

Mascot Search Results

Peptide View

MS/MS Fragmentation of **TQGNKQMLCTCLGNGVSCQETAVTQTYGGNSNGEPCVLPFTYNGR**
Found in **P02751** in **uni_human_nr**, FINC_HUMAN Fibronectin OS=Homo sapiens GN=FN1 PE=1 SV=4

Match to Query 20159: 4987.187696 from(1247.804200,4+) intensity(1131854.8750) rtinseconds(2674) scans(16239) index(12826)
Title: 130809_HPL_Human_Plaque_BR2_TR3_06_Spectrum034866_scans__16239_RTINSECONDS=2674
Data file E:\\RAW\\HumanPlaques\\mgf\\T\\1D\\T130809_HPL_Human_Plaque_BR2_TR3_06.mgf

Click mouse within plot area to zoom in by factor of two about that point
Or, Plot from 0 to 2200 Da Full range
Label all possible matches ○ Label matches used for scoring ⊙
Show Y-axis ☐

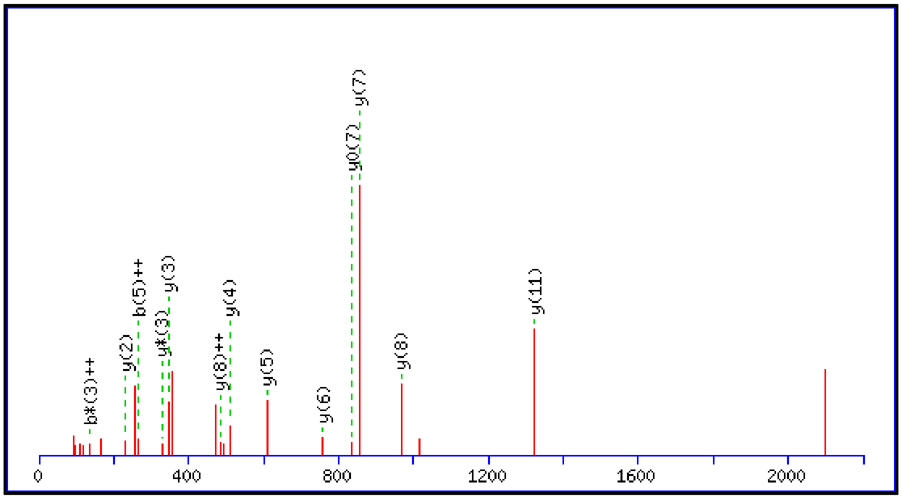

Monoisotopic mass of neutral peptide Mr(calc): 4986.1624
Fixed modifications: Carbamidomethyl (C) (apply to specified residues or termini only)
Variable modifications:
Q2 : Deamidated (NQ)
N4 : Deamidated (NQ)
M7 : Oxidation (M), with neutral losses 0.0000(shown in table), 63.9983
Ions Score: 34 **Expect:** 0.0089
Matches : 13/782 fragment ions using 26 most intense peaks (help)

#	b	b^{++}	b*	b^{*++}	b^0	b^{0++}	Seq.	y	y^{++}	y*	y^{*++}	y^0	y^{0++}	#
1	102.0550	51.5311			84.0444	42.5258	T							45
2	231.0975	116.0524	214.0710	107.5391	213.0870	107.0471	Q	4886.1219	2443.5646	4869.0954	2435.0513	4868.1114	2434.5593	44
3	288.1190	144.5631	271.0925	136.0499	270.1084	135.5579	G	4757.0793	2379.0433	4740.0528	2370.5300	4739.0688	2370.0380	43
4	403.1460	202.0766	386.1194	193.5633	385.1354	193.0713	N	4700.0579	2350.5326	4683.0313	2342.0193	4682.0473	2341.5273	42
5	531.2409	**266.1241**	514.2144	257.6108	513.2304	257.1188	K	4585.0309	2293.0191	4568.0044	2284.5058	4567.0204	2284.0138	41
6	659.2995	330.1534	642.2729	321.6401	641.2889	321.1481	Q	4456.9360	2228.9716	4439.9094	2220.4583	4438.9254	2219.9663	40
7	806.3349	403.6711	789.3083	395.1578	788.3243	394.6658	M	4328.8774	2164.9423	4311.8508	2156.4291	4310.8668	2155.9370	39
8	919.4190	460.2131	902.3924	451.6998	901.4084	451.2078	L	4181.8420	2091.4246	4164.8154	2082.9114	4163.8314	2082.4193	38
9	1079.4496	540.2284	1062.4231	531.7152	1061.4390	531.2232	C	4068.7579	2034.8826	4051.7314	2026.3693	4050.7474	2025.8773	37
10	1180.4973	590.7523	1163.4707	582.2390	1162.4867	581.7470	T	3908.7273	1954.8673	3891.7007	1946.3540	3890.7167	1945.8620	36
11	1340.5279	670.7676	1323.5014	662.2543	1322.5174	661.7623	C	3807.6796	1904.3434	3790.6530	1895.8302	3789.6690	1895.3381	35
12	1453.6120	727.3096	1436.5855	718.7964	1435.6014	718.3044	L	3647.6489	1824.3281	3630.6224	1815.8148	3629.6384	1815.3228	34
13	1510.6335	755.8204	1493.6069	747.3071	1492.6229	746.8151	G	3534.5649	1767.7861	3517.5383	1759.2728	3516.5543	1758.7808	33
14	1624.6764	812.8418	1607.6498	804.3286	1606.6658	803.8366	N	3477.5434	1739.2753	3460.5169	1730.7621	3459.5328	1730.2701	32
15	1681.6979	841.3526	1664.6713	832.8393	1663.6873	832.3473	G	3363.5005	1682.2539	3346.4739	1673.7406	3345.4899	1673.2486	31
16	1780.7663	890.8868	1763.7397	882.3735	1762.7557	881.8815	V	3306.4790	1653.7431	3289.4525	1645.2299	3288.4685	1644.7379	30
17	1867.7983	934.4028	1850.7718	925.8895	1849.7877	925.3975	S	3207.4106	1604.2089	3190.3841	1595.6957	3189.4000	1595.2037	29

18	2027.8289	1014.4181	2010.8024	1005.9048	2009.8184	1005.4128	C	3120.3786	1560.6929	3103.3520	1552.1797	3102.3680	1551.6876	28
19	2155.8875	1078.4474	2138.8610	1069.9341	2137.8770	1069.4421	Q	2960.3479	1480.6776	2943.3214	1472.1643	2942.3374	1471.6723	27
20	2284.9301	1142.9687	2267.9036	1134.4554	2266.9196	1133.9634	E	2832.2894	1416.6483	2815.2628	1408.1350	2814.2788	1407.6430	26
21	2385.9778	1193.4925	2368.9512	1184.9793	2367.9672	1184.4873	T	2703.2468	1352.1270	2686.2202	1343.6137	2685.2362	1343.1217	25
22	2457.0149	1229.0111	2439.9884	1220.4978	2439.0043	1220.0058	A	2602.1991	1301.6032	2585.1725	1293.0899	2584.1885	1292.5979	24
23	2556.0833	1278.5453	2539.0568	1270.0320	2538.0728	1269.5400	V	2531.1620	1266.0846	2514.1354	1257.5713	2513.1514	1257.0793	23
24	2657.1310	1329.0691	2640.1045	1320.5559	2639.1204	1320.0639	T	2432.0936	1216.5504	2415.0670	1208.0371	2414.0830	1207.5451	22
25	2785.1896	1393.0984	2768.1630	1384.5852	2767.1790	1384.0931	Q	2331.0459	1166.0266	2314.0193	1157.5133	2313.0353	1157.0213	21
26	2886.2373	1443.6223	2869.2107	1435.1090	2868.2267	1434.6170	T	2202.9873	1101.9973	2185.9607	1093.4840	2184.9767	1092.9920	20
27	3049.3006	1525.1539	3032.2740	1516.6407	3031.2900	1516.1487	Y	2101.9396	1051.4734	2084.9131	1042.9602	2083.9291	1042.4682	19
28	3106.3221	1553.6647	3089.2955	1545.1514	3088.3115	1544.6594	G	1938.8763	969.9418	1921.8497	961.4285	1920.8657	960.9365	18
29	3163.3435	1582.1754	3146.3170	1573.6621	3145.3330	1573.1701	G	1881.8548	941.4310	1864.8283	932.9178	1863.8443	932.4258	17
30	3277.3864	1639.1969	3260.3599	1630.6836	3259.3759	1630.1916	N	1824.8334	912.9203	1807.8068	904.4070	1806.8228	903.9150	16
31	3364.4185	1682.7129	3347.3919	1674.1996	3346.4079	1673.7076	S	1710.7904	855.8989	1693.7639	847.3856	1692.7799	846.8936	15
32	3478.4614	1739.7343	3461.4349	1731.2211	3460.4508	1730.7291	N	1623.7584	812.3828	1606.7319	803.8696	1605.7478	803.3776	14
33	3535.4829	1768.2451	3518.4563	1759.7318	3517.4723	1759.2398	G	1509.7155	755.3614	1492.6889	746.8481	1491.7049	746.3561	13
34	3664.5255	1832.7664	3647.4989	1824.2531	3646.5149	1823.7611	E	1452.6940	726.8506	1435.6675	718.3374	1434.6834	717.8454	12
35	3761.5782	1881.2927	3744.5517	1872.7795	3743.5677	1872.2875	P	**1323.6514**	662.3293	1306.6249	653.8161	1305.6409	653.3241	11
36	3921.6089	1961.3081	3904.5823	1952.7948	3903.5983	1952.3028	C	1226.5987	613.8030	1209.5721	605.2897	1208.5881	604.7977	10
37	4020.6773	2010.8423	4003.6507	2002.3290	4002.6667	2001.8370	V	1066.5680	533.7876	1049.5415	525.2744	1048.5574	524.7824	9
38	4133.7613	2067.3843	4116.7348	2058.8710	4115.7508	2058.3790	L	**967.4996**	484.2534	950.4730	475.7402	949.4890	475.2482	8
39	4230.8141	2115.9107	4213.7876	2107.3974	4212.8035	2106.9054	P	**854.4155**	427.7114	837.3890	419.1981	836.4050	418.7061	7
40	4377.8825	2189.4449	4360.8560	2180.9316	4359.8720	2180.4396	F	**757.3628**	379.1850	740.3362	370.6717	739.3522	370.1797	6
41	4478.9302	2239.9687	4461.9037	2231.4555	4460.9196	2230.9635	T	**610.2944**	305.6508	593.2678	297.1375	592.2838	296.6455	5
42	4641.9935	2321.5004	4624.9670	2312.9871	4623.9830	2312.4951	Y	**509.2467**	255.1270	492.2201	246.6137			4
43	4756.0365	2378.5219	4739.0099	2370.0086	4738.0259	2369.5166	N	**346.1833**	173.5953	329.1568	165.0820			3
44	4813.0579	2407.0326	4796.0314	2398.5193	4795.0474	2398.0273	G	**232.1404**	116.5738	215.1139	108.0606			2
45							R	175.1190	88.0631	158.0924	79.5498			1

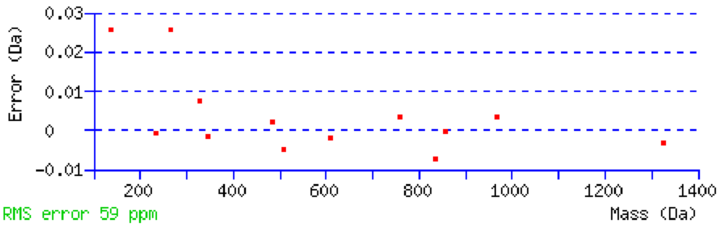

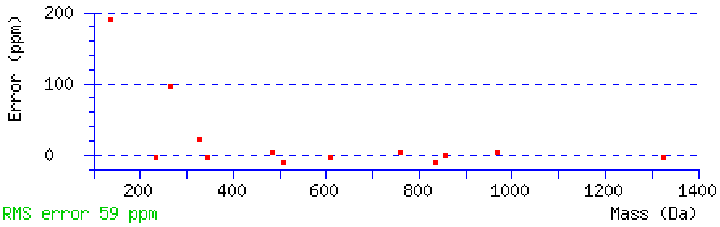

NCBI **BLAST** search of TQGNKQMLCTCLGNGVSCQETAVTQTYGGNSNGEPCVLPFTYNGR
(Parameters: blastp, nr protein database, expect=20000, no filter, PAM30)
Other BLAST web gateways

All matches to this query

Score	Mr(calc)	Delta	Sequence	Site Analysis
33.5	4986.1624	1.0253	TQGNKQMLCTCLGNGVSCQETAVTQTYGGNSNGEPCVLPFTYNGR	Deamidated Q2, N4 3.38%
33.5	4985.1783	2.0094	TQGNKQMLCTCLGNGVSCQETAVTQTYGGNSNGEPCVLPFTYNGR	
33.5	4985.1783	2.0094	TQGNKQMLCTCLGNGVSCQETAVTQTYGGNSNGEPCVLPFTYNGR	
33.5	4986.1624	1.0253	TQGNKQMLCTCLGNGVSCQETAVTQTYGGNSNGEPCVLPFTYNGR	Deamidated N30, N32 3.38%
33.5	4985.1783	2.0094	TQGNKQMLCTCLGNGVSCQETAVTQTYGGNSNGEPCVLPFTYNGR	
33.5	4986.1624	1.0253	TQGNKQMLCTCLGNGVSCQETAVTQTYGGNSNGEPCVLPFTYNGR	Deamidated Q25, N30 3.38%
33.5	4985.1783	2.0094	TQGNKQMLCTCLGNGVSCQETAVTQTYGGNSNGEPCVLPFTYNGR	
33.5	4986.1624	1.0253	TQGNKQMLCTCLGNGVSCQETAVTQTYGGNSNGEPCVLPFTYNGR	Deamidated Q19, Q25 3.38%
33.5	4985.1783	2.0094	TQGNKQMLCTCLGNGVSCQETAVTQTYGGNSNGEPCVLPFTYNGR	

33.5	4986.1624	1.0253	TQGNKQMLCTCLGNGVSCQETAVTQTYGGNSNGEPCVLPFTYNGR	Deamidated N14, Q19 3.38%

**Mascot:** http://www.matrixscience.com/

Mascot Search Results

Peptide View

MS/MS Fragmentation of **TQGNKQMLCTCLGNGVSCQETAVTQTYGGNSNGEPCVLPFTYNGR**
Found in **P02751** in **uni_human_nr**, FINC_HUMAN Fibronectin OS=Homo sapiens GN=FN1 PE=1 SV=4

Match to Query 20002: 4987.199896 from(1247.807250,4+) intensity(1689172.8750) rtinseconds(2692) scans(16187) index(12672)
Title: 130806_HPL_Human_Plaque_BR2_TR2_06_Spectrum034477_scans__16187_RTINSECONDS=2692
Data file E:\\RAW\\HumanPlaques\\mgf\\T\\1D\\T130806_HPL_Human_Plaque_BR2_TR2_06.mgf

Click mouse within plot area to zoom in by factor of two about that point
Or, [Plot from] 0 to 2200 Da [Full range]
Label all possible matches ○ Label matches used for scoring ⊙
Show Y-axis ☐

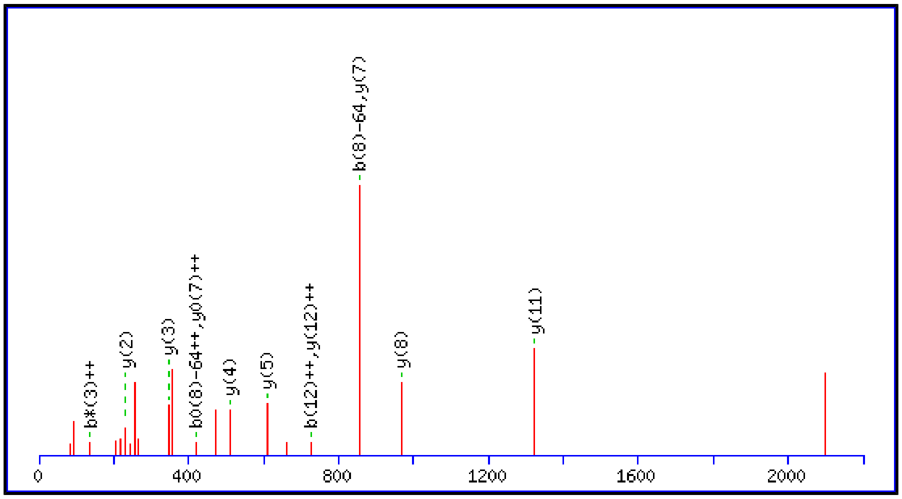

Monoisotopic mass of neutral peptide Mr(calc): 4985.1783
Fixed modifications: Carbamidomethyl (C) (apply to specified residues or termini only)
Variable modifications:
Q2 : Deamidated (NQ)
M7 : Oxidation (M), with neutral losses 63.9983(shown in table), 0.0000
Ions Score: 36 **Expect:** 0.0055
Matches : 13/782 fragment ions using 19 most intense peaks (help)

#	b	b^{++}	b*	b^{*++}	b^0	b^{0++}	Seq.	y	y^{++}	y*	y^{*++}	y^0	y^{0++}	#
1	102.0550	51.5311			84.0444	42.5258	T							45
2	231.0975	116.0524	214.0710	107.5391	213.0870	107.0471	Q	4821.1396	2411.0734	4804.1131	2402.5602	4803.1291	2402.0682	44
3	288.1190	144.5631	271.0925	136.0499	270.1084	135.5579	G	4692.0970	2346.5522	4675.0705	2338.0389	4674.0865	2337.5469	43
4	402.1619	201.5846	385.1354	193.0713	384.1514	192.5793	N	4635.0756	2318.0414	4618.0490	2309.5281	4617.0650	2309.0361	42
5	530.2569	265.6321	513.2304	257.1188	512.2463	256.6268	K	4521.0326	2261.0200	4504.0061	2252.5067	4503.0221	2252.0147	41
6	658.3155	329.6614	641.2889	321.1481	640.3049	320.6561	Q	4392.9377	2196.9725	4375.9111	2188.4592	4374.9271	2187.9672	40
7	741.3526	371.1799	724.3260	362.6667	723.3420	362.1747	M	4264.8791	2132.9432	4247.8525	2124.4299	4246.8685	2123.9379	39
8	854.4367	427.7220	837.4101	419.2087	836.4261	418.7167	L	4181.8420	2091.4246	4164.8154	2082.9114	4163.8314	2082.4193	38
9	1014.4673	507.7373	997.4408	499.2240	996.4567	498.7320	C	4068.7579	2034.8826	4051.7314	2026.3693	4050.7474	2025.8773	37
10	1115.5150	558.2611	1098.4884	549.7479	1097.5044	549.2558	T	3908.7273	1954.8673	3891.7007	1946.3540	3890.7167	1945.8620	36
11	1275.5456	638.2765	1258.5191	629.7632	1257.5351	629.2712	C	3807.6796	1904.3434	3790.6530	1895.8302	3789.6690	1895.3381	35
12	1388.6297	694.8185	1371.6032	686.3052	1370.6191	685.8132	L	3647.6489	1824.3281	3630.6224	1815.8148	3629.6384	1815.3228	34
13	1445.6512	723.3292	1428.6246	714.8159	1427.6406	714.3239	G	3534.5649	1767.7861	3517.5383	1759.2728	3516.5543	1758.7808	33
14	1559.6941	780.3507	1542.6675	771.8374	1541.6835	771.3454	N	3477.5434	1739.2753	3460.5169	1730.7621	3459.5328	1730.2701	32
15	1616.7156	808.8614	1599.6890	800.3481	1598.7050	799.8561	G	3363.5005	1682.2539	3346.4739	1673.7406	3345.4899	1673.2486	31
16	1715.7840	858.3956	1698.7574	849.8823	1697.7734	849.3903	V	3306.4790	1653.7431	3289.4525	1645.2299	3288.4685	1644.7379	30
17	1802.8160	901.9116	1785.7894	893.3984	1784.8054	892.9064	S	3207.4106	1604.2089	3190.3841	1595.6957	3189.4000	1595.2037	29

**18**	1962.8466	981.9270	1945.8201	973.4137	1944.8361	972.9217	**C**	3120.3786	1560.6929	3103.3520	1552.1797	3102.3680	1551.6876	**28**
**19**	2090.9052	1045.9563	2073.8787	1037.4430	2072.8947	1036.9510	**Q**	2960.3479	1480.6776	2943.3214	1472.1643	2942.3374	1471.6723	**27**
**20**	2219.9478	1110.4775	2202.9213	1101.9643	2201.9373	1101.4723	**E**	2832.2894	1416.6483	2815.2628	1408.1350	2814.2788	1407.6430	**26**
**21**	2320.9955	1161.0014	2303.9689	1152.4881	2302.9849	1151.9961	**T**	2703.2468	1352.1270	2686.2202	1343.6137	2685.2362	1343.1217	**25**
**22**	2392.0326	1196.5199	2375.0061	1188.0067	2374.0220	1187.5147	**A**	2602.1991	1301.6032	2585.1725	1293.0899	2584.1885	1292.5979	**24**
**23**	2491.1010	1246.0542	2474.0745	1237.5409	2473.0905	1237.0489	**V**	2531.1620	1266.0846	2514.1354	1257.5713	2513.1514	1257.0793	**23**
**24**	2592.1487	1296.5780	2575.1222	1288.0647	2574.1381	1287.5727	**T**	2432.0936	1216.5504	2415.0670	1208.0371	2414.0830	1207.5451	**22**
**25**	2720.2073	1360.6073	2703.1807	1352.0940	2702.1967	1351.6020	**Q**	2331.0459	1166.0266	2314.0193	1157.5133	2313.0353	1157.0213	**21**
**26**	2821.2550	1411.1311	2804.2284	1402.6178	2803.2444	1402.1258	**T**	2202.9873	1101.9973	2185.9607	1093.4840	2184.9767	1092.9920	**20**
**27**	2984.3183	1492.6628	2967.2917	1484.1495	2966.3077	1483.6575	**Y**	2101.9396	1051.4734	2084.9131	1042.9602	2083.9291	1042.4682	**19**
**28**	3041.3398	1521.1735	3024.3132	1512.6602	3023.3292	1512.1682	**G**	1938.8763	969.9418	1921.8497	961.4285	1920.8657	960.9365	**18**
**29**	3098.3612	1549.6842	3081.3347	1541.1710	3080.3507	1540.6790	**G**	1881.8548	941.4310	1864.8283	932.9178	1863.8443	932.4258	**17**
**30**	3212.4041	1606.7057	3195.3776	1598.1924	3194.3936	1597.7004	**N**	1824.8334	912.9203	1807.8068	904.4070	1806.8228	903.9150	**16**
**31**	3299.4362	1650.2217	3282.4096	1641.7084	3281.4256	1641.2164	**S**	1710.7904	855.8989	1693.7639	847.3856	1692.7799	846.8936	**15**
**32**	3413.4791	1707.2432	3396.4526	1698.7299	3395.4685	1698.2379	**N**	1623.7584	812.3828	1606.7319	803.8696	1605.7478	803.3776	**14**
**33**	3470.5006	1735.7539	3453.4740	1727.2406	3452.4900	1726.7486	**G**	1509.7155	755.3614	1492.6889	746.8481	1491.7049	746.3561	**13**
**34**	3599.5432	1800.2752	3582.5166	1791.7619	3581.5326	1791.2699	**E**	1452.6940	726.8506	1435.6675	718.3374	1434.6834	717.8454	**12**
**35**	3696.5959	1848.8016	3679.5694	1840.2883	3678.5854	1839.7963	**P**	***1323.6514***	662.3293	1306.6249	653.8161	1305.6409	653.3241	**11**
**36**	3856.6266	1928.8169	3839.6000	1920.3036	3838.6160	1919.8116	**C**	1226.5987	613.8030	1209.5721	605.2897	1208.5881	604.7977	**10**
**37**	3955.6950	1978.3511	3938.6684	1969.8379	3937.6844	1969.3458	**V**	1066.5680	533.7876	1049.5415	525.2744	1048.5574	524.7824	**9**
**38**	4068.7790	2034.8932	4051.7525	2026.3799	4050.7685	2025.8879	**L**	***967.4996***	484.2534	950.4730	475.7402	949.4890	475.2482	**8**
**39**	4165.8318	2083.4195	4148.8053	2074.9063	4147.8212	2074.4143	**P**	***854.4155***	427.7114	837.3890	419.1981	836.4050	418.7061	**7**
**40**	4312.9002	2156.9538	4295.8737	2148.4405	4294.8897	2147.9485	**F**	757.3628	379.1850	740.3362	370.6717	739.3522	370.1797	**6**
**41**	4413.9479	2207.4776	4396.9214	2198.9643	4395.9373	2198.4723	**T**	***610.2944***	305.6508	593.2678	297.1375	592.2838	296.6455	**5**
**42**	4577.0112	2289.0093	4559.9847	2280.4960	4559.0007	2280.0040	**Y**	***509.2467***	255.1270	492.2201	246.6137			**4**
**43**	4691.0542	2346.0307	4674.0276	2337.5174	4673.0436	2337.0254	**N**	***346.1833***	173.5953	329.1568	165.0820			**3**
**44**	4748.0756	2374.5415	4731.0491	2366.0282	4730.0651	2365.5362	**G**	***232.1404***	116.5738	215.1139	108.0606			**2**
**45**							**R**	175.1190	88.0631	158.0924	79.5498			**1**

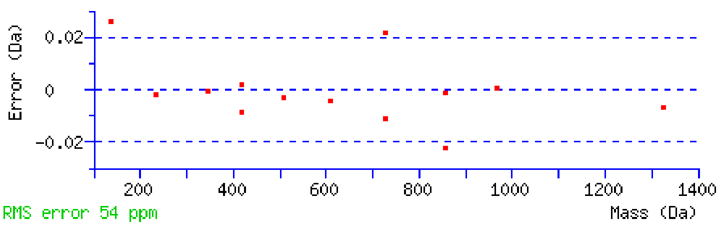

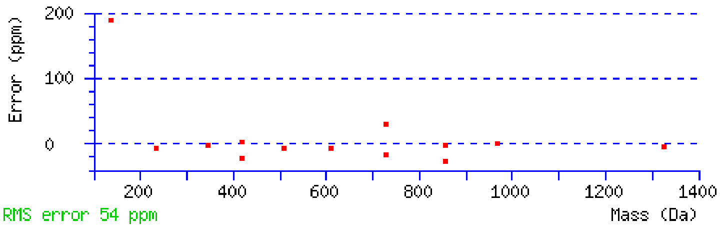

NCBI **BLAST** search of TQGNKQMLCTCLGNGVSCQETAVTQTYGGNSNGEPCVLPFTYNGR
(Parameters: blastp, nr protein database, expect=20000, no filter, PAM30)
Other BLAST web gateways

All matches to this query

Score	Mr(calc)	Delta	Sequence	Site Analysis
36.0	4985.1783	2.0216	TQGNKQMLCTCLGNGVSCQETAVTQTYGGNSNGEPCVLPFTYNGR	Deamidated Q2 18.92%
33.9	4985.1783	2.0216	TQGNKQMLCTCLGNGVSCQETAVTQTYGGNSNGEPCVLPFTYNGR	Deamidated N32 11.58%
33.9	4985.1783	2.0216	TQGNKQMLCTCLGNGVSCQETAVTQTYGGNSNGEPCVLPFTYNGR	Deamidated N30 11.58%
33.9	4985.1783	2.0216	TQGNKQMLCTCLGNGVSCQETAVTQTYGGNSNGEPCVLPFTYNGR	Deamidated Q25 11.58%
33.9	4985.1783	2.0216	TQGNKQMLCTCLGNGVSCQETAVTQTYGGNSNGEPCVLPFTYNGR	Deamidated Q19 11.58%
33.9	4985.1783	2.0216	TQGNKQMLCTCLGNGVSCQETAVTQTYGGNSNGEPCVLPFTYNGR	Deamidated N14 11.58%
33.9	4985.1783	2.0216	TQGNKQMLCTCLGNGVSCQETAVTQTYGGNSNGEPCVLPFTYNGR	Deamidated Q6 11.58%
33.9	4985.1783	2.0216	TQGNKQMLCTCLGNGVSCQETAVTQTYGGNSNGEPCVLPFTYNGR	Deamidated N4 11.58%
0.9	4985.1955	2.0044	HQQITENPQLMQNMLSAPYMRSMMQSLSQNPDLAAQLQEQMR	

0.9	4986.1795	1.0204	HQQITENPQLMQNMLSAPYMRSMMQSLSQNPDLAAQLQEQMR	

Mascot: http://www.matrixscience.com/

Mascot Search Results

Peptide View

MS/MS Fragmentation of **TQGNKQMLCTCLGNGVSCQETAVTQTYGGNSNGEPCVLPFTYNGR**
Found in **P02751** in **uni_human_nr**, FINC_HUMAN Fibronectin OS=Homo sapiens GN=FN1 PE=1 SV=4

Match to Query 20002: 4987.199896 from(1247.807250,4+) intensity(1689172.8750) rtinseconds(2692) scans(16187) index(12672)
Title: 130806_HPL_Human_Plaque_BR2_TR2_06_Spectrum034477_scans__16187_RTINSECONDS=2692
Data file E:\\RAW\\HumanPlaques\\mgf\\T\\1D\\T130806_HPL_Human_Plaque_BR2_TR2_06.mgf

Click mouse within plot area to zoom in by factor of two about that point
Or, [Plot from] [0] to [2200] Da [Full range]
Label all possible matches   Label matches used for scoring
Show Y-axis ☐

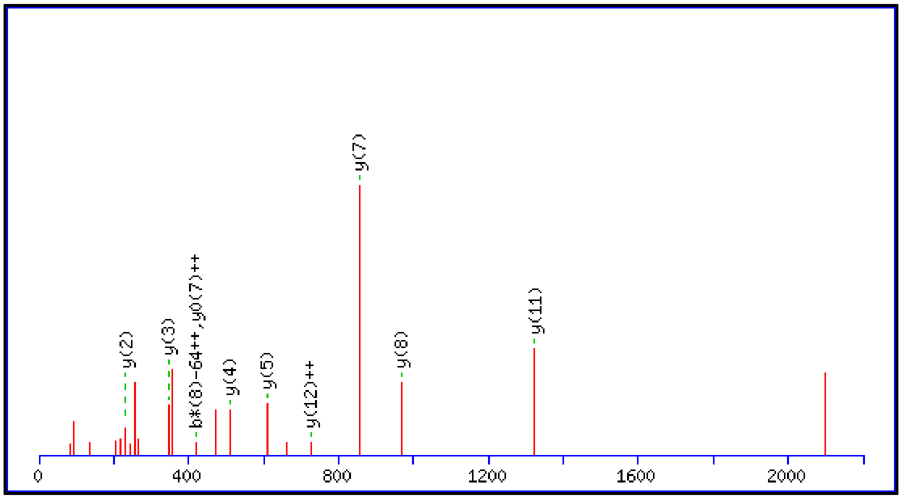

Monoisotopic mass of neutral peptide Mr(calc): 4985.1783
Fixed modifications: Carbamidomethyl (C) (apply to specified residues or termini only)
Variable modifications:
M7 : Oxidation (M), with neutral losses 0.0000(shown in table), 63.9983
N32 : Deamidated (NQ)
Ions Score: 34 **Expect:** 0.009
Matches : 10/782 fragment ions using 19 most intense peaks (help)

#	b	b^{++}	b*	b^{*++}	b^0	b^{0++}	Seq.	y	y^{++}	y*	y^{*++}	y^0	y^{0++}	#
1	102.0550	51.5311			84.0444	42.5258	T							45
2	230.1135	115.5604	213.0870	107.0471	212.1030	106.5551	Q	4885.1379	2443.0726	4868.1114	2434.5593	4867.1273	2434.0673	44
3	287.1350	144.0711	270.1084	135.5579	269.1244	135.0659	G	4757.0793	2379.0433	4740.0528	2370.5300	4739.0688	2370.0380	43
4	401.1779	201.0926	384.1514	192.5793	383.1674	192.0873	N	4700.0579	2350.5326	4683.0313	2342.0193	4682.0473	2341.5273	42
5	529.2729	265.1401	512.2463	256.6268	511.2623	256.1348	K	4586.0149	2293.5111	4568.9884	2284.9978	4568.0044	2284.5058	41
6	657.3315	329.1694	640.3049	320.6561	639.3209	320.1641	Q	4457.9200	2229.4636	4440.8934	2220.9504	4439.9094	2220.4583	40
7	804.3669	402.6871	787.3403	394.1738	786.3563	393.6818	M	4329.8614	2165.4343	4312.8348	2156.9211	4311.8508	2156.4291	39
8	917.4509	459.2291	900.4244	450.7158	899.4404	450.2238	L	4182.8260	2091.9166	4165.7994	2083.4034	4164.8154	2082.9114	38
9	1077.4816	539.2444	1060.4550	530.7312	1059.4710	530.2391	C	4069.7419	2035.3746	4052.7154	2026.8613	4051.7314	2026.3693	37
10	1178.5293	589.7683	1161.5027	581.2550	1160.5187	580.7630	T	3909.7113	1955.3593	3892.6847	1946.8460	3891.7007	1946.3540	36
11	1338.5599	669.7836	1321.5334	661.2703	1320.5493	660.7783	C	3808.6636	1904.8354	3791.6371	1896.3222	3790.6530	1895.8302	35
12	1451.6440	726.3256	1434.6174	717.8123	1433.6334	717.3203	L	3648.6330	1824.8201	3631.6064	1816.3068	3630.6224	1815.8148	34
13	1508.6654	754.8364	1491.6389	746.3231	1490.6549	745.8311	G	3535.5489	1768.2781	3518.5223	1759.7648	3517.5383	1759.2728	33
14	1622.7084	811.8578	1605.6818	803.3445	1604.6978	802.8525	N	3478.5274	1739.7674	3461.5009	1731.2541	3460.5169	1730.7621	32
15	1679.7298	840.3686	1662.7033	831.8553	1661.7193	831.3633	G	3364.4845	1682.7459	3347.4580	1674.2326	3346.4739	1673.7406	31
16	1778.7982	889.9028	1761.7717	881.3895	1760.7877	880.8975	V	3307.4630	1654.2352	3290.4365	1645.7219	3289.4525	1645.2299	30
17	1865.8303	933.4188	1848.8037	924.9055	1847.8197	924.4135	S	3208.3946	1604.7009	3191.3681	1596.1877	3190.3841	1595.6957	29

**18**	2025.8609	1013.4341	2008.8344	1004.9208	2007.8504	1004.4288	**C**	3121.3626	1561.1849	3104.3360	1552.6717	3103.3520	1552.1797	**28**
**19**	2153.9195	1077.4634	2136.8929	1068.9501	2135.9089	1068.4581	**Q**	2961.3319	1481.1696	2944.3054	1472.6563	2943.3214	1472.1643	**27**
**20**	2282.9621	1141.9847	2265.9355	1133.4714	2264.9515	1132.9794	**E**	2833.2734	1417.1403	2816.2468	1408.6270	2815.2628	1408.1350	**26**
**21**	2384.0098	1192.5085	2366.9832	1183.9952	2365.9992	1183.5032	**T**	2704.2308	1352.6190	2687.2042	1344.1058	2686.2202	1343.6137	**25**
**22**	2455.0469	1228.0271	2438.0203	1219.5138	2437.0363	1219.0218	**A**	2603.1831	1302.0952	2586.1565	1293.5819	2585.1725	1293.0899	**24**
**23**	2554.1153	1277.5613	2537.0887	1269.0480	2536.1047	1268.5560	**V**	2532.1460	1266.5766	2515.1194	1258.0634	2514.1354	1257.5713	**23**
**24**	2655.1630	1328.0851	2638.1364	1319.5718	2637.1524	1319.0798	**T**	2433.0776	1217.0424	2416.0510	1208.5291	2415.0670	1208.0371	**22**
**25**	2783.2216	1392.1144	2766.1950	1383.6011	2765.2110	1383.1091	**Q**	2332.0299	1166.5186	2315.0033	1158.0053	2314.0193	1157.5133	**21**
**26**	2884.2692	1442.6383	2867.2427	1434.1250	2866.2587	1433.6330	**T**	2203.9713	1102.4893	2186.9448	1093.9760	2185.9607	1093.4840	**20**
**27**	3047.3326	1524.1699	3030.3060	1515.6566	3029.3220	1515.1646	**Y**	2102.9236	1051.9655	2085.8971	1043.4522	2084.9131	1042.9602	**19**
**28**	3104.3540	1552.6806	3087.3275	1544.1674	3086.3435	1543.6754	**G**	1939.8603	970.4338	1922.8338	961.9205	1921.8497	961.4285	**18**
**29**	3161.3755	1581.1914	3144.3489	1572.6781	3143.3649	1572.1861	**G**	1882.8388	941.9231	1865.8123	933.4098	1864.8283	932.9178	**17**
**30**	3275.4184	1638.2128	3258.3919	1629.6996	3257.4078	1629.2076	**N**	1825.8174	913.4123	1808.7908	904.8991	1807.8068	904.4070	**16**
**31**	3362.4504	1681.7289	3345.4239	1673.2156	3344.4399	1672.7236	**S**	1711.7744	856.3909	1694.7479	847.8776	1693.7639	847.3856	**15**
**32**	3477.4774	1739.2423	3460.4508	1730.7291	3459.4668	1730.2370	**N**	1624.7424	812.8748	1607.7159	804.3616	1606.7319	803.8696	**14**
**33**	3534.4988	1767.7531	3517.4723	1759.2398	3516.4883	1758.7478	**G**	1509.7155	755.3614	1492.6889	746.8481	1491.7049	746.3561	**13**
**34**	3663.5414	1832.2744	3646.5149	1823.7611	3645.5309	1823.2691	**E**	1452.6940	726.8506	1435.6675	718.3374	1434.6834	717.8454	**12**
**35**	3760.5942	1880.8007	3743.5677	1872.2875	3742.5836	1871.7955	**P**	***1323.6514***	662.3293	1306.6249	653.8161	1305.6409	653.3241	**11**
**36**	3920.6249	1960.8161	3903.5983	1952.3028	3902.6143	1951.8108	**C**	1226.5987	613.8030	1209.5721	605.2897	1208.5881	604.7977	**10**
**37**	4019.6933	2010.3503	4002.6667	2001.8370	4001.6827	2001.3450	**V**	1066.5680	533.7876	1049.5415	525.2744	1048.5574	524.7824	**9**
**38**	4132.7773	2066.8923	4115.7508	2058.3790	4114.7668	2057.8870	**L**	***967.4996***	484.2534	950.4730	475.7402	949.4890	475.2482	**8**
**39**	4229.8301	2115.4187	4212.8035	2106.9054	4211.8195	2106.4134	**P**	***854.4155***	427.7114	837.3890	419.1981	836.4050	418.7061	**7**
**40**	4376.8985	2188.9529	4359.8720	2180.4396	4358.8879	2179.9476	**F**	757.3628	379.1850	740.3362	370.6717	739.3522	370.1797	**6**
**41**	4477.9462	2239.4767	4460.9196	2230.9635	4459.9356	2230.4715	**T**	***610.2944***	305.6508	593.2678	297.1375	592.2838	296.6455	**5**
**42**	4641.0095	2321.0084	4623.9830	2312.4951	4622.9990	2312.0031	**Y**	***509.2467***	255.1270	492.2201	246.6137			**4**
**43**	4755.0524	2378.0299	4738.0259	2369.5166	4737.0419	2369.0246	**N**	***346.1833***	173.5953	329.1568	165.0820			**3**
**44**	4812.0739	2406.5406	4795.0474	2398.0273	4794.0633	2397.5353	**G**	***232.1404***	116.5738	215.1139	108.0606			**2**
**45**							**R**	175.1190	88.0631	158.0924	79.5498			**1**

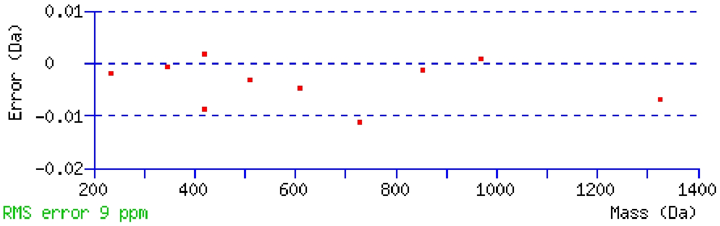

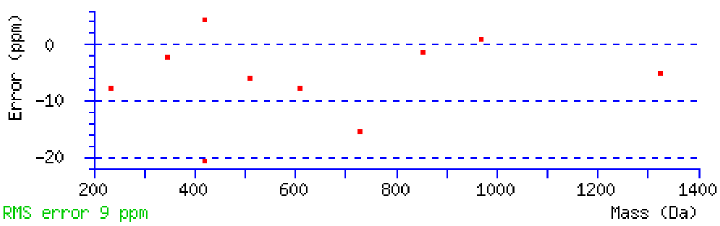

NCBI **BLAST** search of TQGNKQMLCTCLGNGVSCQETAVTQTYGGNSNGEPCVLPFTYNGR
(Parameters: blastp, nr protein database, expect=20000, no filter, PAM30)
Other BLAST web gateways

All matches to this query

Score	Mr(calc)	Delta	Sequence	Site Analysis
33.9	4985.1783	2.0216	TQGNKQMLCTCLGNGVSCQETAVTQTYGGNSNGEPCVLPFTYNGR	Deamidated N32 12.50%
33.9	4985.1783	2.0216	TQGNKQMLCTCLGNGVSCQETAVTQTYGGNSNGEPCVLPFTYNGR	Deamidated N30 12.50%
33.9	4985.1783	2.0216	TQGNKQMLCTCLGNGVSCQETAVTQTYGGNSNGEPCVLPFTYNGR	Deamidated Q25 12.50%
33.9	4985.1783	2.0216	TQGNKQMLCTCLGNGVSCQETAVTQTYGGNSNGEPCVLPFTYNGR	Deamidated Q19 12.50%
33.9	4985.1783	2.0216	TQGNKQMLCTCLGNGVSCQETAVTQTYGGNSNGEPCVLPFTYNGR	Deamidated N14 12.50%
33.9	4985.1783	2.0216	TQGNKQMLCTCLGNGVSCQETAVTQTYGGNSNGEPCVLPFTYNGR	Deamidated Q6 12.50%
33.9	4985.1783	2.0216	TQGNKQMLCTCLGNGVSCQETAVTQTYGGNSNGEPCVLPFTYNGR	Deamidated N4 12.50%
33.9	4985.1783	2.0216	TQGNKQMLCTCLGNGVSCQETAVTQTYGGNSNGEPCVLPFTYNGR	Deamidated Q2 12.50%
0.9	4985.1955	2.0044	HQQITENPQLMQNMLSAPYMRSMMQSLSQNPDLAAQLQEQMR	

0.9	4986.1795	1.0204	HQQITENPQLMQNMLSAPYMRSMMQSLSQNPDLAAQLQEQMR	

**Mascot:** http://www.matrixscience.com/

Mascot Search Results

Peptide View

MS/MS Fragmentation of **TQGNKQMLCTCLGNGVSCQETAVTQTYGGNSNGEPCVLPFTYNGR**
Found in **P02751** in **uni_human_nr**, FINC_HUMAN Fibronectin OS=Homo sapiens GN=FN1 PE=1 SV=4

Match to Query 19840: 4984.190656 from(1247.054940,4+) intensity(0.0000) rtinseconds(2672) scans(15844) index(12088)
Title: 130801_HPL_Human_Plaque_BR1_TR1_07_Spectrum033791_scans__15844_RTINSECONDS=2672
Data file E:\\RAW\\HumanPlaques\\mgf\\T\\1D\\T130801_HPL_Human_Plaque_BR1_TR1_07.mgf

Click mouse within plot area to zoom in by factor of two about that point
Or, Plot from 0 to 1400 Da Full range
Label all possible matches ○ Label matches used for scoring ⊙
Show Y-axis ☐

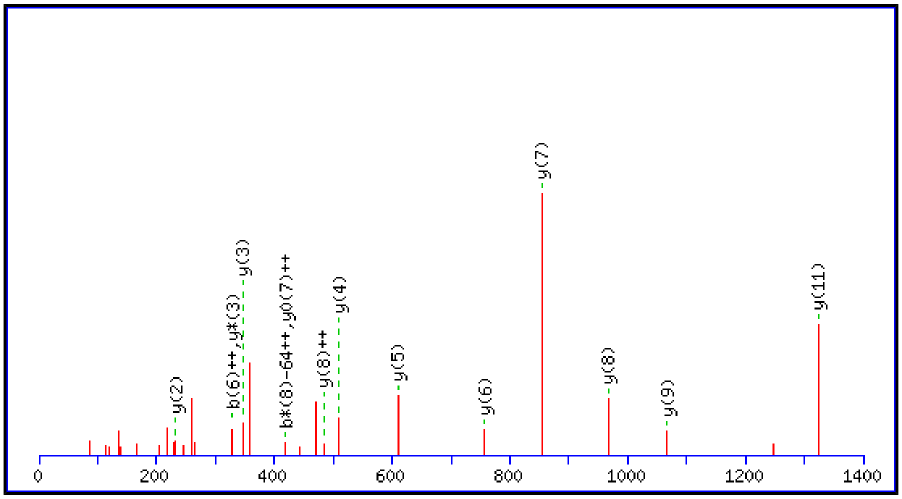

Monoisotopic mass of neutral peptide Mr(calc): 4984.1943
Fixed modifications: Carbamidomethyl (C) (apply to specified residues or termini only)
Variable modifications:
M7 : Oxidation (M), with neutral losses 0.0000(shown in table), 63.9983
Ions Score: 47 **Expect:** 0.00051
Matches : 14/782 fragment ions using 22 most intense peaks (help)

#	b	b^{++}	b*	b^{*++}	b^0	b^{0++}	Seq.	y	y^{++}	y*	y^{*++}	y^0	y^{0++}	#
1	102.0550	51.5311			84.0444	42.5258	T							45
2	230.1135	115.5604	213.0870	107.0471	212.1030	106.5551	Q	4884.1539	2442.5806	4867.1273	2434.0673	4866.1433	2433.5753	44
3	287.1350	144.0711	270.1084	135.5579	269.1244	135.0659	G	4756.0953	2378.5513	4739.0688	2370.0380	4738.0847	2369.5460	43
4	401.1779	201.0926	384.1514	192.5793	383.1674	192.0873	N	4699.0738	2350.0406	4682.0473	2341.5273	4681.0633	2341.0353	42
5	529.2729	265.1401	512.2463	256.6268	511.2623	256.1348	K	4585.0309	2293.0191	4568.0044	2284.5058	4567.0204	2284.0138	41
6	657.3315	**329.1694**	640.3049	320.6561	639.3209	320.1641	Q	4456.9360	2228.9716	4439.9094	2220.4583	4438.9254	2219.9663	40
7	804.3669	402.6871	787.3403	394.1738	786.3563	393.6818	M	4328.8774	2164.9423	4311.8508	2156.4291	4310.8668	2155.9370	39
8	917.4509	459.2291	900.4244	450.7158	899.4404	450.2238	L	4181.8420	2091.4246	4164.8154	2082.9114	4163.8314	2082.4193	38
9	1077.4816	539.2444	1060.4550	530.7312	1059.4710	530.2391	C	4068.7579	2034.8826	4051.7314	2026.3693	4050.7474	2025.8773	37
10	1178.5293	589.7683	1161.5027	581.2550	1160.5187	580.7630	T	3908.7273	1954.8673	3891.7007	1946.3540	3890.7167	1945.8620	36
11	1338.5599	669.7836	1321.5334	661.2703	1320.5493	660.7783	C	3807.6796	1904.3434	3790.6530	1895.8302	3789.6690	1895.3381	35
12	1451.6440	726.3256	1434.6174	717.8123	1433.6334	717.3203	L	3647.6489	1824.3281	3630.6224	1815.8148	3629.6384	1815.3228	34
13	1508.6654	754.8364	1491.6389	746.3231	1490.6549	745.8311	G	3534.5649	1767.7861	3517.5383	1759.2728	3516.5543	1758.7808	33
14	1622.7084	811.8578	1605.6818	803.3445	1604.6978	802.8525	N	3477.5434	1739.2753	3460.5169	1730.7621	3459.5328	1730.2701	32
15	1679.7298	840.3686	1662.7033	831.8553	1661.7193	831.3633	G	3363.5005	1682.2539	3346.4739	1673.7406	3345.4899	1673.2486	31
16	1778.7982	889.9028	1761.7717	881.3895	1760.7877	880.8975	V	3306.4790	1653.7431	3289.4525	1645.2299	3288.4685	1644.7379	30
17	1865.8303	933.4188	1848.8037	924.9055	1847.8197	924.4135	S	3207.4106	1604.2089	3190.3841	1595.6957	3189.4000	1595.2037	29
18	2025.8609	1013.4341	2008.8344	1004.9208	2007.8504	1004.4288	C	3120.3786	1560.6929	3103.3520	1552.1797	3102.3680	1551.6876	28

**19**	2153.9195	1077.4634	2136.8929	1068.9501	2135.9089	1068.4581	**Q**	2960.3479	1480.6776	2943.3214	1472.1643	2942.3374	1471.6723	**27**
**20**	2282.9621	1141.9847	2265.9355	1133.4714	2264.9515	1132.9794	**E**	2832.2894	1416.6483	2815.2628	1408.1350	2814.2788	1407.6430	**26**
**21**	2384.0098	1192.5085	2366.9832	1183.9952	2365.9992	1183.5032	**T**	2703.2468	1352.1270	2686.2202	1343.6137	2685.2362	1343.1217	**25**
**22**	2455.0469	1228.0271	2438.0203	1219.5138	2437.0363	1219.0218	**A**	2602.1991	1301.6032	2585.1725	1293.0899	2584.1885	1292.5979	**24**
**23**	2554.1153	1277.5613	2537.0887	1269.0480	2536.1047	1268.5560	**V**	2531.1620	1266.0846	2514.1354	1257.5713	2513.1514	1257.0793	**23**
**24**	2655.1630	1328.0851	2638.1364	1319.5718	2637.1524	1319.0798	**T**	2432.0936	1216.5504	2415.0670	1208.0371	2414.0830	1207.5451	**22**
**25**	2783.2216	1392.1144	2766.1950	1383.6011	2765.2110	1383.1091	**Q**	2331.0459	1166.0266	2314.0193	1157.5133	2313.0353	1157.0213	**21**
**26**	2884.2692	1442.6383	2867.2427	1434.1250	2866.2587	1433.6330	**T**	2202.9873	1101.9973	2185.9607	1093.4840	2184.9767	1092.9920	**20**
**27**	3047.3326	1524.1699	3030.3060	1515.6566	3029.3220	1515.1646	**Y**	2101.9396	1051.4734	2084.9131	1042.9602	2083.9291	1042.4682	**19**
**28**	3104.3540	1552.6806	3087.3275	1544.1674	3086.3435	1543.6754	**G**	1938.8763	969.9418	1921.8497	961.4285	1920.8657	960.9365	**18**
**29**	3161.3755	1581.1914	3144.3489	1572.6781	3143.3649	1572.1861	**G**	1881.8548	941.4310	1864.8283	932.9178	1863.8443	932.4258	**17**
**30**	3275.4184	1638.2128	3258.3919	1629.6996	3257.4078	1629.2076	**N**	1824.8334	912.9203	1807.8068	904.4070	1806.8228	903.9150	**16**
**31**	3362.4504	1681.7289	3345.4239	1673.2156	3344.4399	1672.7236	**S**	1710.7904	855.8989	1693.7639	847.3856	1692.7799	846.8936	**15**
**32**	3476.4934	1738.7503	3459.4668	1730.2370	3458.4828	1729.7450	**N**	1623.7584	812.3828	1606.7319	803.8696	1605.7478	803.3776	**14**
**33**	3533.5148	1767.2611	3516.4883	1758.7478	3515.5043	1758.2558	**G**	1509.7155	755.3614	1492.6889	746.8481	1491.7049	746.3561	**13**
**34**	3662.5574	1831.7824	3645.5309	1823.2691	3644.5469	1822.7771	**E**	1452.6940	726.8506	1435.6675	718.3374	1434.6834	717.8454	**12**
**35**	3759.6102	1880.3087	3742.5836	1871.7955	3741.5996	1871.3035	**P**	***1323.6514***	662.3293	1306.6249	653.8161	1305.6409	653.3241	**11**
**36**	3919.6408	1960.3241	3902.6143	1951.8108	3901.6303	1951.3188	**C**	1226.5987	613.8030	1209.5721	605.2897	1208.5881	604.7977	**10**
**37**	4018.7093	2009.8583	4001.6827	2001.3450	4000.6987	2000.8530	**V**	***1066.5680***	533.7876	1049.5415	525.2744	1048.5574	524.7824	**9**
**38**	4131.7933	2066.4003	4114.7668	2057.8870	4113.7828	2057.3950	**L**	***967.4996***	484.2534	950.4730	475.7402	949.4890	475.2482	**8**
**39**	4228.8461	2114.9267	4211.8195	2106.4134	4210.8355	2105.9214	**P**	***854.4155***	427.7114	837.3890	419.1981	836.4050	418.7061	**7**
**40**	4375.9145	2188.4609	4358.8879	2179.9476	4357.9039	2179.4556	**F**	***757.3628***	379.1850	740.3362	370.6717	739.3522	370.1797	**6**
**41**	4476.9622	2238.9847	4459.9356	2230.4715	4458.9516	2229.9794	**T**	***610.2944***	305.6508	593.2678	297.1375	592.2838	296.6455	**5**
**42**	4640.0255	2320.5164	4622.9990	2312.0031	4622.0149	2311.5111	**Y**	***509.2467***	255.1270	492.2201	246.6137			**4**
**43**	4754.0684	2377.5379	4737.0419	2369.0246	4736.0579	2368.5326	**N**	***346.1833***	173.5953	329.1568	165.0820			**3**
**44**	4811.0899	2406.0486	4794.0633	2397.5353	4793.0793	2397.0433	**G**	***232.1404***	116.5738	215.1139	108.0606			**2**
**45**							**R**	175.1190	88.0631	158.0924	79.5498			**1**

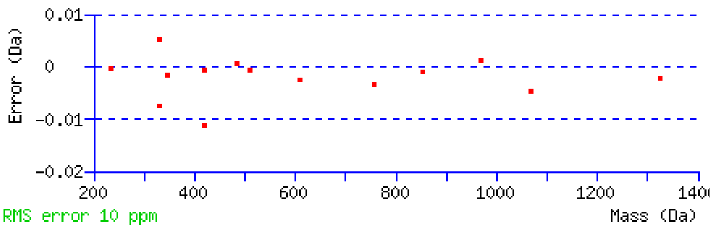

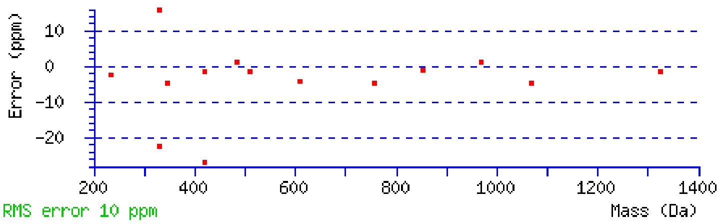

NCBI **BLAST** search of TQGNKQMLCTCLGNGVSCQETAVTQTYGGNSNGEPCVLPFTYNGR
(Parameters: blastp, nr protein database, expect=20000, no filter, PAM30)
Other BLAST web gateways

All matches to this query

Score	Mr(calc)	Delta	Sequence
47.2	4984.1943	-0.0037	TQGNKQMLCTCLGNGVSCQETAVTQTYGGNSNGEPCVLPFTYNGR

Mascot: http://www.matrixscience.com/

Mascot Search Results

Peptide View

MS/MS Fragmentation of **TQGNKQMLCTCLGNGVSCQETAVTQTYGGNSNGEPCVLPFTYNGR**
Found in **P02751** in **uni_human_nr**, FINC_HUMAN Fibronectin OS=Homo sapiens GN=FN1 PE=1 SV=4

Match to Query 19986: 4971.202336 from(1243.807860,4+) intensity(1117394.6250) rtinseconds(2778) scans(16785) index(13164)
Title: 130806_HPL_Human_Plaque_BR2_TR2_06_Spectrum034969_scans__16785_RTINSECONDS=2778
Data file E:\\RAW\\HumanPlaques\\mgf\\T\\1D\\T130806_HPL_Human_Plaque_BR2_TR2_06.mgf

Click mouse within plot area to zoom in by factor of two about that point
Or, Plot from 0 to 1400 Da Full range
Label all possible matches ○ Label matches used for scoring ⊙
Show Y-axis ☐

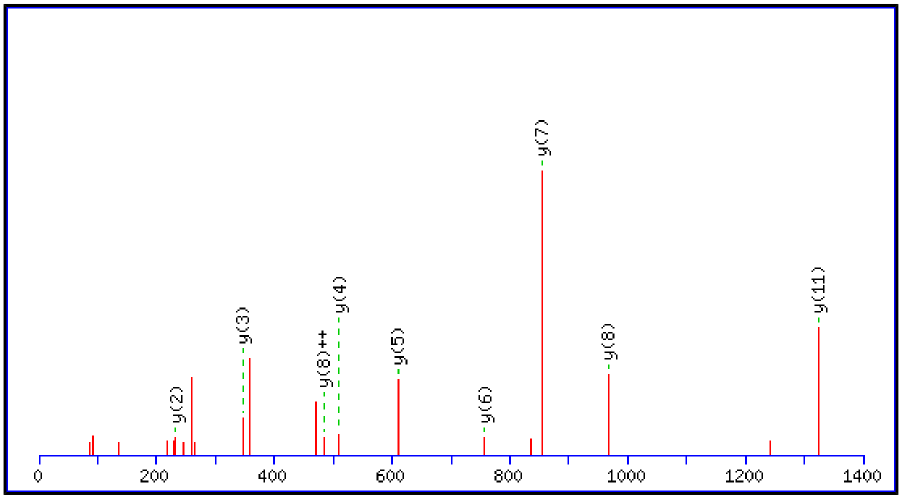

Monoisotopic mass of neutral peptide Mr(calc): 4969.1834
Fixed modifications: Carbamidomethyl (C) (apply to specified residues or termini only)
Variable modifications:
N32 : Deamidated (NQ)
Ions Score: 43 **Expect:** 0.0012
Matches : 9/518 fragment ions using 17 most intense peaks (help)

#	b	b^{++}	b*	b^{*++}	b^0	b^{0++}	Seq.	y	y^{++}	y*	y^{*++}	y^0	y^{0++}	#
1	102.0550	51.5311			84.0444	42.5258	T							45
2	230.1135	115.5604	213.0870	107.0471	212.1030	106.5551	Q	4869.1430	2435.0751	4852.1164	2426.5619	4851.1324	2426.0699	44
3	287.1350	144.0711	270.1084	135.5579	269.1244	135.0659	G	4741.0844	2371.0458	4724.0579	2362.5326	4723.0738	2362.0406	43
4	401.1779	201.0926	384.1514	192.5793	383.1674	192.0873	N	4684.0630	2342.5351	4667.0364	2334.0218	4666.0524	2333.5298	42
5	529.2729	265.1401	512.2463	256.6268	511.2623	256.1348	K	4570.0200	2285.5136	4552.9935	2277.0004	4552.0095	2276.5084	41
6	657.3315	329.1694	640.3049	320.6561	639.3209	320.1641	Q	4441.9251	2221.4662	4424.8985	2212.9529	4423.9145	2212.4609	40
7	788.3720	394.6896	771.3454	386.1763	770.3614	385.6843	M	4313.8665	2157.4369	4296.8399	2148.9236	4295.8559	2148.4316	39
8	901.4560	451.2316	884.4295	442.7184	883.4454	442.2264	L	4182.8260	2091.9166	4165.7994	2083.4034	4164.8154	2082.9114	38
9	1061.4867	531.2470	1044.4601	522.7337	1043.4761	522.2417	C	4069.7419	2035.3746	4052.7154	2026.8613	4051.7314	2026.3693	37
10	1162.5343	581.7708	1145.5078	573.2575	1144.5238	572.7655	T	3909.7113	1955.3593	3892.6847	1946.8460	3891.7007	1946.3540	36
11	1322.5650	661.7861	1305.5384	653.2729	1304.5544	652.7809	C	3808.6636	1904.8354	3791.6371	1896.3222	3790.6530	1895.8302	35
12	1435.6491	718.3282	1418.6225	709.8149	1417.6385	709.3229	L	3648.6330	1824.8201	3631.6064	1816.3068	3630.6224	1815.8148	34
13	1492.6705	746.8389	1475.6440	738.3256	1474.6600	737.8336	G	3535.5489	1768.2781	3518.5223	1759.7648	3517.5383	1759.2728	33
14	1606.7134	803.8604	1589.6869	795.3471	1588.7029	794.8551	N	3478.5274	1739.7674	3461.5009	1731.2541	3460.5169	1730.7621	32
15	1663.7349	832.3711	1646.7084	823.8578	1645.7243	823.3658	G	3364.4845	1682.7459	3347.4580	1674.2326	3346.4739	1673.7406	31
16	1762.8033	881.9053	1745.7768	873.3920	1744.7928	872.9000	V	3307.4630	1654.2352	3290.4365	1645.7219	3289.4525	1645.2299	30
17	1849.8354	925.4213	1832.8088	916.9080	1831.8248	916.4160	S	3208.3946	1604.7009	3191.3681	1596.1877	3190.3841	1595.6957	29
18	2009.8660	1005.4366	1992.8395	996.9234	1991.8554	996.4314	C	3121.3626	1561.1849	3104.3360	1552.6717	3103.3520	1552.1797	28

19	2137.9246	1069.4659	2120.8980	1060.9527	2119.9140	1060.4606	Q	2961.3319	1481.1696	2944.3054	1472.6563	2943.3214	1472.1643	27
20	2266.9672	1133.9872	2249.9406	1125.4739	2248.9566	1124.9819	E	2833.2734	1417.1403	2816.2468	1408.6270	2815.2628	1408.1350	26
21	2368.0149	1184.5111	2350.9883	1175.9978	2350.0043	1175.5058	T	2704.2308	1352.6190	2687.2042	1344.1058	2686.2202	1343.6137	25
22	2439.0520	1220.0296	2422.0254	1211.5163	2421.0414	1211.0243	A	2603.1831	1302.0952	2586.1565	1293.5819	2585.1725	1293.0899	24
23	2538.1204	1269.5638	2521.0938	1261.0506	2520.1098	1260.5585	V	2532.1460	1266.5766	2515.1194	1258.0634	2514.1354	1257.5713	23
24	2639.1681	1320.0877	2622.1415	1311.5744	2621.1575	1311.0824	T	2433.0776	1217.0424	2416.0510	1208.5291	2415.0670	1208.0371	22
25	2767.2266	1384.1170	2750.2001	1375.6037	2749.2161	1375.1117	Q	2332.0299	1166.5186	2315.0033	1158.0053	2314.0193	1157.5133	21
26	2868.2743	1434.6408	2851.2478	1426.1275	2850.2637	1425.6355	T	2203.9713	1102.4893	2186.9448	1093.9760	2185.9607	1093.4840	20
27	3031.3376	1516.1725	3014.3111	1507.6592	3013.3271	1507.1672	Y	2102.9236	1051.9655	2085.8971	1043.4522	2084.9131	1042.9602	19
28	3088.3591	1544.6832	3071.3326	1536.1699	3070.3485	1535.6779	G	1939.8603	970.4338	1922.8338	961.9205	1921.8497	961.4285	18
29	3145.3806	1573.1939	3128.3540	1564.6806	3127.3700	1564.1886	G	1882.8388	941.9231	1865.8123	933.4098	1864.8283	932.9178	17
30	3259.4235	1630.2154	3242.3969	1621.7021	3241.4129	1621.2101	N	1825.8174	913.4123	1808.7908	904.8991	1807.8068	904.4070	16
31	3346.4555	1673.7314	3329.4290	1665.2181	3328.4450	1664.7261	S	1711.7744	856.3909	1694.7479	847.8776	1693.7639	847.3856	15
32	3461.4825	1731.2449	3444.4559	1722.7316	3443.4719	1722.2396	N	1624.7424	812.8748	1607.7159	804.3616	1606.7319	803.8696	14
33	3518.5039	1759.7556	3501.4774	1751.2423	3500.4934	1750.7503	G	1509.7155	755.3614	1492.6889	746.8481	1491.7049	746.3561	13
34	3647.5465	1824.2769	3630.5200	1815.7636	3629.5360	1815.2716	E	1452.6940	726.8506	1435.6675	718.3374	1434.6834	717.8454	12
35	3744.5993	1872.8033	3727.5727	1864.2900	3726.5887	1863.7980	P	***1323.6514***	662.3293	1306.6249	653.8161	1305.6409	653.3241	11
36	3904.6299	1952.8186	3887.6034	1944.3053	3886.6194	1943.8133	C	1226.5987	613.8030	1209.5721	605.2897	1208.5881	604.7977	10
37	4003.6984	2002.3528	3986.6718	1993.8395	3985.6878	1993.3475	V	1066.5680	533.7876	1049.5415	525.2744	1048.5574	524.7824	9
38	4116.7824	2058.8948	4099.7559	2050.3816	4098.7719	2049.8896	L	***967.4996***	484.2534	950.4730	475.7402	949.4890	475.2482	8
39	4213.8352	2107.4212	4196.8086	2098.9080	4195.8246	2098.4159	P	***854.4155***	427.7114	837.3890	419.1981	836.4050	418.7061	7
40	4360.9036	2180.9554	4343.8770	2172.4422	4342.8930	2171.9502	F	***757.3628***	379.1850	740.3362	370.6717	739.3522	370.1797	6
41	4461.9513	2231.4793	4444.9247	2222.9660	4443.9407	2222.4740	T	***610.2944***	305.6508	593.2678	297.1375	592.2838	296.6455	5
42	4625.0146	2313.0109	4607.9881	2304.4977	4607.0040	2304.0057	Y	***509.2467***	255.1270	492.2201	246.6137			4
43	4739.0575	2370.0324	4722.0310	2361.5191	4721.0470	2361.0271	N	***346.1833***	173.5953	329.1568	165.0820			3
44	4796.0790	2398.5431	4779.0524	2390.0299	4778.0684	2389.5379	G	***232.1404***	116.5738	215.1139	108.0606			2
45							R	175.1190	88.0631	158.0924	79.5498			1

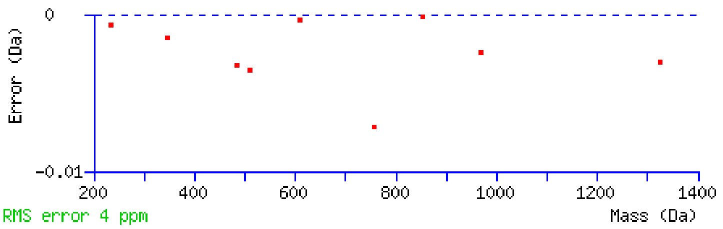

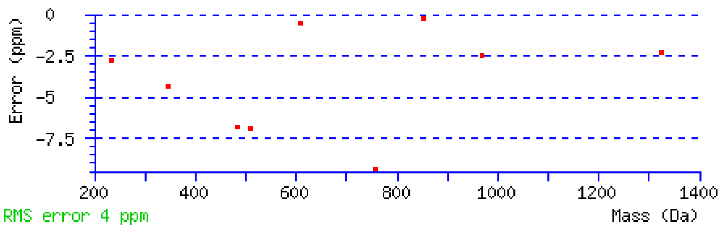

NCBI **BLAST** search of TQGNKQMLCTCLGNGVSCQETAVTQTYGGNSNGEPCVLPFTYNGR
(Parameters: blastp, nr protein database, expect=20000, no filter, PAM30)
Other BLAST web gateways

All matches to this query

Score	Mr(calc)	Delta	Sequence	Site Analysis
43.0	4969.1834	2.0189	TQGNKQMLCTCLGNGVSCQETAVTQTYGGNSNGEPCVLPFTYNGR	Deamidated N32 12.50%
43.0	4969.1834	2.0189	TQGNKQMLCTCLGNGVSCQETAVTQTYGGNSNGEPCVLPFTYNGR	Deamidated N30 12.50%
43.0	4969.1834	2.0189	TQGNKQMLCTCLGNGVSCQETAVTQTYGGNSNGEPCVLPFTYNGR	Deamidated Q25 12.50%
43.0	4969.1834	2.0189	TQGNKQMLCTCLGNGVSCQETAVTQTYGGNSNGEPCVLPFTYNGR	Deamidated Q19 12.50%
43.0	4969.1834	2.0189	TQGNKQMLCTCLGNGVSCQETAVTQTYGGNSNGEPCVLPFTYNGR	Deamidated N14 12.50%
43.0	4969.1834	2.0189	TQGNKQMLCTCLGNGVSCQETAVTQTYGGNSNGEPCVLPFTYNGR	Deamidated Q6 12.50%
43.0	4969.1834	2.0189	TQGNKQMLCTCLGNGVSCQETAVTQTYGGNSNGEPCVLPFTYNGR	Deamidated N4 12.50%
43.0	4969.1834	2.0189	TQGNKQMLCTCLGNGVSCQETAVTQTYGGNSNGEPCVLPFTYNGR	Deamidated Q2 12.50%
1.0	4970.1993	1.0031	YDFAIMIPPGGTECFWQFAHQTGYFYFSYEVQRTVGMSHDR	
1.0	4971.1833	0.0191	YDFAIMIPPGGTECFWQFAHQTGYFYFSYEVQRTVGMSHDR	

Mascot: http://www.matrixscience.com/

Mascot Search Results

Peptide View

MS/MS Fragmentation of **TQGNKQMLCTCLGNGVSCQETAVTQTYGGNSNGEPCVLPFTYNGR**
Found in **P02751** in **uni_human_nr**, FINC_HUMAN Fibronectin OS=Homo sapiens GN=FN1 PE=1 SV=4

Match to Query 20586: 4968.206256 from(1243.058840,4+) intensity(1150345.1250) rtinseconds(2770) scans(16961) index(13382)
Title: 130806_HPL_Human_Plaque_BR1_TR2_06_Spectrum035724_scans__16961_RTINSECONDS=2770
Data file E:\\RAW\\HumanPlaques\\mgf\\T\\1D\\T130806_HPL_Human_Plaque_BR1_TR2_06.mgf

Click mouse within plot area to zoom in by factor of two about that point
Or, Plot from 0 to 3200 Da Full range
Label all possible matches ○ Label matches used for scoring ⊙
Show Y-axis ☐

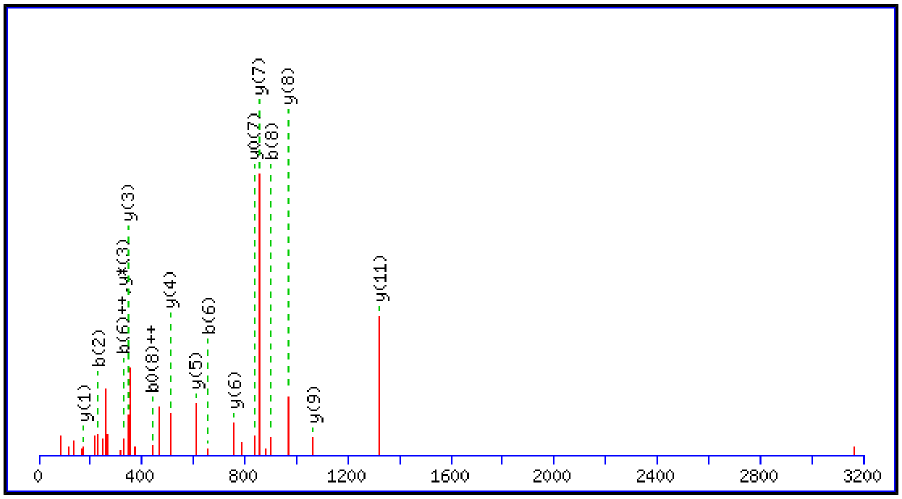

Monoisotopic mass of neutral peptide Mr(calc): 4968.1994
Fixed modifications: Carbamidomethyl (C) (apply to specified residues or termini only)
Ions Score: 51 **Expect:** 0.00023
Matches : 16/518 fragment ions using 25 most intense peaks (help)

#	b	b^{++}	b*	b^{*++}	b^{0}	b^{0++}	Seq.	y	y^{++}	y*	y^{*++}	y^{0}	y^{0++}	#
1	102.0550	51.5311			84.0444	42.5258	T							45
2	230.1135	115.5604	213.0870	107.0471	212.1030	106.5551	Q	4868.1590	2434.5831	4851.1324	2426.0699	4850.1484	2425.5778	44
3	287.1350	144.0711	270.1084	135.5579	269.1244	135.0659	G	4740.1004	2370.5538	4723.0738	2362.0406	4722.0898	2361.5486	43
4	401.1779	201.0926	384.1514	192.5793	383.1674	192.0873	N	4683.0789	2342.0431	4666.0524	2333.5298	4665.0684	2333.0378	42
5	529.2729	265.1401	512.2463	256.6268	511.2623	256.1348	K	4569.0360	2285.0216	4552.0095	2276.5084	4551.0254	2276.0164	41
6	657.3315	329.1694	640.3049	320.6561	639.3209	320.1641	Q	4440.9410	2220.9742	4423.9145	2212.4609	4422.9305	2211.9689	40
7	788.3720	394.6896	771.3454	386.1763	770.3614	385.6843	M	4312.8825	2156.9449	4295.8559	2148.4316	4294.8719	2147.9396	39
8	901.4560	451.2316	884.4295	442.7184	883.4454	442.2264	L	4181.8420	2091.4246	4164.8154	2082.9114	4163.8314	2082.4193	38
9	1061.4867	531.2470	1044.4601	522.7337	1043.4761	522.2417	C	4068.7579	2034.8826	4051.7314	2026.3693	4050.7474	2025.8773	37
10	1162.5343	581.7708	1145.5078	573.2575	1144.5238	572.7655	T	3908.7273	1954.8673	3891.7007	1946.3540	3890.7167	1945.8620	36
11	1322.5650	661.7861	1305.5384	653.2729	1304.5544	652.7809	C	3807.6796	1904.3434	3790.6530	1895.8302	3789.6690	1895.3381	35
12	1435.6491	718.3282	1418.6225	709.8149	1417.6385	709.3229	L	3647.6489	1824.3281	3630.6224	1815.8148	3629.6384	1815.3228	34
13	1492.6705	746.8389	1475.6440	738.3256	1474.6600	737.8336	G	3534.5649	1767.7861	3517.5383	1759.2728	3516.5543	1758.7808	33
14	1606.7134	803.8604	1589.6869	795.3471	1588.7029	794.8551	N	3477.5434	1739.2753	3460.5169	1730.7621	3459.5328	1730.2701	32
15	1663.7349	832.3711	1646.7084	823.8578	1645.7243	823.3658	G	3363.5005	1682.2539	3346.4739	1673.7406	3345.4899	1673.2486	31
16	1762.8033	881.9053	1745.7768	873.3920	1744.7928	872.9000	V	3306.4790	1653.7431	3289.4525	1645.2299	3288.4685	1644.7379	30
17	1849.8354	925.4213	1832.8088	916.9080	1831.8248	916.4160	S	3207.4106	1604.2089	3190.3841	1595.6957	3189.4000	1595.2037	29
18	2009.8660	1005.4366	1992.8395	996.9234	1991.8554	996.4314	C	3120.3786	1560.6929	3103.3520	1552.1797	3102.3680	1551.6876	28
19	2137.9246	1069.4659	2120.8980	1060.9527	2119.9140	1060.4606	Q	2960.3479	1480.6776	2943.3214	1472.1643	2942.3374	1471.6723	27

**20**	2266.9672	1133.9872	2249.9406	1125.4739	2248.9566	1124.9819	**E**	2832.2894	1416.6483	2815.2628	1408.1350	2814.2788	1407.6430	**26**
**21**	2368.0149	1184.5111	2350.9883	1175.9978	2350.0043	1175.5058	**T**	2703.2468	1352.1270	2686.2202	1343.6137	2685.2362	1343.1217	**25**
**22**	2439.0520	1220.0296	2422.0254	1211.5163	2421.0414	1211.0243	**A**	2602.1991	1301.6032	2585.1725	1293.0899	2584.1885	1292.5979	**24**
**23**	2538.1204	1269.5638	2521.0938	1261.0506	2520.1098	1260.5585	**V**	2531.1620	1266.0846	2514.1354	1257.5713	2513.1514	1257.0793	**23**
**24**	2639.1681	1320.0877	2622.1415	1311.5744	2621.1575	1311.0824	**T**	2432.0936	1216.5504	2415.0670	1208.0371	2414.0830	1207.5451	**22**
**25**	2767.2266	1384.1170	2750.2001	1375.6037	2749.2161	1375.1117	**Q**	2331.0459	1166.0266	2314.0193	1157.5133	2313.0353	1157.0213	**21**
**26**	2868.2743	1434.6408	2851.2478	1426.1275	2850.2637	1425.6355	**T**	2202.9873	1101.9973	2185.9607	1093.4840	2184.9767	1092.9920	**20**
**27**	3031.3376	1516.1725	3014.3111	1507.6592	3013.3271	1507.1672	**Y**	2101.9396	1051.4734	2084.9131	1042.9602	2083.9291	1042.4682	**19**
**28**	3088.3591	1544.6832	3071.3326	1536.1699	3070.3485	1535.6779	**G**	1938.8763	969.9418	1921.8497	961.4285	1920.8657	960.9365	**18**
**29**	3145.3806	1573.1939	3128.3540	1564.6806	3127.3700	1564.1886	**G**	1881.8548	941.4310	1864.8283	932.9178	1863.8443	932.4258	**17**
**30**	3259.4235	1630.2154	3242.3969	1621.7021	3241.4129	1621.2101	**N**	1824.8334	912.9203	1807.8068	904.4070	1806.8228	903.9150	**16**
**31**	3346.4555	1673.7314	3329.4290	1665.2181	3328.4450	1664.7261	**S**	1710.7904	855.8989	1693.7639	847.3856	1692.7799	846.8936	**15**
**32**	3460.4985	1730.7529	3443.4719	1722.2396	3442.4879	1721.7476	**N**	1623.7584	812.3828	1606.7319	803.8696	1605.7478	803.3776	**14**
**33**	3517.5199	1759.2636	3500.4934	1750.7503	3499.5094	1750.2583	**G**	1509.7155	755.3614	1492.6889	746.8481	1491.7049	746.3561	**13**
**34**	3646.5625	1823.7849	3629.5360	1815.2716	3628.5519	1814.7796	**E**	1452.6940	726.8506	1435.6675	718.3374	1434.6834	717.8454	**12**
**35**	3743.6153	1872.3113	3726.5887	1863.7980	3725.6047	1863.3060	**P**	***1323.6514***	662.3293	1306.6249	653.8161	1305.6409	653.3241	**11**
**36**	3903.6459	1952.3266	3886.6194	1943.8133	3885.6354	1943.3213	**C**	1226.5987	613.8030	1209.5721	605.2897	1208.5881	604.7977	**10**
**37**	4002.7143	2001.8608	3985.6878	1993.3475	3984.7038	1992.8555	**V**	***1066.5680***	533.7876	1049.5415	525.2744	1048.5574	524.7824	**9**
**38**	4115.7984	2058.4028	4098.7719	2049.8896	4097.7878	2049.3976	**L**	***967.4996***	484.2534	950.4730	475.7402	949.4890	475.2482	**8**
**39**	4212.8512	2106.9292	4195.8246	2098.4159	4194.8406	2097.9239	**P**	***854.4155***	427.7114	837.3890	419.1981	836.4050	418.7061	**7**
**40**	4359.9196	2180.4634	4342.8930	2171.9502	4341.9090	2171.4581	**F**	***757.3628***	379.1850	740.3362	370.6717	739.3522	370.1797	**6**
**41**	4460.9673	2230.9873	4443.9407	2222.4740	4442.9567	2221.9820	**T**	***610.2944***	305.6508	593.2678	297.1375	592.2838	296.6455	**5**
**42**	4624.0306	2312.5189	4607.0040	2304.0057	4606.0200	2303.5136	**Y**	***509.2467***	255.1270	492.2201	246.6137			**4**
**43**	4738.0735	2369.5404	4721.0470	2361.0271	4720.0630	2360.5351	**N**	***346.1833***	173.5953	329.1568	165.0820			**3**
**44**	4795.0950	2398.0511	4778.0684	2389.5379	4777.0844	2389.0458	**G**	232.1404	116.5738	215.1139	108.0606			**2**
**45**							**R**	***175.1190***	88.0631	158.0924	79.5498			**1**

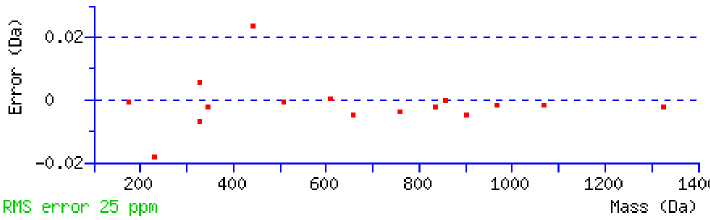

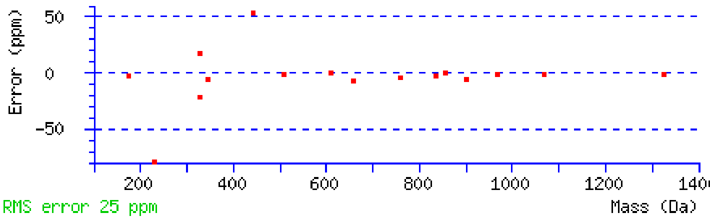

NCBI **BLAST** search of TQGNKQMLCTCLGNGVSCQETAVTQTYGGNSNGEPCVLPFTYNGR
(Parameters: blastp, nr protein database, expect=20000, no filter, PAM30)
Other BLAST web gateways

All matches to this query

Score	Mr(calc)	Delta	Sequence
50.5	4968.1994	0.0068	TQGNKQMLCTCLGNGVSCQETAVTQTYGGNSNGEPCVLPFTYNGR
1.3	4968.2159	-0.0097	AVLIDEVCTMNDGPHSALYTMMKDQYANYVVQKMIDVAEPGQR

Mascot: http://www.matrixscience.com/

Mascot Search Results

Peptide View

MS/MS Fragmentation of **QGYNGRPNVEGK**
Found in **Q4ZHG4** in **uni_human_nr**, FNDC1_HUMAN Fibronectin type III domain-containing protein 1 OS=Homo sapiens GN=FNDC1 PE=2 SV=4

Match to Query 2387: 1318.627212 from(440.549680,3+) intensity(238158.1094) rtinseconds(831) scans(2878) index(1182)
Title: 130809_HPL_Human_Plaque_BR1_TR3_05_Spectrum023747_scans__2878_RTINSECONDS=831
Data file E:\\RAW\\HumanPlaques\\mgf\\T\\1D\\T130809_HPL_Human_Plaque_BR1_TR3_05.mgf

Click mouse within plot area to zoom in by factor of two about that point
Or, Plot from 0 to 1400 Da Full range
Label all possible matches ○ Label matches used for scoring ⊙
Show Y-axis ☐

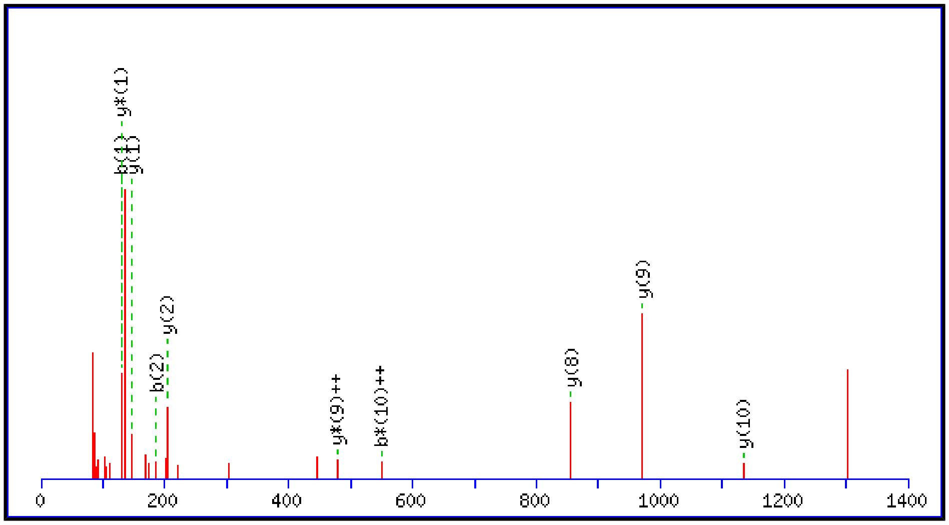

Monoisotopic mass of neutral peptide Mr(calc): 1318.6266
Fixed modifications: Carbamidomethyl (C) (apply to specified residues or termini only)
Variable modifications:
N4 : Deamidated (NQ)
Ions Score: 28 **Expect:** 0.083
Matches : 10/110 fragment ions using 19 most intense peaks (help)

#	b	b^{++}	b*	b^{*++}	b^0	b^{0++}	Seq.	y	y^{++}	y*	y^{*++}	y^0	y^{0++}	#
1	**129.0659**	65.0366	112.0393	56.5233			Q							12
2	**186.0873**	93.5473	169.0608	85.0340			G	1191.5753	596.2913	1174.5487	587.7780	1173.5647	587.2860	11
3	349.1506	175.0790	332.1241	166.5657			Y	***1134.5538***	567.7805	1117.5273	559.2673	1116.5432	558.7753	10
4	464.1776	232.5924	447.1510	224.0792			N	***971.4905***	486.2489	954.4639	477.7356	953.4799	477.2436	9
5	521.1991	261.1032	504.1725	252.5899			G	***856.4635***	428.7354	839.4370	420.2221	838.4530	419.7301	8
6	677.3002	339.1537	660.2736	330.6404			R	799.4421	400.2247	782.4155	391.7114	781.4315	391.2194	7
7	774.3529	387.6801	757.3264	379.1668			P	643.3410	322.1741	626.3144	313.6608	625.3304	313.1688	6
8	888.3959	444.7016	871.3693	436.1883			N	546.2882	273.6477	529.2617	265.1345	528.2776	264.6425	5
9	987.4643	494.2358	970.4377	485.7225			V	432.2453	216.6263	415.2187	208.1130	414.2347	207.6210	4
10	1116.5069	558.7571	1099.4803	550.2438	1098.4963	549.7518	E	333.1769	167.0921	316.1503	158.5788	315.1663	158.0868	3
11	1173.5283	587.2678	1156.5018	578.7545	1155.5178	578.2625	G	***204.1343***	102.5708	187.1077	94.0575			2
12							K	***147.1128***	74.0600	130.0863	65.5468			1

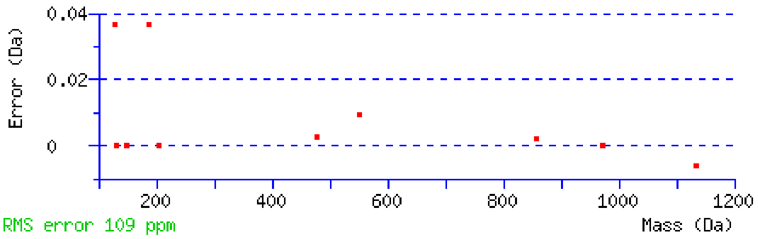

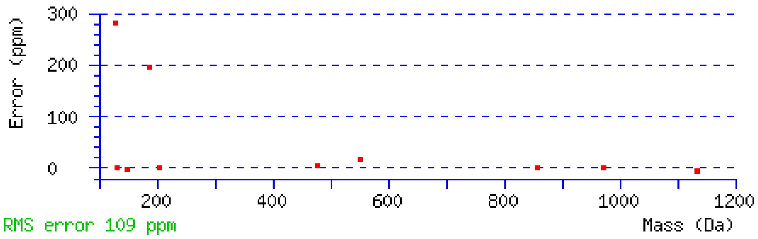

NCBI **BLAST** search of QGYNGRPNVEGK
(Parameters: blastp, nr protein database, expect=20000, no filter, PAM30)
Other BLAST web gateways

All matches to this query

Score	Mr(calc)	Delta	Sequence
28.4	1318.6266	0.0007	QGYNGRPNVEGK
19.5	1318.6266	0.0007	QGYNGRPNVEGK
8.2	1318.6228	0.0045	QTFSFLSGMTGK
5.2	1318.6266	0.0007	QGYNGRPNVEGK
4.8	1317.6234	1.0038	SQPSAAQPSKMGK
3.0	1318.6265	0.0007	QAQARSWASQGK
3.0	1318.6265	0.0007	QAQARSWASQGK
0.4	1318.6225	0.0047	RNRSDTEQEGK

Mascot: http://www.matrixscience.com/

Mascot Search Results

Peptide View

MS/MS Fragmentation of **RNANGRSPNNSDK**
Found in **H7C550** in **uni_human_nr**, H7C550_HUMAN FERM domain-containing protein 4B (Fragment) OS=Homo sapiens GN=FRMD4B PE=4 SV=1

Match to Query 3146: 1433.616188 from(717.815370,2+) intensity(269886.9375) rtinseconds(1563) scans(7830) index(5676)
Title: 130806_HPL_Human_Plaque_BR1_TR2_20_Spectrum025544_scans__7830_RTINSECONDS=1563
Data file E:\\RAW\\HumanPlaques\\mgf\\T\\1D\\T130806_HPL_Human_Plaque_BR1_TR2_20.mgf

Click mouse within plot area to zoom in by factor of two about that point
Or, Plot from 50 to 700 Da Full range
Label all possible matches ○ Label matches used for scoring ⊙
Show Y-axis ☐

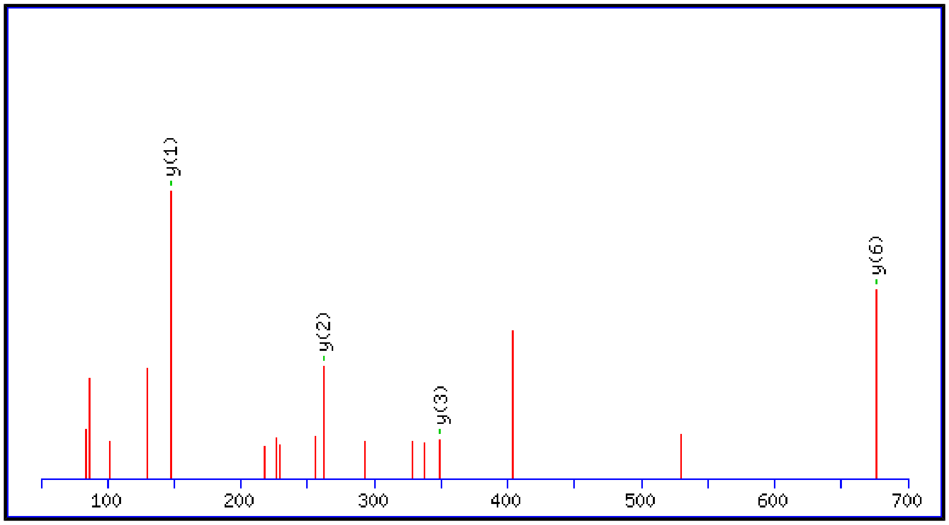

Monoisotopic mass of neutral peptide Mr(calc): 1432.6178
Fixed modifications: Carbamidomethyl (C) (apply to specified residues or termini only)
Variable modifications:
N2 : Deamidated (NQ)
N4 : Deamidated (NQ)
N9 : Deamidated (NQ)
N10 : Deamidated (NQ)
Ions Score: 27 **Expect:** 0.043
Matches : 4/130 fragment ions using 6 most intense peaks (help)

#	b	b^{++}	b*	b^{*++}	b^{0}	b^{0++}	Seq.	y	y^{++}	y*	y^{*++}	y^{0}	y^{0++}	#
1	157.1084	79.0578	140.0818	70.5446			R							13
2	272.1353	136.5713	255.1088	128.0580			N	1277.5240	639.2657	1260.4975	630.7524	1259.5135	630.2604	12
3	343.1724	172.0899	326.1459	163.5766			A	1162.4971	581.7522	1145.4705	573.2389	1144.4865	572.7469	11
4	458.1994	229.6033	441.1728	221.0901			N	1091.4600	546.2336	1074.4334	537.7203	1073.4494	537.2283	10
5	515.2209	258.1141	498.1943	249.6008			G	976.4330	488.7202	959.4065	480.2069	958.4225	479.7149	9
6	671.3220	336.1646	654.2954	327.6513			R	919.4116	460.2094	902.3850	451.6961	901.4010	451.2041	8
7	758.3540	379.6806	741.3274	371.1674	740.3434	370.6754	S	763.3105	382.1589	746.2839	373.6456	745.2999	373.1536	7
8	855.4068	428.2070	838.3802	419.6937	837.3962	419.2017	P	***676.2784***	338.6429	659.2519	330.1296	658.2679	329.6376	6
9	970.4337	485.7205	953.4071	477.2072	952.4231	476.7152	N	579.2257	290.1165	562.1991	281.6032	561.2151	281.1112	5
10	1085.4606	543.2340	1068.4341	534.7207	1067.4501	534.2287	N	464.1987	232.6030	447.1722	224.0897	446.1882	223.5977	4
11	1172.4927	586.7500	1155.4661	578.2367	1154.4821	577.7447	S	***349.1718***	175.0895	332.1452	166.5763	331.1612	166.0842	3
12	1287.5196	644.2634	1270.4931	635.7502	1269.5090	635.2582	D	***262.1397***	131.5735	245.1132	123.0602	244.1292	122.5682	2
13							K	***147.1128***	74.0600	130.0863	65.5468			1

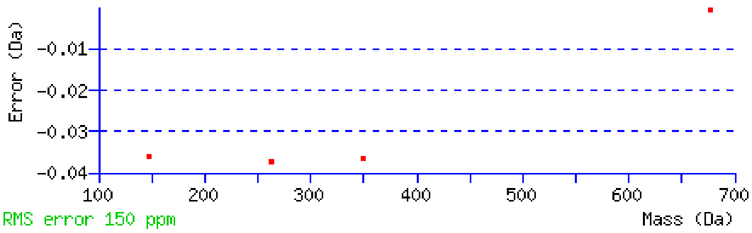

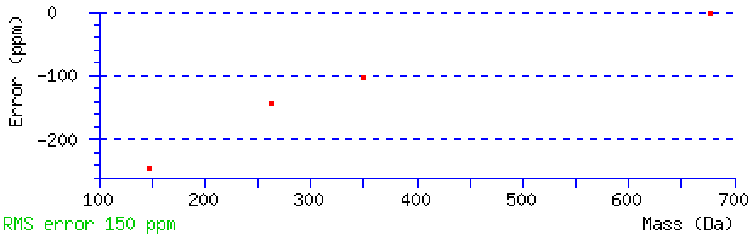

NCBI **BLAST** search of RNANGRSPNNSDK
(Parameters: blastp, nr protein database, expect=20000, no filter, PAM30)
Other BLAST web gateways

All matches to this query

Score	Mr(calc)	Delta	Sequence
26.7	1432.6178	0.9984	RNANGRSPNNSDK
18.3	1431.6154	2.0008	DTQWPENNLADK
15.2	1431.6089	2.0073	EWMTQQTEHNK
14.7	1432.6140	1.0022	SHMEEKDGLEDK
5.1	1431.6089	2.0073	EWMTQQTEHNK
4.7	1431.6048	2.0114	QREKMNDDHNK
4.7	1431.6048	2.0114	QREKMNDDHNK
3.4	1433.6207	-0.0045	FDYLMQLMNDK
3.4	1433.6207	-0.0045	FDYLMQLMNDK
3.4	1433.6207	-0.0045	YDYLMQLMNDK

Mascot: http://www.matrixscience.com/

Mascot Search Results

Peptide View

MS/MS Fragmentation of **SRYDVFCFTSNFNGR**
Found in **P10915** in **uni_human_nr**, HPLN1_HUMAN Hyaluronan and proteoglycan link protein 1 OS=Homo sapiens GN=HAPLN1 PE=2 SV=2

Match to Query 6444: 1870.794582 from(624.605470,3+) intensity(1459466.8750) rtinseconds(2632) scans(15301) index(11764)
Title: 130806_HPL_Human_Plaque_BR2_TR2_09_Spectrum032986_scans__15301_RTINSECONDS=2632
Data file E:\\RAW\\HumanPlaques\\mgf\\T\\1D\\T130806_HPL_Human_Plaque_BR2_TR2_09.mgf

Click mouse within plot area to zoom in by factor of two about that point
Or, [Plot from] [0] to [1900] Da [Full range]
Label all possible matches ○ Label matches used for scoring ⊙
Show Y-axis ☐

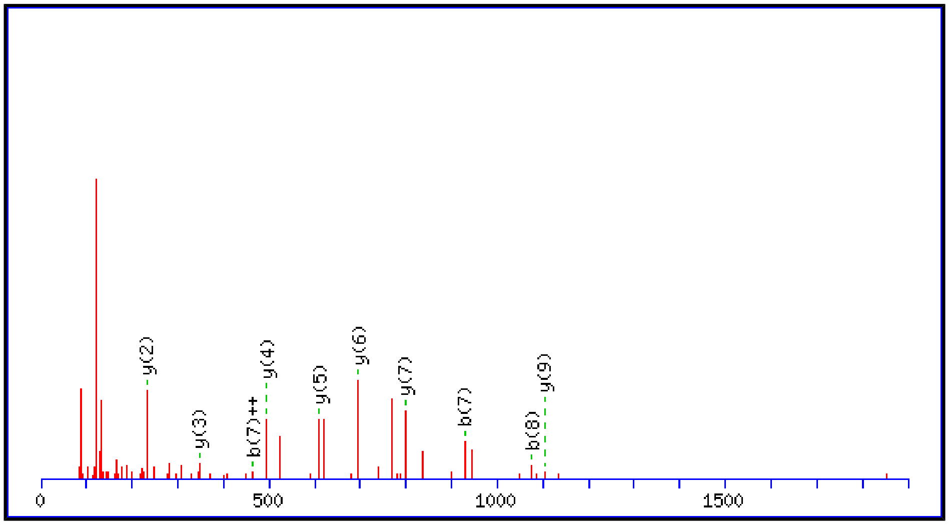


```
Monoisotopic mass of neutral peptide Mr(calc): 1870.7944
Fixed modifications: Carbamidomethyl (C) (apply to specified residues or termini only)
Variable modifications:
N11    : Deamidated (NQ)
N13    : Deamidated (NQ)
Ions Score: 60  Expect: 1.8e-005
Matches : 10/156 fragment ions using 12 most intense peaks   (help)
```

#	b	b^{++}	b*	b^{*++}	b^0	b^{0++}	Seq.	y	y^{++}	y*	y^{*++}	y^0	y^{0++}	#
1	88.0393	44.5233			70.0287	35.5180	S							15
2	244.1404	122.5738	227.1139	114.0606	226.1298	113.5686	R	1784.7697	892.8885	1767.7431	884.3752	1766.7591	883.8832	14
3	407.2037	204.1055	390.1772	195.5922	389.1932	195.1002	Y	1628.6686	814.8379	1611.6420	806.3247	1610.6580	805.8326	13
4	522.2307	261.6190	505.2041	253.1057	504.2201	252.6137	D	1465.6053	733.3063	1448.5787	724.7930	1447.5947	724.3010	12
5	621.2991	311.1532	604.2726	302.6399	603.2885	302.1479	V	1350.5783	675.7928	1333.5518	667.2795	1332.5677	666.7875	11
6	768.3675	384.6874	751.3410	376.1741	750.3569	375.6821	F	1251.5099	626.2586	1234.4834	617.7453	1233.4993	617.2533	10
7	**928.3982**	**464.7027**	911.3716	456.1894	910.3876	455.6974	C	***1104.4415***	552.7244	1087.4149	544.2111	1086.4309	543.7191	9
8	**1075.4666**	538.2369	1058.4400	529.7237	1057.4560	529.2316	F	944.4108	472.7091	927.3843	464.1958	926.4003	463.7038	8
9	1176.5143	588.7608	1159.4877	580.2475	1158.5037	579.7555	T	***797.3424***	399.1748	780.3159	390.6616	779.3319	390.1696	7
10	1263.5463	632.2768	1246.5197	623.7635	1245.5357	623.2715	S	***696.2947***	348.6510	679.2682	340.1377	678.2842	339.6457	6
11	1378.5732	689.7903	1361.5467	681.2770	1360.5627	680.7850	N	***609.2627***	305.1350	592.2362	296.6217			5
12	1525.6416	763.3245	1508.6151	754.8112	1507.6311	754.3192	F	***494.2358***	247.6215	477.2092	239.1083			4
13	1640.6686	820.8379	1623.6420	812.3247	1622.6580	811.8326	N	***347.1674***	174.0873	330.1408	165.5740			3
14	1697.6900	849.3487	1680.6635	840.8354	1679.6795	840.3434	G	***232.1404***	116.5738	215.1139	108.0606			2

**15**							**R**	175.1190	88.0631	158.0924	79.5498			**1**

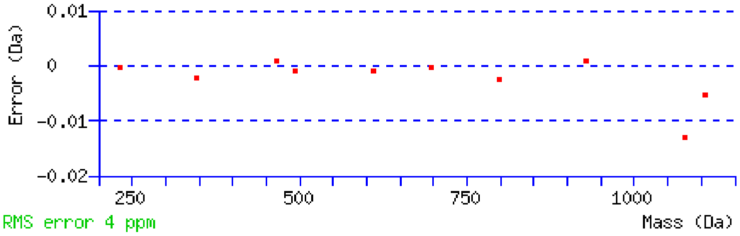

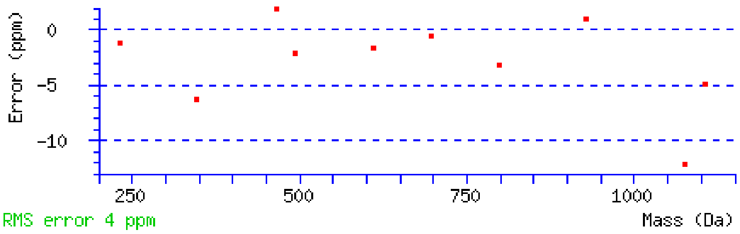

NCBI **BLAST** search of SRYDVFCFTSNFNGR
(Parameters: blastp, nr protein database, expect=20000, no filter, PAM30)
Other BLAST web gateways

All matches to this query

Score	Mr(calc)	Delta	Sequence
60.1	1870.7944	0.0001	SRYDVFCFTSNFNGR

**Mascot:** http://www.matrixscience.com/

Mascot Search Results

Peptide View

MS/MS Fragmentation of **SRYDVFCFTSNFNGR**
Found in **P10915** in **uni_human_nr**, HPLN1_HUMAN Hyaluronan and proteoglycan link protein 1 OS=Homo sapiens GN=HAPLN1 PE=2 SV=2

Match to Query 7282: 1869.811662 from(624.277830,3+) intensity(5708795.0000) rtinseconds(2501) scans(15486) index(12280)
Title: 130801_HPL_Human_Plaque_BR1_TR1_04_Spectrum035278_scans__15486_RTINSECONDS=2501
Data file E:\\RAW\\HumanPlaques\\mgf\\T\\1D\\T130801_HPL_Human_Plaque_BR1_TR1_04.mgf

Click mouse within plot area to zoom in by factor of two about that point
Or, Plot from 0 to 1900 Da Full range
Label all possible matches ○ Label matches used for scoring ⊙
Show Y-axis ☐

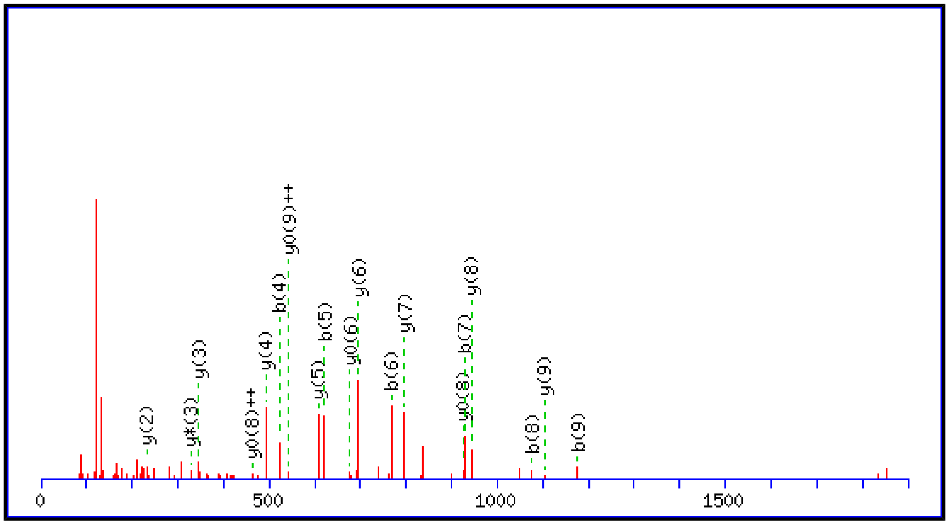


```
Monoisotopic mass of neutral peptide Mr(calc): 1869.8104
Fixed modifications: Carbamidomethyl (C) (apply to specified residues or termini only)
Variable modifications:
N11    : Deamidated (NQ)
Ions Score: 51  Expect: 0.00019
Matches : 19/156 fragment ions using 33 most intense peaks   (help)
```

#	b	b^{++}	b*	b^{*++}	b^0	b^{0++}	Seq.	y	y^{++}	y*	y^{*++}	y^0	y^{0++}	#
1	88.0393	44.5233			70.0287	35.5180	S							15
2	244.1404	122.5738	227.1139	114.0606	226.1298	113.5686	R	1783.7857	892.3965	1766.7591	883.8832	1765.7751	883.3912	14
3	407.2037	204.1055	390.1772	195.5922	389.1932	195.1002	Y	1627.6846	814.3459	1610.6580	805.8326	1609.6740	805.3406	13
4	***522.2307***	261.6190	505.2041	253.1057	504.2201	252.6137	D	1464.6212	732.8143	1447.5947	724.3010	1446.6107	723.8090	12
5	***621.2991***	311.1532	604.2726	302.6399	603.2885	302.1479	V	1349.5943	675.3008	1332.5677	666.7875	1331.5837	666.2955	11
6	***768.3675***	384.6874	751.3410	376.1741	750.3569	375.6821	F	1250.5259	625.7666	1233.4993	617.2533	1232.5153	616.7613	10
7	***928.3982***	464.7027	911.3716	456.1894	910.3876	455.6974	C	***1103.4575***	552.2324	1086.4309	543.7191	1085.4469	543.2271	9
8	***1075.4666***	538.2369	1058.4400	529.7237	1057.4560	529.2316	F	***943.4268***	472.2170	926.4003	463.7038	925.4163	463.2118	8
9	***1176.5143***	588.7608	1159.4877	580.2475	1158.5037	579.7555	T	***796.3584***	398.6828	779.3319	390.1696	778.3478	389.6776	7
10	1263.5463	632.2768	1246.5197	623.7635	1245.5357	623.2715	S	***695.3107***	348.1590	678.2842	339.6457	677.3002	339.1537	6
11	1378.5732	689.7903	1361.5467	681.2770	1360.5627	680.7850	N	***608.2787***	304.6430	591.2522	296.1297			5
12	1525.6416	763.3245	1508.6151	754.8112	1507.6311	754.3192	F	***493.2518***	247.1295	476.2252	238.6162			4
13	1639.6846	820.3459	1622.6580	811.8326	1621.6740	811.3406	N	***346.1833***	173.5953	329.1568	165.0820			3
14	1696.7060	848.8567	1679.6795	840.3434	1678.6955	839.8514	G	***232.1404***	116.5738	215.1139	108.0606			2
15							R	175.1190	88.0631	158.0924	79.5498			1

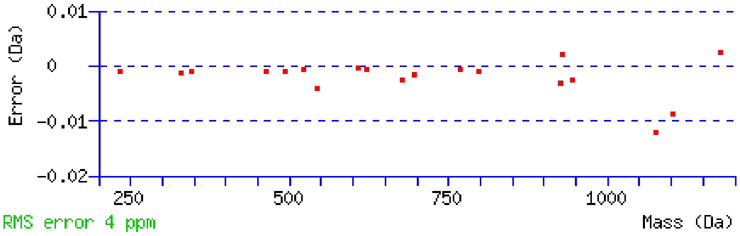

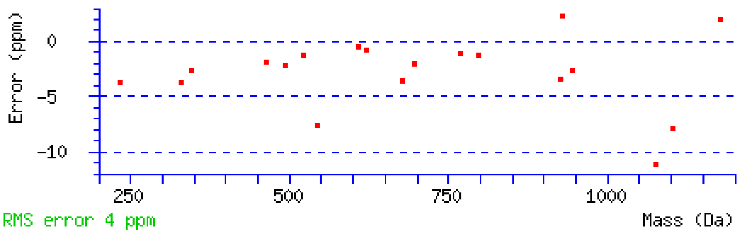

NCBI **BLAST** search of SRYDVFCFTSNFNGR
(Parameters: blastp, nr protein database, expect=20000, no filter, PAM30)
Other BLAST web gateways

All matches to this query

Score	Mr(calc)	Delta	Sequence	Site Analysis
51.5	1869.8104	0.0012	SRYDVFCFTSNFNGR	Deamidated N11 98.55%
33.2	1869.8104	0.0012	SRYDVFCFTSNFNGR	Deamidated N13 1.45%

Mascot: http://www.matrixscience.com/

Mascot Search Results

Peptide View

MS/MS Fragmentation of **SRYDVFCFTSNFNGR**
Found in **P10915** in **uni_human_nr**, HPLN1_HUMAN Hyaluronan and proteoglycan link protein 1 OS=Homo sapiens GN=HAPLN1 PE=2 SV=2

Match to Query 8021: 1869.811988 from(935.913270,2+) intensity(2719202.2500) rtinseconds(2479) scans(15953) index(12909)
Title: 130806_HPL_Human_Plaque_BR1_TR2_03_Spectrum036848_scans__15953_RTINSECONDS=2479
Data file E:\\RAW\\HumanPlaques\\mgf\\T\\1D\\T130806_HPL_Human_Plaque_BR1_TR2_03.mgf

Click mouse within plot area to zoom in by factor of two about that point
Or, Plot from 0 to 1900 Da Full range
Label all possible matches ○ Label matches used for scoring ⊙
Show Y-axis ☐

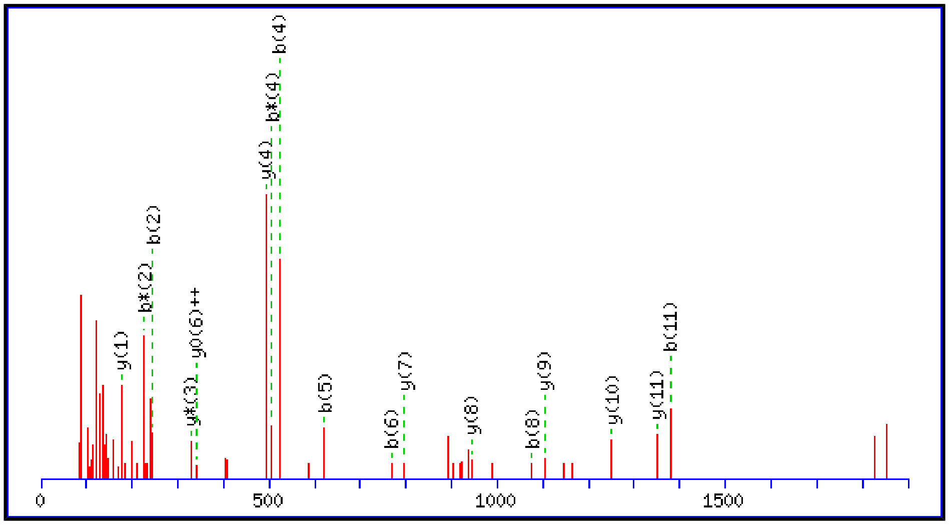


```
Monoisotopic mass of neutral peptide Mr(calc): 1869.8104
Fixed modifications: Carbamidomethyl (C) (apply to specified residues or termini only)
Variable modifications:
N11     : Deamidated (NQ)
Ions Score: 37  Expect: 0.0059
Matches : 17/156 fragment ions using 33 most intense peaks   (help)
```

#	b	b^{++}	b*	b^{*++}	b^0	b^{0++}	Seq.	y	y^{++}	y*	y^{*++}	y^0	y^{0++}	#
1	88.0393	44.5233			70.0287	35.5180	S							15
2	***244.1404***	122.5738	227.1139	114.0606	226.1298	113.5686	R	1783.7857	892.3965	1766.7591	883.8832	1765.7751	883.3912	14
3	407.2037	204.1055	390.1772	195.5922	389.1932	195.1002	Y	1627.6846	814.3459	1610.6580	805.8326	1609.6740	805.3406	13
4	***522.2307***	261.6190	505.2041	253.1057	504.2201	252.6137	D	1464.6212	732.8143	1447.5947	724.3010	1446.6107	723.8090	12
5	***621.2991***	311.1532	604.2726	302.6399	603.2885	302.1479	V	***1349.5943***	675.3008	1332.5677	666.7875	1331.5837	666.2955	11
6	***768.3675***	384.6874	751.3410	376.1741	750.3569	375.6821	F	***1250.5259***	625.7666	1233.4993	617.2533	1232.5153	616.7613	10
7	928.3982	464.7027	911.3716	456.1894	910.3876	455.6974	C	***1103.4575***	552.2324	1086.4309	543.7191	1085.4469	543.2271	9
8	***1075.4666***	538.2369	1058.4400	529.7237	1057.4560	529.2316	F	***943.4268***	472.2170	926.4003	463.7038	925.4163	463.2118	8
9	1176.5143	588.7608	1159.4877	580.2475	1158.5037	579.7555	T	***796.3584***	398.6828	779.3319	390.1696	778.3478	389.6776	7
10	1263.5463	632.2768	1246.5197	623.7635	1245.5357	623.2715	S	695.3107	348.1590	678.2842	339.6457	677.3002	339.1537	6
11	***1378.5732***	689.7903	1361.5467	681.2770	1360.5627	680.7850	N	608.2787	304.6430	591.2522	296.1297			5
12	1525.6416	763.3245	1508.6151	754.8112	1507.6311	754.3192	F	***493.2518***	247.1295	476.2252	238.6162			4
13	1639.6846	820.3459	1622.6580	811.8326	1621.6740	811.3406	N	346.1833	173.5953	329.1568	165.0820			3
14	1696.7060	848.8567	1679.6795	840.3434	1678.6955	839.8514	G	232.1404	116.5738	215.1139	108.0606			2
15							R	***175.1190***	88.0631	158.0924	79.5498			1

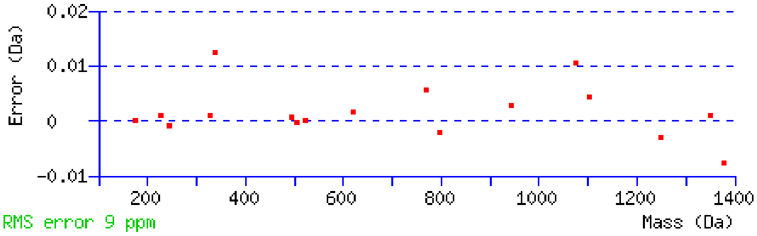

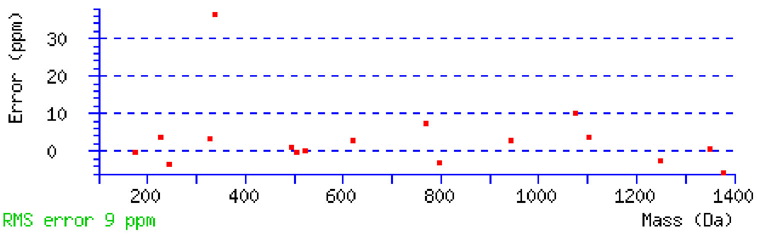

NCBI **BLAST** search of SRYDVFCFTSNFNGR
(Parameters: blastp, nr protein database, expect=20000, no filter, PAM30)
Other BLAST web gateways

All matches to this query

Score	Mr(calc)	Delta	Sequence	Site Analysis
36.8	1869.8104	0.0015	SRYDVFCFTSNFNGR	Deamidated N11 95.07%
23.9	1869.8104	0.0015	SRYDVFCFTSNFNGR	Deamidated N13 4.93%

Mascot: http://www.matrixscience.com/

Mascot Search Results

Peptide View

MS/MS Fragmentation of **SRYDVFCFTSNFNGR**
Found in **P10915** in **uni_human_nr**, HPLN1_HUMAN Hyaluronan and proteoglycan link protein 1 OS=Homo sapiens GN=HAPLN1 PE=2 SV=2

Match to Query 8867: 1868.827122 from(623.949650,3+) intensity(3956803.0000) rtinseconds(2301) scans(14978) index(11978)
Title: 130809_HPL_Human_Plaque_BR1_TR3_02_Spectrum036504_scans__14978_RTINSECONDS=2301
Data file E:\\RAW\\HumanPlaques\\mgf\\T\\1D\\T130809_HPL_Human_Plaque_BR1_TR3_02.mgf

Click mouse within plot area to zoom in by factor of two about that point
Or, [Plot from] [0] to [1900] Da [Full range]
Label all possible matches ○ Label matches used for scoring ⦿
Show Y-axis ☐

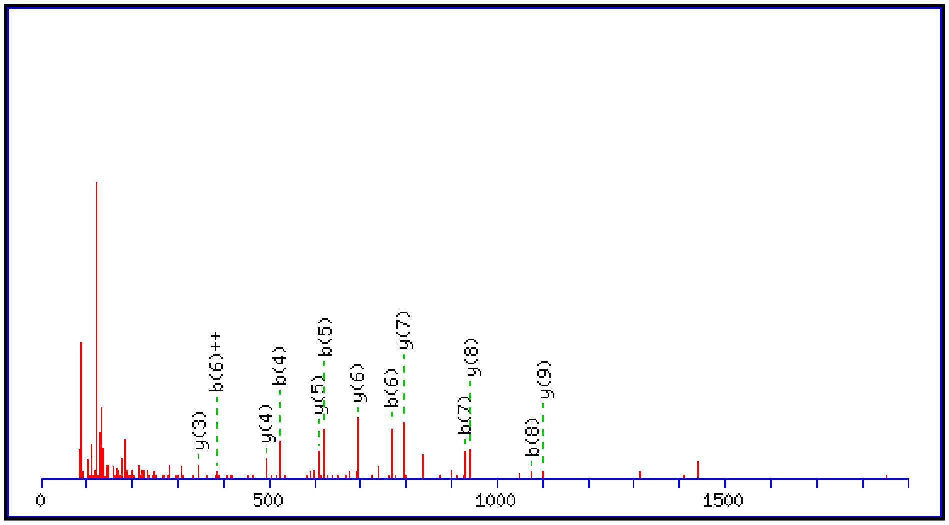

Monoisotopic mass of neutral peptide Mr(calc): 1868.8264
Fixed modifications: Carbamidomethyl (C) (apply to specified residues or termini only)
Ions Score: 39 **Expect:** 0.0053
Matches : 13/156 fragment ions using 25 most intense peaks (help)

#	b	b^{++}	b*	b^{*++}	b^{0}	b^{0++}	Seq.	y	y^{++}	y*	y^{*++}	y^{0}	y^{0++}	#
1	88.0393	44.5233			70.0287	35.5180	S							15
2	244.1404	122.5738	227.1139	114.0606	226.1298	113.5686	R	1782.8017	891.9045	1765.7751	883.3912	1764.7911	882.8992	14
3	407.2037	204.1055	390.1772	195.5922	389.1932	195.1002	Y	1626.7006	813.8539	1609.6740	805.3406	1608.6900	804.8486	13
4	***522.2307***	261.6190	505.2041	253.1057	504.2201	252.6137	D	1463.6372	732.3223	1446.6107	723.8090	1445.6267	723.3170	12
5	***621.2991***	311.1532	604.2726	302.6399	603.2885	302.1479	V	1348.6103	674.8088	1331.5837	666.2955	1330.5997	665.8035	11
6	***768.3675***	**384.6874**	751.3410	376.1741	750.3569	375.6821	F	1249.5419	625.2746	1232.5153	616.7613	1231.5313	616.2693	10
7	***928.3982***	464.7027	911.3716	456.1894	910.3876	455.6974	C	***1102.4735***	551.7404	1085.4469	543.2271	1084.4629	542.7351	9
8	***1075.4666***	538.2369	1058.4400	529.7237	1057.4560	529.2316	F	***942.4428***	471.7250	925.4163	463.2118	924.4322	462.7198	8
9	1176.5143	588.7608	1159.4877	580.2475	1158.5037	579.7555	T	***795.3744***	398.1908	778.3478	389.6776	777.3638	389.1856	7
10	1263.5463	632.2768	1246.5197	623.7635	1245.5357	623.2715	S	***694.3267***	347.6670	677.3002	339.1537	676.3161	338.6617	6
11	1377.5892	689.2982	1360.5627	680.7850	1359.5786	680.2930	N	***607.2947***	304.1510	590.2681	295.6377			5
12	1524.6576	762.8325	1507.6311	754.3192	1506.6471	753.8272	F	***493.2518***	247.1295	476.2252	238.6162			4
13	1638.7006	819.8539	1621.6740	811.3406	1620.6900	810.8486	N	***346.1833***	173.5953	329.1568	165.0820			3
14	1695.7220	848.3646	1678.6955	839.8514	1677.7115	839.3594	G	232.1404	116.5738	215.1139	108.0606			2
15							R	175.1190	88.0631	158.0924	79.5498			1

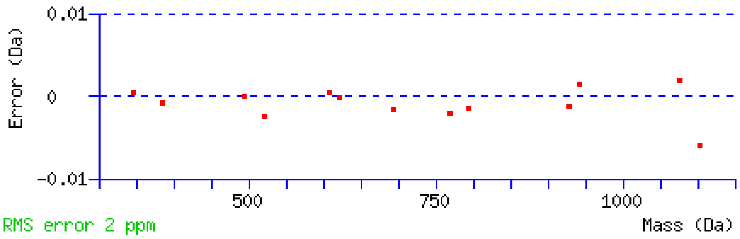

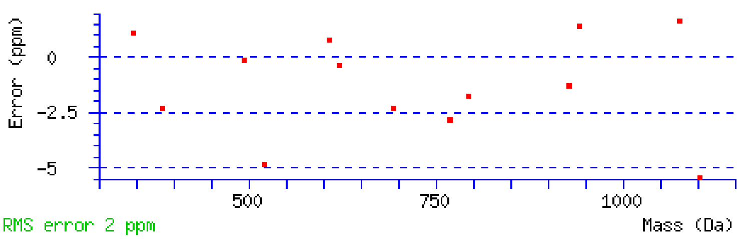

NCBI **BLAST** search of SRYDVFCFTSNFNGR
(Parameters: blastp, nr protein database, expect=20000, no filter, PAM30)
Other BLAST web gateways

All matches to this query

Score	Mr(calc)	Delta	Sequence
38.9	1868.8264	0.0007	SRYDVFCFTSNFNGR

Mascot: http://www.matrixscience.com/

Mascot Search Results

Peptide View

MS/MS Fragmentation of **YDVFCFTSNFNGR**
Found in **P10915** in **uni_human_nr**, HPLN1_HUMAN Hyaluronan and proteoglycan link protein 1 OS=Homo sapiens GN=HAPLN1 PE=2 SV=2

Match to Query 4782: 1627.661588 from(814.838070,2+) intensity(362244.4375) rtinseconds(3026) scans(17353) index(13432)
Title: 130801_HPL_Human_Plaque_BR1_TR1_18_Spectrum033083_scans__17353_RTINSECONDS=3026
Data file E:\\RAW\\HumanPlaques\\mgf\\T\\1D\\T130801_HPL_Human_Plaque_BR1_TR1_18.mgf

Click mouse within plot area to zoom in by factor of two about that point
Or, [Plot from] [0] to [1300] Da [Full range]
Label all possible matches ○ Label matches used for scoring ⊙
Show Y-axis ☐

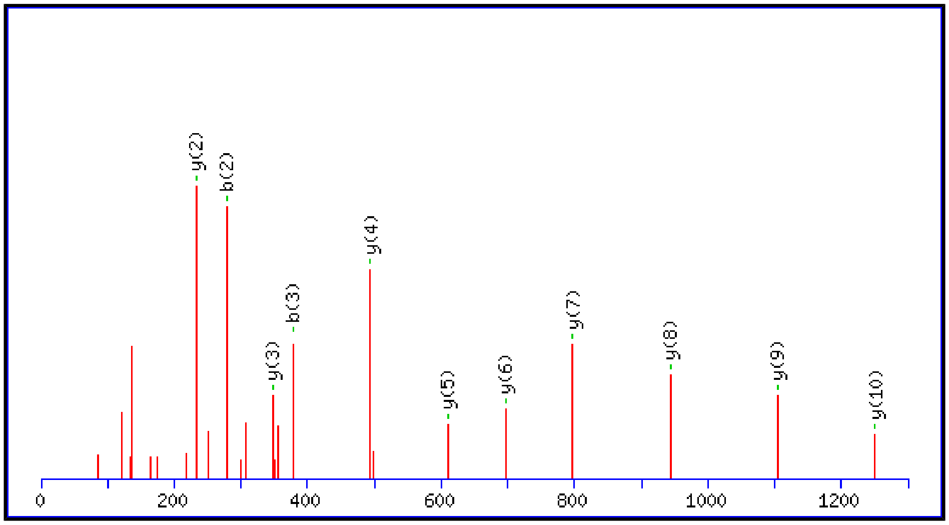

Monoisotopic mass of neutral peptide Mr(calc): 1627.6613
Fixed modifications: Carbamidomethyl (C) (apply to specified residues or termini only)
Variable modifications:
N9 : Deamidated (NQ)
N11 : Deamidated (NQ)
Ions Score: 82 **Expect:** 5.4e-008
Matches : 11/116 fragment ions using 14 most intense peaks (help)

#	b	b^{++}	b*	b^{*++}	b^0	b^{0++}	Seq.	y	y^{++}	y*	y^{*++}	y^0	y^{0++}	#
1	164.0706	82.5389					Y							13
2	**279.0975**	140.0524			261.0870	131.0471	D	1465.6053	733.3063	1448.5787	724.7930	1447.5947	724.3010	12
3	**378.1660**	189.5866			360.1554	180.5813	V	1350.5783	675.7928	1333.5518	667.2795	1332.5677	666.7875	11
4	525.2344	263.1208			507.2238	254.1155	F	***1251.5099***	626.2586	1234.4834	617.7453	1233.4993	617.2533	10
5	685.2650	343.1362			667.2545	334.1309	C	***1104.4415***	552.7244	1087.4149	544.2111	1086.4309	543.7191	9
6	832.3334	416.6704			814.3229	407.6651	F	***944.4108***	472.7091	927.3843	464.1958	926.4003	463.7038	8
7	933.3811	467.1942			915.3706	458.1889	T	***797.3424***	399.1748	780.3159	390.6616	779.3319	390.1696	7
8	1020.4131	510.7102			1002.4026	501.7049	S	***696.2947***	348.6510	679.2682	340.1377	678.2842	339.6457	6
9	1135.4401	568.2237	1118.4135	559.7104	1117.4295	559.2184	N	***609.2627***	305.1350	592.2362	296.6217			5
10	1282.5085	641.7579	1265.4820	633.2446	1264.4979	632.7526	F	***494.2358***	247.6215	477.2092	239.1083			4
11	1397.5354	699.2714	1380.5089	690.7581	1379.5249	690.2661	N	***347.1674***	174.0873	330.1408	165.5740			3
12	1454.5569	727.7821	1437.5304	719.2688	1436.5463	718.7768	G	***232.1404***	116.5738	215.1139	108.0606			2
13							R	175.1190	88.0631	158.0924	79.5498			1

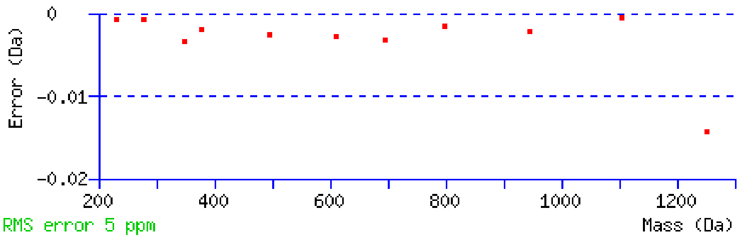

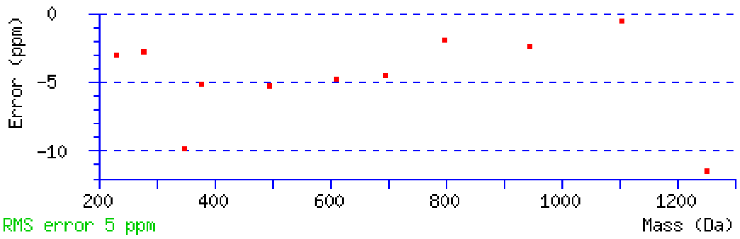

NCBI **BLAST** search of YDVFCFTSNFNGR
(Parameters: blastp, nr protein database, expect=20000, no filter, PAM30)
Other BLAST web gateways

All matches to this query

Score	Mr(calc)	Delta	Sequence
82.0	1627.6613	0.0003	YDVFCFTSNFNGR

Mascot: http://www.matrixscience.com/

Mascot Search Results

Peptide View

MS/MS Fragmentation of **YDVFCFTSNFNGR**
Found in **P10915** in **uni_human_nr**, HPLN1_HUMAN Hyaluronan and proteoglycan link protein 1 OS=Homo sapiens GN=HAPLN1 PE=2 SV=2

Match to Query 4919: 1626.675508 from(814.345030,2+) intensity(1667035.3750) rtinseconds(2880) scans(17543) index(13927)
Title: 130801_HPL_Human_Plaque_BR2_TR1_05_Spectrum035992_scans__17543_RTINSECONDS=2880
Data file E:\\RAW\\HumanPlaques\\mgf\\T\\1D\\T130801_HPL_Human_Plaque_BR2_TR1_05.mgf

Click mouse within plot area to zoom in by factor of two about that point
Or, Plot from 0 to 1400 Da Full range
Label all possible matches ○ Label matches used for scoring ⊙
Show Y-axis ☐

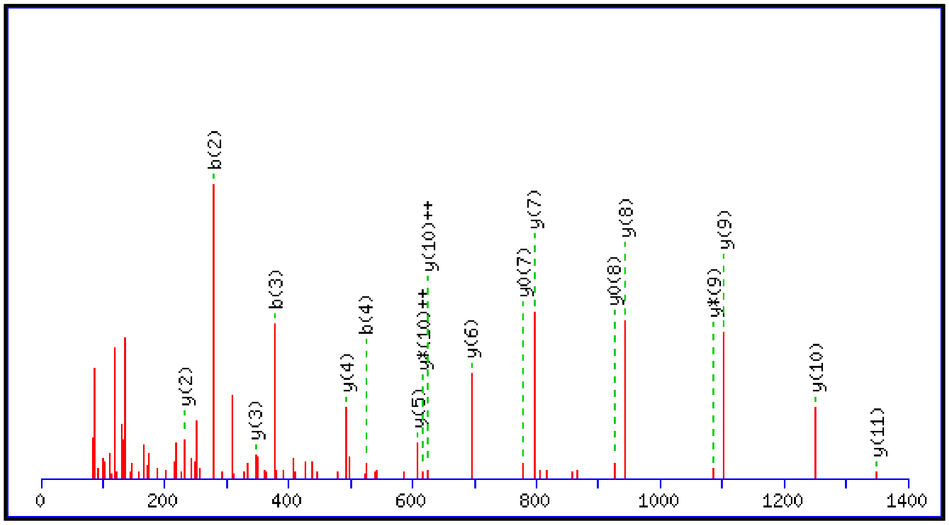

Monoisotopic mass of neutral peptide Mr(calc): 1626.6773
Fixed modifications: Carbamidomethyl (C) (apply to specified residues or termini only)
Variable modifications:
N9 : Deamidated (NQ)
Ions Score: 75 **Expect:** 4.7e-007
Matches : 18/116 fragment ions using 30 most intense peaks (help)

#	b	b^{++}	b*	b^{*++}	b^{0}	b^{0++}	Seq.	y	y^{++}	y*	y^{*++}	y^{0}	y^{0++}	#
1	164.0706	82.5389					Y							13
2	**279.0975**	140.0524			261.0870	131.0471	D	1464.6212	732.8143	1447.5947	724.3010	1446.6107	723.8090	12
3	**378.1660**	189.5866			360.1554	180.5813	V	***1349.5943***	675.3008	1332.5677	666.7875	1331.5837	666.2955	11
4	**525.2344**	263.1208			507.2238	254.1155	F	***1250.5259***	625.7666	1233.4993	617.2533	1232.5153	616.7613	10
5	685.2650	343.1362			667.2545	334.1309	C	***1103.4575***	552.2324	1086.4309	543.7191	1085.4469	543.2271	9
6	832.3334	416.6704			814.3229	407.6651	F	***943.4268***	472.2170	926.4003	463.7038	925.4163	463.2118	8
7	933.3811	467.1942			915.3706	458.1889	T	***796.3584***	398.6828	779.3319	390.1696	778.3478	389.6776	7
8	1020.4131	510.7102			1002.4026	501.7049	S	***695.3107***	348.1590	678.2842	339.6457	677.3002	339.1537	6
9	1135.4401	568.2237	1118.4135	559.7104	1117.4295	559.2184	N	***608.2787***	304.6430	591.2522	296.1297			5
10	1282.5085	641.7579	1265.4820	633.2446	1264.4979	632.7526	F	***493.2518***	247.1295	476.2252	238.6162			4
11	1396.5514	698.7794	1379.5249	690.2661	1378.5409	689.7741	N	***346.1833***	173.5953	329.1568	165.0820			3
12	1453.5729	727.2901	1436.5463	718.7768	1435.5623	718.2848	G	***232.1404***	116.5738	215.1139	108.0606			2
13							R	175.1190	88.0631	158.0924	79.5498			1

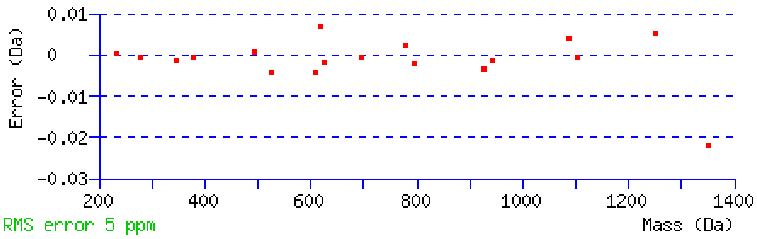

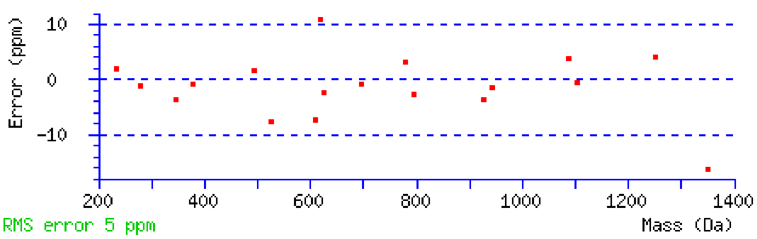

NCBI **BLAST** search of YDVFCFTSNFNGR
(Parameters: blastp, nr protein database, expect=20000, no filter, PAM30)
Other BLAST web gateways

All matches to this query

Score	Mr(calc)	Delta	Sequence	Site Analysis
74.5	1626.6773	-0.0018	YDVFCFTSNFNGR	Deamidated N9 98.79%
55.4	1626.6773	-0.0018	YDVFCFTSNFNGR	Deamidated N11 1.21%

**Mascot:** http://www.matrixscience.com/

Mascot Search Results

Peptide View

MS/MS Fragmentation of **YDVFCFTSNFNGRFYYLIHPTK**
Found in **P10915** in **uni_human_nr**, HPLN1_HUMAN Hyaluronan and proteoglycan link protein 1 OS=Homo sapiens GN=HAPLN1 PE=2 SV=2

Match to Query 16289: 2791.277296 from(698.826600,4+) intensity(1876208.0000) rtinseconds(3424) scans(23168) index(19046)
Title: 130801_HPL_Human_Plaque_BR1_TR1_02_Spectrum043053_scans__23168_RTINSECONDS=3424
Data file E:\\RAW\\HumanPlaques\\mgf\\T\\1D\\T130801_HPL_Human_Plaque_BR1_TR1_02.mgf

Click mouse within plot area to zoom in by factor of two about that point
Or, Plot from 0 to 2400 Da Full range
Label all possible matches ○ Label matches used for scoring ⊙
Show Y-axis ☐

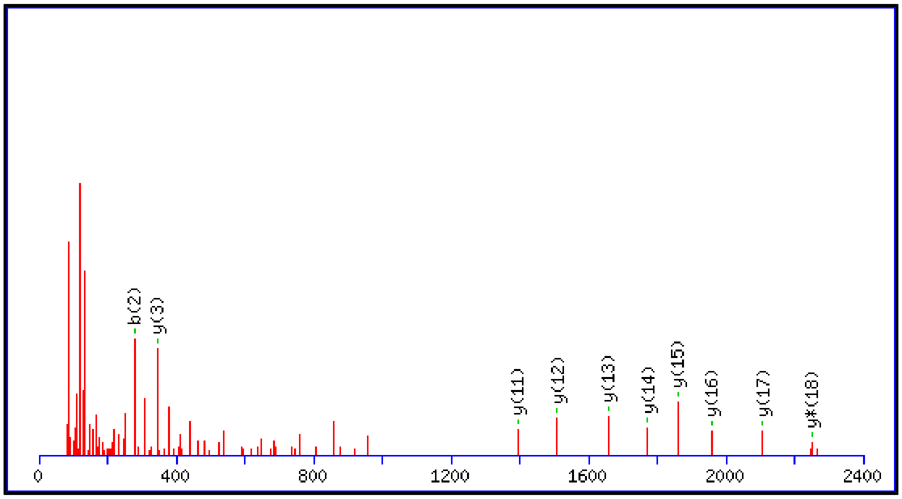

Monoisotopic mass of neutral peptide Mr(calc): 2790.2788
Fixed modifications: Carbamidomethyl (C) (apply to specified residues or termini only)
Variable modifications:
N9 : Deamidated (NQ)
N11 : Deamidated (NQ)
Ions Score: 43 **Expect:** 0.0036
Matches : 10/232 fragment ions using 17 most intense peaks (help)

#	b	b^{++}	b*	b^{*++}	b^{0}	b^{0++}	Seq.	y	y^{++}	y*	y^{*++}	y^{0}	y^{0++}	#
1	164.0706	82.5389					Y							22
2	**279.0975**	140.0524			261.0870	131.0471	D	2628.2228	1314.6150	2611.1962	1306.1018	2610.2122	1305.6097	21
3	378.1660	189.5866			360.1554	180.5813	V	2513.1958	1257.1016	2496.1693	1248.5883	2495.1853	1248.0963	20
4	525.2344	263.1208			507.2238	254.1155	F	2414.1274	1207.5673	2397.1009	1199.0541	2396.1169	1198.5621	19
5	685.2650	343.1362			667.2545	334.1309	C	2267.0590	1134.0331	2250.0325	1125.5199	2249.0484	1125.0279	18
6	832.3334	416.6704			814.3229	407.6651	F	***2107.0284***	1054.0178	2090.0018	1045.5045	2089.0178	1045.0125	17
7	933.3811	467.1942			915.3706	458.1889	T	***1959.9599***	980.4836	1942.9334	971.9703	1941.9494	971.4783	16
8	1020.4131	510.7102			1002.4026	501.7049	S	***1858.9123***	929.9598	1841.8857	921.4465	1840.9017	920.9545	15
9	1135.4401	568.2237	1118.4135	559.7104	1117.4295	559.2184	N	***1771.8802***	886.4438	1754.8537	877.9305	1753.8697	877.4385	14
10	1282.5085	641.7579	1265.4820	633.2446	1264.4979	632.7526	F	***1656.8533***	828.9303	1639.8267	820.4170	1638.8427	819.9250	13
11	1397.5354	699.2714	1380.5089	690.7581	1379.5249	690.2661	N	***1509.7849***	755.3961	1492.7583	746.8828	1491.7743	746.3908	12
12	1454.5569	727.7821	1437.5304	719.2688	1436.5463	718.7768	G	***1394.7579***	697.8826	1377.7314	689.3693	1376.7474	688.8773	11
13	1610.6580	805.8326	1593.6315	797.3194	1592.6475	796.8274	R	1337.7365	669.3719	1320.7099	660.8586	1319.7259	660.3666	10
14	1757.7264	879.3669	1740.6999	870.8536	1739.7159	870.3616	F	1181.6354	591.3213	1164.6088	582.8080	1163.6248	582.3160	9
15	1920.7898	960.8985	1903.7632	952.3852	1902.7792	951.8932	Y	1034.5669	517.7871	1017.5404	509.2738	1016.5564	508.7818	8
16	2083.8531	1042.4302	2066.8265	1033.9169	2065.8425	1033.4249	Y	871.5036	436.2554	854.4771	427.7422	853.4931	427.2502	7
17	2196.9372	1098.9722	2179.9106	1090.4589	2178.9266	1089.9669	L	708.4403	354.7238	691.4137	346.2105	690.4297	345.7185	6

**18**	2310.0212	1155.5142	2292.9947	1147.0010	2292.0107	1146.5090	**I**	595.3562	298.1817	578.3297	289.6685	577.3457	289.1765	**5**
**19**	2447.0801	1224.0437	2430.0536	1215.5304	2429.0696	1215.0384	**H**	482.2722	241.6397	465.2456	233.1264	464.2616	232.6344	**4**
**20**	2544.1329	1272.5701	2527.1063	1264.0568	2526.1223	1263.5648	**P**	***345.2132***	173.1103	328.1867	164.5970	327.2027	164.1050	**3**
**21**	2645.1806	1323.0939	2628.1540	1314.5807	2627.1700	1314.0886	**T**	248.1605	124.5839	231.1339	116.0706	230.1499	115.5786	**2**
**22**							**K**	147.1128	74.0600	130.0863	65.5468			**1**

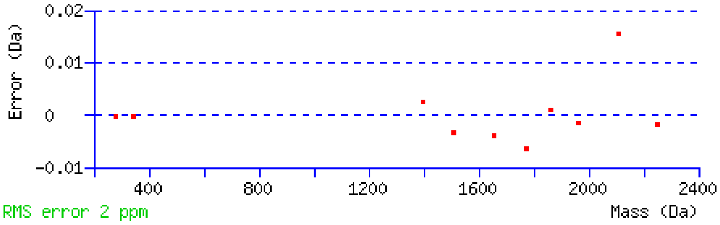

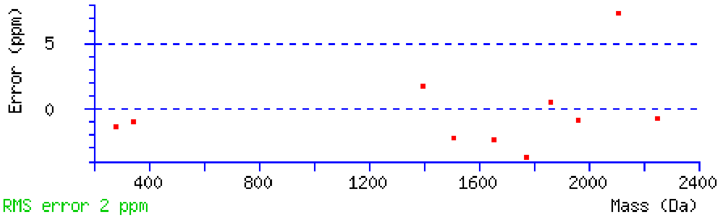

NCBI **BLAST** search of YDVFCFTSNFNGRFYYLIHPTK
(Parameters: blastp, nr protein database, expect=20000, no filter, PAM30)
Other BLAST web gateways

All matches to this query

Score	Mr(calc)	Delta	Sequence
42.6	2790.2788	0.9985	YDVFCFTSNFNGRFYYLIHPTK

Mascot: http://www.matrixscience.com/

Mascot Search Results

Peptide View

MS/MS Fragmentation of **LLQDFFNGR**
Found in **P0DMV8** in **uni_human_nr**, HS71A_HUMAN Heat shock 70 kDa protein 1A OS=Homo sapiens GN=HSPA1A PE=1 SV=1

Match to Query 1104: 1109.551248 from(555.782900,2+) intensity(488457.7500) rtinseconds(2446) scans(14245) index(10984)
Title: 130801_HPL_Human_Plaque_BR2_TR1_06_Spectrum032478_scans__14245_RTINSECONDS=2446
Data file E:\\RAW\\HumanPlaques\\mgf\\T\\1D\\T130801_HPL_Human_Plaque_BR2_TR1_06.mgf

Click mouse within plot area to zoom in by factor of two about that point
Or, Plot from 50 to 1000 Da Full range
Label all possible matches ○ Label matches used for scoring ⦿
Show Y-axis ☐

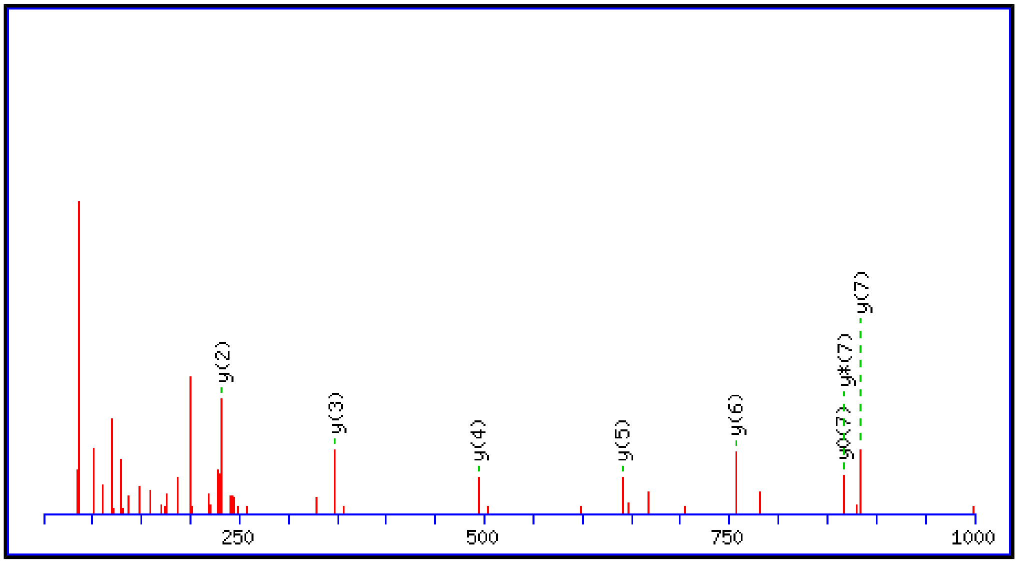

Monoisotopic mass of neutral peptide Mr(calc): 1109.5506
Fixed modifications: Carbamidomethyl (C) (apply to specified residues or termini only)
Variable modifications:
N7 : Deamidated (NQ)
Ions Score: 36 **Expect:** 0.012
Matches : 8/76 fragment ions using 16 most intense peaks (help)

#	b	b++	b*	b*++	b0	b0++	Seq.	y	y++	y*	y*++	y0	y0++	#
1	114.0913	57.5493					L							9
2	227.1754	114.0913					L	997.4738	499.2405	980.4472	490.7272	979.4632	490.2352	8
3	355.2340	178.1206	338.2074	169.6074			Q	***884.3897***	442.6985	867.3632	434.1852	866.3791	433.6932	7
4	470.2609	235.6341	453.2344	227.1208	452.2504	226.6288	D	***756.3311***	378.6692	739.3046	370.1559	738.3206	369.6639	6
5	617.3293	309.1683	600.3028	300.6550	599.3188	300.1630	F	***641.3042***	321.1557	624.2776	312.6425			5
6	764.3978	382.7025	747.3712	374.1892	746.3872	373.6972	F	***494.2358***	247.6215	477.2092	239.1083			4
7	879.4247	440.2160	862.3981	431.7027	861.4141	431.2107	N	***347.1674***	174.0873	330.1408	165.5740			3
8	936.4462	468.7267	919.4196	460.2134	918.4356	459.7214	G	***232.1404***	116.5738	215.1139	108.0606			2
9							R	175.1190	88.0631	158.0924	79.5498			1

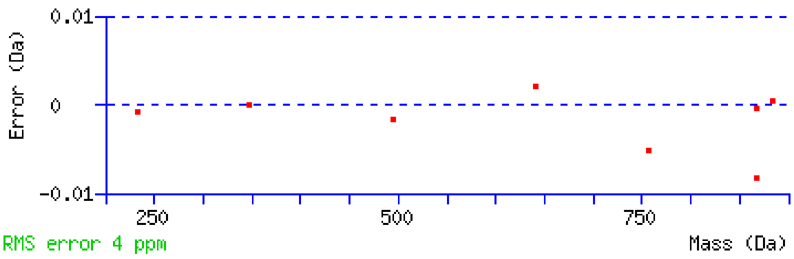

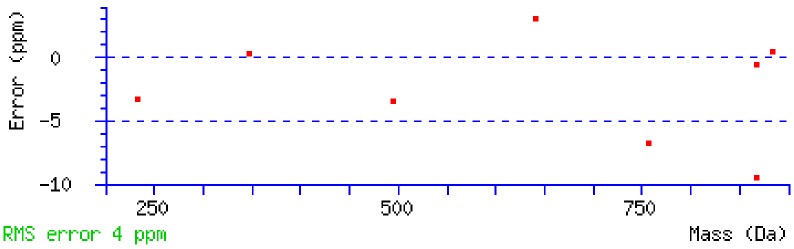

NCBI **BLAST** search of LLQDFFNGR
(Parameters: blastp, nr protein database, expect=20000, no filter, PAM30)
Other BLAST web gateways

All matches to this query

Score	Mr(calc)	Delta	Sequence
36.2	1109.5506	0.0007	LLQDFFNGR
4.3	1107.5448	2.0065	KEDLEFQAK

Mascot: http://www.matrixscience.com/

Mascot Search Results

Peptide View

MS/MS Fragmentation of **LLQDFFNGR**
Found in **P0DMV8** in **uni_human_nr**, HS71A_HUMAN Heat shock 70 kDa protein 1A OS=Homo sapiens GN=HSPA1A PE=1 SV=1

Match to Query 1262: 1108.567608 from(555.291080,2+) intensity(19745542.0000) rtinseconds(2229) scans(14416) index(11494)
Title: 130809_HPL_Human_Plaque_BR1_TR3_02_Spectrum036020_scans__14416_RTINSECONDS=2229
Data file E:\\RAW\\HumanPlaques\\mgf\\T\\1D\\T130809_HPL_Human_Plaque_BR1_TR3_02.mgf

Click mouse within plot area to zoom in by factor of two about that point
Or, Plot from 50 to 1000 Da Full range
Label all possible matches ○ Label matches used for scoring ⦿
Show Y-axis ☐

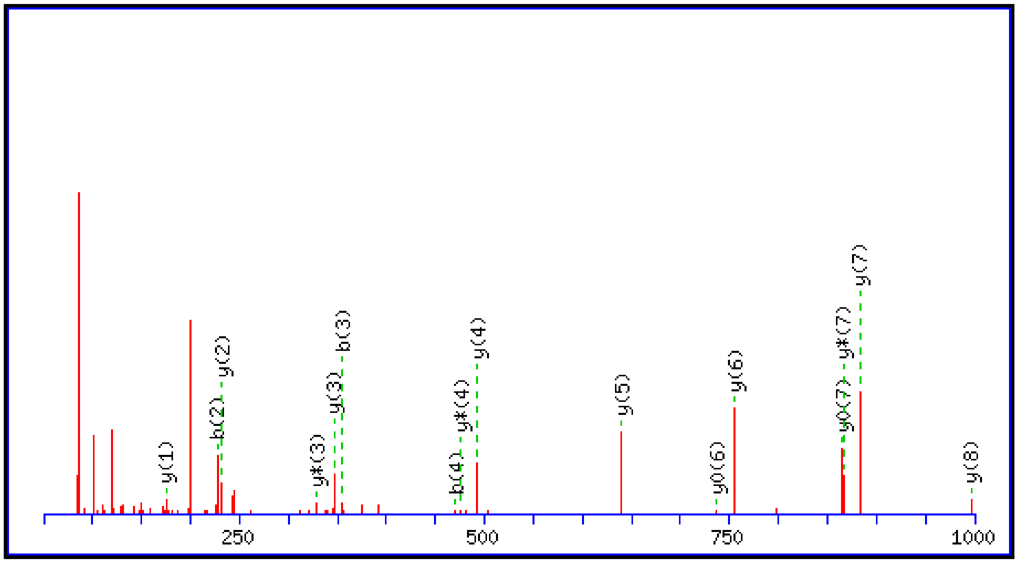

Monoisotopic mass of neutral peptide Mr(calc): 1108.5665
Fixed modifications: Carbamidomethyl (C) (apply to specified residues or termini only)
Ions Score: 61 **Expect:** 4.5e-005
Matches : 16/76 fragment ions using 30 most intense peaks (help)

#	b	b^{++}	b*	b^{*++}	b^0	b^{0++}	Seq.	y	y^{++}	y*	y^{*++}	y^0	y^{0++}	#
1	114.0913	57.5493					L							9
2	**227.1754**	114.0913					L	***996.4898***	498.7485	979.4632	490.2352	978.4792	489.7432	8
3	**355.2340**	178.1206	338.2074	169.6074			Q	***883.4057***	442.2065	866.3791	433.6932	865.3951	433.2012	7
4	**470.2609**	235.6341	453.2344	227.1208	452.2504	226.6288	D	***755.3471***	378.1772	738.3206	369.6639	737.3365	369.1719	6
5	617.3293	309.1683	600.3028	300.6550	599.3188	300.1630	F	***640.3202***	320.6637	623.2936	312.1504			5
6	764.3978	382.7025	747.3712	374.1892	746.3872	373.6972	F	***493.2518***	247.1295	476.2252	238.6162			4
7	878.4407	439.7240	861.4141	431.2107	860.4301	430.7187	N	***346.1833***	173.5953	329.1568	165.0820			3
8	935.4621	468.2347	918.4356	459.7214	917.4516	459.2294	G	***232.1404***	116.5738	215.1139	108.0606			2
9							R	***175.1190***	88.0631	158.0924	79.5498			1

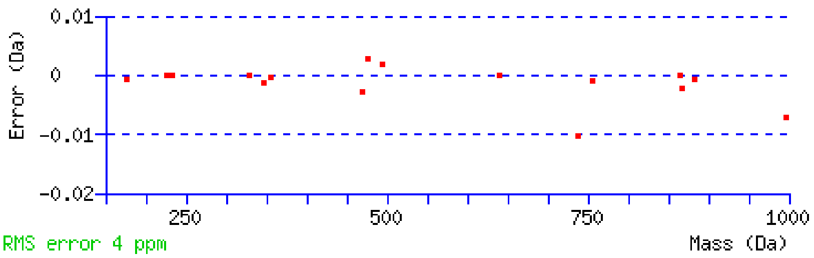

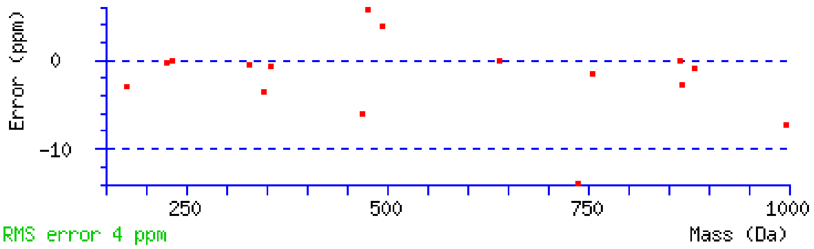

NCBI **BLAST** search of LLQDFFNGR
(Parameters: blastp, nr protein database, expect=20000, no filter, PAM30)
Other BLAST web gateways

All matches to this query

Score	Mr(calc)	Delta	Sequence
61.5	1108.5665	0.0011	LLQDFFNGR
11.7	1108.5625	0.0051	ILAGQHEDAR
10.7	1108.5733	-0.0056	IIKNTAMMR
5.8	1108.5659	0.0017	LSRLMSGSSR
5.5	1106.5556	2.0120	CGKFFRHR
2.2	1108.5699	-0.0023	ILNKYGDMR
2.2	1108.5699	-0.0023	ILREMYER
1.3	1106.5650	2.0026	LGLICMCLK

Mascot: http://www.matrixscience.com/

Mascot Search Results

Peptide View

MS/MS Fragmentation of **LLQDFFNGRDLNK**
Found in **P0DMV8** in **uni_human_nr**, HS71A_HUMAN Heat shock 70 kDa protein 1A OS=Homo sapiens GN=HSPA1A PE=1 SV=1

Match to Query 3788: 1582.763802 from(528.595210,3+) intensity(202067.5781) rtinseconds(1924) scans(10132) index(7344)
Title: 130806_HPL_Human_Plaque_BR2_TR2_13_Spectrum027977_scans__10132_RTINSECONDS=1924
Data file E:\\RAW\\HumanPlaques\\mgf\\T\\1D\\T130806_HPL_Human_Plaque_BR2_TR2_13.mgf

Click mouse within plot area to zoom in by factor of two about that point
Or, Plot from 0 to 1500 Da Full range
Label all possible matches ○ Label matches used for scoring ⊙
Show Y-axis ☐

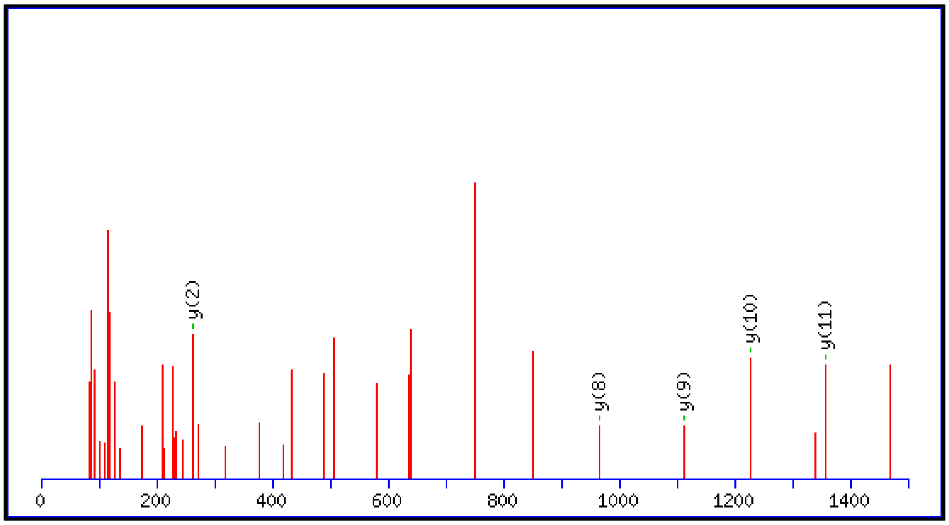

Monoisotopic mass of neutral peptide Mr(calc): 1581.7675
Fixed modifications: Carbamidomethyl (C) (apply to specified residues or termini only)
Variable modifications:
Q3 : Deamidated (NQ)
N7 : Deamidated (NQ)
N12 : Deamidated (NQ)
Ions Score: 17 **Expect:** 1.3
Matches : 5/128 fragment ions using 13 most intense peaks (help)

#	b	b^{++}	b*	b^{*++}	b^{0}	b^{0++}	Seq.	y	y^{++}	y*	y^{*++}	y^{0}	y^{0++}	#
1	114.0913	57.5493					L							13
2	227.1754	114.0913					L	1469.6907	735.3490	1452.6642	726.8357	1451.6801	726.3437	12
3	356.2180	178.6126	339.1914	170.0994			Q	***1356.6066***	678.8070	1339.5801	670.2937	1338.5961	669.8017	11
4	471.2449	236.1261	454.2184	227.6128	453.2344	227.1208	D	***1227.5640***	614.2857	1210.5375	605.7724	1209.5535	605.2804	10
5	618.3134	309.6603	601.2868	301.1470	600.3028	300.6550	F	***1112.5371***	556.7722	1095.5106	548.2589	1094.5265	547.7669	9
6	765.3818	383.1945	748.3552	374.6812	747.3712	374.1892	F	***965.4687***	483.2380	948.4421	474.7247	947.4581	474.2327	8
7	880.4087	440.7080	863.3822	432.1947	862.3981	431.7027	N	818.4003	409.7038	801.3737	401.1905	800.3897	400.6985	7
8	937.4302	469.2187	920.4036	460.7055	919.4196	460.2134	G	703.3733	352.1903	686.3468	343.6770	685.3628	343.1850	6
9	1093.5313	547.2693	1076.5047	538.7560	1075.5207	538.2640	R	646.3519	323.6796	629.3253	315.1663	628.3413	314.6743	5
10	1208.5582	604.7828	1191.5317	596.2695	1190.5477	595.7775	D	490.2508	245.6290	473.2242	237.1157	472.2402	236.6237	4
11	1321.6423	661.3248	1304.6157	652.8115	1303.6317	652.3195	L	375.2238	188.1155	358.1973	179.6023			3
12	1436.6692	718.8383	1419.6427	710.3250	1418.6587	709.8330	N	***262.1397***	131.5735	245.1132	123.0602			2
13							K	147.1128	74.0600	130.0863	65.5468			1

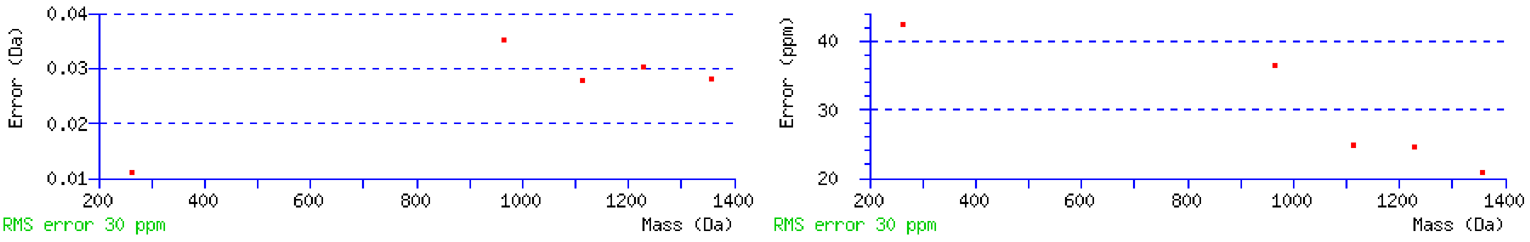

NCBI **BLAST** search of LLQDFFNGRDLNK
(Parameters: blastp, nr protein database, expect=20000, no filter, PAM30)
Other BLAST web gateways

All matches to this query

Score	Mr(calc)	Delta	Sequence
16.9	1581.7675	0.9963	LLQDFFNGRDLNK
0.1	1580.7583	2.0055	PQTGANNLFFGTGTR
0.1	1580.7583	2.0055	PQTGANNLFFGTGTR

Mascot: http://www.matrixscience.com/

Mascot Search Results

Peptide View

MS/MS Fragmentation of **LLQDFFNGRDLNK**
Found in **P0DMV8** in **uni_human_nr**, HS71A_HUMAN Heat shock 70 kDa protein 1A OS=Homo sapiens GN=HSPA1A PE=1 SV=1

Match to Query 3943: 1580.781882 from(527.934570,3+) intensity(152219.7031) rtinseconds(2480) scans(14092) index(10686)
Title: 130806_HPL_Human_Plaque_BR2_TR2_10_Spectrum031607_scans__14092_RTINSECONDS=2480
Data file E:\\RAW\\HumanPlaques\\mgf\\T\\1D\\T130806_HPL_Human_Plaque_BR2_TR2_10.mgf

Click mouse within plot area to zoom in by factor of two about that point
Or, Plot from 0 to 1400 Da Full range
Label all possible matches ○ Label matches used for scoring ⊙
Show Y-axis ☐

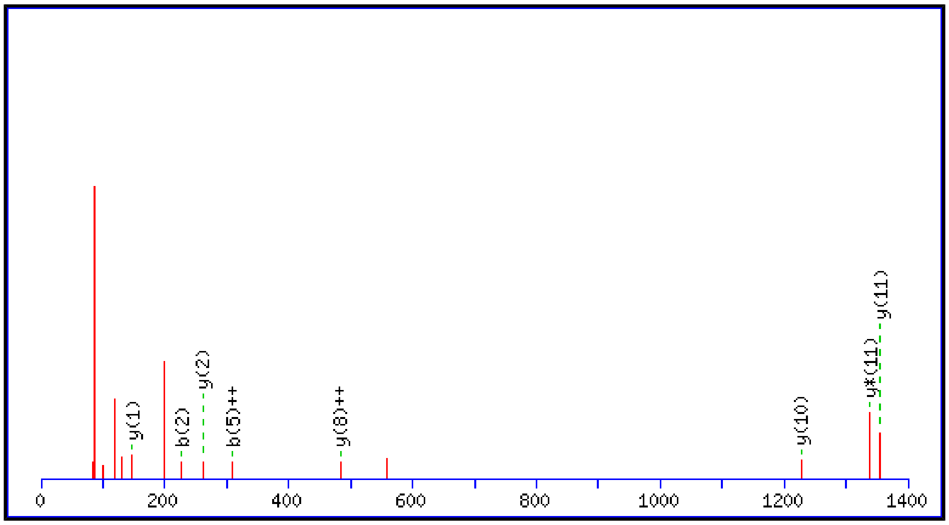

Monoisotopic mass of neutral peptide Mr(calc): 1580.7834
Fixed modifications: Carbamidomethyl (C) (apply to specified residues or termini only)
Variable modifications:
N7 : Deamidated (NQ)
N12 : Deamidated (NQ)
Ions Score: 20 **Expect:** 0.7
Matches : 8/128 fragment ions using 12 most intense peaks (help)

#	b	b^{++}	b*	b^{*++}	b^{0}	b^{0++}	Seq.	y	y^{++}	y*	y^{*++}	y^{0}	y^{0++}	#
1	114.0913	57.5493					L							13
2	**227.1754**	114.0913					L	1468.7067	734.8570	1451.6801	726.3437	1450.6961	725.8517	12
3	355.2340	178.1206	338.2074	169.6074			Q	***1355.6226***	678.3149	1338.5961	669.8017	1337.6121	669.3097	11
4	470.2609	235.6341	453.2344	227.1208	452.2504	226.6288	D	***1227.5640***	614.2857	1210.5375	605.7724	1209.5535	605.2804	10
5	617.3293	**309.1683**	600.3028	300.6550	599.3188	300.1630	F	1112.5371	556.7722	1095.5106	548.2589	1094.5265	547.7669	9
6	764.3978	382.7025	747.3712	374.1892	746.3872	373.6972	F	965.4687	483.2380	948.4421	474.7247	947.4581	474.2327	8
7	879.4247	440.2160	862.3981	431.7027	861.4141	431.2107	N	818.4003	409.7038	801.3737	401.1905	800.3897	400.6985	7
8	936.4462	468.7267	919.4196	460.2134	918.4356	459.7214	G	703.3733	352.1903	686.3468	343.6770	685.3628	343.1850	6
9	1092.5473	546.7773	1075.5207	538.2640	1074.5367	537.7720	R	646.3519	323.6796	629.3253	315.1663	628.3413	314.6743	5
10	1207.5742	604.2907	1190.5477	595.7775	1189.5636	595.2855	D	490.2508	245.6290	473.2242	237.1157	472.2402	236.6237	4
11	1320.6583	660.8328	1303.6317	652.3195	1302.6477	651.8275	L	375.2238	188.1155	358.1973	179.6023			3
12	1435.6852	718.3462	1418.6587	709.8330	1417.6747	709.3410	N	***262.1397***	131.5735	245.1132	123.0602			2
13							K	***147.1128***	74.0600	130.0863	65.5468			1

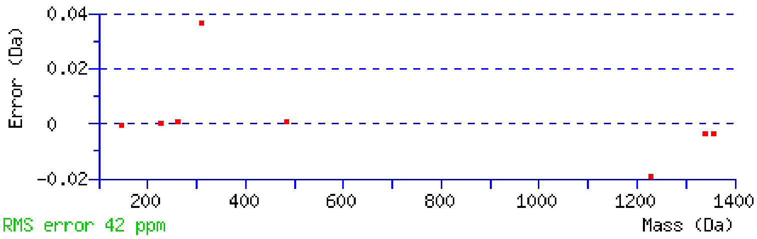

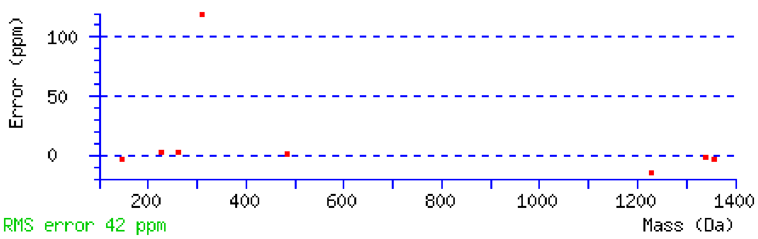

NCBI **BLAST** search of LLQDFFNGRDLNK
(Parameters: blastp, nr protein database, expect=20000, no filter, PAM30)
Other BLAST web gateways

All matches to this query

Score	Mr(calc)	Delta	Sequence
20.2	1580.7834	-0.0016	LLQDFFNGRDLNK
6.8	1580.7834	-0.0016	LLQDFFNGRDLNK
2.8	1580.7769	0.0050	LGFPGARDLANMFR
2.7	1579.7803	1.0016	MPQLLSVYLEENK
2.0	1578.7777	2.0042	AVAGEYSDPVTLETK
1.4	1579.7842	0.9977	IGSLDNITHVPGGGNK

Mascot: http://www.matrixscience.com/

Mascot Search Results

Peptide View

MS/MS Fragmentation of **LLQDFFNGRDLNK**
Found in **P0DMV8** in **uni_human_nr**, HS71A_HUMAN Heat shock 70 kDa protein 1A OS=Homo sapiens GN=HSPA1A PE=1 SV=1

Match to Query 4257: 1579.798962 from(527.606930,3+) intensity(2126163.0000) rtinseconds(2356) scans(14112) index(11024)
Title: 130801_HPL_Human_Plaque_BR1_TR1_05_Spectrum033530_scans__14112_RTINSECONDS=2356
Data file E:\\RAW\\HumanPlaques\\mgf\\T\\1D\\T130801_HPL_Human_Plaque_BR1_TR1_05.mgf

Click mouse within plot area to zoom in by factor of two about that point
Or, [Plot from] [0] to [1500] Da [Full range]
Label all possible matches ○ Label matches used for scoring ⊙
Show Y-axis ☐

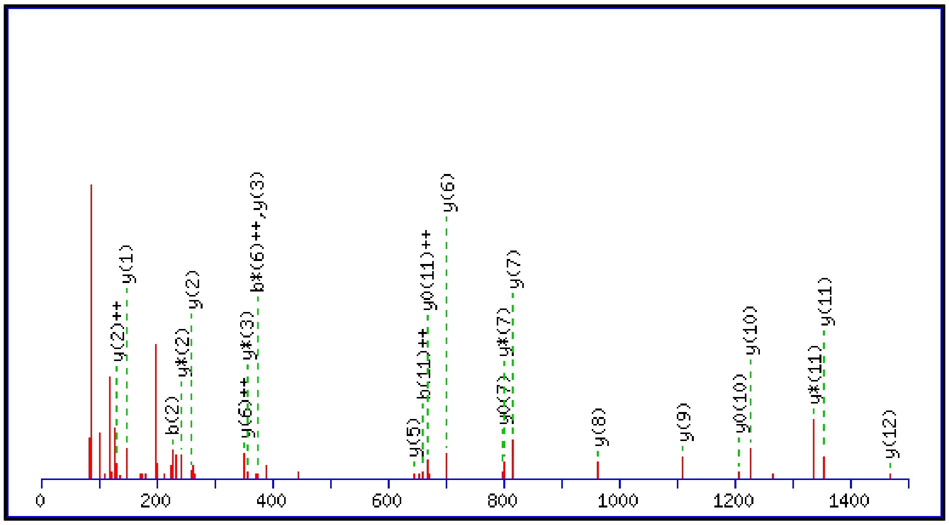


```
Monoisotopic mass of neutral peptide Mr(calc): 1579.7994
Fixed modifications: Carbamidomethyl (C) (apply to specified residues or termini only)
Variable modifications:
N7      : Deamidated (NQ)
Ions Score: 76  Expect: 1.7e-006
Matches : 23/128 fragment ions using 42 most intense peaks   (help)
```

#	b	b++	b*	b*++	b0	b0++	Seq.	y	y++	y*	y*++	y0	y0++	#
1	114.0913	57.5493					L							13
2	**227.1754**	114.0913					L	***1467.7227***	734.3650	1450.6961	725.8517	1449.7121	725.3597	12
3	355.2340	178.1206	338.2074	169.6074			Q	***1354.6386***	677.8229	1337.6121	669.3097	1336.6280	668.8177	11
4	470.2609	235.6341	453.2344	227.1208	452.2504	226.6288	D	***1226.5800***	613.7937	1209.5535	605.2804	1208.5695	604.7884	10
5	617.3293	309.1683	600.3028	300.6550	599.3188	300.1630	F	***1111.5531***	556.2802	1094.5265	547.7669	1093.5425	547.2749	9
6	764.3978	382.7025	747.3712	374.1892	746.3872	373.6972	F	***964.4847***	482.7460	947.4581	474.2327	946.4741	473.7407	8
7	879.4247	440.2160	862.3981	431.7027	861.4141	431.2107	N	***817.4163***	409.2118	800.3897	400.6985	799.4057	400.2065	7
8	936.4462	468.7267	919.4196	460.2134	918.4356	459.7214	G	***702.3893***	351.6983	685.3628	343.1850	684.3787	342.6930	6
9	1092.5473	546.7773	1075.5207	538.2640	1074.5367	537.7720	R	***645.3678***	323.1876	628.3413	314.6743	627.3573	314.1823	5
10	1207.5742	604.2907	1190.5477	595.7775	1189.5636	595.2855	D	489.2667	245.1370	472.2402	236.6237	471.2562	236.1317	4
11	1320.6583	**660.8328**	1303.6317	652.3195	1302.6477	651.8275	L	***374.2398***	187.6235	357.2132	179.1103			3
12	1434.7012	717.8542	1417.6747	709.3410	1416.6906	708.8490	N	***261.1557***	131.0815	244.1292	122.5682			2
13							K	***147.1128***	74.0600	130.0863	65.5468			1

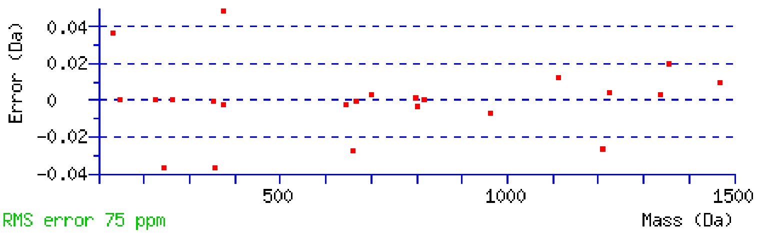

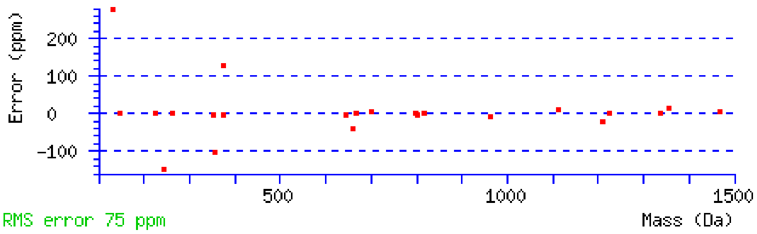

NCBI **BLAST** search of LLQDFFNGRDLNK
(Parameters: blastp, nr protein database, expect=20000, no filter, PAM30)
Other BLAST web gateways

All matches to this query

Score	Mr(calc)	Delta	Sequence	Site Analysis
76.4	1579.7994	-0.0005	LLQDFFNGRDLNK	Deamidated N7 100.00%
30.3	1579.7994	-0.0005	LLQDFFNGRDLNK	Deamidated N12 0.00%
24.2	1579.7994	-0.0005	LLQDFFNGRDLNK	Deamidated Q3 0.00%

Mascot: http://www.matrixscience.com/

Mascot Search Results

Peptide View

MS/MS Fragmentation of **LLQDFFNGRDLNK**
Found in **P0DMV8** in **uni_human_nr**, HS71A_HUMAN Heat shock 70 kDa protein 1A OS=Homo sapiens GN=HSPA1A PE=1 SV=1

Match to Query 5517: 1578.811748 from(790.413150,2+) intensity(5705839.0000) rtinseconds(2118) scans(13496) index(10694)
Title: 130806_HPL_Human_Plaque_BR1_TR2_02_Spectrum035398_scans__13496_RTINSECONDS=2118
Data file E:\\RAW\\HumanPlaques\\mgf\\T\\1D\\T130806_HPL_Human_Plaque_BR1_TR2_02.mgf

Click mouse within plot area to zoom in by factor of two about that point
Or, [Plot from] [0] to [1600] Da [Full range]
Label all possible matches ○ Label matches used for scoring ⦿
Show Y-axis ☐

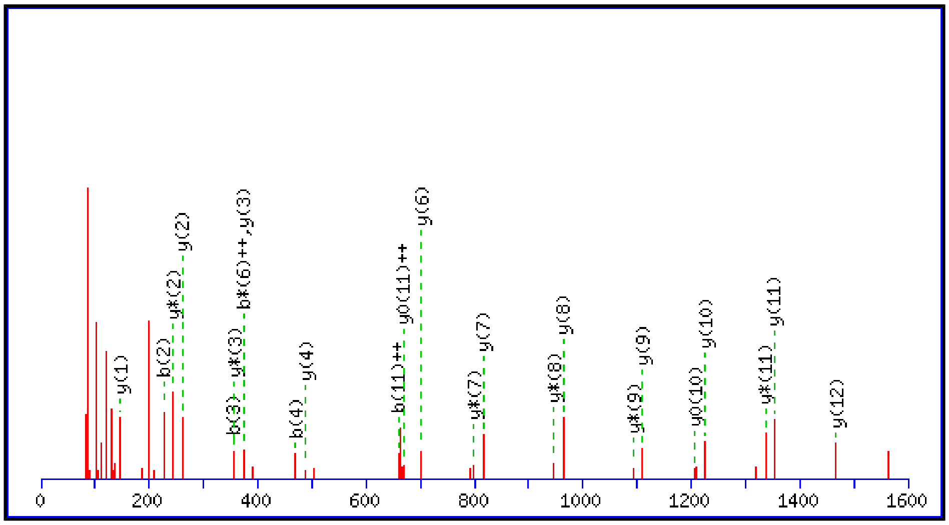

Monoisotopic mass of neutral peptide Mr(calc): 1578.8154
Fixed modifications: Carbamidomethyl (C) (apply to specified residues or termini only)
Ions Score: 78 **Expect:** 1.3e-006
Matches : 24/128 fragment ions using 44 most intense peaks (help)

#	b	b^{++}	b^{*}	b^{*++}	b^{0}	b^{0++}	Seq.	y	y^{++}	y^{*}	y^{*++}	y^{0}	y^{0++}	#
1	114.0913	57.5493					L							13
2	**227.1754**	114.0913					L	***1466.7387***	733.8730	1449.7121	725.3597	1448.7281	724.8677	12
3	**355.2340**	178.1206	338.2074	169.6074			Q	***1353.6546***	677.3309	1336.6280	668.8177	1335.6440	668.3257	11
4	**470.2609**	235.6341	453.2344	227.1208	452.2504	226.6288	D	***1225.5960***	613.3016	1208.5695	604.7884	1207.5854	604.2964	10
5	617.3293	309.1683	600.3028	300.6550	599.3188	300.1630	F	***1110.5691***	555.7882	1093.5425	547.2749	1092.5585	546.7829	9
6	764.3978	382.7025	747.3712	374.1892	746.3872	373.6972	F	***963.5007***	482.2540	946.4741	473.7407	945.4901	473.2487	8
7	878.4407	439.7240	861.4141	431.2107	860.4301	430.7187	N	***816.4322***	408.7198	799.4057	400.2065	798.4217	399.7145	7
8	935.4621	468.2347	918.4356	459.7214	917.4516	459.2294	G	***702.3893***	351.6983	685.3628	343.1850	684.3787	342.6930	6
9	1091.5633	546.2853	1074.5367	537.7720	1073.5527	537.2800	R	645.3678	323.1876	628.3413	314.6743	627.3573	314.1823	5
10	1206.5902	603.7987	1189.5636	595.2855	1188.5796	594.7935	D	***489.2667***	245.1370	472.2402	236.6237	471.2562	236.1317	4
11	1319.6743	**660.3408**	1302.6477	651.8275	1301.6637	651.3355	L	***374.2398***	187.6235	357.2132	179.1103			3
12	1433.7172	717.3622	1416.6906	708.8490	1415.7066	708.3569	N	***261.1557***	131.0815	244.1292	122.5682			2
13							K	***147.1128***	74.0600	130.0863	65.5468			1

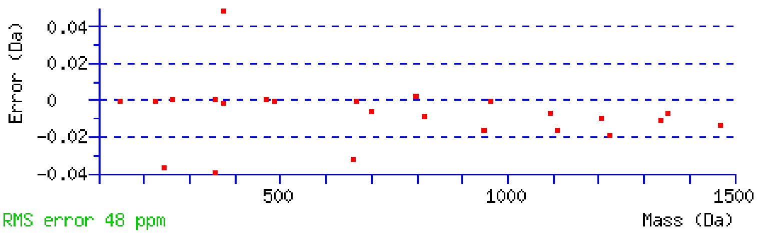

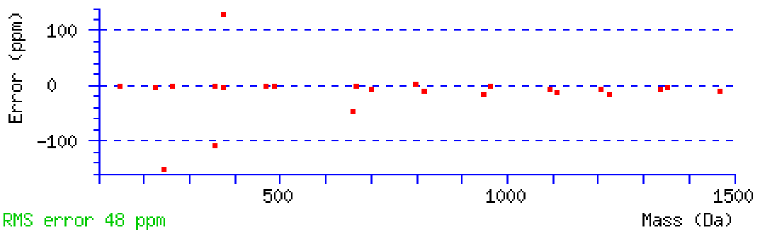

NCBI **BLAST** search of LLQDFFNGRDLNK
(Parameters: blastp, nr protein database, expect=20000, no filter, PAM30)
Other BLAST web gateways

All matches to this query

Score	Mr(calc)	Delta	Sequence
77.9	1578.8154	-0.0037	LLQDFFNGRDLNK
9.8	1578.8076	0.0042	DKLLAMGSGNFGEIK
8.9	1578.8042	0.0075	IIFIDSTWNQTNK
4.7	1576.7984	2.0133	EKLDEFNELAIQK
2.7	1578.8114	0.0003	LLDAGGDLRLHDER
2.6	1577.8049	1.0068	KDLQDPNLQGEPPK

Mascot: http://www.matrixscience.com/

Mascot Search Results

Peptide View

MS/MS Fragmentation of **LLQDFFNGRDLNK**
Found in **P0DMV8** in **uni_human_nr**, HS71A_HUMAN Heat shock 70 kDa protein 1A OS=Homo sapiens GN=HSPA1A PE=1 SV=1

Match to Query 5268: 1578.811482 from(527.277770,3+) intensity(12430808.0000) rtinseconds(2099) scans(12947) index(9966)
Title: 130801_HPL_Human_Plaque_BR2_TR1_02_Spectrum034121_scans__12947_RTINSECONDS=2099
Data file E:\\RAW\\HumanPlaques\\mgf\\T\\1D\\T130801_HPL_Human_Plaque_BR2_TR1_02.mgf

Click mouse within plot area to zoom in by factor of two about that point
Or, Plot from 0 to 1600 Da Full range
Label all possible matches ○ Label matches used for scoring ⊙
Show Y-axis ☐

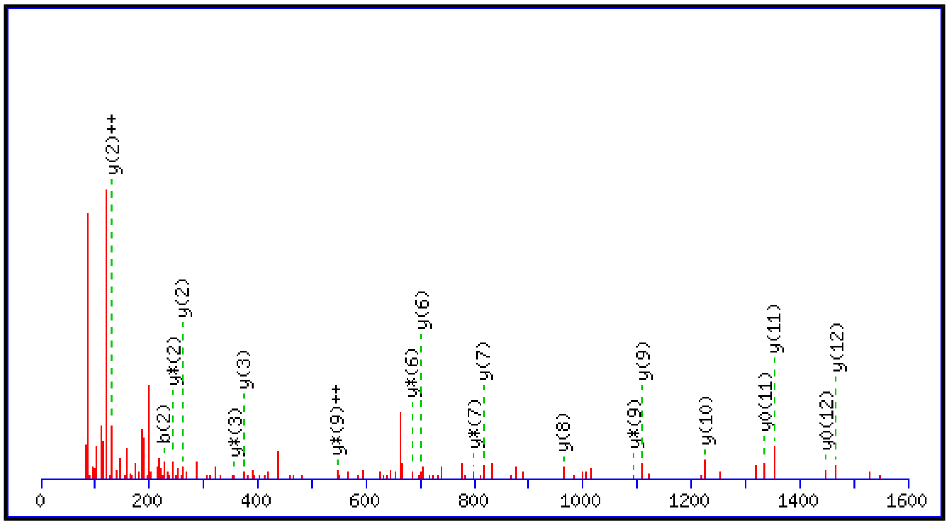

Monoisotopic mass of neutral peptide Mr(calc): 1578.8154
Fixed modifications: Carbamidomethyl (C) (apply to specified residues or termini only)
Ions Score: 21 **Expect:** 0.67
Matches : 19/128 fragment ions using 79 most intense peaks (help)

#	b	b^{++}	b*	b^{*++}	b^0	b^{0++}	Seq.	y	y^{++}	y*	y^{*++}	y^0	y^{0++}	#
1	114.0913	57.5493					L							13
2	227.1754	114.0913					L	***1466.7387***	733.8730	1449.7121	725.3597	1448.7281	724.8677	12
3	355.2340	178.1206	338.2074	169.6074			Q	***1353.6546***	677.3309	1336.6280	668.8177	1335.6440	668.3257	11
4	470.2609	235.6341	453.2344	227.1208	452.2504	226.6288	D	***1225.5960***	613.3016	1208.5695	604.7884	1207.5854	604.2964	10
5	617.3293	309.1683	600.3028	300.6550	599.3188	300.1630	F	***1110.5691***	555.7882	1093.5425	547.2749	1092.5585	546.7829	9
6	764.3978	382.7025	747.3712	374.1892	746.3872	373.6972	F	***963.5007***	482.2540	946.4741	473.7407	945.4901	473.2487	8
7	878.4407	439.7240	861.4141	431.2107	860.4301	430.7187	N	***816.4322***	408.7198	799.4057	400.2065	798.4217	399.7145	7
8	935.4621	468.2347	918.4356	459.7214	917.4516	459.2294	G	***702.3893***	351.6983	685.3628	343.1850	684.3787	342.6930	6
9	1091.5633	546.2853	1074.5367	537.7720	1073.5527	537.2800	R	645.3678	323.1876	628.3413	314.6743	627.3573	314.1823	5
10	1206.5902	603.7987	1189.5636	595.2855	1188.5796	594.7935	D	489.2667	245.1370	472.2402	236.6237	471.2562	236.1317	4
11	1319.6743	660.3408	1302.6477	651.8275	1301.6637	651.3355	L	***374.2398***	187.6235	357.2132	179.1103			3
12	1433.7172	717.3622	1416.6906	708.8490	1415.7066	708.3569	N	***261.1557***	131.0815	244.1292	122.5682			2
13							K	147.1128	74.0600	130.0863	65.5468			1

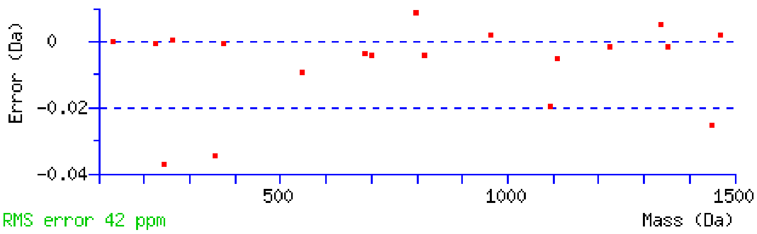

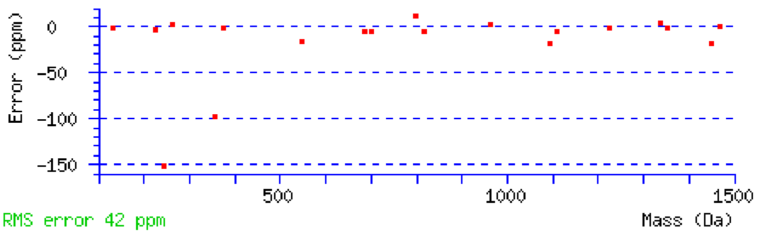

NCBI **BLAST** search of LLQDFFNGRDLNK
(Parameters: blastp, nr protein database, expect=20000, no filter, PAM30)
Other BLAST web gateways

All matches to this query

Score	Mr(calc)	Delta	Sequence
20.7	1578.8154	-0.0039	LLQDFFNGRDLNK

Mascot: http://www.matrixscience.com/

Mascot Search Results

Peptide View

MS/MS Fragmentation of **NGRVEIIANDQGNR**
Found in **P11021** in **uni_human_nr**, GRP78_HUMAN 78 kDa glucose-regulated protein OS=Homo sapiens GN=HSPA5 PE=1 SV=2

Match to Query 3830: 1555.769502 from(519.597110,3+) intensity(1626998.0000) rtinseconds(1252) scans(5936) index(3938)
Title: 130809_HPL_Human_Plaque_BR2_TR3_08_Spectrum025436_scans__5936_RTINSECONDS=1252
Data file E:\\RAW\\HumanPlaques\\mgf\\T\\1D\\T130809_HPL_Human_Plaque_BR2_TR3_08.mgf

Click mouse within plot area to zoom in by factor of two about that point
Or, Plot from 50 to 950 Da Full range
Label all possible matches ○ Label matches used for scoring ⊙
Show Y-axis ☐

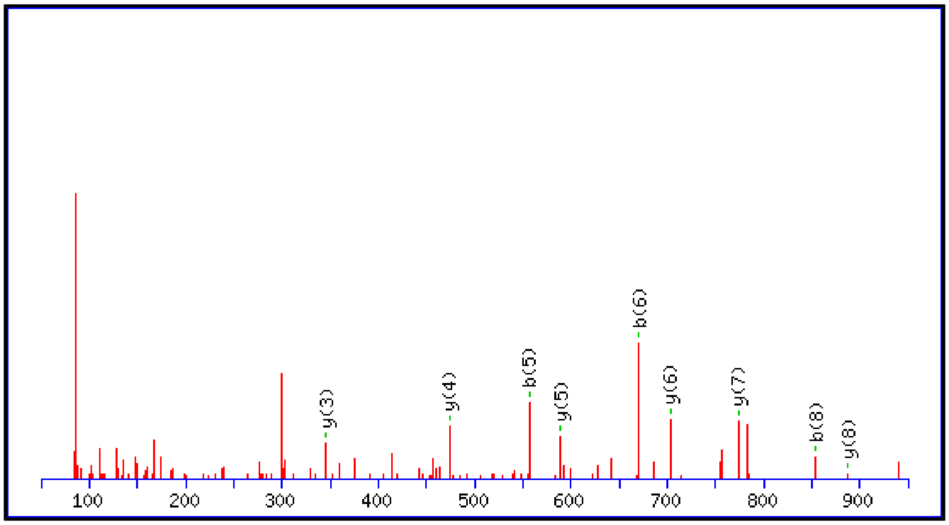

Monoisotopic mass of neutral peptide Mr(calc): 1555.7702
Fixed modifications: Carbamidomethyl (C) (apply to specified residues or termini only)
Variable modifications:
N1 : Deamidated (NQ)
Ions Score: 30 **Expect:** 0.07
Matches : 9/140 fragment ions using 18 most intense peaks (help)

#	b	b^{++}	b*	b^{*++}	b^{0}	b^{0++}	Seq.	y	y^{++}	y*	y^{*++}	y^{0}	y^{0++}	#
1	116.0342	58.5207	99.0077	50.0075			N							14
2	173.0557	87.0315	156.0291	78.5182			G	1441.7506	721.3789	1424.7241	712.8657	1423.7400	712.3737	13
3	329.1568	165.0820	312.1302	156.5688			R	1384.7292	692.8682	1367.7026	684.3549	1366.7186	683.8629	12
4	428.2252	214.6162	411.1987	206.1030			V	1228.6280	614.8177	1211.6015	606.3044	1210.6175	605.8124	11
5	**557.2678**	279.1375	540.2413	270.6243	539.2572	270.1323	E	1129.5596	565.2835	1112.5331	556.7702	1111.5491	556.2782	10
6	**670.3519**	335.6796	653.3253	327.1663	652.3413	326.6743	I	1000.5170	500.7622	983.4905	492.2489	982.5065	491.7569	9
7	783.4359	392.2216	766.4094	383.7083	765.4254	383.2163	I	***887.4330***	444.2201	870.4064	435.7068	869.4224	435.2148	8
8	**854.4730**	427.7402	837.4465	419.2269	836.4625	418.7349	A	***774.3489***	387.6781	757.3224	379.1648	756.3383	378.6728	7
9	968.5160	484.7616	951.4894	476.2483	950.5054	475.7563	N	***703.3118***	352.1595	686.2852	343.6463	685.3012	343.1543	6
10	1083.5429	542.2751	1066.5164	533.7618	1065.5323	533.2698	D	***589.2689***	295.1381	572.2423	286.6248	571.2583	286.1328	5
11	1211.6015	606.3044	1194.5749	597.7911	1193.5909	597.2991	Q	***474.2419***	237.6246	457.2154	229.1113			4
12	1268.6230	634.8151	1251.5964	626.3018	1250.6124	625.8098	G	***346.1833***	173.5953	329.1568	165.0820			3
13	1382.6659	691.8366	1365.6393	683.3233	1364.6553	682.8313	N	289.1619	145.0846	272.1353	136.5713			2
14							R	175.1190	88.0631	158.0924	79.5498			1

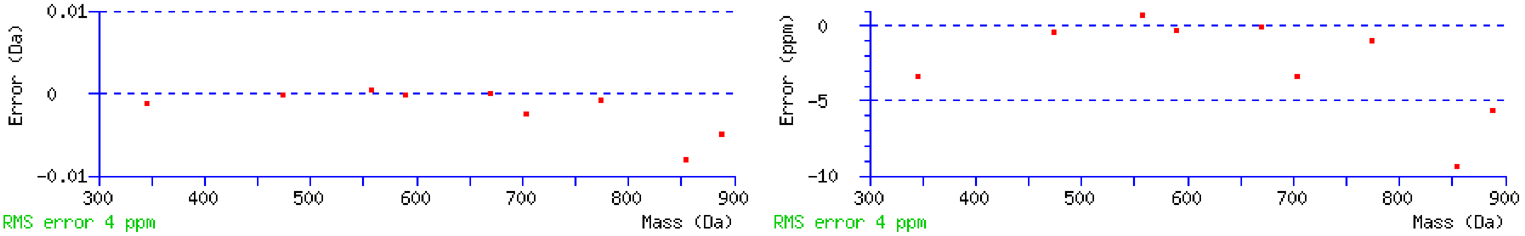

NCBI **BLAST** search of NGRVEIIANDQGNR
(Parameters: blastp, nr protein database, expect=20000, no filter, PAM30)
Other BLAST web gateways

All matches to this query

Score	Mr(calc)	Delta	Sequence
29.8	1555.7702	-0.0007	NGRVEIIANDQGNR

Mascot: http://www.matrixscience.com/

Mascot Search Results

Peptide View

MS/MS Fragmentation of **NGRVEIIANDQGNR**
Found in **P11021** in **uni_human_nr**, GRP78_HUMAN 78 kDa glucose-regulated protein OS=Homo sapiens GN=HSPA5 PE=1 SV=2

Match to Query 4101: 1554.786582 from(519.269470,3+) intensity(3866337.5000) rtinseconds(985) scans(3965) index(2116)
Title: 130801_HPL_Human_Plaque_BR1_TR1_04_Spectrum025114_scans__3965_RTINSECONDS=985
Data file E:\\RAW\\HumanPlaques\\mgf\\T\\1D\\T130801_HPL_Human_Plaque_BR1_TR1_04.mgf

Click mouse within plot area to zoom in by factor of two about that point
Or, Plot from 50 to 900 Da Full range
Label all possible matches ○ Label matches used for scoring ⦿
Show Y-axis ☐

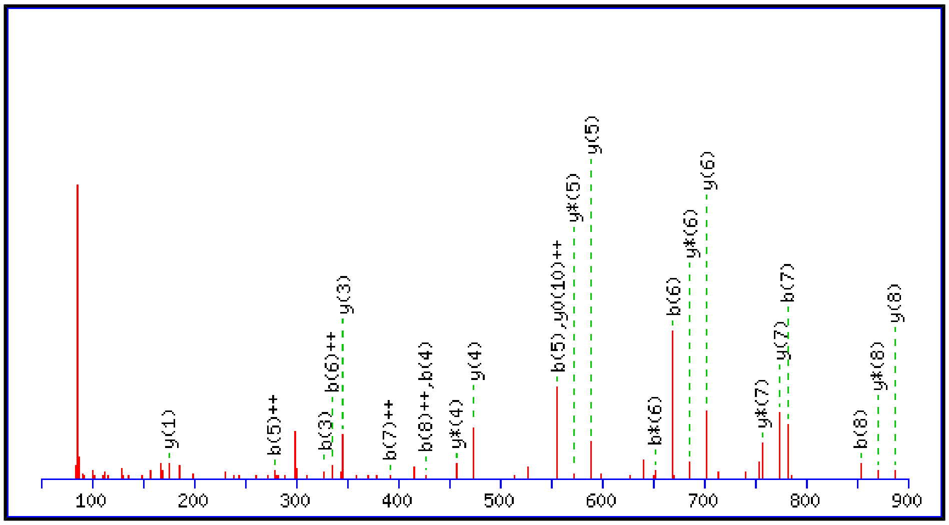

Monoisotopic mass of neutral peptide Mr(calc): 1554.7862
Fixed modifications: Carbamidomethyl (C) (apply to specified residues or termini only)
Ions Score: 46 **Expect:** 0.0017
Matches : 24/140 fragment ions using 38 most intense peaks (help)

#	b	b^{++}	b*	b^{*++}	b^0	b^{0++}	Seq.	y	y^{++}	y*	y^{*++}	y^0	y^{0++}	#
1	115.0502	58.0287	98.0237	49.5155			N							14
2	172.0717	86.5395	155.0451	78.0262			G	1441.7506	721.3789	1424.7241	712.8657	1423.7401	712.3737	13
3	**328.1728**	164.5900	311.1462	156.0768			R	1384.7292	692.8682	1367.7026	684.3549	1366.7186	683.8629	12
4	**427.2412**	214.1242	410.2146	205.6110			V	1228.6280	614.8177	1211.6015	606.3044	1210.6175	605.8124	11
5	**556.2838**	**278.6455**	539.2572	270.1323	538.2732	269.6402	E	1129.5596	565.2835	1112.5331	556.7702	1111.5491	556.2782	10
6	**669.3678**	**335.1876**	652.3413	326.6743	651.3573	326.1823	I	1000.5170	500.7622	983.4905	492.2489	982.5065	491.7569	9
7	**782.4519**	**391.7296**	765.4254	383.2163	764.4413	382.7243	I	**887.4330**	444.2201	870.4064	435.7068	869.4224	435.2148	8
8	**853.4890**	**427.2482**	836.4625	418.7349	835.4785	418.2429	A	**774.3489**	387.6781	757.3224	379.1648	756.3383	378.6728	7
9	967.5320	484.2696	950.5054	475.7563	949.5214	475.2643	N	**703.3118**	352.1595	686.2852	343.6463	685.3012	343.1543	6
10	1082.5589	541.7831	1065.5323	533.2698	1064.5483	532.7778	D	**589.2689**	295.1381	572.2423	286.6248	571.2583	286.1328	5
11	1210.6175	605.8124	1193.5909	597.2991	1192.6069	596.8071	Q	**474.2419**	237.6246	457.2154	229.1113			4
12	1267.6389	634.3231	1250.6124	625.8098	1249.6284	625.3178	G	**346.1833**	173.5953	329.1568	165.0820			3
13	1381.6819	691.3446	1364.6553	682.8313	1363.6713	682.3393	N	289.1619	145.0846	272.1353	136.5713			2
14							R	**175.1190**	88.0631	158.0924	79.5498			1

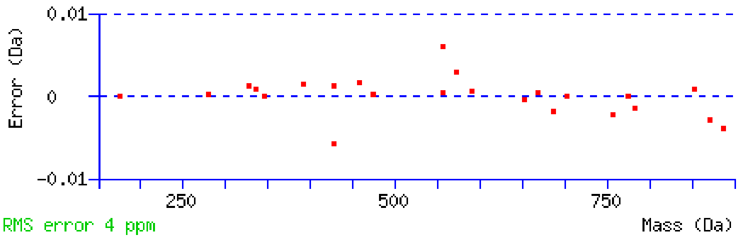

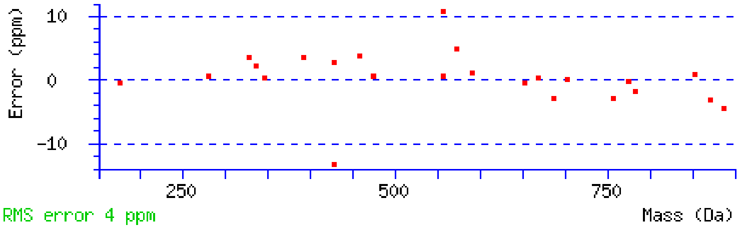

NCBI **BLAST** search of NGRVEIIANDQGNR
(Parameters: blastp, nr protein database, expect=20000, no filter, PAM30)
Other BLAST web gateways

All matches to this query

Score	Mr(calc)	Delta	Sequence
46.3	1554.7862	0.0003	NGRVEIIANDQGNR

Mascot: http://www.matrixscience.com/

Mascot Search Results

Peptide View

MS/MS Fragmentation of **NGRVEIIANDQGNR**
Found in **P11021** in **uni_human_nr**, GRP78_HUMAN 78 kDa glucose-regulated protein OS=Homo sapiens GN=HSPA5 PE=1 SV=2

Match to Query 4299: 1554.785748 from(778.400150,2+) intensity(742938.5625) rtinseconds(958) scans(4000) index(2269)
Title: 130806_HPL_Human_Plaque_BR1_TR2_04_Spectrum025683_scans__4000_RTINSECONDS=958
Data file E:\\RAW\\HumanPlaques\\mgf\\T\\1D\\T130806_HPL_Human_Plaque_BR1_TR2_04.mgf

Click mouse within plot area to zoom in by factor of two about that point
Or, Plot from 0 to 1600 Da Full range
Label all possible matches ○ Label matches used for scoring ⊙
Show Y-axis ☐

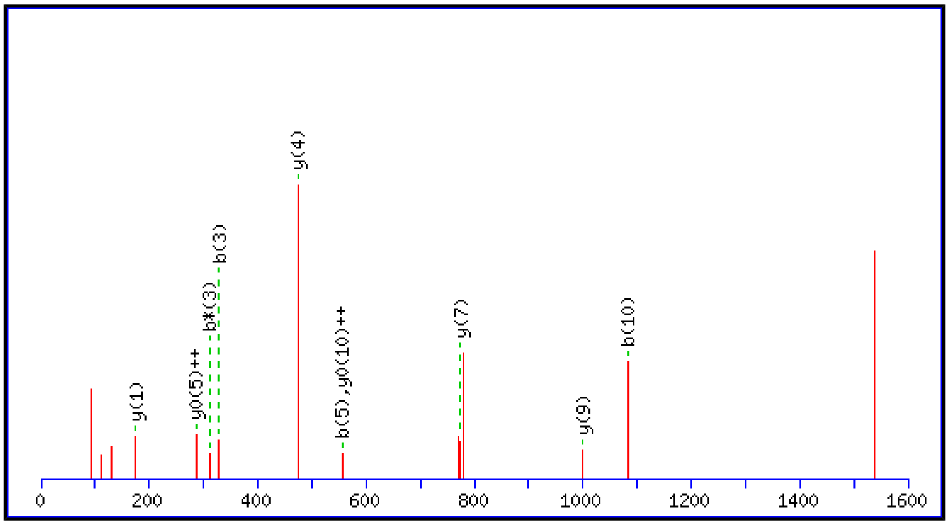

Monoisotopic mass of neutral peptide Mr(calc): 1554.7862
Fixed modifications: Carbamidomethyl (C) (apply to specified residues or termini only)
Ions Score: 30 **Expect:** 0.079
Matches : 10/140 fragment ions using 14 most intense peaks (help)

#	b	b++	b*	b*++	b0	b0++	Seq.	y	y++	y*	y*++	y0	y0++	#
1	115.0502	58.0287	98.0237	49.5155			N							14
2	172.0717	86.5395	155.0451	78.0262			G	1441.7506	721.3789	1424.7241	712.8657	1423.7401	712.3737	13
3	***328.1728***	164.5900	311.1462	156.0768			R	1384.7292	692.8682	1367.7026	684.3549	1366.7186	683.8629	12
4	427.2412	214.1242	410.2146	205.6110			V	1228.6280	614.8177	1211.6015	606.3044	1210.6175	605.8124	11
5	***556.2838***	278.6455	539.2572	270.1323	538.2732	269.6402	E	1129.5596	565.2835	1112.5331	556.7702	1111.5491	556.2782	10
6	669.3678	335.1876	652.3413	326.6743	651.3573	326.1823	I	***1000.5170***	500.7622	983.4905	492.2489	982.5065	491.7569	9
7	782.4519	391.7296	765.4254	383.2163	764.4413	382.7243	I	887.4330	444.2201	870.4064	435.7068	869.4224	435.2148	8
8	853.4890	427.2482	836.4625	418.7349	835.4785	418.2429	A	***774.3489***	387.6781	757.3224	379.1648	756.3383	378.6728	7
9	967.5320	484.2696	950.5054	475.7563	949.5214	475.2643	N	703.3118	352.1595	686.2852	343.6463	685.3012	343.1543	6
10	***1082.5589***	541.7831	1065.5323	533.2698	1064.5483	532.7778	D	589.2689	295.1381	572.2423	286.6248	571.2583	286.1328	5
11	1210.6175	605.8124	1193.5909	597.2991	1192.6069	596.8071	Q	***474.2419***	237.6246	457.2154	229.1113			4
12	1267.6389	634.3231	1250.6124	625.8098	1249.6284	625.3178	G	346.1833	173.5953	329.1568	165.0820			3
13	1381.6819	691.3446	1364.6553	682.8313	1363.6713	682.3393	N	289.1619	145.0846	272.1353	136.5713			2
14							R	***175.1190***	88.0631	158.0924	79.5498			1

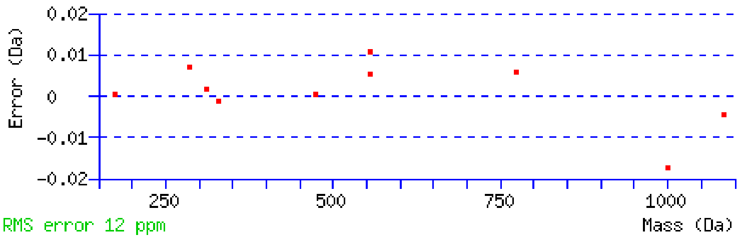

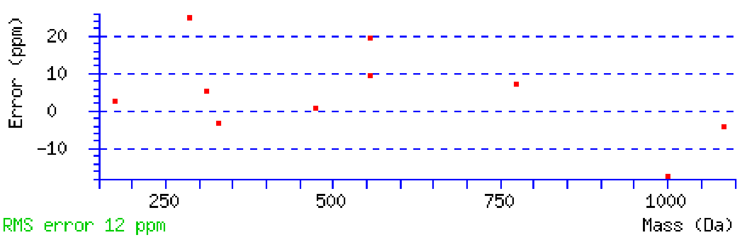

NCBI **BLAST** search of NGRVEIIANDQGNR
(Parameters: blastp, nr protein database, expect=20000, no filter, PAM30)
Other BLAST web gateways

All matches to this query

Score	Mr(calc)	Delta	Sequence
29.5	1554.7862	-0.0005	NGRVEIIANDQGNR
5.7	1554.7824	0.0034	KPAATSPLSPMANGGR
3.9	1554.7864	-0.0007	CLVTQVAYFLANR
3.9	1554.7864	-0.0007	CLVTQVAYFLANR
3.9	1554.7871	-0.0014	LCLSRPCPPSRGR
1.0	1553.7872	0.9986	VGNMDTFTKIQRK
0.9	1554.7936	-0.0079	RENRHTIEMIEK
0.3	1553.7838	1.0019	DVTIFSVGVYNANR
0.3	1553.7871	0.9986	VPMAAAVPKAENPSR

Mascot: http://www.matrixscience.com/

Mascot Search Results

Peptide View

MS/MS Fragmentation of **ATAESASECLPCDCNGR**
Found in **P11047** in **uni_human_nr**, LAMC1_HUMAN Laminin subunit gamma-1 OS=Homo sapiens GN=LAMC1 PE=1 SV=3

Match to Query 7979: 1897.723128 from(949.868840,2+) intensity(587877.8125) rtinseconds(1353) scans(6444) index(4314)
Title: 130806_HPL_Human_Plaque_BR1_TR2_18_Spectrum024462_scans__6444_RTINSECONDS=1353
Data file E:\\RAW\\HumanPlaques\\mgf\\T\\1D\\T130806_HPL_Human_Plaque_BR1_TR2_18.mgf

Click mouse within plot area to zoom in by factor of two about that point
Or, Plot from 100 to 1400 Da Full range
Label all possible matches ○ Label matches used for scoring ⊙
Show Y-axis ☐

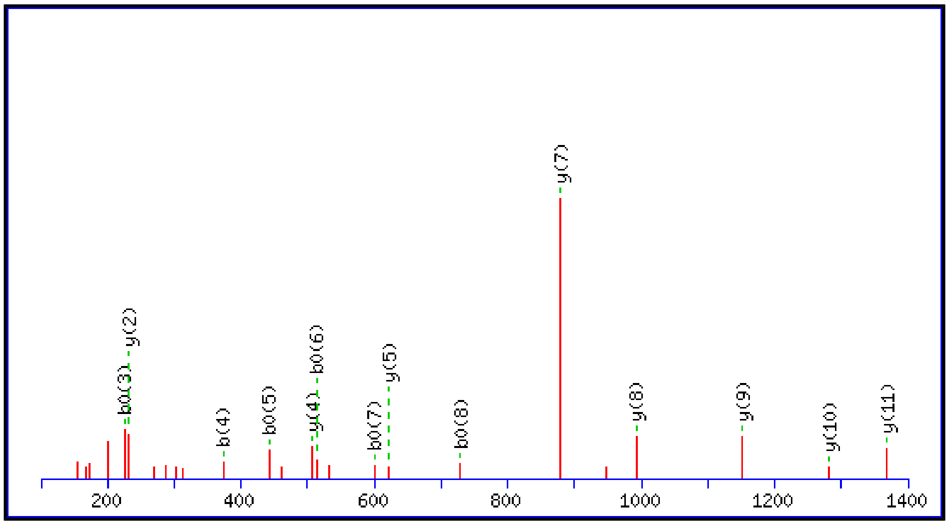

Monoisotopic mass of neutral peptide Mr(calc): 1897.7240
Fixed modifications: Carbamidomethyl (C) (apply to specified residues or termini only)
Variable modifications:
N15 : Deamidated (NQ)
Ions Score: 67 **Expect:** 3.7e-007
Matches : 14/154 fragment ions using 17 most intense peaks (help)

#	b	b^{++}	b^{*}	b^{*++}	b^{0}	b^{0++}	Seq.	y	y^{++}	y^{*}	y^{*++}	y^{0}	y^{0++}	#
1	72.0444	36.5258					A							17
2	173.0921	87.0497			155.0815	78.0444	T	1827.6942	914.3508	1810.6677	905.8375	1809.6837	905.3455	16
3	244.1292	122.5682			226.1186	113.5629	A	1726.6465	863.8269	1709.6200	855.3136	1708.6360	854.8216	15
4	**373.1718**	187.0895			355.1612	178.0842	E	1655.6094	828.3084	1638.5829	819.7951	1637.5989	819.3031	14
5	460.2038	230.6055			442.1932	221.6003	S	1526.5668	763.7871	1509.5403	755.2738	1508.5563	754.7818	13
6	531.2409	266.1241			513.2304	257.1188	A	1439.5348	720.2710	1422.5083	711.7578	1421.5242	711.2658	12
7	618.2729	309.6401			600.2624	300.6348	S	***1368.4977***	684.7525	1351.4711	676.2392	1350.4871	675.7472	11
8	747.3155	374.1614			729.3050	365.1561	E	***1281.4657***	641.2365	1264.4391	632.7232	1263.4551	632.2312	10
9	907.3462	454.1767			889.3356	445.1714	C	***1152.4231***	576.7152	1135.3965	568.2019	1134.4125	567.7099	9
10	1020.4303	510.7188			1002.4197	501.7135	L	***992.3924***	496.6999	975.3659	488.1866	974.3819	487.6946	8
11	1117.4830	559.2451			1099.4725	550.2399	P	***879.3084***	440.1578	862.2818	431.6445	861.2978	431.1525	7
12	1277.5137	639.2605			1259.5031	630.2552	C	782.2556	391.6314	765.2291	383.1182	764.2450	382.6262	6
13	1392.5406	696.7739			1374.5300	687.7687	D	***622.2250***	311.6161	605.1984	303.1028	604.2144	302.6108	5
14	1552.5713	776.7893			1534.5607	767.7840	C	***507.1980***	254.1026	490.1715	245.5894			4
15	1667.5982	834.3027	1650.5717	825.7895	1649.5876	825.2975	N	347.1674	174.0873	330.1408	165.5740			3

**16**	1724.6197	862.8135	1707.5931	854.3002	1706.6091	853.8082	**G**	*232.1404*	116.5738	215.1139	108.0606			**2**
**17**							**R**	175.1190	88.0631	158.0924	79.5498			**1**

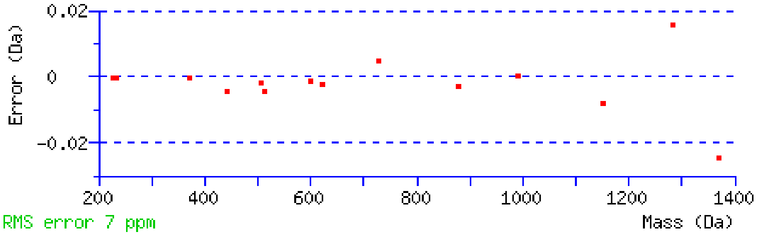

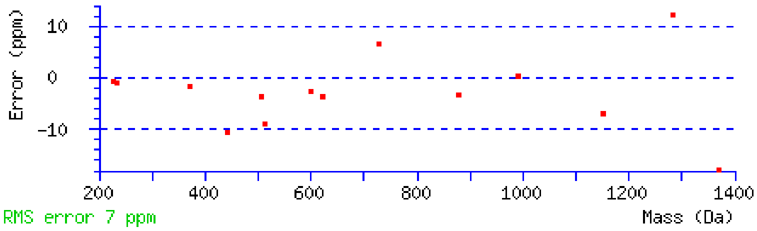

NCBI **BLAST** search of ATAESASECLPCDCNGR
(Parameters: blastp, nr protein database, expect=20000, no filter, PAM30)
Other BLAST web gateways

All matches to this query

Score	Mr(calc)	Delta	Sequence
67.1	1897.7240	-0.0009	ATAESASECLPCDCNGR

Mascot: http://www.matrixscience.com/

Mascot Search Results

Peptide View

MS/MS Fragmentation of **ATAESASECLPCDCNGR**
Found in **P11047** in **uni_human_nr**, LAMC1_HUMAN Laminin subunit gamma-1 OS=Homo sapiens GN=LAMC1 PE=1 SV=3

Match to Query 6339: 1896.738248 from(949.376400,2+) intensity(4645359.5000) rtinseconds(1299) scans(5791) index(3571)
Title: 130806_HPL_Human_Plaque_BR1_TR2_15_Spectrum023902_scans__5791_RTINSECONDS=1299
Data file E:\\RAW\\HumanPlaques\\mgf\\T\\1D\\T130806_HPL_Human_Plaque_BR1_TR2_15.mgf

Click mouse within plot area to zoom in by factor of two about that point
Or, Plot from 0 to 1900 Da Full range
Label all possible matches ○ Label matches used for scoring ⊙
Show Y-axis ☐

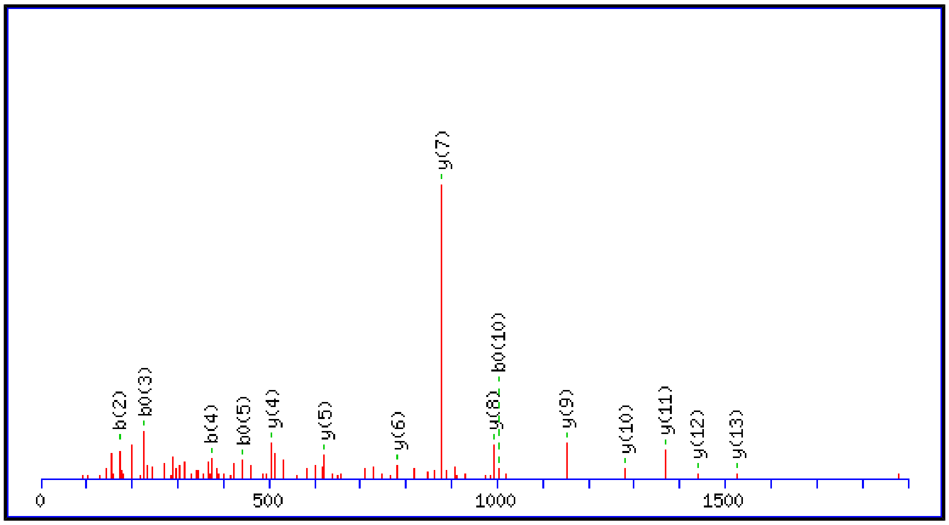

Monoisotopic mass of neutral peptide Mr(calc): 1896.7400
Fixed modifications: Carbamidomethyl (C) (apply to specified residues or termini only)
Ions Score: 105 **Expect:** 1.1e-010
Matches : 15/154 fragment ions using 16 most intense peaks (help)

#	b	b^{++}	b*	b^{*++}	b^{0}	b^{0++}	Seq.	y	y^{++}	y*	y^{*++}	y^{0}	y^{0++}	#
1	72.0444	36.5258					A							17
2	**173.0921**	87.0497			155.0815	78.0444	T	1826.7102	913.8587	1809.6837	905.3455	1808.6996	904.8535	16
3	244.1292	122.5682			226.1186	113.5629	A	1725.6625	863.3349	1708.6360	854.8216	1707.6520	854.3296	15
4	**373.1718**	187.0895			355.1612	178.0842	E	1654.6254	827.8163	1637.5989	819.3031	1636.6149	818.8111	14
5	460.2038	230.6055			442.1932	221.6003	S	***1525.5828***	763.2950	1508.5563	754.7818	1507.5723	754.2898	13
6	531.2409	266.1241			513.2304	257.1188	A	***1438.5508***	719.7790	1421.5242	711.2658	1420.5402	710.7738	12
7	618.2729	309.6401			600.2624	300.6348	S	***1367.5137***	684.2605	1350.4871	675.7472	1349.5031	675.2552	11
8	747.3155	374.1614			729.3050	365.1561	E	***1280.4817***	640.7445	1263.4551	632.2312	1262.4711	631.7392	10
9	907.3462	454.1767			889.3356	445.1714	C	***1151.4391***	576.2232	1134.4125	567.7099	1133.4285	567.2179	9
10	1020.4303	510.7188			1002.4197	501.7135	L	***991.4084***	496.2078	974.3819	487.6946	973.3978	487.2026	8
11	1117.4830	559.2451			1099.4725	550.2399	P	***878.3243***	439.6658	861.2978	431.1525	860.3138	430.6605	7
12	1277.5137	639.2605			1259.5031	630.2552	C	***781.2716***	391.1394	764.2450	382.6262	763.2610	382.1341	6
13	1392.5406	696.7739			1374.5300	687.7687	D	***621.2409***	311.1241	604.2144	302.6108	603.2304	302.1188	5
14	1552.5713	776.7893			1534.5607	767.7840	C	***506.2140***	253.6106	489.1874	245.0974			4
15	1666.6142	833.8107	1649.5876	825.2975	1648.6036	824.8054	N	346.1833	173.5953	329.1568	165.0820			3
16	1723.6356	862.3215	1706.6091	853.8082	1705.6251	853.3162	G	232.1404	116.5738	215.1139	108.0606			2

**17**							**R**	175.1190	88.0631	158.0924	79.5498			**1**

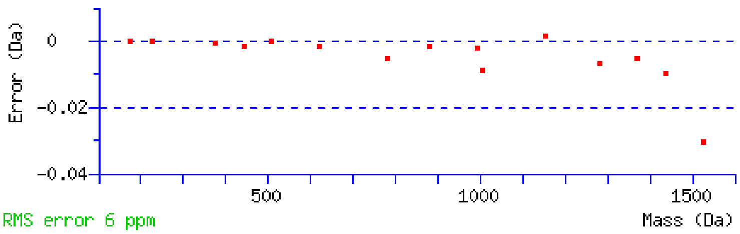

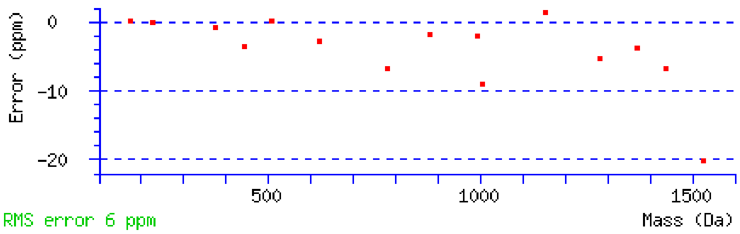

NCBI **BLAST** search of ATAESASECLPCDCNGR
(Parameters: blastp, nr protein database, expect=20000, no filter, PAM30)
Other BLAST web gateways

All matches to this query

Score	Mr(calc)	Delta	Sequence
105.3	1896.7400	-0.0018	ATAESASECLPCDCNGR

Mascot: http://www.matrixscience.com/

Mascot Search Results

Peptide View

MS/MS Fragmentation of **ATAESASECLPCDCNGR**
Found in **P11047** in **uni_human_nr**, LAMC1_HUMAN Laminin subunit gamma-1 OS=Homo sapiens GN=LAMC1 PE=1 SV=3

Match to Query 6251: 1896.739332 from(633.253720,3+) intensity(1366721.5000) rtinseconds(1290) scans(5802) index(3614)
Title: 130806_HPL_Human_Plaque_BR1_TR2_14_Spectrum024365_scans__5802_RTINSECONDS=1290
Data file E:\\RAW\\HumanPlaques\\mgf\\T\\1D\\T130806_HPL_Human_Plaque_BR1_TR2_14.mgf

Click mouse within plot area to zoom in by factor of two about that point
Or, [Plot from] 50 to 1000 Da [Full range]
Label all possible matches ○ Label matches used for scoring ⦿
Show Y-axis ☐

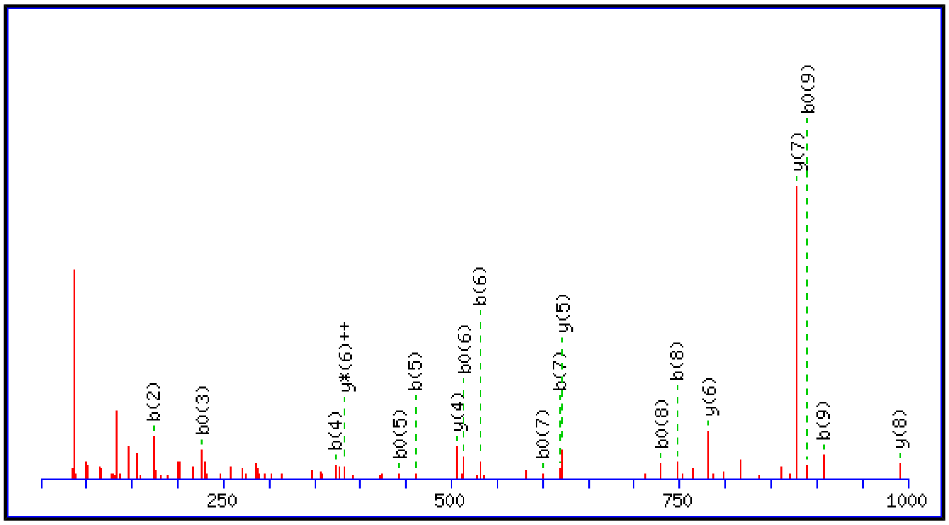

Monoisotopic mass of neutral peptide Mr(calc): 1896.7400
Fixed modifications: Carbamidomethyl (C) (apply to specified residues or termini only)
Ions Score: 58 **Expect:** 6.8e-006
Matches : 19/154 fragment ions using 28 most intense peaks (help)

#	b	b^{++}	b*	b^{*++}	b^0	b^{0++}	Seq.	y	y^{++}	y*	y^{*++}	y^0	y^{0++}	#
1	72.0444	36.5258					A							17
2	***173.0921***	87.0497			155.0815	78.0444	T	1826.7102	913.8587	1809.6837	905.3455	1808.6996	904.8535	16
3	244.1292	122.5682			226.1186	113.5629	A	1725.6625	863.3349	1708.6360	854.8216	1707.6520	854.3296	15
4	***373.1718***	187.0895			355.1612	178.0842	E	1654.6254	827.8163	1637.5989	819.3031	1636.6149	818.8111	14
5	***460.2038***	230.6055			442.1932	221.6003	S	1525.5828	763.2950	1508.5563	754.7818	1507.5723	754.2898	13
6	***531.2409***	266.1241			513.2304	257.1188	A	1438.5508	719.7790	1421.5242	711.2658	1420.5402	710.7738	12
7	***618.2729***	309.6401			600.2624	300.6348	S	1367.5137	684.2605	1350.4871	675.7472	1349.5031	675.2552	11
8	***747.3155***	374.1614			729.3050	365.1561	E	1280.4817	640.7445	1263.4551	632.2312	1262.4711	631.7392	10
9	***907.3462***	454.1767			889.3356	445.1714	C	1151.4391	576.2232	1134.4125	567.7099	1133.4285	567.2179	9
10	1020.4303	510.7188			1002.4197	501.7135	L	***991.4084***	496.2078	974.3819	487.6946	973.3978	487.2026	8
11	1117.4830	559.2451			1099.4725	550.2399	P	***878.3243***	439.6658	861.2978	431.1525	860.3138	430.6605	7
12	1277.5137	639.2605			1259.5031	630.2552	C	***781.2716***	391.1394	764.2450	382.6262	763.2610	382.1341	6
13	1392.5406	696.7739			1374.5300	687.7687	D	***621.2409***	311.1241	604.2144	302.6108	603.2304	302.1188	5
14	1552.5713	776.7893			1534.5607	767.7840	C	***506.2140***	253.6106	489.1874	245.0974			4
15	1666.6142	833.8107	1649.5876	825.2975	1648.6036	824.8054	N	346.1833	173.5953	329.1568	165.0820			3
16	1723.6356	862.3215	1706.6091	853.8082	1705.6251	853.3162	G	232.1404	116.5738	215.1139	108.0606			2

17							R	175.1190	88.0631	158.0924	79.5498			1

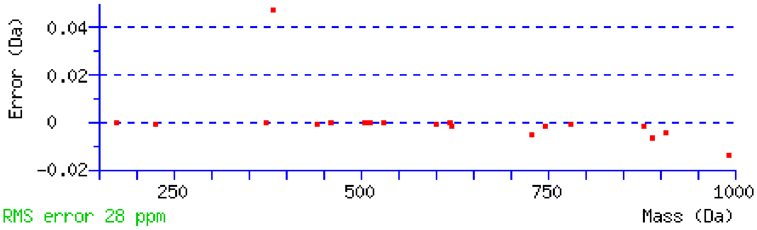

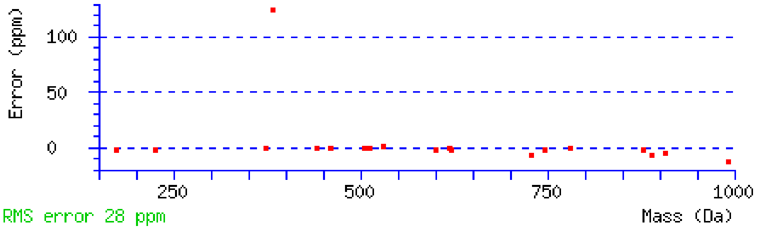

NCBI **BLAST** search of ATAESASECLPCDCNGR
(Parameters: blastp, nr protein database, expect=20000, no filter, PAM30)
Other BLAST web gateways

All matches to this query

Score	Mr(calc)	Delta	Sequence
57.9	1896.7400	-0.0007	ATAESASECLPCDCNGR

**Mascot:** http://www.matrixscience.com/

Mascot Search Results

Peptide View

MS/MS Fragmentation of **RATAESASECLPCDCNGR**
Found in **P11047** in **uni_human_nr**, LAMC1_HUMAN Laminin subunit gamma-1 OS=Homo sapiens GN=LAMC1 PE=1 SV=3

Match to Query 6963: 2053.824252 from(685.615360,3+) intensity(226995.8438) rtinseconds(1174) scans(4879) index(2661)
Title: 130801_HPL_Human_Plaque_BR1_TR1_14_Spectrum023162_scans__4879_RTINSECONDS=1174
Data file E:\\RAW\\HumanPlaques\\mgf\\T\\1D\\T130801_HPL_Human_Plaque_BR1_TR1_14.mgf

Click mouse within plot area to zoom in by factor of two about that point
Or, [Plot from] 50 to 1100 Da [Full range]
Label all possible matches ○ Label matches used for scoring ⊙
Show Y-axis ☐

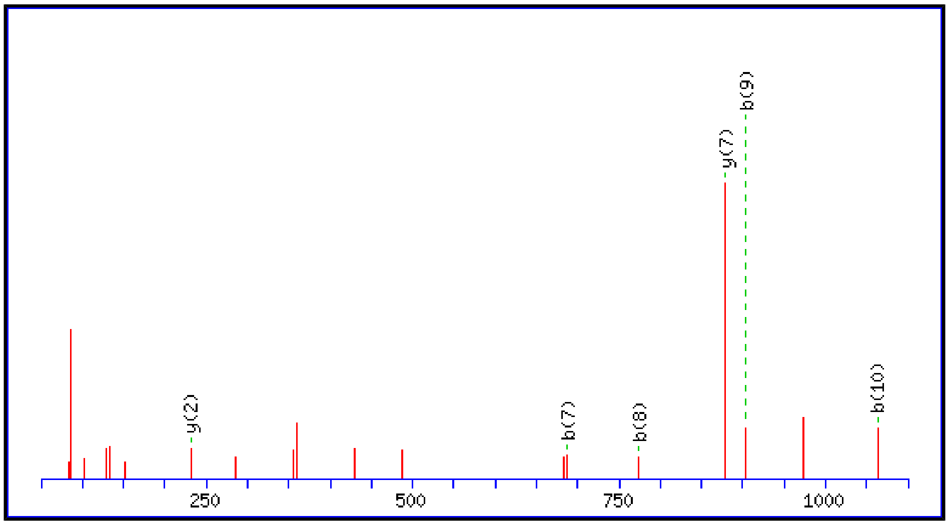

Monoisotopic mass of neutral peptide Mr(calc): 2053.8252
Fixed modifications: Carbamidomethyl (C) (apply to specified residues or termini only)
Variable modifications:
N16 : Deamidated (NQ)
Ions Score: 17 **Expect:** 0.15
Matches : 6/192 fragment ions using 15 most intense peaks (help)

#	b	b^{++}	b*	b^{*++}	b^{0}	b^{0++}	Seq.	y	y^{++}	y*	y^{*++}	y^{0}	y^{0++}	#
1	157.1084	79.0578	140.0818	70.5446			R							18
2	228.1455	114.5764	211.1190	106.0631			A	1898.7313	949.8693	1881.7048	941.3560	1880.7208	940.8640	17
3	329.1932	165.1002	312.1666	156.5870	311.1826	156.0949	T	1827.6942	914.3508	1810.6677	905.8375	1809.6837	905.3455	16
4	400.2303	200.6188	383.2037	192.1055	382.2197	191.6135	A	1726.6465	863.8269	1709.6200	855.3136	1708.6360	854.8216	15
5	529.2729	265.1401	512.2463	256.6268	511.2623	256.1348	E	1655.6094	828.3084	1638.5829	819.7951	1637.5989	819.3031	14
6	616.3049	308.6561	599.2784	300.1428	598.2944	299.6508	S	1526.5668	763.7871	1509.5403	755.2738	1508.5563	754.7818	13
7	***687.3420***	344.1747	670.3155	335.6614	669.3315	335.1694	A	1439.5348	720.2710	1422.5083	711.7578	1421.5242	711.2658	12
8	***774.3741***	387.6907	757.3475	379.1774	756.3635	378.6854	S	1368.4977	684.7525	1351.4711	676.2392	1350.4871	675.7472	11
9	***903.4167***	452.2120	886.3901	443.6987	885.4061	443.2067	E	1281.4657	641.2365	1264.4391	632.7232	1263.4551	632.2312	10
10	***1063.4473***	532.2273	1046.4208	523.7140	1045.4367	523.2220	C	1152.4231	576.7152	1135.3965	568.2019	1134.4125	567.7099	9
11	1176.5314	588.7693	1159.5048	580.2560	1158.5208	579.7640	L	992.3924	496.6999	975.3659	488.1866	974.3819	487.6946	8
12	1273.5841	637.2957	1256.5576	628.7824	1255.5736	628.2904	P	***879.3084***	440.1578	862.2818	431.6445	861.2978	431.1525	7
13	1433.6148	717.3110	1416.5882	708.7978	1415.6042	708.3057	C	782.2556	391.6314	765.2291	383.1182	764.2450	382.6262	6
14	1548.6417	774.8245	1531.6152	766.3112	1530.6312	765.8192	D	622.2250	311.6161	605.1984	303.1028	604.2144	302.6108	5
15	1708.6724	854.8398	1691.6458	846.3265	1690.6618	845.8345	C	507.1980	254.1026	490.1715	245.5894			4

**16**	1823.6993	912.3533	1806.6728	903.8400	1805.6887	903.3480	**N**	347.1674	174.0873	330.1408	165.5740			**3**
**17**	1880.7208	940.8640	1863.6942	932.3508	1862.7102	931.8587	**G**	***232.1404***	116.5738	215.1139	108.0606			**2**
**18**							**R**	175.1190	88.0631	158.0924	79.5498			**1**

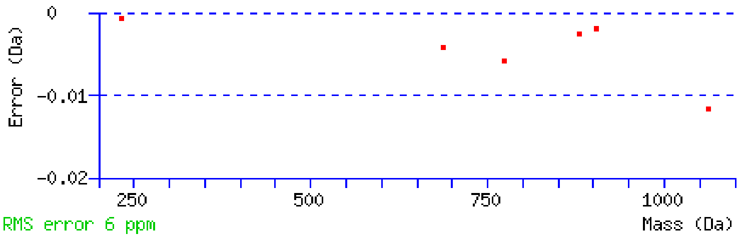

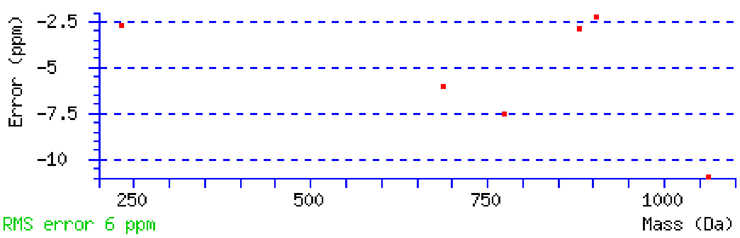

NCBI **BLAST** search of RATAESASECLPCDCNGR
(Parameters: blastp, nr protein database, expect=20000, no filter, PAM30)
Other BLAST web gateways

All matches to this query

Score	Mr(calc)	Delta	Sequence
16.8	2053.8252	-0.0009	RATAESASECLPCDCNGR

Mascot: http://www.matrixscience.com/

Mascot Search Results

Peptide View

MS/MS Fragmentation of **RATAESASECLPCDCNGR**
Found in **P11047** in **uni_human_nr**, LAMC1_HUMAN Laminin subunit gamma-1 OS=Homo sapiens GN=LAMC1 PE=1 SV=3

Match to Query 7946: 2052.841512 from(685.287780,3+) intensity(5531704.5000) rtinseconds(1094) scans(4579) index(2665)
Title: 130806_HPL_Human_Plaque_BR2_TR2_09_Spectrum023887_scans__4579_RTINSECONDS=1094
Data file E:\\RAW\\HumanPlaques\\mgf\\T\\1D\\T130806_HPL_Human_Plaque_BR2_TR2_09.mgf

Click mouse within plot area to zoom in by factor of two about that point
Or, Plot from 0 to 2000 Da Full range
Label all possible matches ○ Label matches used for scoring ⊙
Show Y-axis ☐

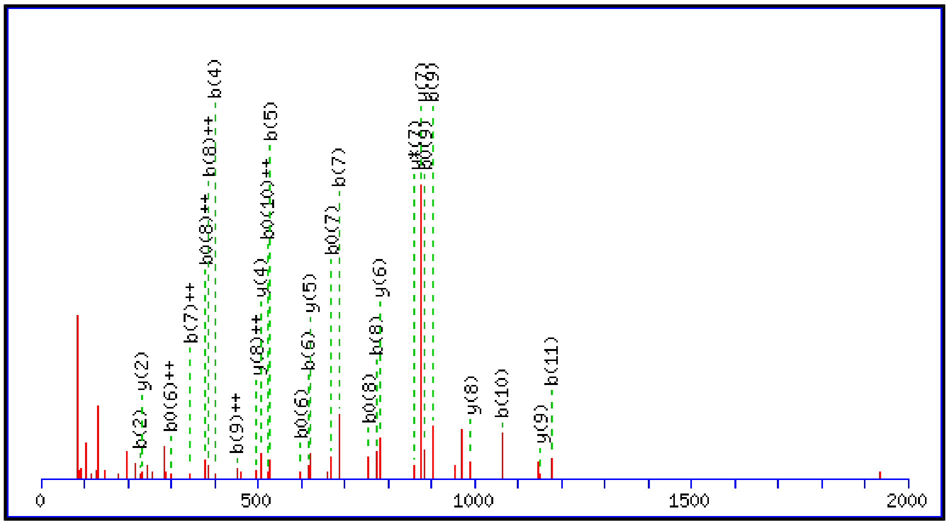

Monoisotopic mass of neutral peptide Mr(calc): 2052.8411
Fixed modifications: Carbamidomethyl (C) (apply to specified residues or termini only)
Ions Score: 58 **Expect:** 1.6e-005
Matches : 28/192 fragment ions using 52 most intense peaks (help)

#	b	b^{++}	b*	b^{*++}	b^0	b^{0++}	Seq.	y	y^{++}	y*	y^{*++}	y^0	y^{0++}	#
1	157.1084	79.0578	140.0818	70.5446			R							18
2	**228.1455**	114.5764	211.1190	106.0631			A	1897.7473	949.3773	1880.7208	940.8640	1879.7368	940.3720	17
3	329.1932	165.1002	312.1666	156.5870	311.1826	156.0949	T	1826.7102	913.8587	1809.6837	905.3455	1808.6996	904.8535	16
4	**400.2303**	200.6188	383.2037	192.1055	382.2197	191.6135	A	1725.6625	863.3349	1708.6360	854.8216	1707.6520	854.3296	15
5	**529.2729**	265.1401	512.2463	256.6268	511.2623	256.1348	E	1654.6254	827.8163	1637.5989	819.3031	1636.6149	818.8111	14
6	**616.3049**	308.6561	599.2784	300.1428	598.2944	299.6508	S	1525.5828	763.2950	1508.5563	754.7818	1507.5723	754.2898	13
7	**687.3420**	**344.1747**	670.3155	335.6614	669.3315	335.1694	A	1438.5508	719.7790	1421.5242	711.2658	1420.5402	710.7738	12
8	**774.3741**	**387.6907**	757.3475	379.1774	756.3635	378.6854	S	1367.5137	684.2605	1350.4871	675.7472	1349.5031	675.2552	11
9	**903.4167**	**452.2120**	886.3901	443.6987	885.4061	443.2067	E	1280.4817	640.7445	1263.4551	632.2312	1262.4711	631.7392	10
10	**1063.4473**	532.2273	1046.4208	523.7140	1045.4367	523.2220	C	**1151.4391**	576.2232	1134.4125	567.7099	1133.4285	567.2179	9
11	**1176.5314**	588.7693	1159.5048	580.2560	1158.5208	579.7640	L	**991.4084**	496.2078	974.3819	487.6946	973.3978	487.2026	8
12	1273.5841	637.2957	1256.5576	628.7824	1255.5736	628.2904	P	**878.3243**	439.6658	861.2978	431.1525	860.3138	430.6605	7
13	1433.6148	717.3110	1416.5882	708.7978	1415.6042	708.3057	C	**781.2716**	391.1394	764.2450	382.6262	763.2610	382.1341	6
14	1548.6417	774.8245	1531.6152	766.3112	1530.6312	765.8192	D	**621.2409**	311.1241	604.2144	302.6108	603.2304	302.1188	5
15	1708.6724	854.8398	1691.6458	846.3265	1690.6618	845.8345	C	**506.2140**	253.6106	489.1874	245.0974			4
16	1822.7153	911.8613	1805.6887	903.3480	1804.7047	902.8560	N	346.1833	173.5953	329.1568	165.0820			3

**17**	1879.7368	940.3720	1862.7102	931.8587	1861.7262	931.3667	**G**	*232.1404*	116.5738	215.1139	108.0606			**2**
**18**							**R**	175.1190	88.0631	158.0924	79.5498			**1**

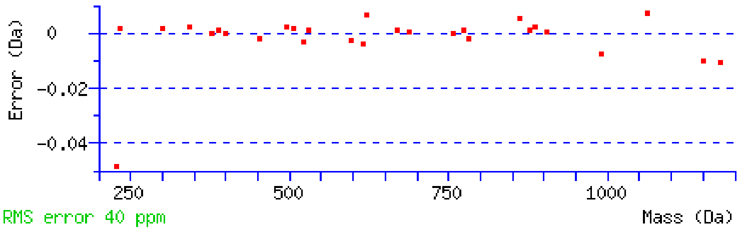

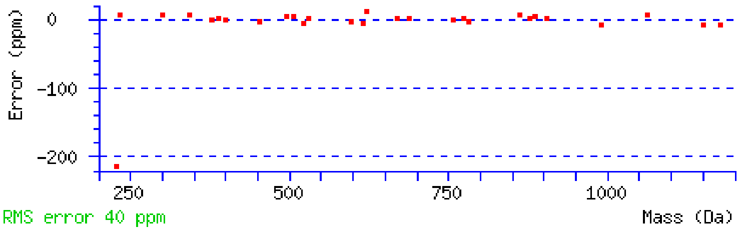

NCBI **BLAST** search of RATAESASECLPCDCNGR
(Parameters: blastp, nr protein database, expect=20000, no filter, PAM30)
Other BLAST web gateways

All matches to this query

Score	Mr(calc)	Delta	Sequence
57.7	2052.8411	0.0004	RATAESASECLPCDCNGR

**Mascot:** http://www.matrixscience.com/

Mascot Search Results

Peptide View

MS/MS Fragmentation of **LSVGSVYRPNQNGR**
Found in **Q08397** in **uni_human_nr**, LOXL1_HUMAN Lysyl oxidase homolog 1 OS=Homo sapiens GN=LOXL1 PE=1 SV=2

Match to Query 4440: 1546.784862 from(516.602230,3+) intensity(2036954.6250) rtinseconds(1313) scans(6372) index(4169)
Title: 130801_HPL_Human_Plaque_BR1_TR1_03_Spectrum027419_scans__6372_RTINSECONDS=1313
Data file E:\\RAW\\HumanPlaques\\mgf\\T\\1D\\T130801_HPL_Human_Plaque_BR1_TR1_03.mgf

Click mouse within plot area to zoom in by factor of two about that point
Or, Plot from 0 to 1500 Da Full range
Label all possible matches ○ Label matches used for scoring ⊙
Show Y-axis ☐

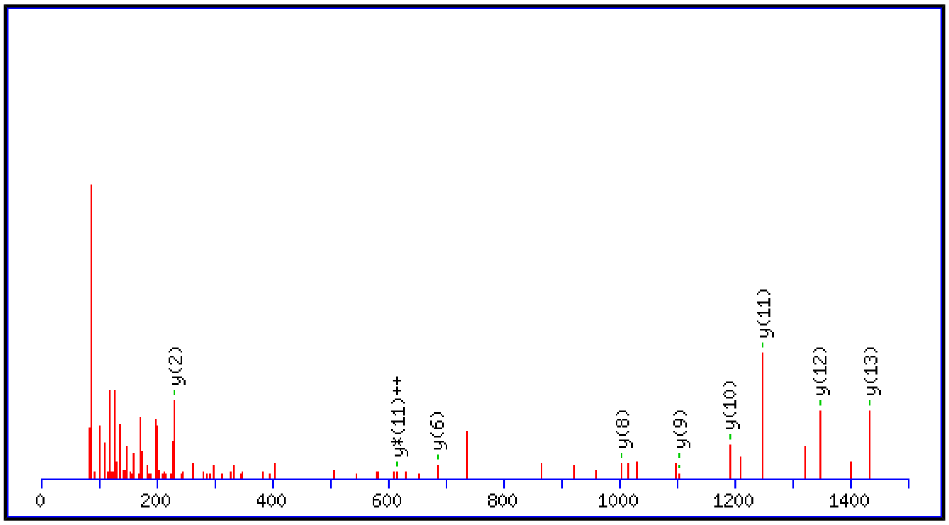

Monoisotopic mass of neutral peptide Mr(calc): 1546.7852
Fixed modifications: Carbamidomethyl (C) (apply to specified residues or termini only)
Variable modifications:
N12 : Deamidated (NQ)
Ions Score: 35 **Expect:** 0.029
Matches : 9/122 fragment ions using 27 most intense peaks (help)

#	b	b^{++}	b*	b^{*++}	b^0	b^{0++}	Seq.	y	y^{++}	y*	y^{*++}	y^0	y^{0++}	#
1	114.0913	57.5493					L							14
2	201.1234	101.0653			183.1128	92.0600	S	***1434.7084***	717.8578	1417.6819	709.3446	1416.6979	708.8526	13
3	300.1918	150.5995			282.1812	141.5942	V	***1347.6764***	674.3418	1330.6498	665.8286	1329.6658	665.3365	12
4	357.2132	179.1103			339.2027	170.1050	G	***1248.6080***	624.8076	1231.5814	616.2944	1230.5974	615.8023	11
5	444.2453	222.6263			426.2347	213.6210	S	***1191.5865***	596.2969	1174.5600	587.7836	1173.5759	587.2916	10
6	543.3137	272.1605			525.3031	263.1552	V	***1104.5545***	552.7809	1087.5279	544.2676			9
7	706.3770	353.6921			688.3665	344.6869	Y	***1005.4861***	503.2467	988.4595	494.7334			8
8	862.4781	431.7427	845.4516	423.2294	844.4676	422.7374	R	842.4227	421.7150	825.3962	413.2017			7
9	959.5309	480.2691	942.5043	471.7558	941.5203	471.2638	P	***686.3216***	343.6645	669.2951	335.1512			6
10	1073.5738	537.2905	1056.5473	528.7773	1055.5633	528.2853	N	589.2689	295.1381	572.2423	286.6248			5
11	1201.6324	601.3198	1184.6058	592.8066	1183.6218	592.3146	Q	475.2259	238.1166	458.1994	229.6033			4
12	1316.6593	658.8333	1299.6328	650.3200	1298.6488	649.8280	N	347.1674	174.0873	330.1408	165.5740			3
13	1373.6808	687.3440	1356.6543	678.8308	1355.6702	678.3388	G	***232.1404***	116.5738	215.1139	108.0606			2
14							R	175.1190	88.0631	158.0924	79.5498			1

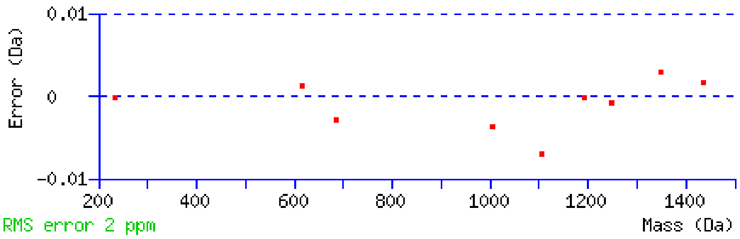

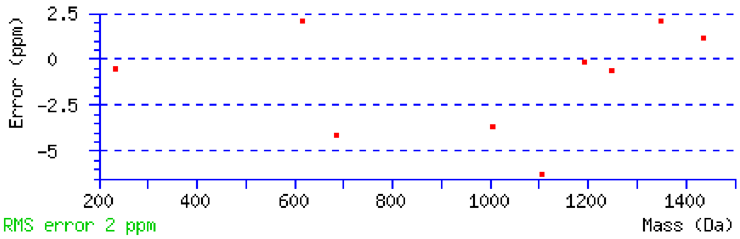

NCBI **BLAST** search of LSVGSVYRPNQNGR
(Parameters: blastp, nr protein database, expect=20000, no filter, PAM30)
Other BLAST web gateways

All matches to this query

Score	Mr(calc)	Delta	Sequence	Site Analysis
34.5	1546.7852	-0.0003	LSVGSVYRPNQNGR	Deamidated N12 33.33%
34.5	1546.7852	-0.0003	LSVGSVYRPNQNGR	Deamidated Q11 33.33%
34.5	1546.7852	-0.0003	LSVGSVYRPNQNGR	Deamidated N10 33.33%

Mascot: http://www.matrixscience.com/

Mascot Search Results

Peptide View

MS/MS Fragmentation of **LSVGSVYRPNQNGRGLPDLVPDPNYVQASTYVQR**
Found in **Q08397** in **uni_human_nr**, LOXL1_HUMAN Lysyl oxidase homolog 1 OS=Homo sapiens GN=LOXL1 PE=1 SV=2

Match to Query 16302: 3762.876416 from(941.726380,4+) intensity(351759.3125) rtinseconds(2845) scans(16494) index(12665)
Title: 130801_HPL_Human_Plaque_BR2_TR1_09_Spectrum033477_scans__16494_RTINSECONDS=2845
Data file E:\\RAW\\HumanPlaques\\mgf\\T\\1D\\T130801_HPL_Human_Plaque_BR2_TR1_09.mgf

Click mouse within plot area to zoom in by factor of two about that point
Or, Plot from 0 to 3800 Da Full range
Label all possible matches ○ Label matches used for scoring ⊙
Show Y-axis ☐

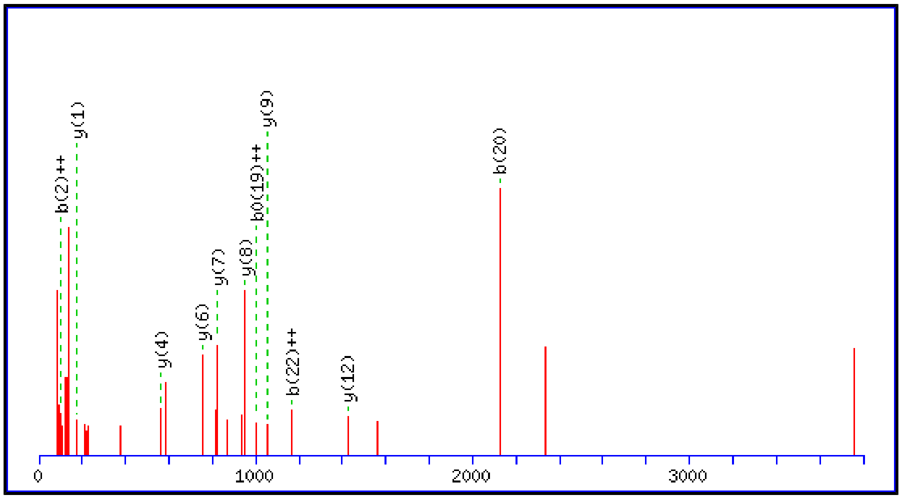

Monoisotopic mass of neutral peptide Mr(calc): 3761.8649
Fixed modifications: Carbamidomethyl (C) (apply to specified residues or termini only)
Variable modifications:
Q11 : Deamidated (NQ)
N12 : Deamidated (NQ)
N24 : Deamidated (NQ)
Ions Score: 23 **Expect:** 0.43
Matches : 11/372 fragment ions using 27 most intense peaks (help)

#	b	b++	b*	b*++	b0	b0++	Seq.	y	y++	y*	y*++	y0	y0++	#
1	114.0913	57.5493					L							34
2	201.1234	**101.0653**			183.1128	92.0600	S	3649.7881	1825.3977	3632.7616	1816.8844	3631.7776	1816.3924	33
3	300.1918	150.5995			282.1812	141.5942	V	3562.7561	1781.8817	3545.7296	1773.3684	3544.7455	1772.8764	32
4	357.2132	179.1103			339.2027	170.1050	G	3463.6877	1732.3475	3446.6611	1723.8342	3445.6771	1723.3422	31
5	444.2453	222.6263			426.2347	213.6210	S	3406.6662	1703.8368	3389.6397	1695.3235	3388.6557	1694.8315	30
6	543.3137	272.1605			525.3031	263.1552	V	3319.6342	1660.3207	3302.6076	1651.8075	3301.6236	1651.3155	29
7	706.3770	353.6921			688.3665	344.6869	Y	3220.5658	1610.7865	3203.5392	1602.2733	3202.5552	1601.7812	28
8	862.4781	431.7427	845.4516	423.2294	844.4676	422.7374	R	3057.5025	1529.2549	3040.4759	1520.7416	3039.4919	1520.2496	27
9	959.5309	480.2691	942.5043	471.7558	941.5203	471.2638	P	2901.4013	1451.2043	2884.3748	1442.6910	2883.3908	1442.1990	26
10	1073.5738	537.2905	1056.5473	528.7773	1055.5633	528.2853	N	2804.3486	1402.6779	2787.3220	1394.1647	2786.3380	1393.6726	25
11	1202.6164	601.8118	1185.5899	593.2986	1184.6058	592.8066	Q	2690.3057	1345.6565	2673.2791	1337.1432	2672.2951	1336.6512	24
12	1317.6434	659.3253	1300.6168	650.8120	1299.6328	650.3200	N	2561.2631	1281.1352	2544.2365	1272.6219	2543.2525	1272.1299	23
13	1374.6648	687.8360	1357.6383	679.3228	1356.6543	678.8308	G	2446.2361	1223.6217	2429.2096	1215.1084	2428.2256	1214.6164	22
14	1530.7659	765.8866	1513.7394	757.3733	1512.7554	756.8813	R	2389.2147	1195.1110	2372.1881	1186.5977	2371.2041	1186.1057	21
15	1587.7874	794.3973	1570.7608	785.8841	1569.7768	785.3921	G	2233.1135	1117.0604	2216.0870	1108.5471	2215.1030	1108.0551	20
16	1700.8715	850.9394	1683.8449	842.4261	1682.8609	841.9341	L	2176.0921	1088.5497	2159.0655	1080.0364	2158.0815	1079.5444	19
17	1797.9242	899.4657	1780.8977	890.9525	1779.9137	890.4605	P	2063.0080	1032.0076	2045.9815	1023.4944	2044.9974	1023.0024	18

**18**	1912.9512	956.9792	1895.9246	948.4659	1894.9406	947.9739	**D**	1965.9553	983.4813	1948.9287	974.9680	1947.9447	974.4760	**17**
**19**	2026.0352	1013.5213	2009.0087	1005.0080	2008.0247	1004.5160	**L**	1850.9283	925.9678	1833.9018	917.4545	1832.9177	916.9625	**16**
**20**	**2125.1036**	1063.0555	2108.0771	1054.5422	2107.0931	1054.0502	**V**	1737.8442	869.4258	1720.8177	860.9125	1719.8337	860.4205	**15**
**21**	2222.1564	1111.5818	2205.1299	1103.0686	2204.1458	1102.5766	**P**	1638.7758	819.8916	1621.7493	811.3783	1620.7653	810.8863	**14**
**22**	2337.1834	**1169.0953**	2320.1568	1160.5820	2319.1728	1160.0900	**D**	1541.7231	771.3652	1524.6965	762.8519	1523.7125	762.3599	**13**
**23**	2434.2361	1217.6217	2417.2096	1209.1084	2416.2255	1208.6164	**P**	***1426.6961***	713.8517	1409.6696	705.3384	1408.6856	704.8464	**12**
**24**	2549.2631	1275.1352	2532.2365	1266.6219	2531.2525	1266.1299	**N**	1329.6434	665.3253	1312.6168	656.8120	1311.6328	656.3200	**11**
**25**	2712.3264	1356.6668	2695.2998	1348.1536	2694.3158	1347.6615	**Y**	1214.6164	607.8118	1197.5899	599.2986	1196.6059	598.8066	**10**
**26**	2811.3948	1406.2010	2794.3683	1397.6878	2793.3842	1397.1958	**V**	***1051.5531***	526.2802	1034.5265	517.7669	1033.5425	517.2749	**9**
**27**	2939.4534	1470.2303	2922.4268	1461.7171	2921.4428	1461.2250	**Q**	***952.4847***	476.7460	935.4581	468.2327	934.4741	467.7407	**8**
**28**	3010.4905	1505.7489	2993.4639	1497.2356	2992.4799	1496.7436	**A**	***824.4261***	412.7167	807.3995	404.2034	806.4155	403.7114	**7**
**29**	3097.5225	1549.2649	3080.4960	1540.7516	3079.5120	1540.2596	**S**	***753.3890***	377.1981	736.3624	368.6849	735.3784	368.1928	**6**
**30**	3198.5702	1599.7887	3181.5437	1591.2755	3180.5596	1590.7835	**T**	666.3570	333.6821	649.3304	325.1688	648.3464	324.6768	**5**
**31**	3361.6335	1681.3204	3344.6070	1672.8071	3343.6230	1672.3151	**Y**	***565.3093***	283.1583	548.2827	274.6450			**4**
**32**	3460.7019	1730.8546	3443.6754	1722.3413	3442.6914	1721.8493	**V**	402.2459	201.6266	385.2194	193.1133			**3**
**33**	3588.7605	1794.8839	3571.7340	1786.3706	3570.7500	1785.8786	**Q**	303.1775	152.0924	286.1510	143.5791			**2**
**34**							**R**	***175.1190***	88.0631	158.0924	79.5498			**1**

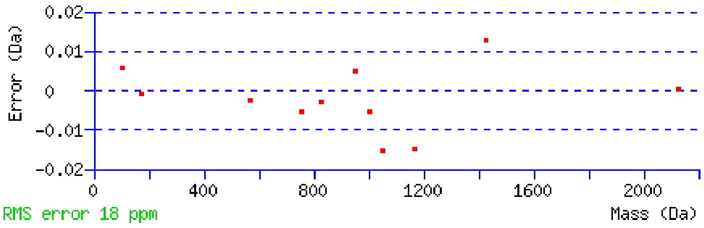

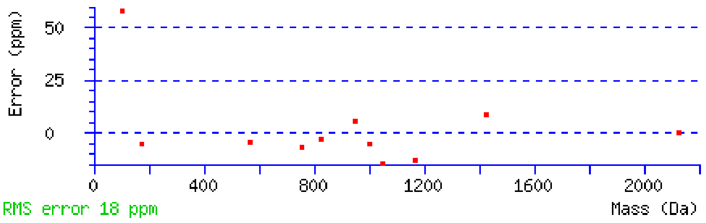

NCBI **BLAST** search of LSVGSVYRPNQNGRGLPDLVPDPNYVQASTYVQR
(Parameters: blastp, nr protein database, expect=20000, no filter, PAM30)
Other BLAST web gateways

All matches to this query

Score	Mr(calc)	Delta	Sequence
22.6	3761.8649	1.0115	LSVGSVYRPNQNGRGLPDLVPDPNYVQASTYVQR
17.4	3760.8809	1.9955	LSVGSVYRPNQNGRGLPDLVPDPNYVQASTYVQR
17.4	3760.8809	1.9955	LSVGSVYRPNQNGRGLPDLVPDPNYVQASTYVQR
17.4	3760.8809	1.9955	LSVGSVYRPNQNGRGLPDLVPDPNYVQASTYVQR
17.0	3760.8809	1.9955	LSVGSVYRPNQNGRGLPDLVPDPNYVQASTYVQR
17.0	3760.8809	1.9955	LSVGSVYRPNQNGRGLPDLVPDPNYVQASTYVQR
17.0	3760.8809	1.9955	LSVGSVYRPNQNGRGLPDLVPDPNYVQASTYVQR
10.4	3761.8649	1.0115	LSVGSVYRPNQNGRGLPDLVPDPNYVQASTYVQR
3.2	3760.8809	1.9955	LSVGSVYRPNQNGRGLPDLVPDPNYVQASTYVQR
3.2	3760.8809	1.9955	LSVGSVYRPNQNGRGLPDLVPDPNYVQASTYVQR

Mascot: http://www.matrixscience.com/

Mascot Search Results

Peptide View

MS/MS Fragmentation of **LSVGSVYRPNQNGRGLPDLVPDPNYVQASTYVQR**
Found in **Q08397** in **uni_human_nr**, LOXL1_HUMAN Lysyl oxidase homolog 1 OS=Homo sapiens GN=LOXL1 PE=1 SV=2

Match to Query 16528: 3760.874456 from(941.225890,4+) intensity(246132.0781) rtinseconds(2842) scans(16585) index(12622)
Title: 130801_HPL_Human_Plaque_BR1_TR1_09_Spectrum033744_scans__16585_RTINSECONDS=2842
Data file E:\\RAW\\HumanPlaques\\mgf\\T\\1D\\T130801_HPL_Human_Plaque_BR1_TR1_09.mgf

Click mouse within plot area to zoom in by factor of two about that point
Or, [Plot from] [0] to [2800] Da [Full range]
Label all possible matches ○ Label matches used for scoring ◉
Show Y-axis ☐

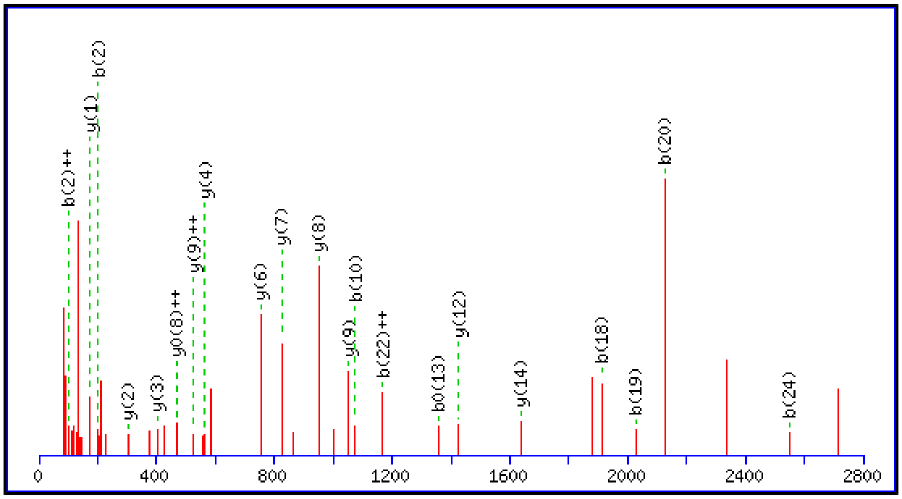

Monoisotopic mass of neutral peptide Mr(calc): 3760.8809
Fixed modifications: Carbamidomethyl (C) (apply to specified residues or termini only)
Variable modifications:
N10 : Deamidated (NQ)
N12 : Deamidated (NQ)
Ions Score: 52 **Expect:** 0.0005
Matches : 21/372 fragment ions using 39 most intense peaks (help)

#	b	b^{++}	b*	b^{*++}	b^0	b^{0++}	Seq.	y	y^{++}	y*	y^{*++}	y^0	y^{0++}	#
1	114.0913	57.5493					L							34
2	***201.1234***	**101.0653**			183.1128	92.0600	S	3648.8041	1824.9057	3631.7776	1816.3924	3630.7936	1815.9004	33
3	300.1918	150.5995			282.1812	141.5942	V	3561.7721	1781.3897	3544.7455	1772.8764	3543.7615	1772.3844	32
4	357.2132	179.1103			339.2027	170.1050	G	3462.7037	1731.8555	3445.6771	1723.3422	3444.6931	1722.8502	31
5	444.2453	222.6263			426.2347	213.6210	S	3405.6822	1703.3447	3388.6557	1694.8315	3387.6716	1694.3395	30
6	543.3137	272.1605			525.3031	263.1552	V	3318.6502	1659.8287	3301.6236	1651.3155	3300.6396	1650.8234	29
7	706.3770	353.6921			688.3665	344.6869	Y	3219.5818	1610.2945	3202.5552	1601.7812	3201.5712	1601.2892	28
8	862.4781	431.7427	845.4516	423.2294	844.4676	422.7374	R	3056.5184	1528.7629	3039.4919	1520.2496	3038.5079	1519.7576	27
9	959.5309	480.2691	942.5043	471.7558	941.5203	471.2638	P	2900.4173	1450.7123	2883.3908	1442.1990	2882.4068	1441.7070	26
10	***1074.5578***	537.7826	1057.5313	529.2693	1056.5473	528.7773	N	2803.3646	1402.1859	2786.3380	1393.6726	2785.3540	1393.1806	25
11	1202.6164	601.8118	1185.5899	593.2986	1184.6058	592.8066	Q	2688.3376	1344.6724	2671.3111	1336.1592	2670.3271	1335.6672	24
12	1317.6434	659.3253	1300.6168	650.8120	1299.6328	650.3200	N	2560.2790	1280.6432	2543.2525	1272.1299	2542.2685	1271.6379	23
13	1374.6648	687.8360	1357.6383	679.3228	1356.6543	678.8308	G	2445.2521	1223.1297	2428.2256	1214.6164	2427.2415	1214.1244	22
14	1530.7659	765.8866	1513.7394	757.3733	1512.7554	756.8813	R	2388.2306	1194.6190	2371.2041	1186.1057	2370.2201	1185.6137	21
15	1587.7874	794.3973	1570.7608	785.8841	1569.7768	785.3921	G	2232.1295	1116.5684	2215.1030	1108.0551	2214.1190	1107.5631	20
16	1700.8715	850.9394	1683.8449	842.4261	1682.8609	841.9341	L	2175.1081	1088.0577	2158.0815	1079.5444	2157.0975	1079.0524	19
17	1797.9242	899.4657	1780.8977	890.9525	1779.9137	890.4605	P	2062.0240	1031.5156	2044.9974	1023.0024	2044.0134	1022.5104	18

**18**	***1912.9512***	956.9792	1895.9246	948.4659	1894.9406	947.9739	**D**	1964.9712	982.9893	1947.9447	974.4760	1946.9607	973.9840	**17**
**19**	***2026.0352***	1013.5213	2009.0087	1005.0080	2008.0247	1004.5160	**L**	1849.9443	925.4758	1832.9177	916.9625	1831.9337	916.4705	**16**
**20**	***2125.1036***	1063.0555	2108.0771	1054.5422	2107.0931	1054.0502	**V**	1736.8602	868.9338	1719.8337	860.4205	1718.8497	859.9285	**15**
**21**	2222.1564	1111.5818	2205.1299	1103.0686	2204.1458	1102.5766	**P**	***1637.7918***	819.3995	1620.7653	810.8863	1619.7812	810.3943	**14**
**22**	2337.1834	**1169.0953**	2320.1568	1160.5820	2319.1728	1160.0900	**D**	1540.7390	770.8732	1523.7125	762.3599	1522.7285	761.8679	**13**
**23**	2434.2361	1217.6217	2417.2096	1209.1084	2416.2255	1208.6164	**P**	***1425.7121***	713.3597	1408.6856	704.8464	1407.7015	704.3544	**12**
**24**	***2548.2790***	1274.6432	2531.2525	1266.1299	2530.2685	1265.6379	**N**	1328.6593	664.8333	1311.6328	656.3200	1310.6488	655.8280	**11**
**25**	2711.3424	1356.1748	2694.3158	1347.6615	2693.3318	1347.1695	**Y**	1214.6164	607.8118	1197.5899	599.2986	1196.6059	598.8066	**10**
**26**	2810.4108	1405.7090	2793.3842	1397.1958	2792.4002	1396.7037	**V**	***1051.5531***	526.2802	1034.5265	517.7669	1033.5425	517.2749	**9**
**27**	2938.4694	1469.7383	2921.4428	1461.2250	2920.4588	1460.7330	**Q**	***952.4847***	476.7460	935.4581	468.2327	934.4741	467.7407	**8**
**28**	3009.5065	1505.2569	2992.4799	1496.7436	2991.4959	1496.2516	**A**	***824.4261***	412.7167	807.3995	404.2034	806.4155	403.7114	**7**
**29**	3096.5385	1548.7729	3079.5120	1540.2596	3078.5279	1539.7676	**S**	***753.3890***	377.1981	736.3624	368.6849	735.3784	368.1928	**6**
**30**	3197.5862	1599.2967	3180.5596	1590.7835	3179.5756	1590.2914	**T**	666.3570	333.6821	649.3304	325.1688	648.3464	324.6768	**5**
**31**	3360.6495	1680.8284	3343.6230	1672.3151	3342.6389	1671.8231	**Y**	***565.3093***	283.1583	548.2827	274.6450			**4**
**32**	3459.7179	1730.3626	3442.6914	1721.8493	3441.7074	1721.3573	**V**	***402.2459***	201.6266	385.2194	193.1133			**3**
**33**	3587.7765	1794.3919	3570.7500	1785.8786	3569.7659	1785.3866	**Q**	***303.1775***	152.0924	286.1510	143.5791			**2**
**34**							**R**	***175.1190***	88.0631	158.0924	79.5498			**1**

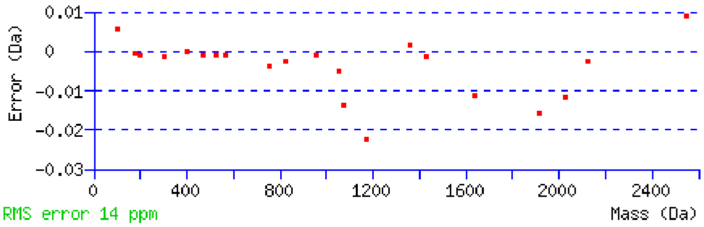

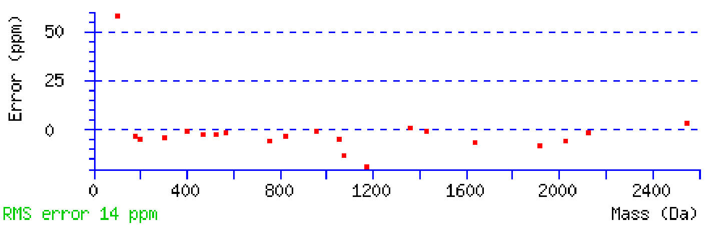

NCBI **BLAST** search of LSVGSVYRPNQNGRGLPDLVPDPNYVQASTYVQR
(Parameters: blastp, nr protein database, expect=20000, no filter, PAM30)
Other BLAST web gateways

All matches to this query

Score	Mr(calc)	Delta	Sequence	Site Analysis
51.8	3760.8809	-0.0064	LSVGSVYRPNQNGRGLPDLVPDPNYVQASTYVQR	Deamidated N10, N12 45.36%
51.8	3760.8809	-0.0064	LSVGSVYRPNQNGRGLPDLVPDPNYVQASTYVQR	Deamidated N10, Q11 45.36%
44.8	3760.8809	-0.0064	LSVGSVYRPNQNGRGLPDLVPDPNYVQASTYVQR	Deamidated Q11, N12 9.16%
21.7	3760.8809	-0.0064	LSVGSVYRPNQNGRGLPDLVPDPNYVQASTYVQR	Deamidated N10, N24 0.04%
20.4	3760.8809	-0.0064	LSVGSVYRPNQNGRGLPDLVPDPNYVQASTYVQR	Deamidated N12, N24 0.03%
20.4	3760.8809	-0.0064	LSVGSVYRPNQNGRGLPDLVPDPNYVQASTYVQR	Deamidated Q11, N24 0.03%
3.8	3760.8809	-0.0064	LSVGSVYRPNQNGRGLPDLVPDPNYVQASTYVQR	Deamidated N10, Q27 0.00%
3.2	3760.8809	-0.0064	LSVGSVYRPNQNGRGLPDLVPDPNYVQASTYVQR	Deamidated N12, Q27 0.00%
3.2	3760.8809	-0.0064	LSVGSVYRPNQNGRGLPDLVPDPNYVQASTYVQR	Deamidated Q11, Q27 0.00%
2.7	3760.8809	-0.0064	LSVGSVYRPNQNGRGLPDLVPDPNYVQASTYVQR	Deamidated N24, Q27 0.00%

Mascot: http://www.matrixscience.com/

Mascot Search Results

Peptide View

MS/MS Fragmentation of **LSVGSVYRPNQNGRGLPDLVPDPNYVQASTYVQR**
Found in **Q08397** in **uni_human_nr**, LOXL1_HUMAN Lysyl oxidase homolog 1 OS=Homo sapiens GN=LOXL1 PE=1 SV=2

Match to Query 20036: 3760.897656 from(941.231690,4+) intensity(870937.9375) rtinseconds(2742) scans(17517) index(14144)
Title: 130806_HPL_Human_Plaque_BR1_TR2_04_Spectrum037558_scans__17517_RTINSECONDS=2742
Data file E:\\RAW\\HumanPlaques\\mgf\\T\\1D\\T130806_HPL_Human_Plaque_BR1_TR2_04.mgf

Click mouse within plot area to zoom in by factor of two about that point
Or, Plot from 0 to 2600 Da Full range
Label all possible matches ○ Label matches used for scoring ⊙
Show Y-axis ☐

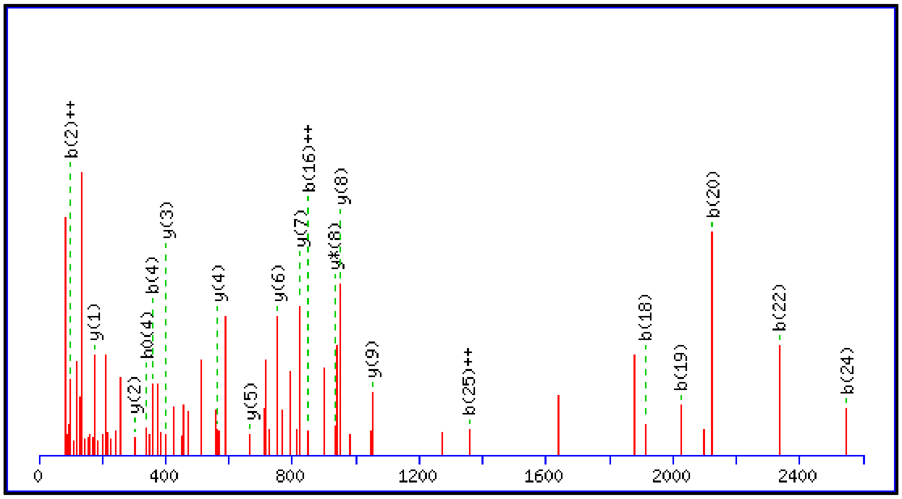

Monoisotopic mass of neutral peptide Mr(calc): 3760.8809
Fixed modifications: Carbamidomethyl (C) (apply to specified residues or termini only)
Variable modifications:
N12 : Deamidated (NQ)
N24 : Deamidated (NQ)
Ions Score: 32 **Expect:** 0.044
Matches : 20/372 fragment ions using 53 most intense peaks (help)

#	b	b^{++}	b*	b^{*++}	b^0	b^{0++}	Seq.	y	y^{++}	y*	y^{*++}	y^0	y^{0++}	#
1	114.0913	57.5493					L							34
2	201.1234	**101.0653**			183.1128	92.0600	S	3648.8041	1824.9057	3631.7776	1816.3924	3630.7936	1815.9004	33
3	300.1918	150.5995			282.1812	141.5942	V	3561.7721	1781.3897	3544.7455	1772.8764	3543.7615	1772.3844	32
4	***357.2132***	179.1103			339.2027	170.1050	G	3462.7037	1731.8555	3445.6771	1723.3422	3444.6931	1722.8502	31
5	444.2453	222.6263			426.2347	213.6210	S	3405.6822	1703.3447	3388.6557	1694.8315	3387.6716	1694.3395	30
6	543.3137	272.1605			525.3031	263.1552	V	3318.6502	1659.8287	3301.6236	1651.3155	3300.6396	1650.8234	29
7	706.3770	353.6921			688.3665	344.6869	Y	3219.5818	1610.2945	3202.5552	1601.7812	3201.5712	1601.2892	28
8	862.4781	431.7427	845.4516	423.2294	844.4676	422.7374	R	3056.5184	1528.7629	3039.4919	1520.2496	3038.5079	1519.7576	27
9	959.5309	480.2691	942.5043	471.7558	941.5203	471.2638	P	2900.4173	1450.7123	2883.3908	1442.1990	2882.4068	1441.7070	26
10	1073.5738	537.2905	1056.5473	528.7773	1055.5633	528.2853	N	2803.3646	1402.1859	2786.3380	1393.6726	2785.3540	1393.1806	25
11	1201.6324	601.3198	1184.6058	592.8066	1183.6218	592.3146	Q	2689.3216	1345.1645	2672.2951	1336.6512	2671.3111	1336.1592	24
12	1316.6593	658.8333	1299.6328	650.3200	1298.6488	649.8280	N	2561.2631	1281.1352	2544.2365	1272.6219	2543.2525	1272.1299	23
13	1373.6808	687.3440	1356.6543	678.8308	1355.6702	678.3388	G	2446.2361	1223.6217	2429.2096	1215.1084	2428.2256	1214.6164	22
14	1529.7819	765.3946	1512.7554	756.8813	1511.7714	756.3893	R	2389.2147	1195.1110	2372.1881	1186.5977	2371.2041	1186.1057	21
15	1586.8034	793.9053	1569.7768	785.3921	1568.7928	784.9000	G	2233.1135	1117.0604	2216.0870	1108.5471	2215.1030	1108.0551	20
16	1699.8874	**850.4474**	1682.8609	841.9341	1681.8769	841.4421	L	2176.0921	1088.5497	2159.0655	1080.0364	2158.0815	1079.5444	19
17	1796.9402	898.9737	1779.9137	890.4605	1778.9296	889.9685	P	2063.0080	1032.0076	2045.9815	1023.4944	2044.9974	1023.0024	18

18	1911.9671	956.4872	1894.9406	947.9739	1893.9566	947.4819	D	1965.9552	983.4813	1948.9287	974.9680	1947.9447	974.4760	17
19	2025.0512	1013.0292	2008.0247	1004.5160	2007.0406	1004.0240	L	1850.9283	925.9678	1833.9018	917.4545	1832.9177	916.9625	16
20	2124.1196	1062.5635	2107.0931	1054.0502	2106.1091	1053.5582	V	1737.8442	869.4258	1720.8177	860.9125	1719.8337	860.4205	15
21	2221.1724	1111.0898	2204.1458	1102.5766	2203.1618	1102.0846	P	1638.7758	819.8916	1621.7493	811.3783	1620.7653	810.8863	14
22	2336.1993	1168.6033	2319.1728	1160.0900	2318.1888	1159.5980	D	1541.7231	771.3652	1524.6965	762.8519	1523.7125	762.3599	13
23	2433.2521	1217.1297	2416.2255	1208.6164	2415.2415	1208.1244	P	1426.6961	713.8517	1409.6696	705.3384	1408.6856	704.8464	12
24	2548.2790	1274.6432	2531.2525	1266.1299	2530.2685	1265.6379	N	1329.6434	665.3253	1312.6168	656.8120	1311.6328	656.3200	11
25	2711.3424	1356.1748	2694.3158	1347.6615	2693.3318	1347.1695	Y	1214.6164	607.8118	1197.5899	599.2986	1196.6059	598.8066	10
26	2810.4108	1405.7090	2793.3842	1397.1958	2792.4002	1396.7037	V	1051.5531	526.2802	1034.5265	517.7669	1033.5425	517.2749	9
27	2938.4694	1469.7383	2921.4428	1461.2250	2920.4588	1460.7330	Q	952.4847	476.7460	935.4581	468.2327	934.4741	467.7407	8
28	3009.5065	1505.2569	2992.4799	1496.7436	2991.4959	1496.2516	A	824.4261	412.7167	807.3995	404.2034	806.4155	403.7114	7
29	3096.5385	1548.7729	3079.5120	1540.2596	3078.5279	1539.7676	S	753.3890	377.1981	736.3624	368.6849	735.3784	368.1928	6
30	3197.5862	1599.2967	3180.5596	1590.7835	3179.5756	1590.2914	T	666.3570	333.6821	649.3304	325.1688	648.3464	324.6768	5
31	3360.6495	1680.8284	3343.6230	1672.3151	3342.6389	1671.8231	Y	565.3093	283.1583	548.2827	274.6450			4
32	3459.7179	1730.3626	3442.6914	1721.8493	3441.7074	1721.3573	V	402.2459	201.6266	385.2194	193.1133			3
33	3587.7765	1794.3919	3570.7500	1785.8786	3569.7659	1785.3866	Q	303.1775	152.0924	286.1510	143.5791			2
34							R	175.1190	88.0631	158.0924	79.5498			1

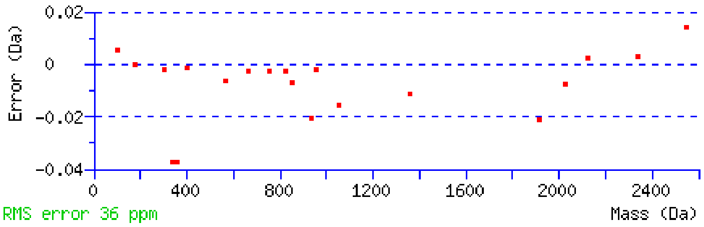

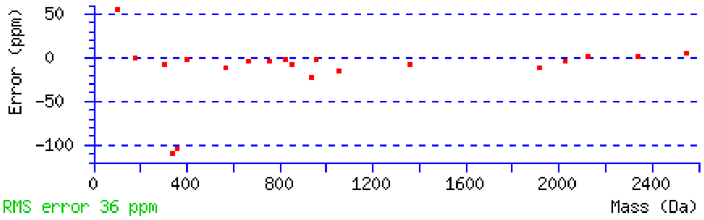

NCBI **BLAST** search of LSVGSVYRPNQNGRGLPDLVPDPNYVQASTYVQR
(Parameters: blastp, nr protein database, expect=20000, no filter, PAM30)
Other BLAST web gateways

All matches to this query

Score	Mr(calc)	Delta	Sequence	Site Analysis
31.9	3760.8809	0.0168	LSVGSVYRPNQNGRGLPDLVPDPNYVQASTYVQR	Deamidated N12, N24 26.84%
31.9	3760.8809	0.0168	LSVGSVYRPNQNGRGLPDLVPDPNYVQASTYVQR	Deamidated Q11, N24 26.84%
31.9	3760.8809	0.0168	LSVGSVYRPNQNGRGLPDLVPDPNYVQASTYVQR	Deamidated N10, N24 26.84%
31.6	3759.8969	1.0008	LSVGSVYRPNQNGRGLPDLVPDPNYVQASTYVQR	
31.6	3759.8969	1.0008	LSVGSVYRPNQNGRGLPDLVPDPNYVQASTYVQR	
31.6	3759.8969	1.0008	LSVGSVYRPNQNGRGLPDLVPDPNYVQASTYVQR	
24.1	3760.8809	0.0168	LSVGSVYRPNQNGRGLPDLVPDPNYVQASTYVQR	Deamidated Q11, N12 4.39%
24.1	3760.8809	0.0168	LSVGSVYRPNQNGRGLPDLVPDPNYVQASTYVQR	Deamidated N10, N12 4.39%
24.1	3760.8809	0.0168	LSVGSVYRPNQNGRGLPDLVPDPNYVQASTYVQR	Deamidated N10, Q11 4.39%
17.1	3759.8969	1.0008	LSVGSVYRPNQNGRGLPDLVPDPNYVQASTYVQR	

Mascot: http://www.matrixscience.com/

Mascot Search Results

Peptide View

MS/MS Fragmentation of **LSVGSVYRPNQNGRGLPDLVPDPNYVQASTYVQR**
Found in **Q08397** in **uni_human_nr**, LOXL1_HUMAN Lysyl oxidase homolog 1 OS=Homo sapiens GN=LOXL1 PE=1 SV=2

Match to Query 19403: 3762.893016 from(941.730530,4+) intensity(695549.1250) rtinseconds(2739) scans(17049) index(13721)
Title: 130801_HPL_Human_Plaque_BR2_TR1_04_Spectrum036295_scans__17049_RTINSECONDS=2739
Data file E:\\RAW\\HumanPlaques\\mgf\\T\\1D\\T130801_HPL_Human_Plaque_BR2_TR1_04.mgf

Click mouse within plot area to zoom in by factor of two about that point
Or, [Plot from] [0] to [3000] Da [Full range]
Label all possible matches ○ Label matches used for scoring ⦿
Show Y-axis ☐

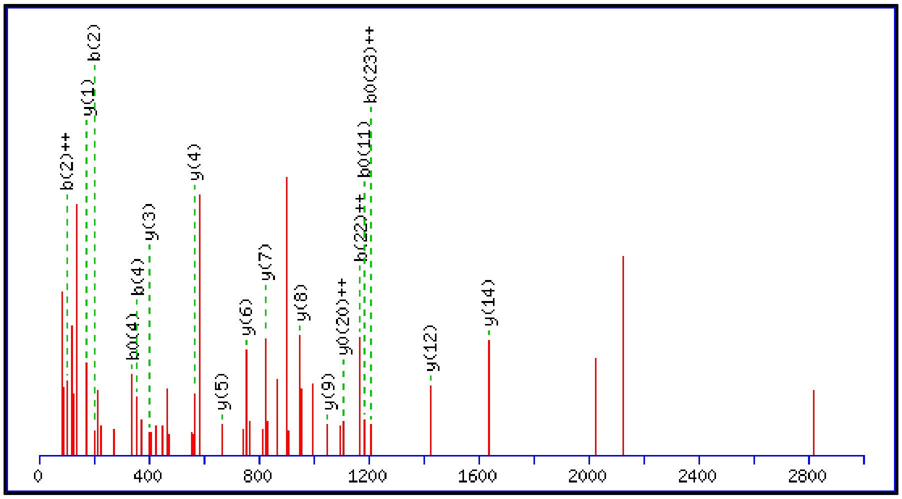

Monoisotopic mass of neutral peptide Mr(calc): 3760.8809
Fixed modifications: Carbamidomethyl (C) (apply to specified residues or termini only)
Variable modifications:
Q11 : Deamidated (NQ)
N12 : Deamidated (NQ)
Ions Score: 28 **Expect:** 0.12
Matches : 18/372 fragment ions using 50 most intense peaks (help)

#	b	b^{++}	b*	b^{*++}	b^0	b^{0++}	Seq.	y	y^{++}	y*	y^{*++}	y^0	y^{0++}	#
1	114.0913	57.5493					L							34
2	**201.1234**	**101.0653**			183.1128	92.0600	S	3648.8041	1824.9057	3631.7776	1816.3924	3630.7936	1815.9004	33
3	300.1918	150.5995			282.1812	141.5942	V	3561.7721	1781.3897	3544.7455	1772.8764	3543.7615	1772.3844	32
4	**357.2132**	179.1103			339.2027	170.1050	G	3462.7037	1731.8555	3445.6771	1723.3422	3444.6931	1722.8502	31
5	444.2453	222.6263			426.2347	213.6210	S	3405.6822	1703.3447	3388.6557	1694.8315	3387.6716	1694.3395	30
6	543.3137	272.1605			525.3031	263.1552	V	3318.6502	1659.8287	3301.6236	1651.3155	3300.6396	1650.8234	29
7	706.3770	353.6921			688.3665	344.6869	Y	3219.5818	1610.2945	3202.5552	1601.7812	3201.5712	1601.2892	28
8	862.4781	431.7427	845.4516	423.2294	844.4676	422.7374	R	3056.5184	1528.7629	3039.4919	1520.2496	3038.5079	1519.7576	27
9	959.5309	480.2691	942.5043	471.7558	941.5203	471.2638	P	2900.4173	1450.7123	2883.3908	1442.1990	2882.4068	1441.7070	26
10	1073.5738	537.2905	1056.5473	528.7773	1055.5633	528.2853	N	2803.3646	1402.1859	2786.3380	1393.6726	2785.3540	1393.1806	25
11	1202.6164	601.8118	1185.5899	593.2986	1184.6058	592.8066	Q	2689.3216	1345.1645	2672.2951	1336.6512	2671.3111	1336.1592	24
12	1317.6434	659.3253	1300.6168	650.8120	1299.6328	650.3200	N	2560.2790	1280.6432	2543.2525	1272.1299	2542.2685	1271.6379	23
13	1374.6648	687.8360	1357.6383	679.3228	1356.6543	678.8308	G	2445.2521	1223.1297	2428.2256	1214.6164	2427.2415	1214.1244	22
14	1530.7659	765.8866	1513.7394	757.3733	1512.7554	756.8813	R	2388.2306	1194.6190	2371.2041	1186.1057	2370.2201	1185.6137	21
15	1587.7874	794.3973	1570.7608	785.8841	1569.7768	785.3921	G	2232.1295	1116.5684	2215.1030	1108.0551	2214.1190	1107.5631	20
16	1700.8715	850.9394	1683.8449	842.4261	1682.8609	841.9341	L	2175.1081	1088.0577	2158.0815	1079.5444	2157.0975	1079.0524	19
17	1797.9242	899.4657	1780.8977	890.9525	1779.9137	890.4605	P	2062.0240	1031.5156	2044.9974	1023.0024	2044.0134	1022.5104	18

http://mascotxp.student.main.ntu.edu.sg/mascot/cgi/peptide_view.pl?file=F:/data/20160625/F018266.dat&hit=1&px=1&query=19403[12/15/2016 7:45:03 PM]

**18**	1912.9512	956.9792	1895.9246	948.4659	1894.9406	947.9739	**D**	1964.9712	982.9893	1947.9447	974.4760	1946.9607	973.9840	**17**
**19**	2026.0352	1013.5213	2009.0087	1005.0080	2008.0247	1004.5160	**L**	1849.9443	925.4758	1832.9177	916.9625	1831.9337	916.4705	**16**
**20**	2125.1036	1063.0555	2108.0771	1054.5422	2107.0931	1054.0502	**V**	1736.8602	868.9338	1719.8337	860.4205	1718.8497	859.9285	**15**
**21**	2222.1564	1111.5818	2205.1299	1103.0686	2204.1458	1102.5766	**P**	***1637.7918***	819.3995	1620.7653	810.8863	1619.7812	810.3943	**14**
**22**	2337.1834	**1169.0953**	2320.1568	1160.5820	2319.1728	1160.0900	**D**	1540.7390	770.8732	1523.7125	762.3599	1522.7285	761.8679	**13**
**23**	2434.2361	1217.6217	2417.2096	1209.1084	2416.2255	1208.6164	**P**	***1425.7121***	713.3597	1408.6856	704.8464	1407.7015	704.3544	**12**
**24**	2548.2790	1274.6432	2531.2525	1266.1299	2530.2685	1265.6379	**N**	1328.6593	664.8333	1311.6328	656.3200	1310.6488	655.8280	**11**
**25**	2711.3424	1356.1748	2694.3158	1347.6615	2693.3318	1347.1695	**Y**	1214.6164	607.8118	1197.5899	599.2986	1196.6059	598.8066	**10**
**26**	2810.4108	1405.7090	2793.3842	1397.1958	2792.4002	1396.7037	**V**	***1051.5531***	526.2802	1034.5265	517.7669	1033.5425	517.2749	**9**
**27**	2938.4694	1469.7383	2921.4428	1461.2250	2920.4588	1460.7330	**Q**	***952.4847***	476.7460	935.4581	468.2327	934.4741	467.7407	**8**
**28**	3009.5065	1505.2569	2992.4799	1496.7436	2991.4959	1496.2516	**A**	***824.4261***	412.7167	807.3995	404.2034	806.4155	403.7114	**7**
**29**	3096.5385	1548.7729	3079.5120	1540.2596	3078.5279	1539.7676	**S**	***753.3890***	377.1981	736.3624	368.6849	735.3784	368.1928	**6**
**30**	3197.5862	1599.2967	3180.5596	1590.7835	3179.5756	1590.2914	**T**	***666.3570***	333.6821	649.3304	325.1688	648.3464	324.6768	**5**
**31**	3360.6495	1680.8284	3343.6230	1672.3151	3342.6389	1671.8231	**Y**	***565.3093***	283.1583	548.2827	274.6450			**4**
**32**	3459.7179	1730.3626	3442.6914	1721.8493	3441.7074	1721.3573	**V**	***402.2459***	201.6266	385.2194	193.1133			**3**
**33**	3587.7765	1794.3919	3570.7500	1785.8786	3569.7659	1785.3866	**Q**	303.1775	152.0924	286.1510	143.5791			**2**
**34**							**R**	***175.1190***	88.0631	158.0924	79.5498			**1**

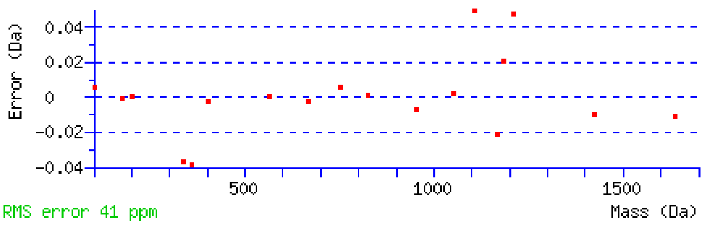

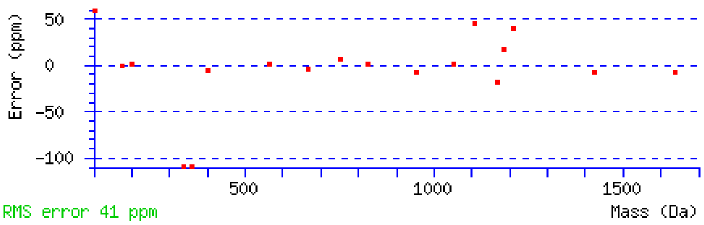

NCBI **BLAST** search of LSVGSVYRPNQNGRGLPDLVPDPNYVQASTYVQR
(Parameters: blastp, nr protein database, expect=20000, no filter, PAM30)
Other BLAST web gateways

All matches to this query

Score	Mr(calc)	Delta	Sequence
27.7	3760.8809	2.0121	LSVGSVYRPNQNGRGLPDLVPDPNYVQASTYVQR
27.7	3760.8809	2.0121	LSVGSVYRPNQNGRGLPDLVPDPNYVQASTYVQR
26.4	3760.8809	2.0121	LSVGSVYRPNQNGRGLPDLVPDPNYVQASTYVQR
20.0	3760.8809	2.0121	LSVGSVYRPNQNGRGLPDLVPDPNYVQASTYVQR
20.0	3760.8809	2.0121	LSVGSVYRPNQNGRGLPDLVPDPNYVQASTYVQR
20.0	3760.8809	2.0121	LSVGSVYRPNQNGRGLPDLVPDPNYVQASTYVQR
10.9	3760.8809	2.0121	LSVGSVYRPNQNGRGLPDLVPDPNYVQASTYVQR
10.9	3760.8809	2.0121	LSVGSVYRPNQNGRGLPDLVPDPNYVQASTYVQR
10.9	3760.8809	2.0121	LSVGSVYRPNQNGRGLPDLVPDPNYVQASTYVQR
1.4	3760.8809	2.0121	LSVGSVYRPNQNGRGLPDLVPDPNYVQASTYVQR

Mascot: http://www.matrixscience.com/

Mascot Search Results

Peptide View

MS/MS Fragmentation of **LSVGSVYRPNQNGRGLPDLVPDPNYVQASTYVQR**
Found in **Q08397** in **uni_human_nr**, LOXL1_HUMAN Lysyl oxidase homolog 1 OS=Homo sapiens GN=LOXL1 PE=1 SV=2

Match to Query 19604: 3760.901056 from(941.232540,4+) intensity(1145813.7500) rtinseconds(2733) scans(17188) index(13750)
Title: 130801_HPL_Human_Plaque_BR1_TR1_04_Spectrum036748_scans__17188_RTINSECONDS=2733
Data file E:\\RAW\\HumanPlaques\\mgf\\T\\1D\\T130801_HPL_Human_Plaque_BR1_TR1_04.mgf

Click mouse within plot area to zoom in by factor of two about that point
Or, Plot from 0 to 3200 Da Full range
Label all possible matches ○ Label matches used for scoring ◉
Show Y-axis ☐

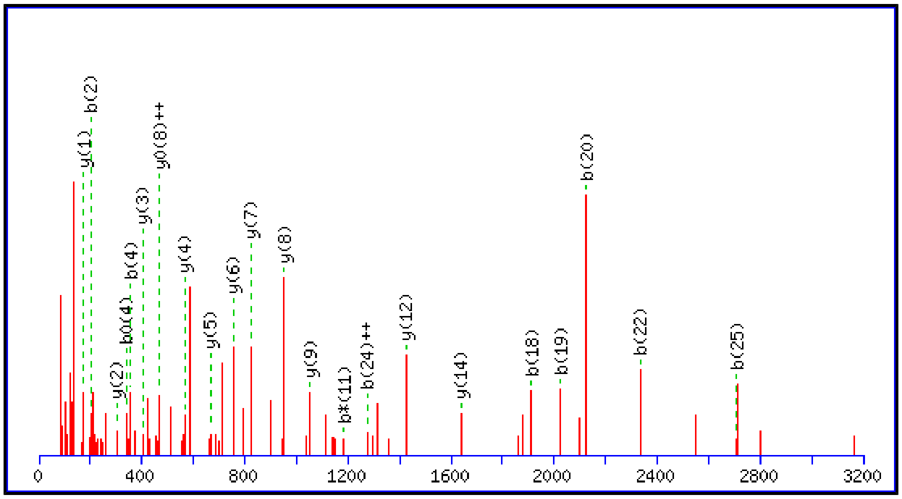

Monoisotopic mass of neutral peptide Mr(calc): 3759.8969
Fixed modifications: Carbamidomethyl (C) (apply to specified residues or termini only)
Variable modifications:
N12 : Deamidated (NQ)
Ions Score: 37 **Expect:** 0.013
Matches : 22/372 fragment ions using 58 most intense peaks (help)

#	b	b^{++}	b*	b^{*++}	b^0	b^{0++}	Seq.	y	y^{++}	y*	y^{*++}	y^0	y^{0++}	#
1	114.0913	57.5493					L							34
2	***201.1234***	101.0653			183.1128	92.0600	S	3647.8201	1824.4137	3630.7936	1815.9004	3629.8095	1815.4084	33
3	300.1918	150.5995			282.1812	141.5942	V	3560.7881	1780.8977	3543.7615	1772.3844	3542.7775	1771.8924	32
4	***357.2132***	179.1103			339.2027	170.1050	G	3461.7197	1731.3635	3444.6931	1722.8502	3443.7091	1722.3582	31
5	444.2453	222.6263			426.2347	213.6210	S	3404.6982	1702.8527	3387.6716	1694.3395	3386.6876	1693.8475	30
6	543.3137	272.1605			525.3031	263.1552	V	3317.6662	1659.3367	3300.6396	1650.8234	3299.6556	1650.3314	29
7	706.3770	353.6921			688.3665	344.6869	Y	3218.5978	1609.8025	3201.5712	1601.2892	3200.5872	1600.7972	28
8	862.4781	431.7427	845.4516	423.2294	844.4676	422.7374	R	3055.5344	1528.2708	3038.5079	1519.7576	3037.5239	1519.2656	27
9	959.5309	480.2691	942.5043	471.7558	941.5203	471.2638	P	2899.4333	1450.2203	2882.4068	1441.7070	2881.4227	1441.2150	26
10	1073.5738	537.2905	1056.5473	528.7773	1055.5633	528.2853	N	2802.3805	1401.6939	2785.3540	1393.1806	2784.3700	1392.6886	25
11	1201.6324	601.3198	1184.6058	592.8066	1183.6218	592.3146	Q	2688.3376	1344.6724	2671.3111	1336.1592	2670.3271	1335.6672	24
12	1316.6593	658.8333	1299.6328	650.3200	1298.6488	649.8280	N	2560.2790	1280.6432	2543.2525	1272.1299	2542.2685	1271.6379	23
13	1373.6808	687.3440	1356.6543	678.8308	1355.6702	678.3388	G	2445.2521	1223.1297	2428.2256	1214.6164	2427.2415	1214.1244	22
14	1529.7819	765.3946	1512.7554	756.8813	1511.7714	756.3893	R	2388.2306	1194.6190	2371.2041	1186.1057	2370.2201	1185.6137	21
15	1586.8034	793.9053	1569.7768	785.3921	1568.7928	784.9000	G	2232.1295	1116.5684	2215.1030	1108.0551	2214.1190	1107.5631	20
16	1699.8874	850.4474	1682.8609	841.9341	1681.8769	841.4421	L	2175.1081	1088.0577	2158.0815	1079.5444	2157.0975	1079.0524	19
17	1796.9402	898.9737	1779.9137	890.4605	1778.9296	889.9685	P	2062.0240	1031.5156	2044.9974	1023.0024	2044.0134	1022.5104	18
18	***1911.9671***	956.4872	1894.9406	947.9739	1893.9566	947.4819	D	1964.9712	982.9893	1947.9447	974.4760	1946.9607	973.9840	17

**19**	***2025.0512***	1013.0292	2008.0247	1004.5160	2007.0406	1004.0240	**L**	1849.9443	925.4758	1832.9177	916.9625	1831.9337	916.4705	**16**
**20**	***2124.1196***	1062.5635	2107.0931	1054.0502	2106.1091	1053.5582	**V**	1736.8602	868.9338	1719.8337	860.4205	1718.8497	859.9285	**15**
**21**	2221.1724	1111.0898	2204.1458	1102.5766	2203.1618	1102.0846	**P**	***1637.7918***	819.3995	1620.7653	810.8863	1619.7812	810.3943	**14**
**22**	***2336.1993***	1168.6033	2319.1728	1160.0900	2318.1888	1159.5980	**D**	1540.7390	770.8732	1523.7125	762.3599	1522.7285	761.8679	**13**
**23**	2433.2521	1217.1297	2416.2255	1208.6164	2415.2415	1208.1244	**P**	***1425.7121***	713.3597	1408.6856	704.8464	1407.7015	704.3544	**12**
**24**	2547.2950	**1274.1512**	2530.2685	1265.6379	2529.2845	1265.1459	**N**	1328.6593	664.8333	1311.6328	656.3200	1310.6488	655.8280	**11**
**25**	***2710.3584***	1355.6828	2693.3318	1347.1695	2692.3478	1346.6775	**Y**	1214.6164	607.8118	1197.5899	599.2986	1196.6059	598.8066	**10**
**26**	2809.4268	1405.2170	2792.4002	1396.7037	2791.4162	1396.2117	**V**	***1051.5531***	526.2802	1034.5265	517.7669	1033.5425	517.2749	**9**
**27**	2937.4853	1469.2463	2920.4588	1460.7330	2919.4748	1460.2410	**Q**	***952.4847***	476.7460	935.4581	468.2327	934.4741	467.7407	**8**
**28**	3008.5225	1504.7649	2991.4959	1496.2516	2990.5119	1495.7596	**A**	***824.4261***	412.7167	807.3995	404.2034	806.4155	403.7114	**7**
**29**	3095.5545	1548.2809	3078.5279	1539.7676	3077.5439	1539.2756	**S**	***753.3890***	377.1981	736.3624	368.6849	735.3784	368.1928	**6**
**30**	3196.6022	1598.8047	3179.5756	1590.2914	3178.5916	1589.7994	**T**	***666.3570***	333.6821	649.3304	325.1688	648.3464	324.6768	**5**
**31**	3359.6655	1680.3364	3342.6389	1671.8231	3341.6549	1671.3311	**Y**	***565.3093***	283.1583	548.2827	274.6450			**4**
**32**	3458.7339	1729.8706	3441.7074	1721.3573	3440.7233	1720.8653	**V**	***402.2459***	201.6266	385.2194	193.1133			**3**
**33**	3586.7925	1793.8999	3569.7659	1785.3866	3568.7819	1784.8946	**Q**	***303.1775***	152.0924	286.1510	143.5791			**2**
**34**							**R**	***175.1190***	88.0631	158.0924	79.5498			**1**

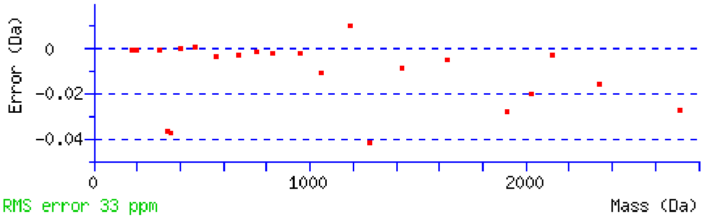

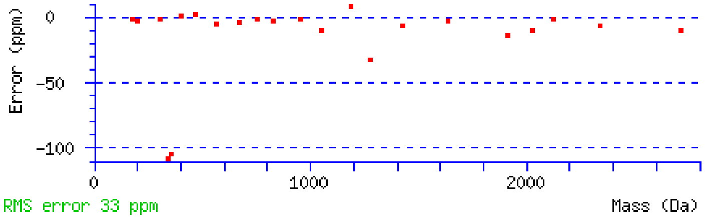

NCBI **BLAST** search of LSVGSVYRPNQNGRGLPDLVPDPNYVQASTYVQR
(Parameters: blastp, nr protein database, expect=20000, no filter, PAM30)
Other BLAST web gateways

All matches to this query

Score	Mr(calc)	Delta	Sequence	Site Analysis
37.2	3759.8969	1.0042	LSVGSVYRPNQNGRGLPDLVPDPNYVQASTYVQR	Deamidated N12 33.24%
37.2	3759.8969	1.0042	LSVGSVYRPNQNGRGLPDLVPDPNYVQASTYVQR	Deamidated Q11 33.24%
37.2	3759.8969	1.0042	LSVGSVYRPNQNGRGLPDLVPDPNYVQASTYVQR	Deamidated N10 33.24%
25.8	3758.9129	1.9882	LSVGSVYRPNQNGRGLPDLVPDPNYVQASTYVQR	
16.1	3759.8969	1.0042	LSVGSVYRPNQNGRGLPDLVPDPNYVQASTYVQR	Deamidated N24 0.25%
1.8	3759.8969	1.0042	LSVGSVYRPNQNGRGLPDLVPDPNYVQASTYVQR	Deamidated Q27 0.01%

Mascot: http://www.matrixscience.com/

Mascot Search Results

Peptide View

MS/MS Fragmentation of **SNGRGGSQAGGSGSAGQYGSDQQHHLGSGSGAGGTGGPAGQAGR**
Found in **Q9NP98** in **uni_human_nr**, MYOZ1_HUMAN Myozenin-1 OS=Homo sapiens GN=MYOZ1 PE=1 SV=1

Match to Query 18184: 3882.697070 from(777.546690,5+) intensity(418300.5313) rtinseconds(1006) scans(4114) index(2306)
Title: 130806_HPL_Human_Plaque_BR1_TR2_07_Spectrum024277_scans__4114_RTINSECONDS=1006
Data file E:\\RAW\\HumanPlaques\\mgf\\T\\1D\\T130806_HPL_Human_Plaque_BR1_TR2_07.mgf

Click mouse within plot area to zoom in by factor of two about that point
Or, Plot from 0 to 3400 Da Full range
Label all possible matches ○ Label matches used for scoring ⦿
Show Y-axis ☐

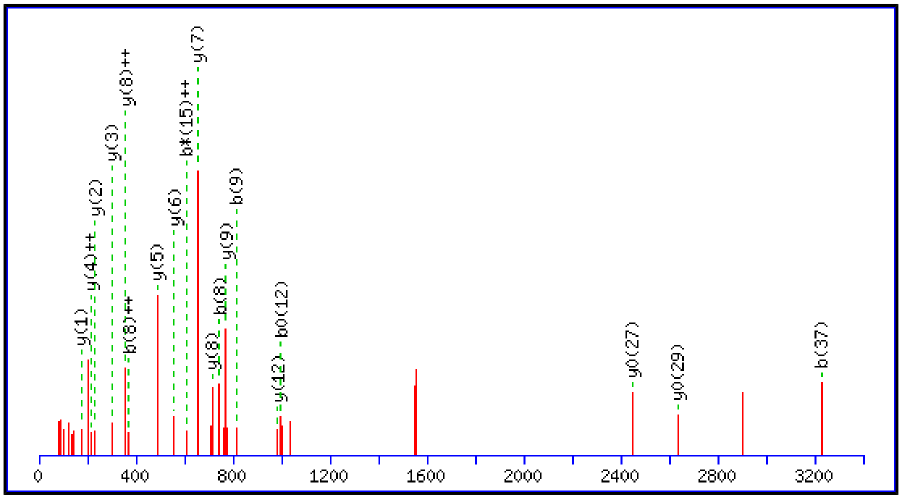

Monoisotopic mass of neutral peptide Mr(calc): 3882.6999
Fixed modifications: Carbamidomethyl (C) (apply to specified residues or termini only)
Variable modifications:
Q8 : Deamidated (NQ)
Ions Score: 39 **Expect:** 0.0037
Matches : 19/496 fragment ions using 34 most intense peaks (help)

#	b	b^{++}	b*	b^{*++}	b^0	b^{0++}	Seq.	y	y^{++}	y*	y^{*++}	y^0	y^{0++}	#
1	88.0393	44.5233			70.0287	35.5180	S							44
2	202.0822	101.5448	185.0557	93.0315	184.0717	92.5395	N	3796.6751	1898.8412	3779.6486	1890.3279	3778.6646	1889.8359	43
3	259.1037	130.0555	242.0771	121.5422	241.0931	121.0502	G	3682.6322	1841.8197	3665.6056	1833.3065	3664.6216	1832.8145	42
4	415.2048	208.1060	398.1783	199.5928	397.1942	199.1008	R	3625.6107	1813.3090	3608.5842	1804.7957	3607.6002	1804.3037	41
5	472.2263	236.6168	455.1997	228.1035	454.2157	227.6115	G	3469.5096	1735.2584	3452.4831	1726.7452	3451.4991	1726.2532	40
6	529.2477	265.1275	512.2212	256.6142	511.2372	256.1222	G	3412.4882	1706.7477	3395.4616	1698.2344	3394.4776	1697.7424	39
7	616.2798	308.6435	599.2532	300.1302	598.2692	299.6382	S	3355.4667	1678.2370	3338.4401	1669.7237	3337.4561	1669.2317	38
8	**745.3224**	**373.1648**	728.2958	364.6515	727.3118	364.1595	Q	3268.4347	1634.7210	3251.4081	1626.2077	3250.4241	1625.7157	37
9	**816.3595**	408.6834	799.3329	400.1701	798.3489	399.6781	A	3139.3921	1570.1997	3122.3655	1561.6864	3121.3815	1561.1944	36
10	873.3809	437.1941	856.3544	428.6808	855.3704	428.1888	G	3068.3550	1534.6811	3051.3284	1526.1678	3050.3444	1525.6758	35
11	930.4024	465.7048	913.3758	457.1916	912.3918	456.6996	G	3011.3335	1506.1704	2994.3069	1497.6571	2993.3229	1497.1651	34
12	1017.4344	509.2209	1000.4079	500.7076	999.4239	500.2156	S	2954.3120	1477.6597	2937.2855	1469.1464	2936.3015	1468.6544	33
13	1074.4559	537.7316	1057.4293	529.2183	1056.4453	528.7263	G	2867.2800	1434.1436	2850.2535	1425.6304	2849.2694	1425.1384	32
14	1161.4879	581.2476	1144.4614	572.7343	1143.4774	572.2423	S	2810.2585	1405.6329	2793.2320	1397.1196	2792.2480	1396.6276	31
15	1232.5250	616.7662	1215.4985	608.2529	1214.5145	607.7609	A	2723.2265	1362.1169	2706.2000	1353.6036	2705.2159	1353.1116	30
16	1289.5465	645.2769	1272.5199	636.7636	1271.5359	636.2716	G	2652.1894	1326.5983	2635.1628	1318.0851	2634.1788	1317.5931	29
17	1417.6051	709.3062	1400.5785	700.7929	1399.5945	700.3009	Q	2595.1679	1298.0876	2578.1414	1289.5743	2577.1574	1289.0823	28
18	1580.6684	790.8378	1563.6419	782.3246	1562.6578	781.8326	Y	2467.1094	1234.0583	2450.0828	1225.5450	2449.0988	1225.0530	27

**19**	1637.6899	819.3486	1620.6633	810.8353	1619.6793	810.3433	**G**	2304.0460	1152.5267	2287.0195	1144.0134	2286.0355	1143.5214	**26**
**20**	1724.7219	862.8646	1707.6953	854.3513	1706.7113	853.8593	**S**	2247.0246	1124.0159	2229.9980	1115.5026	2229.0140	1115.0106	**25**
**21**	1839.7488	920.3781	1822.7223	911.8648	1821.7383	911.3728	**D**	2159.9925	1080.4999	2142.9660	1071.9866	2141.9820	1071.4946	**24**
**22**	1967.8074	984.4073	1950.7809	975.8941	1949.7969	975.4021	**Q**	2044.9656	1022.9864	2027.9390	1014.4732	2026.9550	1013.9812	**23**
**23**	2095.8660	1048.4366	2078.8394	1039.9234	2077.8554	1039.4314	**Q**	1916.9070	958.9571	1899.8805	950.4439	1898.8964	949.9519	**22**
**24**	2232.9249	1116.9661	2215.8984	1108.4528	2214.9143	1107.9608	**H**	1788.8484	894.9279	1771.8219	886.4146	1770.8379	885.9226	**21**
**25**	2369.9838	1185.4955	2352.9573	1176.9823	2351.9733	1176.4903	**H**	1651.7895	826.3984	1634.7630	817.8851	1633.7790	817.3931	**20**
**26**	2483.0679	1242.0376	2466.0413	1233.5243	2465.0573	1233.0323	**L**	1514.7306	757.8689	1497.7041	749.3557	1496.7200	748.8637	**19**
**27**	2540.0893	1270.5483	2523.0628	1262.0350	2522.0788	1261.5430	**G**	1401.6465	701.3269	1384.6200	692.8136	1383.6360	692.3216	**18**
**28**	2627.1214	1314.0643	2610.0948	1305.5511	2609.1108	1305.0590	**S**	1344.6251	672.8162	1327.5985	664.3029	1326.6145	663.8109	**17**
**29**	2684.1428	1342.5751	2667.1163	1334.0618	2666.1323	1333.5698	**G**	1257.5931	629.3002	1240.5665	620.7869	1239.5825	620.2949	**16**
**30**	2771.1749	1386.0911	2754.1483	1377.5778	2753.1643	1377.0858	**S**	1200.5716	600.7894	1183.5450	592.2762	1182.5610	591.7842	**15**
**31**	2828.1963	1414.6018	2811.1698	1406.0885	2810.1858	1405.5965	**G**	1113.5396	557.2734	1096.5130	548.7601	1095.5290	548.2681	**14**
**32**	2899.2334	1450.1204	2882.2069	1441.6071	2881.2229	1441.1151	**A**	1056.5181	528.7627	1039.4916	520.2494	1038.5075	519.7574	**13**
**33**	2956.2549	1478.6311	2939.2284	1470.1178	2938.2443	1469.6258	**G**	***985.4810***	493.2441	968.4544	484.7309	967.4704	484.2388	**12**
**34**	3013.2764	1507.1418	2996.2498	1498.6285	2995.2658	1498.1365	**G**	928.4595	464.7334	911.4330	456.2201	910.4490	455.7281	**11**
**35**	3114.3241	1557.6657	3097.2975	1549.1524	3096.3135	1548.6604	**T**	871.4381	436.2227	854.4115	427.7094	853.4275	427.2174	**10**
**36**	3171.3455	1586.1764	3154.3190	1577.6631	3153.3349	1577.1711	**G**	***770.3904***	385.6988	753.3638	377.1856			**9**
**37**	3228.3670	1614.6871	3211.3404	1606.1739	3210.3564	1605.6818	**G**	***713.3689***	357.1881	696.3424	348.6748			**8**
**38**	3325.4197	1663.2135	3308.3932	1654.7002	3307.4092	1654.2082	**P**	***656.3475***	328.6774	639.3209	320.1641			**7**
**39**	3396.4569	1698.7321	3379.4303	1690.2188	3378.4463	1689.7268	**A**	***559.2947***	280.1510	542.2681	271.6377			**6**
**40**	3453.4783	1727.2428	3436.4518	1718.7295	3435.4678	1718.2375	**G**	***488.2576***	244.6324	471.2310	236.1191			**5**
**41**	3581.5369	1791.2721	3564.5103	1782.7588	3563.5263	1782.2668	**Q**	431.2361	216.1217	414.2096	207.6084			**4**
**42**	3652.5740	1826.7906	3635.5475	1818.2774	3634.5634	1817.7854	**A**	***303.1775***	152.0924	286.1510	143.5791			**3**
**43**	3709.5955	1855.3014	3692.5689	1846.7881	3691.5849	1846.2961	**G**	***232.1404***	116.5738	215.1139	108.0606			**2**
**44**							**R**	***175.1190***	88.0631	158.0924	79.5498			**1**

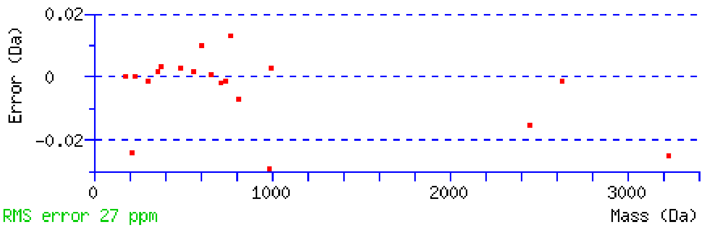

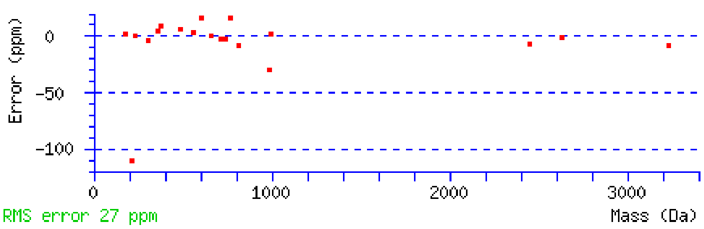

NCBI **BLAST** search of SNGRGGSQAGGSGSAGQYGSDQQHHLGSGSGAGGTGGPAGQAGR
(Parameters: blastp, nr protein database, expect=20000, no filter, PAM30)
Other BLAST web gateways

All matches to this query

Score	Mr(calc)	Delta	Sequence	Site Analysis
39.3	3882.6999	-0.0029	SNGRGGSQAGGSGSAGQYGSDQQHHLGSGSGAGGTGGPAGQAGR	Deamidated Q8 40.09%
39.3	3882.6999	-0.0029	SNGRGGSQAGGSGSAGQYGSDQQHHLGSGSGAGGTGGPAGQAGR	Deamidated N2 40.09%
31.5	3882.6999	-0.0029	SNGRGGSQAGGSGSAGQYGSDQQHHLGSGSGAGGTGGPAGQAGR	Deamidated Q23 6.65%
31.5	3882.6999	-0.0029	SNGRGGSQAGGSGSAGQYGSDQQHHLGSGSGAGGTGGPAGQAGR	Deamidated Q22 6.65%
31.4	3882.6999	-0.0029	SNGRGGSQAGGSGSAGQYGSDQQHHLGSGSGAGGTGGPAGQAGR	Deamidated Q17 6.52%
3.0	3881.6792	1.0179	QGAACRTSECCFQDPPYPDADSGSLPGSASGPRDLR	

Mascot: http://www.matrixscience.com/

Mascot Search Results

Peptide View

MS/MS Fragmentation of **SNGRGGSQAGGSGSAGQYGSDQQHHLGSGSGAGGTGGPAGQAGR**
Found in **Q9NP98** in **uni_human_nr**, MYOZ1_HUMAN Myozenin-1 OS=Homo sapiens GN=MYOZ1 PE=1 SV=1

Match to Query 18185: 3882.703470 from(777.547970,5+) intensity(93002.0625) rtinseconds(1043) scans(4375) index(2533)
Title: 130806_HPL_Human_Plaque_BR1_TR2_07_Spectrum024504_scans__4375_RTINSECONDS=1043
Data file E:\\RAW\\HumanPlaques\\mgf\\T\\1D\\T130806_HPL_Human_Plaque_BR1_TR2_07.mgf

Click mouse within plot area to zoom in by factor of two about that point
Or, Plot from 100 to 950 Da Full range
Label all possible matches ○ Label matches used for scoring ⊙
Show Y-axis ☐

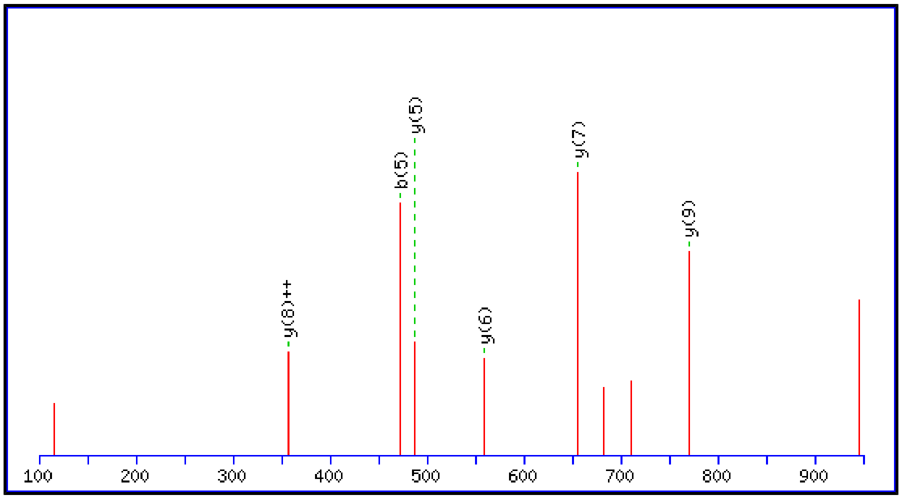

Monoisotopic mass of neutral peptide Mr(calc): 3882.6999
Fixed modifications: Carbamidomethyl (C) (apply to specified residues or termini only)
Variable modifications:
N2 : Deamidated (NQ)
Ions Score: 23 **Expect:** 0.15
Matches : 6/496 fragment ions using 9 most intense peaks (help)

#	b	b^{++}	b*	b^{*++}	b^0	b^{0++}	Seq.	y	y^{++}	y*	y^{*++}	y^0	y^{0++}	#
1	88.0393	44.5233			70.0287	35.5180	S							44
2	203.0662	102.0368	186.0397	93.5235	185.0557	93.0315	N	3796.6751	1898.8412	3779.6486	1890.3279	3778.6646	1889.8359	43
3	260.0877	130.5475	243.0612	122.0342	242.0771	121.5422	G	3681.6482	1841.3277	3664.6216	1832.8145	3663.6376	1832.3224	42
4	416.1888	208.5980	399.1623	200.0848	398.1783	199.5928	R	3624.6267	1812.8170	3607.6002	1804.3037	3606.6162	1803.8117	41
5	**473.2103**	237.1088	456.1837	228.5955	455.1997	228.1035	G	3468.5256	1734.7664	3451.4991	1726.2532	3450.5150	1725.7612	40
6	530.2318	265.6195	513.2052	257.1062	512.2212	256.6142	G	3411.5041	1706.2557	3394.4776	1697.7424	3393.4936	1697.2504	39
7	617.2638	309.1355	600.2372	300.6223	599.2532	300.1302	S	3354.4827	1677.7450	3337.4561	1669.2317	3336.4721	1668.7397	38
8	745.3224	373.1648	728.2958	364.6515	727.3118	364.1595	Q	3267.4507	1634.2290	3250.4241	1625.7157	3249.4401	1625.2237	37
9	816.3595	408.6834	799.3329	400.1701	798.3489	399.6781	A	3139.3921	1570.1997	3122.3655	1561.6864	3121.3815	1561.1944	36
10	873.3809	437.1941	856.3544	428.6808	855.3704	428.1888	G	3068.3550	1534.6811	3051.3284	1526.1678	3050.3444	1525.6758	35
11	930.4024	465.7048	913.3758	457.1916	912.3918	456.6996	G	3011.3335	1506.1704	2994.3069	1497.6571	2993.3229	1497.1651	34
12	1017.4344	509.2209	1000.4079	500.7076	999.4239	500.2156	S	2954.3120	1477.6597	2937.2855	1469.1464	2936.3015	1468.6544	33
13	1074.4559	537.7316	1057.4293	529.2183	1056.4453	528.7263	G	2867.2800	1434.1436	2850.2535	1425.6304	2849.2694	1425.1384	32
14	1161.4879	581.2476	1144.4614	572.7343	1143.4774	572.2423	S	2810.2585	1405.6329	2793.2320	1397.1196	2792.2480	1396.6276	31
15	1232.5250	616.7662	1215.4985	608.2529	1214.5145	607.7609	A	2723.2265	1362.1169	2706.2000	1353.6036	2705.2159	1353.1116	30
16	1289.5465	645.2769	1272.5199	636.7636	1271.5359	636.2716	G	2652.1894	1326.5983	2635.1628	1318.0851	2634.1788	1317.5931	29
17	1417.6051	709.3062	1400.5785	700.7929	1399.5945	700.3009	Q	2595.1679	1298.0876	2578.1414	1289.5743	2577.1574	1289.0823	28
18	1580.6684	790.8378	1563.6419	782.3246	1562.6578	781.8326	Y	2467.1094	1234.0583	2450.0828	1225.5450	2449.0988	1225.0530	27

**19**	1637.6899	819.3486	1620.6633	810.8353	1619.6793	810.3433	**G**	2304.0460	1152.5267	2287.0195	1144.0134	2286.0355	1143.5214	**26**
**20**	1724.7219	862.8646	1707.6953	854.3513	1706.7113	853.8593	**S**	2247.0246	1124.0159	2229.9980	1115.5026	2229.0140	1115.0106	**25**
**21**	1839.7488	920.3781	1822.7223	911.8648	1821.7383	911.3728	**D**	2159.9925	1080.4999	2142.9660	1071.9866	2141.9820	1071.4946	**24**
**22**	1967.8074	984.4073	1950.7809	975.8941	1949.7969	975.4021	**Q**	2044.9656	1022.9864	2027.9390	1014.4732	2026.9550	1013.9812	**23**
**23**	2095.8660	1048.4366	2078.8394	1039.9234	2077.8554	1039.4314	**Q**	1916.9070	958.9571	1899.8805	950.4439	1898.8964	949.9519	**22**
**24**	2232.9249	1116.9661	2215.8984	1108.4528	2214.9143	1107.9608	**H**	1788.8484	894.9279	1771.8219	886.4146	1770.8379	885.9226	**21**
**25**	2369.9838	1185.4955	2352.9573	1176.9823	2351.9733	1176.4903	**H**	1651.7895	826.3984	1634.7630	817.8851	1633.7790	817.3931	**20**
**26**	2483.0679	1242.0376	2466.0413	1233.5243	2465.0573	1233.0323	**L**	1514.7306	757.8689	1497.7041	749.3557	1496.7200	748.8637	**19**
**27**	2540.0893	1270.5483	2523.0628	1262.0350	2522.0788	1261.5430	**G**	1401.6465	701.3269	1384.6200	692.8136	1383.6360	692.3216	**18**
**28**	2627.1214	1314.0643	2610.0948	1305.5511	2609.1108	1305.0590	**S**	1344.6251	672.8162	1327.5985	664.3029	1326.6145	663.8109	**17**
**29**	2684.1428	1342.5751	2667.1163	1334.0618	2666.1323	1333.5698	**G**	1257.5931	629.3002	1240.5665	620.7869	1239.5825	620.2949	**16**
**30**	2771.1749	1386.0911	2754.1483	1377.5778	2753.1643	1377.0858	**S**	1200.5716	600.7894	1183.5450	592.2762	1182.5610	591.7842	**15**
**31**	2828.1963	1414.6018	2811.1698	1406.0885	2810.1858	1405.5965	**G**	1113.5396	557.2734	1096.5130	548.7601	1095.5290	548.2681	**14**
**32**	2899.2334	1450.1204	2882.2069	1441.6071	2881.2229	1441.1151	**A**	1056.5181	528.7627	1039.4916	520.2494	1038.5075	519.7574	**13**
**33**	2956.2549	1478.6311	2939.2284	1470.1178	2938.2443	1469.6258	**G**	985.4810	493.2441	968.4544	484.7309	967.4704	484.2388	**12**
**34**	3013.2764	1507.1418	2996.2498	1498.6285	2995.2658	1498.1365	**G**	928.4595	464.7334	911.4330	456.2201	910.4490	455.7281	**11**
**35**	3114.3241	1557.6657	3097.2975	1549.1524	3096.3135	1548.6604	**T**	871.4381	436.2227	854.4115	427.7094	853.4275	427.2174	**10**
**36**	3171.3455	1586.1764	3154.3190	1577.6631	3153.3349	1577.1711	**G**	***770.3904***	385.6988	753.3638	377.1856			**9**
**37**	3228.3670	1614.6871	3211.3404	1606.1739	3210.3564	1605.6818	**G**	713.3689	357.1881	696.3424	348.6748			**8**
**38**	3325.4197	1663.2135	3308.3932	1654.7002	3307.4092	1654.2082	**P**	***656.3475***	328.6774	639.3209	320.1641			**7**
**39**	3396.4569	1698.7321	3379.4303	1690.2188	3378.4463	1689.7268	**A**	***559.2947***	280.1510	542.2681	271.6377			**6**
**40**	3453.4783	1727.2428	3436.4518	1718.7295	3435.4678	1718.2375	**G**	***488.2576***	244.6324	471.2310	236.1191			**5**
**41**	3581.5369	1791.2721	3564.5103	1782.7588	3563.5263	1782.2668	**Q**	431.2361	216.1217	414.2096	207.6084			**4**
**42**	3652.5740	1826.7906	3635.5475	1818.2774	3634.5634	1817.7854	**A**	303.1775	152.0924	286.1510	143.5791			**3**
**43**	3709.5955	1855.3014	3692.5689	1846.7881	3691.5849	1846.2961	**G**	232.1404	116.5738	215.1139	108.0606			**2**
**44**							**R**	175.1190	88.0631	158.0924	79.5498			**1**

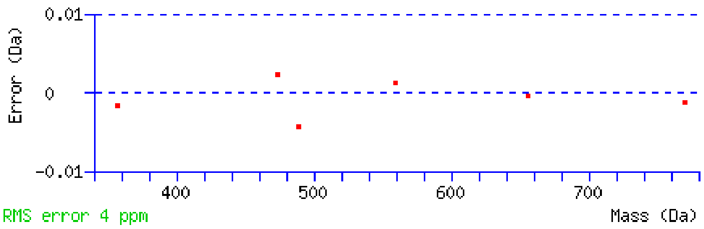

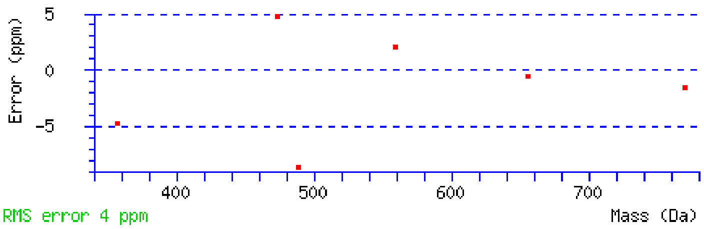

NCBI **BLAST** search of [SNGRGGSQAGGSGSAGQYGSDQQHHLGSGSAGGTGGPAGQAGR]
(Parameters: blastp, nr protein database, expect=20000, no filter, PAM30)
Other BLAST [web gateways]

All matches to this query

Score	Mr(calc)	Delta	Sequence
23.3	3882.6999	0.0035	SNGRGGSQAGGSGSAGQYGSDQQHHLGSGSAGGTGGPAGQAGR
16.6	3881.7159	0.9876	SNGRGGSQAGGSGSAGQYGSDQQHHLGSGSAGGTGGPAGQAGR
16.6	3882.6999	0.0035	SNGRGGSQAGGSGSAGQYGSDQQHHLGSGSAGGTGGPAGQAGR
16.6	3882.6999	0.0035	SNGRGGSQAGGSGSAGQYGSDQQHHLGSGSAGGTGGPAGQAGR
16.6	3882.6999	0.0035	SNGRGGSQAGGSGSAGQYGSDQQHHLGSGSAGGTGGPAGQAGR
16.6	3882.6999	0.0035	SNGRGGSQAGGSGSAGQYGSDQQHHLGSGSAGGTGGPAGQAGR

Mascot: http://www.matrixscience.com/

Mascot Search Results

Peptide View

MS/MS Fragmentation of **TLPNGRDALDGPAAEAEPEHSFDGLR**
Found in **Q15149** in **uni_human_nr**, PLEC_HUMAN Plectin OS=Homo sapiens GN=PLEC PE=1 SV=3

Match to Query 11159: 2735.278496 from(684.826900,4+) intensity(496650.0313) rtinseconds(2253) scans(12153) index(8764)
Title: 130801_HPL_Human_Plaque_BR1_TR1_13_Spectrum029234_scans__12153_RTINSECONDS=2253
Data file E:\\RAW\\HumanPlaques\\mgf\\T\\1D\\T130801_HPL_Human_Plaque_BR1_TR1_13.mgf

Click mouse within plot area to zoom in by factor of two about that point
Or, Plot from 0 to 2600 Da Full range
Label all possible matches ○ Label matches used for scoring ⦿
Show Y-axis ☐

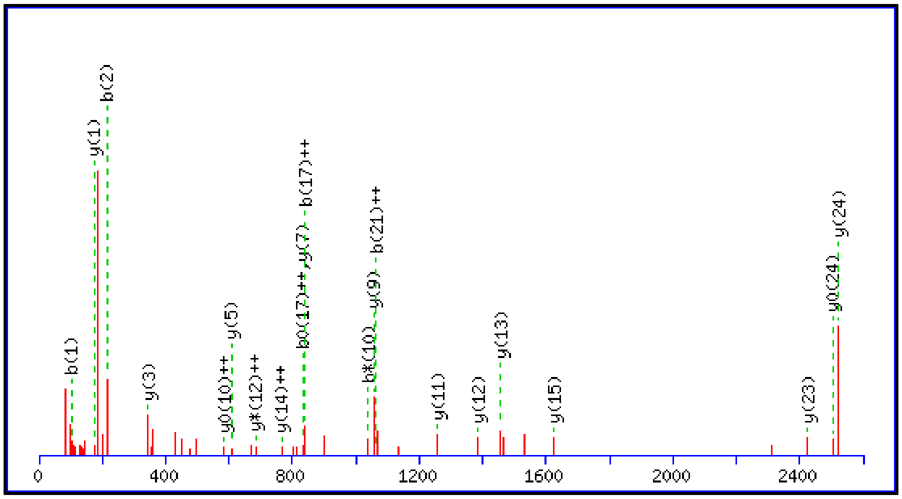

Monoisotopic mass of neutral peptide Mr(calc): 2735.2787
Fixed modifications: Carbamidomethyl (C) (apply to specified residues or termini only)
Variable modifications:
N4 : Deamidated (NQ)
Ions Score: 56 **Expect:** 0.00022
Matches : 21/288 fragment ions using 45 most intense peaks (help)

#	b	b^{++}	b*	b^{*++}	b^0	b^{0++}	Seq.	y	y^{++}	y*	y^{*++}	y^0	y^{0++}	#
1	**102.0550**	51.5311			84.0444	42.5258	T							26
2	**215.1390**	108.0731			197.1285	99.0679	L	2635.2383	1318.1228	2618.2118	1309.6095	2617.2277	1309.1175	25
3	312.1918	156.5995			294.1812	147.5942	P	***2522.1542***	1261.5808	2505.1277	1253.0675	2504.1437	1252.5755	24
4	427.2187	214.1130	410.1922	205.5997	409.2082	205.1077	N	***2425.1015***	1213.0544	2408.0749	1204.5411	2407.0909	1204.0491	23
5	484.2402	242.6237	467.2136	234.1105	466.2296	233.6185	G	2310.0745	1155.5409	2293.0480	1147.0276	2292.0640	1146.5356	22
6	640.3413	320.6743	623.3148	312.1610	622.3307	311.6690	R	2253.0531	1127.0302	2236.0265	1118.5169	2235.0425	1118.0249	21
7	755.3682	378.1878	738.3417	369.6745	737.3577	369.1825	D	2096.9520	1048.9796	2079.9254	1040.4663	2078.9414	1039.9743	20
8	826.4054	413.7063	809.3788	405.1930	808.3948	404.7010	A	1981.9250	991.4661	1964.8985	982.9529	1963.9144	982.4609	19
9	939.4894	470.2483	922.4629	461.7351	921.4789	461.2431	L	1910.8879	955.9476	1893.8613	947.4343	1892.8773	946.9423	18
10	1054.5164	527.7618	1037.4898	519.2485	1036.5058	518.7565	D	1797.8038	899.4056	1780.7773	890.8923	1779.7933	890.4003	17
11	1111.5378	556.2726	1094.5113	547.7593	1093.5273	547.2673	G	1682.7769	841.8921	1665.7503	833.3788	1664.7663	832.8868	16
12	1208.5906	604.7989	1191.5640	596.2857	1190.5800	595.7937	P	***1625.7554***	813.3814	1608.7289	804.8681	1607.7449	804.3761	15
13	1279.6277	640.3175	1262.6012	631.8042	1261.6171	631.3122	A	1528.7027	764.8550	1511.6761	756.3417	1510.6921	755.8497	14
14	1350.6648	675.8360	1333.6383	667.3228	1332.6543	666.8308	A	***1457.6655***	729.3364	1440.6390	720.8231	1439.6550	720.3311	13
15	1479.7074	740.3573	1462.6809	731.8441	1461.6968	731.3521	E	***1386.6284***	693.8179	1369.6019	685.3046	1368.6179	684.8126	12
16	1550.7445	775.8759	1533.7180	767.3626	1532.7340	766.8706	A	***1257.5858***	629.2966	1240.5593	620.7833	1239.5753	620.2913	11
17	1679.7871	**840.3972**	1662.7606	831.8839	1661.7766	831.3919	E	1186.5487	593.7780	1169.5222	585.2647	1168.5382	584.7727	10
18	1776.8399	888.9236	1759.8133	880.4103	1758.8293	879.9183	P	***1057.5061***	529.2567	1040.4796	520.7434	1039.4956	520.2514	9

**19**	1905.8825	953.4449	1888.8559	944.9316	1887.8719	944.4396	**E**	960.4534	480.7303	943.4268	472.2170	942.4428	471.7250	**8**
**20**	2042.9414	1021.9743	2025.9148	1013.4611	2024.9308	1012.9691	**H**	***831.4108***	416.2090	814.3842	407.6958	813.4002	407.2037	**7**
**21**	2129.9734	**1065.4903**	2112.9469	1056.9771	2111.9629	1056.4851	**S**	694.3519	347.6796	677.3253	339.1663	676.3413	338.6743	**6**
**22**	2277.0418	1139.0246	2260.0153	1130.5113	2259.0313	1130.0193	**F**	***607.3198***	304.1636	590.2933	295.6503	589.3093	295.1583	**5**
**23**	2392.0688	1196.5380	2375.0422	1188.0248	2374.0582	1187.5327	**D**	460.2514	230.6293	443.2249	222.1161	442.2409	221.6241	**4**
**24**	2449.0902	1225.0488	2432.0637	1216.5355	2431.0797	1216.0435	**G**	***345.2245***	173.1159	328.1979	164.6026			**3**
**25**	2562.1743	1281.5908	2545.1478	1273.0775	2544.1637	1272.5855	**L**	288.2030	144.6051	271.1765	136.0919			**2**
**26**							**R**	***175.1190***	88.0631	158.0924	79.5498			**1**

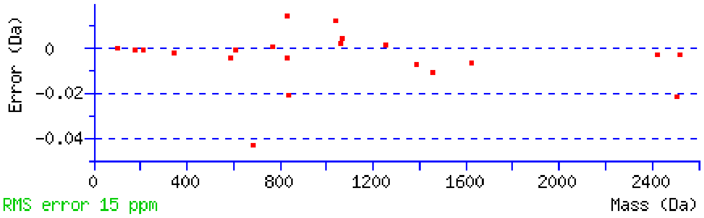

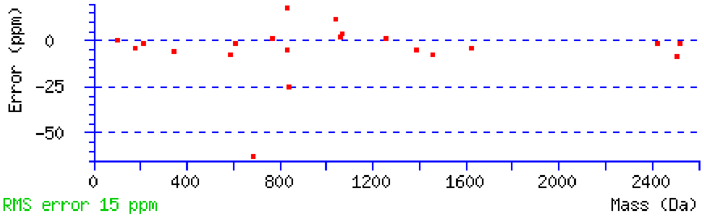

NCBI **BLAST** search of TLPNGRDALDGPAAEAEPEHSFDGLR
(Parameters: blastp, nr protein database, expect=20000, no filter, PAM30)
Other BLAST web gateways

All matches to this query

Score	Mr(calc)	Delta	Sequence
55.6	2735.2787	-0.0002	TLPNGRDALDGPAAEAEPEHSFDGLR
0.1	2733.2599	2.0186	VSGGQAHEDLLNESIKACQRGFCR

**Mascot:** http://www.matrixscience.com/

Mascot Search Results

Peptide View

MS/MS Fragmentation of **TLPNGRDALDGPAAEAEPEHSFDGLR**
Found in **Q15149** in **uni_human_nr**, PLEC_HUMAN Plectin OS=Homo sapiens GN=PLEC PE=1 SV=3

Match to Query 12788: 2734.295856 from(684.581240,4+) intensity(350750.9375) rtinseconds(2111) scans(11896) index(8681)
Title: 130806_HPL_Human_Plaque_BR1_TR2_08_Spectrum030373_scans__11896_RTINSECONDS=2111
Data file E:\\RAW\\HumanPlaques\\mgf\\T\\1D\\T130806_HPL_Human_Plaque_BR1_TR2_08.mgf

Click mouse within plot area to zoom in by factor of two about that point
Or, Plot from 0 to 2600 Da Full range
Label all possible matches ○ Label matches used for scoring ⦿
Show Y-axis ☐

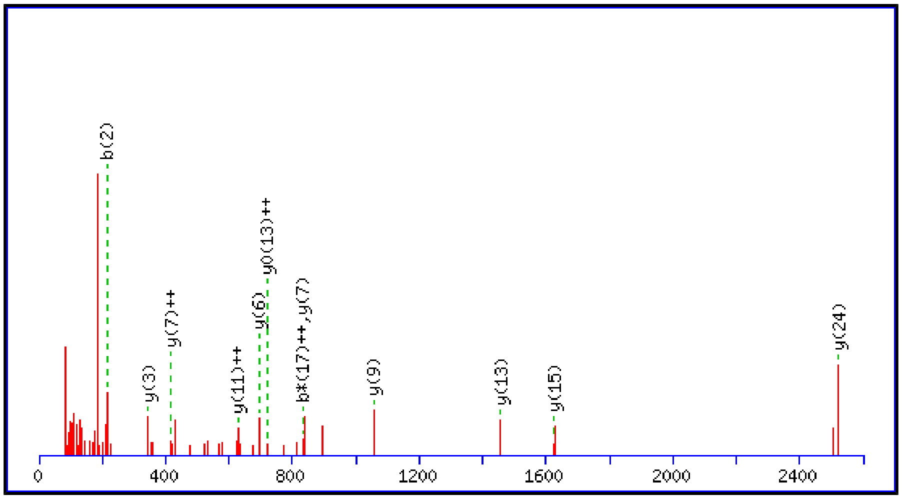

Monoisotopic mass of neutral peptide Mr(calc): 2734.2947
Fixed modifications: Carbamidomethyl (C) (apply to specified residues or termini only)
Ions Score: 30 **Expect:** 0.082
Matches : 12/288 fragment ions using 24 most intense peaks (help)

#	b	b^{++}	b*	b^{*++}	b^0	b^{0++}	Seq.	y	y^{++}	y*	y^{*++}	y^0	y^{0++}	#
1	102.0550	51.5311			84.0444	42.5258	T							26
2	**215.1390**	108.0731			197.1285	99.0679	L	2634.2543	1317.6308	2617.2277	1309.1175	2616.2437	1308.6255	25
3	312.1918	156.5995			294.1812	147.5942	P	***2521.1702***	1261.0887	2504.1437	1252.5755	2503.1597	1252.0835	24
4	426.2347	213.6210	409.2082	205.1077	408.2241	204.6157	N	2424.1175	1212.5624	2407.0909	1204.0491	2406.1069	1203.5571	23
5	483.2562	242.1317	466.2296	233.6185	465.2456	233.1264	G	2310.0745	1155.5409	2293.0480	1147.0276	2292.0640	1146.5356	22
6	639.3573	320.1823	622.3307	311.6690	621.3467	311.1770	R	2253.0531	1127.0302	2236.0265	1118.5169	2235.0425	1118.0249	21
7	754.3842	377.6958	737.3577	369.1825	736.3737	368.6905	D	2096.9520	1048.9796	2079.9254	1040.4663	2078.9414	1039.9743	20
8	825.4213	413.2143	808.3948	404.7010	807.4108	404.2090	A	1981.9250	991.4661	1964.8985	982.9529	1963.9144	982.4609	19
9	938.5054	469.7563	921.4789	461.2431	920.4948	460.7511	L	1910.8879	955.9476	1893.8613	947.4343	1892.8773	946.9423	18
10	1053.5323	527.2698	1036.5058	518.7565	1035.5218	518.2645	D	1797.8038	899.4056	1780.7773	890.8923	1779.7933	890.4003	17
11	1110.5538	555.7805	1093.5273	547.2673	1092.5432	546.7753	G	1682.7769	841.8921	1665.7503	833.3788	1664.7663	832.8868	16
12	1207.6066	604.3069	1190.5800	595.7937	1189.5960	595.3016	P	***1625.7554***	813.3814	1608.7289	804.8681	1607.7449	804.3761	15
13	1278.6437	639.8255	1261.6171	631.3122	1260.6331	630.8202	A	1528.7027	764.8550	1511.6761	756.3417	1510.6921	755.8497	14
14	1349.6808	675.3440	1332.6543	666.8308	1331.6702	666.3388	A	***1457.6655***	729.3364	1440.6390	720.8231	1439.6550	720.3311	13
15	1478.7234	739.8653	1461.6968	731.3521	1460.7128	730.8601	E	1386.6284	693.8179	1369.6019	685.3046	1368.6179	684.8126	12
16	1549.7605	775.3839	1532.7340	766.8706	1531.7499	766.3786	A	1257.5858	629.2966	1240.5593	620.7833	1239.5753	620.2913	11
17	1678.8031	839.9052	1661.7766	831.3919	1660.7925	830.8999	E	1186.5487	593.7780	1169.5222	585.2647	1168.5382	584.7727	10
18	1775.8559	888.4316	1758.8293	879.9183	1757.8453	879.4263	P	***1057.5061***	529.2567	1040.4796	520.7434	1039.4956	520.2514	9
19	1904.8985	952.9529	1887.8719	944.4396	1886.8879	943.9476	E	960.4534	480.7303	943.4268	472.2170	942.4428	471.7250	8

**20**	2041.9574	1021.4823	2024.9308	1012.9691	2023.9468	1012.4770	**H**	***831.4108***	416.2090	814.3842	407.6958	813.4002	407.2037	**7**
**21**	2128.9894	1064.9983	2111.9629	1056.4851	2110.9788	1055.9931	**S**	***694.3519***	347.6796	677.3253	339.1663	676.3413	338.6743	**6**
**22**	2276.0578	1138.5325	2259.0313	1130.0193	2258.0473	1129.5273	**F**	607.3198	304.1636	590.2933	295.6503	589.3093	295.1583	**5**
**23**	2391.0848	1196.0460	2374.0582	1187.5327	2373.0742	1187.0407	**D**	460.2514	230.6293	443.2249	222.1161	442.2409	221.6241	**4**
**24**	2448.1062	1224.5567	2431.0797	1216.0435	2430.0957	1215.5515	**G**	***345.2245***	173.1159	328.1979	164.6026			**3**
**25**	2561.1903	1281.0988	2544.1637	1272.5855	2543.1797	1272.0935	**L**	288.2030	144.6051	271.1765	136.0919			**2**
**26**							**R**	175.1190	88.0631	158.0924	79.5498			**1**

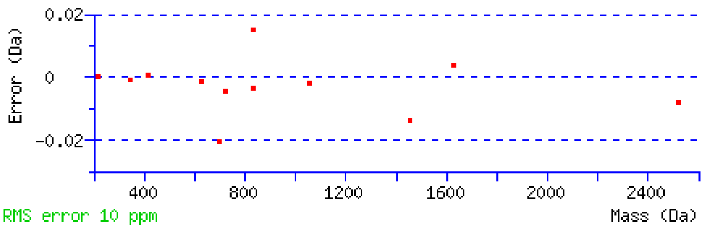

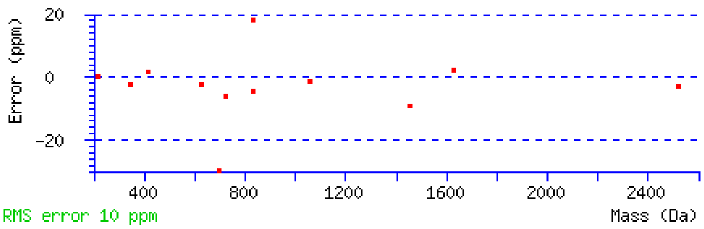

NCBI **BLAST** search of TLPNGRDALDGPAAEAEPEHSFDGLR
(Parameters: blastp, nr protein database, expect=20000, no filter, PAM30)
Other BLAST web gateways

All matches to this query

Score	Mr(calc)	Delta	Sequence
30.1	2734.2947	0.0012	TLPNGRDALDGPAAEAEPEHSFDGLR

Mascot: http://www.matrixscience.com/

Mascot Search Results

Peptide View

MS/MS Fragmentation of **TLPNGRDALDGPAAEAEPEHSFDGLRR**
Found in **Q15149** in **uni_human_nr**, PLEC_HUMAN Plectin OS=Homo sapiens GN=PLEC PE=1 SV=3

Match to Query 14558: 2891.380870 from(579.283450,5+) intensity(996279.5625) rtinseconds(2049) scans(11466) index(8505)
Title: 130806_HPL_Human_Plaque_BR2_TR2_07_Spectrum030181_scans__11466_RTINSECONDS=2049
Data file E:\\RAW\\HumanPlaques\\mgf\\T\\1D\\T130806_HPL_Human_Plaque_BR2_TR2_07.mgf

Click mouse within plot area to zoom in by factor of two about that point
Or, Plot from 0 to 2800 Da Full range
Label all possible matches ○ Label matches used for scoring ⦿
Show Y-axis ☐

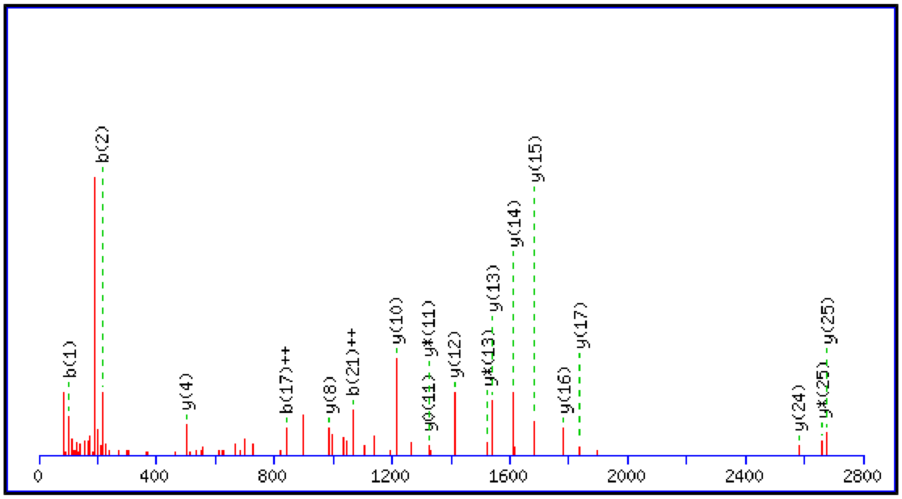

Monoisotopic mass of neutral peptide Mr(calc): 2891.3798
Fixed modifications: Carbamidomethyl (C) (apply to specified residues or termini only)
Variable modifications:
N4 : Deamidated (NQ)
Ions Score: 62 **Expect:** 5.1e-005
Matches : 19/298 fragment ions using 38 most intense peaks (help)

#	b	b++	b*	b*++	b0	b0++	Seq.	y	y++	y*	y*++	y0	y0++	#
1	**102.0550**	51.5311			84.0444	42.5258	T							27
2	**215.1390**	108.0731			197.1285	99.0679	L	2791.3394	1396.1733	2774.3129	1387.6601	2773.3288	1387.1681	26
3	312.1918	156.5995			294.1812	147.5942	P	***2678.2553***	1339.6313	2661.2288	1331.1180	2660.2448	1330.6260	25
4	427.2187	214.1130	410.1922	205.5997	409.2082	205.1077	N	***2581.2026***	1291.1049	2564.1760	1282.5917	2563.1920	1282.0996	24
5	484.2402	242.6237	467.2136	234.1105	466.2296	233.6185	G	2466.1756	1233.5915	2449.1491	1225.0782	2448.1651	1224.5862	23
6	640.3413	320.6743	623.3148	312.1610	622.3307	311.6690	R	2409.1542	1205.0807	2392.1276	1196.5675	2391.1436	1196.0754	22
7	755.3682	378.1878	738.3417	369.6745	737.3577	369.1825	D	2253.0531	1127.0302	2236.0265	1118.5169	2235.0425	1118.0249	21
8	826.4054	413.7063	809.3788	405.1930	808.3948	404.7010	A	2138.0261	1069.5167	2120.9996	1061.0034	2120.0156	1060.5114	20
9	939.4894	470.2483	922.4629	461.7351	921.4789	461.2431	L	2066.9890	1033.9981	2049.9625	1025.4849	2048.9784	1024.9929	19
10	1054.5164	527.7618	1037.4898	519.2485	1036.5058	518.7565	D	1953.9049	977.4561	1936.8784	968.9428	1935.8944	968.4508	18
11	1111.5378	556.2726	1094.5113	547.7593	1093.5273	547.2673	G	***1838.8780***	919.9426	1821.8515	911.4294	1820.8674	910.9374	17
12	1208.5906	604.7989	1191.5640	596.2857	1190.5800	595.7937	P	***1781.8565***	891.4319	1764.8300	882.9186	1763.8460	882.4266	16
13	1279.6277	640.3175	1262.6012	631.8042	1261.6171	631.3122	A	***1684.8038***	842.9055	1667.7772	834.3922	1666.7932	833.9002	15
14	1350.6648	675.8360	1333.6383	667.3228	1332.6543	666.8308	A	***1613.7667***	807.3870	1596.7401	798.8737	1595.7561	798.3817	14
15	1479.7074	740.3573	1462.6809	731.8441	1461.6968	731.3521	E	***1542.7295***	771.8684	1525.7030	763.3551	1524.7190	762.8631	13
16	1550.7445	775.8759	1533.7180	767.3626	1532.7340	766.8706	A	***1413.6870***	707.3471	1396.6604	698.8338	1395.6764	698.3418	12
17	1679.7871	**840.3972**	1662.7606	831.8839	1661.7766	831.3919	E	1342.6498	671.8286	1325.6233	663.3153	1324.6393	662.8233	11
18	1776.8399	888.9236	1759.8133	880.4103	1758.8293	879.9183	P	***1213.6072***	607.3073	1196.5807	598.7940	1195.5967	598.3020	10

**19**	1905.8825	953.4449	1888.8559	944.9316	1887.8719	944.4396	**E**	1116.5545	558.7809	1099.5279	550.2676	1098.5439	549.7756	**9**
**20**	2042.9414	1021.9743	2025.9148	1013.4611	2024.9308	1012.9691	**H**	***987.5119***	494.2596	970.4853	485.7463	969.5013	485.2543	**8**
**21**	2129.9734	**1065.4903**	2112.9469	1056.9771	2111.9629	1056.4851	**S**	850.4530	425.7301	833.4264	417.2169	832.4424	416.7248	**7**
**22**	2277.0418	1139.0246	2260.0153	1130.5113	2259.0313	1130.0193	**F**	763.4209	382.2141	746.3944	373.7008	745.4104	373.2088	**6**
**23**	2392.0688	1196.5380	2375.0422	1188.0248	2374.0582	1187.5327	**D**	616.3525	308.6799	599.3260	300.1666	598.3420	299.6746	**5**
**24**	2449.0902	1225.0488	2432.0637	1216.5355	2431.0797	1216.0435	**G**	***501.3256***	251.1664	484.2990	242.6532			**4**
**25**	2562.1743	1281.5908	2545.1478	1273.0775	2544.1637	1272.5855	**L**	444.3041	222.6557	427.2776	214.1424			**3**
**26**	2718.2754	1359.6413	2701.2489	1351.1281	2700.2648	1350.6361	**R**	331.2201	166.1137	314.1935	157.6004			**2**
**27**							**R**	175.1190	88.0631	158.0924	79.5498			**1**

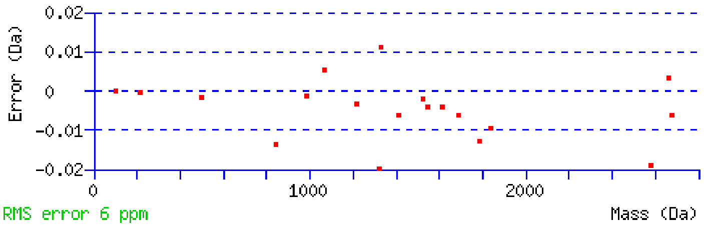

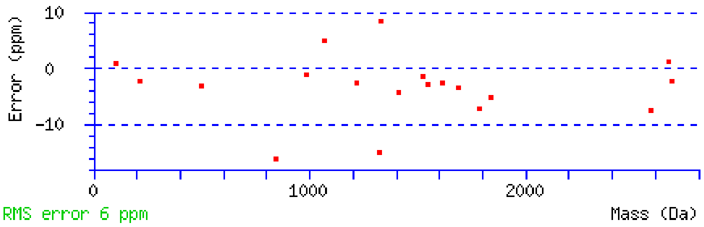

NCBI **BLAST** search of TLPNGRDALDGPAAEAEPEHSFDGLRR
(Parameters: blastp, nr protein database, expect=20000, no filter, PAM30)
Other BLAST web gateways

All matches to this query

Score	Mr(calc)	Delta	Sequence
62.4	2891.3798	0.0011	TLPNGRDALDGPAAEAEPEHSFDGLRR

Mascot: http://www.matrixscience.com/

Mascot Search Results

Peptide View

MS/MS Fragmentation of **TLPNGRDALDGPAAEAEPEHSFDGLRR**
Found in **Q15149** in **uni_human_nr**, PLEC_HUMAN Plectin OS=Homo sapiens GN=PLEC PE=1 SV=3

Match to Query 14184: 2891.380056 from(723.852290,4+) intensity(537516.7500) rtinseconds(2028) scans(11319) index(8233)
Title: 130801_HPL_Human_Plaque_BR1_TR1_07_Spectrum029936_scans__11319_RTINSECONDS=2028
Data file E:\\RAW\\HumanPlaques\\mgf\\T\\1D\\T130801_HPL_Human_Plaque_BR1_TR1_07.mgf

Click mouse within plot area to zoom in by factor of two about that point
Or, Plot from 0 to 2800 Da Full range
Label all possible matches ○ Label matches used for scoring ⊙
Show Y-axis ☐

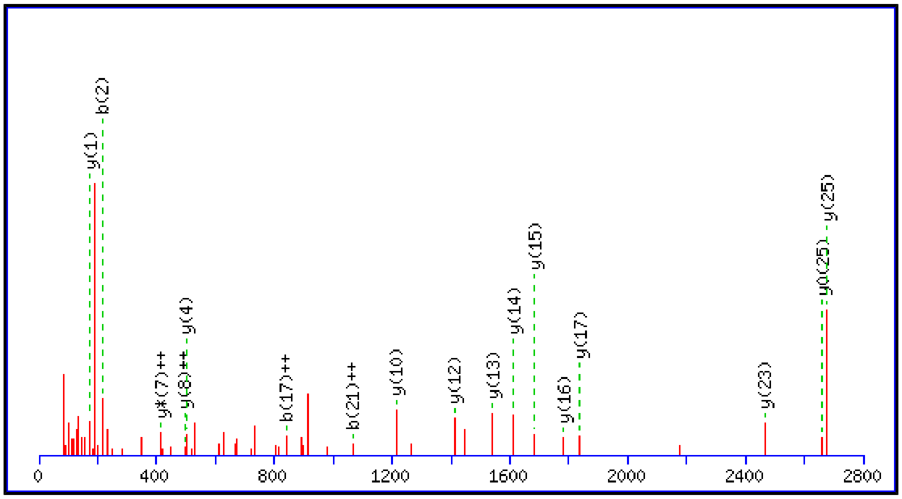

Monoisotopic mass of neutral peptide Mr(calc): 2891.3798
Fixed modifications: Carbamidomethyl (C) (apply to specified residues or termini only)
Variable modifications:
N4 : Deamidated (NQ)
Ions Score: 51 **Expect:** 0.00073
Matches : 17/298 fragment ions using 39 most intense peaks (help)

#	b	b^{++}	b*	b*$^{++}$	b^{0}	b^{0++}	Seq.	y	y^{++}	y*	y*$^{++}$	y^{0}	y^{0++}	#
1	102.0550	51.5311			84.0444	42.5258	T							27
2	**215.1390**	108.0731			197.1285	99.0679	L	2791.3394	1396.1733	2774.3129	1387.6601	2773.3288	1387.1681	26
3	312.1918	156.5995			294.1812	147.5942	P	***2678.2553***	1339.6313	2661.2288	1331.1180	2660.2448	1330.6260	25
4	427.2187	214.1130	410.1922	205.5997	409.2082	205.1077	N	2581.2026	1291.1049	2564.1760	1282.5917	2563.1920	1282.0996	24
5	484.2402	242.6237	467.2136	234.1105	466.2296	233.6185	G	***2466.1756***	1233.5915	2449.1491	1225.0782	2448.1651	1224.5862	23
6	640.3413	320.6743	623.3148	312.1610	622.3307	311.6690	R	2409.1542	1205.0807	2392.1276	1196.5675	2391.1436	1196.0754	22
7	755.3682	378.1878	738.3417	369.6745	737.3577	369.1825	D	2253.0531	1127.0302	2236.0265	1118.5169	2235.0425	1118.0249	21
8	826.4054	413.7063	809.3788	405.1930	808.3948	404.7010	A	2138.0261	1069.5167	2120.9996	1061.0034	2120.0156	1060.5114	20
9	939.4894	470.2483	922.4629	461.7351	921.4789	461.2431	L	2066.9890	1033.9981	2049.9625	1025.4849	2048.9784	1024.9929	19
10	1054.5164	527.7618	1037.4898	519.2485	1036.5058	518.7565	D	1953.9049	977.4561	1936.8784	968.9428	1935.8944	968.4508	18
11	1111.5378	556.2726	1094.5113	547.7593	1093.5273	547.2673	G	***1838.8780***	919.9426	1821.8515	911.4294	1820.8674	910.9374	17
12	1208.5906	604.7989	1191.5640	596.2857	1190.5800	595.7937	P	***1781.8565***	891.4319	1764.8300	882.9186	1763.8460	882.4266	16
13	1279.6277	640.3175	1262.6012	631.8042	1261.6171	631.3122	A	***1684.8038***	842.9055	1667.7772	834.3922	1666.7932	833.9002	15
14	1350.6648	675.8360	1333.6383	667.3228	1332.6543	666.8308	A	***1613.7667***	807.3870	1596.7401	798.8737	1595.7561	798.3817	14
15	1479.7074	740.3573	1462.6809	731.8441	1461.6968	731.3521	E	***1542.7295***	771.8684	1525.7030	763.3551	1524.7190	762.8631	13
16	1550.7445	775.8759	1533.7180	767.3626	1532.7340	766.8706	A	***1413.6870***	707.3471	1396.6604	698.8338	1395.6764	698.3418	12
17	1679.7871	**840.3972**	1662.7606	831.8839	1661.7766	831.3919	E	1342.6498	671.8286	1325.6233	663.3153	1324.6393	662.8233	11
18	1776.8399	888.9236	1759.8133	880.4103	1758.8293	879.9183	P	***1213.6072***	607.3073	1196.5807	598.7940	1195.5967	598.3020	10

**19**	1905.8825	953.4449	1888.8559	944.9316	1887.8719	944.4396	**E**	1116.5545	558.7809	1099.5279	550.2676	1098.5439	549.7756	**9**
**20**	2042.9414	1021.9743	2025.9148	1013.4611	2024.9308	1012.9691	**H**	987.5119	494.2596	970.4853	485.7463	969.5013	485.2543	**8**
**21**	2129.9734	**1065.4903**	2112.9469	1056.9771	2111.9629	1056.4851	**S**	850.4530	425.7301	833.4264	417.2169	832.4424	416.7248	**7**
**22**	2277.0418	1139.0246	2260.0153	1130.5113	2259.0313	1130.0193	**F**	763.4209	382.2141	746.3944	373.7008	745.4104	373.2088	**6**
**23**	2392.0688	1196.5380	2375.0422	1188.0248	2374.0582	1187.5327	**D**	616.3525	308.6799	599.3260	300.1666	598.3420	299.6746	**5**
**24**	2449.0902	1225.0488	2432.0637	1216.5355	2431.0797	1216.0435	**G**	***501.3256***	251.1664	484.2990	242.6532			**4**
**25**	2562.1743	1281.5908	2545.1478	1273.0775	2544.1637	1272.5855	**L**	444.3041	222.6557	427.2776	214.1424			**3**
**26**	2718.2754	1359.6413	2701.2489	1351.1281	2700.2648	1350.6361	**R**	331.2201	166.1137	314.1935	157.6004			**2**
**27**							**R**	***175.1190***	88.0631	158.0924	79.5498			**1**

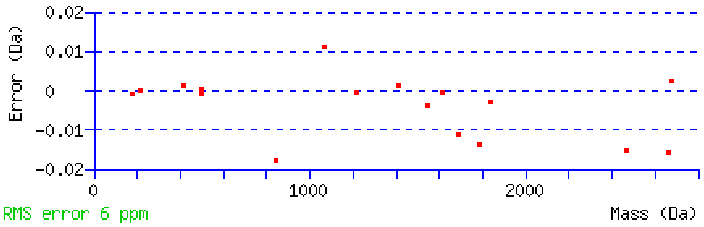

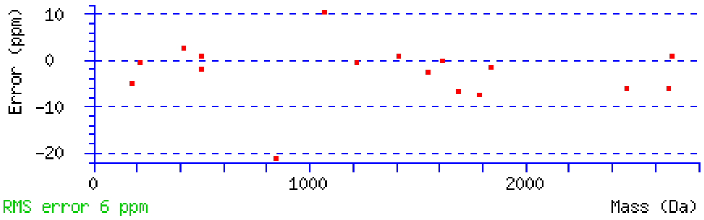

NCBI **BLAST** search of TLPNGRDALDGPAAEAEPEHSFDGLRR
(Parameters: blastp, nr protein database, expect=20000, no filter, PAM30)
Other BLAST web gateways

All matches to this query

Score	Mr(calc)	Delta	Sequence
50.9	2891.3798	0.0003	TLPNGRDALDGPAAEAEPEHSFDGLRR
0.3	2891.3771	0.0030	KGILENQGGKGQNTLSSDVQTNEDILK

Mascot: http://www.matrixscience.com/

Mascot Search Results

Peptide View

MS/MS Fragmentation of **GCVVTSVESNSNGR**
Found in **P08567** in **uni_human_nr**, PLEK_HUMAN Pleckstrin OS=Homo sapiens GN=PLEK PE=1 SV=3

Match to Query 2811: 1465.650008 from(733.832280,2+) intensity(106508.4609) rtinseconds(1170) scans(4976) index(2857)
Title: 130809_HPL_Human_Plaque_BR1_TR3_16_Spectrum022829_scans__4976_RTINSECONDS=1170
Data file E:\\RAW\\HumanPlaques\\mgf\\T\\1D\\T130809_HPL_Human_Plaque_BR1_TR3_16.mgf

Click mouse within plot area to zoom in by factor of two about that point
Or, Plot from 100 to 1100 Da Full range
Label all possible matches ○ Label matches used for scoring ⊙
Show Y-axis ☐

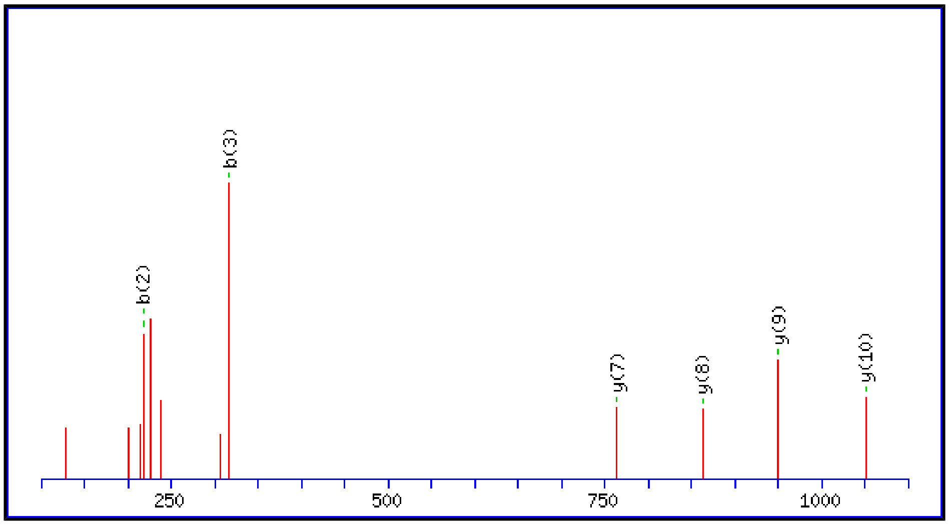

Monoisotopic mass of neutral peptide Mr(calc): 1465.6467
Fixed modifications: Carbamidomethyl (C) (apply to specified residues or termini only)
Variable modifications:
N12 : Deamidated (NQ)
Ions Score: 31 **Expect:** 0.031
Matches : 6/124 fragment ions using 8 most intense peaks (help)

#	b	b++	b*	b*++	b0	b0++	Seq.	y	y++	y*	y*++	y0	y0++	#
1	58.0287	29.5180					G							14
2	***218.0594***	109.5333					C	1409.6325	705.3199	1392.6060	696.8066	1391.6220	696.3146	13
3	***317.1278***	159.0675					V	1249.6019	625.3046	1232.5753	616.7913	1231.5913	616.2993	12
4	416.1962	208.6017					V	1150.5335	575.7704	1133.5069	567.2571	1132.5229	566.7651	11
5	517.2439	259.1256			499.2333	250.1203	T	***1051.4651***	526.2362	1034.4385	517.7229	1033.4545	517.2309	10
6	604.2759	302.6416			586.2654	293.6363	S	***950.4174***	475.7123	933.3908	467.1991	932.4068	466.7070	9
7	703.3443	352.1758			685.3338	343.1705	V	***863.3853***	432.1963	846.3588	423.6830	845.3748	423.1910	8
8	832.3869	416.6971			814.3764	407.6918	E	***764.3169***	382.6621	747.2904	374.1488	746.3064	373.6568	7
9	919.4190	460.2131			901.4084	451.2078	S	635.2743	318.1408	618.2478	309.6275	617.2638	309.1355	6
10	1033.4619	517.2346	1016.4353	508.7213	1015.4513	508.2293	N	548.2423	274.6248	531.2158	266.1115	530.2317	265.6195	5
11	1120.4939	560.7506	1103.4674	552.2373	1102.4833	551.7453	S	434.1994	217.6033	417.1728	209.0901	416.1888	208.5980	4
12	1235.5209	618.2641	1218.4943	609.7508	1217.5103	609.2588	N	347.1674	174.0873	330.1408	165.5740			3
13	1292.5423	646.7748	1275.5158	638.2615	1274.5318	637.7695	G	232.1404	116.5738	215.1139	108.0606			2
14							R	175.1190	88.0631	158.0924	79.5498			1

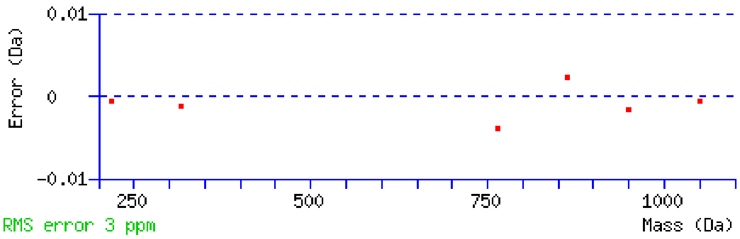

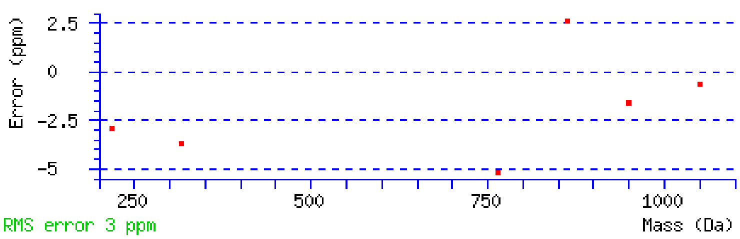

NCBI **BLAST** search of GCVVTSVESNSNGR
(Parameters: blastp, nr protein database, expect=20000, no filter, PAM30)
Other BLAST web gateways

All matches to this query

Score	Mr(calc)	Delta	Sequence	Site Analysis
30.6	1465.6467	0.0033	GCVVTSVESNSNGR	Deamidated N12 50.00%
30.6	1465.6467	0.0033	GCVVTSVESNSNGR	Deamidated N10 50.00%

Mascot: http://www.matrixscience.com/

Mascot Search Results

Peptide View

MS/MS Fragmentation of **GCVVTSVESNSNGR**
Found in **P08567** in **uni_human_nr**, PLEK_HUMAN Pleckstrin OS=Homo sapiens GN=PLEK PE=1 SV=3

Match to Query 3691: 1464.664648 from(733.339600,2+) intensity(897571.6875) rtinseconds(1066) scans(4722) index(2914)
Title: 130806_HPL_Human_Plaque_BR2_TR2_04_Spectrum025927_scans__4722_RTINSECONDS=1066
Data file E:\\RAW\\HumanPlaques\\mgf\\T\\1D\\T130806_HPL_Human_Plaque_BR2_TR2_04.mgf

Click mouse within plot area to zoom in by factor of two about that point
Or, Plot from 0 to 1500 Da Full range
Label all possible matches ○ Label matches used for scoring ⦿
Show Y-axis ☐

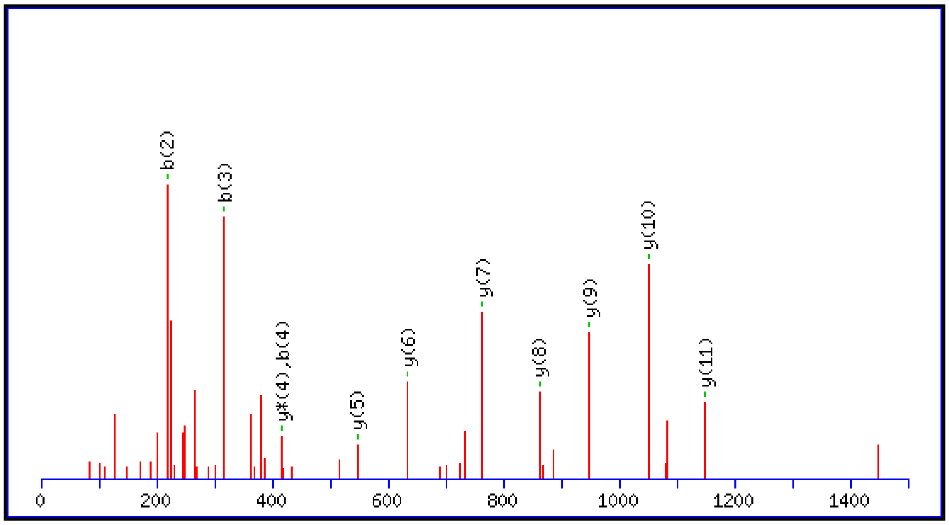

Monoisotopic mass of neutral peptide Mr(calc): 1464.6627
Fixed modifications: Carbamidomethyl (C) (apply to specified residues or termini only)
Ions Score: 66 **Expect:** 1e-005
Matches : 11/124 fragment ions using 12 most intense peaks (help)

#	b	b^{++}	b*	b^{*++}	b^0	b^{0++}	Seq.	y	y^{++}	y*	y^{*++}	y^0	y^{0++}	#
1	58.0287	29.5180					G							14
2	**218.0594**	109.5333					C	1408.6485	704.8279	1391.6220	696.3146	1390.6380	695.8226	13
3	**317.1278**	159.0675					V	1248.6179	624.8126	1231.5913	616.2993	1230.6073	615.8073	12
4	**416.1962**	208.6017					V	***1149.5495***	575.2784	1132.5229	566.7651	1131.5389	566.2731	11
5	517.2439	259.1256			499.2333	250.1203	T	***1050.4810***	525.7442	1033.4545	517.2309	1032.4705	516.7389	10
6	604.2759	302.6416			586.2654	293.6363	S	***949.4334***	475.2203	932.4068	466.7070	931.4228	466.2150	9
7	703.3443	352.1758			685.3338	343.1705	V	***862.4013***	431.7043	845.3748	423.1910	844.3908	422.6990	8
8	832.3869	416.6971			814.3764	407.6918	E	***763.3329***	382.1701	746.3064	373.6568	745.3224	373.1648	7
9	919.4190	460.2131			901.4084	451.2078	S	***634.2903***	317.6488	617.2638	309.1355	616.2798	308.6435	6
10	1033.4619	517.2346	1016.4353	508.7213	1015.4513	508.2293	N	***547.2583***	274.1328	530.2317	265.6195	529.2477	265.1275	5
11	1120.4939	560.7506	1103.4674	552.2373	1102.4833	551.7453	S	433.2154	217.1113	416.1888	208.5980	415.2048	208.1060	4
12	1234.5368	617.7721	1217.5103	609.2588	1216.5263	608.7668	N	346.1833	173.5953	329.1568	165.0820			3
13	1291.5583	646.2828	1274.5318	637.7695	1273.5477	637.2775	G	232.1404	116.5738	215.1139	108.0606			2
14							R	175.1190	88.0631	158.0924	79.5498			1

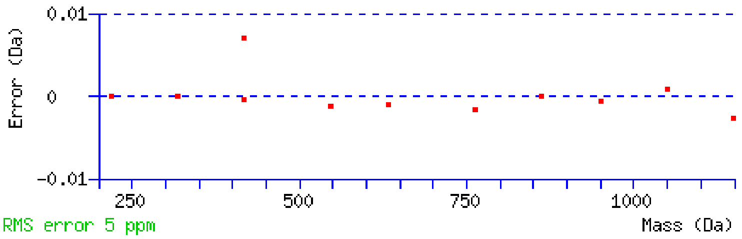

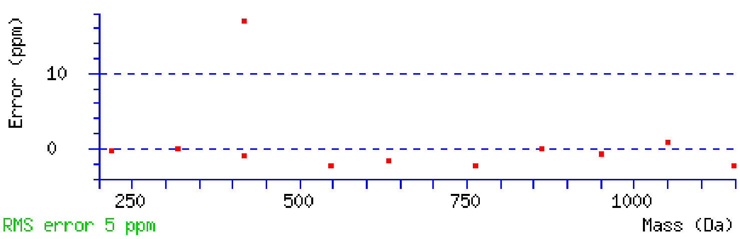

NCBI **BLAST** search of GCVVTSVESNSNGR
(Parameters: blastp, nr protein database, expect=20000, no filter, PAM30)
Other BLAST web gateways

All matches to this query

Score	Mr(calc)	Delta	Sequence
66.2	1464.6627	0.0019	GCVVTSVESNSNGR
3.0	1463.6675	0.9972	DSLETGVGDQCRK
3.0	1463.6674	0.9972	NSLETGVGDQCRK
1.2	1462.6609	2.0037	KDENKVDGMNAPK

Mascot: http://www.matrixscience.com/

Mascot Search Results

Peptide View

MS/MS Fragmentation of **GCVVTSVESNSNGRK**
Found in **P08567** in **uni_human_nr**, PLEK_HUMAN Pleckstrin OS=Homo sapiens GN=PLEK PE=1 SV=3

Match to Query 4548: 1594.725912 from(532.582580,3+) intensity(115125.6719) rtinseconds(972) scans(3748) index(1853)
Title: 130809_HPL_Human_Plaque_BR2_TR3_15_Spectrum022008_scans__3748_RTINSECONDS=972
Data file E:\\RAW\\HumanPlaques\\mgf\\T\\1D\\T130809_HPL_Human_Plaque_BR2_TR3_15.mgf

Click mouse within plot area to zoom in by factor of two about that point
Or, Plot from 0 to 1200 Da Full range
Label all possible matches ○ Label matches used for scoring ⦿
Show Y-axis ☐

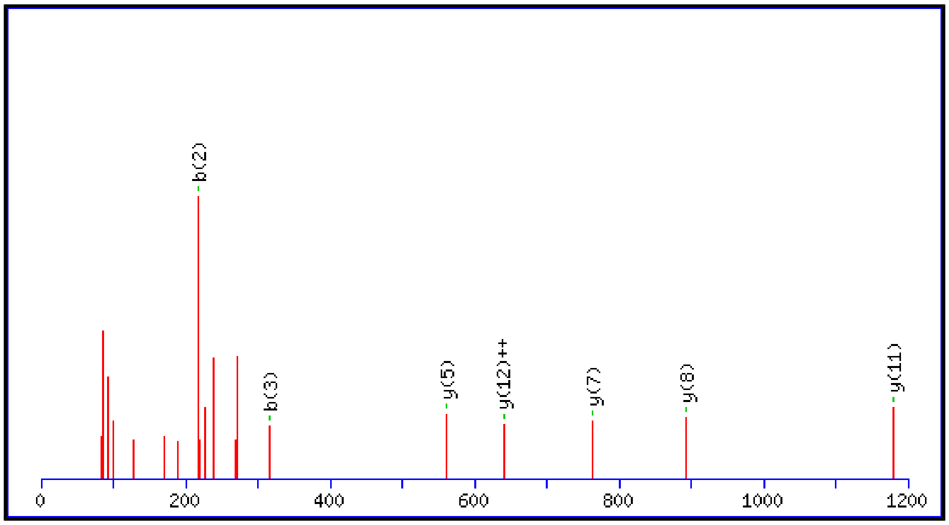

Monoisotopic mass of neutral peptide Mr(calc): 1594.7257
Fixed modifications: Carbamidomethyl (C) (apply to specified residues or termini only)
Variable modifications:
N10 : Deamidated (NQ)
N12 : Deamidated (NQ)
Ions Score: 32 **Expect:** 0.034
Matches : 7/134 fragment ions using 8 most intense peaks (help)

#	b	b^{++}	b^{*}	b^{*++}	b^{0}	b^{0++}	Seq.	y	y^{++}	y^{*}	y^{*++}	y^{0}	y^{0++}	#
1	58.0287	29.5180					G							15
2	***218.0594***	109.5333					C	1538.7115	769.8594	1521.6850	761.3461	1520.7009	760.8541	14
3	***317.1278***	159.0675					V	1378.6809	689.8441	1361.6543	681.3308	1360.6703	680.8388	13
4	416.1962	208.6017					V	1279.6124	640.3099	1262.5859	631.7966	1261.6019	631.3046	12
5	517.2439	259.1256			499.2333	250.1203	T	***1180.5440***	590.7757	1163.5175	582.2624	1162.5335	581.7704	11
6	604.2759	302.6416			586.2654	293.6363	S	1079.4964	540.2518	1062.4698	531.7385	1061.4858	531.2465	10
7	703.3443	352.1758			685.3338	343.1705	V	992.4643	496.7358	975.4378	488.2225	974.4538	487.7305	9
8	832.3869	416.6971			814.3764	407.6918	E	***893.3959***	447.2016	876.3694	438.6883	875.3853	438.1963	8
9	919.4190	460.2131			901.4084	451.2078	S	***764.3533***	382.6803	747.3268	374.1670	746.3428	373.6750	7
10	1034.4459	517.7266	1017.4194	509.2133	1016.4353	508.7213	N	677.3213	339.1643	660.2947	330.6510	659.3107	330.1590	6
11	1121.4779	561.2426	1104.4514	552.7293	1103.4674	552.2373	S	***562.2944***	281.6508	545.2678	273.1375	544.2838	272.6455	5
12	1236.5049	618.7561	1219.4783	610.2428	1218.4943	609.7508	N	475.2623	238.1348	458.2358	229.6215			4
13	1293.5263	647.2668	1276.4998	638.7535	1275.5158	638.2615	G	360.2354	180.6213	343.2088	172.1081			3
14	1449.6274	725.3174	1432.6009	716.8041	1431.6169	716.3121	R	303.2139	152.1106	286.1874	143.5973			2
15							K	147.1128	74.0600	130.0863	65.5468			1

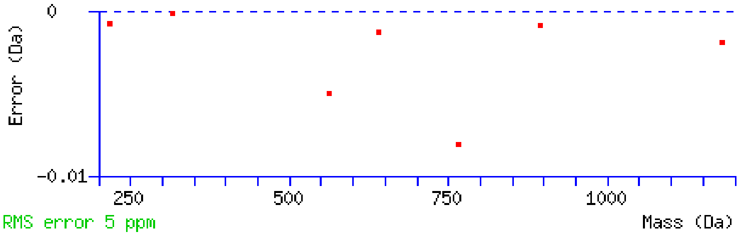

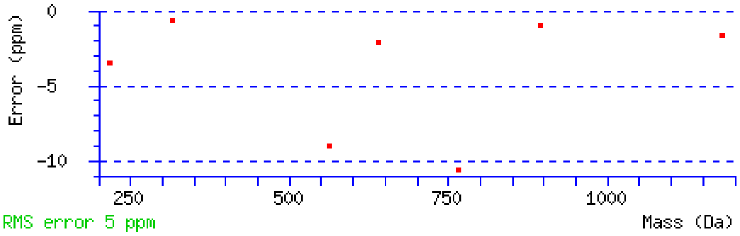

NCBI **BLAST** search of GCVVTSVESNSNGRK
(Parameters: blastp, nr protein database, expect=20000, no filter, PAM30)
Other BLAST web gateways

All matches to this query

Score	Mr(calc)	Delta	Sequence
31.7	1594.7257	0.0002	GCVVTSVESNSNGRK

Mascot: http://www.matrixscience.com/

Mascot Search Results

Peptide View

MS/MS Fragmentation of **GCVVTSVESNSNGRK**
Found in **P08567** in **uni_human_nr**, PLEK_HUMAN Pleckstrin OS=Homo sapiens GN=PLEK PE=1 SV=3

Match to Query 4661: 1593.733842 from(532.251890,3+) intensity(147656.5000) rtinseconds(943) scans(3695) index(1943)
Title: 130806_HPL_Human_Plaque_BR2_TR2_06_Spectrum023748_scans__3695_RTINSECONDS=943
Data file E:\\RAW\\HumanPlaques\\mgf\\T\\1D\\T130806_HPL_Human_Plaque_BR2_TR2_06.mgf

Click mouse within plot area to zoom in by factor of two about that point
Or, Plot from 0 to 1400 Da Full range
Label all possible matches ○ Label matches used for scoring ⊙
Show Y-axis ☐

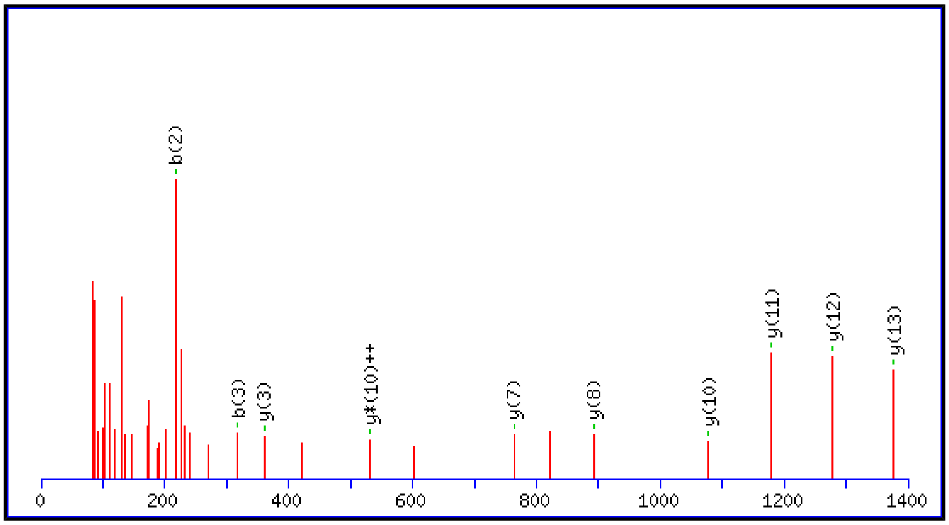

Monoisotopic mass of neutral peptide Mr(calc): 1593.7417
Fixed modifications: Carbamidomethyl (C) (apply to specified residues or termini only)
Variable modifications:
N12 : Deamidated (NQ)
Ions Score: 46 **Expect:** 0.0013
Matches : 10/134 fragment ions using 16 most intense peaks (help)

#	b	b^{++}	b*	b^{*++}	b^0	b^{0++}	Seq.	y	y^{++}	y*	y^{*++}	y^0	y^{0++}	#
1	58.0287	29.5180					G							15
2	**218.0594**	109.5333					C	1537.7275	769.3674	1520.7009	760.8541	1519.7169	760.3621	14
3	**317.1278**	159.0675					V	***1377.6968***	689.3521	1360.6703	680.8388	1359.6863	680.3468	13
4	416.1962	208.6017					V	***1278.6284***	639.8179	1261.6019	631.3046	1260.6179	630.8126	12
5	517.2439	259.1256			499.2333	250.1203	T	***1179.5600***	590.2836	1162.5335	581.7704	1161.5495	581.2784	11
6	604.2759	302.6416			586.2654	293.6363	S	***1078.5123***	539.7598	1061.4858	531.2465	1060.5018	530.7545	10
7	703.3443	352.1758			685.3338	343.1705	V	991.4803	496.2438	974.4538	487.7305	973.4697	487.2385	9
8	832.3869	416.6971			814.3764	407.6918	E	***892.4119***	446.7096	875.3853	438.1963	874.4013	437.7043	8
9	919.4190	460.2131			901.4084	451.2078	S	***763.3693***	382.1883	746.3428	373.6750	745.3587	373.1830	7
10	1033.4619	517.2346	1016.4353	508.7213	1015.4513	508.2293	N	676.3373	338.6723	659.3107	330.1590	658.3267	329.6670	6
11	1120.4939	560.7506	1103.4674	552.2373	1102.4833	551.7453	S	562.2944	281.6508	545.2678	273.1375	544.2838	272.6455	5
12	1235.5209	618.2641	1218.4943	609.7508	1217.5103	609.2588	N	475.2623	238.1348	458.2358	229.6215			4
13	1292.5423	646.7748	1275.5158	638.2615	1274.5318	637.7695	G	***360.2354***	180.6213	343.2088	172.1081			3
14	1448.6434	724.8254	1431.6169	716.3121	1430.6329	715.8201	R	303.2139	152.1106	286.1874	143.5973			2
15							K	147.1128	74.0600	130.0863	65.5468			1

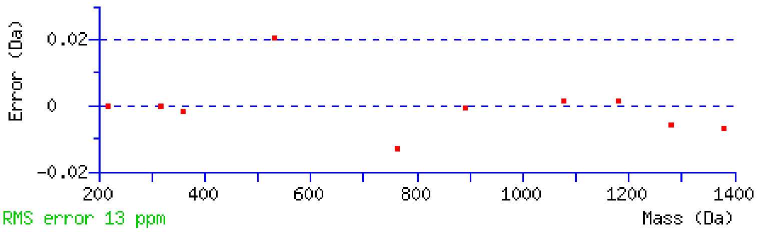

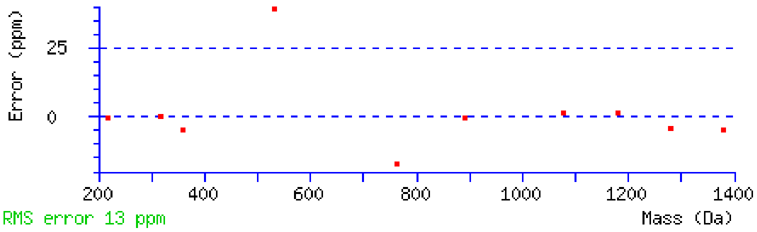

NCBI **BLAST** search of GCVVTSVESNSNGRK
(Parameters: blastp, nr protein database, expect=20000, no filter, PAM30)
Other BLAST web gateways

All matches to this query

Score	Mr(calc)	Delta	Sequence	Site Analysis
46.4	1593.7417	-0.0078	GCVVTSVESNSNGRK	Deamidated N12 50.00%
46.4	1593.7417	-0.0078	GCVVTSVESNSNGRK	Deamidated N10 50.00%
0.3	1592.7351	0.9987	KQEESVQKQEAMR	

Mascot: http://www.matrixscience.com/

Mascot Search Results

Peptide View

MS/MS Fragmentation of **EAQLPVIENKVCNRYEFLNGR**
Found in **P00747** in **uni_human_nr**, PLMN_HUMAN Plasminogen OS=Homo sapiens GN=PLG PE=1 SV=2

Match to Query 10498: 2551.237256 from(638.816590,4+) intensity(184588.1875) rtinseconds(2439) scans(13237) index(9678)
Title: 130801_HPL_Human_Plaque_BR2_TR1_15_Spectrum029440_scans__13237_RTINSECONDS=2439
Data file E:\\RAW\\HumanPlaques\\mgf\\T\\1D\\T130801_HPL_Human_Plaque_BR2_TR1_15.mgf

Click mouse within plot area to zoom in by factor of two about that point
Or, [Plot from] [0] to [2200] Da [Full range]
Label all possible matches ○ Label matches used for scoring ⊙
Show Y-axis ☐

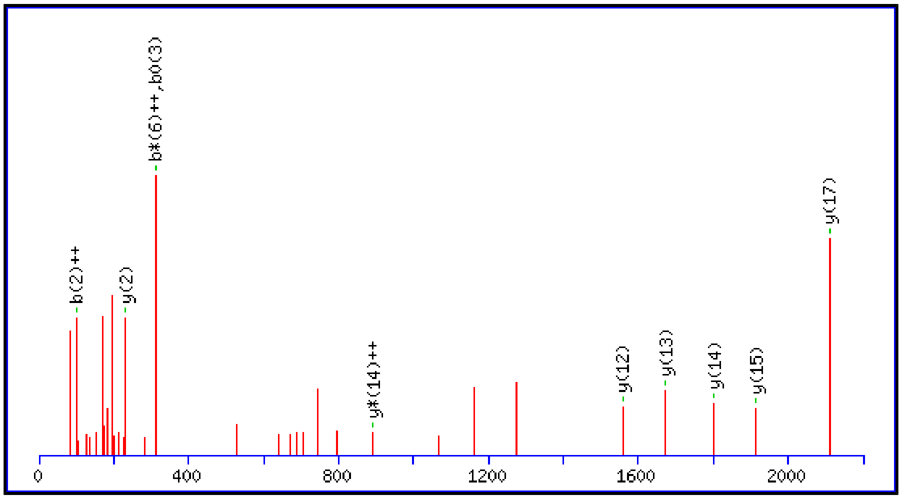

Monoisotopic mass of neutral peptide Mr(calc): 2551.2376
Fixed modifications: Carbamidomethyl (C) (apply to specified residues or termini only)
Variable modifications:
N9 : Deamidated (NQ)
N13 : Deamidated (NQ)
N19 : Deamidated (NQ)
Ions Score: 22 **Expect:** 0.63
Matches : 10/226 fragment ions using 20 most intense peaks (help)

#	b	b^{++}	b*	b^{*++}	b^{0}	b^{0++}	Seq.	y	y^{++}	y*	y^{*++}	y^{0}	y^{0++}	#
1	130.0499	65.5286			112.0393	56.5233	E							21
2	201.0870	**101.0471**			183.0764	92.0418	A	2423.2024	1212.1048	2406.1758	1203.5915	2405.1918	1203.0995	20
3	329.1456	165.0764	312.1190	156.5631	311.1350	156.0711	Q	2352.1653	1176.5863	2335.1387	1168.0730	2334.1547	1167.5810	19
4	442.2296	221.6185	425.2031	213.1052	424.2191	212.6132	L	2224.1067	1112.5570	2207.0801	1104.0437	2206.0961	1103.5517	18
5	539.2824	270.1448	522.2558	261.6316	521.2718	261.1396	P	***2111.0226***	1056.0149	2093.9961	1047.5017	2093.0121	1047.0097	17
6	638.3508	319.6790	621.3243	311.1658	620.3402	310.6738	V	2013.9699	1007.4886	1996.9433	998.9753	1995.9593	998.4833	16
7	751.4349	376.2211	734.4083	367.7078	733.4243	367.2158	I	***1914.9014***	957.9544	1897.8749	949.4411	1896.8909	948.9491	15
8	880.4775	440.7424	863.4509	432.2291	862.4669	431.7371	E	***1801.8174***	901.4123	1784.7908	892.8991	1783.8068	892.4070	14
9	995.5044	498.2558	978.4779	489.7426	977.4938	489.2506	N	***1672.7748***	836.8910	1655.7482	828.3778	1654.7642	827.8857	13
10	1123.5994	562.3033	1106.5728	553.7900	1105.5888	553.2980	K	***1557.7478***	779.3776	1540.7213	770.8643	1539.7373	770.3723	12
11	1222.6678	611.8375	1205.6412	603.3243	1204.6572	602.8322	V	1429.6529	715.3301	1412.6263	706.8168	1411.6423	706.3248	11
12	1382.6984	691.8529	1365.6719	683.3396	1364.6879	682.8476	C	1330.5845	665.7959	1313.5579	657.2826	1312.5739	656.7906	10
13	1497.7254	749.3663	1480.6988	740.8530	1479.7148	740.3610	N	1170.5538	585.7805	1153.5273	577.2673	1152.5432	576.7753	9
14	1653.8265	827.4169	1636.7999	818.9036	1635.8159	818.4116	R	1055.5269	528.2671	1038.5003	519.7538	1037.5163	519.2618	8
15	1816.8898	908.9485	1799.8633	900.4353	1798.8792	899.9433	Y	899.4258	450.2165	882.3992	441.7032	881.4152	441.2112	7
16	1945.9324	973.4698	1928.9059	964.9566	1927.9218	964.4646	E	736.3624	368.6849	719.3359	360.1716	718.3519	359.6796	6
17	2093.0008	1047.0040	2075.9743	1038.4908	2074.9903	1037.9988	F	607.3198	304.1636	590.2933	295.6503			5

18	2206.0849	1103.5461	2189.0583	1095.0328	2188.0743	1094.5408	L	460.2514	230.6293	443.2249	222.1161			4
19	2321.1118	1161.0596	2304.0853	1152.5463	2303.1013	1152.0543	N	347.1674	174.0873	330.1408	165.5740			3
20	2378.1333	1189.5703	2361.1067	1181.0570	2360.1227	1180.5650	G	***232.1404***	116.5738	215.1139	108.0606			2
21							R	175.1190	88.0631	158.0924	79.5498			1

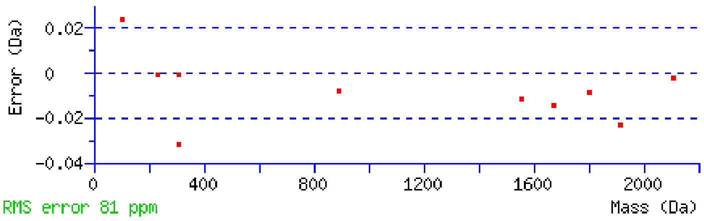

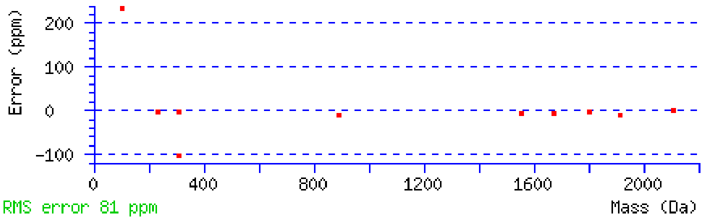

NCBI **BLAST** search of EAQLPVIENKVCNRYEFLNGR
(Parameters: blastp, nr protein database, expect=20000, no filter, PAM30)
Other BLAST web gateways

All matches to this query

Score	Mr(calc)	Delta	Sequence
21.8	2551.2376	-0.0004	EAQLPVIENKVCNRYEFLNGR

Mascot: http://www.matrixscience.com/

Mascot Search Results

Peptide View

MS/MS Fragmentation of **EAQLPVIENKVCNRYEFLNGR**
Found in **P00747** in **uni_human_nr**, PLMN_HUMAN Plasminogen OS=Homo sapiens GN=PLG PE=1 SV=2

Match to Query 11809: 2550.258496 from(638.571900,4+) intensity(236151.2969) rtinseconds(2350) scans(13506) index(10352)
Title: 130809_HPL_Human_Plaque_BR2_TR3_09_Spectrum031679_scans__13506_RTINSECONDS=2350
Data file E:\\RAW\\HumanPlaques\\mgf\\T\\1D\\T130809_HPL_Human_Plaque_BR2_TR3_09.mgf

Click mouse within plot area to zoom in by factor of two about that point
Or, Plot from 0 to 2400 Da Full range
Label all possible matches ○ Label matches used for scoring ⦿
Show Y-axis ☐

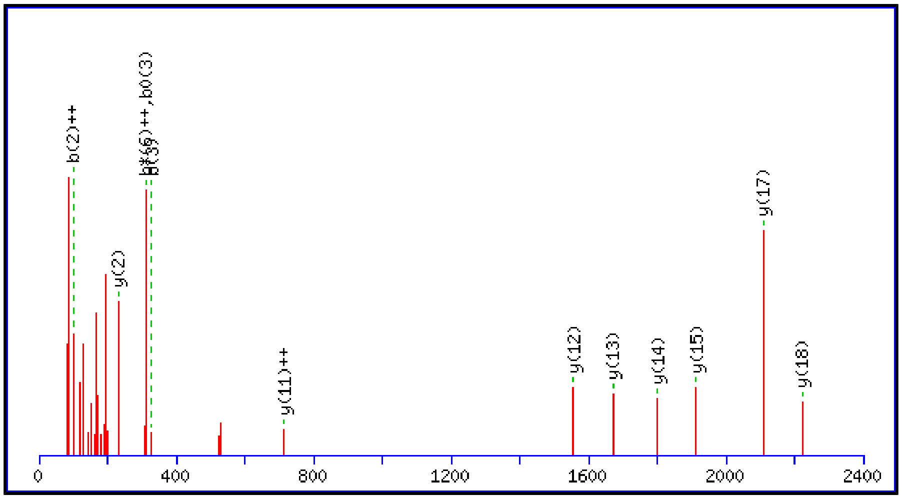

Monoisotopic mass of neutral peptide Mr(calc): 2550.2536
Fixed modifications: Carbamidomethyl (C) (apply to specified residues or termini only)
Variable modifications:
N9 : Deamidated (NQ)
N19 : Deamidated (NQ)
Ions Score: 40 **Expect:** 0.0098
Matches : 12/226 fragment ions using 19 most intense peaks (help)

#	b	b^{++}	b*	b^{*++}	b^0	b^{0++}	Seq.	y	y^{++}	y*	y^{*++}	y^0	y^{0++}	#
1	130.0499	65.5286			112.0393	56.5233	E							21
2	201.0870	**101.0471**			183.0764	92.0418	A	2422.2184	1211.6128	2405.1918	1203.0995	2404.2078	1202.6075	20
3	**329.1456**	165.0764	312.1190	156.5631	311.1350	156.0711	Q	2351.1812	1176.0943	2334.1547	1167.5810	2333.1707	1167.0890	19
4	442.2296	221.6185	425.2031	213.1052	424.2191	212.6132	L	***2223.1227***	1112.0650	2206.0961	1103.5517	2205.1121	1103.0597	18
5	539.2824	270.1448	522.2558	261.6316	521.2718	261.1396	P	***2110.0386***	1055.5229	2093.0121	1047.0097	2092.0280	1046.5177	17
6	638.3508	319.6790	621.3243	311.1658	620.3402	310.6738	V	2012.9858	1006.9966	1995.9593	998.4833	1994.9753	997.9913	16
7	751.4349	376.2211	734.4083	367.7078	733.4243	367.2158	I	***1913.9174***	957.4623	1896.8909	948.9491	1895.9069	948.4571	15
8	880.4775	440.7424	863.4509	432.2291	862.4669	431.7371	E	***1800.8334***	900.9203	1783.8068	892.4070	1782.8228	891.9150	14
9	995.5044	498.2558	978.4779	489.7426	977.4938	489.2506	N	***1671.7908***	836.3990	1654.7642	827.8857	1653.7802	827.3937	13
10	1123.5994	562.3033	1106.5728	553.7900	1105.5888	553.2980	K	***1556.7638***	778.8855	1539.7373	770.3723	1538.7533	769.8803	12
11	1222.6678	611.8375	1205.6412	603.3243	1204.6572	602.8322	V	1428.6689	714.8381	1411.6423	706.3248	1410.6583	705.8328	11
12	1382.6984	691.8529	1365.6719	683.3396	1364.6879	682.8476	C	1329.6004	665.3039	1312.5739	656.7906	1311.5899	656.2986	10
13	1496.7414	748.8743	1479.7148	740.3610	1478.7308	739.8690	N	1169.5698	585.2885	1152.5432	576.7753	1151.5592	576.2833	9
14	1652.8425	826.9249	1635.8159	818.4116	1634.8319	817.9196	R	1055.5269	528.2671	1038.5003	519.7538	1037.5163	519.2618	8
15	1815.9058	908.4565	1798.8792	899.9433	1797.8952	899.4513	Y	899.4258	450.2165	882.3992	441.7032	881.4152	441.2112	7
16	1944.9484	972.9778	1927.9218	964.4646	1926.9378	963.9726	E	736.3624	368.6849	719.3359	360.1716	718.3519	359.6796	6
17	2092.0168	1046.5120	2074.9903	1037.9988	2074.0062	1037.5068	F	607.3198	304.1636	590.2933	295.6503			5

**18**	2205.1009	1103.0541	2188.0743	1094.5408	2187.0903	1094.0488	**L**	460.2514	230.6293	443.2249	222.1161			**4**
**19**	2320.1278	1160.5675	2303.1013	1152.0543	2302.1172	1151.5623	**N**	347.1674	174.0873	330.1408	165.5740			**3**
**20**	2377.1493	1189.0783	2360.1227	1180.5650	2359.1387	1180.0730	**G**	***232.1404***	116.5738	215.1139	108.0606			**2**
**21**							**R**	175.1190	88.0631	158.0924	79.5498			**1**

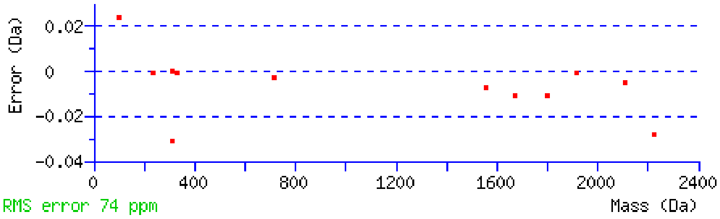

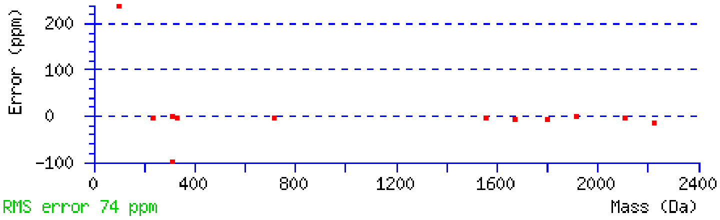

NCBI **BLAST** search of EAQLPVIENKVCNRYEFLNGR
(Parameters: blastp, nr protein database, expect=20000, no filter, PAM30)
Other BLAST web gateways

All matches to this query

Score	Mr(calc)	Delta	Sequence	Site Analysis
39.9	2550.2536	0.0049	EAQLPVIENKVCNRYEFLNGR	Deamidated N9, N19 48.66%
39.9	2550.2536	0.0049	EAQLPVIENKVCNRYEFLNGR	Deamidated N9, N13 48.66%
27.3	2550.2536	0.0049	EAQLPVIENKVCNRYEFLNGR	Deamidated N13, N19 2.67%
0.7	2550.2496	0.0089	MEAPEEPAPVRGGPEATLEVRGSR	

Mascot: http://www.matrixscience.com/

Mascot Search Results

Peptide View

MS/MS Fragmentation of **VCNRYEFLNGR**
Found in **P00747** in **uni_human_nr**, PLMN_HUMAN Plasminogen OS=Homo sapiens GN=PLG PE=1 SV=2

Match to Query 3162: 1428.646872 from(477.222900,3+) intensity(612972.4375) rtinseconds(1600) scans(8281) index(5949)
Title: 130809_HPL_Human_Plaque_BR2_TR3_09_Spectrum027276_scans__8281_RTINSECONDS=1600
Data file E:\\RAW\\HumanPlaques\\mgf\\T\\1D\\T130809_HPL_Human_Plaque_BR2_TR3_09.mgf

Click mouse within plot area to zoom in by factor of two about that point
Or, Plot from 0 to 1400 Da Full range
Label all possible matches ○ Label matches used for scoring ⊙
Show Y-axis ☐

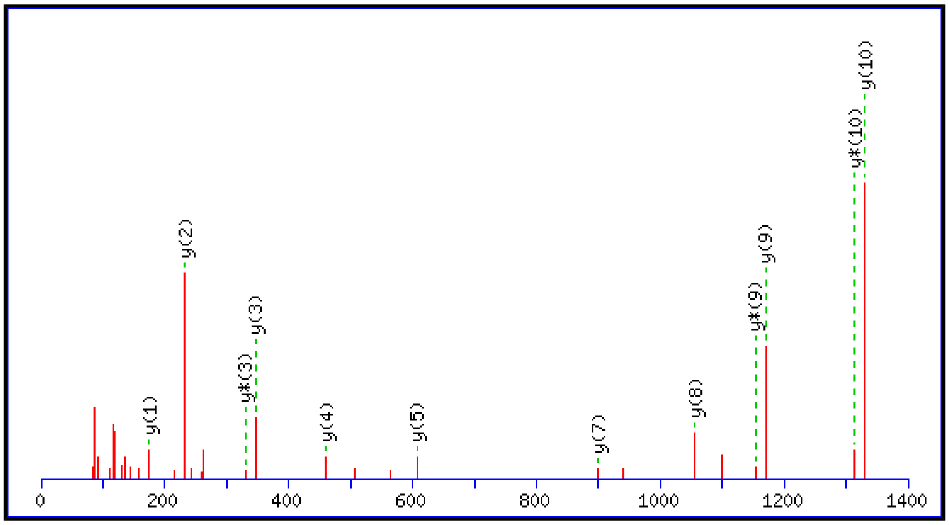

Monoisotopic mass of neutral peptide Mr(calc): 1428.6456
Fixed modifications: Carbamidomethyl (C) (apply to specified residues or termini only)
Variable modifications:
N3 : Deamidated (NQ)
N9 : Deamidated (NQ)
Ions Score: 69 **Expect:** 3.8e-006
Matches : 12/96 fragment ions using 22 most intense peaks (help)

#	b	b++	b*	b*++	b0	b0++	Seq.	y	y++	y*	y*++	y0	y0++	#
1	100.0757	50.5415					V							11
2	260.1063	130.5568					C	***1330.5845***	665.7959	1313.5579	657.2826	1312.5739	656.7906	10
3	375.1333	188.0703	358.1067	179.5570			N	***1170.5538***	585.7805	1153.5273	577.2673	1152.5432	576.7753	9
4	531.2344	266.1208	514.2078	257.6076			R	***1055.5269***	528.2671	1038.5003	519.7538	1037.5163	519.2618	8
5	694.2977	347.6525	677.2712	339.1392			Y	***899.4258***	450.2165	882.3992	441.7032	881.4152	441.2112	7
6	823.3403	412.1738	806.3138	403.6605	805.3297	403.1685	E	736.3624	368.6849	719.3359	360.1716	718.3519	359.6796	6
7	970.4087	485.7080	953.3822	477.1947	952.3982	476.7027	F	***607.3198***	304.1636	590.2933	295.6503			5
8	1083.4928	542.2500	1066.4662	533.7368	1065.4822	533.2448	L	***460.2514***	230.6293	443.2249	222.1161			4
9	1198.5197	599.7635	1181.4932	591.2502	1180.5092	590.7582	N	***347.1674***	174.0873	330.1408	165.5740			3
10	1255.5412	628.2742	1238.5147	619.7610	1237.5306	619.2690	G	***232.1404***	116.5738	215.1139	108.0606			2
11							R	***175.1190***	88.0631	158.0924	79.5498			1

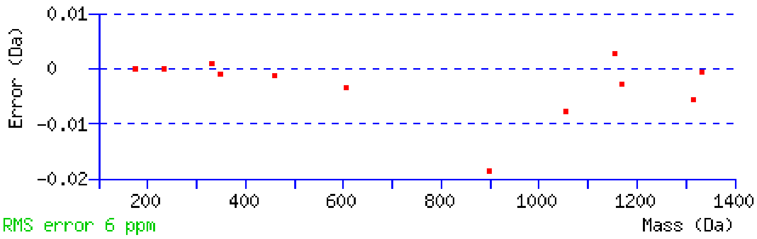

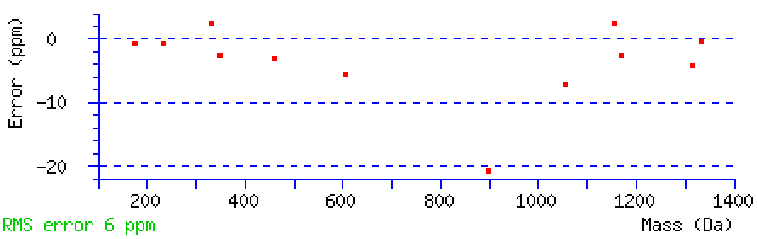

NCBI **BLAST** search of VCNRYEFLNGR
(Parameters: blastp, nr protein database, expect=20000, no filter, PAM30)
Other BLAST web gateways

All matches to this query

Score	Mr(calc)	Delta	Sequence
69.4	1428.6456	0.0013	VCNRYEFLNGR
11.5	1428.6456	0.0013	VCFQYLNRGDR
2.8	1427.6463	1.0006	KMSQNTQDGYTR
0.1	1427.6389	1.0080	EHLQRQNSSNGR
0.1	1427.6389	1.0080	EHLQRQNSSNGR
0.1	1427.6389	1.0080	EHLQRQNSSNGR

Mascot: http://www.matrixscience.com/

Mascot Search Results

Peptide View

MS/MS Fragmentation of **VCNRYEFLNGR**
Found in **P00747** in **uni_human_nr**, PLMN_HUMAN Plasminogen OS=Homo sapiens GN=PLG PE=1 SV=2

Match to Query 3527: 1427.660022 from(476.893950,3+) intensity(1944138.5000) rtinseconds(1401) scans(7580) index(5459)
Title: 130806_HPL_Human_Plaque_BR1_TR2_03_Spectrum029398_scans__7580_RTINSECONDS=1401
Data file E:\\RAW\\HumanPlaques\\mgf\\T\\1D\\T130806_HPL_Human_Plaque_BR1_TR2_03.mgf

Click mouse within plot area to zoom in by factor of two about that point
Or, [Plot from] [0] to [1400] Da [Full range]
Label all possible matches ○ Label matches used for scoring ⊙
Show Y-axis ☐

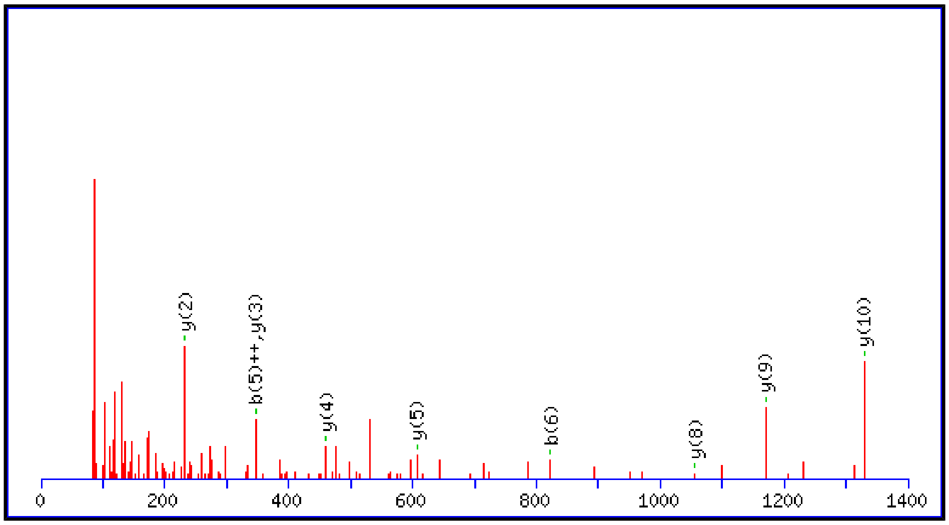

Monoisotopic mass of neutral peptide Mr(calc): 1427.6616
Fixed modifications: Carbamidomethyl (C) (apply to specified residues or termini only)
Variable modifications:
N9 : Deamidated (NQ)
Ions Score: 49 **Expect:** 0.00063
Matches : 9/96 fragment ions using 13 most intense peaks (help)

#	b	b^{++}	b*	b^{*++}	b^{0}	b^{0++}	Seq.	y	y^{++}	y*	y^{*++}	y^{0}	y^{0++}	#
1	100.0757	50.5415					V							11
2	260.1063	130.5568					C	***1329.6004***	665.3039	1312.5739	656.7906	1311.5899	656.2986	10
3	374.1493	187.5783	357.1227	179.0650			N	***1169.5698***	585.2885	1152.5432	576.7753	1151.5592	576.2833	9
4	530.2504	265.6288	513.2238	257.1156			R	***1055.5269***	528.2671	1038.5003	519.7538	1037.5163	519.2618	8
5	693.3137	**347.1605**	676.2872	338.6472			Y	899.4258	450.2165	882.3992	441.7032	881.4152	441.2112	7
6	**822.3563**	411.6818	805.3298	403.1685	804.3457	402.6765	E	736.3624	368.6849	719.3359	360.1716	718.3519	359.6796	6
7	969.4247	485.2160	952.3982	476.7027	951.4141	476.2107	F	***607.3198***	304.1636	590.2933	295.6503			5
8	1082.5088	541.7580	1065.4822	533.2448	1064.4982	532.7527	L	***460.2514***	230.6293	443.2249	222.1161			4
9	1197.5357	599.2715	1180.5092	590.7582	1179.5252	590.2662	N	***347.1674***	174.0873	330.1408	165.5740			3
10	1254.5572	627.7822	1237.5306	619.2690	1236.5466	618.7769	G	***232.1404***	116.5738	215.1139	108.0606			2
11							R	175.1190	88.0631	158.0924	79.5498			1

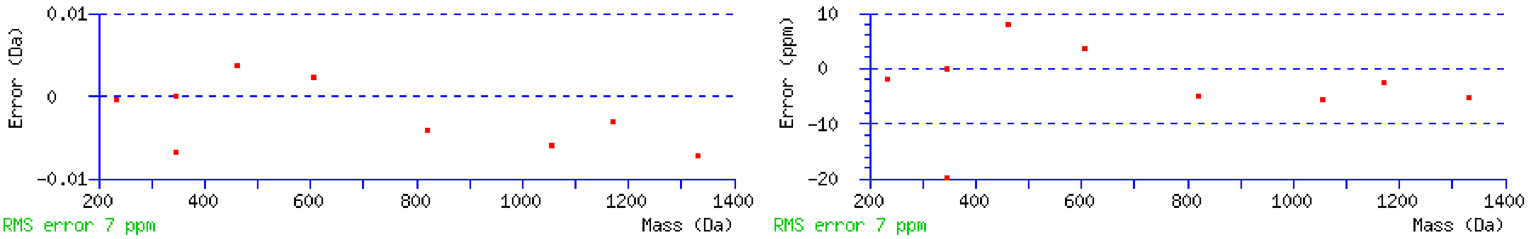

NCBI **BLAST** search of VCNRYEFLNGR
(Parameters: blastp, nr protein database, expect=20000, no filter, PAM30)
Other BLAST web gateways

All matches to this query

Score	Mr(calc)	Delta	Sequence	Site Analysis
48.5	1427.6616	-0.0015	VCNRYEFLNGR	Deamidated N9 100.00%
0.7	1427.6616	-0.0015	VCNRYEFLNGR	Deamidated N3 0.00%
0.3	1427.6616	-0.0016	VCFQYLNRGDR	

Mascot: http://www.matrixscience.com/

Mascot Search Results

Peptide View

MS/MS Fragmentation of **VCNRYEFLNGR**
Found in **P00747** in **uni_human_nr**, PLMN_HUMAN Plasminogen OS=Homo sapiens GN=PLG PE=1 SV=2

Match to Query 3981: 1426.676472 from(476.566100,3+) intensity(14840261.0000) rtinseconds(1181) scans(5596) index(3506)
Title: 130809_HPL_Human_Plaque_BR2_TR3_02_Spectrum028359_scans__5596_RTINSECONDS=1181
Data file E:\\RAW\\HumanPlaques\\mgf\\T\\1D\\T130809_HPL_Human_Plaque_BR2_TR3_02.mgf

Click mouse within plot area to zoom in by factor of two about that point
Or, Plot from 0 to 1400 Da Full range
Label all possible matches ○ Label matches used for scoring ◉
Show Y-axis ☐

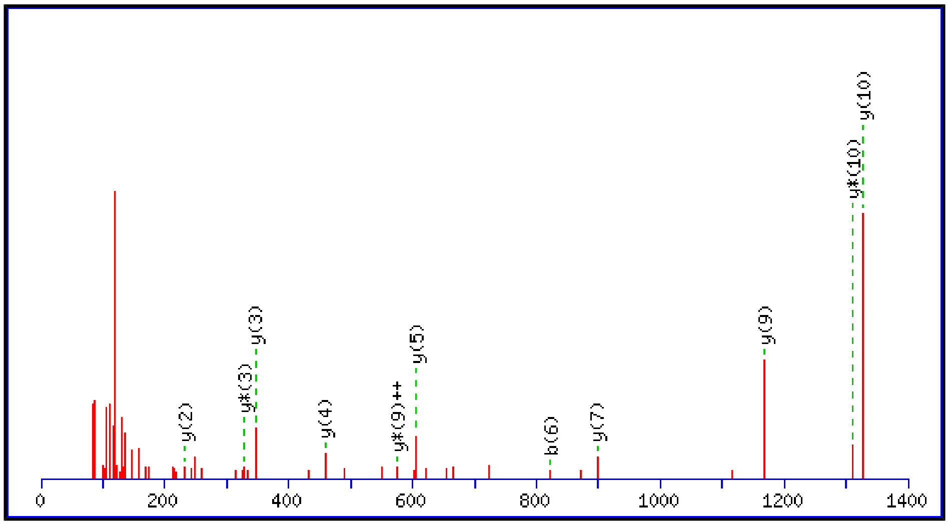

Monoisotopic mass of neutral peptide Mr(calc): 1426.6776
Fixed modifications: Carbamidomethyl (C) (apply to specified residues or termini only)
Ions Score: 44 **Expect:** 0.0024
Matches : 11/96 fragment ions using 20 most intense peaks (help)

#	b	b++	b*	b*++	b0	b0++	Seq.	y	y++	y*	y*++	y0	y0++	#
1	100.0757	50.5415					V							11
2	260.1063	130.5568					C	***1328.6164***	664.8119	1311.5899	656.2986	1310.6059	655.8066	10
3	374.1493	187.5783	357.1227	179.0650			N	***1168.5858***	584.7965	1151.5592	576.2833	1150.5752	575.7912	9
4	530.2504	265.6288	513.2238	257.1156			R	1054.5429	527.7751	1037.5163	519.2618	1036.5323	518.7698	8
5	693.3137	347.1605	676.2872	338.6472			Y	***898.4417***	449.7245	881.4152	441.2112	880.4312	440.7192	7
6	**822.3563**	411.6818	805.3298	403.1685	804.3457	402.6765	E	735.3784	368.1928	718.3519	359.6796	717.3678	359.1876	6
7	969.4247	485.2160	952.3982	476.7027	951.4141	476.2107	F	***606.3358***	303.6715	589.3093	295.1583			5
8	1082.5088	541.7580	1065.4822	533.2448	1064.4982	532.7527	L	***459.2674***	230.1373	442.2409	221.6241			4
9	1196.5517	598.7795	1179.5252	590.2662	1178.5411	589.7742	N	***346.1833***	173.5953	329.1568	165.0820			3
10	1253.5732	627.2902	1236.5466	618.7769	1235.5626	618.2849	G	***232.1404***	116.5738	215.1139	108.0606			2
11							R	175.1190	88.0631	158.0924	79.5498			1

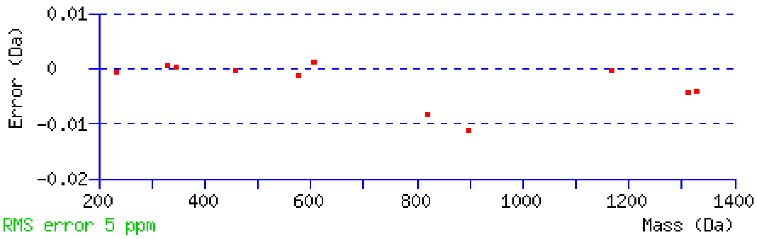

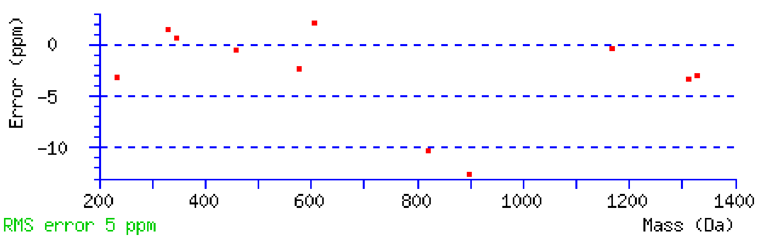

NCBI **BLAST** search of VCNRYEFLNGR
(Parameters: blastp, nr protein database, expect=20000, no filter, PAM30)
Other BLAST web gateways

All matches to this query

Score	Mr(calc)	Delta	Sequence
43.5	1426.6776	-0.0011	VCNRYEFLNGR
0.8	1426.6704	0.0061	VAPIWACPHFET

Mascot: http://www.matrixscience.com/

Mascot Search Results

Peptide View

MS/MS Fragmentation of **YEFLNGR**
Found in **P00747** in **uni_human_nr**, PLMN_HUMAN Plasminogen OS=Homo sapiens GN=PLG PE=1 SV=2

Match to Query 294: 898.418368 from(450.216460,2+) intensity(4647658.0000) rtinseconds(1664) scans(9090) index(6699)
Title: 130809_HPL_Human_Plaque_BR1_TR3_06_Spectrum029053_scans__9090_RTINSECONDS=1664
Data file E:\\RAW\\HumanPlaques\\mgf\\T\\1D\\T130809_HPL_Human_Plaque_BR1_TR3_06.mgf

Click mouse within plot area to zoom in by factor of two about that point
Or, Plot from 50 to 750 Da Full range
Label all possible matches ○ Label matches used for scoring ◉
Show Y-axis ☐

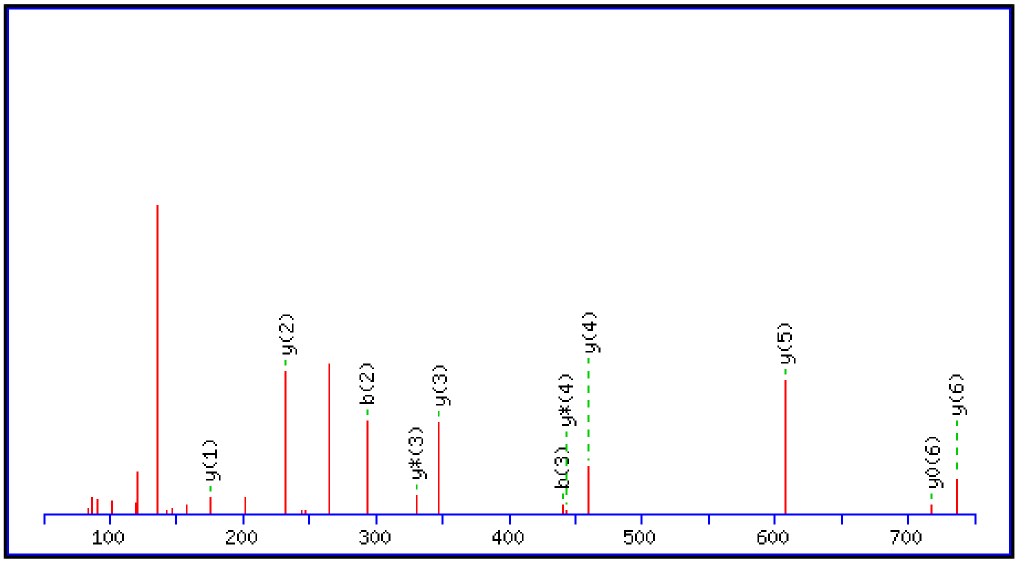

Monoisotopic mass of neutral peptide Mr(calc): 898.4185
Fixed modifications: Carbamidomethyl (C) (apply to specified residues or termini only)
Variable modifications:
N5 : Deamidated (NQ)
Ions Score: 51 **Expect:** 0.00023
Matches : 11/52 fragment ions using 15 most intense peaks (help)

#	b	b^{++}	b*	b^{*++}	b^{0}	b^{0++}	Seq.	y	y^{++}	y*	y^{*++}	y^{0}	y^{0++}	#
1	164.0706	82.5389					Y							7
2	**293.1132**	147.0602			275.1026	138.0550	E	***736.3624***	368.6849	719.3359	360.1716	718.3519	359.6796	6
3	**440.1816**	220.5944			422.1710	211.5892	F	***607.3198***	304.1636	590.2933	295.6503			5
4	553.2657	277.1365			535.2551	268.1312	L	***460.2514***	230.6293	443.2249	222.1161			4
5	668.2926	334.6499	651.2661	326.1367	650.2821	325.6447	N	***347.1674***	174.0873	330.1408	165.5740			3
6	725.3141	363.1607	708.2875	354.6474	707.3035	354.1554	G	***232.1404***	116.5738	215.1139	108.0606			2
7							R	***175.1190***	88.0631	158.0924	79.5498			1

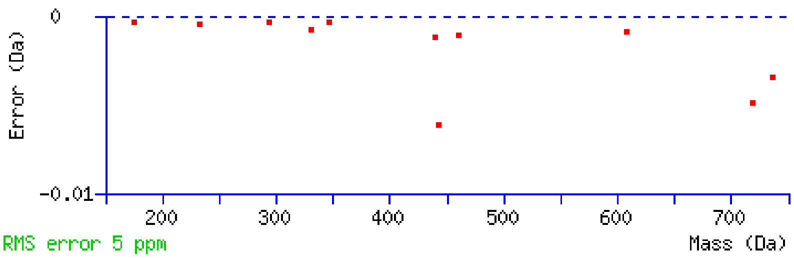

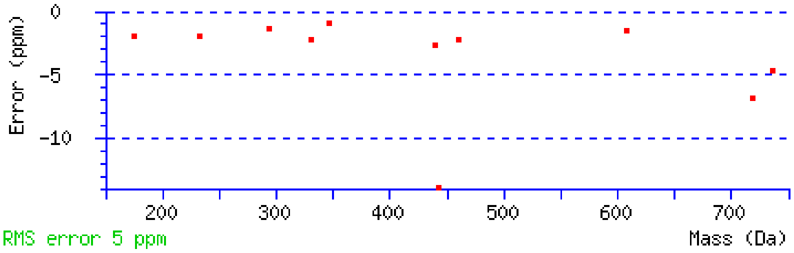

NCBI **BLAST** search of YEFLNGR
(Parameters: blastp, nr protein database, expect=20000, no filter, PAM30)
Other BLAST web gateways

All matches to this query

Score	Mr(calc)	Delta	Sequence
50.7	898.4185	-0.0001	YEFLNGR
15.9	898.4185	-0.0001	YFIEDGR
15.9	898.4185	-0.0001	YLFENGR
4.2	898.4144	0.0039	YKDSSSGR

Mascot: http://www.matrixscience.com/

Mascot Search Results

Peptide View

MS/MS Fragmentation of **YEFLNGRVQSTELCAGHLAGGTDSCQGDSGGPLVCFEK**
Found in **P00747** in **uni_human_nr**, PLMN_HUMAN Plasminogen OS=Homo sapiens GN=PLG PE=1 SV=2

Match to Query 17111: 4117.822936 from(1030.463010,4+) intensity(752991.0625) rtinseconds(2874) scans(16785) index(12921)
Title: 130806_HPL_Human_Plaque_BR2_TR2_10_Spectrum033842_scans__16785_RTINSECONDS=2874
Data file E:\\RAW\\HumanPlaques\\mgf\\T\\1D\\T130806_HPL_Human_Plaque_BR2_TR2_10.mgf

Click mouse within plot area to zoom in by factor of two about that point
Or, Plot from 0 to 2600 Da Full range
Label all possible matches ○ Label matches used for scoring ⊙
Show Y-axis ☐

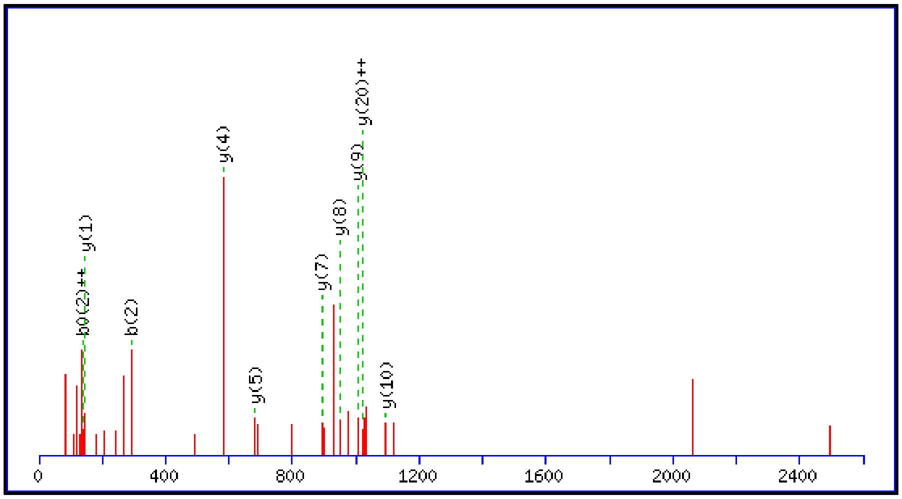

Monoisotopic mass of neutral peptide Mr(calc): 4117.8092
Fixed modifications: Carbamidomethyl (C) (apply to specified residues or termini only)
Variable modifications:
N5 : Deamidated (NQ)
Q9 : Deamidated (NQ)
Ions Score: 18 **Expect:** 0.57
Matches : 10/432 fragment ions using 32 most intense peaks (help)

#	b	b^{++}	b*	b^{*++}	b^0	b^{0++}	Seq.	y	y^{++}	y*	y^{*++}	y^0	y^{0++}	#
1	164.0706	82.5389					Y							38
2	**293.1132**	147.0602			275.1026	138.0550	E	3955.7531	1978.3802	3938.7266	1969.8669	3937.7426	1969.3749	37
3	440.1816	220.5944			422.1710	211.5892	F	3826.7106	1913.8589	3809.6840	1905.3456	3808.7000	1904.8536	36
4	553.2657	277.1365			535.2551	268.1312	L	3679.6421	1840.3247	3662.6156	1831.8114	3661.6316	1831.3194	35
5	668.2926	334.6499	651.2661	326.1367	650.2821	325.6447	N	3566.5581	1783.7827	3549.5315	1775.2694	3548.5475	1774.7774	34
6	725.3141	363.1607	708.2875	354.6474	707.3035	354.1554	G	3451.5311	1726.2692	3434.5046	1717.7559	3433.5206	1717.2639	33
7	881.4152	441.2112	864.3886	432.6980	863.4046	432.2060	R	3394.5097	1697.7585	3377.4831	1689.2452	3376.4991	1688.7532	32
8	980.4836	490.7454	963.4571	482.2322	962.4730	481.7402	V	3238.4086	1619.7079	3221.3820	1611.1946	3220.3980	1610.7026	31
9	1109.5262	555.2667	1092.4997	546.7535	1091.5156	546.2615	Q	3139.3401	1570.1737	3122.3136	1561.6604	3121.3296	1561.1684	30
10	1196.5582	598.7828	1179.5317	590.2695	1178.5477	589.7775	S	3010.2976	1505.6524	2993.2710	1497.1391	2992.2870	1496.6471	29
11	1297.6059	649.3066	1280.5794	640.7933	1279.5953	640.3013	T	2923.2655	1462.1364	2906.2390	1453.6231	2905.2550	1453.1311	28
12	1426.6485	713.8279	1409.6220	705.3146	1408.6379	704.8226	E	2822.2178	1411.6126	2805.1913	1403.0993	2804.2073	1402.6073	27
13	1539.7326	770.3699	1522.7060	761.8566	1521.7220	761.3646	L	2693.1753	1347.0913	2676.1487	1338.5780	2675.1647	1338.0860	26
14	1699.7632	850.3852	1682.7367	841.8720	1681.7527	841.3800	C	2580.0912	1290.5492	2563.0646	1282.0360	2562.0806	1281.5439	25
15	1770.8003	885.9038	1753.7738	877.3905	1752.7898	876.8985	A	2420.0605	1210.5339	2403.0340	1202.0206	2402.0500	1201.5286	24
16	1827.8218	914.4145	1810.7952	905.9013	1809.8112	905.4093	G	2349.0234	1175.0153	2331.9969	1166.5021	2331.0129	1166.0101	23
17	1964.8807	982.9440	1947.8542	974.4307	1946.8701	973.9387	H	2292.0020	1146.5046	2274.9754	1137.9913	2273.9914	1137.4993	22

**18**	2077.9648	1039.4860	2060.9382	1030.9727	2059.9542	1030.4807	**L**	2154.9430	1077.9752	2137.9165	1069.4619	2136.9325	1068.9699	**21**
**19**	2149.0019	1075.0046	2131.9753	1066.4913	2130.9913	1065.9993	**A**	2041.8590	1021.4331	2024.8324	1012.9199	2023.8484	1012.4278	**20**
**20**	2206.0233	1103.5153	2188.9968	1095.0020	2188.0128	1094.5100	**G**	1970.8219	985.9146	1953.7953	977.4013	1952.8113	976.9093	**19**
**21**	2263.0448	1132.0260	2246.0183	1123.5128	2245.0342	1123.0208	**G**	1913.8004	957.4038	1896.7739	948.8906	1895.7898	948.3986	**18**
**22**	2364.0925	1182.5499	2347.0659	1174.0366	2346.0819	1173.5446	**T**	1856.7789	928.8931	1839.7524	920.3798	1838.7684	919.8878	**17**
**23**	2479.1194	1240.0634	2462.0929	1231.5501	2461.1089	1231.0581	**D**	1755.7313	878.3693	1738.7047	869.8560	1737.7207	869.3640	**16**
**24**	2566.1515	1283.5794	2549.1249	1275.0661	2548.1409	1274.5741	**S**	1640.7043	820.8558	1623.6778	812.3425	1622.6938	811.8505	**15**
**25**	2726.1821	1363.5947	2709.1556	1355.0814	2708.1715	1354.5894	**C**	1553.6723	777.3398	1536.6457	768.8265	1535.6617	768.3345	**14**
**26**	2854.2407	1427.6240	2837.2141	1419.1107	2836.2301	1418.6187	**Q**	1393.6416	697.3245	1376.6151	688.8112	1375.6311	688.3192	**13**
**27**	2911.2622	1456.1347	2894.2356	1447.6214	2893.2516	1447.1294	**G**	1265.5831	633.2952	1248.5565	624.7819	1247.5725	624.2899	**12**
**28**	3026.2891	1513.6482	3009.2625	1505.1349	3008.2785	1504.6429	**D**	1208.5616	604.7844	1191.5351	596.2712	1190.5510	595.7792	**11**
**29**	3113.3211	1557.1642	3096.2946	1548.6509	3095.3106	1548.1589	**S**	***1093.5347***	547.2710	1076.5081	538.7577	1075.5241	538.2657	**10**
**30**	3170.3426	1585.6749	3153.3160	1577.1617	3152.3320	1576.6696	**G**	***1006.5026***	503.7550	989.4761	495.2417	988.4921	494.7497	**9**
**31**	3227.3641	1614.1857	3210.3375	1605.6724	3209.3535	1605.1804	**G**	***949.4812***	475.2442	932.4546	466.7309	931.4706	466.2389	**8**
**32**	3324.4168	1662.7120	3307.3903	1654.1988	3306.4062	1653.7068	**P**	***892.4597***	446.7335	875.4332	438.2202	874.4491	437.7282	**7**
**33**	3437.5009	1719.2541	3420.4743	1710.7408	3419.4903	1710.2488	**L**	795.4069	398.2071	778.3804	389.6938	777.3964	389.2018	**6**
**34**	3536.5693	1768.7883	3519.5427	1760.2750	3518.5587	1759.7830	**V**	***682.3229***	341.6651	665.2963	333.1518	664.3123	332.6598	**5**
**35**	3696.5999	1848.8036	3679.5734	1840.2903	3678.5894	1839.7983	**C**	***583.2545***	292.1309	566.2279	283.6176	565.2439	283.1256	**4**
**36**	3843.6684	1922.3378	3826.6418	1913.8245	3825.6578	1913.3325	**F**	423.2238	212.1155	406.1973	203.6023	405.2132	203.1103	**3**
**37**	3972.7109	1986.8591	3955.6844	1978.3458	3954.7004	1977.8538	**E**	276.1554	138.5813	259.1288	130.0681	258.1448	129.5761	**2**
**38**							**K**	***147.1128***	74.0600	130.0863	65.5468			**1**

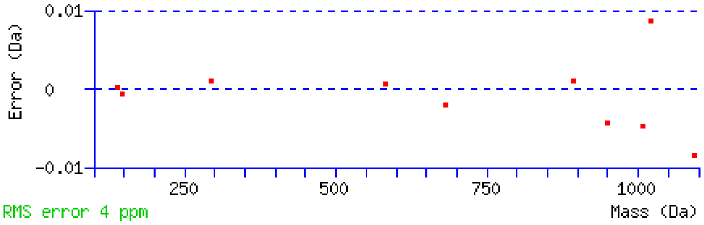

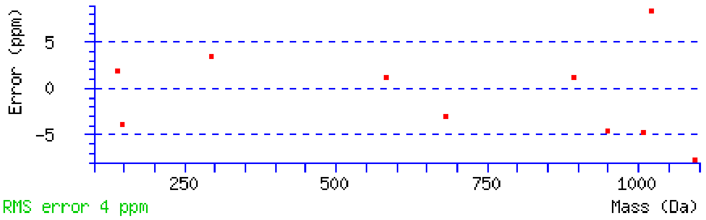

NCBI **BLAST** search of [YEFLNGRVQSTELCAGHLAGGTDSCQGDSGGPLVCFEK]
(Parameters: blastp, nr protein database, expect=20000, no filter, PAM30)
Other BLAST [web gateways]

All matches to this query

Score	Mr(calc)	Delta	Sequence
17.9	4117.8092	0.0137	YEFLNGRVQSTELCAGHLAGGTDSCQGDSGGPLVCFEK
12.6	4116.8252	0.9977	YEFLNGRVQSTELCAGHLAGGTDSCQGDSGGPLVCFEK
12.5	4116.8252	0.9977	YEFLNGRVQSTELCAGHLAGGTDSCQGDSGGPLVCFEK
12.5	4116.8252	0.9977	YEFLNGRVQSTELCAGHLAGGTDSCQGDSGGPLVCFEK
12.4	4117.8092	0.0137	YEFLNGRVQSTELCAGHLAGGTDSCQGDSGGPLVCFEK
12.4	4117.8092	0.0137	YEFLNGRVQSTELCAGHLAGGTDSCQGDSGGPLVCFEK

Mascot: http://www.matrixscience.com/

Mascot Search Results

Peptide View

MS/MS Fragmentation of **YEFLNGRVQSTELCAGHLAGGTDSCQGDSGGPLVCFEK**
Found in **P00747** in **uni_human_nr**, PLMN_HUMAN Plasminogen OS=Homo sapiens GN=PLG PE=1 SV=2

Match to Query 17111: 4117.822936 from(1030.463010,4+) intensity(752991.0625) rtinseconds(2874) scans(16785) index(12921)
Title: 130806_HPL_Human_Plaque_BR2_TR2_10_Spectrum033842_scans__16785_RTINSECONDS=2874
Data file E:\\RAW\\HumanPlaques\\mgf\\T\\1D\\T130806_HPL_Human_Plaque_BR2_TR2_10.mgf

Click mouse within plot area to zoom in by factor of two about that point
Or, Plot from 0 to 2600 Da Full range
Label all possible matches Label matches used for scoring
Show Y-axis

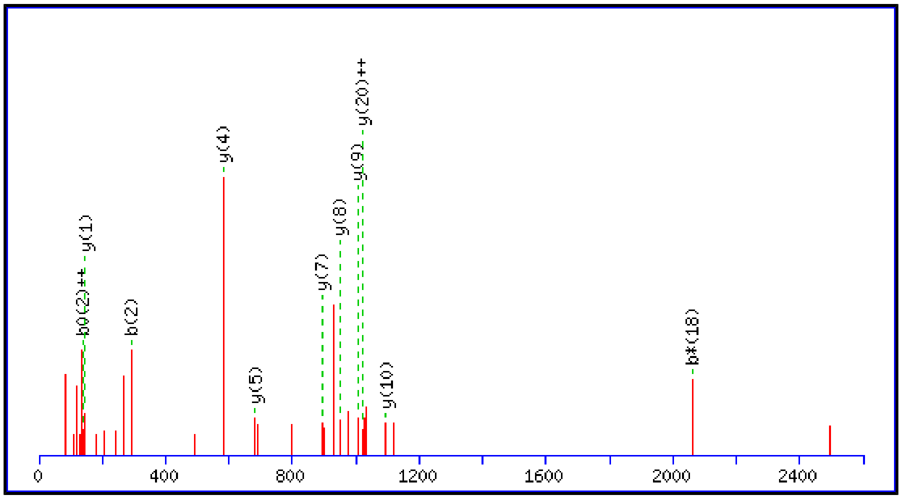

Monoisotopic mass of neutral peptide Mr(calc): 4116.8252
Fixed modifications: Carbamidomethyl (C) (apply to specified residues or termini only)
Variable modifications:
Q9 : Deamidated (NQ)
Ions Score: 18 **Expect:** 0.57
Matches : 11/432 fragment ions using 32 most intense peaks (help)

#	b	b^{++}	b*	b^{*++}	b^0	b^{0++}	Seq.	y	y^{++}	y*	y^{*++}	y^0	y^{0++}	#
1	164.0706	82.5389					Y							38
2	**293.1132**	147.0602			275.1026	138.0550	E	3954.7691	1977.8882	3937.7426	1969.3749	3936.7586	1968.8829	37
3	440.1816	220.5944			422.1710	211.5892	F	3825.7265	1913.3669	3808.7000	1904.8536	3807.7160	1904.3616	36
4	553.2657	277.1365			535.2551	268.1312	L	3678.6581	1839.8327	3661.6316	1831.3194	3660.6476	1830.8274	35
5	667.3086	334.1579	650.2821	325.6447	649.2980	325.1527	N	3565.5741	1783.2907	3548.5475	1774.7774	3547.5635	1774.2854	34
6	724.3301	362.6687	707.3035	354.1554	706.3195	353.6634	G	3451.5311	1726.2692	3434.5046	1717.7559	3433.5206	1717.2639	33
7	880.4312	440.7192	863.4046	432.2060	862.4206	431.7139	R	3394.5097	1697.7585	3377.4831	1689.2452	3376.4991	1688.7532	32
8	979.4996	490.2534	962.4730	481.7402	961.4890	481.2482	V	3238.4086	1619.7079	3221.3820	1611.1946	3220.3980	1610.7026	31
9	1108.5422	554.7747	1091.5156	546.2615	1090.5316	545.7694	Q	3139.3401	1570.1737	3122.3136	1561.6604	3121.3296	1561.1684	30
10	1195.5742	598.2907	1178.5477	589.7775	1177.5636	589.2855	S	3010.2976	1505.6524	2993.2710	1497.1391	2992.2870	1496.6471	29
11	1296.6219	648.8146	1279.5953	640.3013	1278.6113	639.8093	T	2923.2655	1462.1364	2906.2390	1453.6231	2905.2550	1453.1311	28
12	1425.6645	713.3359	1408.6379	704.8226	1407.6539	704.3306	E	2822.2178	1411.6126	2805.1913	1403.0993	2804.2073	1402.6073	27
13	1538.7486	769.8779	1521.7220	761.3646	1520.7380	760.8726	L	2693.1753	1347.0913	2676.1487	1338.5780	2675.1647	1338.0860	26
14	1698.7792	849.8932	1681.7527	841.3800	1680.7686	840.8880	C	2580.0912	1290.5492	2563.0646	1282.0360	2562.0806	1281.5439	25
15	1769.8163	885.4118	1752.7898	876.8985	1751.8057	876.4065	A	2420.0605	1210.5339	2403.0340	1202.0206	2402.0500	1201.5286	24
16	1826.8378	913.9225	1809.8112	905.4093	1808.8272	904.9172	G	2349.0234	1175.0153	2331.9969	1166.5021	2331.0129	1166.0101	23
17	1963.8967	982.4520	1946.8701	973.9387	1945.8861	973.4467	H	2292.0020	1146.5046	2274.9754	1137.9913	2273.9914	1137.4993	22
18	2076.9808	1038.9940	2059.9542	1030.4807	2058.9702	1029.9887	L	2154.9430	1077.9752	2137.9165	1069.4619	2136.9325	1068.9699	21

19	2148.0179	1074.5126	2130.9913	1065.9993	2130.0073	1065.5073	A	2041.8590	1021.4331	2024.8324	1012.9199	2023.8484	1012.4278	20
20	2205.0393	1103.0233	2188.0128	1094.5100	2187.0288	1094.0180	G	1970.8219	985.9146	1953.7953	977.4013	1952.8113	976.9093	19
21	2262.0608	1131.5340	2245.0342	1123.0208	2244.0502	1122.5288	G	1913.8004	957.4038	1896.7739	948.8906	1895.7898	948.3986	18
22	2363.1085	1182.0579	2346.0819	1173.5446	2345.0979	1173.0526	T	1856.7789	928.8931	1839.7524	920.3798	1838.7684	919.8878	17
23	2478.1354	1239.5713	2461.1089	1231.0581	2460.1249	1230.5661	D	1755.7313	878.3693	1738.7047	869.8560	1737.7207	869.3640	16
24	2565.1674	1283.0874	2548.1409	1274.5741	2547.1569	1274.0821	S	1640.7043	820.8558	1623.6778	812.3425	1622.6938	811.8505	15
25	2725.1981	1363.1027	2708.1715	1354.5894	2707.1875	1354.0974	C	1553.6723	777.3398	1536.6457	768.8265	1535.6617	768.3345	14
26	2853.2567	1427.1320	2836.2301	1418.6187	2835.2461	1418.1267	Q	1393.6416	697.3245	1376.6151	688.8112	1375.6311	688.3192	13
27	2910.2781	1455.6427	2893.2516	1447.1294	2892.2676	1446.6374	G	1265.5831	633.2952	1248.5565	624.7819	1247.5725	624.2899	12
28	3025.3051	1513.1562	3008.2785	1504.6429	3007.2945	1504.1509	D	1208.5616	604.7844	1191.5351	596.2712	1190.5510	595.7792	11
29	3112.3371	1556.6722	3095.3106	1548.1589	3094.3265	1547.6669	S	***1093.5347***	547.2710	1076.5081	538.7577	1075.5241	538.2657	10
30	3169.3586	1585.1829	3152.3320	1576.6696	3151.3480	1576.1776	G	***1006.5026***	503.7550	989.4761	495.2417	988.4921	494.7497	9
31	3226.3800	1613.6937	3209.3535	1605.1804	3208.3695	1604.6884	G	***949.4812***	475.2442	932.4546	466.7309	931.4706	466.2389	8
32	3323.4328	1662.2200	3306.4062	1653.7068	3305.4222	1653.2148	P	***892.4597***	446.7335	875.4332	438.2202	874.4491	437.7282	7
33	3436.5169	1718.7621	3419.4903	1710.2488	3418.5063	1709.7568	L	795.4069	398.2071	778.3804	389.6938	777.3964	389.2018	6
34	3535.5853	1768.2963	3518.5587	1759.7830	3517.5747	1759.2910	V	***682.3229***	341.6651	665.2963	333.1518	664.3123	332.6598	5
35	3695.6159	1848.3116	3678.5894	1839.7983	3677.6054	1839.3063	C	***583.2545***	292.1309	566.2279	283.6176	565.2439	283.1256	4
36	3842.6843	1921.8458	3825.6578	1913.3325	3824.6738	1912.8405	F	423.2238	212.1155	406.1973	203.6023	405.2132	203.1103	3
37	3971.7269	1986.3671	3954.7004	1977.8538	3953.7164	1977.3618	E	276.1554	138.5813	259.1288	130.0681	258.1448	129.5761	2
38							K	***147.1128***	74.0600	130.0863	65.5468			1

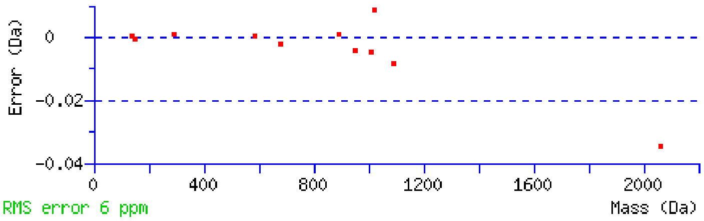

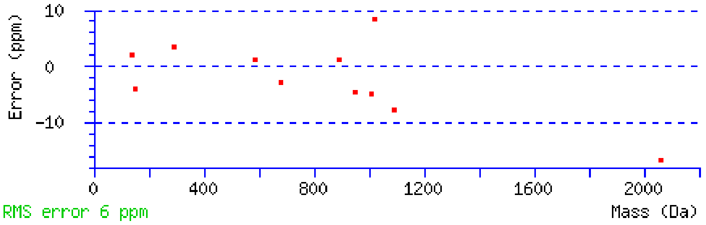

NCBI **BLAST** search of YEFLNGRVQSTELCAGHLAGGTDSCQGDSGGPLVCFEK
(Parameters: blastp, nr protein database, expect=20000, no filter, PAM30)
Other BLAST web gateways

All matches to this query

Score	Mr(calc)	Delta	Sequence
17.9	4116.8252	0.9977	YEFLNGRVQSTELCAGHLAGGTDSCQGDSGGPLVCFEK
17.9	4116.8252	0.9977	YEFLNGRVQSTELCAGHLAGGTDSCQGDSGGPLVCFEK
17.9	4117.8092	0.0137	YEFLNGRVQSTELCAGHLAGGTDSCQGDSGGPLVCFEK
12.8	4117.8092	0.0137	YEFLNGRVQSTELCAGHLAGGTDSCQGDSGGPLVCFEK
12.8	4117.8092	0.0137	YEFLNGRVQSTELCAGHLAGGTDSCQGDSGGPLVCFEK
12.6	4116.8252	0.9977	YEFLNGRVQSTELCAGHLAGGTDSCQGDSGGPLVCFEK

Mascot: http://www.matrixscience.com/

Mascot Search Results

Peptide View

MS/MS Fragmentation of **SLLDLENGR**
Found in **O60437** in **uni_human_nr**, PEPL_HUMAN Periplakin OS=Homo sapiens GN=PPL PE=1 SV=4

Match to Query 457: 1016.513888 from(509.264220,2+) intensity(463070.1250) rtinseconds(2004) scans(10725) index(7830)
Title: 130801_HPL_Human_Plaque_BR2_TR1_09_Spectrum028642_scans__10725_RTINSECONDS=2004
Data file E:\\RAW\\HumanPlaques\\mgf\\T\\1D\\T130801_HPL_Human_Plaque_BR2_TR1_09.mgf

Click mouse within plot area to zoom in by factor of two about that point
Or, Plot from 50 to 850 Da Full range
Label all possible matches ○ Label matches used for scoring ◉
Show Y-axis ☐

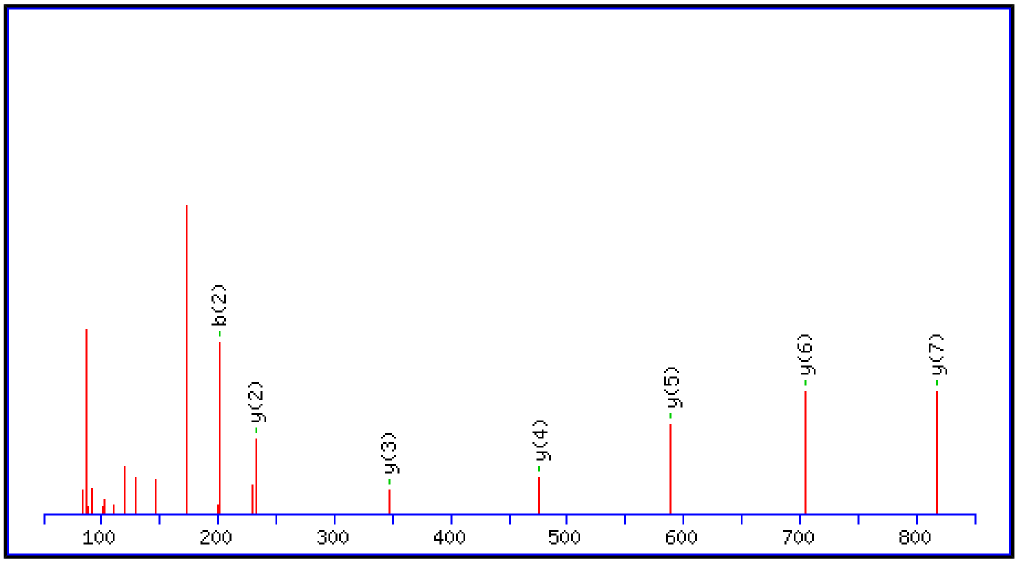

Monoisotopic mass of neutral peptide Mr(calc): 1016.5138
Fixed modifications: Carbamidomethyl (C) (apply to specified residues or termini only)
Variable modifications:
N7 : Deamidated (NQ)
Ions Score: 53 **Expect:** 0.00025
Matches : 7/78 fragment ions using 9 most intense peaks (help)

#	b	b^{++}	b*	b^{*++}	b^0	b^{0++}	Seq.	y	y^{++}	y*	y^{*++}	y^0	y^{0++}	#
1	88.0393	44.5233			70.0287	35.5180	S							9
2	**201.1234**	101.0653			183.1128	92.0600	L	930.4891	465.7482	913.4625	457.2349	912.4785	456.7429	8
3	314.2074	157.6074			296.1969	148.6021	L	***817.4050***	409.2061	800.3785	400.6929	799.3945	400.2009	7
4	429.2344	215.1208			411.2238	206.1155	D	***704.3210***	352.6641	687.2944	344.1508	686.3104	343.6588	6
5	542.3184	271.6629			524.3079	262.6576	L	***589.2940***	295.1506	572.2675	286.6374	571.2835	286.1454	5
6	671.3610	336.1842			653.3505	327.1789	E	***476.2100***	238.6086	459.1834	230.0953	458.1994	229.6033	4
7	786.3880	393.6976	769.3614	385.1844	768.3774	384.6923	N	***347.1674***	174.0873	330.1408	165.5740			3
8	843.4094	422.2084	826.3829	413.6951	825.3989	413.2031	G	***232.1404***	116.5738	215.1139	108.0606			2
9							R	175.1190	88.0631	158.0924	79.5498			1

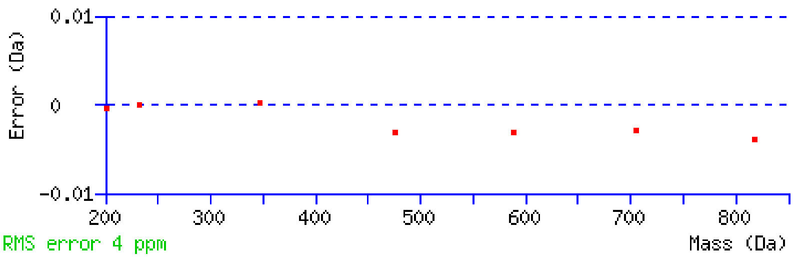

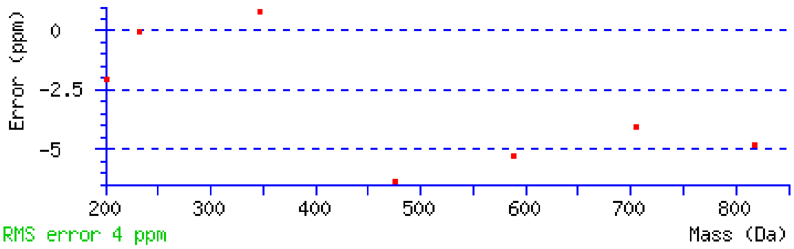

NCBI **BLAST** search of SLLDLENGR
(Parameters: blastp, nr protein database, expect=20000, no filter, PAM30)
Other BLAST web gateways

All matches to this query

Score	Mr(calc)	Delta	Sequence
53.3	1016.5138	0.0001	SLLDLENGR
0.6	1016.5138	0.0001	SLEDEIVGR

Mascot: http://www.matrixscience.com/

Mascot Search Results

Peptide View

MS/MS Fragmentation of **EVVEEAENGR**
Found in **P06454** in **uni_human_nr**, PTMA_HUMAN Prothymosin alpha OS=Homo sapiens GN=PTMA PE=1 SV=2

Match to Query 1171: 1131.504848 from(566.759700,2+) intensity(840734.6250) rtinseconds(891) scans(3064) index(1096)
Title: 130801_HPL_Human_Plaque_BR2_TR1_19_Spectrum019345_scans__3064_RTINSECONDS=891
Data file E:\\RAW\\HumanPlaques\\mgf\\T\\1D\\T130801_HPL_Human_Plaque_BR2_TR1_19.mgf

Click mouse within plot area to zoom in by factor of two about that point
Or, [Plot from] [50] to [950] Da [Full range]
Label all possible matches ○ Label matches used for scoring ⦿
Show Y-axis ☐

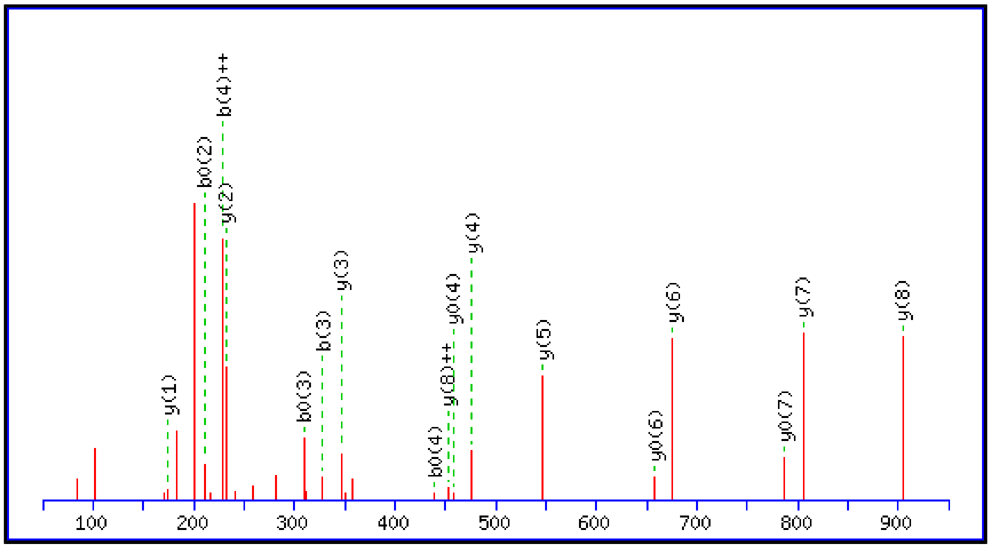

Monoisotopic mass of neutral peptide Mr(calc): 1131.5044
Fixed modifications: Carbamidomethyl (C) (apply to specified residues or termini only)
Variable modifications:
N8 : Deamidated (NQ)
Ions Score: 72 **Expect:** 1.2e-006
Matches : 18/88 fragment ions using 22 most intense peaks (help)

#	b	b^{++}	b*	b^{*++}	b^0	b^{0++}	Seq.	y	y^{++}	y*	y^{*++}	y^0	y^{0++}	#
1	130.0499	65.5286			112.0393	56.5233	E							10
2	**229.1183**	115.0628			211.1077	106.0575	V	1003.4691	502.2382	986.4425	493.7249	985.4585	493.2329	9
3	**328.1867**	164.5970			310.1761	155.5917	V	***904.4007***	452.7040	887.3741	444.1907	886.3901	443.6987	8
4	457.2293	**229.1183**			439.2187	220.1130	E	***805.3323***	403.1698	788.3057	394.6565	787.3217	394.1645	7
5	586.2719	293.6396			568.2613	284.6343	E	***676.2897***	338.6485	659.2631	330.1352	658.2791	329.6432	6
6	657.3090	329.1581			639.2984	320.1529	A	***547.2471***	274.1272	530.2205	265.6139	529.2365	265.1219	5
7	786.3516	393.6794			768.3410	384.6742	E	***476.2100***	238.6086	459.1834	230.0953	458.1994	229.6033	4
8	901.3785	451.1929	884.3520	442.6796	883.3680	442.1876	N	***347.1674***	174.0873	330.1408	165.5740			3
9	958.4000	479.7036	941.3734	471.1904	940.3894	470.6984	G	***232.1404***	116.5738	215.1139	108.0606			2
10							R	***175.1190***	88.0631	158.0924	79.5498			1

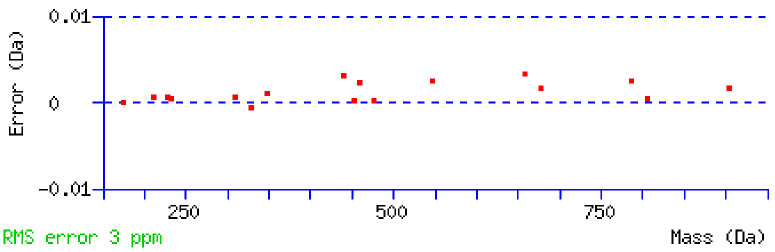

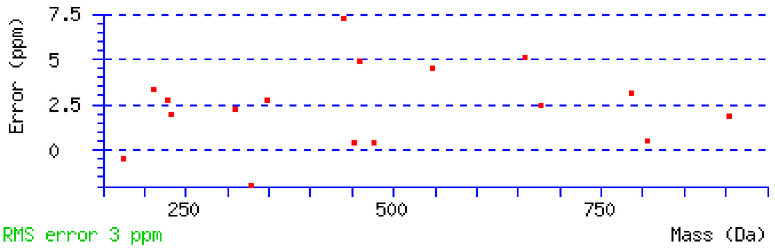

NCBI **BLAST** search of EVVEEAENGR
(Parameters: blastp, nr protein database, expect=20000, no filter, PAM30)
Other BLAST web gateways

All matches to this query

Score	Mr(calc)	Delta	Sequence
72.5	1131.5044	0.0005	EVVEEAENGR
8.3	1131.5044	0.0005	DIVNQVGDNR

Mascot: http://www.matrixscience.com/

Mascot Search Results

Peptide View

MS/MS Fragmentation of **EVVEEAENGR**
Found in **P06454** in **uni_human_nr**, PTMA_HUMAN Prothymosin alpha OS=Homo sapiens GN=PTMA PE=1 SV=2

Match to Query 794: 1130.520488 from(566.267520,2+) intensity(1215421.5000) rtinseconds(888) scans(3149) index(1284)
Title: 130806_HPL_Human_Plaque_BR1_TR2_16_Spectrum021305_scans__3149_RTINSECONDS=888
Data file E:\\RAW\\HumanPlaques\\mgf\\T\\1D\\T130806_HPL_Human_Plaque_BR1_TR2_16.mgf

Click mouse within plot area to zoom in by factor of two about that point
Or, [Plot from] 50 to 950 Da [Full range]
Label all possible matches ○ Label matches used for scoring ⦿
Show Y-axis ☐

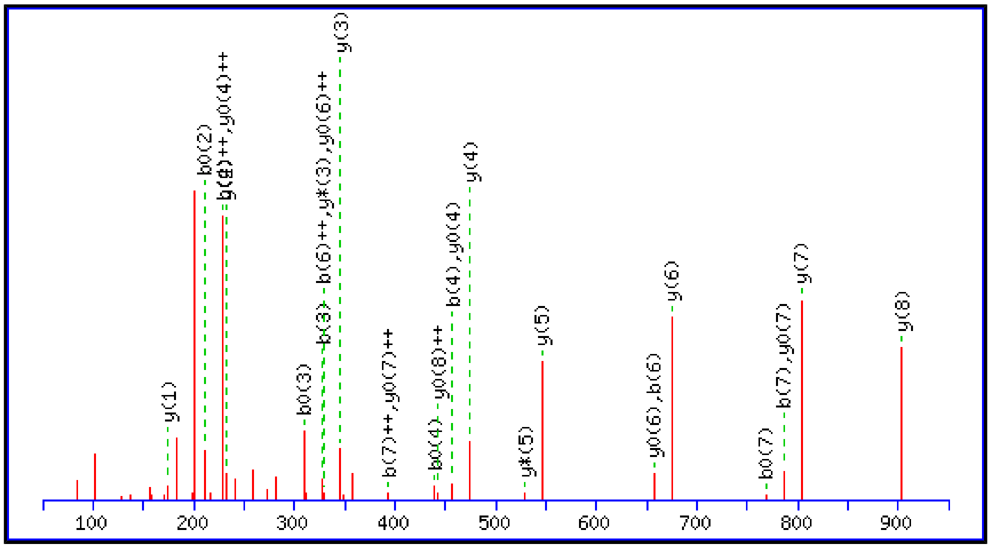

Monoisotopic mass of neutral peptide Mr(calc): 1130.5204
Fixed modifications: Carbamidomethyl (C) (apply to specified residues or termini only)
Ions Score: 69 **Expect:** 4.2e-006
Matches : 29/88 fragment ions using 29 most intense peaks (help)

#	b	b^{++}	b*	b^{*++}	b^{0}	b^{0++}	Seq.	y	y^{++}	y*	y^{*++}	y^{0}	y^{0++}	#
1	130.0499	65.5286			112.0393	56.5233	E							10
2	**229.1183**	115.0628			211.1077	106.0575	V	1002.4851	501.7462	985.4585	493.2329	984.4745	492.7409	9
3	**328.1867**	164.5970			310.1761	155.5917	V	***903.4167***	452.2120	886.3901	443.6987	885.4061	443.2067	8
4	**457.2293**	**229.1183**			439.2187	220.1130	E	***804.3482***	402.6778	787.3217	394.1645	786.3377	393.6725	7
5	586.2719	293.6396			568.2613	284.6343	E	***675.3056***	338.1565	658.2791	329.6432	657.2951	329.1512	6
6	**657.3090**	**329.1581**			639.2984	320.1529	A	***546.2631***	273.6352	529.2365	265.1219	528.2525	264.6299	5
7	**786.3516**	**393.6794**			768.3410	384.6742	E	***475.2259***	238.1166	458.1994	229.6033	457.2154	229.1113	4
8	900.3945	450.7009	883.3680	442.1876	882.3840	441.6956	N	***346.1833***	173.5953	329.1568	165.0820			3
9	957.4160	479.2116	940.3894	470.6984	939.4054	470.2063	G	***232.1404***	116.5738	215.1139	108.0606			2
10							R	***175.1190***	88.0631	158.0924	79.5498			1

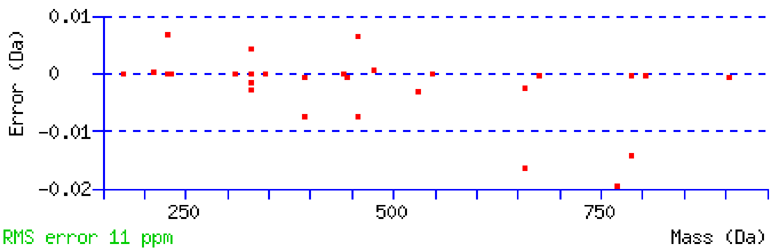

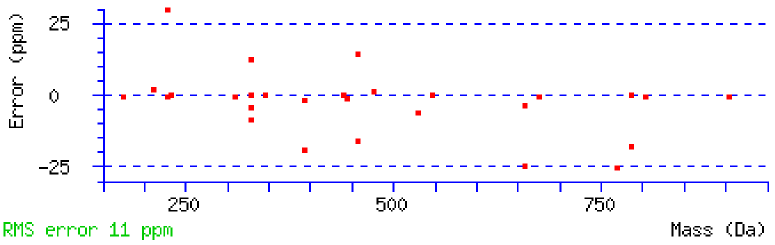

NCBI **BLAST** search of EVVEEAENGR
(Parameters: blastp, nr protein database, expect=20000, no filter, PAM30)
Other BLAST web gateways

All matches to this query

Score	Mr(calc)	Delta	Sequence
68.8	1130.5204	0.0001	EVVEEAENGR
9.0	1130.5204	0.0001	DIVNQVGDNR
9.0	1130.5204	0.0001	DIVNQVGDNR
7.7	1130.5204	0.0001	DIVNQVGDNR

Mascot: http://www.matrixscience.com/

Mascot Search Results

Peptide View

MS/MS Fragmentation of **KEVVEEAENGR**
Found in **P06454** in **uni_human_nr**, PTMA_HUMAN Prothymosin alpha OS=Homo sapiens GN=PTMA PE=1 SV=2

Match to Query 1897: 1258.614942 from(420.545590,3+) intensity(119168.4063) rtinseconds(720) scans(2328) index(794)
Title: 130809_HPL_Human_Plaque_BR1_TR3_09_Spectrum022583_scans__2328_RTINSECONDS=720
Data file E:\\RAW\\HumanPlaques\\mgf\\T\\1D\\T130809_HPL_Human_Plaque_BR1_TR3_09.mgf

Click mouse within plot area to zoom in by factor of two about that point
Or, Plot from 50 to 750 Da Full range
Label all possible matches ○ Label matches used for scoring ⊙
Show Y-axis ☐

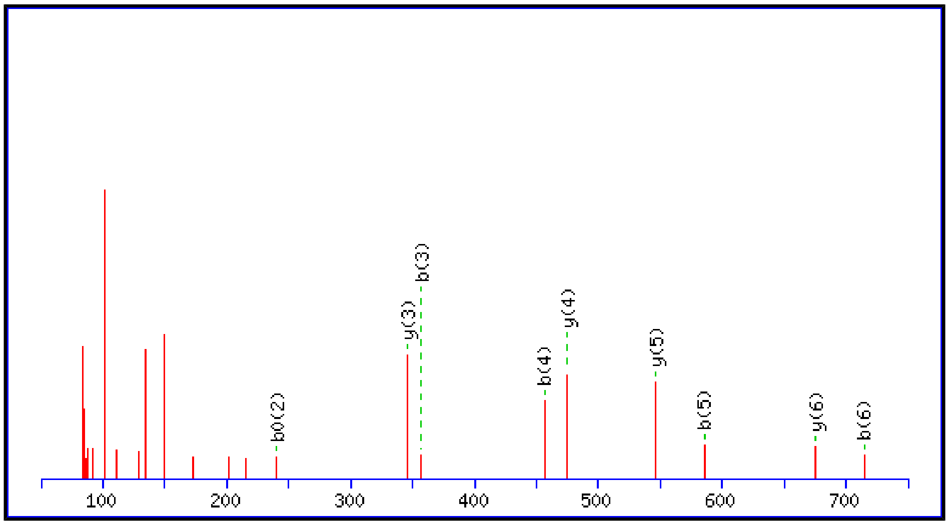

Monoisotopic mass of neutral peptide Mr(calc): 1258.6153
Fixed modifications: Carbamidomethyl (C) (apply to specified residues or termini only)
Ions Score: 35 **Expect:** 0.02
Matches : 9/112 fragment ions using 12 most intense peaks (help)

#	b	b^{++}	b*	b^{*++}	b^{0}	b^{0++}	Seq.	y	y^{++}	y*	y^{*++}	y^{0}	y^{0++}	#
1	129.1022	65.0548	112.0757	56.5415			K							11
2	258.1448	129.5761	241.1183	121.0628	240.1343	120.5708	E	1131.5277	566.2675	1114.5011	557.7542	1113.5171	557.2622	10
3	***357.2132***	179.1103	340.1867	170.5970	339.2027	170.1050	V	1002.4851	501.7462	985.4585	493.2329	984.4745	492.7409	9
4	***456.2817***	228.6445	439.2551	220.1312	438.2711	219.6392	V	903.4167	452.2120	886.3901	443.6987	885.4061	443.2067	8
5	***585.3243***	293.1658	568.2977	284.6525	567.3137	284.1605	E	804.3482	402.6778	787.3217	394.1645	786.3377	393.6725	7
6	***714.3668***	357.6871	697.3403	349.1738	696.3563	348.6818	E	***675.3056***	338.1565	658.2791	329.6432	657.2951	329.1512	6
7	785.4040	393.2056	768.3774	384.6923	767.3934	384.2003	A	***546.2631***	273.6352	529.2365	265.1219	528.2525	264.6299	5
8	914.4466	457.7269	897.4200	449.2136	896.4360	448.7216	E	***475.2259***	238.1166	458.1994	229.6033	457.2154	229.1113	4
9	1028.4895	514.7484	1011.4629	506.2351	1010.4789	505.7431	N	***346.1833***	173.5953	329.1568	165.0820			3
10	1085.5109	543.2591	1068.4844	534.7458	1067.5004	534.2538	G	232.1404	116.5738	215.1139	108.0606			2
11							R	175.1190	88.0631	158.0924	79.5498			1

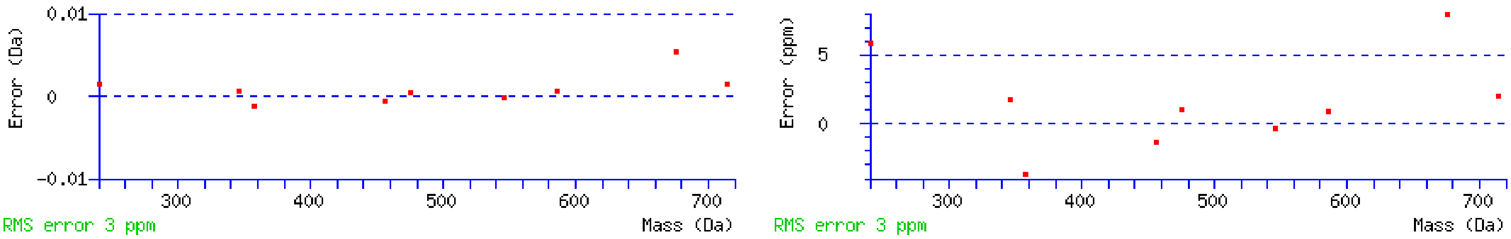

NCBI **BLAST** search of KEVVEEAENGR
(Parameters: blastp, nr protein database, expect=20000, no filter, PAM30)
Other BLAST web gateways

All matches to this query

Score	Mr(calc)	Delta	Sequence
34.5	1258.6153	-0.0004	KEVVEEAENGR

Mascot: http://www.matrixscience.com/

Mascot Search Results

Peptide View

MS/MS Fragmentation of **KEDGGWWEGQINGR**
Found in **Q96B97** in **uni_human_nr**, SH3K1_HUMAN SH3 domain-containing kinase-binding protein 1 OS=Homo sapiens GN=SH3KBP1 PE=1 SV=2

Match to Query 4047: 1631.737572 from(544.919800,3+) intensity(345093.9375) rtinseconds(1960) scans(10641) index(7761)
Title: 130806_HPL_Human_Plaque_BR1_TR2_11_Spectrum028797_scans__10641_RTINSECONDS=1960
Data file E:\\RAW\\HumanPlaques\\mgf\\T\\1D\\T130806_HPL_Human_Plaque_BR1_TR2_11.mgf

Click mouse within plot area to zoom in by factor of two about that point
Or, [Plot from] [50] to [900] Da [Full range]
Label all possible matches ○ Label matches used for scoring ⦿
Show Y-axis ☐

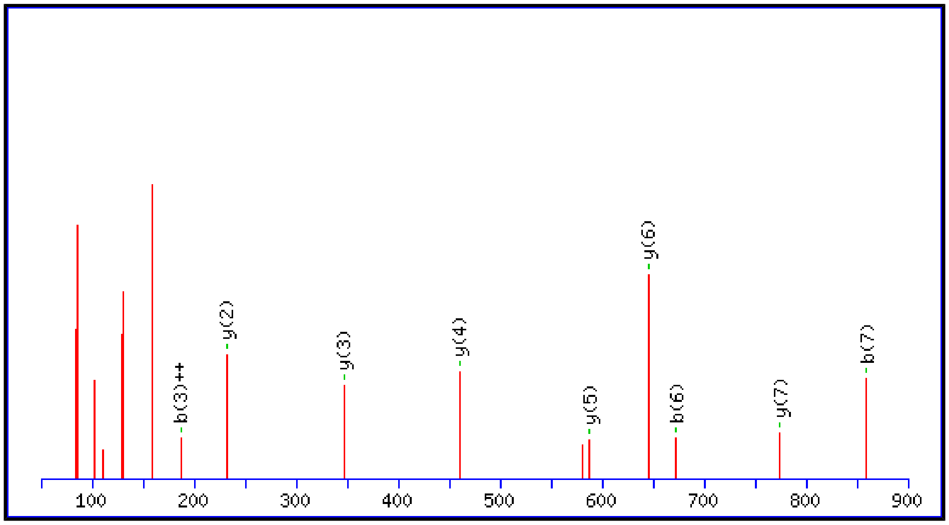

Monoisotopic mass of neutral peptide Mr(calc): 1631.7328
Fixed modifications: Carbamidomethyl (C) (apply to specified residues or termini only)
Variable modifications:
N12 : Deamidated (NQ)
Ions Score: 41 **Expect:** 0.0031
Matches : 9/142 fragment ions using 13 most intense peaks (help)

#	b	b^{++}	b*	b^{*++}	b^0	b^{0++}	Seq.	y	y^{++}	y*	y^{*++}	y^0	y^{0++}	#
1	129.1022	65.0548	112.0757	56.5415			K							14
2	258.1448	129.5761	241.1183	121.0628	240.1343	120.5708	E	1504.6451	752.8262	1487.6186	744.3129	1486.6346	743.8209	13
3	373.1718	**187.0895**	356.1452	178.5763	355.1612	178.0842	D	1375.6026	688.3049	1358.5760	679.7916	1357.5920	679.2996	12
4	430.1932	215.6003	413.1667	207.0870	412.1827	206.5950	G	1260.5756	630.7914	1243.5491	622.2782	1242.5650	621.7862	11
5	487.2147	244.1110	470.1882	235.5977	469.2041	235.1057	G	1203.5541	602.2807	1186.5276	593.7674	1185.5436	593.2754	10
6	**673.2940**	337.1506	656.2675	328.6374	655.2835	328.1454	W	1146.5327	573.7700	1129.5061	565.2567	1128.5221	564.7647	9
7	**859.3733**	430.1903	842.3468	421.6770	841.3628	421.1850	W	960.4534	480.7303	943.4268	472.2170	942.4428	471.7250	8
8	988.4159	494.7116	971.3894	486.1983	970.4054	485.7063	E	***774.3741***	387.6907	757.3475	379.1774	756.3635	378.6854	7
9	1045.4374	523.2223	1028.4108	514.7091	1027.4268	514.2170	G	***645.3315***	323.1694	628.3049	314.6561			6
10	1173.4960	587.2516	1156.4694	578.7383	1155.4854	578.2463	Q	***588.3100***	294.6586	571.2835	286.1454			5
11	1286.5800	643.7937	1269.5535	635.2804	1268.5695	634.7884	I	***460.2514***	230.6293	443.2249	222.1161			4
12	1401.6070	701.3071	1384.5804	692.7938	1383.5964	692.3018	N	***347.1674***	174.0873	330.1408	165.5740			3
13	1458.6284	729.8179	1441.6019	721.3046	1440.6179	720.8126	G	***232.1404***	116.5738	215.1139	108.0606			2
14							R	175.1190	88.0631	158.0924	79.5498			1

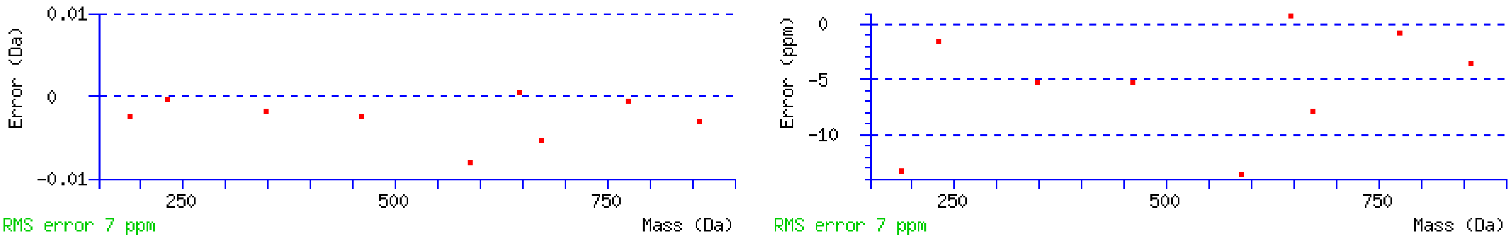

NCBI **BLAST** search of KEDGGWWEGQINGR
(Parameters: blastp, nr protein database, expect=20000, no filter, PAM30)
Other BLAST web gateways

All matches to this query

Score	Mr(calc)	Delta	Sequence	Site Analysis
40.9	1631.7328	0.0048	KEDGGWWEGQINGR	Deamidated N12 98.96%
21.1	1631.7328	0.0048	KEDGGWWEGQINGR	Deamidated Q10 1.04%
1.1	1629.7271	2.0105	YFGSIDSSEAEAGAAR	

Mascot: http://www.matrixscience.com/

Mascot Search Results

Peptide View

MS/MS Fragmentation of **KEDGGWWEGQINGR**
Found in **Q96B97** in **uni_human_nr**, SH3K1_HUMAN SH3 domain-containing kinase-binding protein 1 OS=Homo sapiens GN=SH3KBP1 PE=1 SV=2

Match to Query 5173: 1630.750992 from(544.590940,3+) intensity(208488.5781) rtinseconds(1799) scans(10073) index(7566)
Title: 130809_HPL_Human_Plaque_BR2_TR3_06_Spectrum029606_scans__10073_RTINSECONDS=1799
Data file E:\\RAW\\HumanPlaques\\mgf\\T\\1D\\T130809_HPL_Human_Plaque_BR2_TR3_06.mgf

Click mouse within plot area to zoom in by factor of two about that point
Or, Plot from 0 to 1100 Da Full range
Label all possible matches ○ Label matches used for scoring ⦿
Show Y-axis ☐

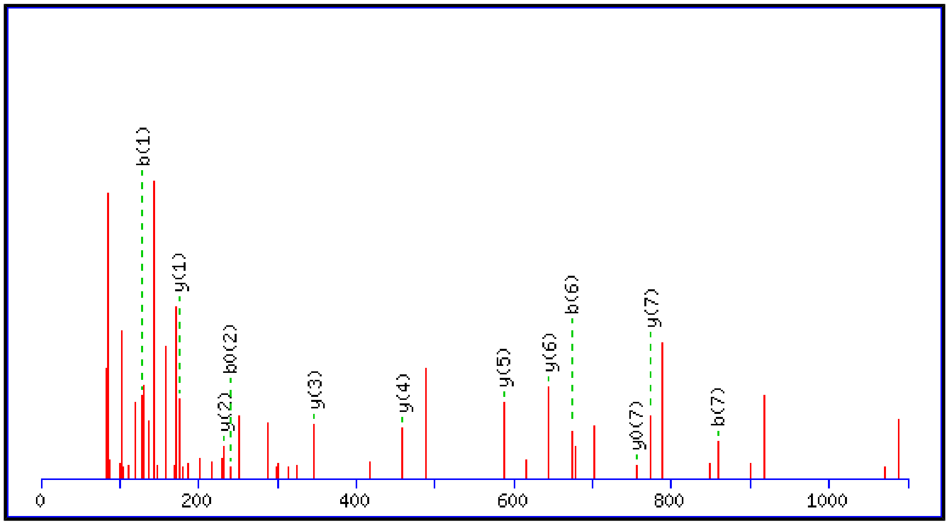

Monoisotopic mass of neutral peptide Mr(calc): 1630.7488
Fixed modifications: Carbamidomethyl (C) (apply to specified residues or termini only)
Ions Score: 17 **Expect:** 0.82
Matches : 12/142 fragment ions using 43 most intense peaks (help)

#	b	b^{++}	b*	b^{*++}	b^{0}	b^{0++}	Seq.	y	y^{++}	y*	y^{*++}	y^{0}	y^{0++}	#
1	**129.1022**	65.0548	112.0757	56.5415			K							14
2	258.1448	129.5761	241.1183	121.0628	240.1343	120.5708	E	1503.6611	752.3342	1486.6346	743.8209	1485.6506	743.3289	13
3	373.1718	187.0895	356.1452	178.5763	355.1612	178.0842	D	1374.6185	687.8129	1357.5920	679.2996	1356.6080	678.8076	12
4	430.1932	215.6003	413.1667	207.0870	412.1827	206.5950	G	1259.5916	630.2994	1242.5650	621.7862	1241.5810	621.2942	11
5	487.2147	244.1110	470.1882	235.5977	469.2041	235.1057	G	1202.5701	601.7887	1185.5436	593.2754	1184.5596	592.7834	10
6	**673.2940**	337.1506	656.2675	328.6374	655.2835	328.1454	W	1145.5487	573.2780	1128.5221	564.7647	1127.5381	564.2727	9
7	**859.3733**	430.1903	842.3468	421.6770	841.3628	421.1850	W	959.4694	480.2383	942.4428	471.7250	941.4588	471.2330	8
8	988.4159	494.7116	971.3894	486.1983	970.4054	485.7063	E	***773.3900***	387.1987	756.3635	378.6854	755.3795	378.1934	7
9	1045.4374	523.2223	1028.4108	514.7091	1027.4268	514.2170	G	***644.3474***	322.6774	627.3209	314.1641			6
10	1173.4960	587.2516	1156.4694	578.7383	1155.4854	578.2463	Q	***587.3260***	294.1666	570.2994	285.6534			5
11	1286.5800	643.7937	1269.5535	635.2804	1268.5695	634.7884	I	***459.2674***	230.1373	442.2409	221.6241			4
12	1400.6230	700.8151	1383.5964	692.3018	1382.6124	691.8098	N	***346.1833***	173.5953	329.1568	165.0820			3
13	1457.6444	729.3258	1440.6179	720.8126	1439.6339	720.3206	G	***232.1404***	116.5738	215.1139	108.0606			2
14							R	***175.1190***	88.0631	158.0924	79.5498			1

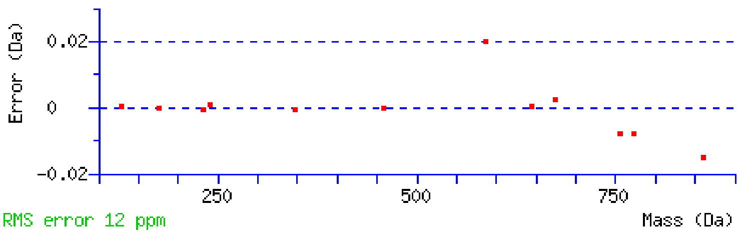

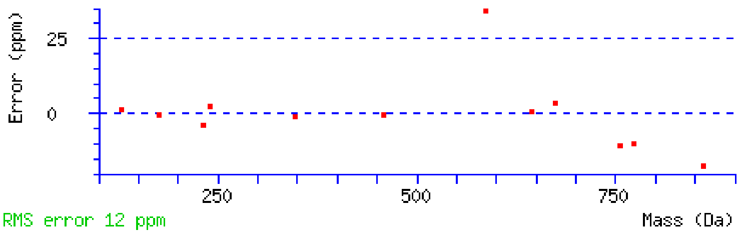

NCBI **BLAST** search of KEDGGWWEGQINGR
(Parameters: blastp, nr protein database, expect=20000, no filter, PAM30)
Other BLAST web gateways

All matches to this query

Score	Mr(calc)	Delta	Sequence
17.5	1630.7488	0.0022	KEDGGWWEGQINGR

Mascot: http://www.matrixscience.com/

Mascot Search Results

Peptide View

MS/MS Fragmentation of **FMNVPGGGAAAVMMTGYNNGR**
Found in **H0Y7S5** in **uni_human_nr**, H0Y7S5_HUMAN SH3 and multiple ankyrin repeat domains protein 2 (Fragment) OS=Homo sapiens GN=SHANK2 PE=1 SV=1

Match to Query 8089: 2132.905842 from(711.975890,3+) intensity(355070.5000) rtinseconds(2625) scans(15442) index(11653)
Title: 130806_HPL_Human_Plaque_BR1_TR2_08_Spectrum033345_scans__15442_RTINSECONDS=2625
Data file E:\\RAW\\HumanPlaques\\mgf\\T\\1D\\T130806_HPL_Human_Plaque_BR1_TR2_08.mgf

Click mouse within plot area to zoom in by factor of two about that point
Or, [Plot from] [0] to [1300] Da [Full range]
Label all possible matches ○ Label matches used for scoring ⊙
Show Y-axis ☐

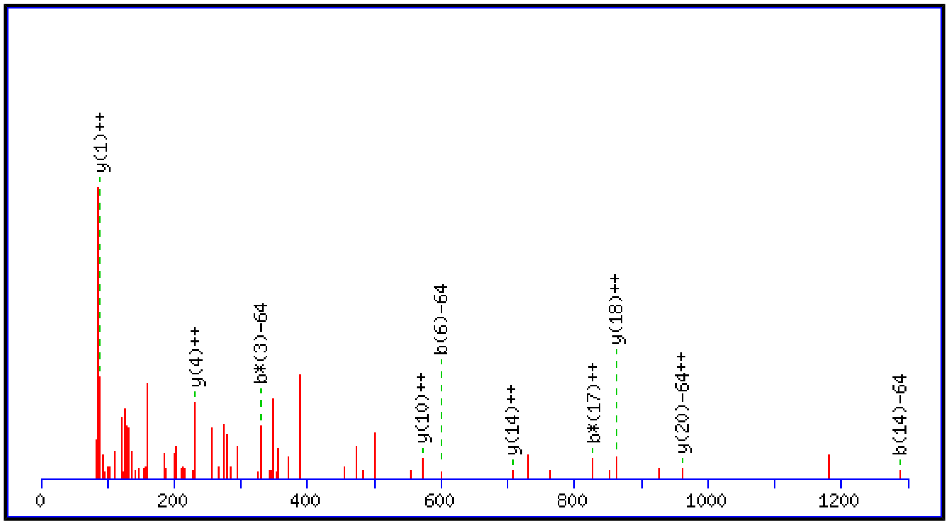


```
Monoisotopic mass of neutral peptide Mr(calc): 2131.9125
Fixed modifications: Carbamidomethyl (C) (apply to specified residues or termini only)
Variable modifications:
M2     : Oxidation (M), with neutral losses 63.9983(shown in table), 0.0000
N3     : Deamidated (NQ)
N19    : Deamidated (NQ)
Ions Score: 22  Expect: 0.13
Matches : 10/288 fragment ions using 19 most intense peaks   (help)
```

#	b	b++	b*	b*++	b0	b0++	Seq.	y	y++	y*	y*++	y0	y0++	#
1	148.0757	74.5415					F							21
2	231.1128	116.0600					M	1921.8531	961.4302	1904.8266	952.9169	1903.8425	952.4249	20
3	346.1397	173.5735	329.1132	165.0602			N	1838.8160	919.9116	1821.7894	911.3984	1820.8054	910.9064	19
4	445.2082	223.1077	428.1816	214.5944			V	1723.7891	862.3982	1706.7625	853.8849	1705.7785	853.3929	18
5	542.2609	271.6341	525.2344	263.1208			P	1624.7206	812.8640	1607.6941	804.3507	1606.7101	803.8587	17
6	**599.2824**	300.1448	582.2558	291.6316			G	1527.6679	764.3376	1510.6413	755.8243	1509.6573	755.3323	16
7	656.3039	328.6556	639.2773	320.1423			G	1470.6464	735.8268	1453.6199	727.3136	1452.6358	726.8216	15
8	713.3253	357.1663	696.2988	348.6530			G	1413.6249	707.3161	1396.5984	698.8028	1395.6144	698.3108	14
9	784.3624	392.6849	767.3359	384.1716			A	1356.6035	678.8054	1339.5769	670.2921	1338.5929	669.8001	13
10	855.3995	428.2034	838.3730	419.6901			A	1285.5664	643.2868	1268.5398	634.7735	1267.5558	634.2815	12
11	926.4367	463.7220	909.4101	455.2087			A	1214.5293	607.7683	1197.5027	599.2550	1196.5187	598.7630	11
12	1025.5051	513.2562	1008.4785	504.7429			V	1143.4921	572.2497	1126.4656	563.7364	1125.4816	563.2444	10
13	1156.5456	578.7764	1139.5190	570.2631			M	1044.4237	522.7155	1027.3972	514.2022	1026.4132	513.7102	9

**14**	**1287.5860**	644.2967	1270.5595	635.7834			**M**	913.3832	457.1953	896.3567	448.6820	895.3727	448.1900	**8**
**15**	1388.6337	694.8205	1371.6072	686.3072	1370.6232	685.8152	**T**	782.3428	391.6750	765.3162	383.1617	764.3322	382.6697	**7**
**16**	1445.6552	723.3312	1428.6286	714.8180	1427.6446	714.3259	**G**	681.2951	341.1512	664.2685	332.6379			**6**
**17**	1608.7185	804.8629	1591.6920	796.3496	1590.7080	795.8576	**Y**	624.2736	312.6404	607.2471	304.1272			**5**
**18**	1722.7614	861.8844	1705.7349	853.3711	1704.7509	852.8791	**N**	461.2103	231.1088	444.1837	222.5955			**4**
**19**	1837.7884	919.3978	1820.7618	910.8846	1819.7778	910.3925	**N**	347.1674	174.0873	330.1408	165.5740			**3**
**20**	1894.8099	947.9086	1877.7833	939.3953	1876.7993	938.9033	**G**	232.1404	116.5738	215.1139	108.0606			**2**
**21**							**R**	175.1190	88.0631	158.0924	79.5498			**1**

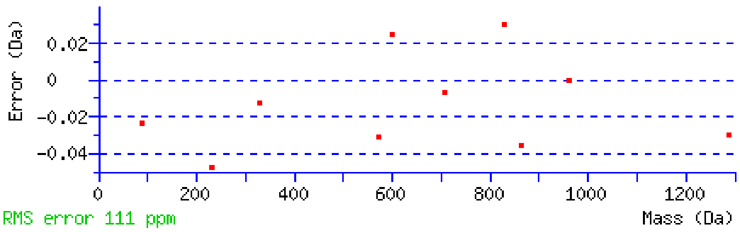

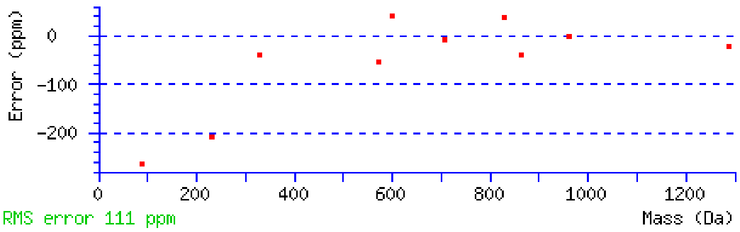

NCBI **BLAST** search of FMNVPGGGAAAVMMTGYNNGR
(Parameters: blastp, nr protein database, expect=20000, no filter, PAM30)
Other BLAST web gateways

All matches to this query

Score	Mr(calc)	Delta	Sequence
22.1	2131.9125	0.9933	FMNVPGGGAAAVMMTGYNNGR
22.1	2131.9125	0.9933	FMNVPGGGAAAVMMTGYNNGR
8.9	2131.9125	0.9933	YMNVPGGGAAAVMMTGYNNGR
8.9	2131.9125	0.9933	YMNVPGGGAAAVMMTGYNNGR
8.5	2130.8955	2.0103	MMNVPGGGAAAVMMTGYNNGR
8.5	2130.8955	2.0103	MMNVPGGGAAAVMMTGYNNGR
8.5	2130.8955	2.0103	MMNVPGGGAAAVMMTGYNNGR
8.5	2130.8955	2.0103	MMNVPGGGAAAVMMTGYNNGR

Mascot: http://www.matrixscience.com/

Mascot Search Results

Peptide View

MS/MS Fragmentation of **GMNVPGGGAAAVMMTGYNNGR**
Found in **H0Y7S5** in **uni_human_nr**, H0Y7S5_HUMAN SH3 and multiple ankyrin repeat domains protein 2 (Fragment) OS=Homo sapiens GN=SHANK2 PE=1 SV=1

Match to Query 7121: 2027.874848 from(1014.944700,2+) intensity(69315.4688) rtinseconds(3073) scans(17901) index(13654)
Title: 130809_HPL_Human_Plaque_BR2_TR3_13_Spectrum034300_scans__17901_RTINSECONDS=3073
Data file E:\\RAW\\HumanPlaques\\mgf\\T\\1D\\T130809_HPL_Human_Plaque_BR2_TR3_13.mgf

Click mouse within plot area to zoom in by factor of two about that point
Or, Plot from 50 to 650 Da Full range
Label all possible matches ○ Label matches used for scoring ⊙
Show Y-axis ☐

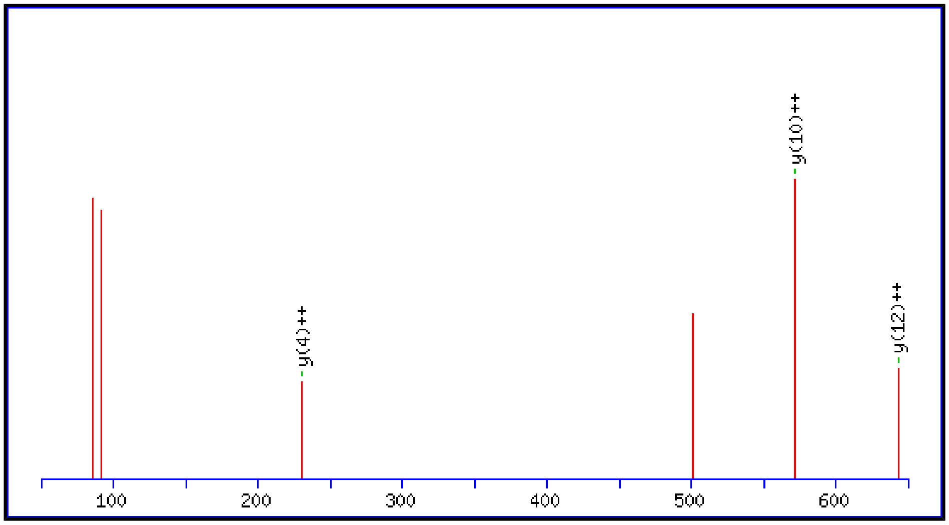


```
Monoisotopic mass of neutral peptide Mr(calc): 2025.8707
Fixed modifications: Carbamidomethyl (C) (apply to specified residues or termini only)
Variable modifications:
N3     : Deamidated (NQ)
N19    : Deamidated (NQ)
Ions Score: 18  Expect: 0.41
Matches : 3/196 fragment ions using 4 most intense peaks   (help)
```

#	b	b++	b*	b*++	b0	b0++	Seq.	y	y++	y*	y*++	y0	y0++	#
1	58.0287	29.5180					G							21
2	189.0692	95.0383					M	1969.8565	985.4319	1952.8299	976.9186	1951.8459	976.4266	20
3	304.0962	152.5517	287.0696	144.0384			N	1838.8160	919.9116	1821.7894	911.3984	1820.8054	910.9064	19
4	403.1646	202.0859	386.1380	193.5727			V	1723.7891	862.3982	1706.7625	853.8849	1705.7785	853.3929	18
5	500.2173	250.6123	483.1908	242.0990			P	1624.7206	812.8640	1607.6941	804.3507	1606.7101	803.8587	17
6	557.2388	279.1230	540.2123	270.6098			G	1527.6679	764.3376	1510.6413	755.8243	1509.6573	755.3323	16
7	614.2603	307.6338	597.2337	299.1205			G	1470.6464	735.8268	1453.6199	727.3136	1452.6358	726.8216	15
8	671.2817	336.1445	654.2552	327.6312			G	1413.6249	707.3161	1396.5984	698.8028	1395.6144	698.3108	14
9	742.3189	371.6631	725.2923	363.1498			A	1356.6035	678.8054	1339.5769	670.2921	1338.5929	669.8001	13
10	813.3560	407.1816	796.3294	398.6683			A	1285.5664	643.2868	1268.5398	634.7735	1267.5558	634.2815	12
11	884.3931	442.7002	867.3665	434.1869			A	1214.5293	607.7683	1197.5027	599.2550	1196.5187	598.7630	11
12	983.4615	492.2344	966.4349	483.7211			V	1143.4921	572.2497	1126.4656	563.7364	1125.4816	563.2444	10
13	1114.5020	557.7546	1097.4754	549.2414			M	1044.4237	522.7155	1027.3972	514.2022	1026.4132	513.7102	9
14	1245.5425	623.2749	1228.5159	614.7616			M	913.3832	457.1953	896.3567	448.6820	895.3727	448.1900	8

**15**	1346.5901	673.7987	1329.5636	665.2854	1328.5796	664.7934	**T**	782.3428	391.6750	765.3162	383.1617	764.3322	382.6697	**7**
**16**	1403.6116	702.3094	1386.5851	693.7962	1385.6010	693.3042	**G**	681.2951	341.1512	664.2685	332.6379			**6**
**17**	1566.6749	783.8411	1549.6484	775.3278	1548.6644	774.8358	**Y**	624.2736	312.6404	607.2471	304.1272			**5**
**18**	1680.7179	840.8626	1663.6913	832.3493	1662.7073	831.8573	**N**	461.2103	231.1088	444.1837	222.5955			**4**
**19**	1795.7448	898.3760	1778.7183	889.8628	1777.7342	889.3708	**N**	347.1674	174.0873	330.1408	165.5740			**3**
**20**	1852.7663	926.8868	1835.7397	918.3735	1834.7557	917.8815	**G**	232.1404	116.5738	215.1139	108.0606			**2**
**21**							**R**	175.1190	88.0631	158.0924	79.5498			**1**

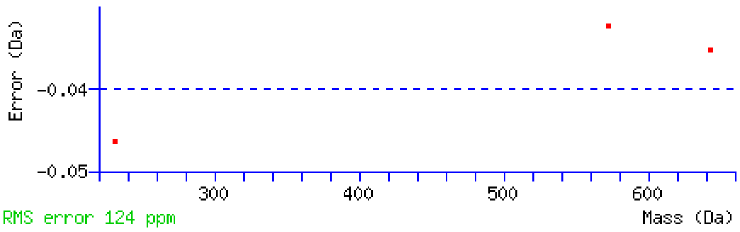

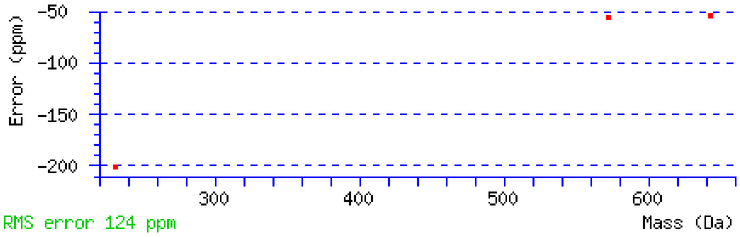

NCBI **BLAST** search of GMNVPGGGAAAVMMTGYNNGR
(Parameters: blastp, nr protein database, expect=20000, no filter, PAM30)
Other BLAST web gateways

All matches to this query

Score	Mr(calc)	Delta	Sequence
17.5	2025.8707	2.0042	GMNVPGGGAAAVMMTGYNNGR
17.5	2025.8707	2.0042	GMNVPGGGAAAVMMTGYNNGR
3.4	2025.8648	2.0100	HFSEAMTCPCGWRPFK

Mascot: http://www.matrixscience.com/

Mascot Search Results

Peptide View

MS/MS Fragmentation of **DIQMTQSPSSLSATVGDR**
Found in **P01613** in **uni_human_nr**, KV121_HUMAN Ig kappa chain V-I region Ni OS=Homo sapiens PE=1 SV=1

Match to Query 6546: 1892.876808 from(947.445680,2+) intensity(79460.0547) rtinseconds(2299) scans(12408) index(9080)
Title: 130806_HPL_Human_Plaque_BR2_TR2_16_Spectrum028722_scans__12408_RTINSECONDS=2299
Data file E:\\RAW\\HumanPlaques\\mgf\\T\\1D\\T130806_HPL_Human_Plaque_BR2_TR2_16.mgf

Click mouse within plot area to zoom in by factor of two about that point
Or, Plot from 200 to 360 Da Full range
Label all possible matches ○ Label matches used for scoring ⊙
Show Y-axis ☐

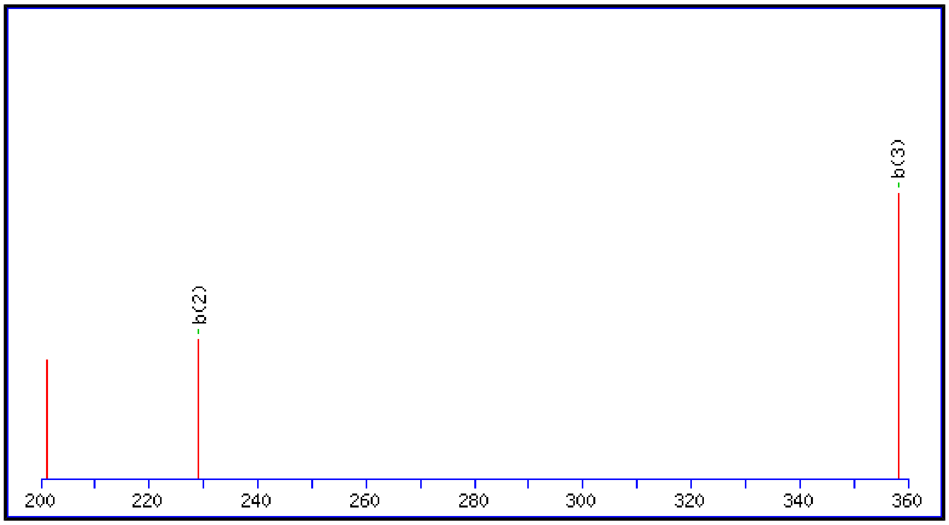

Monoisotopic mass of neutral peptide Mr(calc): 1892.8786
Fixed modifications: Carbamidomethyl (C) (apply to specified residues or termini only)
Variable modifications:
Q3 : Deamidated (NQ)
Ions Score: 16 **Expect:** 1.6
Matches : 2/198 fragment ions using 2 most intense peaks (help)

#	b	b^{++}	b*	b^{*++}	b^0	b^{0++}	Seq.	y	y^{++}	y*	y^{*++}	y^0	y^{0++}	#
1	116.0342	58.5207			98.0237	49.5155	D							18
2	***229.1183***	115.0628			211.1077	106.0575	I	1778.8589	889.9331	1761.8324	881.4198	1760.8483	880.9278	17
3	***358.1609***	179.5841	341.1343	171.0708	340.1503	170.5788	Q	1665.7748	833.3911	1648.7483	824.8778	1647.7643	824.3858	16
4	489.2014	245.1043	472.1748	236.5910	471.1908	236.0990	M	1536.7322	768.8698	1519.7057	760.3565	1518.7217	759.8645	15
5	590.2490	295.6282	573.2225	287.1149	572.2385	286.6229	T	1405.6918	703.3495	1388.6652	694.8362	1387.6812	694.3442	14
6	718.3076	359.6574	701.2811	351.1442	700.2971	350.6522	Q	1304.6441	652.8257	1287.6175	644.3124	1286.6335	643.8204	13
7	805.3396	403.1735	788.3131	394.6602	787.3291	394.1682	S	1176.5855	588.7964	1159.5590	580.2831	1158.5749	579.7911	12
8	902.3924	451.6998	885.3659	443.1866	884.3818	442.6946	P	1089.5535	545.2804	1072.5269	536.7671	1071.5429	536.2751	11
9	989.4244	495.2159	972.3979	486.7026	971.4139	486.2106	S	992.5007	496.7540	975.4742	488.2407	974.4901	487.7487	10
10	1076.4565	538.7319	1059.4299	530.2186	1058.4459	529.7266	S	905.4687	453.2380	888.4421	444.7247	887.4581	444.2327	9
11	1189.5405	595.2739	1172.5140	586.7606	1171.5300	586.2686	L	818.4367	409.7220	801.4101	401.2087	800.4261	400.7167	8
12	1276.5726	638.7899	1259.5460	630.2766	1258.5620	629.7846	S	705.3526	353.1799	688.3260	344.6667	687.3420	344.1747	7
13	1347.6097	674.3085	1330.5831	665.7952	1329.5991	665.3032	A	618.3206	309.6639	601.2940	301.1506	600.3100	300.6586	6
14	1448.6574	724.8323	1431.6308	716.3190	1430.6468	715.8270	T	547.2835	274.1454	530.2569	265.6321	529.2729	265.1401	5
15	1547.7258	774.3665	1530.6992	765.8532	1529.7152	765.3612	V	446.2358	223.6215	429.2092	215.1083	428.2252	214.6162	4

16	1604.7472	802.8773	1587.7207	794.3640	1586.7367	793.8720	G	347.1674	174.0873	330.1408	165.5740	329.1568	165.0820	3
17	1719.7742	860.3907	1702.7476	851.8775	1701.7636	851.3854	D	290.1459	145.5766	273.1193	137.0633	272.1353	136.5713	2
18							R	175.1190	88.0631	158.0924	79.5498			1

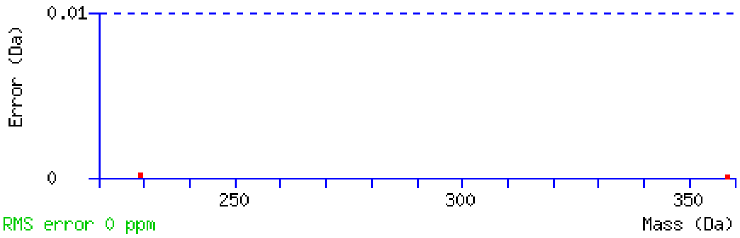

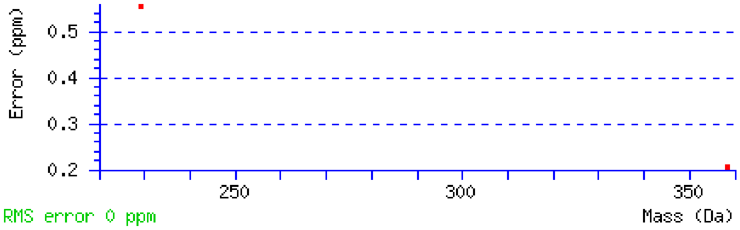

NCBI **BLAST** search of DIQMTQSPSSLSATVGDR
(Parameters: blastp, nr protein database, expect=20000, no filter, PAM30)
Other BLAST web gateways

All matches to this query

Score	Mr(calc)	Delta	Sequence
15.8	1892.8786	-0.0018	DIQMTQSPSSLSATVGDR
15.8	1892.8786	-0.0018	DIQMTQSPSTLSASVGDR
15.8	1890.8642	2.0126	DLQDRLSQMNGRWDR
15.8	1890.8642	2.0126	DLQDRLSQMNGRWDR
15.8	1890.8629	2.0139	IDELEEQCSEINREK
15.8	1891.8721	1.0048	NIEGATLTLQSECPETK
15.8	1891.8761	1.0008	NIQNFPSDLIENPIMK
15.8	1890.8694	2.0074	QVEEAEEEIDRLESSK

Mascot: http://www.matrixscience.com/

Mascot Search Results

Peptide View

MS/MS Fragmentation of **KQNLDLLEQLQVANGR**
Found in **Q86TI0** in **uni_human_nr**, TBCD1_HUMAN TBC1 domain family member 1 OS=Homo sapiens GN=TBC1D1 PE=1 SV=2

Match to Query 7266: 1843.938132 from(615.653320,3+) intensity(9126563.0000) rtinseconds(1139) scans(5220) index(3352)
Title: 130806_HPL_Human_Plaque_BR2_TR2_05_Spectrum025863_scans__5220_RTINSECONDS=1139
Data file E:\\RAW\\HumanPlaques\\mgf\\T\\1D\\T130806_HPL_Human_Plaque_BR2_TR2_05.mgf

Click mouse within plot area to zoom in by factor of two about that point
Or, Plot from 0 to 1900 Da Full range
Label all possible matches ○ Label matches used for scoring ⦿
Show Y-axis ☐

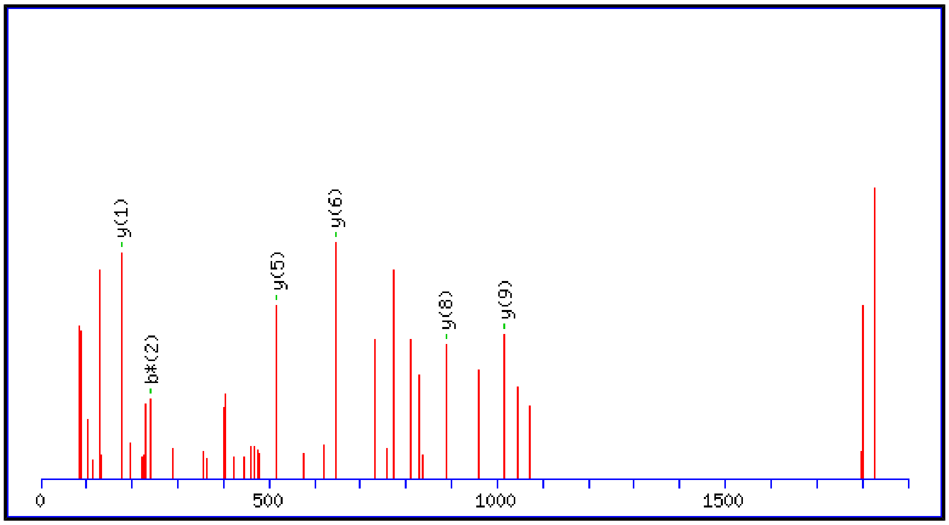

Monoisotopic mass of neutral peptide Mr(calc): 1841.9370
Fixed modifications: Carbamidomethyl (C) (apply to specified residues or termini only)
Variable modifications:
Q2 : Deamidated (NQ)
N3 : Deamidated (NQ)
Q11 : Deamidated (NQ)
N14 : Deamidated (NQ)
Ions Score: 22 **Expect:** 0.51
Matches : 6/156 fragment ions using 11 most intense peaks (help)

#	b	b^{++}	b*	b^{*++}	b^0	b^{0++}	Seq.	y	y^{++}	y*	y^{*++}	y^0	y^{0++}	#
1	129.1022	65.0548	112.0757	56.5415			K							16
2	258.1448	129.5761	241.1183	121.0628			Q	1714.8494	857.9283	1697.8228	849.4151	1696.8388	848.9230	15
3	373.1718	187.0895	356.1452	178.5763			N	1585.8068	793.4070	1568.7802	784.8938	1567.7962	784.4018	14
4	486.2558	243.6316	469.2293	235.1183			L	1470.7799	735.8936	1453.7533	727.3803	1452.7693	726.8883	13
5	601.2828	301.1450	584.2562	292.6318	583.2722	292.1397	D	1357.6958	679.3515	1340.6692	670.8383	1339.6852	670.3462	12
6	714.3668	357.6871	697.3403	349.1738	696.3563	348.6818	L	1242.6688	621.8381	1225.6423	613.3248	1224.6583	612.8328	11
7	827.4509	414.2291	810.4244	405.7158	809.4403	405.2238	L	1129.5848	565.2960	1112.5582	556.7828	1111.5742	556.2907	10
8	956.4935	478.7504	939.4670	470.2371	938.4829	469.7451	E	***1016.5007***	508.7540	999.4742	500.2407	998.4902	499.7487	9
9	1084.5521	542.7797	1067.5255	534.2664	1066.5415	533.7744	Q	***887.4581***	444.2327	870.4316	435.7194			8
10	1197.6361	599.3217	1180.6096	590.8084	1179.6256	590.3164	L	759.3995	380.2034	742.3730	371.6901			7
11	1326.6787	663.8430	1309.6522	655.3297	1308.6682	654.8377	Q	***646.3155***	323.6614	629.2889	315.1481			6
12	1425.7472	713.3772	1408.7206	704.8639	1407.7366	704.3719	V	***517.2729***	259.1401	500.2463	250.6268			5
13	1496.7843	748.8958	1479.7577	740.3825	1478.7737	739.8905	A	418.2045	209.6059	401.1779	201.0926			4
14	1611.8112	806.4092	1594.7847	797.8960	1593.8006	797.4040	N	347.1674	174.0873	330.1408	165.5740			3

**15**	1668.8327	834.9200	1651.8061	826.4067	1650.8221	825.9147	**G**	232.1404	116.5738	215.1139	108.0606			**2**
**16**							**R**	***175.1190***	88.0631	158.0924	79.5498			**1**

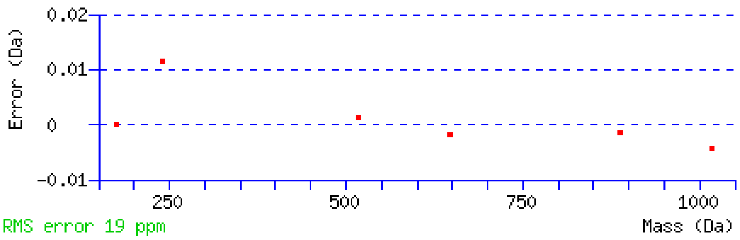

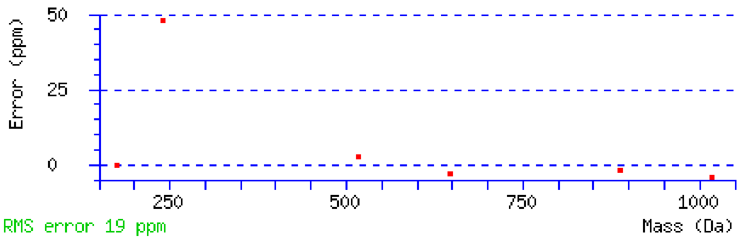

NCBI **BLAST** search of KQNLDLLEQLQVANGR
(Parameters: blastp, nr protein database, expect=20000, no filter, PAM30)
Other BLAST web gateways

All matches to this query

Score	Mr(calc)	Delta	Sequence
22.0	1841.9370	2.0011	KQNLDLLEQLQVANGR
11.7	1841.9370	2.0011	KQNLDLLEQLQVANGR
5.8	1842.9370	1.0012	MRLEQDLKNQALDNR
3.7	1843.9462	-0.0080	MIKETLPNVRVANDAR
2.8	1841.9370	2.0011	KQNLDLLEQLQVANGR
2.6	1843.9316	0.0066	QQFLEQSLQPGLQVAR
2.0	1841.9370	2.0011	KQNLDLLEQLQVANGR
2.0	1843.9428	-0.0047	ANISVAEAPSAFAVLGDGR
1.1	1841.9370	2.0011	KQNLDLLEQLQVANGR
1.0	1841.9384	1.9997	SLERDQVQLGLDWQR

Mascot: http://www.matrixscience.com/

Mascot Search Results

Peptide View

MS/MS Fragmentation of **LKVEGYSGTAGDSMAYHNGR**
Found in **P24821** in **uni_human_nr**, TENA_HUMAN Tenascin OS=Homo sapiens GN=TNC PE=1 SV=3

Match to Query 10463: 2127.961092 from(710.327640,3+) intensity(5838764.0000) rtinseconds(1108) scans(4724) index(2724)
Title: 130809_HPL_Human_Plaque_BR1_TR3_03_Spectrum026011_scans__4724_RTINSECONDS=1108
Data file E:\\RAW\\HumanPlaques\\mgf\\T\\1D\\T130809_HPL_Human_Plaque_BR1_TR3_03.mgf

Click mouse within plot area to zoom in by factor of two about that point
Or, [Plot from] [0] to [2200] Da [Full range]
Label all possible matches ○ Label matches used for scoring ⊙
Show Y-axis ☐

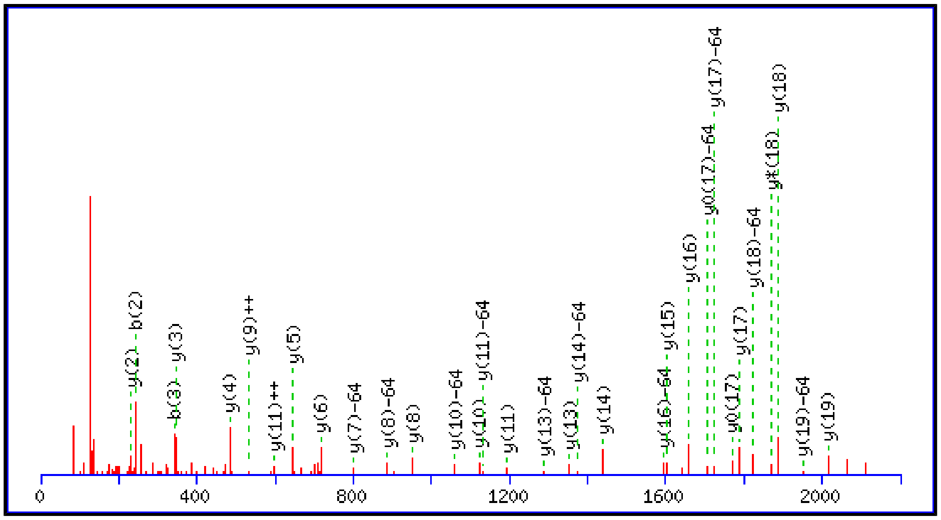

Monoisotopic mass of neutral peptide Mr(calc): 2127.9644
Fixed modifications: Carbamidomethyl (C) (apply to specified residues or termini only)
Variable modifications:
M14 : Oxidation (M), with neutral losses 0.0000(shown in table), 63.9983
Ions Score: 130 **Expect:** 5.5e-012
Matches : 32/318 fragment ions using 47 most intense peaks (help)

#	b	b^{++}	b*	b*$^{++}$	b^{0}	b^{0++}	Seq.	y	y^{++}	y*	y*$^{++}$	y^{0}	y^{0++}	#
1	114.0913	57.5493					L							20
2	**242.1863**	121.5968	225.1598	113.0835			K	***2015.8876***	1008.4474	1998.8610	999.9342	1997.8770	999.4421	19
3	**341.2547**	171.1310	324.2282	162.6177			V	***1887.7926***	944.3999	1870.7661	935.8867	1869.7821	935.3947	18
4	470.2973	235.6523	453.2708	227.1390	452.2867	226.6470	E	***1788.7242***	894.8657	1771.6977	886.3525	1770.7136	885.8605	17
5	527.3188	264.1630	510.2922	255.6498	509.3082	255.1577	G	***1659.6816***	830.3444	1642.6551	821.8312	1641.6710	821.3392	16
6	690.3821	345.6947	673.3556	337.1814	672.3715	336.6894	Y	***1602.6601***	801.8337	1585.6336	793.3204	1584.6496	792.8284	15
7	777.4141	389.2107	760.3876	380.6974	759.4036	380.2054	S	***1439.5968***	720.3020	1422.5703	711.7888	1421.5863	711.2968	14
8	834.4356	417.7214	817.4090	409.2082	816.4250	408.7162	G	***1352.5648***	676.7860	1335.5382	668.2728	1334.5542	667.7808	13
9	935.4833	468.2453	918.4567	459.7320	917.4727	459.2400	T	1295.5433	648.2753	1278.5168	639.7620	1277.5328	639.2700	12
10	1006.5204	503.7638	989.4938	495.2506	988.5098	494.7585	A	***1194.4956***	597.7515	1177.4691	589.2382	1176.4851	588.7462	11
11	1063.5419	532.2746	1046.5153	523.7613	1045.5313	523.2693	G	***1123.4585***	562.2329	1106.4320	553.7196	1105.4480	553.2276	10
12	1178.5688	589.7880	1161.5422	581.2748	1160.5582	580.7828	D	1066.4371	533.7222	1049.4105	525.2089	1048.4265	524.7169	9
13	1265.6008	633.3040	1248.5743	624.7908	1247.5903	624.2988	S	***951.4101***	476.2087	934.3836	467.6954	933.3996	467.2034	8
14	1412.6362	706.8217	1395.6097	698.3085	1394.6257	697.8165	M	864.3781	432.6927	847.3515	424.1794			7
15	1483.6733	742.3403	1466.6468	733.8270	1465.6628	733.3350	A	***717.3427***	359.1750	700.3161	350.6617			6
16	1646.7367	823.8720	1629.7101	815.3587	1628.7261	814.8667	Y	***646.3056***	323.6564	629.2790	315.1432			5

**17**	1783.7956	892.4014	1766.7690	883.8882	1765.7850	883.3961	**H**	***483.2423***	242.1248	466.2157	233.6115			**4**
**18**	1897.8385	949.4229	1880.8120	940.9096	1879.8279	940.4176	**N**	***346.1833***	173.5953	329.1568	165.0820			**3**
**19**	1954.8600	977.9336	1937.8334	969.4203	1936.8494	968.9283	**G**	***232.1404***	116.5738	215.1139	108.0606			**2**
**20**							**R**	175.1190	88.0631	158.0924	79.5498			**1**

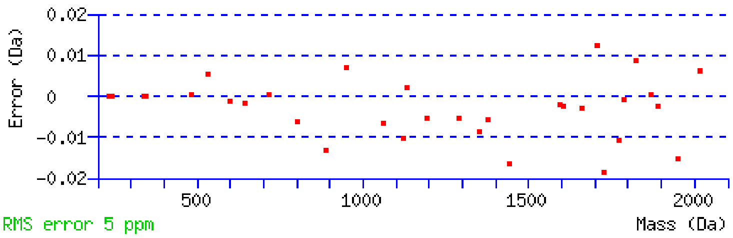

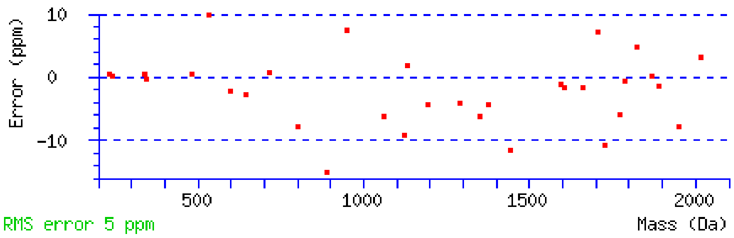

NCBI **BLAST** search of LKVEGYSGTAGDSMAYHNGR
(Parameters: blastp, nr protein database, expect=20000, no filter, PAM30)
Other BLAST web gateways

All matches to this query

Score	Mr(calc)	Delta	Sequence
129.8	2127.9644	-0.0033	LKVEGYSGTAGDSMAYHNGR

Mascot: http://www.matrixscience.com/

Mascot Search Results

Peptide View

MS/MS Fragmentation of **LKVEGYSGTAGDSMAYHNGR**
Found in **P24821** in **uni_human_nr**, TENA_HUMAN Tenascin OS=Homo sapiens GN=TNC PE=1 SV=3

Match to Query 11122: 2127.963336 from(532.998110,4+) intensity(8622274.0000) rtinseconds(1101) scans(4214) index(2107)
Title: 130801_HPL_Human_Plaque_BR2_TR1_02_Spectrum026262_scans__4214_RTINSECONDS=1101
Data file E:\\RAW\\HumanPlaques\\mgf\\T\\1D\\T130801_HPL_Human_Plaque_BR2_TR1_02.mgf

Click mouse within plot area to zoom in by factor of two about that point
Or, Plot from 0 to 2200 Da Full range
Label all possible matches ○ Label matches used for scoring ⊙
Show Y-axis ☐

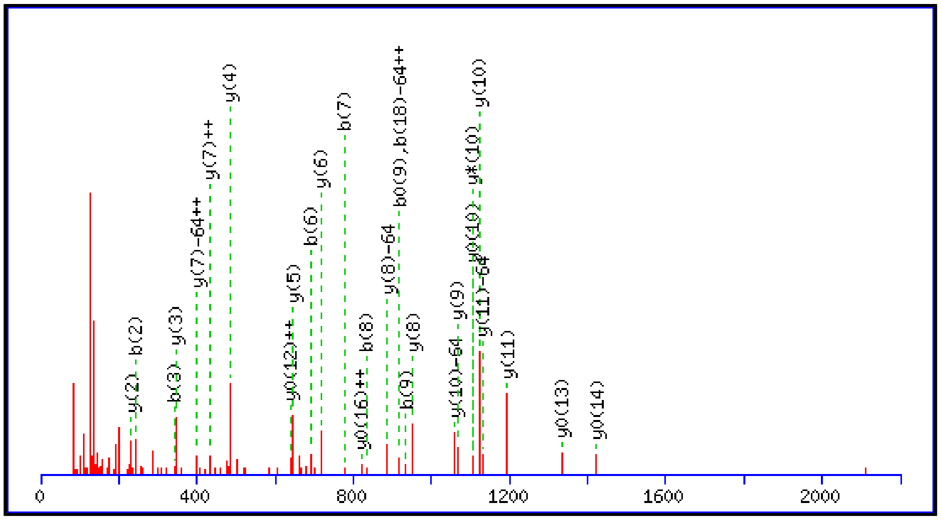

Monoisotopic mass of neutral peptide Mr(calc): 2127.9644
Fixed modifications: Carbamidomethyl (C) (apply to specified residues or termini only)
Variable modifications:
M14 : Oxidation (M), with neutral losses 0.0000(shown in table), 63.9983
Ions Score: 65 **Expect:** 1.6e-005
Matches : 28/318 fragment ions using 44 most intense peaks (help)

#	b	b^{++}	b*	b^{*++}	b^0	b^{0++}	Seq.	y	y^{++}	y*	y^{*++}	y^0	y^{0++}	#
1	114.0913	57.5493					L							20
2	***242.1863***	121.5968	225.1598	113.0835			K	2015.8876	1008.4474	1998.8610	999.9342	1997.8770	999.4421	19
3	***341.2547***	171.1310	324.2282	162.6177			V	1887.7926	944.3999	1870.7661	935.8867	1869.7821	935.3947	18
4	470.2973	235.6523	453.2708	227.1390	452.2867	226.6470	E	1788.7242	894.8657	1771.6977	886.3525	1770.7136	885.8605	17
5	527.3188	264.1630	510.2922	255.6498	509.3082	255.1577	G	1659.6816	830.3444	1642.6551	821.8312	1641.6710	821.3392	16
6	***690.3821***	345.6947	673.3556	337.1814	672.3715	336.6894	Y	1602.6601	801.8337	1585.6336	793.3204	1584.6496	792.8284	15
7	***777.4141***	389.2107	760.3876	380.6974	759.4036	380.2054	S	1439.5968	720.3020	1422.5703	711.7888	1421.5863	711.2968	14
8	***834.4356***	417.7214	817.4090	409.2082	816.4250	408.7162	G	1352.5648	676.7860	1335.5382	668.2728	1334.5542	667.7808	13
9	***935.4833***	468.2453	918.4567	459.7320	917.4727	459.2400	T	1295.5433	648.2753	1278.5168	639.7620	1277.5328	639.2700	12
10	1006.5204	503.7638	989.4938	495.2506	988.5098	494.7585	A	***1194.4956***	597.7515	1177.4691	589.2382	1176.4851	588.7462	11
11	1063.5419	532.2746	1046.5153	523.7613	1045.5313	523.2693	G	***1123.4585***	562.2329	1106.4320	553.7196	1105.4480	553.2276	10
12	1178.5688	589.7880	1161.5422	581.2748	1160.5582	580.7828	D	***1066.4371***	533.7222	1049.4105	525.2089	1048.4265	524.7169	9
13	1265.6008	633.3040	1248.5743	624.7908	1247.5903	624.2988	S	***951.4101***	476.2087	934.3836	467.6954	933.3996	467.2034	8
14	1412.6362	706.8217	1395.6097	698.3085	1394.6257	697.8165	M	864.3781	432.6927	847.3515	424.1794			7
15	1483.6733	742.3403	1466.6468	733.8270	1465.6628	733.3350	A	***717.3427***	359.1750	700.3161	350.6617			6
16	1646.7367	823.8720	1629.7101	815.3587	1628.7261	814.8667	Y	***646.3056***	323.6564	629.2790	315.1432			5

**17**	1783.7956	892.4014	1766.7690	883.8882	1765.7850	883.3961	**H**	***483.2423***	242.1248	466.2157	233.6115			**4**
**18**	1897.8385	949.4229	1880.8120	940.9096	1879.8279	940.4176	**N**	***346.1833***	173.5953	329.1568	165.0820			**3**
**19**	1954.8600	977.9336	1937.8334	969.4203	1936.8494	968.9283	**G**	***232.1404***	116.5738	215.1139	108.0606			**2**
**20**							**R**	175.1190	88.0631	158.0924	79.5498			**1**

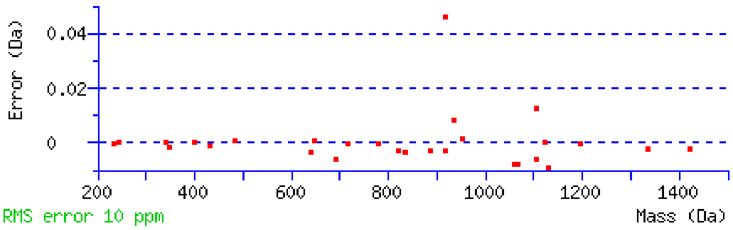

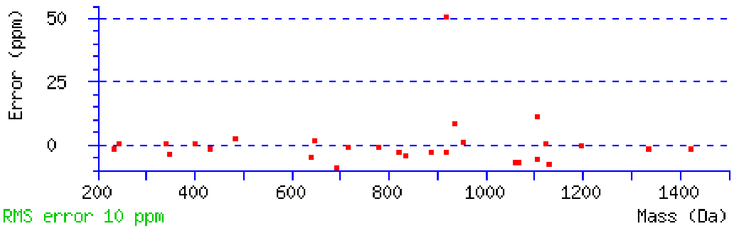

NCBI **BLAST** search of LKVEGYSGTAGDSMAYHNGR
(Parameters: blastp, nr protein database, expect=20000, no filter, PAM30)
Other BLAST web gateways

All matches to this query

Score	Mr(calc)	Delta	Sequence
65.3	2127.9644	-0.0010	LKVEGYSGTAGDSMAYHNGR

Mascot: http://www.matrixscience.com/

Mascot Search Results

Peptide View

MS/MS Fragmentation of **LKVEGYSGTAGDSMAYHNGR**
Found in **P24821** in **uni_human_nr**, TENA_HUMAN Tenascin OS=Homo sapiens GN=TNC PE=1 SV=3

Match to Query 9463: 2112.949722 from(705.323850,3+) intensity(495964.6875) rtinseconds(1473) scans(7773) index(5576)
Title: 130806_HPL_Human_Plaque_BR1_TR2_05_Spectrum028372_scans__7773_RTINSECONDS=1473
Data file E:\\RAW\\HumanPlaques\\mgf\\T\\1D\\T130806_HPL_Human_Plaque_BR1_TR2_05.mgf

Click mouse within plot area to zoom in by factor of two about that point
Or, Plot from 0 to 1900 Da Full range
Label all possible matches ○ Label matches used for scoring ⦿
Show Y-axis ☐

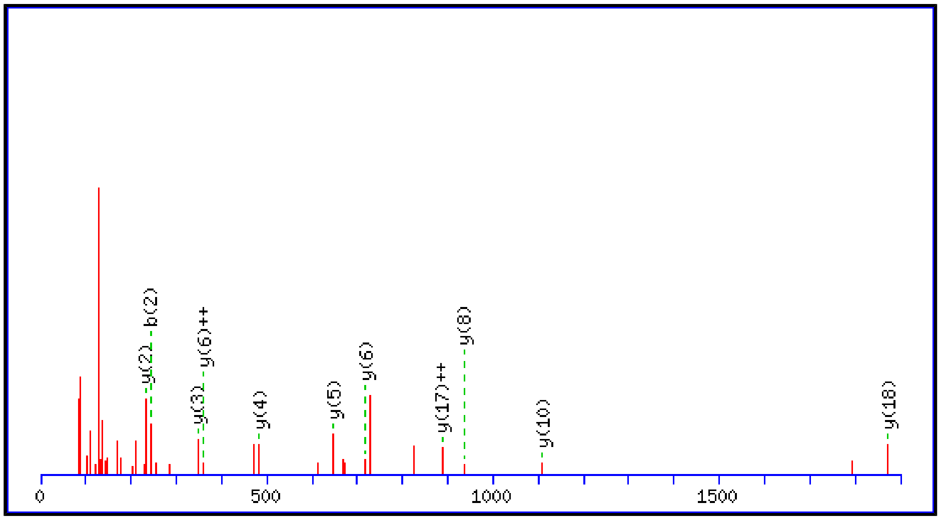

Monoisotopic mass of neutral peptide Mr(calc): 2112.9534
Fixed modifications: Carbamidomethyl (C) (apply to specified residues or termini only)
Variable modifications:
N18 : Deamidated (NQ)
Ions Score: 39 **Expect:** 0.0079
Matches : 11/206 fragment ions using 23 most intense peaks (help)

#	b	b^{++}	b*	b^{*++}	b^{0}	b^{0++}	Seq.	y	y^{++}	y*	y^{*++}	y^{0}	y^{0++}	#
1	114.0913	57.5493					L							20
2	**242.1863**	121.5968	225.1598	113.0835			K	2000.8767	1000.9420	1983.8501	992.4287	1982.8661	991.9367	19
3	341.2547	171.1310	324.2282	162.6177			V	***1872.7817***	936.8945	1855.7552	928.3812	1854.7712	927.8892	18
4	470.2973	235.6523	453.2708	227.1390	452.2867	226.6470	E	1773.7133	887.3603	1756.6868	878.8470	1755.7027	878.3550	17
5	527.3188	264.1630	510.2922	255.6498	509.3082	255.1577	G	1644.6707	822.8390	1627.6442	814.3257	1626.6601	813.8337	16
6	690.3821	345.6947	673.3556	337.1814	672.3715	336.6894	Y	1587.6492	794.3283	1570.6227	785.8150	1569.6387	785.3230	15
7	777.4141	389.2107	760.3876	380.6974	759.4036	380.2054	S	1424.5859	712.7966	1407.5594	704.2833	1406.5754	703.7913	14
8	834.4356	417.7214	817.4090	409.2082	816.4250	408.7162	G	1337.5539	669.2806	1320.5273	660.7673	1319.5433	660.2753	13
9	935.4833	468.2453	918.4567	459.7320	917.4727	459.2400	T	1280.5324	640.7699	1263.5059	632.2566	1262.5219	631.7646	12
10	1006.5204	503.7638	989.4938	495.2506	988.5098	494.7585	A	1179.4847	590.2460	1162.4582	581.7327	1161.4742	581.2407	11
11	1063.5419	532.2746	1046.5153	523.7613	1045.5313	523.2693	G	***1108.4476***	554.7275	1091.4211	546.2142	1090.4371	545.7222	10
12	1178.5688	589.7880	1161.5422	581.2748	1160.5582	580.7828	D	1051.4262	526.2167	1034.3996	517.7034	1033.4156	517.2114	9
13	1265.6008	633.3040	1248.5743	624.7908	1247.5903	624.2988	S	***936.3992***	468.7033	919.3727	460.1900	918.3887	459.6980	8
14	1396.6413	698.8243	1379.6148	690.3110	1378.6307	689.8190	M	849.3672	425.1872	832.3407	416.6740			7
15	1467.6784	734.3428	1450.6519	725.8296	1449.6679	725.3376	A	***718.3267***	359.6670	701.3002	351.1537			6
16	1630.7418	815.8745	1613.7152	807.3612	1612.7312	806.8692	Y	***647.2896***	324.1484	630.2631	315.6352			5

**17**	1767.8007	884.4040	1750.7741	875.8907	1749.7901	875.3987	**H**	***484.2263***	242.6168	467.1997	234.1035			**4**
**18**	1882.8276	941.9174	1865.8011	933.4042	1864.8170	932.9122	**N**	***347.1674***	174.0873	330.1408	165.5740			**3**
**19**	1939.8491	970.4282	1922.8225	961.9149	1921.8385	961.4229	**G**	***232.1404***	116.5738	215.1139	108.0606			**2**
**20**							**R**	175.1190	88.0631	158.0924	79.5498			**1**

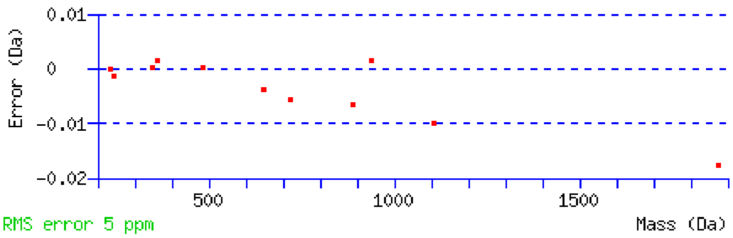

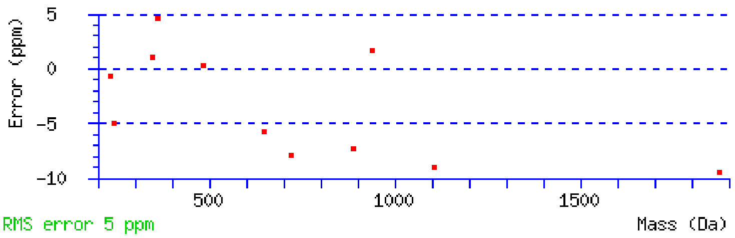

NCBI **BLAST** search of LKVEGYSGTAGDSMAYHNGR
(Parameters: blastp, nr protein database, expect=20000, no filter, PAM30)
Other BLAST web gateways

All matches to this query

Score	Mr(calc)	Delta	Sequence
38.5	2112.9534	-0.0037	LKVEGYSGTAGDSMAYHNGR
0.5	2111.9550	0.9947	KMNVPGGGAAAVMMTGYNNGR
0.5	2112.9391	0.0107	KMNVPGGGAAAVMMTGYNNGR
0.5	2111.9550	0.9947	KMNVPGGGAAAVMMTGYNNGR
0.5	2112.9391	0.0107	KMNVPGGGAAAVMMTGYNNGR
0.5	2112.9391	0.0107	KMNVPGGGAAAVMMTGYNNGR
0.5	2112.9391	0.0107	KMNVPGGGAAAVMMTGYNNGR
0.5	2111.9550	0.9947	KMNVPGGGAAAVMMTGYNNGR
0.5	2112.9391	0.0107	KMNVPGGGAAAVMMTGYNNGR
0.5	2112.9391	0.0107	KMNVPGGGAAAVMMTGYNNGR

Mascot: http://www.matrixscience.com/

Mascot Search Results

Peptide View

MS/MS Fragmentation of **LKVEGYSGTAGDSMAYHNGR**
Found in **P24821** in **uni_human_nr**, TENA_HUMAN Tenascin OS=Homo sapiens GN=TNC PE=1 SV=3

Match to Query 11125: 2111.967192 from(704.996340,3+) intensity(44985036.0000) rtinseconds(1239) scans(5782) index(3614)
Title: 130801_HPL_Human_Plaque_BR1_TR1_02_Spectrum027621_scans__5782_RTINSECONDS=1239
Data file E:\\RAW\\HumanPlaques\\mgf\\T\\1D\\T130801_HPL_Human_Plaque_BR1_TR1_02.mgf

Click mouse within plot area to zoom in by factor of two about that point
Or, Plot from 0 to 2200 Da Full range
Label all possible matches ○ Label matches used for scoring ⊙
Show Y-axis ☐

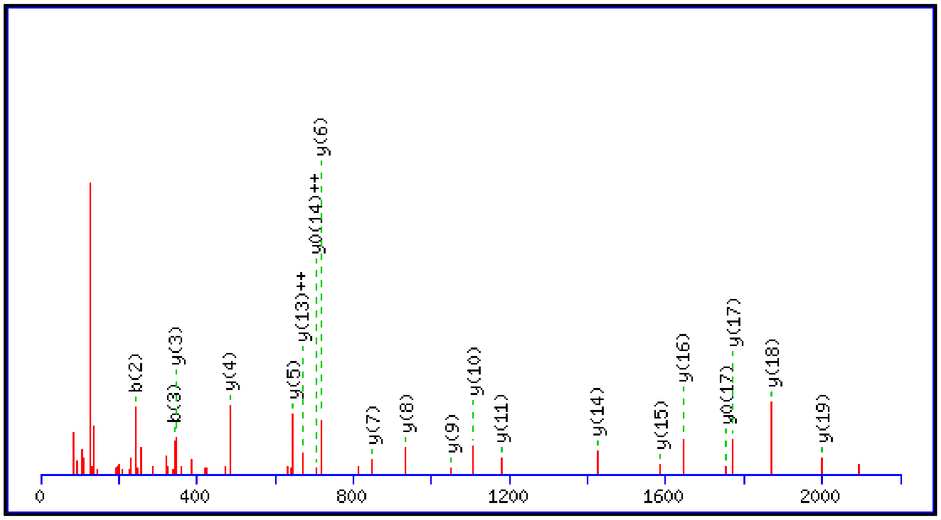

Monoisotopic mass of neutral peptide Mr(calc): 2111.9694
Fixed modifications: Carbamidomethyl (C) (apply to specified residues or termini only)
Ions Score: 145 **Expect:** 2.2e-013
Matches : 20/206 fragment ions using 27 most intense peaks (help)

#	b	b^{++}	b*	b^{*++}	b^0	b^{0++}	Seq.	y	y^{++}	y*	y^{*++}	y^0	y^{0++}	#
1	114.0913	57.5493					L							20
2	**242.1863**	121.5968	225.1598	113.0835			K	***1999.8927***	1000.4500	1982.8661	991.9367	1981.8821	991.4447	19
3	**341.2547**	171.1310	324.2282	162.6177			V	***1871.7977***	936.4025	1854.7712	927.8892	1853.7871	927.3972	18
4	470.2973	235.6523	453.2708	227.1390	452.2867	226.6470	E	***1772.7293***	886.8683	1755.7027	878.3550	1754.7187	877.8630	17
5	527.3188	264.1630	510.2922	255.6498	509.3082	255.1577	G	***1643.6867***	822.3470	1626.6601	813.8337	1625.6761	813.3417	16
6	690.3821	345.6947	673.3556	337.1814	672.3715	336.6894	Y	***1586.6652***	793.8363	1569.6387	785.3230	1568.6547	784.8310	15
7	777.4141	389.2107	760.3876	380.6974	759.4036	380.2054	S	***1423.6019***	712.3046	1406.5754	703.7913	1405.5913	703.2993	14
8	834.4356	417.7214	817.4090	409.2082	816.4250	408.7162	G	1336.5699	668.7886	1319.5433	660.2753	1318.5593	659.7833	13
9	935.4833	468.2453	918.4567	459.7320	917.4727	459.2400	T	1279.5484	640.2778	1262.5219	631.7646	1261.5378	631.2726	12
10	1006.5204	503.7638	989.4938	495.2506	988.5098	494.7585	A	***1178.5007***	589.7540	1161.4742	581.2407	1160.4902	580.7487	11
11	1063.5419	532.2746	1046.5153	523.7613	1045.5313	523.2693	G	***1107.4636***	554.2354	1090.4371	545.7222	1089.4531	545.2302	10
12	1178.5688	589.7880	1161.5422	581.2748	1160.5582	580.7828	D	***1050.4422***	525.7247	1033.4156	517.2114	1032.4316	516.7194	9
13	1265.6008	633.3040	1248.5743	624.7908	1247.5903	624.2988	S	***935.4152***	468.2112	918.3887	459.6980	917.4046	459.2060	8
14	1396.6413	698.8243	1379.6148	690.3110	1378.6307	689.8190	M	***848.3832***	424.6952	831.3566	416.1820			7
15	1467.6784	734.3428	1450.6519	725.8296	1449.6679	725.3376	A	***717.3427***	359.1750	700.3161	350.6617			6
16	1630.7418	815.8745	1613.7152	807.3612	1612.7312	806.8692	Y	***646.3056***	323.6564	629.2790	315.1432			5
17	1767.8007	884.4040	1750.7741	875.8907	1749.7901	875.3987	H	***483.2423***	242.1248	466.2157	233.6115			4

18	1881.8436	941.4254	1864.8170	932.9122	1863.8330	932.4202	N	*346.1833*	173.5953	329.1568	165.0820			3
19	1938.8651	969.9362	1921.8385	961.4229	1920.8545	960.9309	G	232.1404	116.5738	215.1139	108.0606			2
20							R	175.1190	88.0631	158.0924	79.5498			1

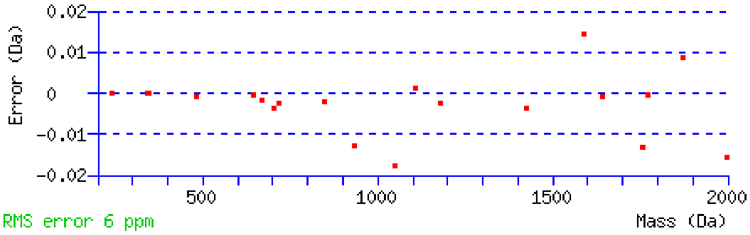

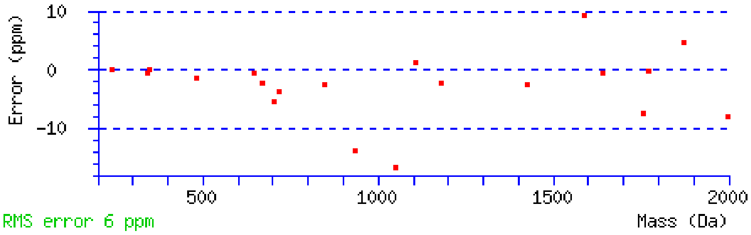

NCBI **BLAST** search of LKVEGYSGTAGDSMAYHNGR
(Parameters: blastp, nr protein database, expect=20000, no filter, PAM30)
Other BLAST web gateways

All matches to this query

Score	Mr(calc)	Delta	Sequence
144.7	2111.9694	-0.0022	LKVEGYSGTAGDSMAYHNGR
3.2	2109.9524	2.0148	KICEQSTESLNNDWEKK
2.3	2109.9524	2.0148	KICEQSTESLNNDWEKK

**Mascot:** http://www.matrixscience.com/

Mascot Search Results

Peptide View

MS/MS Fragmentation of **LKVEGYSGTAGDSMAYHNGR**
Found in **P24821** in **uni_human_nr**, TENA_HUMAN Tenascin OS=Homo sapiens GN=TNC PE=1 SV=3

Match to Query 10986: 2111.967128 from(1056.990840,2+) intensity(8721993.0000) rtinseconds(1238) scans(5386) index(3168)
Title: 130801_HPL_Human_Plaque_BR2_TR1_02_Spectrum027323_scans__5386_RTINSECONDS=1238
Data file E:\\RAW\\HumanPlaques\\mgf\\T\\1D\\T130801_HPL_Human_Plaque_BR2_TR1_02.mgf

Click mouse within plot area to zoom in by factor of two about that point
Or, Plot from 0 to 2200 Da Full range
Label all possible matches ○ Label matches used for scoring ⊙
Show Y-axis ☐

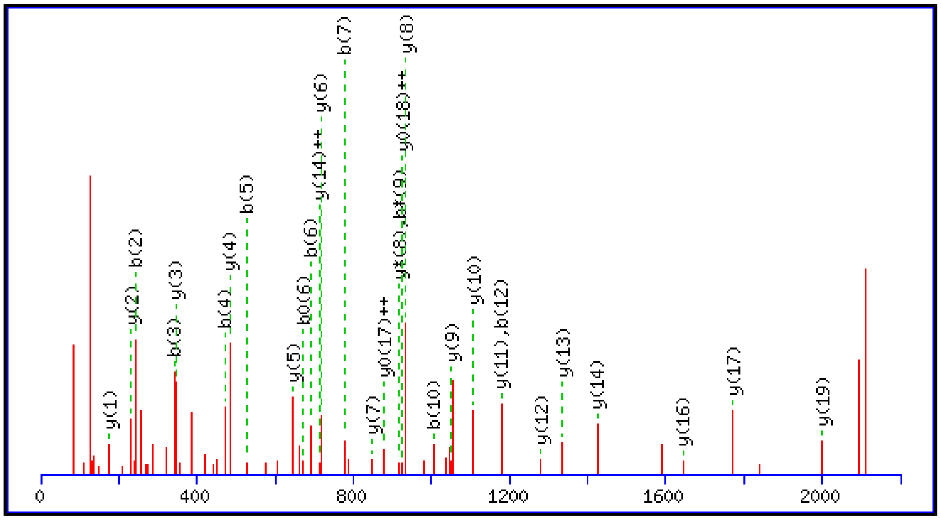

Monoisotopic mass of neutral peptide Mr(calc): 2111.9694
Fixed modifications: Carbamidomethyl (C) (apply to specified residues or termini only)
Ions Score: 132 **Expect:** 4.2e-012
Matches : 31/206 fragment ions using 55 most intense peaks (help)

#	b	b^{++}	b*	b^{*++}	b^{0}	b^{0++}	Seq.	y	y^{++}	y*	y^{*++}	y^{0}	y^{0++}	#
1	114.0913	57.5493					L							20
2	**242.1863**	121.5968	225.1598	113.0835			K	***1999.8927***	1000.4500	1982.8661	991.9367	1981.8821	991.4447	19
3	**341.2547**	171.1310	324.2282	162.6177			V	1871.7977	936.4025	1854.7712	927.8892	1853.7871	927.3972	18
4	**470.2973**	235.6523	453.2708	227.1390	452.2867	226.6470	E	***1772.7293***	886.8683	1755.7027	878.3550	1754.7187	877.8630	17
5	**527.3188**	264.1630	510.2922	255.6498	509.3082	255.1577	G	***1643.6867***	822.3470	1626.6601	813.8337	1625.6761	813.3417	16
6	**690.3821**	345.6947	673.3556	337.1814	672.3715	336.6894	Y	1586.6652	793.8363	1569.6387	785.3230	1568.6547	784.8310	15
7	**777.4141**	389.2107	760.3876	380.6974	759.4036	380.2054	S	***1423.6019***	712.3046	1406.5754	703.7913	1405.5913	703.2993	14
8	834.4356	417.7214	817.4090	409.2082	816.4250	408.7162	G	***1336.5699***	668.7886	1319.5433	660.2753	1318.5593	659.7833	13
9	935.4833	468.2453	918.4567	459.7320	917.4727	459.2400	T	***1279.5484***	640.2778	1262.5219	631.7646	1261.5378	631.2726	12
10	**1006.5204**	503.7638	989.4938	495.2506	988.5098	494.7585	A	***1178.5007***	589.7540	1161.4742	581.2407	1160.4902	580.7487	11
11	1063.5419	532.2746	1046.5153	523.7613	1045.5313	523.2693	G	***1107.4636***	554.2354	1090.4371	545.7222	1089.4531	545.2302	10
12	**1178.5688**	589.7880	1161.5422	581.2748	1160.5582	580.7828	D	***1050.4422***	525.7247	1033.4156	517.2114	1032.4316	516.7194	9
13	1265.6008	633.3040	1248.5743	624.7908	1247.5903	624.2988	S	***935.4152***	468.2112	918.3887	459.6980	917.4046	459.2060	8
14	1396.6413	698.8243	1379.6148	690.3110	1378.6307	689.8190	M	***848.3832***	424.6952	831.3566	416.1820			7
15	1467.6784	734.3428	1450.6519	725.8296	1449.6679	725.3376	A	***717.3427***	359.1750	700.3161	350.6617			6
16	1630.7418	815.8745	1613.7152	807.3612	1612.7312	806.8692	Y	***646.3056***	323.6564	629.2790	315.1432			5
17	1767.8007	884.4040	1750.7741	875.8907	1749.7901	875.3987	H	***483.2423***	242.1248	466.2157	233.6115			4

**18**	1881.8436	941.4254	1864.8170	932.9122	1863.8330	932.4202	**N**	*346.1833*	173.5953	329.1568	165.0820			**3**
**19**	1938.8651	969.9362	1921.8385	961.4229	1920.8545	960.9309	**G**	*232.1404*	116.5738	215.1139	108.0606			**2**
**20**							**R**	*175.1190*	88.0631	158.0924	79.5498			**1**

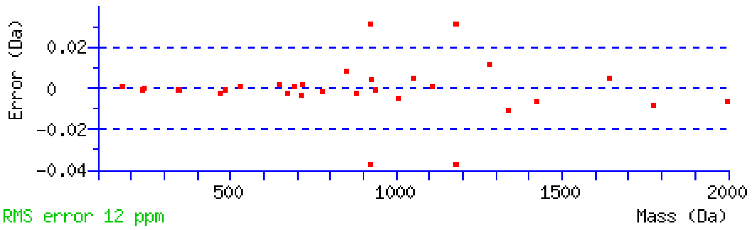

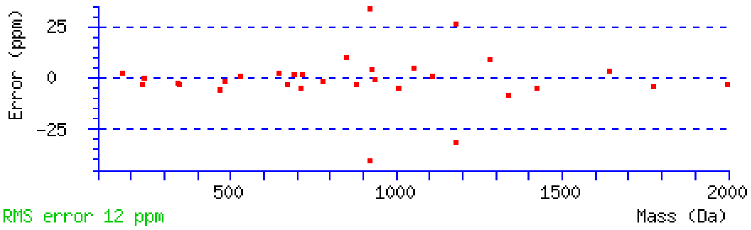

NCBI **BLAST** search of LKVEGYSGTAGDSMAYHNGR
(Parameters: blastp, nr protein database, expect=20000, no filter, PAM30)
Other BLAST web gateways

All matches to this query

Score	Mr(calc)	Delta	Sequence
131.8	2111.9694	-0.0023	LKVEGYSGTAGDSMAYHNGR

**Mascot:** http://www.matrixscience.com/

Mascot Search Results

Peptide View

MS/MS Fragmentation of **NGRENFYQNWK**
Found in **P24821** in **uni_human_nr**, TENA_HUMAN Tenascin OS=Homo sapiens GN=TNC PE=1 SV=3

Match to Query 3794: 1455.651828 from(728.833190,2+) intensity(1754434.0000) rtinseconds(1640) scans(9333) index(6946)
Title: 130809_HPL_Human_Plaque_BR2_TR3_03_Spectrum030734_scans__9333_RTINSECONDS=1640
Data file E:\\RAW\\HumanPlaques\\mgf\\T\\1D\\T130809_HPL_Human_Plaque_BR2_TR3_03.mgf

Click mouse within plot area to zoom in by factor of two about that point
Or, Plot from 0 to 1500 Da Full range
Label all possible matches ○ Label matches used for scoring ◉
Show Y-axis ☐

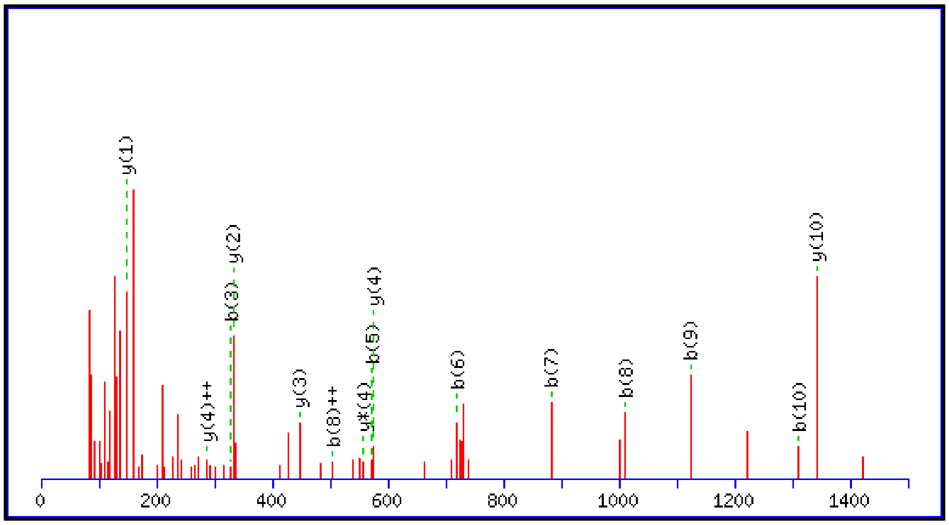

Monoisotopic mass of neutral peptide Mr(calc): 1455.6531
Fixed modifications: Carbamidomethyl (C) (apply to specified residues or termini only)
Variable modifications:
N1 : Deamidated (NQ)
Ions Score: 25 **Expect:** 0.11
Matches : 15/100 fragment ions using 43 most intense peaks (help)

#	b	b^{++}	b*	b^{*++}	b^{0}	b^{0++}	Seq.	y	y^{++}	y*	y^{*++}	y^{0}	y^{0++}	#
1	116.0342	58.5207	99.0077	50.0075			N							11
2	173.0557	87.0315	156.0291	78.5182			G	**1341.6335**	671.3204	1324.6069	662.8071	1323.6229	662.3151	10
3	***329.1568***	165.0820	312.1302	156.5688			R	1284.6120	642.8096	1267.5854	634.2964	1266.6014	633.8044	9
4	458.1994	229.6033	441.1728	221.0901	440.1888	220.5980	E	1128.5109	564.7591	1111.4843	556.2458	1110.5003	555.7538	8
5	***572.2423***	286.6248	555.2158	278.1115	554.2317	277.6195	N	999.4683	500.2378	982.4417	491.7245			7
6	***719.3107***	360.1590	702.2842	351.6457	701.3002	351.1537	F	885.4254	443.2163	868.3988	434.7030			6
7	***882.3741***	441.6907	865.3475	433.1774	864.3635	432.6854	Y	738.3570	369.6821	721.3304	361.1688			5
8	***1010.4326***	**505.7200**	993.4061	497.2067	992.4221	496.7147	Q	**575.2936**	288.1504	558.2671	279.6372			4
9	***1124.4756***	562.7414	1107.4490	554.2281	1106.4650	553.7361	N	**447.2350**	224.1212	430.2085	215.6079			3
10	***1310.5549***	655.7811	1293.5283	647.2678	1292.5443	646.7758	W	**333.1921**	167.0997	316.1656	158.5864			2
11							K	**147.1128**	74.0600	130.0863	65.5468			1

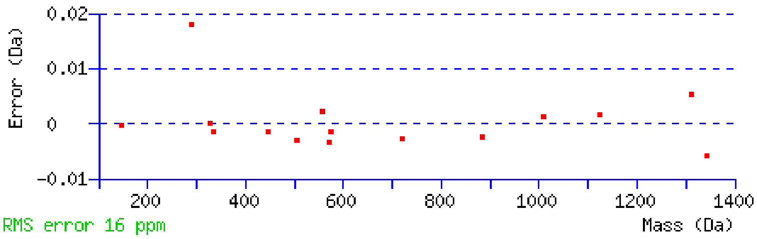

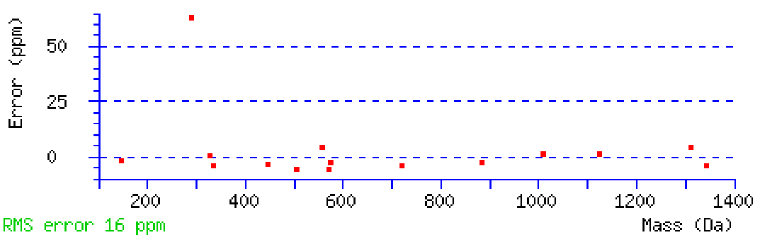

NCBI **BLAST** search of NGRENFYQNWK
(Parameters: blastp, nr protein database, expect=20000, no filter, PAM30)
Other BLAST web gateways

All matches to this query

Score	Mr(calc)	Delta	Sequence
25.2	1455.6531	-0.0013	NGRENFYQNWK
21.9	1455.6531	-0.0013	NGRENFYQNWK
0.6	1455.6531	-0.0013	NGRENFYQNWK

Mascot: http://www.matrixscience.com/

Mascot Search Results

Peptide View

MS/MS Fragmentation of **NGRENFYQNWK**
Found in **P24821** in **uni_human_nr**, TENA_HUMAN Tenascin OS=Homo sapiens GN=TNC PE=1 SV=3

Match to Query 4089: 1454.669922 from(485.897250,3+) intensity(11428533.0000) rtinseconds(1191) scans(5372) index(3246)
Title: 130801_HPL_Human_Plaque_BR1_TR1_02_Spectrum027253_scans__5372_RTINSECONDS=1191
Data file E:\\RAW\\HumanPlaques\\mgf\\T\\1D\\T130801_HPL_Human_Plaque_BR1_TR1_02.mgf

Click mouse within plot area to zoom in by factor of two about that point
Or, Plot from 0 to 1500 Da Full range
Label all possible matches ○ Label matches used for scoring ⦿
Show Y-axis ☐

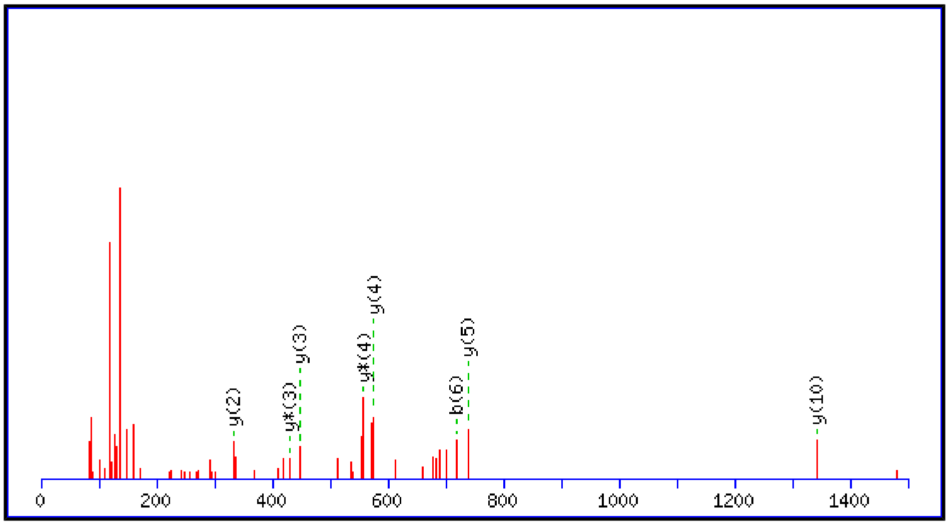

Monoisotopic mass of neutral peptide Mr(calc): 1454.6691
Fixed modifications: Carbamidomethyl (C) (apply to specified residues or termini only)
Ions Score: 23 **Expect:** 0.24
Matches : 8/100 fragment ions using 15 most intense peaks (help)

#	b	b^{++}	b*	b^{*++}	b^{0}	b^{0++}	Seq.	y	y^{++}	y*	y^{*++}	y^{0}	y^{0++}	#
1	115.0502	58.0287	98.0237	49.5155			N							11
2	172.0717	86.5395	155.0451	78.0262			G	***1341.6335***	671.3204	1324.6069	662.8071	1323.6229	662.3151	10
3	328.1728	164.5900	311.1462	156.0768			R	1284.6120	642.8096	1267.5854	634.2964	1266.6014	633.8044	9
4	457.2154	229.1113	440.1888	220.5980	439.2048	220.1060	E	1128.5109	564.7591	1111.4843	556.2458	1110.5003	555.7538	8
5	571.2583	286.1328	554.2317	277.6195	553.2477	277.1275	N	999.4683	500.2378	982.4417	491.7245			7
6	**718.3267**	359.6670	701.3002	351.1537	700.3161	350.6617	F	885.4254	443.2163	868.3988	434.7030			6
7	881.3900	441.1987	864.3635	432.6854	863.3795	432.1934	Y	***738.3570***	369.6821	721.3304	361.1688			5
8	1009.4486	505.2279	992.4221	496.7147	991.4381	496.2227	Q	***575.2936***	288.1504	558.2671	279.6372			4
9	1123.4915	562.2494	1106.4650	553.7361	1105.4810	553.2441	N	***447.2350***	224.1212	430.2085	215.6079			3
10	1309.5709	655.2891	1292.5443	646.7758	1291.5603	646.2838	W	***333.1921***	167.0997	316.1656	158.5864			2
11							K	147.1128	74.0600	130.0863	65.5468			1

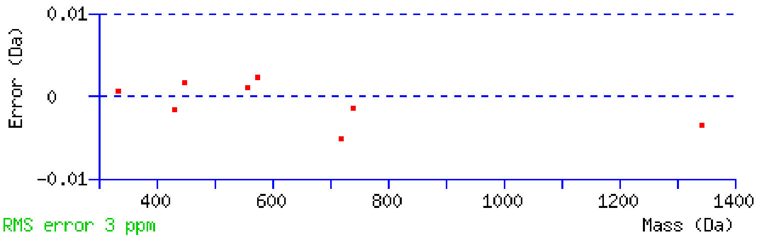

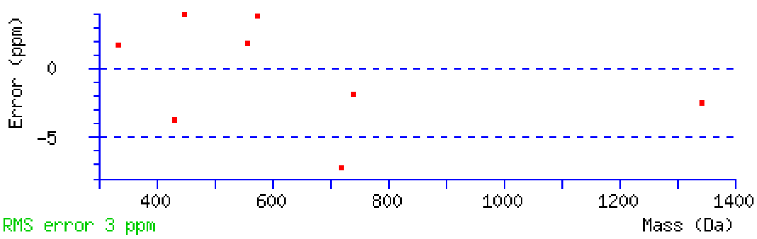

NCBI **BLAST** search of NGRENFYQNWK
(Parameters: blastp, nr protein database, expect=20000, no filter, PAM30)
Other BLAST web gateways

All matches to this query

Score	Mr(calc)	Delta	Sequence
23.1	1454.6691	0.0008	NGRENFYQNWK

Mascot: http://www.matrixscience.com/

Mascot Search Results

Peptide View

MS/MS Fragmentation of **NGRENFYQNWK**
Found in **P24821** in **uni_human_nr**, TENA_HUMAN Tenascin OS=Homo sapiens GN=TNC PE=1 SV=3

Match to Query 4283: 1454.670988 from(728.342770,2+) intensity(8415359.0000) rtinseconds(1223) scans(5851) index(3799)
Title: 130809_HPL_Human_Plaque_BR1_TR3_02_Spectrum028325_scans__5851_RTINSECONDS=1223
Data file E:\\RAW\\HumanPlaques\\mgf\\T\\1D\\T130809_HPL_Human_Plaque_BR1_TR3_02.mgf

Click mouse within plot area to zoom in by factor of two about that point
Or, Plot from 0 to 1500 Da Full range
Label all possible matches ○ Label matches used for scoring ⊙
Show Y-axis ☐

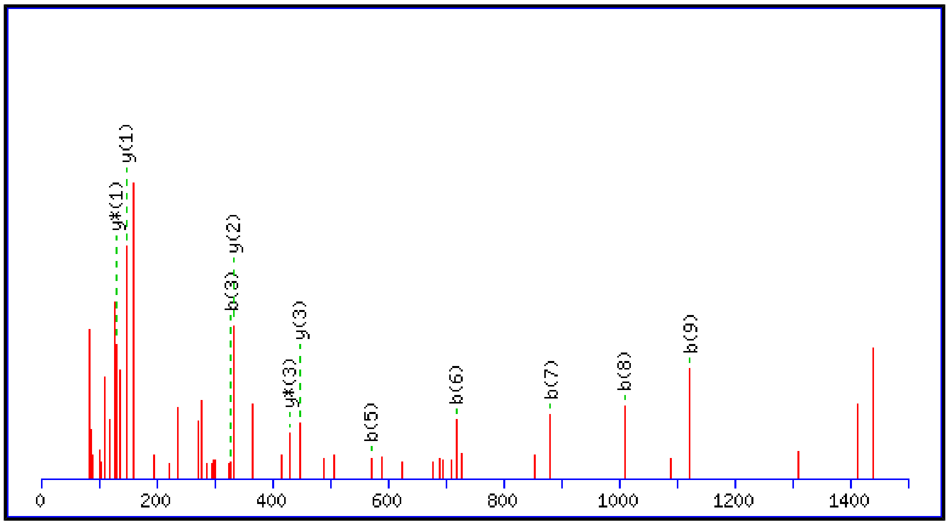

Monoisotopic mass of neutral peptide Mr(calc): 1454.6691
Fixed modifications: Carbamidomethyl (C) (apply to specified residues or termini only)
Ions Score: 19 **Expect:** 0.65
Matches : 11/100 fragment ions using 38 most intense peaks (help)

#	b	b^{++}	b*	b^{*++}	b^0	b^{0++}	Seq.	y	y^{++}	y*	y^{*++}	y^0	y^{0++}	#
1	115.0502	58.0287	98.0237	49.5155			N							11
2	172.0717	86.5395	155.0451	78.0262			G	1341.6335	671.3204	1324.6069	662.8071	1323.6229	662.3151	10
3	***328.1728***	164.5900	311.1462	156.0768			R	1284.6120	642.8096	1267.5854	634.2964	1266.6014	633.8044	9
4	457.2154	229.1113	440.1888	220.5980	439.2048	220.1060	E	1128.5109	564.7591	1111.4843	556.2458	1110.5003	555.7538	8
5	***571.2583***	286.1328	554.2317	277.6195	553.2477	277.1275	N	999.4683	500.2378	982.4417	491.7245			7
6	***718.3267***	359.6670	701.3002	351.1537	700.3161	350.6617	F	885.4254	443.2163	868.3988	434.7030			6
7	***881.3900***	441.1987	864.3635	432.6854	863.3795	432.1934	Y	738.3570	369.6821	721.3304	361.1688			5
8	***1009.4486***	505.2279	992.4221	496.7147	991.4381	496.2227	Q	575.2936	288.1504	558.2671	279.6372			4
9	***1123.4915***	562.2494	1106.4650	553.7361	1105.4810	553.2441	N	**447.2350**	224.1212	430.2085	215.6079			3
10	1309.5709	655.2891	1292.5443	646.7758	1291.5603	646.2838	W	**333.1921**	167.0997	316.1656	158.5864			2
11							K	**147.1128**	74.0600	130.0863	65.5468			1

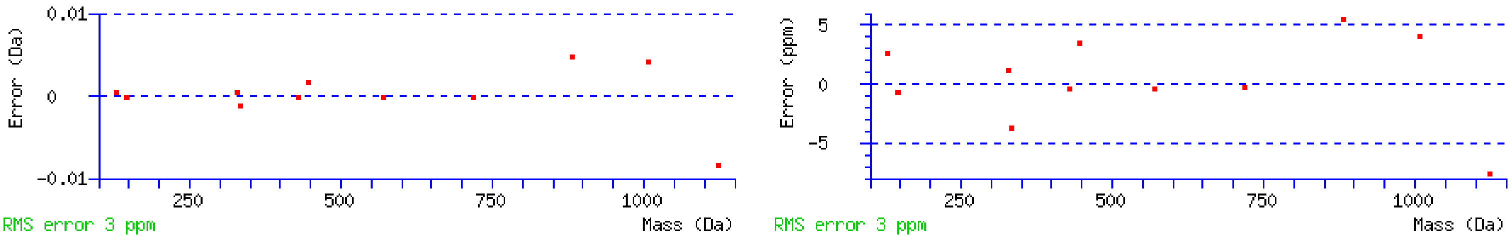

NCBI **BLAST** search of NGRENFYQNWK
(Parameters: blastp, nr protein database, expect=20000, no filter, PAM30)
Other BLAST web gateways

All matches to this query

Score	Mr(calc)	Delta	Sequence
18.9	1454.6691	0.0019	NGRENFYQNWK

Mascot: http://www.matrixscience.com/

Mascot Search Results

Peptide View

MS/MS Fragmentation of **VEGYSGTAGDSMAYHNGR**
Found in **P24821** in **uni_human_nr**, TENA_HUMAN Tenascin OS=Homo sapiens GN=TNC PE=1 SV=3

Match to Query 7542: 1886.786292 from(629.936040,3+) intensity(951566.1875) rtinseconds(969) scans(3843) index(2006)
Title: 130801_HPL_Human_Plaque_BR1_TR1_04_Spectrum025004_scans__3843_RTINSECONDS=969
Data file E:\\RAW\\HumanPlaques\\mgf\\T\\1D\\T130801_HPL_Human_Plaque_BR1_TR1_04.mgf

Click mouse within plot area to zoom in by factor of two about that point
Or, [Plot from] [0] to [1800] Da [Full range]
Label all possible matches ○ Label matches used for scoring ⊙
Show Y-axis ☐

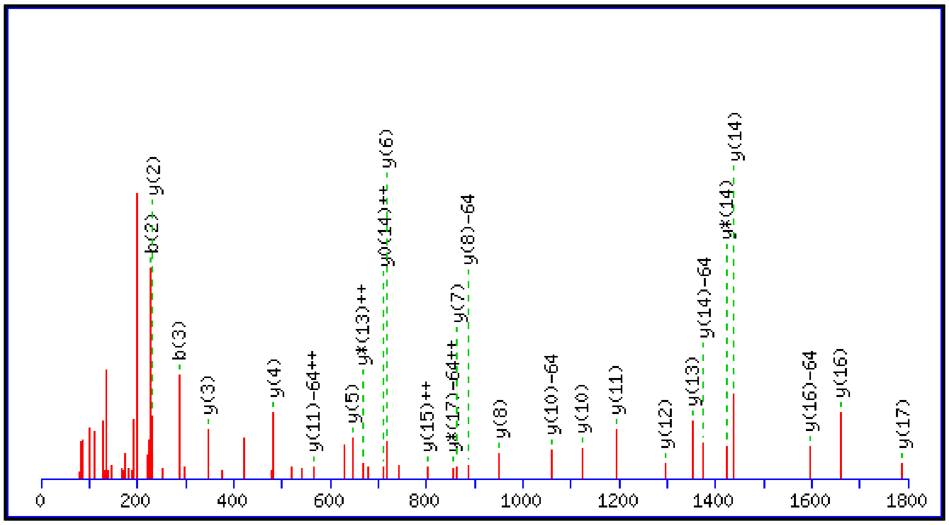

Monoisotopic mass of neutral peptide Mr(calc): 1886.7853
Fixed modifications: Carbamidomethyl (C) (apply to specified residues or termini only)
Variable modifications:
M12 : Oxidation (M), with neutral losses 0.0000(shown in table), 63.9983
Ions Score: 120 **Expect:** 1.2e-011
Matches : 26/250 fragment ions using 37 most intense peaks (help)

#	b	b^{++}	b*	b^{*++}	b^{0}	b^{0++}	Seq.	y	y^{++}	y*	y^{*++}	y^{0}	y^{0++}	#
1	100.0757	50.5415					V							18
2	229.1183	115.0628			211.1077	106.0575	E	1788.7242	894.8657	1771.6977	886.3525	1770.7136	885.8605	17
3	286.1397	143.5735			268.1292	134.5682	G	1659.6816	830.3444	1642.6551	821.8312	1641.6710	821.3392	16
4	449.2031	225.1052			431.1925	216.0999	Y	1602.6601	801.8337	1585.6336	793.3204	1584.6496	792.8284	15
5	536.2351	268.6212			518.2245	259.6159	S	1439.5968	720.3020	1422.5703	711.7888	1421.5863	711.2968	14
6	593.2566	297.1319			575.2460	288.1266	G	1352.5648	676.7860	1335.5382	668.2728	1334.5542	667.7808	13
7	694.3042	347.6558			676.2937	338.6505	T	1295.5433	648.2753	1278.5168	639.7620	1277.5328	639.2700	12
8	765.3414	383.1743			747.3308	374.1690	A	1194.4956	597.7515	1177.4691	589.2382	1176.4851	588.7462	11
9	822.3628	411.6851			804.3523	402.6798	G	1123.4585	562.2329	1106.4320	553.7196	1105.4480	553.2276	10
10	937.3898	469.1985			919.3792	460.1932	D	1066.4371	533.7222	1049.4105	525.2089	1048.4265	524.7169	9
11	1024.4218	512.7145			1006.4112	503.7093	S	951.4101	476.2087	934.3836	467.6954	933.3996	467.2034	8
12	1171.4572	586.2322			1153.4466	577.2270	M	864.3781	432.6927	847.3515	424.1794			7
13	1242.4943	621.7508			1224.4837	612.7455	A	717.3427	359.1750	700.3161	350.6617			6
14	1405.5576	703.2825			1387.5471	694.2772	Y	646.3056	323.6564	629.2790	315.1432			5
15	1542.6166	771.8119			1524.6060	762.8066	H	483.2423	242.1248	466.2157	233.6115			4

**16**	1656.6595	828.8334	1639.6329	820.3201	1638.6489	819.8281	**N**	***346.1833***	173.5953	329.1568	165.0820			**3**
**17**	1713.6809	857.3441	1696.6544	848.8308	1695.6704	848.3388	**G**	***232.1404***	116.5738	215.1139	108.0606			**2**
**18**							**R**	175.1190	88.0631	158.0924	79.5498			**1**

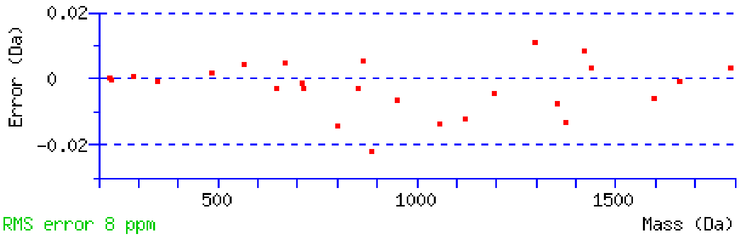

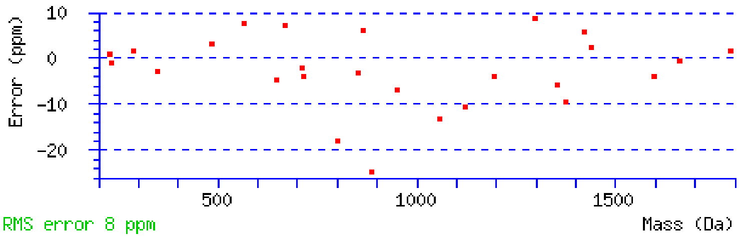

NCBI **BLAST** search of VEGYSGTAGDSMAYHNGR
(Parameters: blastp, nr protein database, expect=20000, no filter, PAM30)
Other BLAST web gateways

All matches to this query

Score	Mr(calc)	Delta	Sequence
119.7	1886.7853	0.0010	VEGYSGTAGDSMAYHNGR

Mascot: http://www.matrixscience.com/

Mascot Search Results

Peptide View

MS/MS Fragmentation of **VEGYSGTAGDSMAYHNGR**
Found in **P24821** in **uni_human_nr**, TENA_HUMAN Tenascin OS=Homo sapiens GN=TNC PE=1 SV=3

Match to Query 5981: 1871.774202 from(624.932010,3+) intensity(764445.6875) rtinseconds(1368) scans(6230) index(3921)
Title: 130801_HPL_Human_Plaque_BR2_TR1_11_Spectrum024108_scans__6230_RTINSECONDS=1368
Data file E:\\RAW\\HumanPlaques\\mgf\\T\\1D\\T130801_HPL_Human_Plaque_BR2_TR1_11.mgf

Click mouse within plot area to zoom in by factor of two about that point
Or, [Plot from] [0] to [1700] Da [Full range]
Label all possible matches ○ Label matches used for scoring ⦿
Show Y-axis ☐

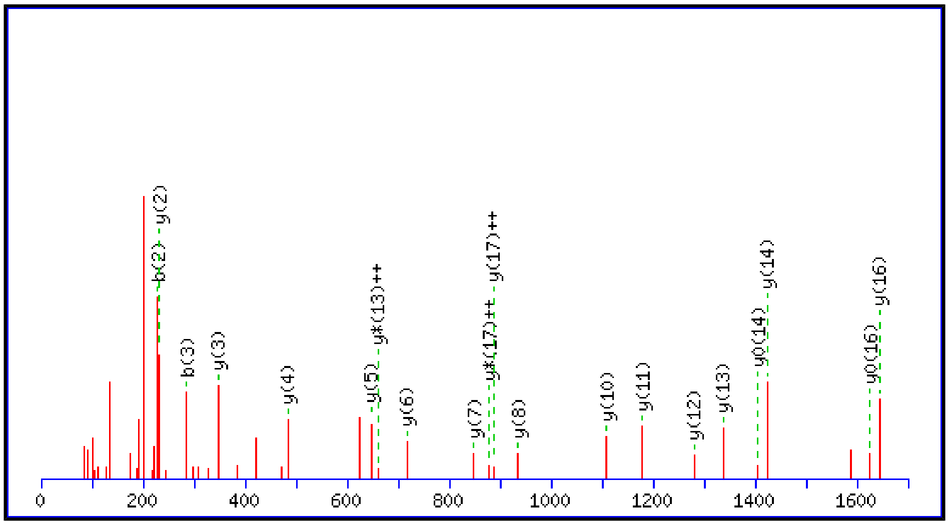

Monoisotopic mass of neutral peptide Mr(calc): 1871.7744
Fixed modifications: Carbamidomethyl (C) (apply to specified residues or termini only)
Variable modifications:
N16 : Deamidated (NQ)
Ions Score: 108 **Expect:** 1.7e-010
Matches : 20/158 fragment ions using 31 most intense peaks (help)

#	b	b^{++}	b*	b^{*++}	b^0	b^{0++}	Seq.	y	y^{++}	y*	y^{*++}	y^0	y^{0++}	#
1	100.0757	50.5415					V							18
2	229.1183	115.0628			211.1077	106.0575	E	1773.7133	887.3603	1756.6868	878.8470	1755.7027	878.3550	17
3	286.1397	143.5735			268.1292	134.5682	G	1644.6707	822.8390	1627.6442	814.3257	1626.6601	813.8337	16
4	449.2031	225.1052			431.1925	216.0999	Y	1587.6492	794.3283	1570.6227	785.8150	1569.6387	785.3230	15
5	536.2351	268.6212			518.2245	259.6159	S	1424.5859	712.7966	1407.5594	704.2833	1406.5754	703.7913	14
6	593.2566	297.1319			575.2460	288.1266	G	1337.5539	669.2806	1320.5273	660.7673	1319.5433	660.2753	13
7	694.3042	347.6558			676.2937	338.6505	T	1280.5324	640.7699	1263.5059	632.2566	1262.5219	631.7646	12
8	765.3414	383.1743			747.3308	374.1690	A	1179.4847	590.2460	1162.4582	581.7327	1161.4742	581.2407	11
9	822.3628	411.6851			804.3523	402.6798	G	1108.4476	554.7275	1091.4211	546.2142	1090.4371	545.7222	10
10	937.3898	469.1985			919.3792	460.1932	D	1051.4262	526.2167	1034.3996	517.7034	1033.4156	517.2114	9
11	1024.4218	512.7145			1006.4112	503.7093	S	936.3992	468.7033	919.3727	460.1900	918.3887	459.6980	8
12	1155.4623	578.2348			1137.4517	569.2295	M	849.3672	425.1872	832.3406	416.6740			7
13	1226.4994	613.7533			1208.4888	604.7481	A	718.3267	359.6670	701.3002	351.1537			6
14	1389.5627	695.2850			1371.5522	686.2797	Y	647.2896	324.1484	630.2631	315.6352			5
15	1526.6216	763.8145			1508.6111	754.8092	H	484.2263	242.6168	467.1997	234.1035			4

**16**	1641.6486	821.3279	1624.6220	812.8147	1623.6380	812.3226	**N**	*347.1674*	174.0873	330.1408	165.5740			**3**
**17**	1698.6700	849.8387	1681.6435	841.3254	1680.6595	840.8334	**G**	*232.1404*	116.5738	215.1139	108.0606			**2**
**18**							**R**	175.1190	88.0631	158.0924	79.5498			**1**

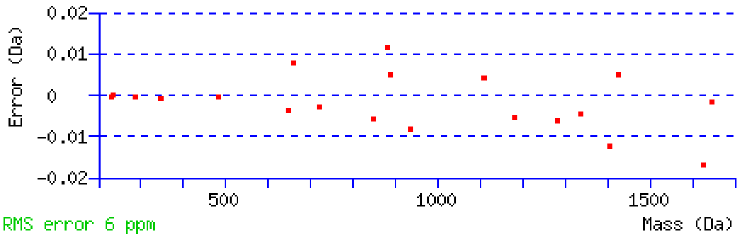

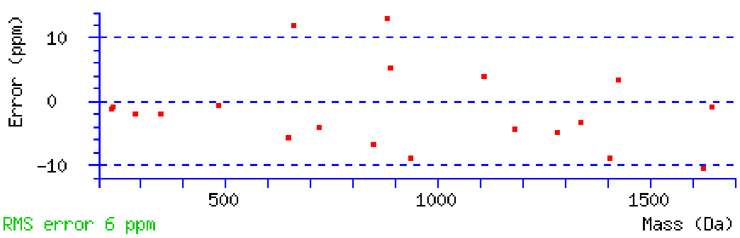

NCBI **BLAST** search of VEGYSGTAGDSMAYHNGR
(Parameters: blastp, nr protein database, expect=20000, no filter, PAM30)
Other BLAST web gateways

All matches to this query

Score	Mr(calc)	Delta	Sequence
107.6	1871.7744	-0.0002	VEGYSGTAGDSMAYHNGR

Mascot: http://www.matrixscience.com/

Mascot Search Results

Peptide View

MS/MS Fragmentation of **VEGYSGTAGDSMAYHNGR**
Found in **P24821** in **uni_human_nr**, TENA_HUMAN Tenascin OS=Homo sapiens GN=TNC PE=1 SV=3

Match to Query 7735: 1870.790008 from(936.402280,2+) intensity(7641343.5000) rtinseconds(1186) scans(5462) index(3484)
Title: 130801_HPL_Human_Plaque_BR2_TR1_04_Spectrum026058_scans__5462_RTINSECONDS=1186
Data file E:\\RAW\\HumanPlaques\\mgf\\T\\1D\\T130801_HPL_Human_Plaque_BR2_TR1_04.mgf

Click mouse within plot area to zoom in by factor of two about that point
Or, Plot from 0 to 1900 Da Full range
Label all possible matches ○ Label matches used for scoring ⦿
Show Y-axis ☐

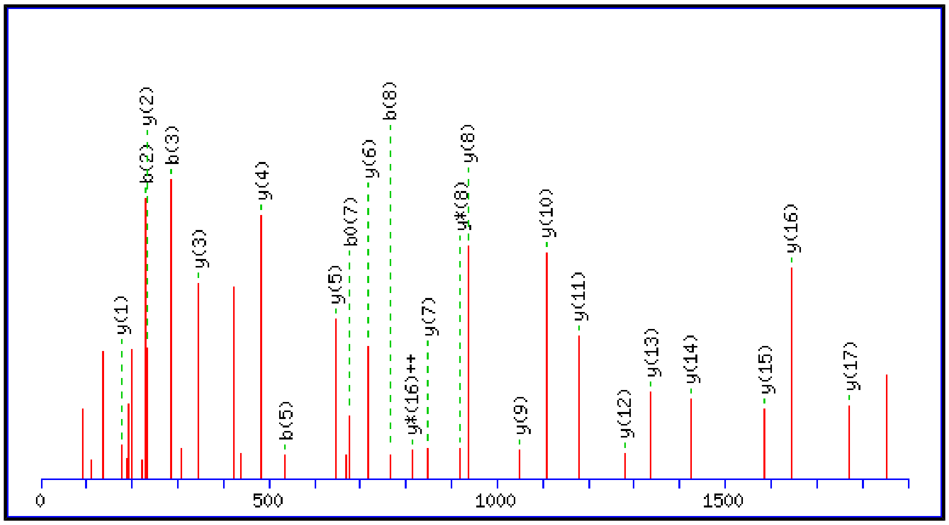

Monoisotopic mass of neutral peptide Mr(calc): 1870.7904
Fixed modifications: Carbamidomethyl (C) (apply to specified residues or termini only)
Ions Score: 182 **Expect:** 1.1e-017
Matches : 24/158 fragment ions using 32 most intense peaks (help)

#	b	b^{++}	b*	b*$^{++}$	b^{0}	b^{0++}	Seq.	y	y^{++}	y*	y*$^{++}$	y^{0}	y^{0++}	#
1	100.0757	50.5415					V							18
2	**229.1183**	115.0628			211.1077	106.0575	E	***1772.7293***	886.8683	1755.7027	878.3550	1754.7187	877.8630	17
3	**286.1397**	143.5735			268.1292	134.5682	G	***1643.6867***	822.3470	1626.6601	813.8337	1625.6761	813.3417	16
4	449.2031	225.1052			431.1925	216.0999	Y	***1586.6652***	793.8363	1569.6387	785.3230	1568.6547	784.8310	15
5	**536.2351**	268.6212			518.2245	259.6159	S	***1423.6019***	712.3046	1406.5754	703.7913	1405.5913	703.2993	14
6	593.2566	297.1319			575.2460	288.1266	G	***1336.5699***	668.7886	1319.5433	660.2753	1318.5593	659.7833	13
7	694.3042	347.6558			676.2937	338.6505	T	***1279.5484***	640.2778	1262.5219	631.7646	1261.5378	631.2726	12
8	**765.3414**	383.1743			747.3308	374.1690	A	***1178.5007***	589.7540	1161.4742	581.2407	1160.4902	580.7487	11
9	822.3628	411.6851			804.3523	402.6798	G	***1107.4636***	554.2354	1090.4371	545.7222	1089.4531	545.2302	10
10	937.3898	469.1985			919.3792	460.1932	D	***1050.4422***	525.7247	1033.4156	517.2114	1032.4316	516.7194	9
11	1024.4218	512.7145			1006.4112	503.7093	S	***935.4152***	468.2112	918.3887	459.6980	917.4046	459.2060	8
12	1155.4623	578.2348			1137.4517	569.2295	M	***848.3832***	424.6952	831.3566	416.1820			7
13	1226.4994	613.7533			1208.4888	604.7481	A	***717.3427***	359.1750	700.3161	350.6617			6
14	1389.5627	695.2850			1371.5522	686.2797	Y	***646.3056***	323.6564	629.2790	315.1432			5
15	1526.6216	763.8145			1508.6111	754.8092	H	***483.2423***	242.1248	466.2157	233.6115			4
16	1640.6646	820.8359	1623.6380	812.3226	1622.6540	811.8306	N	***346.1833***	173.5953	329.1568	165.0820			3

**17**	1697.6860	849.3467	1680.6595	840.8334	1679.6755	840.3414	**G**	***232.1404***	116.5738	215.1139	108.0606			**2**
**18**							**R**	***175.1190***	88.0631	158.0924	79.5498			**1**

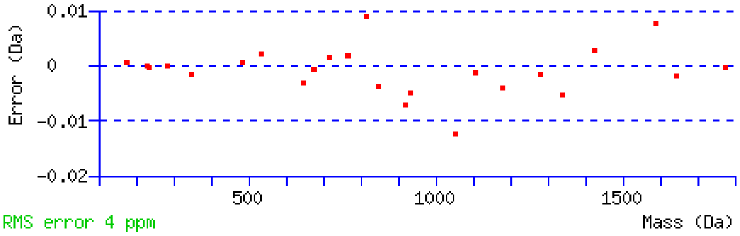

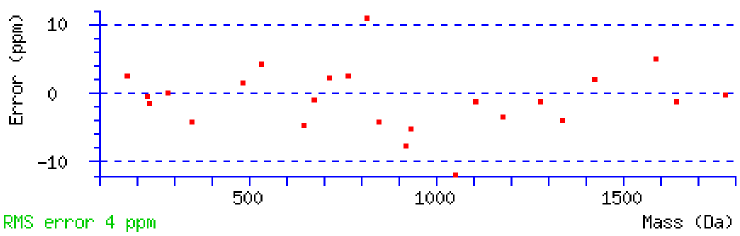

NCBI **BLAST** search of VEGYSGTAGDSMAYHNGR
(Parameters: blastp, nr protein database, expect=20000, no filter, PAM30)
Other BLAST web gateways

All matches to this query

Score	Mr(calc)	Delta	Sequence
182.1	1870.7904	-0.0004	VEGYSGTAGDSMAYHNGR

**Mascot:** http://www.matrixscience.com/

Mascot Search Results

Peptide View

MS/MS Fragmentation of **VEGYSGTAGDSMAYHNGR**
Found in **P24821** in **uni_human_nr**, TENA_HUMAN Tenascin OS=Homo sapiens GN=TNC PE=1 SV=3

Match to Query 7736: 1870.791282 from(624.604370,3+) intensity(76699016.0000) rtinseconds(1186) scans(5466) index(3488)
Title: 130801_HPL_Human_Plaque_BR2_TR1_04_Spectrum026062_scans__5466_RTINSECONDS=1186
Data file E:\\RAW\\HumanPlaques\\mgf\\T\\1D\\T130801_HPL_Human_Plaque_BR2_TR1_04.mgf

Click mouse within plot area to zoom in by factor of two about that point
Or, Plot from 0 to 1800 Da Full range
Label all possible matches ○ Label matches used for scoring ⊙
Show Y-axis ☐

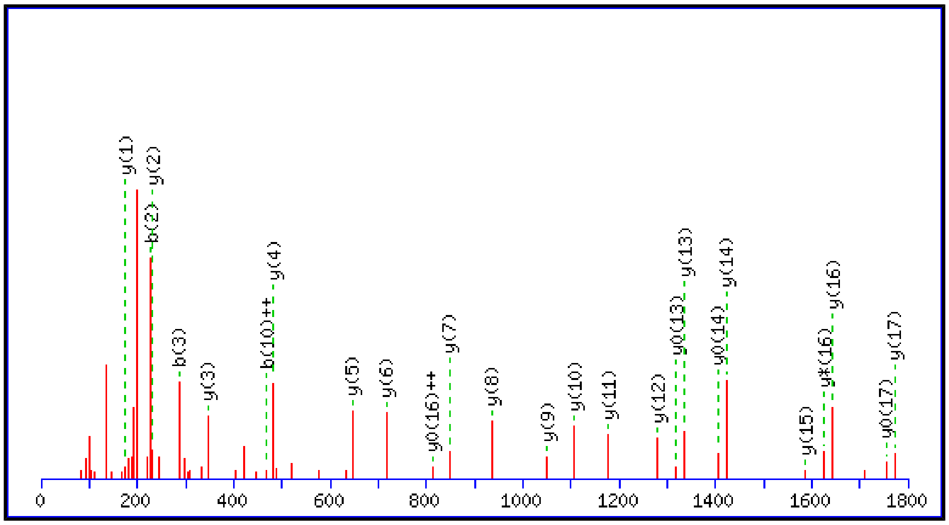

Monoisotopic mass of neutral peptide Mr(calc): 1870.7904
Fixed modifications: Carbamidomethyl (C) (apply to specified residues or termini only)
Ions Score: 148 **Expect:** 2.7e-014
Matches : 25/158 fragment ions using 46 most intense peaks (help)

#	b	b^{++}	b*	b^{*++}	b^{0}	b^{0++}	Seq.	y	y^{++}	y*	y^{*++}	y^{0}	y^{0++}	#
1	100.0757	50.5415					V							18
2	**229.1183**	115.0628			211.1077	106.0575	E	***1772.7293***	886.8683	1755.7027	878.3550	1754.7187	877.8630	17
3	**286.1397**	143.5735			268.1292	134.5682	G	***1643.6867***	822.3470	1626.6601	813.8337	1625.6761	813.3417	16
4	449.2031	225.1052			431.1925	216.0999	Y	***1586.6652***	793.8363	1569.6387	785.3230	1568.6547	784.8310	15
5	536.2351	268.6212			518.2245	259.6159	S	***1423.6019***	712.3046	1406.5754	703.7913	1405.5913	703.2993	14
6	593.2566	297.1319			575.2460	288.1266	G	***1336.5699***	668.7886	1319.5433	660.2753	1318.5593	659.7833	13
7	694.3042	347.6558			676.2937	338.6505	T	***1279.5484***	640.2778	1262.5219	631.7646	1261.5378	631.2726	12
8	765.3414	383.1743			747.3308	374.1690	A	***1178.5007***	589.7540	1161.4742	581.2407	1160.4902	580.7487	11
9	822.3628	411.6851			804.3523	402.6798	G	***1107.4636***	554.2354	1090.4371	545.7222	1089.4531	545.2302	10
10	937.3898	**469.1985**			919.3792	460.1932	D	***1050.4422***	525.7247	1033.4156	517.2114	1032.4316	516.7194	9
11	1024.4218	512.7145			1006.4112	503.7093	S	***935.4152***	468.2112	918.3887	459.6980	917.4046	459.2060	8
12	1155.4623	578.2348			1137.4517	569.2295	M	***848.3832***	424.6952	831.3566	416.1820			7
13	1226.4994	613.7533			1208.4888	604.7481	A	***717.3427***	359.1750	700.3161	350.6617			6
14	1389.5627	695.2850			1371.5522	686.2797	Y	***646.3056***	323.6564	629.2790	315.1432			5
15	1526.6216	763.8145			1508.6111	754.8092	H	***483.2423***	242.1248	466.2157	233.6115			4
16	1640.6646	820.8359	1623.6380	812.3226	1622.6540	811.8306	N	***346.1833***	173.5953	329.1568	165.0820			3

**17**	1697.6860	849.3467	1680.6595	840.8334	1679.6755	840.3414	**G**	*232.1404*	116.5738	215.1139	108.0606			**2**
**18**							**R**	*175.1190*	88.0631	158.0924	79.5498			**1**

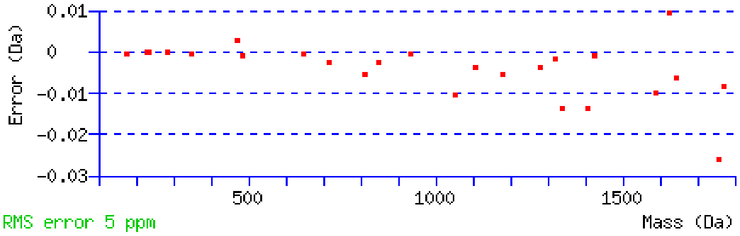

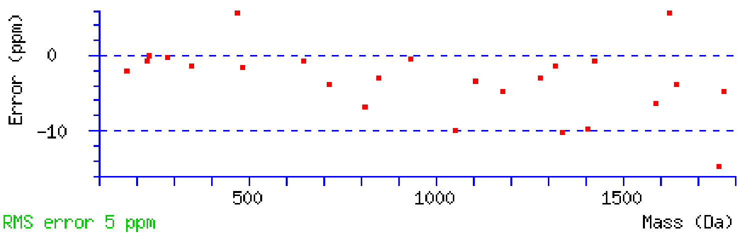

NCBI **BLAST** search of VEGYSGTAGDSMAYHNGR
(Parameters: blastp, nr protein database, expect=20000, no filter, PAM30)
Other BLAST web gateways

All matches to this query

Score	Mr(calc)	Delta	Sequence
148.3	1870.7904	0.0009	VEGYSGTAGDSMAYHNGR

Mascot: http://www.matrixscience.com/

Mascot Search Results

Peptide View

MS/MS Fragmentation of **YKLKVEGYSGTAGDSMAYHNGR**
Found in **P24821** in **uni_human_nr**, TENA_HUMAN Tenascin OS=Homo sapiens GN=TNC PE=1 SV=3

Match to Query 13601: 2419.113976 from(605.785770,4+) intensity(4479103.5000) rtinseconds(1133) scans(4881) index(2802)
Title: 130801_HPL_Human_Plaque_BR1_TR1_02_Spectrum026809_scans__4881_RTINSECONDS=1133
Data file E:\\RAW\\HumanPlaques\\mgf\\T\\1D\\T130801_HPL_Human_Plaque_BR1_TR1_02.mgf

Click mouse within plot area to zoom in by factor of two about that point
Or, Plot from 0 to 2600 Da Full range
Label all possible matches ○ Label matches used for scoring ⊙
Show Y-axis ☐

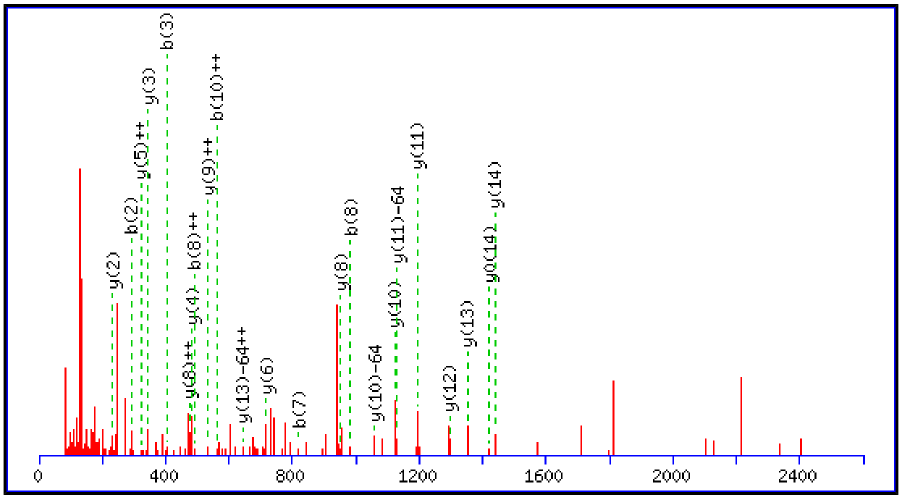

Monoisotopic mass of neutral peptide Mr(calc): 2419.1226
Fixed modifications: Carbamidomethyl (C) (apply to specified residues or termini only)
Variable modifications:
M16 : Oxidation (M), with neutral losses 0.0000(shown in table), 63.9983
Ions Score: 28 **Expect:** 0.091
Matches : 23/350 fragment ions using 73 most intense peaks (help)

#	b	b^{++}	b*	b^{*++}	b^0	b^{0++}	Seq.	y	y^{++}	y*	y^{*++}	y^0	y^{0++}	#
1	164.0706	82.5389					Y							22
2	**292.1656**	146.5864	275.1390	138.0731			K	2257.0666	1129.0369	2240.0401	1120.5237	2239.0560	1120.0317	21
3	**405.2496**	203.1285	388.2231	194.6152			L	2128.9716	1064.9895	2111.9451	1056.4762	2110.9611	1055.9842	20
4	533.3446	267.1759	516.3180	258.6627			K	2015.8876	1008.4474	1998.8610	999.9342	1997.8770	999.4421	19
5	632.4130	316.7101	615.3865	308.1969			V	1887.7926	944.3999	1870.7661	935.8867	1869.7821	935.3947	18
6	761.4556	381.2314	744.4291	372.7182	743.4450	372.2262	E	1788.7242	894.8657	1771.6977	886.3525	1770.7136	885.8605	17
7	**818.4771**	409.7422	801.4505	401.2289	800.4665	400.7369	G	1659.6816	830.3444	1642.6551	821.8312	1641.6710	821.3392	16
8	**981.5404**	**491.2738**	964.5138	482.7606	963.5298	482.2686	Y	1602.6601	801.8337	1585.6336	793.3204	1584.6496	792.8284	15
9	1068.5724	534.7898	1051.5459	526.2766	1050.5619	525.7846	S	***1439.5968***	720.3020	1422.5703	711.7888	1421.5863	711.2968	14
10	1125.5939	**563.3006**	1108.5673	554.7873	1107.5833	554.2953	G	***1352.5648***	676.7860	1335.5382	668.2728	1334.5542	667.7808	13
11	1226.6416	613.8244	1209.6150	605.3111	1208.6310	604.8191	T	***1295.5433***	648.2753	1278.5168	639.7620	1277.5328	639.2700	12
12	1297.6787	649.3430	1280.6521	640.8297	1279.6681	640.3377	A	***1194.4956***	597.7515	1177.4691	589.2382	1176.4851	588.7462	11
13	1354.7001	677.8537	1337.6736	669.3404	1336.6896	668.8484	G	***1123.4585***	562.2329	1106.4320	553.7196	1105.4480	553.2276	10
14	1469.7271	735.3672	1452.7005	726.8539	1451.7165	726.3619	D	1066.4371	533.7222	1049.4105	525.2089	1048.4265	524.7169	9
15	1556.7591	778.8832	1539.7326	770.3699	1538.7486	769.8779	S	***951.4101***	476.2087	934.3836	467.6954	933.3996	467.2034	8
16	1703.7945	852.4009	1686.7680	843.8876	1685.7840	843.3956	M	864.3781	432.6927	847.3515	424.1794			7
17	1774.8316	887.9195	1757.8051	879.4062	1756.8211	878.9142	A	***717.3427***	359.1750	700.3161	350.6617			6
18	1937.8950	969.4511	1920.8684	960.9378	1919.8844	960.4458	Y	646.3056	323.6564	629.2790	315.1432			5

**19**	2074.9539	1037.9806	2057.9273	1029.4673	2056.9433	1028.9753	**H**	***483.2423***	242.1248	466.2157	233.6115			**4**
**20**	2188.9968	1095.0020	2171.9702	1086.4888	2170.9862	1085.9968	**N**	***346.1833***	173.5953	329.1568	165.0820			**3**
**21**	2246.0183	1123.5128	2228.9917	1114.9995	2228.0077	1114.5075	**G**	***232.1404***	116.5738	215.1139	108.0606			**2**
**22**							**R**	175.1190	88.0631	158.0924	79.5498			**1**

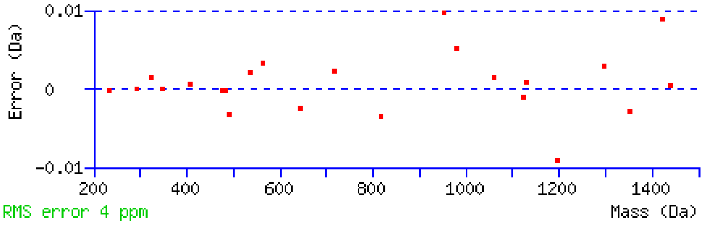

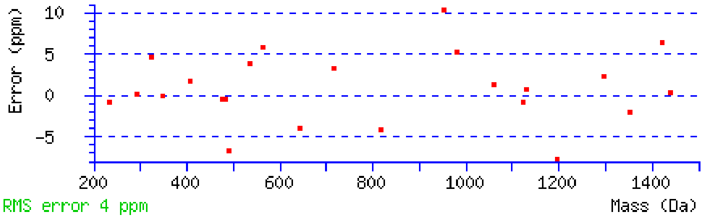

NCBI **BLAST** search of YKLKVEGYSGTAGDSMAYHNGR
(Parameters: blastp, nr protein database, expect=20000, no filter, PAM30)
Other BLAST web gateways

All matches to this query

Score	Mr(calc)	Delta	Sequence
28.2	2419.1226	-0.0087	YKLKVEGYSGTAGDSMAYHNGR

**Mascot:** http://www.matrixscience.com/

Mascot Search Results

Peptide View

MS/MS Fragmentation of **YKLKVEGYSGTAGDSMAYHNGR**
Found in **P24821** in **uni_human_nr**, TENA_HUMAN Tenascin OS=Homo sapiens GN=TNC PE=1 SV=3

Match to Query 13834: 2403.122016 from(601.787780,4+) intensity(18476948.0000) rtinseconds(1306) scans(6589) index(4499)
Title: 130806_HPL_Human_Plaque_BR1_TR2_02_Spectrum029203_scans__6589_RTINSECONDS=1306
Data file E:\\RAW\\HumanPlaques\\mgf\\T\\1D\\T130806_HPL_Human_Plaque_BR1_TR2_02.mgf

Click mouse within plot area to zoom in by factor of two about that point
Or, Plot from 0 to 2400 Da Full range
Label all possible matches ○ Label matches used for scoring ⦿
Show Y-axis ☐

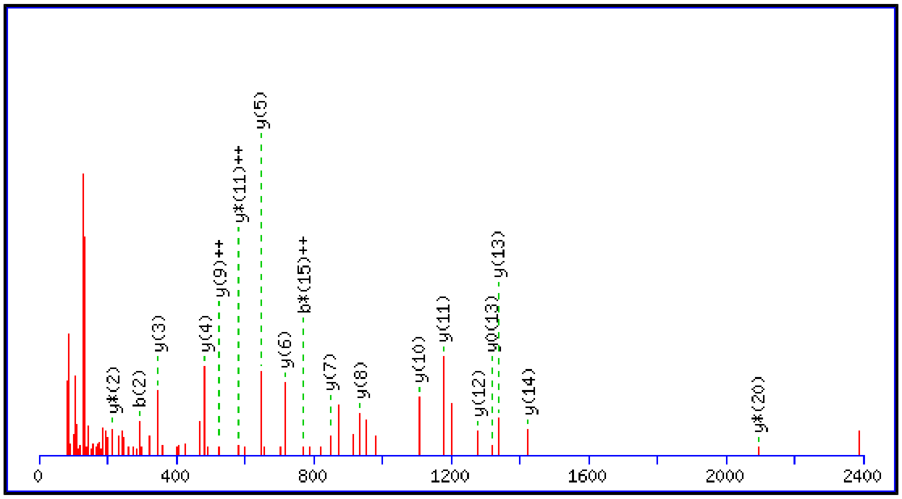

Monoisotopic mass of neutral peptide Mr(calc): 2403.1277
Fixed modifications: Carbamidomethyl (C) (apply to specified residues or termini only)
Ions Score: 79 **Expect:** 8.6e-007
Matches : 18/226 fragment ions using 27 most intense peaks (help)

#	b	b^{++}	b*	b^{*++}	b^0	b^{0++}	Seq.	y	y^{++}	y*	y^{*++}	y^0	y^{0++}	#
1	164.0706	82.5389					Y							22
2	**292.1656**	146.5864	275.1390	138.0731			K	2241.0717	1121.0395	2224.0451	1112.5262	2223.0611	1112.0342	21
3	405.2496	203.1285	388.2231	194.6152			L	2112.9767	1056.9920	2095.9502	1048.4787	2094.9662	1047.9867	20
4	533.3446	267.1759	516.3180	258.6627			K	1999.8927	1000.4500	1982.8661	991.9367	1981.8821	991.4447	19
5	632.4130	316.7101	615.3865	308.1969			V	1871.7977	936.4025	1854.7712	927.8892	1853.7871	927.3972	18
6	761.4556	381.2314	744.4291	372.7182	743.4450	372.2262	E	1772.7293	886.8683	1755.7027	878.3550	1754.7187	877.8630	17
7	818.4771	409.7422	801.4505	401.2289	800.4665	400.7369	G	1643.6867	822.3470	1626.6601	813.8337	1625.6761	813.3417	16
8	981.5404	491.2738	964.5138	482.7606	963.5298	482.2686	Y	1586.6652	793.8363	1569.6387	785.3230	1568.6547	784.8310	15
9	1068.5724	534.7898	1051.5459	526.2766	1050.5619	525.7846	S	***1423.6019***	712.3046	1406.5754	703.7913	1405.5913	703.2993	14
10	1125.5939	563.3006	1108.5673	554.7873	1107.5833	554.2953	G	***1336.5699***	668.7886	1319.5433	660.2753	1318.5593	659.7833	13
11	1226.6416	613.8244	1209.6150	605.3111	1208.6310	604.8191	T	***1279.5484***	640.2778	1262.5219	631.7646	1261.5378	631.2726	12
12	1297.6787	649.3430	1280.6521	640.8297	1279.6681	640.3377	A	***1178.5007***	589.7540	1161.4742	581.2407	1160.4902	580.7487	11
13	1354.7001	677.8537	1337.6736	669.3404	1336.6896	668.8484	G	***1107.4636***	554.2354	1090.4371	545.7222	1089.4531	545.2302	10
14	1469.7271	735.3672	1452.7005	726.8539	1451.7165	726.3619	D	1050.4422	525.7247	1033.4156	517.2114	1032.4316	516.7194	9
15	1556.7591	778.8832	1539.7326	770.3699	1538.7486	769.8779	S	***935.4152***	468.2112	918.3887	459.6980	917.4046	459.2060	8
16	1687.7996	844.4034	1670.7731	835.8902	1669.7890	835.3982	M	***848.3832***	424.6952	831.3566	416.1820			7
17	1758.8367	879.9220	1741.8102	871.4087	1740.8261	870.9167	A	***717.3427***	359.1750	700.3161	350.6617			6
18	1921.9000	961.4537	1904.8735	952.9404	1903.8895	952.4484	Y	***646.3056***	323.6564	629.2790	315.1432			5
19	2058.9590	1029.9831	2041.9324	1021.4698	2040.9484	1020.9778	H	***483.2423***	242.1248	466.2157	233.6115			4

**20**	2173.0019	1087.0046	2155.9753	1078.4913	2154.9913	1077.9993	**N**	***346.1833***	173.5953	329.1568	165.0820			**3**
**21**	2230.0233	1115.5153	2212.9968	1107.0020	2212.0128	1106.5100	**G**	232.1404	116.5738	215.1139	108.0606			**2**
**22**							**R**	175.1190	88.0631	158.0924	79.5498			**1**

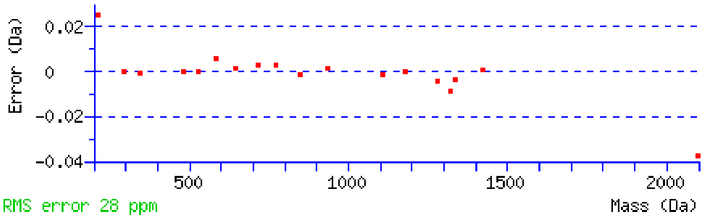

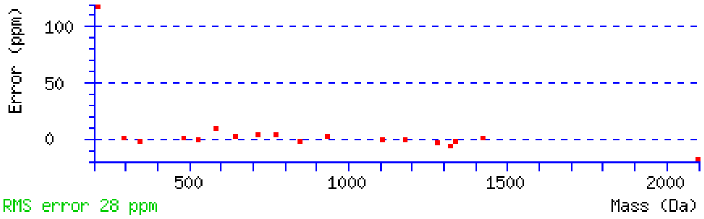

NCBI **BLAST** search of YKLKVEGYSGTAGDSMAYHNGR
(Parameters: blastp, nr protein database, expect=20000, no filter, PAM30)
Other BLAST web gateways

All matches to this query

Score	Mr(calc)	Delta	Sequence
79.0	2403.1277	-0.0057	YKLKVEGYSGTAGDSMAYHNGR
1.0	2401.1082	2.0138	KGEMLNTFCGSPPYAAPELFR

Mascot: http://www.matrixscience.com/

Mascot Search Results

Peptide View

MS/MS Fragmentation of **GALCLLAEDDSSCEVNGR**
Found in **O76076** in **uni_human_nr**, WISP2_HUMAN WNT1-inducible-signaling pathway protein 2 OS=Homo sapiens GN=WISP2 PE=1 SV=1

Match to Query 8558: 1965.842868 from(983.928710,2+) intensity(1426114.8750) rtinseconds(2284) scans(12457) index(9601)
Title: 130809_HPL_Human_Plaque_BR2_TR3_20_Spectrum028790_scans__12457_RTINSECONDS=2284
Data file E:\\RAW\\HumanPlaques\\mgf\\T\\1D\\T130809_HPL_Human_Plaque_BR2_TR3_20.mgf

Click mouse within plot area to zoom in by factor of two about that point
Or, Plot from 0 to 1500 Da Full range
Label all possible matches ○ Label matches used for scoring ⦿
Show Y-axis ☐

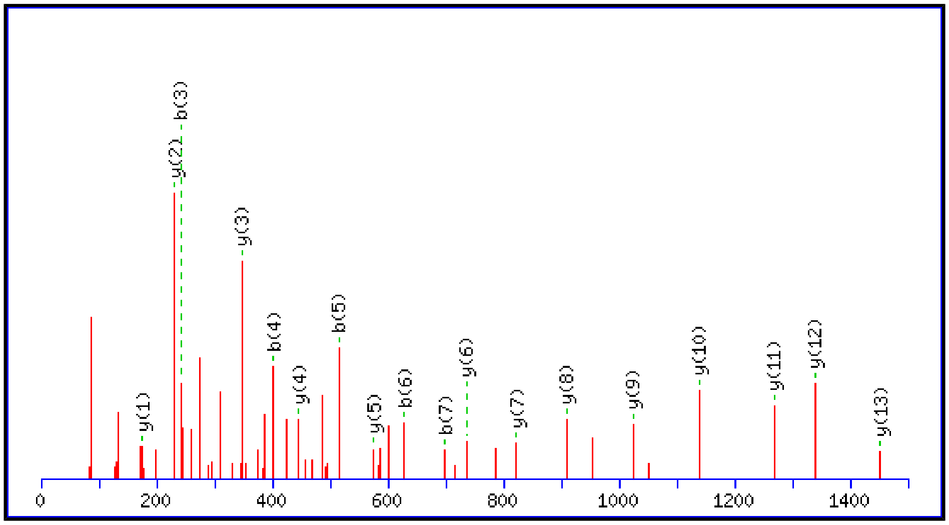

Monoisotopic mass of neutral peptide Mr(calc): 1965.8408
Fixed modifications: Carbamidomethyl (C) (apply to specified residues or termini only)
Variable modifications:
N16 : Deamidated (NQ)
Ions Score: 99 **Expect:** 2.7e-009
Matches : 18/152 fragment ions using 31 most intense peaks (help)

#	b	b^{++}	b*	b^{*++}	b^0	b^{0++}	Seq.	y	y^{++}	y*	y^{*++}	y^0	y^{0++}	#
1	58.0287	29.5180					G							18
2	129.0659	65.0366					A	1909.8266	955.4169	1892.8001	946.9037	1891.8161	946.4117	17
3	**242.1499**	121.5786					L	1838.7895	919.8984	1821.7630	911.3851	1820.7789	910.8931	16
4	**402.1806**	201.5939					C	1725.7054	863.3564	1708.6789	854.8431	1707.6949	854.3511	15
5	**515.2646**	258.1360					L	1565.6748	783.3410	1548.6482	774.8278	1547.6642	774.3358	14
6	**628.3487**	314.6780					L	***1452.5907***	726.7990	1435.5642	718.2857	1434.5802	717.7937	13
7	**699.3858**	350.1965					A	***1339.5067***	670.2570	1322.4801	661.7437	1321.4961	661.2517	12
8	828.4284	414.7178			810.4178	405.7126	E	***1268.4696***	634.7384	1251.4430	626.2251	1250.4590	625.7331	11
9	943.4553	472.2313			925.4448	463.2260	D	***1139.4270***	570.2171	1122.4004	561.7038	1121.4164	561.2118	10
10	1058.4823	529.7448			1040.4717	520.7395	D	***1024.4000***	512.7036	1007.3735	504.1904	1006.3894	503.6984	9
11	1145.5143	573.2608			1127.5038	564.2555	S	***909.3731***	455.1902	892.3465	446.6769	891.3625	446.1849	8
12	1232.5463	616.7768			1214.5358	607.7715	S	***822.3410***	411.6742	805.3145	403.1609	804.3305	402.6689	7
13	1392.5770	696.7921			1374.5664	687.7869	C	***735.3090***	368.1581	718.2825	359.6449	717.2985	359.1529	6
14	1521.6196	761.3134			1503.6090	752.3081	E	***575.2784***	288.1428	558.2518	279.6295	557.2678	279.1375	5
15	1620.6880	810.8476			1602.6774	801.8424	V	***446.2358***	223.6215	429.2092	215.1083			4

**16**	1735.7149	868.3611	1718.6884	859.8478	1717.7044	859.3558	**N**	***347.1674***	174.0873	330.1408	165.5740			**3**
**17**	1792.7364	896.8718	1775.7099	888.3586	1774.7258	887.8666	**G**	***232.1404***	116.5738	215.1139	108.0606			**2**
**18**							**R**	***175.1190***	88.0631	158.0924	79.5498			**1**

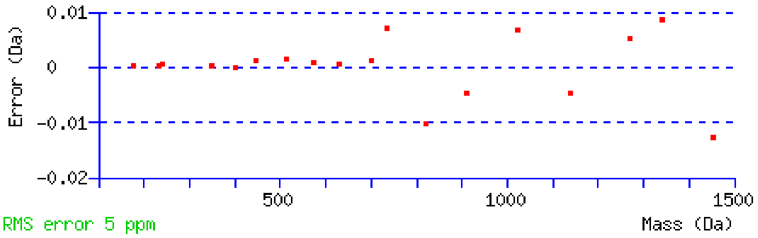

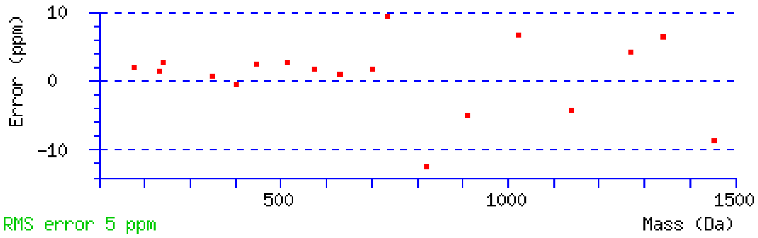

NCBI **BLAST** search of GALCLLAEDDSSCEVNGR
(Parameters: blastp, nr protein database, expect=20000, no filter, PAM30)
Other BLAST web gateways

All matches to this query

Score	Mr(calc)	Delta	Sequence
98.8	1965.8408	0.0021	GALCLLAEDDSSCEVNGR

**Mascot:** http://www.matrixscience.com/

Mascot Search Results

Peptide View

MS/MS Fragmentation of **GALCLLAEDDSSCEVNGR**
Found in **O76076** in **uni_human_nr**, WISP2_HUMAN WNT1-inducible-signaling pathway protein 2 OS=Homo sapiens GN=WISP2 PE=1 SV=1

Match to Query 8482: 1965.844272 from(656.288700,3+) intensity(146933.0781) rtinseconds(2277) scans(12304) index(9435)
Title: 130801_HPL_Human_Plaque_BR2_TR1_20_Spectrum028337_scans__12304_RTINSECONDS=2277
Data file E:\\RAW\\HumanPlaques\\mgf\\T\\1D\\T130801_HPL_Human_Plaque_BR2_TR1_20.mgf

Click mouse within plot area to zoom in by factor of two about that point
Or, [Plot from] [0] to [1300] Da [Full range]
Label all possible matches ○ Label matches used for scoring ⊙
Show Y-axis ☐

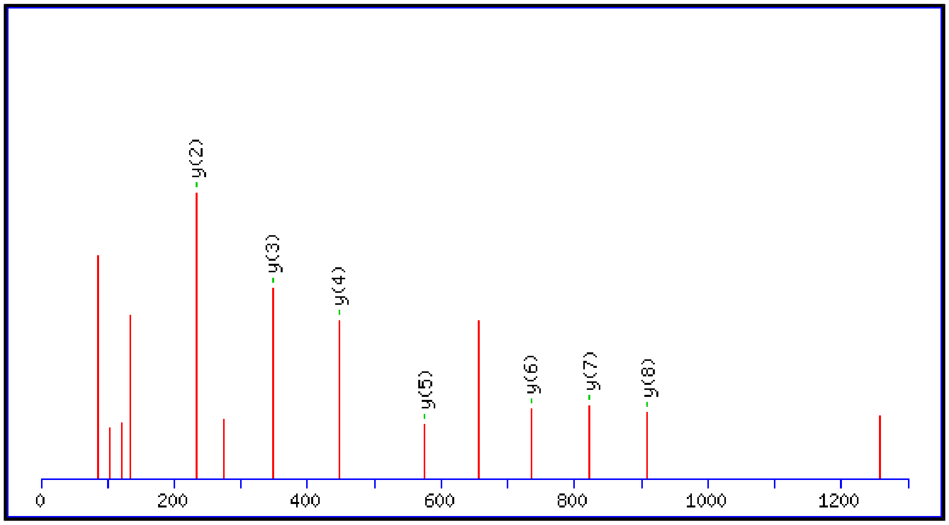

Monoisotopic mass of neutral peptide Mr(calc): 1965.8408
Fixed modifications: Carbamidomethyl (C) (apply to specified residues or termini only)
Variable modifications:
N16 : Deamidated (NQ)
Ions Score: 52 **Expect:** 0.00014
Matches : 7/152 fragment ions using 10 most intense peaks (help)

#	b	b^{++}	b*	b^{*++}	b^0	b^{0++}	Seq.	y	y^{++}	y*	y^{*++}	y^0	y^{0++}	#
1	58.0287	29.5180					G							18
2	129.0659	65.0366					A	1909.8266	955.4169	1892.8001	946.9037	1891.8161	946.4117	17
3	242.1499	121.5786					L	1838.7895	919.8984	1821.7630	911.3851	1820.7789	910.8931	16
4	402.1806	201.5939					C	1725.7054	863.3564	1708.6789	854.8431	1707.6949	854.3511	15
5	515.2646	258.1360					L	1565.6748	783.3410	1548.6482	774.8278	1547.6642	774.3358	14
6	628.3487	314.6780					L	1452.5907	726.7990	1435.5642	718.2857	1434.5802	717.7937	13
7	699.3858	350.1965					A	1339.5067	670.2570	1322.4801	661.7437	1321.4961	661.2517	12
8	828.4284	414.7178			810.4178	405.7126	E	1268.4696	634.7384	1251.4430	626.2251	1250.4590	625.7331	11
9	943.4553	472.2313			925.4448	463.2260	D	1139.4270	570.2171	1122.4004	561.7038	1121.4164	561.2118	10
10	1058.4823	529.7448			1040.4717	520.7395	D	1024.4000	512.7036	1007.3735	504.1904	1006.3894	503.6984	9
11	1145.5143	573.2608			1127.5038	564.2555	S	***909.3731***	455.1902	892.3465	446.6769	891.3625	446.1849	8
12	1232.5463	616.7768			1214.5358	607.7715	S	***822.3410***	411.6742	805.3145	403.1609	804.3305	402.6689	7
13	1392.5770	696.7921			1374.5664	687.7869	C	***735.3090***	368.1581	718.2825	359.6449	717.2985	359.1529	6
14	1521.6196	761.3134			1503.6090	752.3081	E	***575.2784***	288.1428	558.2518	279.6295	557.2678	279.1375	5
15	1620.6880	810.8476			1602.6774	801.8424	V	***446.2358***	223.6215	429.2092	215.1083			4

**16**	1735.7149	868.3611	1718.6884	859.8478	1717.7044	859.3558	**N**	***347.1674***	174.0873	330.1408	165.5740			**3**
**17**	1792.7364	896.8718	1775.7099	888.3586	1774.7258	887.8666	**G**	***232.1404***	116.5738	215.1139	108.0606			**2**
**18**							**R**	175.1190	88.0631	158.0924	79.5498			**1**

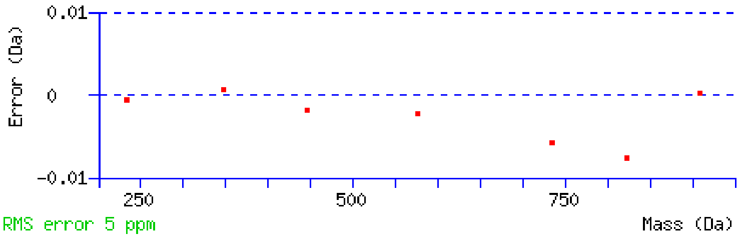

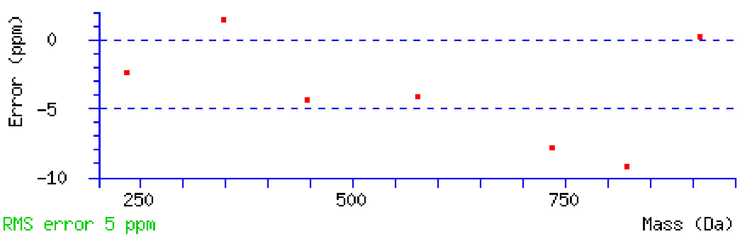

NCBI **BLAST** search of GALCLLAEDDSSCEVNGR
(Parameters: blastp, nr protein database, expect=20000, no filter, PAM30)
Other BLAST web gateways

All matches to this query

Score	Mr(calc)	Delta	Sequence
52.0	1965.8408	0.0035	GALCLLAEDDSSCEVNGR
0.3	1963.8370	2.0073	NFPQQYDLCIHAQNGR
0.3	1963.8370	2.0073	NFPQQYDLCIHAQNGR
0.3	1963.8370	2.0073	NFPQQYDLCIHAQNGR
0.1	1963.8370	2.0073	NFPQQYDLCIHAQNGR

**Mascot:** http://www.matrixscience.com/

Mascot Search Results

Peptide View

MS/MS Fragmentation of **GALCLLAEDDSSCEVNGR**
Found in **O76076** in **uni_human_nr**, WISP2_HUMAN WNT1-inducible-signaling pathway protein 2 OS=Homo sapiens GN=WISP2 PE=1 SV=1

Match to Query 6649: 1964.855688 from(983.435120,2+) intensity(2660228.7500) rtinseconds(2179) scans(11708) index(8578)
Title: 130806_HPL_Human_Plaque_BR1_TR2_16_Spectrum028599_scans__11708_RTINSECONDS=2179
Data file E:\\RAW\\HumanPlaques\\mgf\\T\\1D\\T130806_HPL_Human_Plaque_BR1_TR2_16.mgf

Click mouse within plot area to zoom in by factor of two about that point
Or, Plot from 0 to 1800 Da Full range
Label all possible matches ○ Label matches used for scoring ◉
Show Y-axis ☐

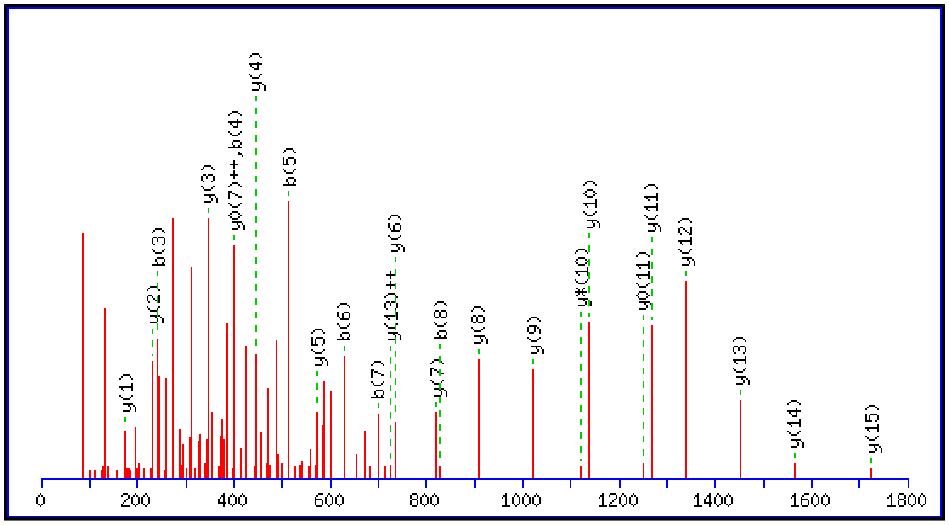

Monoisotopic mass of neutral peptide Mr(calc): 1964.8568
Fixed modifications: Carbamidomethyl (C) (apply to specified residues or termini only)
Ions Score: 121 **Expect:** 2.6e-011
Matches : 25/152 fragment ions using 40 most intense peaks (help)

#	b	b^{++}	b*	b^{*++}	b^{0}	b^{0++}	Seq.	y	y^{++}	y*	y^{*++}	y^{0}	y^{0++}	#
1	58.0287	29.5180					G							18
2	129.0659	65.0366					A	1908.8426	954.9249	1891.8161	946.4117	1890.8320	945.9197	17
3	**242.1499**	121.5786					L	1837.8055	919.4064	1820.7789	910.8931	1819.7949	910.4011	16
4	**402.1806**	201.5939					C	***1724.7214***	862.8644	1707.6949	854.3511	1706.7109	853.8591	15
5	**515.2646**	258.1360					L	***1564.6908***	782.8490	1547.6642	774.3358	1546.6802	773.8437	14
6	**628.3487**	314.6780					L	***1451.6067***	726.3070	1434.5802	717.7937	1433.5961	717.3017	13
7	**699.3858**	350.1965					A	***1338.5226***	669.7650	1321.4961	661.2517	1320.5121	660.7597	12
8	**828.4284**	414.7178			810.4178	405.7126	E	***1267.4855***	634.2464	1250.4590	625.7331	1249.4750	625.2411	11
9	943.4553	472.2313			925.4448	463.2260	D	***1138.4429***	569.7251	1121.4164	561.2118	1120.4324	560.7198	10
10	1058.4823	529.7448			1040.4717	520.7395	D	***1023.4160***	512.2116	1006.3894	503.6984	1005.4054	503.2064	9
11	1145.5143	573.2608			1127.5038	564.2555	S	***908.3891***	454.6982	891.3625	446.1849	890.3785	445.6929	8
12	1232.5463	616.7768			1214.5358	607.7715	S	***821.3570***	411.1822	804.3305	402.6689	803.3465	402.1769	7
13	1392.5770	696.7921			1374.5664	687.7869	C	***734.3250***	367.6661	717.2985	359.1529	716.3144	358.6609	6
14	1521.6196	761.3134			1503.6090	752.3081	E	***574.2944***	287.6508	557.2678	279.1375	556.2838	278.6455	5
15	1620.6880	810.8476			1602.6774	801.8424	V	***445.2518***	223.1295	428.2252	214.6162			4
16	1734.7309	867.8691	1717.7044	859.3558	1716.7204	858.8638	N	***346.1833***	173.5953	329.1568	165.0820			3

**17**	1791.7524	896.3798	1774.7258	887.8666	1773.7418	887.3746	**G**	***232.1404***	116.5738	215.1139	108.0606			**2**
**18**							**R**	***175.1190***	88.0631	158.0924	79.5498			**1**

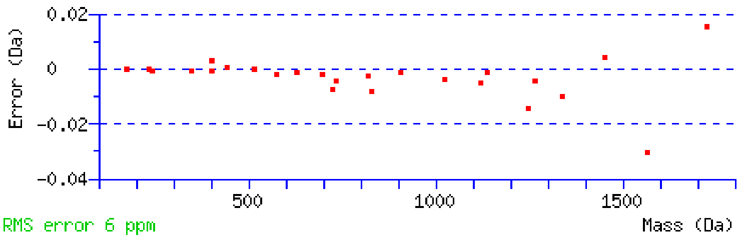

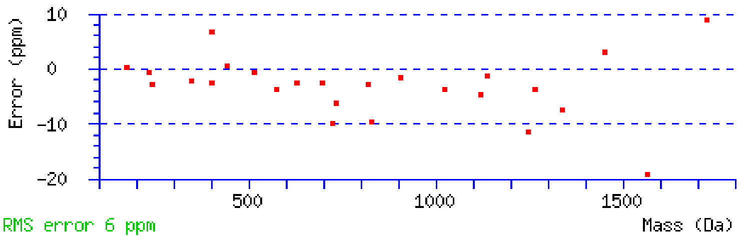

NCBI **BLAST** search of GALCLLAEDDSSCEVNGR
(Parameters: blastp, nr protein database, expect=20000, no filter, PAM30)
Other BLAST web gateways

All matches to this query

Score	Mr(calc)	Delta	Sequence
120.7	1964.8568	-0.0011	GALCLLAEDDSSCEVNGR

**Mascot:** http://www.matrixscience.com/

Mascot Search Results

Peptide View

MS/MS Fragmentation of **GALCLLAEDDSSCEVNGR**
Found in **O76076** in **uni_human_nr**, WISP2_HUMAN WNT1-inducible-signaling pathway protein 2 OS=Homo sapiens GN=WISP2 PE=1 SV=1

Match to Query 7517: 1964.857152 from(655.959660,3+) intensity(9374259.0000) rtinseconds(2158) scans(11659) index(8628)
Title: 130809_HPL_Human_Plaque_BR2_TR3_15_Spectrum028783_scans__11659_RTINSECONDS=2158
Data file E:\\RAW\\HumanPlaques\\mgf\\T\\1D\\T130809_HPL_Human_Plaque_BR2_TR3_15.mgf

Click mouse within plot area to zoom in by factor of two about that point
Or, [Plot from] [0] to [1200] Da [Full range]
Label all possible matches ○ Label matches used for scoring ◉
Show Y-axis ☐

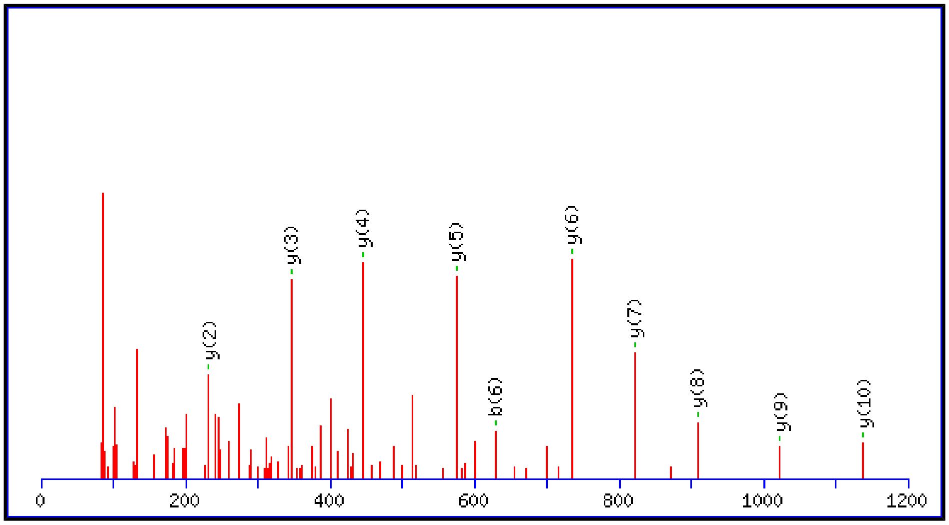

Monoisotopic mass of neutral peptide Mr(calc): 1964.8568
Fixed modifications: Carbamidomethyl (C) (apply to specified residues or termini only)
Ions Score: 84 **Expect:** 1.2e-007
Matches : 10/152 fragment ions using 11 most intense peaks (help)

#	b	b^{++}	b^{*}	b^{*++}	b^{0}	b^{0++}	Seq.	y	y^{++}	y^{*}	y^{*++}	y^{0}	y^{0++}	#
1	58.0287	29.5180					G							18
2	129.0659	65.0366					A	1908.8426	954.9249	1891.8161	946.4117	1890.8320	945.9197	17
3	242.1499	121.5786					L	1837.8055	919.4064	1820.7789	910.8931	1819.7949	910.4011	16
4	402.1806	201.5939					C	1724.7214	862.8644	1707.6949	854.3511	1706.7109	853.8591	15
5	515.2646	258.1360					L	1564.6908	782.8490	1547.6642	774.3358	1546.6802	773.8437	14
6	**628.3487**	314.6780					L	1451.6067	726.3070	1434.5802	717.7937	1433.5961	717.3017	13
7	699.3858	350.1965					A	1338.5226	669.7650	1321.4961	661.2517	1320.5121	660.7597	12
8	828.4284	414.7178			810.4178	405.7126	E	1267.4855	634.2464	1250.4590	625.7331	1249.4750	625.2411	11
9	943.4553	472.2313			925.4448	463.2260	D	***1138.4429***	569.7251	1121.4164	561.2118	1120.4324	560.7198	10
10	1058.4823	529.7448			1040.4717	520.7395	D	***1023.4160***	512.2116	1006.3894	503.6984	1005.4054	503.2064	9
11	1145.5143	573.2608			1127.5038	564.2555	S	***908.3891***	454.6982	891.3625	446.1849	890.3785	445.6929	8
12	1232.5463	616.7768			1214.5358	607.7715	S	***821.3570***	411.1822	804.3305	402.6689	803.3465	402.1769	7
13	1392.5770	696.7921			1374.5664	687.7869	C	***734.3250***	367.6661	717.2985	359.1529	716.3144	358.6609	6
14	1521.6196	761.3134			1503.6090	752.3081	E	***574.2944***	287.6508	557.2678	279.1375	556.2838	278.6455	5
15	1620.6880	810.8476			1602.6774	801.8424	V	***445.2518***	223.1295	428.2252	214.6162			4
16	1734.7309	867.8691	1717.7044	859.3558	1716.7204	858.8638	N	***346.1833***	173.5953	329.1568	165.0820			3

**17**	1791.7524	896.3798	1774.7258	887.8666	1773.7418	887.3746	**G**	***232.1404***	116.5738	215.1139	108.0606			**2**
**18**							**R**	175.1190	88.0631	158.0924	79.5498			**1**

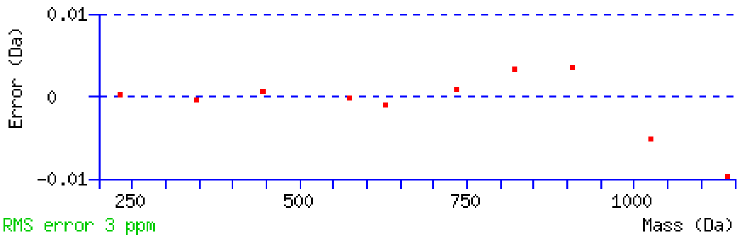

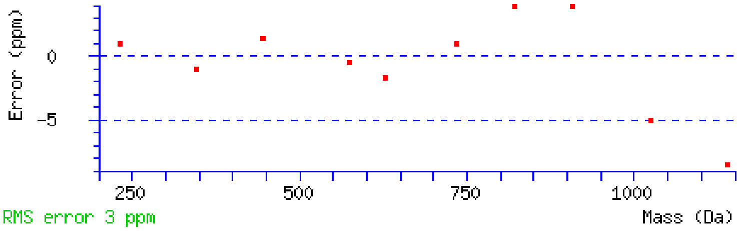

NCBI **BLAST** search of GALCLLAEDDSSCEVNGR
(Parameters: blastp, nr protein database, expect=20000, no filter, PAM30)
Other BLAST web gateways

All matches to this query

Score	Mr(calc)	Delta	Sequence
83.8	1964.8568	0.0004	GALCLLAEDDSSCEVNGR

**Mascot:** http://www.matrixscience.com/
