# Supplementary material for: Monocyte adhesion to atherosclerotic matrix proteins is enhanced by Asn-Gly-Arg deamidation
Source: Sci Rep. 2017 Jul 18;7:5765. doi: 10.1038/s41598-017-06202-2 (PMC5515959; doi:10.1038/s41598-017-06202-2)
Supplement: Supplementary file 2 — Supplimentary Figures [file 41598_2017_6202_MOESM2_ESM.pdf]

Monocyte adhesion to atherosclerotic matrix proteins is enhanced by Asn-Gly-Arg deamidation

Bamaprasad Dutta[1a], Jung Eun Park[1a], Subodh Kumar[1], Piliang Hao[1], Xavier Gallart-Palau[1], Aida Serra[1], Yan Ren[1], Vitaly Sorokin[2,3],Chuen Neng Lee[2,3], Hee Hwa Ho[4], Dominique de Kleijn[3], and Siu Kwan Sze[1*]

[1]School of Biological Sciences, Nanyang Technological University, 60 Nanyang Drive, Singapore 637551.

[2]Department of Surgery, Yong Loo Lin School of Medicine, National University of Singapore, Singapore 119228.

[3]Department of Cardiac, Thoracic and Vascular Surgery, National University Heart Centre, National University Health System, Singapore 119228.

[4]Department of Cardiology, Tan Tock Seng Hospital, 11 Jalan Tan Tock Seng, Singapore 308433.

[a] These authors contributed equally to this work.

Running Title: IsoDGR enhances monocyte adhesion to ECM proteins

Deamidated VEGYSGTAGDSMAYHNGR

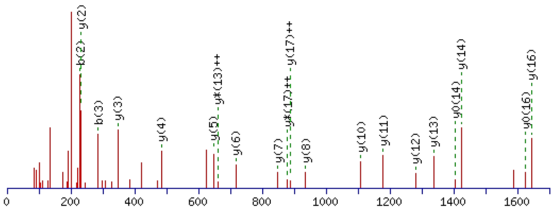

#	b	b^{++}	b^{*}	b^{*++}	b^{0}	b^{0++}	Seq.	y	y^{++}	y^{*}	y^{*++}	y^{0}	y^{0++}	#
1	100.0757	50.5415					V							18
2	**229.1183**	115.0628			211.1077	106.0575	E	1773.7133	887.3603	1756.6868	878.8470	1755.7027	878.3550	17
3	**286.1397**	143.5735			268.1292	134.5682	G	***1644.6707***	822.8390	1627.6442	814.3257	1626.6601	813.8337	16
4	449.2031	225.1052			431.1925	216.0999	Y	1587.6492	794.3283	1570.6227	785.8150	1569.6387	785.3230	15
5	536.2351	268.6212			518.2245	259.6159	S	***1424.5859***	712.7966	1407.5594	704.2833	1406.5754	703.7913	14
6	593.2566	297.1319			575.2460	288.1266	G	***1337.5539***	669.2806	1320.5273	660.7673	1319.5433	660.2753	13
7	694.3042	347.6558			676.2937	338.6505	T	***1280.5324***	640.7699	1263.5059	632.2566	1262.5219	631.7646	12
8	765.3414	383.1743			747.3308	374.1690	A	***1179.4847***	590.2460	1162.4582	581.7327	1161.4742	581.2407	11
9	822.3628	411.6851			804.3523	402.6798	G	***1108.4476***	554.7275	1091.4211	546.2142	1090.4371	545.7222	10
10	937.3898	469.1985			919.3792	460.1932	D	1051.4262	526.2167	1034.3996	517.7034	1033.4156	517.2114	9
11	1024.4218	512.7145			1006.4112	503.7093	S	***936.3992***	468.7033	919.3727	460.1900	918.3887	459.6980	8
12	1155.4623	578.2348			1137.4517	569.2295	M	***849.3672***	425.1872	832.3406	416.6740			7
13	1226.4994	613.7533			1208.4888	604.7481	A	***718.3267***	359.6670	701.3002	351.1537			6
14	1389.5627	695.2850			1371.5522	686.2797	Y	***647.2896***	324.1484	630.2631	315.6352			5
15	1526.6216	763.8145			1508.6111	754.8092	H	***484.2263***	242.6168	467.1997	234.1035			4
16	1641.6486	821.3279	1624.6220	812.8147	1623.6380	812.3226	N	***347.1674***	174.0873	330.1408	165.5740			3
17	1698.6700	849.8387	1681.6435	841.3254	1680.6595	840.8334	G	***232.1404***	116.5738	215.1139	108.0606			2
18							R	175.1190	88.0631	158.0924	79.5498			1

Non-deamidated VEGYSGTAGDSMAYHNGR

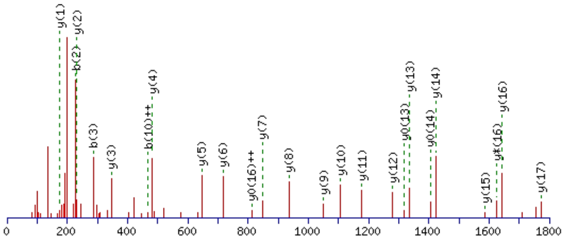

#	b	b^{++}	b^{*}	b^{*++}	b^{0}	b^{0++}	Seq.	y	y^{++}	y^{*}	y^{*++}	y^{0}	y^{0++}	#
1	100.0757	50.5415					V							18
2	**229.1183**	115.0628			211.1077	106.0575	E	***1772.7293***	886.8683	1755.7027	878.3550	1754.7187	877.8630	17
3	**286.1397**	143.5735			268.1292	134.5682	G	***1643.6867***	822.3470	1626.6601	813.8337	1625.6761	813.3417	16
4	449.2031	225.1052			431.1925	216.0999	Y	***1586.6652***	793.8363	1569.6387	785.3230	1568.6547	784.8310	15
5	536.2351	268.6212			518.2245	259.6159	S	***1423.6019***	712.3046	1406.5754	703.7913	1405.5913	703.2993	14
6	593.2566	297.1319			575.2460	288.1266	G	***1336.5699***	668.7886	1319.5433	660.2753	1318.5593	659.7833	13
7	694.3042	347.6558			676.2937	338.6505	T	***1279.5484***	640.2778	1262.5219	631.7646	1261.5378	631.2726	12
8	765.3414	383.1743			747.3308	374.1690	A	***1178.5007***	589.7540	1161.4742	581.2407	1160.4902	580.7487	11
9	822.3628	411.6851			804.3523	402.6798	G	***1107.4636***	554.2354	1090.4371	545.7222	1089.4531	545.2302	10
10	937.3898	**469.1985**			919.3792	460.1932	D	***1050.4422***	525.7247	1033.4156	517.2114	1032.4316	516.7194	9
11	1024.4218	512.7145			1006.4112	503.7093	S	***935.4152***	468.2112	918.3887	459.6980	917.4046	459.2060	8
12	1155.4623	578.2348			1137.4517	569.2295	M	***848.3832***	424.6952	831.3566	416.1820			7
13	1226.4994	613.7533			1208.4888	604.7481	A	***717.3427***	359.1750	700.3161	350.6617			6
14	1389.5627	695.2850			1371.5522	686.2797	Y	***646.3056***	323.6564	629.2790	315.1432			5
15	1526.6216	763.8145			1508.6111	754.8092	H	***483.2423***	242.1248	466.2157	233.6115			4
16	1640.6646	820.8359	1623.6380	812.3226	1622.6540	811.8306	N	***346.1833***	173.5953	329.1568	165.0820			3
17	1697.6860	849.3467	1680.6595	840.8334	1679.6755	840.3414	G	***232.1404***	116.5738	215.1139	108.0606			2
18							R	***175.1190***	88.0631	158.0924	79.5498			1

Figure S1: MS/MS spectra of TNC peptides showing both the deamidated variant (VEGYSGTAGDSMAYH**N**#GR) and native form (VEGYSGTAGDSMAYH**N**GR) as annotated by Mascot database search software. The deamidation site was identified with high confidence as being located between sequences (y2–y8, y10–y14 and y6). Each of the fragmented y-ions containing a deamidation site (y2 – y8) displayed a characteristic mass increase of 0.984 Da compared with the unmodified peptide.

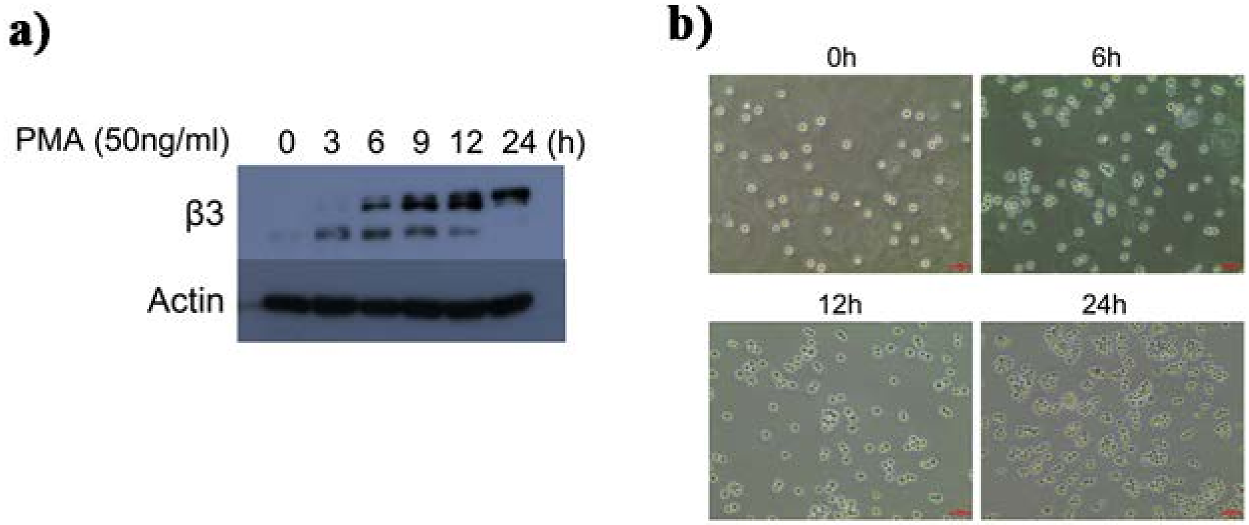

Figure S2. (a) Western blot analysis of integrin β3 and β-Actin protein expression in U937 pre-monocytic cells stimulated with 50 ng/ml PMA for the indicated times. The full length blot was included in the Next page. (b) Macrophage-like morphology of U937 cells after 6h stimulation with PMA as visualized by microscopy.

Full unedited blot for Figure S2a: Western blot analysis of integrin β3 and β-Actin protein expression in U937 pre-monocytic cells stimulated with 50 ng/ml PMA for the indicated times.

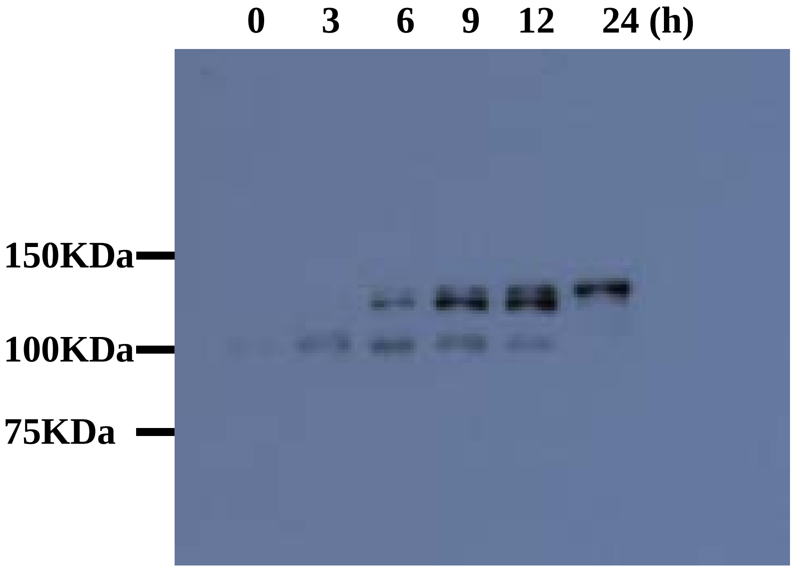

Integrin β3 (30Sec exposer)

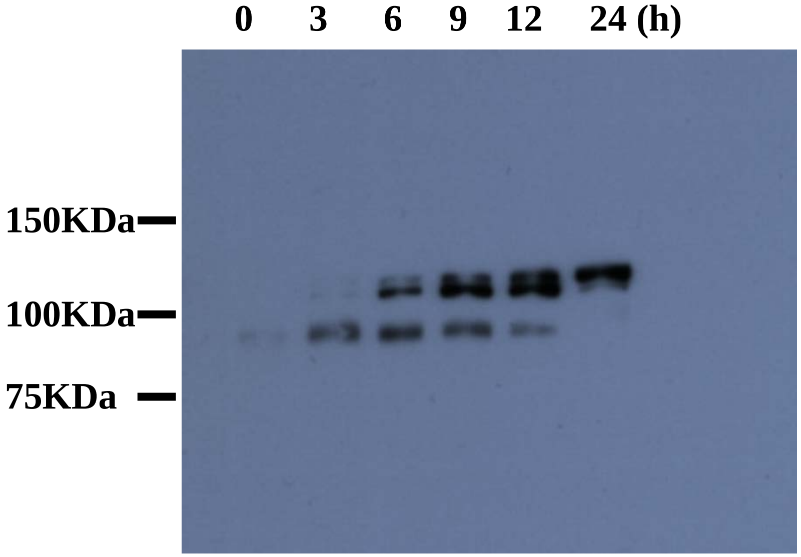

Integrin β3 (1Min exposer)

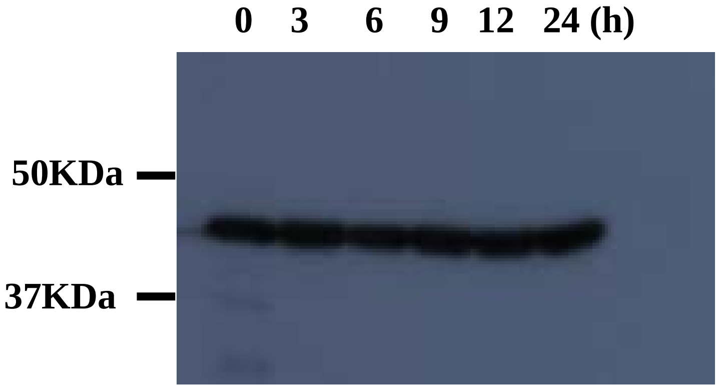

β-Actin
